# Iridium‐Catalyzed Asymmetric Hydrogenation of 2,3‐Diarylallyl Amines with a Threonine‐Derived P‐Stereogenic Ligand for the Synthesis of Tetrahydroquinolines and Tetrahydroisoquinolines

**DOI:** 10.1002/anie.202204300

**Published:** 2022-05-31

**Authors:** Pep Rojo, Medea Molinari, Albert Cabré, Clara García‐Mateos, Antoni Riera, Xavier Verdaguer

**Affiliations:** ^1^ Institute of Research in Biomedicine (IRB Barcelona) The Barcelona Institute of Science and Technology (BIST) Baldiri Reixach 10 08028 Barcelona Spain; ^2^ Departament de Química Inorgànica i Orgànica, Secció Química Orgànica, Universitat de Barcelona Martí i Franquès 1 08028 Barcelona Spain

**Keywords:** Allyl Amines, Asymmetric Hydrogenation, Iridium, P-Stereogenic, Tetrahydroquinolines

## Abstract

Chiral compounds containing nitrogen heteroatoms are fundamental substances for the chemical, pharmaceutical and agrochemical industries. However, the preparation of some of these interesting scaffolds is still underdeveloped. Herein we present the synthesis of a family of P‐stereogenic phosphinooxazoline iridium catalysts from L‐threonine methyl ester and their use in the asymmetric hydrogenation of *N*‐Boc‐2,3‐diarylallyl amines, achieving very high enantioselectivity. Furthermore, the synthetic utility of the 2,3‐diarylpropyl amines obtained is demonstrated by their transformation to 3‐aryl‐tetrahydroquinolines and 4‐benzyl‐tetrahydroisoquinolines, which have not yet been obtained in an enantioselective manner by direct reduction of the corresponding aromatic heterocycles. This strategy allows the preparation of these types of alkaloids with the highest enantioselectivity reported up to date.

## Graphical abstract

A P‐stereogenic phosphinooxazoline iridium catalyst prepared from L‐threonine methyl ester provides very high enantioselectivity in the asymmetric hydrogenation of *N*‐Boc‐2,3‐diarylallyl amines. The synthetic applicability of the resulting 2,3‐diarylpropyl amines is demonstrated with the preparation of 3‐aryl‐tetrahydroquinolines and 4‐benzyl‐tetrahydroisoquinolines, achieving the highest ee values reported to date for this class of compounds.

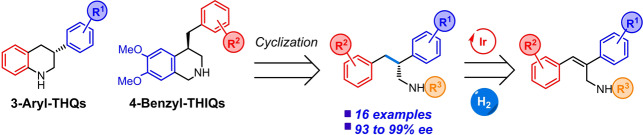

Chiral amines are key structures present in a large number of drugs, natural products, and other biologically active compounds such as agrochemicals.^[1]^ Furthermore, a considerable number of compounds commonly used for diverse synthetic purposes also contain a chiral amine moiety. Thus, the research community has devoted attention to the asymmetric synthesis of chiral amines over the years.^[2]^ Asymmetric hydrogenation is perhaps the most industrially relevant strategy to synthesize chiral amines.^[3]^ However, due to the large chemical space available, the asymmetric hydrogenation of certain types of amine substrates is still underdeveloped. One such family comprises allyl amines, which are challenging substrates because they lack a proper coordinating group.^[4]^

In this regard, the 2,3‐diarylpropyl amine core has shown promising inhibitory activity against biological targets (Figure 1a).[^5^, ^6^] Moreover, cyclization of chiral 2,3‐diarylpropyl amines would grant access to both tetrahydroquinolines (THQs) and tetrahydroisoquinolines (THIQs). These heterocycles are highly relevant substances in the pharmaceutical industry, as reflected by their presence in many different drugs, natural products, and biologically active compounds (Figure 1b).[^7^, ^8^] Despite this, the asymmetric hydrogenation of 2,3‐diarylallyl amines has received little attention.[^4e^, ^9^]

**Figure 1:**
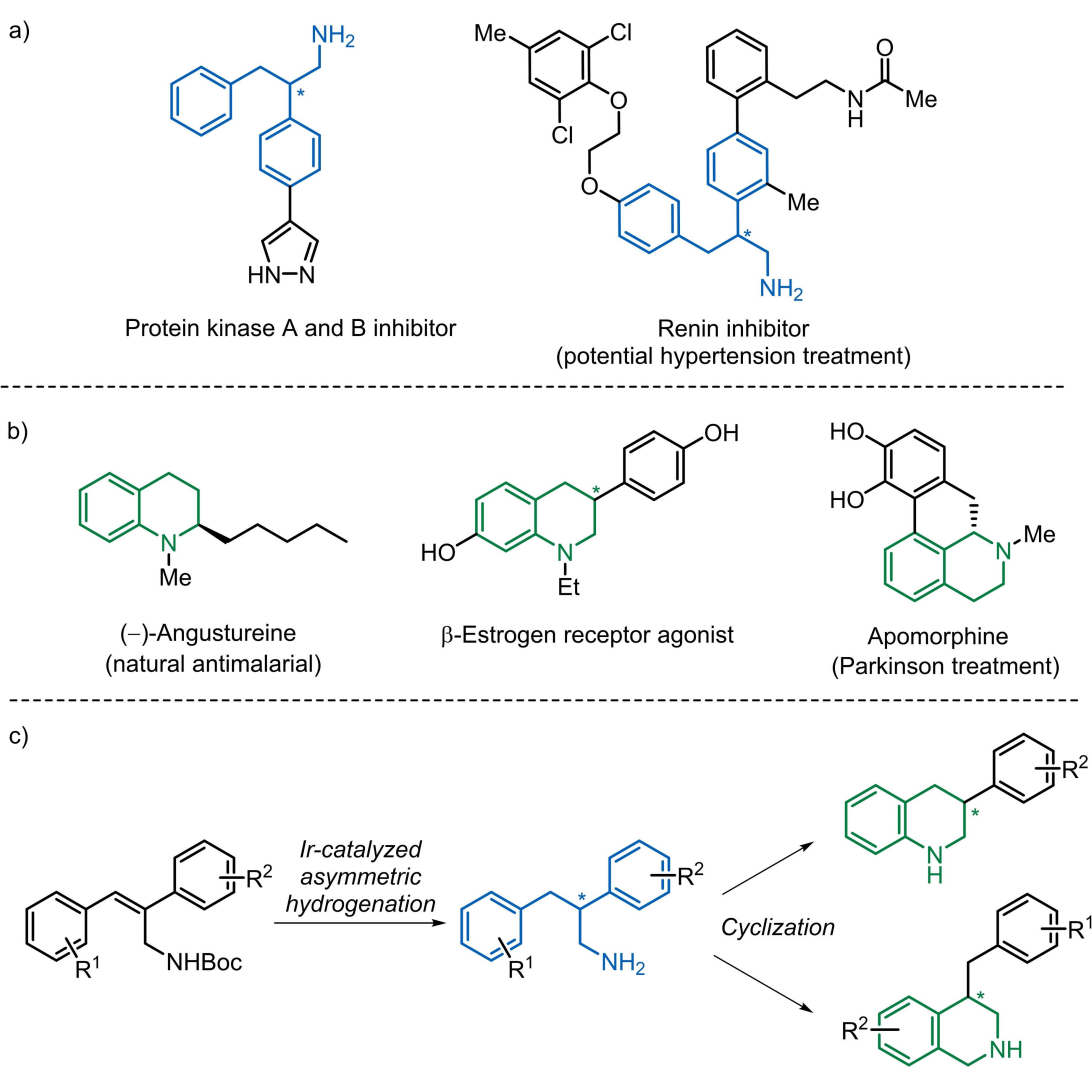
a) Examples of biologically active compounds containing a 2,3‐diarylpropyl amine core. b) Examples of drugs and biologically active compounds with THQ and THIQ cores. c) Strategy envisaged for the preparation of chiral THQs and THIQs.

Since the pioneer work by A. Pfaltz and co‐workers with the Ir‐PHOX catalytic system,^[10]^ phosphinooxazolines emerged as excellent ligands for the iridium‐catalyzed asymmetric hydrogenation of non‐functionalized or minimally functionalized olefins.^[11]^ In the last years, our group has developed P‐stereogenic phosphinooxazoline ligands (MaxPHOX) that show excellent results in the asymmetric hydrogenation of alkene and imine substrates.[^12^, ^13^] In this context, we envisioned applying our expertise to tackle the asymmetric hydrogenation of 2,3‐diarylallyl amines. Herein we report the synthesis of a family of P‐stereogenic phosphinooxazoline iridium catalysts derived from L‐threonine that exhibits selectivity up to 99 % ee in the hydrogenation of *N*‐Boc‐2,3‐diarylallyl amines. We also demonstrate that the resulting free propyl amines can be easily cyclized to the corresponding 3‐aryl‐tetrahydroquinolines and 4‐benzyl‐tetrahydroisoquinolines (Figure 1c).

To study the asymmetric hydrogenation of *N*‐Boc‐2,3‐diarylallyl amines, we devised a strategy for their preparation (Scheme 1). Inspired by a procedure described by Carretero and co‐workers, alkyne **1 a** was subjected to Cu‐catalyzed hydroborylation.^[14]^ The borylation was completely regioselective for the internal position and provided exclusively the *Z*‐alkenyl boronate **2 a**. Suzuki–Miyaura coupling of **2 a** with *p*‐methoxy iodobenzene provided the desired allyl amine **3 a**, which was subsequently used in the catalyst screening study.^[15]^

**Scheme 1:**
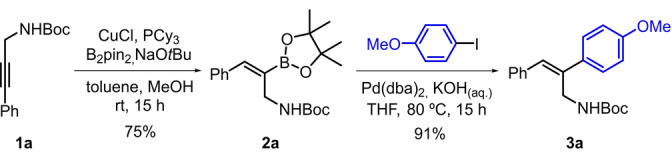
Synthetic strategy of 2,3‐diarylallyl amines **3**, exemplified for **3 a**.

Initially, the asymmetric hydrogenation of **3 a** was attempted with several diastereomeric Ir‐MaxPHOX catalysts previously reported by our group (**16 a**–**c**, Table 1).^[13]^ However, in this case, at 5 mol % and 50 bar of H_2_ in DCM, the conversions were mostly moderate and the highest enantioselectivity was 84 % ee with (*S*_P_,*R*,*S*)‐**16 a** (Table 1, entries 1–4). The replacement of the isopropyl substituent in the oxazoline moiety of the catalyst by a *tert*‐butyl or phenyl group did not lead to any improvement (entries 5, 6).

**Table 1:** Catalyst screening for the asymmetric hydrogenation of **3 a**.^[a]^

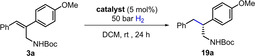			
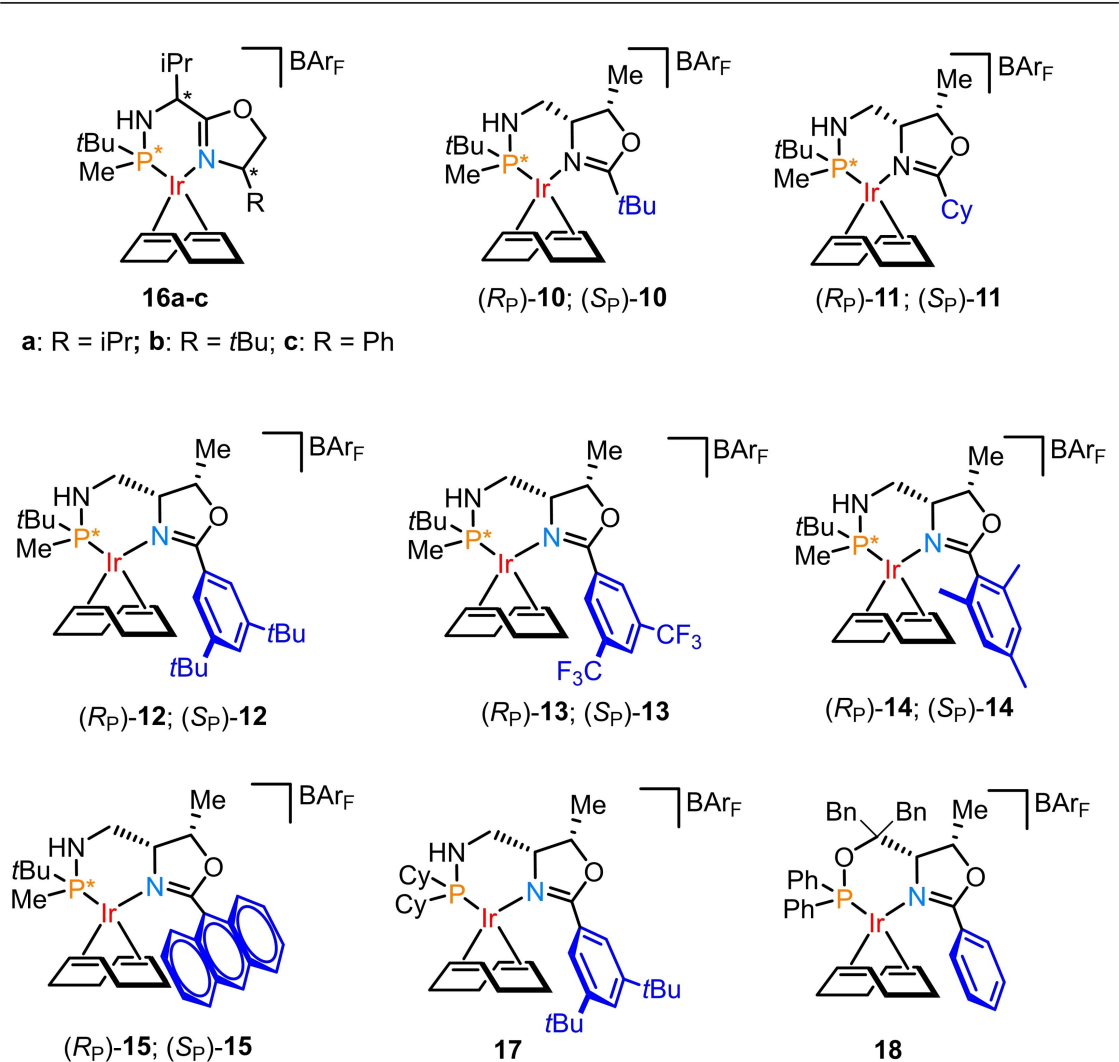			
Entry	Catalyst	Conv. [%]^[b]^	ee [%]^[c]^
1	(*S*_P_,*R*,*S*)‐**16 a**	76	84
2	(*S*_P_,*S*,*S*)‐**16 a**	40	4
3	(*S*_P_,*R*,*R*)‐**16 a**	35	23
4	(*S*_P_,*S*,*R*)‐**16 a**	94	75
5	(*S*_P_,*R*,*S*)‐**16 b**	88	75
6	(*S*_P_,*R*,*S*)‐**16 c**	15	69
7	(*R*_P_)‐**10**	76	13
8	(*S*_P_)‐**10**	50	89
9	(*R*_P_)‐**11**	45	89
10	(*S*_P_)‐**11**	49	71
11	(*R*_P_)‐**12**	99	88
12	(*S*_P_)‐**12**	>99	99
13	(*R*_P_)‐**13**	87	88
14	(*S*_P_)‐**13**	99	94
15	(*R*_P_)‐**14**	19	85
16	(*S*_P_)‐**14**	98	71
17	(*R*_P_)‐**15**	46	84
18	(*S*_P_)‐**15**	86	87
19	**17**	>99	95
20	**18**	70	64

[a] The experiments were carried out at 0.06 M. [b] Conversion was determined by ^1^H NMR analysis of the crude reaction mixtures. [c] The ee values were determined by HPLC analysis on a chiral stationary phase. All experiments yielded the (*R*) product of hydrogenation.

To ameliorate these results, we designed a new family of catalysts derived from commercial L‐threonine methyl ester hydrochloride **4**, following the synthetic procedure shown in Scheme 2.^[16]^ Reaction of aminoalcohol **6** with either (*R*)‐ or (*S*)‐*tert*‐butylmethyl phosphinous mesylate **S2** proved regioselective for the less hindered primary amine and occurred with inversion of configuration at phosphorus, providing the two possible diastereomers of **7** with 98 : 2 d.r.^[17]^ The diversity in the oxazoline ring substituent was introduced by reacting **7** with different acyl chlorides. After cyclization, coordination to iridium and exchange with the BAr_F_ counterion, the resulting catalysts with *tert*‐butyl, cyclohexyl, 3,5‐di‐*tert*‐butylphenyl, 3,5‐bis(trifluoromethyl)phenyl, mesityl and anthracenyl substituents (**10**–**15**) were isolated in the two possible diastereomeric configurations. Of note, the catalyst with a naked phenyl group in the oxazoline provided metallation at the *ortho* position of the aromatic ring, leading to an octahedral iridium complex inactive in hydrogenation reactions.^[18]^

**Scheme 2:**
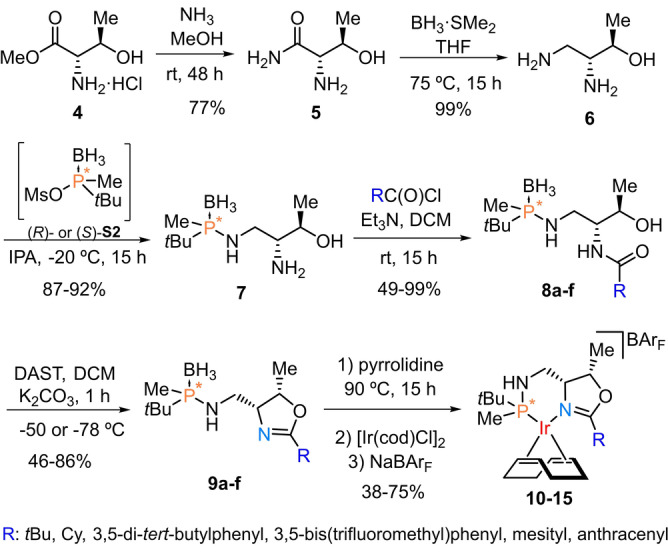
Synthetic procedure of P‐stereogenic iridium catalysts **10**–**15**.

When the family of threonine‐based catalysts was assayed (Table 1, entries 7–18), catalyst (*S*_P_)‐**12** stood out from the rest, showing high activity and 99 % ee (Table 1, entry 12). Figure 2 shows the X‐ray structure of catalyst (*S*_P_)‐**12**.^[19]^ Analysis of the results in Table 1 reveals that the selectivity and also activity of the catalysts are influenced by the configuration of the phosphine fragment. To further check the competitive advantage of having a P‐stereogenic phosphine in the catalyst structure, we synthesized catalyst **17** bearing a non‐chiral P center.^[20]^ Hydrogenation with non‐P‐stereogenic **17** and **18** (ThrePHOX) showed inferior enantioselectivity compared to (*S*_P_)‐**12**, proving that the chiral *tert*‐butylmethyl phosphine moiety is superior in terms of enantioinduction (Table 1, entries 19 and 20).

**Figure 2:**
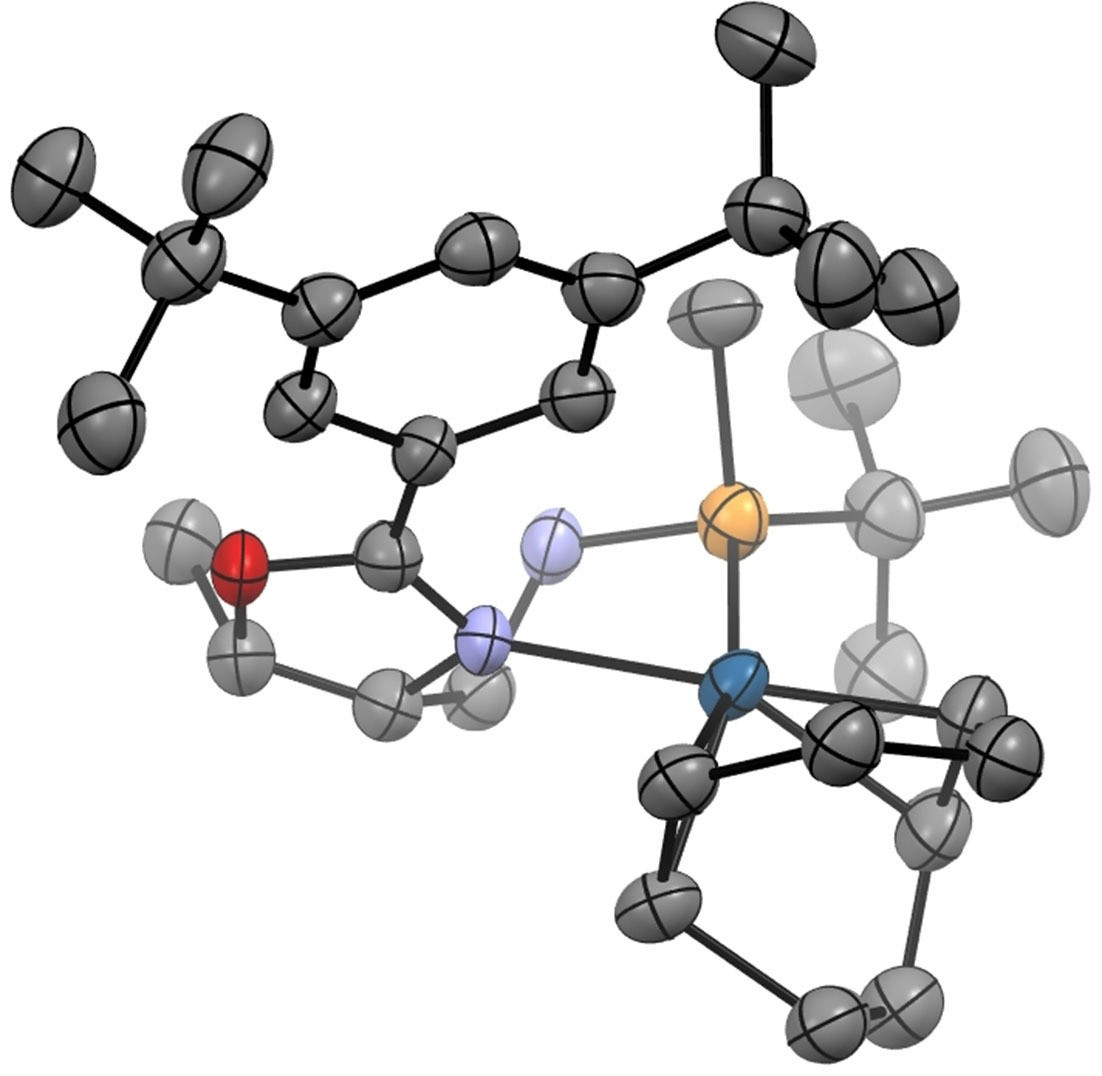
X‐ray structure of (*S*_P_)‐**12**. ORTEP diagram shows thermal ellipsoids at 50 % probability. The BAr_F_ counterion has been omitted for clarity.

Next, we studied the optimization of the hydrogenation parameters with catalyst (*S*_P_)‐**12** and substrate **3 a**. The influence of hydrogen pressure was minimal and similar selectivity was observed between 50 and 1 bar (Table 2, entries 1–3). Regarding the choice of solvent, dichloromethane and 1,2‐dichloroethane (DCE) gave practically the same results (Table 2, entries 1 and 4). α,α,α‐Trifluorotoluene (TFT) also provided comparable selectivity but with a slight loss of activity (Table 2, entry 5). The use of coordinating solvents like THF or weakly coordinating solvents such as EtOAc was detrimental in terms of conversion (Table 2, entries 7 and 8). Most interestingly, the protic solvent 2,2,2‐trifluoroethanol (TFE) provided complete conversion and competitive selectivity (Table 2, entry 9). The asymmetric hydrogenation at 1 mol % under the optimized parameters yielded full conversion and the same enantioselectivity as observed for entry 1 (Table 2, entry 10). Monitoring the rate of the reaction showed a turnover frequency (TOF) of 82 h^−1^. Increase of temperature to 40 °C showed a minimal decrease on the enantioselectivity to 98 % ee (Table 2, entry 11) and raised TOF to >124 h^−1^. Finally, reduction of the catalyst loading to 0.2 mol % also yielded amine **19 a** with complete conversion and 99 % ee (Table 2, entry 12).

**Table 2:** Optimization of pressure, solvent and catalyst loading parameters.^[a]^

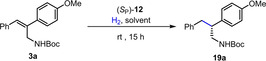					
Entry	H_2_ [bar]	Solvent	Cat. [mol %]	Conv. [%]^[b]^	ee [%]^[c]^
1	50	DCM	5	>99	99
2	15	DCM	5	>99	98
3	1	DCM	5	99	98
4	50	DCE	5	>99	98
5	50	TFT	5	92	98
6	50	Toluene	5	71	96
7	50	THF	5	3	–
8	50	EtOAc	5	35	92
9	50	TFE	5	>99	96
10	50	DCM	1	>99	99
11^[d]^	50	DCM	0.2	>99	98
12^[e]^	50	DCM	0.2	>99	99

[a] The experiments were carried out at 0.06 M unless otherwise specified. [b] Conversion was determined by ^1^H NMR analysis of the crude reaction mixtures. [c] The ee values were determined by HPLC analysis on a chiral stationary phase. [d] The reaction was carried out at 40 °C (4 h) instead. [e] The reaction was carried out at 0.5 M for 24 h.

Following the strategy shown in Scheme 1, we prepared a set of 2,3‐diarylallyl amines with different substitutions in the 2‐aryl ring (**3 a**–**j**), in both aryl rings (**3 k**–**m**), or with a distinct *N*‐protecting group (**3 n**–**p**). In all cases, the *Z*‐olefins were obtained as single regio‐ and stereoisomers. These substrates were subjected to asymmetric hydrogenation at 1 mol % under the optimized conditions (Scheme 3).^[21]^ All olefins bearing *para*‐substituents (**3 a**–**e**) in the 2‐aryl ring gave enantioselectivities ranging from 98 % to 99 % ee. These values were slightly reduced for certain *ortho*‐ and di‐substituted substrates (**3 f**–**j**). Example **3 k** with solely *ortho*‐chloro substitution in the 3‐aryl gave 99 % ee; however, the enantioselectivity slightly decreased when both rings hold some functionalization (**3 l**–**m**). Finally, substrates with a *N*‐tosyl protecting group also provided good results, even with an increase in conversion for **3 p** in comparison to its *N*‐Boc counterpart **3 h**.

**Scheme 3:**
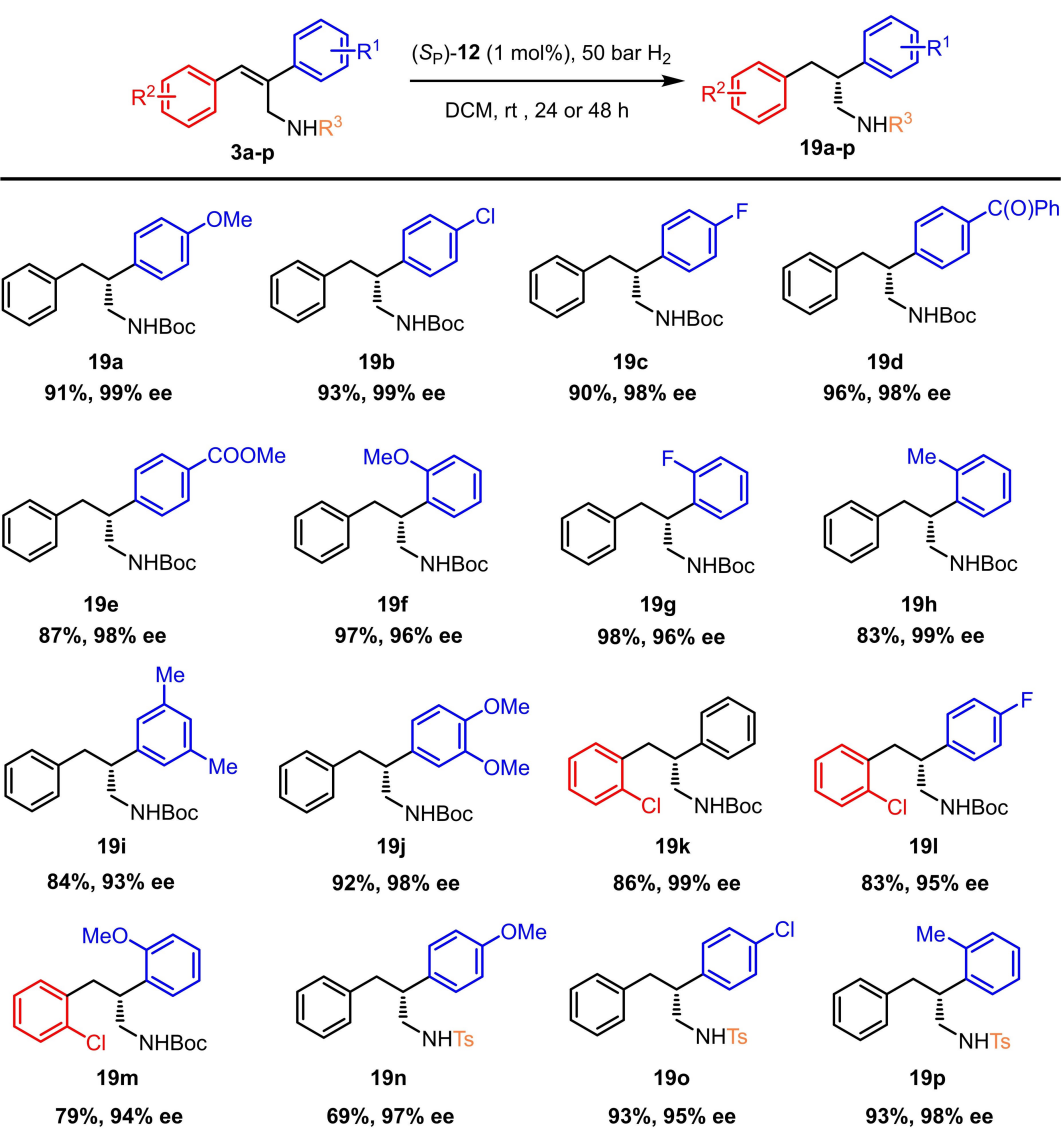
Scope of the catalytic hydrogenation of 2,3‐diarylallyl amines **3**. The reactions were carried out at 0.3 M. Conversion was determined by ^1^H NMR analysis of the crude reaction mixtures. All substrates provided complete conversion except for **3 h** (86 % conv.). The ee values were determined by HPLC analysis on a chiral stationary phase.

With the propyl amines **19** in our hands, we proceeded to demonstrate their utility in the preparation of highly enantioenriched THQs and THIQs, which are important core structures in many natural products and pharmaceuticals.[^7^, ^8^] The metal‐catalyzed asymmetric hydrogenation of the corresponding heteroaromatic quinolines is one of the most straightforward and efficient strategies to obtain the corresponding hydrogenated derivatives.^[22]^ While the metal‐catalyzed asymmetric hydrogenation of 2‐aryl‐quinolines provides high selectivity, hydrogenation of the 3‐phenyl analog yields a racemic tetrahydroquinoline.^[23]^ Also, their reduction by hydrogen transfer with chiral Brønsted acid organocatalysts has been reported.^[24]^ However, this strategy fails to provide high enantioselectivity. In contrast, allyl amines **19 k**–**m**, which hold a 3‐*ortho*‐chlorophenyl, are perfectly suited for cyclization to 3‐phenyl‐THQs. Thus, deprotection of **19 k**–**m** in acidic media yielded the primary amine intermediates **20 k**–**m**, which, upon Buchwald–Hartwig cyclization, efficiently provided the corresponding tetrahydroquinolines **21 k**–**m** (Scheme 4).^[25]^ By comparing the optical rotation of **21 k** with that reported in the literature, we confirmed the (*R*) configuration of the hydrogenated amines **19**.[^24a^, ^26^] To the best of our knowledge, our approach is the most enantioselective pathway to obtain chiral 3‐aryl‐tetrahydroquinolines described to date.^[27]^

**Scheme 4:**
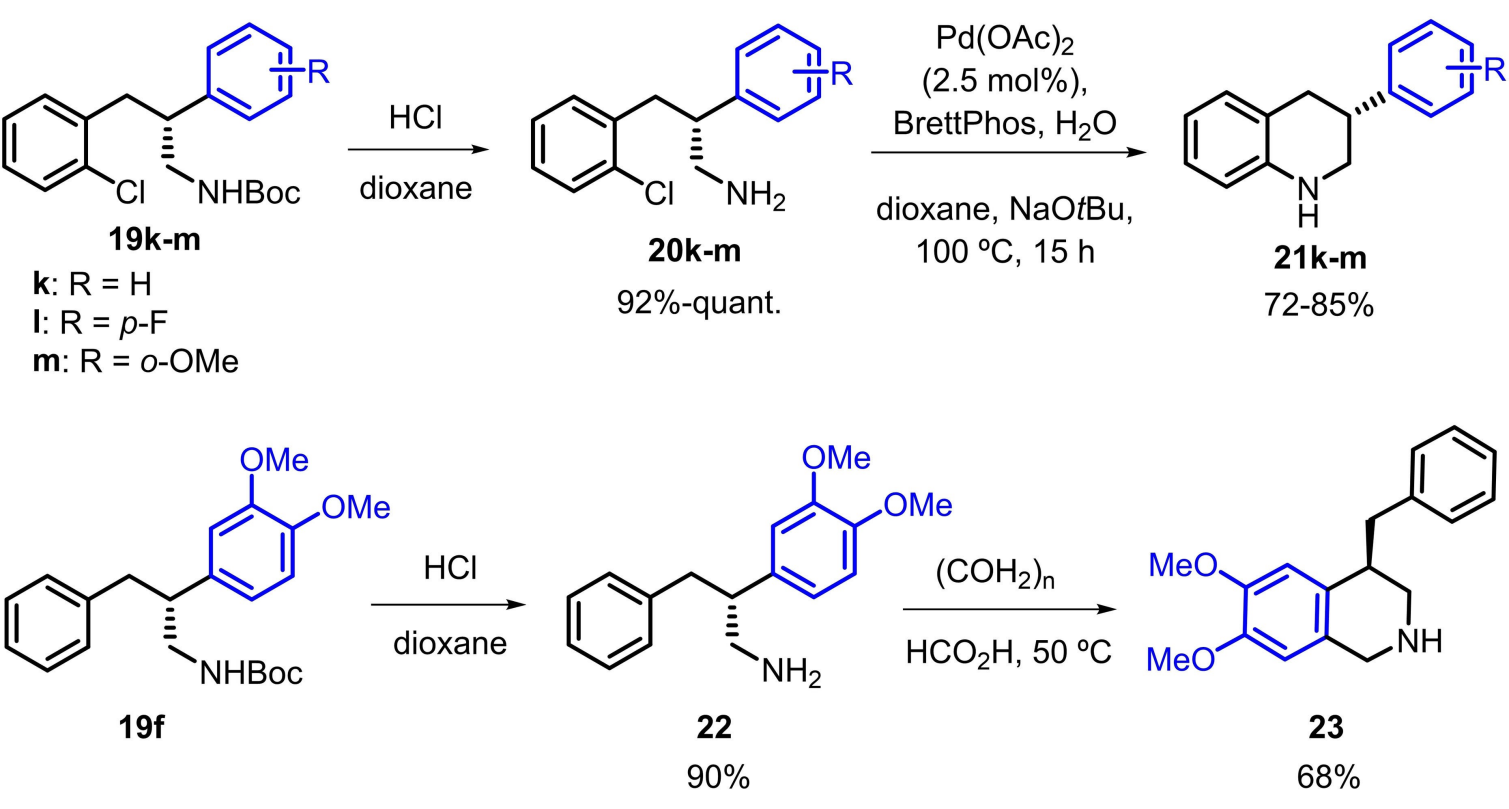
Transformation of hydrogenated amines **19** to THQs **21** and THIQ **23**.

In the same way, the asymmetric hydrogenation of 4‐substituted isoquinolines has not been reported. However, the Pictet–Spengler cyclization of deprotected **22**, bearing an activated aryl ring, allowed the preparation of 4‐benzyl‐tetrahydroisoquinoline **23** in high optical purity (Scheme 4). These applications demonstrate the versatility of the chiral propyl amine intermediates obtained with our methodology.

In summary, we have prepared a family of P‐stereogenic phosphinooxazoline iridium catalysts and identified that (*S*_P_)‐**12** shows excellent performance in the asymmetric hydrogenation of *N*‐Boc‐2,3‐diarylallyl amines. We have also demonstrated that the P‐stereogenic *tert*‐butylmethyl pair in catalyst (*S*_P_)‐**12** provides higher enantioinduction than a non‐chiral phosphine moiety. Additionally, a synthetic procedure to prepare the 2,3‐diarylallyl amine substrates has been developed. The scope of the hydrogenation process has been shown to tolerate different functional groups, substitution in both aryl rings and alternative protecting groups in the amine. The utility of the 2,3‐diarylpropyl amines obtained has been proven by preparing chiral 3‐aryl‐tetrahydroquinolines and 4‐benzyl‐tetrahydroisoquinolines, yielding such alkaloids with the highest enantioselectivity reported to date.

## Conflict of interest

The authors declare no conflict of interest.

## Supporting information

As a service to our authors and readers, this journal provides supporting information supplied by the authors. Such materials are peer reviewed and may be re‐organized for online delivery, but are not copy‐edited or typeset. Technical support issues arising from supporting information (other than missing files) should be addressed to the authors.

Supporting Information

## Data Availability

The data that support the findings of this study are available in the Supporting Information of this article.
