# Supplementary material for: Iridium‐Catalyzed Asymmetric Hydrogenation of 2,3‐Diarylallyl Amines with a Threonine‐Derived P‐Stereogenic Ligand for the Synthesis of Tetrahydroquinolines and Tetrahydroisoquinolines
Source: Angew Chem Int Ed Engl. 2022 May 31;61(29):e202204300. doi: 10.1002/anie.202204300 (PMC9400882; doi:10.1002/anie.202204300)
Supplement: Supplementary file 1 — Supporting Information [file ANIE-61-0-s001.pdf]

Angewandte Chemie
Eine Zeitschrift der Gesellschaft Deutscher Chemiker

Supporting Information

Iridium-Catalyzed Asymmetric Hydrogenation of 2,3-Diarylallyl Amines with a Threonine-Derived P-Stereogenic Ligand for the Synthesis of Tetrahydroquinolines and Tetrahydroisoquinolines

P. Rojo, M. Molinari, A. Cabré, C. García-Mateos, A. Riera, X. Verdaguer**

SUPPORTING INFO

Table of contents

1. General methods S2

2. Experimental procedures and characterization S3

2.1 Preparation of substrates S3

2.1.1 Preparation of 3-aryl propargylic amines **1a-c** S3

2.1.2 Preparation of alkenyl boronates **2a-c** S5

2.1.3 Preparation of 2,3-diarylallyl amines **3a-p** S7

2.2 Preparation of Ir catalysts S15

2.2.1 Preparation of aminophosphines 7 .... S15

2.2.2 Preparation of ligands **9a-f** S18

2.2.3 Preparation of catalysts **10-15** S29

2.2.4 Preparation of dicyclohexylphosphine catalyst **17** S39

2.3 Asymmetric hydrogenation of 2,3-diarylallyl amines **3a-p** S41

2.4 Synthesis of 3-aryl-tetrahydroquinolines **21k-m** S51

2.5 Synthesis of 4-benzyl-tetrahydroisoquinoline **23** S55

2.6 References S56

3. Crystallographic information S57

4. Stereochemical course of the asymmetric hydrogenation. S61

5. ^{1}H-NMR, ^{13}C-NMR and ^{31}P-NMR spectra S62

6. Chiral HPLC chromatographs S158

1. General methods

General procedures and materials

Unless otherwise indicated, materials were obtained from commercial suppliers and used without further purification. All reactions that required anhydrous conditions were performed in dried glassware under inert nitrogen atmosphere. Anhydrous and degassed THF, Et_2O and DCM were taken from a solvent purification system (SPS PS-MD-3). Other anhydrous solvents were purchased from Sigma Aldrich. Benzene-d_6, toluene and pyrrolidine were degassed via freeze-pump-thaw cycles technique. When handling sensitive compounds, the solvent was removed under reduced pressure using a vacuum line. In other cases, a rotary evaporator was used. Silica gel chromatography was performed by using 35-70 mm silica or an automated chromatography system (PuriFlash® 430, Interchim). Employed methodologies that are described in the literature are duly cited. For already characterized compounds, ^{1}H-NMR spectrum is reported.

Instrumentation

NMR spectroscopy: NMR spectra were recorded at 23 °C on the NMR spectrometers of the *Centres Científics i Tecnològics de la Universitat de Barcelona*. The employed spectrometers were Varian Mercury 400 MHz, Varian VNMRS 400 MHz, Varian VNMRS 500 MHz and Bruker 400 MHz. ^{1}H-NMR and ^{13}C-NMR spectra were referenced to internal solvent resonances and reported relatively to TMS. ^{31}P-NMR spectra were reported relatively to phosphoric acid. Chemical shifts (δ) are expressed in ppm and the coupling constants (J) in Hertz (Hz).

HPLC analysis: HPLC analysis was performed on an Agilent Technologies Series 1100 chromatograph with UV detector by Enantia S.L. GC analysis was performed on an Agilent Technologies 6890N with a FID detector by Enantia S.L. The conditions for each analysis are specified in every case.

High Resolution Mass Spectrometry: High resolution ESI-MS spectra were recorded either in an LC/MSD-TOF G1969A (Agilent Technologies) of *Centres Científics i Tecnològics de la Universitat de Barcelona* or in a LTQ-FT Ultra (Thermo Scientific) of Institute for Research in Biomedicine (IRB Barcelona).

IR spectroscopy: IR spectra were recorded in a Thermo Nicolet 6700 FT-IR spectrometer of the *Department de Química Orgànica i Inorgànica* of *Universitat de Barcelona,* using an ATR system. Absorptions are given in wavenumbers (cm^{-1}).

Optical rotations were measured at room temperature (25 °C) using a Jasco P-2000 iRM-800 polarimeter. Concentration is expressed in g/100 mL. The cell sized 10 cm long and had 1 mL of capacity, measuring λ was 589 nm, which corresponds to a sodium lamp.

Melting points were determined using a Büchi M-540 apparatus without recrystallization of the final solids.

2. Experimental procedures and characterization

2.1 Preparation of substrates

General synthetic scheme for the synthesis of substrates **3a-p**:

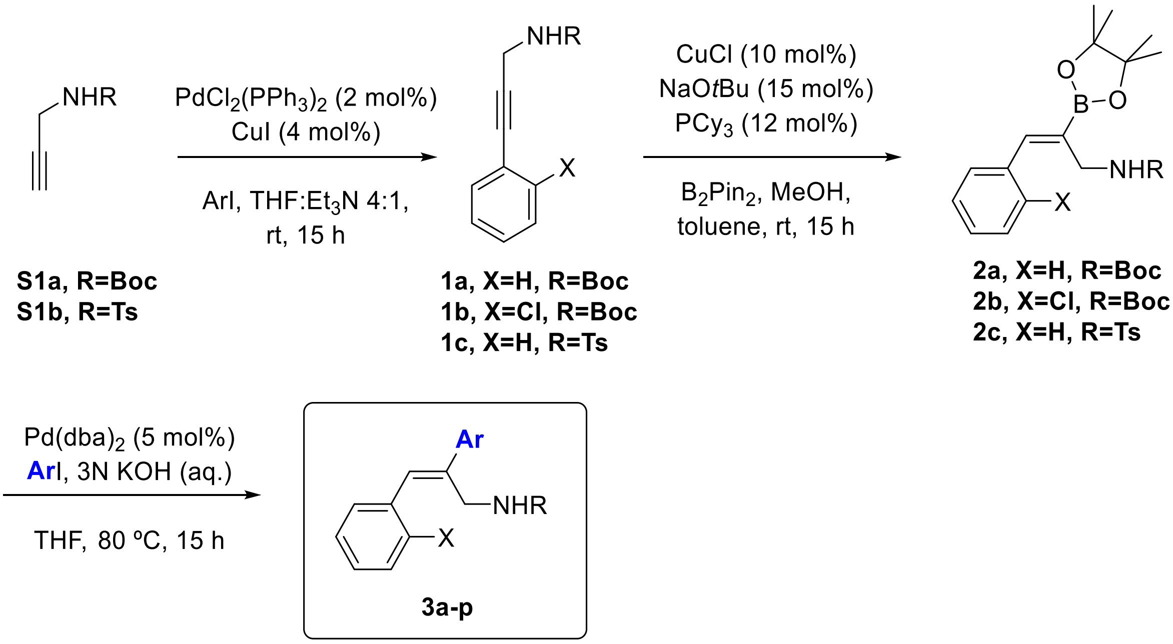

S1a[1] and **S1b**[2] were prepared according to literature procedures from commercially available substrates.

2.1.1 Preparation of 3-aryl propargylic amines 1a-c

General procedure for the preparation of 1a-c (GP1):[3]

A round-bottom flask was charged with CuCl (4 mol%) and $PdCl_2(PPh_3)_2$ (2 mol%), purged with vacuum-nitrogen cycles and dissolved in THF. Then, the corresponding aryl iodide (1.1 eq.) and Et_3N were sequentially added. The mixture was cooled down to 0 °C and a solution of the prepared propargylic amine (1 eq.) was slowly added. The reaction was stirred at room temperature overnight. Afterwards, saturated NH_4Cl aqueous solution was added to the reaction mixture, the organic layer was separated and the aqueous phase was extracted thrice with Et_2O. The combined organic layers

were dried over anhydrous $MgSO_4$, filtered and concentrated under vacuum. The resulting crude was purified by silica column chromatography (hexanes:EtOAc) affording the desired product.

Synthesis of *tert*-butyl (3-phenylprop-2-yn-1-yl)carbamate, 1a

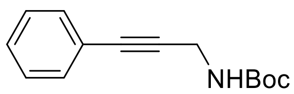

1a

Following GP1, using CuI (76.6 mg, 0.39 mmol, 4 mol%), $PdCl_2(PPh_3)_2$ (141.2 mg, 0.20 mmol, 2 mol%), THF (17 mL), iodobenzene (1.24 mL, 10.84 mmol), Et_3N (4.4 mL) and a solution of **S1a** (1.53 g, 9.86 mmol) in 5 mL of THF. Purification by silica column chromatography (hexanes:EtOAc 9:1) afforded **1a** as white solid (1.88 g, 82% yield). **^{1}H-NMR (400 MHz, $CDCl_3$) δ:** 1.47 (s, 9H), 4.15 (d, *J* = 5.5 Hz, 2H), 4.75 (br s, 1H), 7.27 – 7.32 (m, 3H), 7.39 – 7.43 (m, 2H). The ^{1}H-NMR data agreed with that quoted in the literature.[4]

Synthesis of *tert*-butyl (3-(2-chlorophenylphenyl)prop-2-yn-1-yl)carbamate, 1b

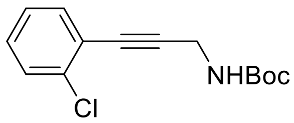

1b

Following GP1, using CuI (121.9 mg, 0.64 mmol, 4 mol%), $PdCl_2(PPh_3)_2$ (229.2 mg, 0.32 mmol, 2 mol%), THF (20.5 mL), 1-chloro-2-iodobenzene (2.15 mL, 17.60 mmol), Et_3N (7.1 mL) and a solution of **S1a** (2.48 g, 16.00 mmol) in 8 mL of THF. Purification by silica column chromatography (hexanes:EtOAc 9:1) afforded **1b** as a white solid (3.42 g, 80% yield). **^{1}H NMR (400 MHz, $CDCl_3$) δ:** 1.47 (s, 9H), 4.21 (d, *J* = 5.6 Hz, 2H), 4.78 (br s, 1H), 7.19 (td, *J* = 7.5, 1.5 Hz, 1H), 7.24 (td, *J* = 7.7, 1.9 Hz, 1H), 7.36 – 7.40 (m, 1H), 7.45 (dd, *J* = 7.3, 2.1 Hz, 1H) ppm. The ^{1}H-NMR data agreed with that quoted in the literature.[5]

Synthesis of 4-methyl-*N*-(3-phenylprop-2-yn-1-yl)benzenesulfonamide, 1c

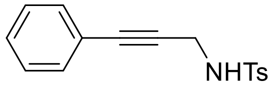

1c

Following GP1, using CuI (56.0 mg, 0.29 mmol, 4 mol%), $PdCl_2(PPh_3)_2$ (102.0 mg, 0.14 mmol, 2 mol%), THF (12 mL), iodobenzene (0.90 mL, 7.88 mmol), Et_3N (4 mL) and a solution of **S1b** (1.50

g, 7.17 mmol) in 4 mL of THF. Purification by silica column chromatography (hexanes:EtOAc 9:1) afforded **1c** as a pale yellow solid (1.31 g, 64% yield). **^{1}H-NMR (400 MHz, $CDCl_3$) δ:** 2.36 (s, 3H), 4.08 (d, *J* = 6.1 Hz, 2H), 4.58 (br s, 1H), 7.11 – 7.16 (m, 2H), 7.22 – 7.25 (m, 1H), 7.27 – 7.32 (m, 4H), 7.78 – 7.84 (m, 2H) ppm. The ^{1}H-NMR data agreed with that quoted in the literature.[6]

2.1.2 Preparation of alkenyl boronates 2a-c

General procedure for the preparation of 2a-c (GP2):[7]

A round-bottom flask was charged with CuCl beads (10 mol%), NaO*t*Bu (15 mol%), tricyclohexylphosphine (12 mol%), B_2pin_2 (1.1 eq.), propargylamine derivative **2a-c** (1 eq.), purged with vacuum-nitrogen cycles and dissolved in toluene (0.38M). Then, the solution was cooled down to 0 °C and MeOH (2 eq.) was added dropwise. The reaction was stirred at room temperature overnight. Afterwards, the solution was cooled down again to 0 °C and quenched by slow addition of MeOH. After 5 minutes, the mixture was filtered through a pad of Celite®, washed with DCM and concentrated under vacuum. The resulting crude was purified by silica column chromatography (equilibrated with hexanes:Et_3N 98:2 and eluted with hexanes:EtOAc) affording the desired product.

Synthesis of alkenyl boronate 2a

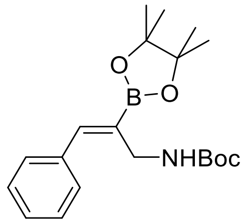

2a

Following GP2, using CuCl beads (40.2 mg, 0.41 mmol, 10 mol%), NaO*t*Bu (60.3 mg, 0.61 mmol, 15 mol%), tricyclohexylphosphine (136.8 mg, 0.49 mmol, 12 mol%), B_2pin_2 (1.13 g, 4.47 mmol), **1a** (938.8 mg, 4.06 mmol), toluene (10.7 mL) and MeOH (0.33 mL, 8.12 mmol). Purification by silica column chromatography (equilibrated with hexanes:Et_3N 98:2 and eluted with hexanes:EtOAc from 9:1 to 1:1) afforded **2a** as a white solid (1.10 g, 75% yield). **M.p.:** 114 – 117 °C. **IR (ATR-FTIR) υ_{max}:** 3419, 2973, 2938, 1710, 1510, 1364 cm^{-1}. **^{1}H-NMR (400 MHz, $CDCl_3$) δ:** 1.32 (s, 12H), 1.43 (s, 9H), 4.05 (br s, 1H), 4.14 (s, 2H), 5.20 (br s, 1H), 7.30 – 7.36 (m, 5H) ppm. **^{13}C-NMR (101 MHz, $CDCl_3$) δ:** 25.0, 28.6, 29.9, 40.3, 79.2, 84.0, 128.0, 128.4, 128.9, 129.4, 144.4, 155.9 ppm. **HRMS (ESI):** calc for $[C_{20}H_{30}BNO_4+H]^+$: 360.2340, found 360.2342.

Synthesis of alkenyl boronate 2b

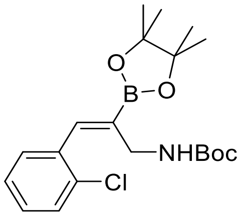

2b

Following GP2, using CuCl beads (48.2 mg, 0.49 mmol, 10 mol%), NaO*t*Bu (72.4 mg, 0.73 mmol, 15 mol%), tricyclohexylphosphine (164.2 mg, 0.58 mmol, 12 mol%), B_2pin_2 (1.36 g, 5.36 mmol), **1b** (1.29 g, 4.87 mmol), toluene (12.8 mL) and MeOH (0.39 mL, 9.74 mmol). Purification by silica column chromatography (equilibrated with hexanes:Et_3N 98:2 and eluted with hexanes:EtOAc from 9:1 to 1:1) afforded **2b** as a white solid (1.58 g, 82% yield). **M.p.:** 96 – 97 °C. **IR (ATR-FTIR) υ_{max}:** 3425, 2976, 2930, 2862, 1709, 1615, 1512 cm^{-1}. **^{1}H-NMR (400 MHz, $CDCl_3$) δ:** 1.32 (s, 12H), 1.42 (s, 9H), 3.98 (s, 2H), 5.12 (br s, 1H), 7.18 – 7.25 (m, 2H), 7.29 – 7.35 (m, 1H), 7.35 – 7.42 (m, 2H) ppm. **^{13}C-NMR (101 MHz, $CDCl_3$) δ:** 24.9, 28.4, 28.6, 40.27, 78.9, 84.1, 126.6, 129.2, 129.5, 130.7, 133.7, 135.2, 141.2, 155.7 ppm. **HRMS (ESI):** calc for $[C_{20}H_{29}BClNO_4+H]^+$: 394.1950, found 394.1951.

Synthesis of alkenyl boronate 2c

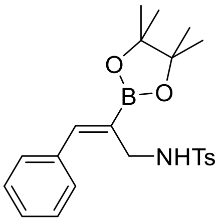

2c

Following GP2, using CuCl beads (53.0 mg, 0.53 mmol, 10 mol%), NaO*t*Bu (76.0 mg, 0.77 mmol, 15 mol%), tricyclohexylphosphine (178.0 mg, 0.63 mmol, 12 mol%), B_2pin_2 (1.45 g, 5.72 mmol), **1c** (1.48 g, 5.18 mmol), toluene (13.6 mL) and MeOH (0.42 mL, 10.36 mmol). Purification by silica column chromatography (equilibrated with hexanes:Et_3N 98:2 and eluted with hexanes:EtOAc from 85:15 to 1:1) afforded **2c** as a white solid (1.03 g, 48% yield). **M.p.:** 128 – 132 °C. **IR (ATR-FTIR) υ_{max}:** 3324, 2976, 2923, 1615, 1449, 1373, 1332 cm^{-1}. **^{1}H-NMR (400 MHz, $CDCl_3$) δ:** 1.25 (s, 12H), 2.40 (s, 3H), 3.94 (dd, *J* = 5.9, 1.5 Hz, 2H), 5.34 (t, *J* = 6.1 Hz, 1H), 7.14 – 7.23 (m, 5H), 7.25 – 7.34 (m, 3H), 7.60 – 7.64 (m, 2H) ppm. **^{13}C-NMR (101 MHz, $CDCl_3$) δ:** 21.5, 24.5, 24.8, 42.7, 84.1,

127.3, 128.1, 128.3, 129.0, 129.4, 136.0, 137.0, 143.0, 145.7 ppm. **HRMS (ESI):** calc for $[C_{22}H_{28}BNO_4+H]^+$: 414.1904, found 414.1914.

2.1.3 Preparation of 2,3-diarylallyl amines 3a-p

General procedure for the preparation of 3a-p (GP3):[8]

A screw-capped vial was charged with $Pd(dba)_2$ (5 mol%) and, if solid, the corresponding aryl iodide (1.1 eq.). The vial was evacuated and purged with nitrogen. Anhydrous THF (7 mL/mmol) and, if liquid, the corresponding ArI, were added to the vial and the reaction was stirred at room temperature for 5 minutes. Then, a solution of the corresponding alkenyl boronate **2a-c** (1 eq.) in THF (2.8 mL/mmol) and an aqueous solution of 3N KOH (1 mL/mmol) were sequentially added. The reaction was heated at 80 °C in an oil bath for 15 h. Afterwards, the mixture was cooled, filtered through a short Celite® pad and washed with DCM. The filtrate was concentrated and the crude was purified by silica column chromatography using hexanes:EtOAc as eluent, affording the desired product.

***tert*-Butyl (Z)-(2-(4-methoxyphenyl)-3-phenylallyl)carbamate, 3a**

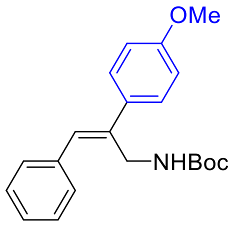

3a

Following GP3, using $Pd(dba)_2$ (48.0 mg, 0.08 mmol), 4-iodoanisole (429.8 mg, 1.83 mmol), THF (16.3 mL), **2a** (600.0 mg, 1.67 mmol) and KOH 3N (1.67 mL). Purification by silica column chromatography (hexanes:EtOAc 9:1) afforded **3a** as a white solid (514.0 mg, 91% yield). **M.p.:** 81 – 83 °C. **IR (ATR-FTIR) υ_{max}:** 3346, 2979, 1669, 1513 cm^{-1}. **^{1}H-NMR (400 MHz, $CDCl_3$) δ:** 1.40 (s, 9H), 3.84 (s, 3H), 4.40 (br s, 3H), 6.88 (s, 1H), 6.90 – 6.95 (m, 2H), 7.27 – 7.33 (m, 3H), 7.35 – 7.40 (m, 2H), 7.47 (d, J = 8.4 Hz, 2H) ppm. **^{13}C-NMR (101 MHz, $CDCl_3$) δ:** 28.4, 39.7, 55.3, 79.4, 114.0, 127.1, 127.7, 128.4, 128.9, 129.6, 132.5, 137.0, 137.3, 155.7, 159.4 ppm. **HRMS (ESI):** calc for $[C_{21}H_{25}NO_3+Na]^+$: 362.1727, found 362.1727.

tert-Butyl (Z)-(2-(4-chlorophenyl)-3-phenylallyl)carbamate, 3b

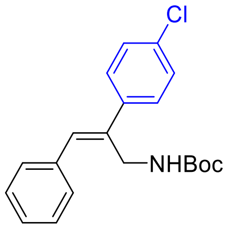

3b

Following GP3, using Pd(dba)$_2$ (20.1 mg, 0.03 mmol), 1-chloro-4-iodobenzene (184.0 mg, 0.77 mmol), THF (7.7 mL), **2a** (251.5 mg, 0.70 mmol) and KOH 3N (0.7 mL). Purification by silica column chromatography (hexanes:EtOAc 9:1) afforded **3b** as a pale yellow solid (191.0 mg, 80% yield). **M.p.:** 94 – 97 °C. **IR (ATR-FTIR) υ_{max}:** 3343, 2973, 2936, 1673, 1519, 1492 cm^{-1}. **^{1}H-NMR (400 MHz, CDCl$_3$) δ:** 1.39 (s, 9H), 4.40 (s, 3H), 6.93 (s, 1H), 7.31 (d, J = 7.5 Hz, 3H), 7.32 – 7.43 (m, 4H), 7.46 (d, J = 8.2 Hz, 2H) ppm. **^{13}C-NMR (101 MHz, CDCl$_3$) δ:** 28.3, 39.6, 79.6, 127.5, 128.4, 128.5, 126.7, 128.79, 129.0, 131.4, 136.5, 137.1, 143.3, 155.5 ppm. **HRMS (ESI):** calc for [$C_{20}H_{22}ClNO_2$+Na]$^+$: 366.1231, found 366.1235.

tert-Butyl (Z)-(2-4-fluorophenyl)-3-phenylallyl)carbamate, 3c

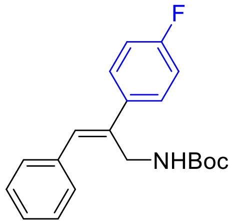

3c

Following GP3, using Pd(dba)$_2$ (20.1 mg, 0.03 mmol), 1-fluoro-4-iodobenzene (171.0 mg, 0.77 mmol), THF (7.7 mL), **2a** (251.5 mg, 0.70 mmol) and KOH 3N (0.7 mL). Purification by silica column chromatography (hexanes:EtOAc 9:1) afforded **3c** as a pale yellow solid (106.0 mg, 47% yield). **M.p.:** 106 – 110 °C. **IR (ATR-FTIR) υ_{max}:** 3352, 2982, 1672, 1518, 1508 cm^{-1}. **^{1}H-NMR (400 MHz, CDCl$_3$) δ:** 1.39 (s, 9H), 4.40 (br s, 3H), 6.89 (s, 1H), 7.07 (t, J = 8.7 Hz, 2H), 7.28 – 7.33 (m, 3H), 7.36 – 7.42 (m, 2H), 7.45 – 7.54 (m, 2H) ppm. **^{13}C-NMR (101 MHz, CDCl$_3$) δ:** 28.3, 39.7, 79.9, 115.4 (d, J_F = 21.3 Hz), 127.4, 128.3 (d, J_F = 8.1 Hz), 128.5, 128.8, 129.0, 131.0, 136.6, 137.2, 155.7, 162.5 (d, J_F = 247.1 Hz) ppm. **HRMS (ESI):** calc for [$C_{20}H_{22}FNO_2$+Na]$^+$: 350.1527, found 350.1527.

tert-Butyl (Z)-(2-(4-benzoylphenyl)-3-phenylallyl)carbamate, 3d

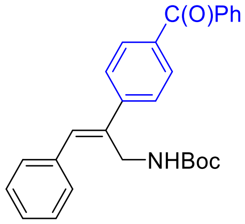

3d

Following GP3, using Pd(dba)$_2$ (20.1 mg, 0.03 mmol), (4-iodophenyl)(phenyl)methanone (237.0 mg, 0.77 mmol), THF (7.7 mL), **2a** (251.5 mg, 0.70 mmol) and KOH 3N (0.7 mL). Purification by silica column chromatography (hexanes:EtOAc 9:1) afforded **3d** as a pale yellow solid (268.0 mg, 93% yield). **M.p.:** 92 – 95 °C. **IR (ATR-FTIR) υ_{max}:** 3333, 2979, 2936, 2368, 1674, 1651, 1598, 1509 cm^{-1}. **^{1}H-NMR (400 MHz, CDCl$_3$) δ:** 1.40 (s, 9H), 4.48 (br s, 3H), 7.07 (s, 1H), 7.29 – 7.38 (m, 3H), 7.38 – 7.45 (m, 2H), 7.50 (t, *J* = 7.6 Hz, 2H), 7.60 (d, *J* = 7.3 Hz, 1H), 7.64 (d, *J* = 7.3 Hz, 2H), 7.80 – 7.88 (m, 4H) ppm. **^{13}C-NMR (101 MHz, CDCl$_3$) δ:** 28.3, 39.5, 79.9, 126.5, 127.7, 128.3 128.4, 128.6, 128.9, 130.0, 130.5, 132.4, 132.8, 136.3, 137.4, 137.6, 144.4, 196.2 ppm. **HRMS (ESI):** calc for [$C_{27}H_{27}NO_3$+Na]$^+$: 436.1883, found 436.1886.

Methyl (*Z*)-4-(3-((*tert*-butoxycarbonyl)amino)-1-phenylprop-1-en-2-yl)benzoate, 3e

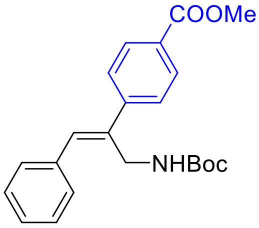

3e

Following GP3, using Pd(dba)$_2$ (20.1 mg, 0.03 mmol), methyl 4-iodobenzoate (202.0 mg, 0.77 mmol), THF (7.7 mL), **2a** (251.5 mg, 0.70 mmol) and KOH 3N (0.7 mL). Purification by silica column chromatography (hexanes:EtOAc 9:1) afforded **3e** as a pale yellow solid (130.0 mg, 51% yield). **M.p.:** 98 – 100 °C. **IR (ATR-FTIR) υ_{max}:** 3349, 2982, 1718, 1671, 1509 cm^{-1}. **^{1}H-NMR (400 MHz, CDCl$_3$) δ:** 1.39 (s, 9H), 3.94 (s, 3H), 4.41 (br s, 1H), 4.45 (s, 2H), 7.03 (s, 1H), 7.29 – 7.35 (m, 3H), 7.38 – 7.42 (m, 2H), 7.59 (d, *J* = 8.2 Hz, 2H), 8.02 – 8.07 (m, 2H) ppm. **^{13}C-NMR (101 MHz, CDCl$_3$) δ:** 28.3, 39.5, 52.1, 79.6, 126.6, 127.7, 128.5, 128.8, 129.3, 129.8, 132.7, 136.3, 137.4, 144.8, 155.6, 166.8 ppm. **HRMS (ESI):** calc for [$C_{22}H_{25}NO_4$+Na]$^+$: 390.1676, found 390.1675.

***tert*-Butyl (Z)-(2-(2-methoxyphenyl)-3-phenylallyl)carbamate, 3f**

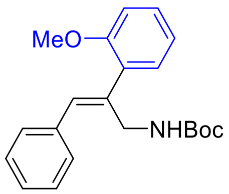

3f

Following GP3, using Pd(dba)$_2$ (20.1 mg, 0.03 mmol), 2-iodoanisole (180.0 mg, 0.77 mmol), THF (7.7 mL), **2a** (251.5 mg, 0.70 mmol) and KOH 3N (0.7 mL). Purification by silica column chromatography (hexanes:EtOAc 9:1) afforded **3f** as a white solid (150.0 mg, 64% yield). **M.p.:** 58 – 61 °C. **IR (ATR-FTIR) υ_{max}:** 3440, 2974, 2243, 1702, 1489 cm^{-1}. **^{1}H-NMR (400 MHz, CDCl$_3$) δ:** 1.32 (s, 9H), 3.86 (s, 3H), 4.37 (d, *J* = 5.5 Hz, 2H), 4.61 (br s, 1H), 6.62 (s, 1H), 6.91 (d, *J* = 8.2, 1H), 6.98 (t, *J* = 7.4 Hz, 1H), 7.26 – 7.33 (m, 3H), 7.34 – 7.41 (m, 4H) ppm. **^{13}C-NMR (101 MHz, CDCl$_3$) δ:** 28.3, 41.3, 55.5, 79.2, 110.6, 114.2, 116.3, 120.9, 127.0, 128.3, 128.9, 129.0, 130.7, 131.7, 155.7 ppm. **HRMS (ESI):** calc for [$C_{21}H_{25}NO_3$+H]$^+$: 340.1907, found 340.1900.

***tert*-Butyl (Z)-(2-(2-fluorophenyl)-3-phenylallyl)carbamate, 3g**

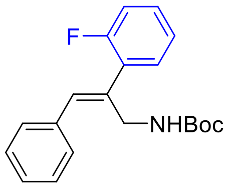

3g

Following GP3, using Pd(dba)$_2$ (20.1 mg, 0.03 mmol), 1-fluoro-2-iodobenzene (171.0 mg, 0.77 mmol), THF (7.7 mL), **2a** (251.5 mg, 0.70 mmol) and KOH 3N (0.7 mL). Purification by silica column chromatography (hexanes:EtOAc 9:1) afforded **3f** as a yellow oil (170.0 mg, 75% yield). **IR (ATR-FTIR) υ_{max}:** 3443, 2976, 2243, 1701, 1494 cm^{-1}. **^{1}H-NMR (400 MHz, CDCl$_3$) δ:** 1.32 (s, 9H), 4.38 (d, *J* = 5.9 Hz, 2H), 4.52 (br s, 1H), 6.76 (s, 1H), 7.04 – 7.12 (m, 1H), 7.11 – 7.20 (m, 1H), 7.25 – 7.34 (m, 2H), 7.33 – 7.43 (m, 5H) ppm. **^{13}C-NMR (101 MHz, CDCl$_3$) δ:** 28.2, 40.9, 79.2, 115.6 (d, J_F = 22.7 Hz), 124.2 (d, J_F = 3.5 Hz), 125.4, 127.4, 128.4, 129.0, 129.1 (d, J_F = 8.2 Hz), 130.7 (d, J_F = 4.0 Hz), 133.2, 136.3, 143.3, 155.4, 160.1 (d, J_F = 246.2 Hz) ppm. **HRMS (ESI):** calc for [$C_{20}H_{22}FNO_2$+Na]$^+$: 350.1527, found 350.1532.

tert-Butyl (Z)-(3-phenyl-2-(_o_-tolyl)allyl)carbamate, 3h

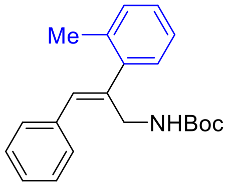

3h

Following GP3, using Pd(dba)$_2$ (20.1 mg, 0.03 mmol), 1-iodo-2-methylbenzene (168.0 mg, 0.77 mmol), THF (7.7 mL), **2a** (251.5 mg, 0.70 mmol) and KOH 3N (0.7 mL). Purification by silica column chromatography (hexanes:EtOAc 9:1) afforded **3h** as a white solid (181.0 mg, 80% yield). **M.p.:** 117 – 119 °C. **IR (ATR-FTIR) υ_{max}:** 3447, 2979, 2251, 1701, 1495 cm^{-1}. **^{1}H-NMR (400 MHz, CDCl$_3$) δ:** 1.33 (s, 9H), 2.38 (s, 3H), 4.33 (s, 3H), 6.51 (s, 1H), 7.17 – 7.24 (m, 4H), 7.27 – 7.31 (m, 1H), 7.34 – 7.40 (m, 3H), 7.41 – 7.44 (m, 1H) ppm. **^{13}C-NMR (101 MHz, CDCl$_3$) δ:** 19.9, 28.3, 41.5, 79.2, 125.6, 127.2, 127.4, 128.4, 128.9, 129.0, 129.1, 130.3, 131.7, 135.8, 136.5, 143.3, 155.5 ppm. **HRMS (ESI):** calc for [$C_{21}H_{25}NO_2$+Na]$^+$: 346.1778, found 346.1778.

tert-Butyl (Z)-(2-(3,5-dimethylphenyl)-3-phenylallyl)carbamate, 3i

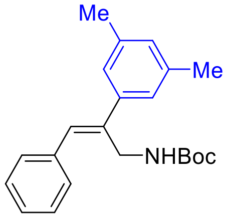

3i

Following GP3, using Pd(dba)$_2$ (20.1 mg, 0.03 mmol), 1-iodo-3,5-dimethylbenzene (179.0 mg, 0.77 mmol), THF (7.7 mL), **2a** (251.5 mg, 0.70 mmol) and KOH 3N (0.7 mL). Purification by silica column chromatography (hexanes:EtOAc 9:1) afforded **3i** as a white solid (170.0 mg, 72% yield). **M.p.:** 84 – 86 °C. **IR (ATR-FTIR) υ_{max}:** 3343, 2982, 2924, 1668, 1518, 1321 cm^{-1}. **^{1}H-NMR (400 MHz, CDCl$_3$) δ:** 1.40 (s, 9H), 2.35 (s, 6H), 4.41 (s, 3H), 6.92 (br s, 1H), 6.97 (s, 1H), 7.13 (s, 2H), 7.26 – 7.34 (m, 3H), 7.35 – 7.40 (m, 2H) ppm. **^{13}C-NMR (101 MHz, CDCl$_3$) δ:** 21.4, 28.3, 39.8, 79.3, 124.5, 127.2, 128.4, 128.8, 129.5, 130.7, 136.9, 138.0, 138.01, 140.1, 155.6 ppm. **HRMS (ESI):** calc for [$C_{21}H_{27}NO_2$+Na]$^+$: 360.1934, found 360.1942.

***tert*-Butyl (Z)-(2-(3,4-dimethoxyphenyl)-3-phenylallyl)carbamate, 3j**

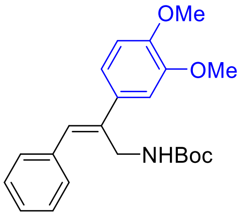

3j

Following GP3, using Pd(dba)$_2$ (72.0 mg, 0.13 mmol), 3,4-dimethoxy-1-iodobenzene (0.44 mL, 2.76 mmol), THF (25 mL), **2a** (900.0 mg, 2.51 mmol) and KOH 3N (2.5 mL). Purification by silica column chromatography (cyclohexane:EtOAc 8:2) afforded **3j** as a pale yellow solid (636.2 mg, 69% yield). **M.p.:** 109 – 110 °C. **IR (ATR-FTIR) υ_{max}:** 3265, 3053, 2971, 2949, 2921, 2832, 1698, 1668, 1548, 1519 cm^{-1}. **^{1}H-NMR (400 MHz, CDCl$_3$) δ:** 1.40 (s, 9H), 3.91 (s, 3H), 3.93 (s, 3H), 4.42 (s, 3H), 6.84 – 6.95 (m, 2H), 7.03 – 7.15 (m, 2H), 7.26 – 7.35 (m, 3H), 7.38 (t, *J* = 7.6 Hz, 2H) ppm. **^{13}C-NMR (101 MHz, CDCl$_3$) δ:** 28.3, 39.6, 55.9, 55.9, 79.4, 109.8, 111.1, 118.9, 127.2, 128.5, 128.8, 129.7, 132.8, 136.9, 137.6, 148.9, 148.9, 155.7 ppm. **HRMS (ESI):** calc for [$C_{22}H_{27}NO_4$-*t*Bu]$^+$: 314.1387, found 314.1390.

***tert*-Butyl (Z)-(3-(2-chlorophenyl)-2-phenylallyl)carbamate, 3k**

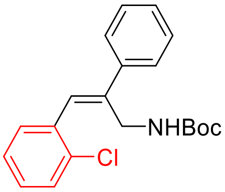

3k

Following GP3, using Pd(dba)$_2$ (99.1 mg, 0.17 mmol), iodobenzene (0.44 mL, 3.79 mmol), THF (34.5 mL), **2b** (1.44 g, 3.45 mmol) and KOH 3N (3.5 mL). Purification by silica column chromatography (hexanes:EtOAc 95:5) afforded **3k** as a pale yellow solid (987.3 mg, 83% yield). **^{1}H-NMR (400 MHz, CDCl$_3$) δ:** 1.37 (s, 9H), 4.32 (d, *J* = 5.2 Hz, 2H), 4.39 (br s, 1H), 6.95 (s, 1H), 7.22 – 7.37 (m, 4H), 7.38 – 7.45 (m, 3H), 7.56 (d, *J* = 7.6 Hz, 2H) ppm. The ^{1}H-NMR data agreed with that quoted in the literature.[5]

***tert*-Butyl (Z)-(3-(2-chlorophenyl)-2-(4-fluorophenyl)allyl)carbamate, 3l**

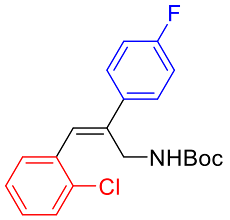

3l

Following GP3, using Pd(dba)$_2$ (103.0 mg, 0.18 mmol), 1-fluoro-2-iodobenzene (0.42 mL, 3.94 mmol), THF (35.5 mL), **2b** (1.50 g, 3.58 mmol) and KOH 3N (3.6 mL). Purification by silica column chromatography (hexanes:EtOAc 95:5) afforded **3l** as a white solid (1.12 g, 87% yield). **M.p.:** 119 – 120 °C. **IR (ATR-FTIR) υ_{max}:** 3322, 2975, 2927, 1672, 1538, 1510 cm^{-1}. **^{1}H-NMR (400 MHz, CDCl$_3$) δ:** 1.37 (s, 9H), 4.29 (d, J = 5.4 Hz, 2H), 4.37 (br s, 1H), 6.88 (s, 1H), 7.08 (t, J = 8.7 Hz, 2H), 7.27 – 7.33 (m, 2H), 7.43 (dd, J = 7.1, 1.8 Hz, 1H), 7.49 – 7.61 (m, 2H) ppm. **^{13}C-NMR (101 MHz, CDCl$_3$) δ:** 28.4, 39.8, 79.7, 115.6 (d, J_F = 21.5 Hz), 126.9, 128.0, 128.7 (d, J_F = 8.0 Hz), 129.0, 129.7, 130.6, 134.1, 135.3, 135.6, 139.0, 155.7, 162.8 (d, J_F = 247.4 Hz) ppm. **HRMS (ESI):** calc for [$C_{20}H_{21}ClFNO_2$-*t*Bu]$^+$: 306.0692, found 306.0699.

***tert*-Butyl (Z)-(3-(2-chlorophenyl)-2-(2-methoxyphenyl)allyl)carbamate, 3m**

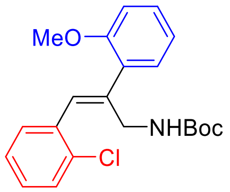

3m

Following GP3, using Pd(dba)$_2$ (68.6 mg, 0.12 mmol), 2-iodoanisole (0.35 mL, 2.63 mmol), THF (23.6 mL), **2b** (1.00 g, 2.39 mmol) and KOH 3N (2.4 mL). Purification by silica column chromatography (hexanes:EtOAc 95:5) afforded **3m** as a white solid (721.0 mg, 81% yield). **M.p.:** 77 – 78 °C. **IR (ATR-FTIR) υ_{max}:** 2987, 2952, 2879, 1716, 1541, 1521, 1507 cm^{-1}. **^{1}H-NMR (400 MHz, CDCl$_3$) δ:** 1.30 (s, 9H), 3.87 (s, 3H), 4.17 – 4.30 (m, 2H), 4.54 (br s, 1H), 6.68 (s, 1H), 6.92 (d, J = 8.2 Hz, 1H), 6.99 (t, J = 6.9 Hz, 1H), 7.18 – 7.25 (m, 1H), 7.26 – 7.30 (m, 1H), 7.29 – 7.37 (m, 2H), 7.43 (dd, J = 17.7, 7.6 Hz, 2H) ppm. **^{13}C-NMR (101 MHz, CDCl$_3$) δ:** 28.4, 41.3, 55.7, 79.0, 110.9, 121.1, 126.7, 128.6, 128.9, 129.3, 129.5, 130.4, 130.9, 131.0, 134.0, 135.3, 140.0, 155.7, 157.0 ppm. **HRMS (ESI):** calc for [$C_{21}H_{24}ClNO_3$+H]$^+$: 374.1517, found 374.1526.

(Z)-N-(2-(4-Methoxyphenyl)-3-phenylallyl)-4-methylbenzenesulfonamide, 3n

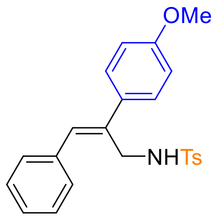

3n

Following GP3, using $Pd(dba)_2$ (20.1 mg, 0.03 mmol), 4-iodoanisole (180.0 mg, 0.77 mmol), THF (7.7 mL), **2c** (251.5 mg, 0.70 mmol) and KOH 3N (0.7 mL). Purification by silica column chromatography (hexanes:EtOAc 85:15) afforded **3n** as a white solid (106.0 mg, 38% yield). **M.p.:** 150 – 152 °C. **IR (ATR-FTIR) υ_{max}:** 3238, 1604, 1515, 1324, 1158 cm^{-1}. **^{1}H-NMR (400 MHz, $CDCl_3$) δ:** 2.46 (s, 3H), 3.83 (s, 3H), 4.15 (d, *J* = 5.5 Hz, 2H), 4.41 (t, *J* = 5.5 Hz, 1H), 6.80 (s, 1H), 6.80 – 6.88 (m, 2H), 7.11 – 7.19 (m, 2H), 7.20 – 7.30 (m, 7H), 7.60 – 7.65 (m, 2H) ppm. **^{13}C-NMR (101 MHz, $CDCl_3$) δ:** 21.6, 42.3, 55.3, 114.2, 127.3, 127.3, 127.6, 128.5, 128.4, 129.6, 130.6, 131.6, 134.9, 136.2, 136.4, 143.4, 159.6 ppm. **HRMS (ESI):** calc for $[C_{23}H_{24}NO_3S+H]^+$: 394.1471, found 394.1471.

(Z)-N-(2-(4-Chlorophenyl)-3-phenylallyl)-4-methylbenzenesulfonamide, 3o

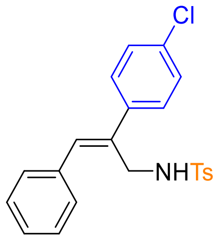

3o

Following GP3, using $Pd(dba)_2$ (20.1 mg, 0.03 mmol), 1-chloro-4-iodobenzene (184.0 mg, 0.77 mmol), THF (7.7 mL), **2c** (251.5 mg, 0.70 mmol) and KOH 3N (0.7 mL). Purification by silica column chromatography (hexanes:EtOAc 85:15) afforded **3o** as a white solid (94.0 mg, 34% yield). **M.p.:** 162 – 165 °C. **IR (ATR-FTIR) υ_{max}:** 3250, 1490, 1417, 1330, 1320, 1156 cm^{-1}. **^{1}H-NMR (400 MHz, $CDCl_3$) δ:** 2.39 (s, 3H), 4.07 (d, *J* = 5.5 Hz, 2H), 4.31 (t, *J* = 5.7 Hz, 1H), 6.78 (s, 1H), 7.06 – 7.11 (m, 2H), 7.10 – 7.24 (m, 9H), 7.53 (d, *J* = 8.2 Hz, 2H) ppm. **^{13}C-NMR (101 MHz, $CDCl_3$) δ:** 21.6, 42.3, 127.2, 127.7, 127.7, 128.5, 128.6, 128.8, 129.6, 132.7, 133.9, 134.6, 135.9, 136.1, 137.9, 143.6 ppm. **HRMS (ESI):** calc for $[C_{22}H_{21}ClNO_2S+H]^+$: 398.0976, found 398.0973.

(*Z*)-4-Methyl-*N*-(3-phenyl-2-(*o*-tolyl)allyl)benzenesulfonamide, 3p

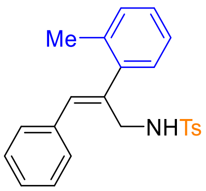

3p

Following GP3, using Pd(dba)$_2$ (20.1 mg, 0.03 mmol), 1-iodo-2-methylbenzene (168.0 mg, 0.77 mmol), THF (7.7 mL), **2c** (251.5 mg, 0.70 mmol) and KOH 3N (0.7 mL). Purification by silica column chromatography (hexanes:EtOAc 85:15) afforded **3p** as a white solid (120.0 mg, 46% yield). **M.p.:** 131 – 133 °C. **IR (ATR-FTIR) ν_{max}:** 3256, 1413, 1327, 1161 cm^{-1}. **^{1}H-NMR (400 MHz, CDCl$_3$) δ:** 2.22 (s, 3H), 2.41 (s, 3H), 4.07 (dd, *J* = 5.8, 1.8 Hz, 2H), 4.24 – 4.36 (m, 1H), 6.49 (s, 1H), 6.97 (dd, *J* =7.4, 1.5 Hz, 1H), 7.09 – 7.22 (m, 7H), 7.27 – 7.34 (m, 3H), 7.50 (d, *J* = 8.3 Hz, 2H) ppm. **^{13}C-NMR (101 MHz, CDCl$_3$) δ:** 19.9, 21.5, 43.8, 126.0, 127.2, 127.50, 127.8, 128.5, 128.6, 129.0, 129.6, 130.6, 133.4, 135.6, 136.0, 136.2, 136.6, 139.8, 143.3 ppm. **HRMS (ESI):** calc for [$C_{23}H_{24}NO_2S$+H]$^+$: 378.1522, found 378.1522.

2.2 Preparation of Ir catalysts

2.2.1 Preparation of aminophosphines 7

The general strategy for the preparation of (R_P)- and (S_P)-**7** is the following:

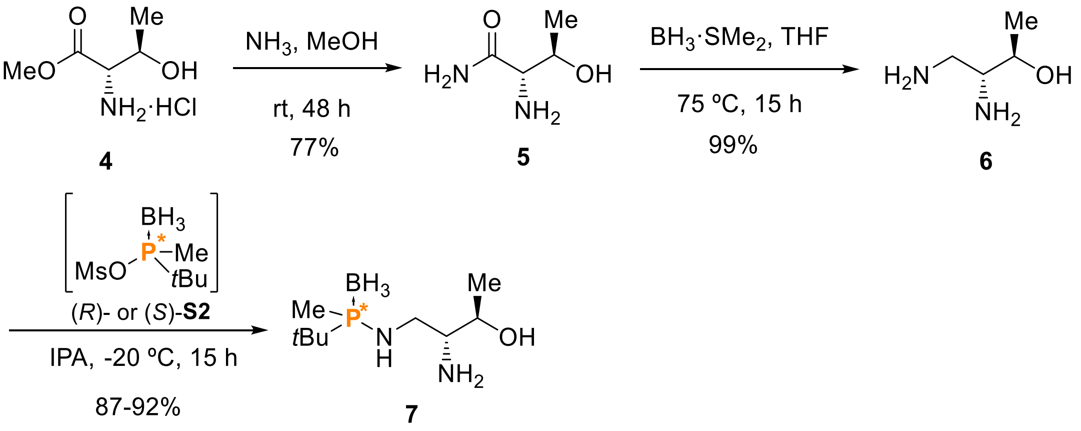

tert-Butylmethyl phosphinous mesylate **S2** was generated *in situ* from the corresponding phosphinous acid salt **S3**, following a procedure developed by our group.[9] Preparation of **S3** was performed as previously described by our group.[9-11] The following Scheme exemplifies the synthesis of (*S*)-**S3**:

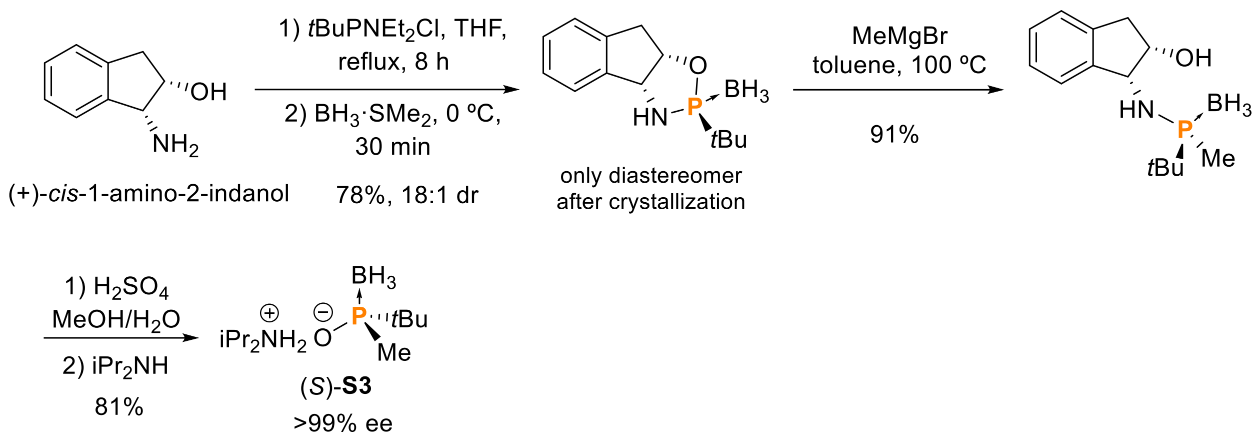

Synthesis of (2*S*,3*R*)-2-amino-3-hydroxybutanamide, 5

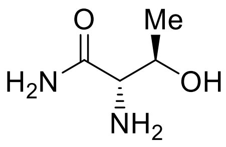

5

Commercial L-threonine methyl ester hydrochloride **4** (6.59 g, 36.91 mmol) was dissolved in 235 mL of MeOH in a 500 mL round-bottom flask. The solution was then cooled down to 0 °C and ammonia was bubbled into the solution for 1.5 h. Then, the ice bath was removed and the solution was allowed to expel the excess of ammonia through a bubbler. The bubbler was exchanged for a nitrogen balloon and the solution was left to stir at room temperature for 48 h. The completion of the reaction was controlled by ^{1}H-NMR. After that, the solution was concentrated under vacuum using a rotary evaporator placed inside a fumehood. The crude was purified by silica column chromatography (DCM:MeOH:$NH_{3(aq.)}$ 75:25:5, equilibrated with DCM:Et_3N 98:2), obtaining **5** as a white solid (3.37g, 77% yield). **M.p.**: 111 – 113 °C. $[\alpha]_D$**:** - 14.1 (c 1.01, $CHCl_3$). **IR (ATR-FTIR) υ_{max}:** 3341, 3302, 3182, 2976, 2909, 2848, 2735, 1667, 1584 cm^{-1}. **^{1}H-NMR (400 MHz, D_2O) δ:** 1.23 (d, *J* = 6.5 Hz, 3H), 3.32 (d, *J* = 4.5 Hz, 1H), 4.04 (qd, *J* = 6.5, 4.5 Hz, 1H). **^{13}C-NMR (101 MHz, D_2O) δ:** 18.4 (s, CH_3), 59.6 (s, CH), 68.6 (s, CH), 178.1 (s, Cq). **HRMS (ESI)**: calc for $[C_4H_{10}N_2O_2+H]^+$: 119.0815, found 119.0813.

Synthesis of (2*R*,3*R*)-3,4-diaminobutan-2-ol, 6

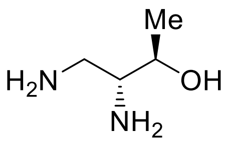

6

A 250 mL two-neck round-bottom flask was charged with **5** (3.30 g, 24.30 mmol) and dissolved in anhydrous THF (51 mL). The solution was cooled down to 0 °C and $BH_3{\cdot}SMe_2$ (9.2 mL, 97.21 mmol) was slowly added. The cooling bath was removed and the reaction was heated at 75°C in an oil bath for 15 h. After that, the solution was cooled down to 0 °C and HCl 6N (18 mL) was added and left to stir until no bubbling was observed. Then, the aqueous solution was evaporated, HCl 6N (18 mL) was added again and the solution was heated at 110 °C in an oil bath for 1 h. The reaction mixture was then filtered, washed twice with DCM and the pH was adjusted to 14 using NaOH pellets. Next, the aqueous phase was evaporated, the residue was dissolved in IPA and the remaining solid was filtered. The solution was evaporated and the solid was washed with hexane to further eliminate the remaining IPA, yielding **6** as a brown oil (2.51 g, 99% yield). **$[\alpha]_D$:** + 1.0 (c 1.02, $CHCl_3$). **IR (ATR-FTIR) υ_{max}:** 3348, 3271, 3179, 2962, 2916, 2867 cm^{-1}. **^{1}H-NMR (400 MHz, D_2O) δ:** 1.19 (d, *J* = 6.5 Hz, 3H), 2.52 (dd, *J* = 12.5, 7.9 Hz, 1H), 2.55 – 2.64 (m, 1H), 2.75 (dd, *J* = 12.5, 4.1 Hz, 1H) 3.75 (qu, *J* = 6.4 Hz, 1H) ppm. **^{13}C-NMR (101 MHz, D_2O) δ:** 18.5 (s, CH_3), 43.3 (s, CH_2), 57.6 (s, CH), 68.8 (s, CH) ppm. **HRMS (ESI)**: calc for $[C_4H_{12}N_2O+H]^+$: 105.1022, found 105.1025.

Synthesis of aminophosphine (R_P)-7

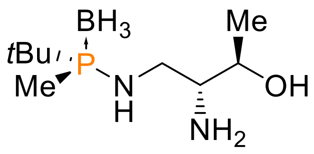

(R_P)-**7**

A solution of methanesulfonic anhydride (1.13 g, 6.32 mmol) in DCM (17 mL) was prepared in a flame-dried 100 mL round-bottom flask and cooled down to -20 °C. Then, phosphinous acid salt (*S*)-**S3** (1.11 g, 6.02 mmol) was dissolved in DCM (3.7 mL) and slowly added to the first solution (for more information regarding the preparation of **S3**, see p. S15). Triethylamine (1.68 mL, 12.05 mmol) was then added dropwise to the mixture and the reaction was stirred at -20 °C for 1.5 h. Afterwards, a solution of **6** (1.35 g, 12.05 mmol) in IPA (9 mL) was slowly added and the reaction was left to stir at the same temperature for 15 h. After that time, NaOH 1M was added and the reaction was allowed to warm up to room temperature. The solution was diluted with DCM, the organic layer was separated and the aqueous phase was extracted twice with DCM. The combined extracts were dried over $MgSO_4$, filtered and concentrated under vacuum, obtaining (R_P)-**7** as a brown oil without need of further purification (1.22 g, 92% yield, 98:2 d.r.). **$[\alpha]_D$:** + 0.7 (c 0.87,

$CHCl_3$). **IR (ATR-FTIR) υ_{max}**: 3345, 2968, 2927, 2869, 2379, 1586 cm^{-1}. **^{1}H-NMR (400 MHz, $CDCl_3$) δ:** 0.03 – 0.91 (m, BH_3), 1.13 (d, J_P = 14.0 Hz, 9H), 1.21 (d, J = 6.3 Hz, 3H), 1.33 (d, J_P = 9.1 Hz, 3H), 1.83 (br s, 3H), 1.95 (s, 1H), 2.58 (dt, J = 7.0, 4.7 Hz, 1H), 2.85 – 2.93 (m, 1H), 3.12 – 3.25 (m, 1H), 3.61 – 3.70 (m, 1H) ppm. **^{13}C-NMR (101 MHz, $CDCl_3$) δ:** 9.7 (d, J_P = 39.9 Hz, CH_3), 20.6 (s, CH_3), 24.9 (d, J_P = 3.0 Hz, $3xCH_3$), 31.5 (d, J_P = 38.0 Hz, Cq), 47.1 (s, CH_2), 58.0 (s, CH), 68.1 (s, CH). **^{31}P-NMR (162 MHz, $CDCl_3$) δ:** 70.4 – 72.3 (m) ppm. **HRMS (ESI)**: calc for $[C_9H_{26}BN_2OP+H]^+$: 221.1950, found 221.1952.

Synthesis of aminophosphine (S_P)-7

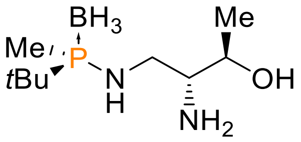

(S_P)-**7**

The reaction was performed following the experimental procedure described above and using methanesulfonic anhydride (1.97 g, 10.96 mmol) in DCM (27.5 mL), (*R*)-**S2** (1.92 g, 10.44 mmol) in DCM (6.5 mL), triethylamine (2.91 mL, 20.88 mmol) and **4** (2.50 g, 20.88 mmol) in IPA (15.2 mL), obtaining (S_P)-**7** as a brown solid without need of further purification (2.00 g, 87% yield, 98:2 d.r.). **M.p.**: 68 – 70 °C. $[\alpha]_D$: + 15.9 (c 1.02, $CHCl_3$). **IR (ATR-FTIR) υ_{max}**: 3338, 2970, 2902, 2869, 2364, 2339, 1585 cm^{-1}. **^{1}H-NMR (400 MHz, $CDCl_3$) δ:** 0.05 – 0.88 (m, BH_3), 1.14 (d, J_P = 13.9 Hz, 9H), 1.20 (d, J = 6.3 Hz, 3H), 1.32 (d, J_P = 9.0 Hz, 3H), 1.89 (br s, 1H), 2.57 (dt, J = 7.3, 4.8 Hz, 1H), 2.94 (dq, J = 13.0, 7.3 Hz, 1H), 3.04 – 3.15 (m, 1H), 3.58 – 3.67 (m, 1H). **^{13}C-NMR (101 MHz, $CDCl_3$) δ:** 9.7 (d, J_P = 39.3 Hz, CH_3), 21.0 (s, CH_3), 24.9 (d, J_P = 3.1 Hz, $3xCH_3$), 31.3 (d, J_P = 38.5 Hz, Cq), 47.4 (s, CH_2), 58.2 (d, J_P = 5.3 Hz, CH), 68.0 (s, CH) ppm. **^{31}P-NMR (162 MHz, $CDCl_3$) δ:** 70.7 – 72.5 (m) ppm. **HRMS (ESI)**: calc for $[C_9H_{26}BN_2OP+H]^+$: 221.1950, found 221.1948.

2.2.2 Preparation of ligands 9a-f

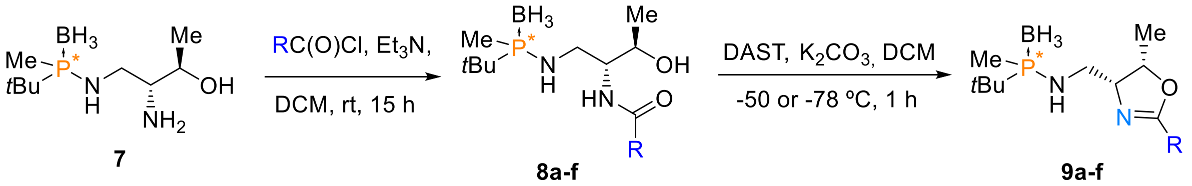

General procedure for the preparation of ligands 9a-f (GP4):

To a solution of **7** (1 eq.) in DCM under inert atmosphere, triethylamine (2 eq.) was slowly added. The solution was then cooled down to 0 °C and the corresponding acid chloride (1 eq.) was added dropwise (if solid, dissolved in DCM). The reaction was left to stir at room temperature for 15 h. Then, HCl 1M was added to the solution, the organic layer was separated and the aqueous phase was extracted twice with DCM. The combined extracts were dried over $MgSO_4$, filtered and concentrated under vacuum, obtaining intermediate **8** without need of further purification. Consecutively, an oven-dried round-bottom flask was charged with **8** (1 eq.), anhydrous K_2CO_3 (4 eq.) and dissolved in DCM. This solution was cooled down to -50 or -78 °C (depending on the solubility of **8** in DCM), another solution of DAST (2 eq.) in DCM was slowly added and the reaction was stirred at the same temperature for 1 h. Then, saturated aqueous $NaHCO_3$ was added, the mixture was warmed up to room temperature, the organic layer was separated and the aqueous phase was extracted twice with DCM. The combined extracts were dried over $MgSO_4$, filtered and concentrated under vacuum. The crude was purified by silica column chromatography using hexanes:EtOAc as eluent, affording the desired product **9**.

Synthesis of (R_P)-9a

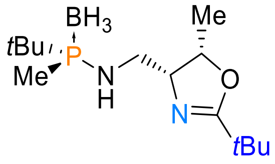

(R_P)-**9a**

Following GP4, using (R_P)-**7** (175.0 mg, 0.76 mmol), DCM (5.7 mL), Et_3N (0.21 mL, 1.53 mmol) and pivaloyl chloride (94 μL, 0.76 mmol) afforded intermediate (R_P)-**8a** as a colourless oil (211.3 mg, 91% yield). **^{1}H-NMR (400 MHz, $CDCl_3$) δ:** 0.06 – 0.91 (m, BH_3), 1.10 (d, J = 14.0 Hz, 9H), 1.16 (d, J = 6.5 Hz, 3H), 1.22 (s, 9H), 1.33 (d, J = 9.2 Hz, 3H), 1.93 (br s, 1H), 2.67 (br s, 1H), 3.02 (dq, J = 13.3, 5.1 Hz, 1H), 3.16 (dq, J = 13.3, 9.4 Hz, 1H), 3.56 – 3.70 (m, 1H), 4.19 (q, J = 6.6 Hz, 1H), 6.13 (d, J = 8.5 Hz, 1H) ppm.

Consecutively, following GP4, (R_P)-**8a** (211.3 mg, 0.69 mmol), K_2CO_3 (384.0 mg, 2.78 mmol), DCM (8.7 mL) and DAST (236.0 mg, 1.39 mmol) were used. Purification by silica column chromatography (hexanes:EtOAc 7:3) afforded (R_P)-**9a** as a colourless oil (141.5 mg, 71% yield). **$[\alpha]_D$:** + 62.7 (c 0.76, $CHCl_3$). **IR (ATR-FTIR) υ_{max}:** 3345, 2972, 2906, 2870, 2381, 2348, 1658, 1645 cm^{-1}. **^{1}H-NMR (400 MHz, $CDCl_3$) δ:** 0.02 – 0.81 (m, BH_3), 1.12 (d, J_P = 13.9 Hz, 9H), 1.20 (s, 9H),

1.27 (d, J = 6.6 Hz, 3H), 1.32 (d, J_P = 9.1 Hz, 3H), 2.02 (br s, 1H), 2.83 – 2.95 (m, 1H), 3.18 – 3.30 (m, 1H), 3.91 – 4.05 (m, 1H), 4.66 (dq, J = 9.4, 6.6 Hz, 1H) ppm. **^{13}C-NMR (101 MHz, CDCl$_3$) δ:** 10.1 (d, J_P = 39.8 Hz, CH$_3$), 14.7 (s, CH$_3$), 24.8 (d, J_P = 3.0 Hz, 3xCH$_3$), 27.9 (s, 3xCH$_3$), 31.4 (d, J_P = 38.8 Hz, Cq), 33.4 (s, Cq), 44.1 (s, CH$_2$), 69.0 (d, J_P = 6.3 Hz, CH), 78.2 (s, CH), 175.5 (s, Cq) ppm. **^{31}P-NMR (162 MHz, CDCl$_3$) δ:** 70.3 (q, J_B = 62.4 Hz) ppm. **HRMS (ESI)**: calc for [$C_{14}H_{32}BN_2OP$+H]$^+$: 287.2418, found 287.2423.

Synthesis of (*S*$_P$)-9a

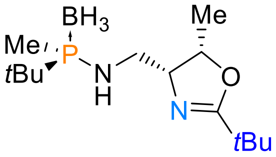

(*S*$_P$)-**9a**

Following GP4, using (*S*$_P$)-**7** (100.0 mg, 0.45 mmol), DCM (4 mL), Et$_3$N (0.13 mL, 0.91 mmol) and pivaloyl chloride (56 μL, 0.45 mmol) afforded intermediate (*S*$_P$)-**8a** as a white solid (138.1 mg, 99% yield). **^{1}H-NMR (400 MHz, CDCl$_3$) δ:** 0.07 – 0.91 (m, BH$_3$), 1.13 (d, J = 14.0 Hz, 9H), 1.17 (d, J = 6.4 Hz, 3H), 1.23 (s, 9H), 1.27 (d, J = 9.1 Hz, 3H), 1.93 (br s, 1H), 2.44 (d, J = 4.5 Hz, 1H), 3.09 – 3.19 (m, 2H), 3.67 – 3.77 (m, 1H), 4.09 (br s, 1H), 6.15 (d, J = 8.2 Hz, 1H) ppm.

Consecutively, following GP4, (*S*$_P$)-**8a** (133.9 mg, 0.44 mmol), K$_2$CO$_3$ (243.3 mg, 1.76 mmol), DCM (5.4 mL) and DAST (141.9 mg, 0.88 mmol) were used. Purification by silica column chromatography (hexanes:EtOAc 7:3) afforded (*S*$_P$)-**9a** as a white solid (108.7 mg, 86% yield). **M.p.**: 79 – 82 °C. **[α]$_D$:** + 94.6 (c 0.96, CHCl$_3$). **IR (ATR-FTIR) υ_{max}**: 3360, 2973, 2904, 2871, 2363, 2338, 1655 cm^{-1}. **^{1}H-NMR (400 MHz, CDCl$_3$) δ:** 0.07 – 0.88 (m, BH$_3$), 1.13 (d, J_P = 14.0 Hz, 9H), 1.20 (s, 9H), 1.28 (d, J = 6.6 Hz, 3H), 1.30 (d, J_P = 8.9 Hz 3H), 1.91 (br s, 1H), 2.92 – 3.05 (m, 1H), 3.08 – 3.19 (m, 1H), 3.93 – 4.04 (m, 1H), 4.57 – 4.75 (m, 1H) ppm. **^{13}C-NMR (101 MHz, CDCl$_3$) δ:** 9.6 (d, J_P = 38.2 Hz, CH$_3$), 14.8 (s, CH$_3$), 24.8 (s, 3xCH$_3$), 27.9 (s, 3xCH$_3$), 31.1 (d, J_P = 39.9 Hz, Cq), 33.3 (s, Cq), 43.7 (s, CH$_2$), 68.5 (d, J_P = 6.9 Hz, CH), 77.9 (s, CH), 174.8 (s, Cq) ppm. **^{31}P-NMR (162 MHz, CDCl$_3$) δ:** 70.1 (q, J_B = 62.4 Hz) ppm. **HRMS (ESI)**: calc for [$C_{14}H_{32}BN_2OP$+H]$^+$: 287.2418, found 287.2420.

Synthesis of (*R*P)-9b

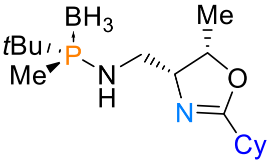

(*R*P)-**9b**

Following GP4, using (*R*P)-7 (125.0 mg, 0.54 mmol), DCM (4.3 mL), Et_3N (0.15 mL, 1.09 mmol) and cyclohexanecarbonyl chloride (74 µL, 0.54 mmol) afforded intermediate (*R*P)-**8b** as a colourless oil (179.1 mg, 99% yield). **^{1}H-NMR (400 MHz, $CDCl_3$) δ:** 0.06 – 0.92 (m, BH_3), 1.10 (d, *J* = 14.0 Hz, 9H), 1.16 (d, *J* = 6.6 Hz, 3H), 1.21 – 1.29 (m, 1H), 1.32 (d, *J* = 9.2 Hz, 3H), 1.37 – 1.97 (m, 10H), 2.06 – 2.18 (m, 1H), 2.73 (d, *J* = 4.8 Hz, 1H), 2.92 – 3.08 (m, 1H), 3.08 – 3.25 (m, 1H), 3.52 – 3.70 (m, 1H), 4.18 (br s, 1H), 5.93 (d, *J* = 8.7 Hz, 1H) ppm.

Consecutively, following GP4, (*R*P)-**8b** (180.1 mg, 0.54 mmol), K_2CO_3 (305.5 mg, 2.21 mmol), DCM (6.8 mL) and DAST (185.0 mg, 1.09 mmol) were used. Purification by silica column chromatography (hexanes:EtOAc 8:2) afforded (*R*P)-**9b** as a colourless oil (111.9 mg, 66% yield). **$[\alpha]_D$:** + 73.5 (c 1.12, $CHCl_3$). **IR (ATR-FTIR) υ_{max}:** 3332, 2971, 2930, 2855, 2372, 2261, 1731, 1641 cm^{-1}. **^{1}H-NMR (400 MHz, $CDCl_3$) δ:** 0.03 – 0.92 (m, BH_3), 1.10 (d, J_P = 13.9 Hz, 9H), 1.20 – 1.46 (m, 4H), 1.24 (d, *J* = 6.7 Hz, 3H), 1.31 (d, J_P = 9.2 Hz, 3H), 1.33 – 1.46 (m, 1H), 1.61 – 1.70 (m, 2H), 1.71 – 1.80 (m, 2H), 1.83 – 1.92 (m, 2H), 2.10 (d, *J* = 9.2 Hz, 1H), 2.26 (tt, *J* = 11.4, 3.5 Hz, 1H), 2.77 – 2.92 (m, 1H), 3.17 – 3. 31 (m, 1H), 3.96 (td, *J* = 9.1, 4.1 Hz, 1H), 4.65 (dq, *J* = 9.4, 6.7 Hz, 1H) ppm. **^{13}C-NMR (101 MHz, $CDCl_3$) δ:** 10.2 (d, J_P = 39.8 Hz, CH_3), 14.8 (s, CH_3), 24.8 (d, J_P = 3.0 Hz, 3xCH_3), 25.7 (s, 2xCH_2), 25.9 (s, CH_2), 28.0 (s, CH_2), 28.0 (s, CH_2), 31.4 (d, J_P = 38.8 Hz, Cq), 37.8 (s, CH), 44.3 (s, CH_2), 69.1 (d, *J* = 5.9 Hz, CH), 77.6 (s, CH), 171.9 (s, Cq) ppm. **^{31}P-NMR (162 MHz, $CDCl_3$) δ:** 69.3 – 71.5 (m) ppm. **HRMS (ESI)**: calc for $[C_{16}H_{34}BN_2OP+H]^+$: 313.2574, found 313.2583.

Synthesis of (*S*P)-9b

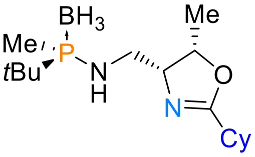

(*S*P)-**9b**

Following GP4, using (*S*P)-7 (125.0 mg, 0.57 mmol), DCM (4.3 mL), Et_3N (0.16 mL, 1.14 mmol) and and cyclohexanecarbonyl chloride (78 µL, 0.57 mmol) afforded intermediate (*S*P)-**8b** as a

colourless oil (175.8 mg, 93% yield). **^{1}H-NMR (400 MHz, $CDCl_3$) δ:** 0.10 – 0.87 (m, 3H), 1.13 (d, J = 14.0 Hz, 9H), 1.17 (d, J = 6.4 Hz, 3H), 1.27 (d, J = 9.0 Hz, 3H), 1.29 – 1.33 (m, 2H), 1.39 – 1.51 (m, 2H), 1.63 – 1.72 (m, 1H), 1.75 – 1.94 (m, 5H), 2.16 (tt, J = 11.7, 3.5 Hz, 1H), 2.45 (d, J = 4.8 Hz, 1H), 3.14 (q, J = 7.0 Hz, 2H), 3.63 – 3.76 (m, 1H), 4.07 (br s, 1H), 5.96 (d, J = 8.6 Hz, 1H) ppm. Consecutively, following GP4, (S_P)-**8b** (174.8 mg, 0.53 mmol), K_2CO_3 (293.0 mg, 2.12 mmol), DCM (6.5 mL) and DAST (180.0 mg, 1.06 mmol) were used. Purification by silica column chromatography (hexanes:EtOAc 8:2) afforded (S_P)-**9b** as a colourless oil (89.1 mg, 54% yield). **$[\alpha]_D$:** + 61.4 (c 1.27, $CHCl_3$). **IR (ATR-FTIR) υ_{max}:** 3360, 2973, 2904, 2871, 2363, 2338, 2265, 1655 cm^{-1}. **^{1}H-NMR (400 MHz, $CDCl_3$) δ:** 0.02 – 0.89 (m, BH_3), 1.12 (d, J_P = 14.0 Hz, 9H), 1.20 – 1.47 (m, 4H), 1.25 (d, J = 6.6 Hz, 3H), 1.28 (d, J_P = 9.0 Hz, 3H), 1.61 – 1.68 (m, 1H), 1.69 – 1.80 (m, 2H), 1.82 – 1.92 (m, 2H), 1.93 – 2.01 (m, 1H), 2.11 (br s, 1H), 2.26 (tt, J = 11.4, 3.6 Hz, 1H), 2.89 – 3.02 (m, 1H), 3.05 – 3. 23 (m, 1H), 3.97 (td, J = 8.9, 4.3 Hz, 1H), 4.64 (dq, J = 9.4, 6.6 Hz, 1H) ppm. **^{13}C-NMR (101 MHz, $CDCl_3$) δ:** 9.7 (d, J_P = 37.9 Hz, CH_3), 14.8 (s, CH_3), 24.8 (d, J_P = 3.1 Hz, 3xCH_3), 25.7 (s, 2xCH_2), 25.9 (s, CH_2), 30.0 (d, J_p = 2.0 Hz, CH_2), 31.0 (d, J_P = 40.4 Hz, Cq), 37.7 (s, CH), 43.8 (s, CH_2), 68.6 (d, J = 6.9 Hz, CH), 77.6 (s, CH), 171.8 (s, Cq) ppm. **^{31}P-NMR (162 MHz, $CDCl_3$) δ:** 69.4 – 71.2 (m) ppm. **HRMS (ESI)**: calc for $[C_{16}H_{34}BN_2OP+H]^+$: 313.2574, found 313.2578.

Synthesis of (R_P)-9c

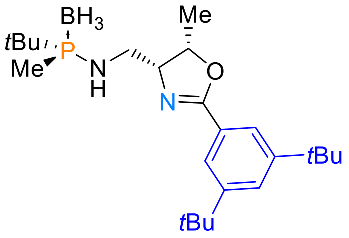

(R_P)-**9c**

Initially, the corresponding acid chloride was prepared by adding oxalyl chloride (0.58 mL, 6.75 mmol) and one drop of DMF to a solution of 3,5-di-*tert*-butyl benzoic acid (318.7 mg, 1.36 mmol) in DCM (4.6 mL) at 0 °C. The solution was stirred at room temperature for 3 h and then evaporated and dried under vacuum for 2 h. Afterwards, following GP4, using (R_P)-**7** (300.0 mg, 1.36 mmol), DCM (10.5 mL), Et_3N (0.38 mL, 2.73 mmol) and 3,5-di-*tert*-butyl benzoyl chloride (342.9 mg, 1.36 mmol) afforded intermediate (R_P)-**8c** as a white solid (472.4 mg, 79% yield). **^{1}H-NMR (400 MHz, $CDCl_3$) δ**: 0.09 – 0.79 (m, 3H), 1.10 (d, J = 14.0 Hz, 9H), 1.26 (d, J = 6.5 Hz, 3H), 1.35 (s, 18H), 1.35 (d, J = 9.2 Hz, 3H), 1.99 (br s, 1H), 2.79 (d, J = 4.7 Hz, 1H), 3.15 – 3.40 (m, 2H), 3.84 – 3.93

(m, 1H), 4.21 – 4.32 (m, 1H), 6.61 (d, *J* = 8.5 Hz, 1H), 7.57 – 7.59 (m, 1H), 7.60 (d, *J* = 1.8 Hz, 2H) ppm.

Consecutively, following GP4, (R_P)-**8c** (467.0 mg, 1.07 mmol), K_2CO_3 (592.0 mg, 4.28 mmol), DCM (13.2 mL) and DAST (364.0 mg, 2.14 mmol) were used. Purification by silica column chromatography (hexanes:EtOAc 85:15) afforded (R_P)-**9c** as a white solid (350.0 mg, 78% yield). **M.p.**: 163 – 167 °C. [**α**]$_D$**:** + 73.8 (c 0.95, $CHCl_3$). **IR (ATR-FTIR) υ$_{max}$**: 3343, 2962, 2905, 2868, 2382, 2348, 1642, 1593 cm^{-1}. **^{1}H-NMR (400 MHz, $CDCl_3$) δ:** 0.09 – 0.81 (m, BH_3), 1.13 (d, J_P = 13.9 Hz, 9H), 1.35 (s, 18H), 1.38 (d, *J* = 6.4 Hz, 3H), 1.39 (d, J_P = 6.8 Hz, 3H), 2.22 (d, *J* = 9.2 Hz, 1H), 2.94 – 3.03 (m, 1H), 3.30 – 3.41 (m, 1H), 4.21 (td, *J* = 9.3, 4.2 Hz, 1H), 4.88 (dq, *J* = 9.5, 6.6 Hz, 1H), 7.56 (t, *J* = 1.9 Hz, 1H), 7.78 (d, *J* = 1.9 Hz, 2H) ppm. **^{13}C-NMR (101 MHz, $CDCl_3$) δ:** 10.3 (d, J_P = 40.4 Hz, CH_3), 14.9 (s, CH_3), 24.8 (d, J_P = 3.0 Hz, 3xCH_3), 31.5 (d, J_P = 38.3 Hz, Cq), 31.5 (s, 6xCH_3), 35.1 (s, 2xCq), 44.5 (s, CH_2), 70.0 (d, J_P = 5.9 Hz, CH), 78.1 (s, CH), 122.6 (s, 2xCH), 125.9 (s, CH), 127.2 (s, Cq), 151.1 (s, 2xCq), 165.1 (s, Cq) ppm. **^{31}P-NMR (162 MHz, $CDCl_3$) δ:** 69.4 – 71.4 (m) ppm. **HRMS (ESI)**: calc for $[C_{24}H_{44}BN_2OP+H]^+$: 419.3357, found 419.3368.

Synthesis of (S_P)-9c

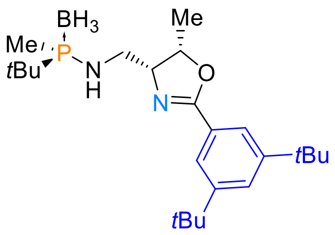

(S_P)-**9c**

Initially, the corresponding acid chloride was prepared by adding oxalyl chloride (0.22 mL, 2.60 mmol) and one drop of DMF to a solution of 3,5-di-*tert*-butyl benzoic acid (121.8 mg, 0.52 mmol) in DCM (1.7 mL) at 0 °C. The solution was stirred at room temperature for 3 h and then evaporated and dried under vacuum for 2 h. Afterwards, following GP4, using (S_P)-**7** (115.0 mg, 0.52 mmol), DCM (4 mL), Et_3N (0.13 mL, 0.91 mmol) and 3,5-di-*tert*-butyl benzoyl chloride (131.0 mg, 0.52 mmol) afforded intermediate (S_P)-**8c** as a colourless oil (171.0 mg, 72% yield). **^{1}H-NMR (400 MHz, $CDCl_3$) δ:** 0.12 – 0.81 (m, 3H), 1.13 (d, *J* = 14.1 Hz, 9H), 1.23 (d, *J* = 9.0 Hz, 3H), 1.26 (d, *J* = 6.4 Hz, 3H), 1.35 (s, 18H), 1.95 (br s, 1H), 2.52 (br s, 1H), 3.22 – 3.41 (m, 2H), 3.94 – 4.05 (m, 1H), 4.10 – 4.20 (m, 1H), 6.68 (d, *J* = 8.5 Hz, 1H), 7.59 (t, *J* = 1.8 Hz, 1H), 7.66 (d, *J* = 1.8 Hz, 2H) ppm.

Consecutively, following GP4, (S_P)-**8c** (119.3 mg, 0.27 mmol), K_2CO_3 (156.2 mg, 1.13 mmol), DCM (3.5 mL) and DAST (95.9 mg, 0.57 mmol) were used. Purification by silica column chromatography (hexanes:EtOAc 9:1) afforded (S_P)-**9c** as a colourless oil (87.5 mg, 77% yield). **[α]$_D$:** + 72.8 (c 1.25, $CHCl_3$). **IR (ATR-FTIR) υ$_{max}$:** 3352, 2962, 2869, 2378, 2263, 1643, 1592 cm^{-1}. **^{1}H-NMR (400 MHz, $CDCl_3$) δ:** 0.08 – 0.91 (m, BH_3), 1.17 (d, J_P = 14.0 Hz, 9H), 1.32 (d, J_P = 9.1 Hz, 3H), 1.35 (s, 18H), 1.41 (d, J = 6.7 Hz, 3H), 2.10 (br s, 1H), 3.05 – 3.14 (m, 1H), 3.22 – 3.32 (m, 1H), 4.22 (td, J = 8.9, 4.3 Hz, 1H), 4.89 (dq, J = 9.6, 6.7 Hz, 1H), 7.56 (t, J = 1.6 Hz, 1H), 7.78 (d, J = 1.2 Hz, 2H) ppm. **^{13}C-NMR (101 MHz, $CDCl_3$) δ:** 9.8 (d, J_P = 37.8 Hz, CH_3), 15.1 (s, CH_3), 24.8 (d, J_P = 2.9 Hz, 3xCH_3), 31.1 (d, J_P = 40.3 Hz, Cq), 31.5 (s, 6xCH_3), 35.1 (s, 2xCq), 43.9 (s, CH_2), 69.4 (d, J_P = 6.9 Hz, CH), 78.1 (s, CH), 122.6 (s, 2xCH), 126.0 (s, CH), 127.2 (s, Cq), 151.1 (s, Cq), 165.0 (s, Cq) ppm. **^{31}P-NMR (162 MHz, $CDCl_3$) δ:** 68.9 – 71.9 (m) ppm. **HRMS (ESI):** calc for $[C_{24}H_{44}BN_2OP+H]^+$: 419.3357, found 419.3366.

Synthesis of (R_P)-9d

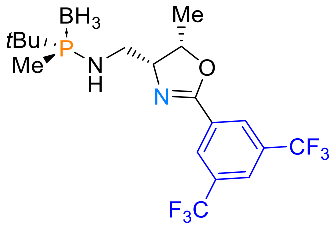

(R_P)-**9d**

Following GP4, using (R_P)-**7** (120.0 mg, 0.54 mmol), DCM (4.1 mL), Et_3N (0.15 mL, 1.09 mmol) and and 3,5-bis(trifluoromethyl)benzoyl chloride (102 µL, 0.54 mmol) afforded intermediate (R_P)-**8d** as a colourless oil (175.1 mg, 70% yield). **^{1}H-NMR (400 MHz, $CDCl_3$) δ**: 0.13 – 0.84 (m, 3H), 1.11 (d, J = 14.1 Hz, 9H), 1.27 (d, J = 6.5 Hz, 3H), 1.36 (d, J = 9.2 Hz, 3H), 1.94 (br s, 1H), 3.15 – 3.25 (m, 1H), 3.25 – 3.38 (m, 1H), 3.83 – 3.99 (m, 1H), 4.26 – 4.39 (m, 1H), 6.78 (d, J = 8.5 Hz, 1H), 8.02 (s, 1H), 8.24 (s, 2H) ppm.

Consecutively, following GP4, (R_P)-**8d** (172.6 mg, 0.38 mmol), K_2CO_3 (207.3 mg, 1.50 mmol), DCM (4.7 mL) and DAST (128.0 mg, 0.75 mmol) were used. Purification by silica column chromatography (hexanes:EtOAc 9:1) afforded (R_P)-**9d** as a colourless oil (75.5 mg, 46% yield). **[α]$_D$:** + 86.4 (c 1.34, $CHCl_3$). **IR (ATR-FTIR) υ$_{max}$:** 3336, 2971, 2945, 2904, 2870, 2374, 1651 cm^{-1}. **^{1}H-NMR (400 MHz, $CDCl_3$) δ:** 0.07 – 0.92 (m, BH_3), 1.14 (d, J_P = 14.0 Hz, 9H), 1.38 (d, J = 3.6 Hz, 3H), 1.40 (d, J_P = 5.9 Hz, 3H), 2.16 (d, J = 9.9 Hz, 1H), 2.89 – 3.06 (m, 1H), 3.35 – 3.51 (m, 1H), 4.28 (td, J = 9.7, 4.0 Hz, 1H), 4.99 (dq, J = 9.6, 6.7 Hz, 1H), 7.98 (s, 1H), 8.39 (s, 2H) ppm.

^{13}**C-NMR (101 MHz, CDCl$_3$) δ:** 10.5 (d, J_P = 40.4 Hz, CH$_3$), 15.0 (s, CH$_3$), 24.8 (d, J_P = 3.0 Hz, 3xCH$_3$), 31.5 (d, J_P = 38.6 Hz, Cq), 44.3 (d, J_P = 2.1 Hz, CH$_2$), 70.7 (d, J_P = 5.1 Hz, CH), 79.2 (s, CH), 123.1 (q, J_F = 272.9 Hz, 2xCF$_3$), 124.7 – 125.1 (m, CH), 128.5 (d, J_F = 3.8 Hz, 2xCH), 130.2 (s, Cq), 132.1 (q, J_F = 34.0 Hz, 2xCq), 161.6 (s, Cq) ppm. ^{31}**P-NMR (162 MHz, CDCl$_3$) δ:** 70.3 – 72.6 (m) ppm. **HRMS (ESI)**: calc for [$C_{18}H_{26}BF_6N_2OP$+H]$^+$: 443.1852, found 443.1860.

Synthesis of (*S*$_P$)-9d

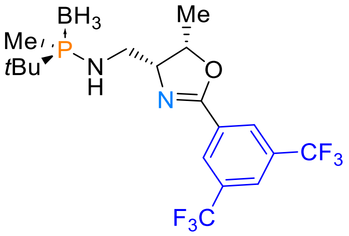

(*S*$_P$)-**9d**

Following GP4, using (*S*$_P$)-**7** (125.0 mg, 0.57 mmol), DCM (4.3 mL), Et$_3$N (0.16 mL, 1.14 mmol) and and 3,5-bis(trifluoromethyl)benzoyl chloride (106 µL, 0.57 mmol) afforded intermediate (*S*$_P$)-**8d** as a colourless oil (250.7 mg, 96% yield). ^{1}**H-NMR (400 MHz, CDCl$_3$) δ**: 0.10 – 0.83 (m, 3H), 1.15 (d, *J* = 14.1 Hz, 9H), 1.23 (d, *J* = 9.0 Hz, 3H), 1.27 (d, *J* = 6.5 Hz, 3H), 3.25 – 3.33 (m, 2H), 3.93 – 4.09 (m, 1H), 4.18 – 4.26 (m, 1H), 6.85 (d, *J* = 8.5 Hz, 1H), 8.02 (s, 1H), 8.29 (s, 2H) ppm. Consecutively, following GP4, (*S*$_P$)-**8d** (248.9 mg, 0.54 mmol), K$_2$CO$_3$ (299.0 mg, 2.16 mmol), DCM (6.8 mL) and DAST (174.0 mg, 1.08 mmol) were used. Purification by silica column chromatography (hexanes:EtOAc 9:1) afforded (*S*$_P$)-**9d** as a colourless oil (121.6 mg, 51% yield). **[α]$_D$:** + 101.4 (c 0.66, CHCl$_3$). **IR (ATR-FTIR) υ_{max}:** 3336, 2971, 2945, 2904, 2870, 2385, 1650 cm^{-1}. ^{1}**H-NMR (400 MHz, CDCl$_3$) δ:** 0.07 – 0.92 (m, BH$_3$), 1.18 (d, J_P = 14.1 Hz, 9H), 1.35 (d, J_P = 9.0 Hz, 3H), 1.41 (d, *J* = 9.0 Hz, 3H), 2.04 (d, *J* = 9.2 Hz, 1H), 2.98 – 3.13 (m, 1H), 3.32 – 3.44 (m, 1H), 4.29 (td, *J* = 9.5, 4.3 Hz, 1H), 4.99 (dq, *J* = 9.7, 6.7 Hz, 1H), 7.99 (s, 1H), 8.39 (s, 2H) ppm. ^{13}**C-NMR (101 MHz, CDCl$_3$) δ:** 10.1 (d, J_P = 37.4 Hz, CH$_3$), 15.1 (s, CH$_3$), 24.8 (d, J_P = 2.9 Hz, 3xCH$_3$), 30.9 (d, J_P = 40.9 Hz, Cq), 43.7 (s, CH$_2$), 70.1 (d, J_P = 6.3 Hz, CH), 79.2 (s, CH), 123.1 (q, J_F = 273.6 Hz, 2xCF$_3$), 124.7 – 125.1 (m, CH), 128.6 (d, J_F = 3.0 Hz, 2xCH), 130.2 (s, Cq), 132.1 (q, J_F = 33.9 Hz, 2xCq), 161.5 (s, Cq) ppm. ^{31}**P-NMR (162 MHz, CDCl$_3$) δ:** 70.4 – 72.2 (m) ppm. **HRMS (ESI)**: calc for [$C_{18}H_{26}BF_6N_2OP$+H]$^+$: 443.1852, found 443.1864.

Synthesis of (R_P)-9e

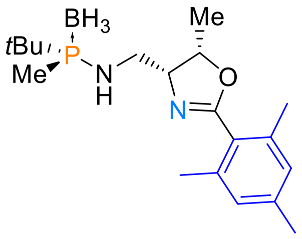

(R_P)-**9e**

Following GP4, using (R_P)-**7** (125.0 mg, 0.52 mmol), DCM (4.3 mL), Et_3N (0.15 mL, 1.05 mmol) and and 2,4,6-trimethylbenzoyl chloride (89 μL, 0.53 mmol) afforded intermediate (R_P)-**8e** as a colourless oil (162.3 mg, 85% yield). **^{1}H-NMR (400 MHz, $CDCl_3$) δ**: 0.10 – 0.87 (m, 3H), 1.12 (d, J = 14.0 Hz, 9H), 1.27 (d, J = 6.5 Hz, 3H), 1.35 (d, J = 9.0 Hz, 3H), 1.98 (s, 1H), 2.27 (s, 3H), 2.29 (s, 6H), 3.14 – 3.34 (m, 2H), 3.75 – 3.90 (m, 1H), 4.29 (q, J = 6.6 Hz, 1H), 6.13 (d, J = 8.6 Hz, 1H), 6.83 (s, 2H) ppm.

Consecutively, following GP4, (R_P)-**8e** (162.3 mg, 0.44 mmol), K_2CO_3 (245.0 mg, 1.77 mmol), DCM (5.4 mL) and DAST (150.4 mg, 0.89 mmol) were used. Purification by silica column chromatography (hexanes:EtOAc 9:1) afforded (R_P)-**9e** as a colourless oil (128.4 mg, 58% yield). **$[\alpha]_D$:** + 69.7 (c 0.65, $CHCl_3$). **IR (ATR-FTIR) υ_{max}:** 3350, 2927, 2867, 2382, 2348, 1660 cm^{-1}. **^{1}H-NMR (400 MHz, $CDCl_3$) δ:** 0.08 – 0.91 (m, BH_3), 1.12 (d, J_P = 13.9 Hz, 9H), 1.34 (d, J_P = 9.2 Hz, 3H), 1.37 (d, J = 6.7 Hz, 3H), 2.27 (s, 3H), 2.29 (s, 6H), 2.95 – 3.08 (m, 1H), 3.40 – 3.53 (m, 1H), 4.27 (td, J = 9.9, 3.8 Hz, 1H), 4.90 (dq, J = 9.7, 6.7 Hz, 1H), 6.86 (s, 2H) ppm. **^{13}C-NMR (101 MHz, $CDCl_3$) δ:** 10.6 (d, J_P = 40.5 Hz, CH_3), 14.9 (s, CH_3), 19.6 (s, 2xCH_3), 21.3 (s, CH_3), 24.8 (s, 3xCH_3), 31.5 (d, J_P = 38.6 Hz, Cq), 44.8 (d, J_P = 2.2 Hz, CH_2), 70.4 (d, J_P = 5.0 Hz, CH), 77.8 (s, CH), 125.9 (s, Cq), 128.4 (s, 2xCH), 136.7 (s, Cq), 139.6 (s, 2xCq), 165.2 (s, Cq) ppm. **^{31}P-NMR (162 MHz, $CDCl_3$) δ:** 70.1 – 71.9 (m) ppm. **HRMS (ESI)**: calc for $[C_{19}H_{34}BN_2OP+H]^+$: 349.2574, found 349.2583.

Synthesis of (S_P)-9e

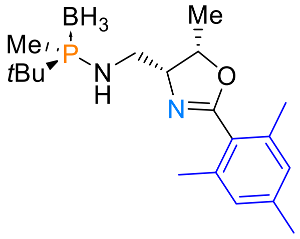

(S_P)-**9e**

Following GP4, using (S_P)-**7** (125.0 mg, 0.57 mmol), DCM (4.3 mL), Et_3N (0.16 mL, 1.14 mmol) and 2,4,6-trimethylbenzoyl chloride (96 µL, 0.57 mmol) afforded intermediate (S_P)-**8e** as a colourless oil (193.2 mg, 93% yield). **^{1}H-NMR (400 MHz, $CDCl_3$) δ**: 0.03 – 0.85 (m, 3H), 1.16 (d, J = 14.1 Hz, 9H), 1.27 (d, J = 6.4 Hz, 3H), 1.29 (d, J = 8.8 Hz, 3H), 2.27 (s, 3H), 2.30 (s, 6H), 3.17 – 3.35 (m, 2H), 3.85 – 3.96 (m, 1H), 4.16 – 4.32 (m, 1H), 6.17 (d, J = 8.4 Hz, 1H), 6.84 (s, 2H) ppm. Consecutively, following GP4, (S_P)-**8e** (192.0 mg, 0.52 mmol), K_2CO_3 (290.0 mg, 2.10 mmol), DCM (6.3 mL) and DAST (178.0 mg, 1.05 mmol) were used. Purification by silica column chromatography (hexanes:EtOAc 8:2) afforded (S_P)-**9e** as a colourless oil (114.0 mg, 62% yield). **$[\alpha]_D$:** + 149.3 (c 0.73, $CHCl_3$). **IR (ATR-FTIR) υ_{max}**: 3373, 2972, 2928, 2868, 2363, 2339, 1658 cm^{-1}. **^{1}H-NMR (400 MHz, $CDCl_3$) δ:** 0.08 – 0.92 (m, BH_3), 1.14 (d, J_P = 14.0 Hz, 9H), 1.30 (d, J_P = 9.0 Hz, 3H), 1.40 (d, J = 6.6 Hz, 3H), 2.15 (d, J = 12.4 Hz, 1H), 2.27 (s, 3H), 2.30 (s, 6H), 3.02 – 3.16 (m, 1H), 3.23 – 3.40 (m, 1H), 4.28 (td, J = 9.5, 4.0 Hz, 1H), 4.89 (dq, J = 9.6, 6.6 Hz, 1H), 6.86 (s, 2H) ppm. **^{13}C-NMR (101 MHz, $CDCl_3$) δ:** 10.0 (d, J_P = 37.6 Hz, CH_3), 15.0 (s, CH_3), 19.7 (s, 2xCH_3), 21.3 (s, CH_3), 24.7 (d, J = 3.0 Hz, 3xCH_3), 30.9 (d, J = 40.9 Hz, Cq), 44.1 (s, CH_2), 69.7 (d, J = 6.9 Hz, CH), 77.8 (s, CH), 126.0 (s, Cq), 128.5 (s, 2xCH), 136.7 (s, 2xCq), 139.5 (s, Cq), 165.0 (s, Cq) ppm. **^{31}P-NMR (162 MHz, $CDCl_3$) δ:** 69.9 – 71.9 (m) ppm. **HRMS (ESI)**: calc for $[C_{19}H_{34}BN_2OP+H]^+$: 349.2574, found 349.2580.

Synthesis of (R_P)-9f

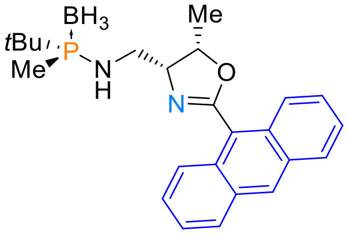

(R_P)-**9f**

Initially, the corresponding acid chloride was prepared by adding oxalyl chloride (0.14 mL, 1.65 mmol) and one drop of DMF to a solution of anthracene-9-carboxylic acid (122.2 mg, 0.55 mmol) in DCM (5.5 mL) at 0 °C. The solution was stirred at room temperature for 4 h and then evaporated and dried under vacuum for 2 h. Afterwards, following GP4, using (R_P)-**7** (125.0 mg, 0.55 mmol), DCM (4.3 mL), Et_3N (0.15 mL, 1.09 mmol) and 9-anthracenecarboxyl chloride (132.4 mg, 0.55 mmol) afforded intermediate (R_P)-**8f** as a yellow solid (129.2 mg, 56% yield). **^{1}H-NMR (400 MHz, $CDCl_3$) δ**: 0.10 – 0.87 (m, 3H), 1.14 (d, J = 14.0 Hz, 9H), 1.35 (d, J = 9.2 Hz, 3H), 1.39 (d, J = 6.5

Hz, 3H), 2.23 (br s, 1H), 2.67 (d, *J* = 4.8 Hz, 1H), 3.37 – 3.49 (m, 2H), 4.12 – 4.21 (m, 1H), 4.30 – 4.38 (m, 1H), 6.54 (d, *J* = 8.6 Hz, 1H), 7.36 – 7.58 (m, 4H), 7.90 – 8.12 (m, 4H), 8.48 (s, 1H) ppm. Consecutively, following GP4, (R_P)-**8f** (129.2 mg, 0.30 mmol), K_2CO_3 (168.0 mg, 1.22 mmol), DCM (3.7 mL) and DAST (104.0 mg, 0.61 mmol) were used. Purification by silica column chromatography (hexanes:EtOAc 8:2) afforded (R_P)-**9f** as a yellow solid (68.7 mg, 56% yield). **M.p.**: 118 – 120 °C. **[α]$_D$:** + 62.6 (c 1.26, $CHCl_3$). **IR (ATR-FTIR)** υ_{max}: 3245, 2965, 2926, 2865, 2370, 2258, 1651 cm^{-1}. **^{1}H-NMR (400 MHz, $CDCl_3$) δ:** 0.14 – 0.95 (m, BH_3), 1.15 (d, J_P = 14.0 Hz, 9H), 1.36 (d, J_P = 9.2 Hz, 3H), 1.58 (d, *J* = 6.7 Hz, 3H), 2.38 (d, *J* = 9.6 Hz, 1H), 3.24 – 3.40 (m, 1H), 3.58 – 3.71 (m, 1H), 4.60 (td, *J* = 9.7, 3.8 Hz, 1H), 5.19 (dq, *J* = 9.8, 6.7 Hz, 1H), 7.45 – 7.59 (m, 4H), 8.02 (d, *J* = 7.7 Hz, 2H), 8.14 (d, *J* = 8.8 Hz, 2H), 8.55 (s, 1H) ppm. **^{13}C-NMR (101 MHz, $CDCl_3$) δ:** 10.5 (d, J_P = 40.1 Hz, CH_3), 15.3 (s, CH_3), 24.8 (d, J_P = 3.1 Hz, 3xCH_3), 31.5 (d, J_P = 38.7 Hz, Cq), 44.9 (d, J_P = 2.3 Hz, CH_2), 71.0 (d, J_P = 5.3 Hz, CH), 78.6 (s, CH), 122.7 (s, Cq), 125.1 (s, CH), 125.6 (s, CH), 127.1 (s, CH), 128.8 (s, CH), 129.8 (s, CH), 130.1 (s, Cq), 131.2 (s, Cq), 164.0 (s, Cq) ppm. **^{31}P-NMR (162 MHz, $CDCl_3$) δ:** 70.3 – 72.5 (m) ppm. **HRMS (ESI)**: calc for $[C_{24}H_{32}BN_2OP+H]^+$: 407.2418, found 407.2433.

Synthesis of (S_P)-9f

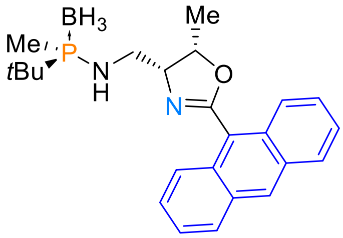

(S_P)-**9f**

Initially, the corresponding acid chloride was prepared by adding oxalyl chloride (0.15 mL, 1.71 mmol) and one drop of DMF to a solution of anthracene-9-carboxylic acid (133.3 mg, 0.57 mmol) in DCM (5.7 mL) at 0 °C. The solution was stirred at room temperature for 4 h and then evaporated and dried under vacuum for 2 h. Afterwards, following GP4, using (S_P)-**7** (125.0 mg, 0.57 mmol), DCM (4.3 mL), Et_3N (0.16 mL, 1.14 mmol) and 9-anthracenecarboxyl chloride (137.2 mg, 0.57 mmol) afforded intermediate (S_P)-**8f** as a yellow solid (117.5 mg, 49% yield). **^{1}H-NMR (400 MHz, $CDCl_3$) δ**: 0.10 – 0.83 (m, 3H), 1.19 (d, *J* = 14.1 Hz, 9H), 1.36 (d, *J* = 9.0 Hz, 3H), 1.41 (d, *J* = 6.4 Hz, 3H), 2.24 (br s, 1H), 2.39 (d, *J* = 4.6 Hz, 1H), 3.33 – 3.57 (m, 2H), 4.19 – 4.40 (m, 2H), 6.58 (d,

J = 8.3 Hz, 1H), 7.42 – 7.61 (m, 4H), 8.02 (d, J = 8.4 Hz, 2H), 8.08 (d, J = 8.6 Hz, 2H), 8.50 (s, 1H) ppm.

Consecutively, following GP4, (S_P)-**8f** (117.5 mg, 0.28 mmol), K_2CO_3 (153.0 mg, 1.11 mmol), DCM (3.4 mL) and DAST (94.0 mg, 0.55 mmol) were used. Purification by silica column chromatography (hexanes:EtOAc 8:2) afforded (S_P)-**9f** as a yellow solid (56.3 mg, 50% yield). **M.p.**: 129 – 131 °C. **$[\alpha]_D$:** + 65.9 (c 1.10, $CHCl_3$). **IR (ATR-FTIR) υ_{max}:** 3327, 2969, 2945, 2903, 2870, 2385, 1638 cm^{-1}. **^{1}H-NMR (400 MHz, $CDCl_3$) δ:** 0.10 – 0.91 (m, BH_3), 1.16 (d, J_P = 14.0 Hz, 9H), 1.36 (d, J_P = 9.1 Hz, 3H), 1.61 (d, J = 6.7 Hz, 3H), 2.26 (br s, 1H), 3.34 – 3.47 (m, 1H), 3.46 – 3.58 (m, 1H), 4.60 (td, J = 9.2, 4.1 Hz, 1H), 5.19 (dq, J = 9.8, 6.7 Hz, 1H), 7.45 – 7.59 (m, 4H), 8.02 (d, J = 9.1 Hz, 2H), 8.15 (d, J = 7.6 Hz, 2H), 8.55 (s, 1H) ppm. **^{13}C-NMR (101 MHz, $CDCl_3$) δ:** 10.2 (d, J_P = 38.0 Hz, CH_3), 15.3 (s, CH_3), 24.8 (d, J_P = 3.0 Hz, 3xCH_3), 31.0 (d, J_P = 40.3 Hz, Cq), 44.3 (d, J_P = 1.9 Hz, CH_2), 70.4 (d, J_P = 7.0 Hz, CH), 78.7 (s, CH), 122.7 (s, Cq), 125.1 (s, CH), 125.6 (s, CH), 127.0 (s, CH), 128.8 (s, CH), 129.8 (s, CH), 130.1 (s, Cq), 131.2 (s, Cq), 164.0 (s, Cq) ppm. **^{31}P-NMR (162 MHz, $CDCl_3$) δ:** 70.5 – 72.3 (m) ppm. **HRMS (ESI)**: calc for $[C_{24}H_{32}BN_2OP+H]^+$: 407.2418, found 407.2417.

2.2.3 Preparation of catalysts 10-15

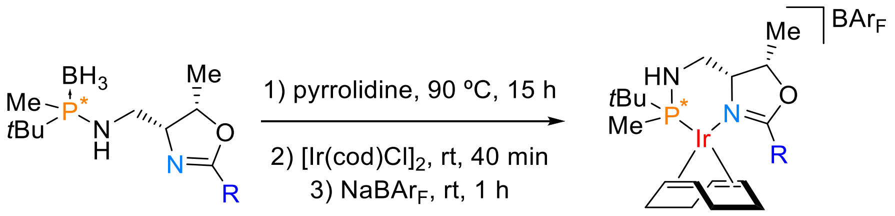

General procedure for the preparation of catalysts 10-15 (GP5):

The corresponding borane-protected ligand (1 eq.) was added to a flame-dried Schlenk round-bottom flask, purged with vacuum-nitrogen cycles and dissolved in freshly distilled and degassed pyrrolidine (0.06M). The mixture was stirred for 15 h at 90 °C. Then, under strict inert atmosphere, pyrrolidine was removed under vacuum and further dried at 50 °C for 30 min. A solution of $[Ir(cod)Cl]_2$ (0.05 eq.) in DCM was added to the free ligand and stirred at room temperature for 40 min. $NaBAr_F$ (1 eq.) was then added and the solution was stirred for 1 h at room temperature. The resulting crude was purified by filtering through a plug of silica gel under nitrogen, eluting with hexanes:DCM 1:1 (the plug was initially washed with anhydrous Et_2O). The coloured fraction was collected and concentrated affording the desired product.

Synthesis of (R_P)-10

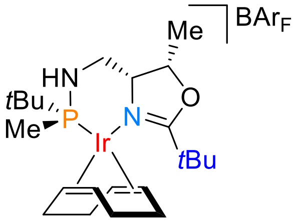

(R_P)-**10**

Following GP5, using (S_P)-**9a** (50.0 mg, 0.17 mmol), pyrrolidine (3.2 mL), $[Ir(cod)Cl]_2$ (58.6 mg, 0.09 mmol), DCM (2.1 mL) and $NaBAr_F$ (154.8 mg, 0.17 mmol) afforded (R_P)-**10** as an orange-brownish solid (106.4 mg, 42% yield). **[α]D:** + 13.4 (c 1.17, $CHCl_3$). **IR (ATR-FTIR) υ_{max}:** 2958, 2925, 2854, 1725, 1599, 1465, 1355, 1277, 1131 cm^{-1}. **^{1}H-NMR (400 MHz, $CDCl_3$) δ:** 1.07 (d, J_P = 15.0 Hz, 9H), 1.30 (d, J = 6.7 Hz, 3H), 1.34 (d, J_P = 8.0 Hz, 3H), 1.48 (s, 9H), 1.95 – 2.13 (m, 5H), 2.23 – 2.33 (m, 1H), 2.37 – 2.44 (m, 2H), 2.99 – 3.12 (m, 1H), 3.30 – 3.44 (m, 1H), 3.50 – 3.57 (m, 1H), 3.84 (qu, J = 7.2 Hz, 1H), 4.28 (td, J = 8.5, 3.6 Hz, 1H), 4.32 – 4.40 (m, 1H), 4.69 – 4.78 (m, 1H), 4.79 – 4.86 (m, 1H), 7.53 (s, 4H), 7.70 (s, 8H) ppm. **^{13}C-NMR (101 MHz, $CDCl_3$) δ:** 11.1 (d, J_P = 35.9 Hz, CH_3), 13.5 (s, CH_3), 25.0 (d, J_P = 2.6 Hz, CH_2), 26.5 (d, J_P = 5.7 Hz, 3xCH_3), 28.7 (s, CH_2), 28.9 (s, 3xCH_3), 33.6 (s, CH_2), 33.9 (s, Cq), 37.7 (d, J_P = 2.8 Hz, Cq), 38.0 (d, J_P = 27.9 Hz, CH_2), 44.0 (s, CH_2), 64.4 (s, CH), 65.9 (s, CH), 70.1 (s, CH), 79.5 (s, CH), 85.6 (d, J_P = 15.9 Hz, CH), 94.1 (d, J_P = 9.8 Hz, CH), 117.6 (s, 4xCH), 124.7 (q, J_F = 272.8 Hz, 8xCF_3), 128.4 – 129.6 (m, 8xCq), 134.9 (s, 8xCH), 161.8 (q, J_B = 49.7 Hz, 4xCq), 180.12 (s, Cq) ppm. **^{31}P-NMR (162 MHz, $CDCl_3$) δ:** 58.7 (s) ppm. **HRMS (ESI, infusion)**: calc for $[C_{22}H_{41}IrN_2OP]^+$: 573.2580, found 573.2583. Calc for $[C_{32}H_{12}BF_{24}]^-$: 863.0654, found 863.0663.

Synthesis of (S_P)-10

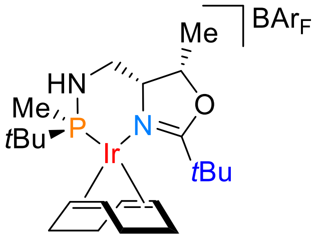

(S_P)-**10**

Following GP5, using (R_P)-**9a** (52.7 mg, 0.18 mmol), pyrrolidine (3.2 mL), $[Ir(cod)Cl]_2$ (61.8 mg, 0.09 mmol), DCM (2.2 mL) and $NaBAr_F$ (168.2 mg, 0.18 mmol) afforded (S_P)-**10** as an orange solid (133.2 mg, 51% yield). **[α]D:** + 43.9 (c 1.06, $CHCl_3$). **IR (ATR-FTIR) υ_{max}:** 2957, 2924, 2873, 2854,

1720, 1602, 1463, 1352, 1272, 1119 cm^{-1}. **^{1}H-NMR (400 MHz, $CDCl_3$) δ:** 1.06 (d, J_P = 14.8 Hz, 9H), 1.26 (d, J = 6.6 Hz, 3H), 1.39 (d, J_P = 6.3 Hz, 3H), 1.54 (s, 9H), 1.90 – 2.40 (m, 8H), 3.17 – 3.30 (m, 1H), 3.76 – 3.90 (m, 2H), 4.01 – 4.12 (m, 2H), 4.75 (br s, 1H), 4.78 – 4.87 (m, 1H), 4.97 (br s, 1H), 7.53 (s, 4H), 7.70 (s, 8H) ppm. **^{13}C-NMR (101 MHz, $CDCl_3$) δ:** 12.9 (d, J_P = 28.9 Hz, CH_3), 15.6 (s, CH_3), 25.0 (d, J_P = 3.9 Hz), 25.1 (d, J = 2.8 Hz), 29.0 (s, CH_2), 29.1 (s, 3xCH_3), 33.9 (s, CH_2), 34.1 (s, CH_2), 35.4 (d, J_P = 30.7 Hz, 3xCH_3), 37.2 (d, J_P = 3.7 Hz, CH_2), 43.6 (d, J_P = 4.2 Hz, CH_2), 60.1 (s, CH), 66.4 (s, CH), 69.4 (s, CH), 79.2 (s, CH), 83.3 (d, J_P = 16.9 Hz, CH), 91.9 (d, J = 8.9 Hz, CH), 117.6 (s, 4xCH), 124.7 (q, J = 272.8 Hz, 8xCF_3), 129.0 (q, J = 30 Hz, Cq), 131.1 (s, Cq), 134.9 (s, 8xCH), 161.8 (q, J_B = 49.9 Hz, 4xCq), 179.2 (s, Cq) **^{31}P-NMR (162 MHz, $CDCl_3$) δ:** 60.1 (s) ppm. **HRMS (ESI, infusion)**: calc for $[C_{22}H_{41}IrN_2OP]^+$: 573.2580, found 573.2607. Calc for $[C_{32}H_{12}BF_{24}]^-$: 863.0654, found 863.0667.

Synthesis of (*R*$_P$)-11

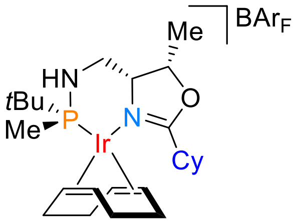

(*R*$_P$)-**11**

Following GP5, using (*S*$_P$)-**9b** (89.1 mg, 0.29 mmol), pyrrolidine (4.8 mL), $[Ir(cod)Cl]_2$ (95.8 mg, 0.14 mmol), DCM (4.8 mL) and NaBAr$_F$ (261.0 mg, 0.29 mmol) afforded (*R*$_P$)-**11** as a red solid (194.6 mg, 47% yield). [α]$_D$: - 24.3 (c 0.96, $CHCl_3$). **IR (ATR-FTIR) υ_{max}**: 2937, 2929, 2853, 1625, 1458, 1354m 1270, 1118 cm^{-1}. **^{1}H-NMR (400 MHz, $CDCl_3$) δ:** 1.13 (d, J_P = 15.0 Hz, 9H), 1.20 (d, J_P = 8.0 Hz, 3H), 1.30 (d, J = 6.7 Hz, 3H), 1.44 – 1.88 (m, 11H), 1.97 – 2.14 (m, 3H), 2.21 – 2.40 (m, 2H), 2.41 – 2.57 (m, 3H), 3.11 (tdd, J = 14.2, 6.1, 4.4 Hz, 1H), 3.34 – 3.51 (m, 1H), 3.58 – 3.70 (m, 1H), 3.71 – 3.80 (m, 1H), 4.18 (m, 1H), 4.51 – 4.64 (m, 1H), 4.64 – 4.76 (m, 1H), 4.88 (dq, J = 9.4, 6.7 Hz, 1H), 7.53 (s, 4H), 7.70 (s, 8H) ppm. **^{13}C-NMR (101 MHz, $CDCl_3$) δ:** 7.6 (d, J_P = 31.6 Hz, CH_3), 14.4 (s, CH_3), 25.0 (s, CH_2), 25.2 (s, CH_2), 25.2 (s, CH_2), 26.8 (d, J_P = 4.8 Hz, 3xCH_3), 27.0 (s, CH_2), 28.0 (s, CH_2), 29.8 (s, CH_2), 31.4 (s, CH_2), 32.1 (s, CH_2), 36.0 (s, CH_2), 37.6 (d, J_P = 33.8 Hz, Cq), 40.5 (s, CH), 42.9 (s, CH_2), 60.5 (s, CH), 65.3 (s, CH), 67.4 (s, CH), 80.4 (s, CH), 91.8 (d, J_P = 12.4 Hz, CH), 95.0 (d, J_P = 12.9 Hz, CH), 117.6 (s, 4xCH), 124.7 (q, J_F = 272.5 Hz, 8xCF_3), 128.5 – 129.6 (m, 8xCq), 134.9 (s, 8xCH), 161.8 (q, J_B = 50.1 Hz, 4xCq), 177.0 (s, Cq) ppm. **^{31}P-**

NMR (162 MHz, CDCl$_3$) δ: 60.4 (s) ppm. **HRMS (ESI, infusion)**: calc for [$C_{27}H_{55}IrN_2OP$]$^+$: 599.2736, found 599.2752. Calc for [$C_{32}H_{12}BF_{24}$]$^-$: 863.0654, found 863.0679.

Synthesis of (S_P)-11

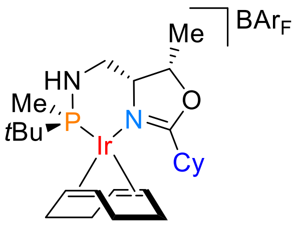

(S_P)-**11**

Following GP5, using (R_P)-**9b** (111.9 mg, 0.36 mmol), pyrrolidine (6 mL), [Ir(cod)Cl]$_2$ (120 mg, 0.18 mmol), DCM (6 mL) and NaBAr$_F$ (328.0 mg, 0.36 mmol) afforded (S_P)-**11** as a red solid (266.8 mg, 51% yield). [**α**]$_D$**:** + 39.9 (c 1.02, CHCl$_3$). **IR (ATR-FTIR) υ_{max}:** 2931, 2856, 1622, 1452, 1353, 1271, 1120 cm^{-1}. **^{1}H-NMR (400 MHz, CDCl$_3$) δ:** 1.04 (d, J_P = 14.7 Hz, 9H), 1.28 (d, J = 6.6 Hz, 3H), 1.40 (d, J_P = 6.6 Hz, 3H), 1.47 – 1.73 (m, 7H), 1.73 – 1.87 (m, 3H), 1.85 – 1.94 (m, 2H), 1.95 – 2.08 (m, 2H), 2.09 – 2.21 (m, 2H), 2.22 – 2.37 (m, 3H), 2.92 – 3.05 (m, 1H), 3.16 – 3.36 (m, 1H), 3.57 – 3.71 (m, 1H), 3.95 (dd, J = 10.1, 4.9 Hz, 1H), 4.05 – 4.18 (m, 2H), 4.55 (br s, 1H), 4.86 – 5.00 (m, 2H), 7.53 (s, 4H), 7.70 (s, 8H) ppm. **^{13}C-NMR (101 MHz, CDCl$_3$) δ:** 12.1 (d, J_P = 31.8 Hz, CH$_3$), 16.1 (s, CH$_3$), 25.1 (d, J_P = 3.9 Hz, 3xCH$_3$), 25.2 (s, CH$_2$), 25.4 (s, CH$_2$), 25.7 (s, CH$_2$), 26.7 (s, CH$_2$), 29.8 (s, CH$_2$), 30.3 (s, CH$_2$), 31.4 (s, CH$_2$), 32.2 (s, CH$_2$), 36.1 (d, J_P = 3.2 Hz, CH$_2$), 36.5 (d, J_P = 29.1 Hz, Cq), 41.2 (s, CH), 43.4 (d, J_P = 4.8 Hz, CH$_2$), 60.7 (s, CH), 65.5 (s, CH), 66.5 (s, CH), 80.6 (s, CH), 87.2 (d, J_P = 15.3 Hz, CH), 93.0 (d, J_P = 10.7 Hz, CH), 117.6 (s, 4xCH), 124.7 (q, J_F = 272.6 Hz, 8xCF$_3$), 128.5 – 129.6 (m, 8xCq), 135.0 (s, 8xCH), 161.9 (q, J_B = 50.0 Hz, 4xCq), 175.7 (s, Cq) ppm. **^{31}P-NMR (162 MHz, CDCl$_3$) δ:** 63.0 (s) ppm. **HRMS (ESI, infusion)**: calc for [$C_{27}H_{55}IrN_2OP$]$^+$: 599.2736, found 599.2735. Calc for [$C_{32}H_{12}BF_{24}$]$^-$: 863.0654, found 863.0679.

Synthesis of (R_P)-12

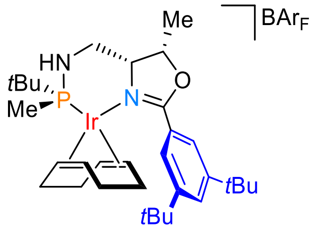

(R_P)-**12**

Following GP5, using (S_P)-**9c** (59.6 mg, 0.14 mmol), pyrrolidine (2.4 mL), $[Ir(cod)Cl]_2$ (47.4 mg, 0.07 mmol), DCM (2.4 mL) and $NaBAr_F$ (128.9 mg, 0.14 mmol) afforded (R_P)-**12** as an orange solid (125.6 mg, 57% yield). **[α]D:** + 28.6 (c 1.19, $CHCl_3$). **IR (ATR-FTIR) υ_{max}:** 2966, 2925, 2870, 2839, 1585, 1354, 1277, 1125 cm^{-1}. **^{1}H-NMR (400 MHz, $CDCl_3$) δ:** 1.16 (d, J_P = 15.0 Hz, 9H), 1.28 (d, J_P = 8.1 Hz, 3H), 1.39 (s, 18H), 1.42 (d, J = 6.7 Hz, 3H), 1.66 – 1.89 (m, 2H), 2.08 – 2.25 (m, 3H), 2.29 – 2.41 (m, 1H), 2.48 – 2.62 (m, 2H), 3.15 – 3.24 (m, 1H), 3.27 – 3.42 (m, 1H), 3.66 – 3.73 (m, 1H), 3.96 (br s, 1H), 4.08 (qu, J = 7.3 Hz, 1H), 4.59 – 4.69 (m, 2H), 5.00 – 5.08 (m, 1H), 7.53 (s, 4H), 7.71 (s, 8H), 7.78 (s, 1H), 8.05 (s, 2H) ppm. **^{13}C-NMR (101 MHz, $CDCl_3$) δ:** 8.7 (d, J_P = 38.7 Hz, CH_3), 13.3 (s, CH_3), 26.1 (s, CH_2), 26.6 (d, J_P = 5.6 Hz, 3xCH_3), 29.7 (d, J_P = 2.1 Hz, CH_2), 31.5 (s, 6xCH_3), 31.6 (d, J_P = 2.1 Hz, CH_2), 35.4 (s, 2xCq), 36.7 (s, CH_2), 38.4 (d, J_P = 27.9 Hz, Cq), 45.1 (s, CH_2), 61.4 (s, CH), 66.0 (s, CH), 70.2 (s, CH), 79.5 (s, CH), 90.8 (d, J_P = 12.6 Hz, CH), 97.8 (d, J_P = 12.2 Hz, CH), 117.6 (s, 4xCH), 122.1 (s, Cq), 124.7 (q, J_F = 272.6 Hz, 8xCF_3), 125.0 (s, 2xCH), 128.5 – 129.6 (m, 8xCq), 129.7 (s, CH), 134.9 (s, 8xCH), 152.3 (s, 2xCq), 161.9 (q, J_B = 49.8 Hz, 4xCq), 169.7 (s, Cq) ppm. **^{31}P-NMR (162 MHz, $CDCl_3$) δ:** 58.0 (s) ppm. **HRMS (ESI, infusion)**: calc for $[C_{32}H_{53}IrN_2OP]^+$: 705.3519, found 705.3522. Calc for $[C_{32}H_{12}BF_{24}]^-$: 863.0654, found 863.0661.

Synthesis of (S_P)-12

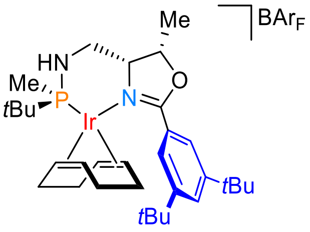

(S_P)-**12**

Following GP5, using (R_P)-**9c** (277.5 mg, 0.66 mmol), pyrrolidine (11.1 mL), $[Ir(cod)Cl]_2$ (222.7 mg, 0.33 mmol), DCM (11.1 mL) and $NaBAr_F$ (605.9 mg, 0.66 mmol) afforded (S_P)-**12** as an orange solid (777.6 mg, 75% yield). **[α]D:** + 48.3 (c 1.03, $CHCl_3$). **IR (ATR-FTIR) υ_{max}:** 2962, 2924, 2875, 2855, 1609, 1585, 1449, 1354, 1277, 1126 cm^{-1}. **^{1}H-NMR (400 MHz, $CDCl_3$) δ:** 1.10 (d, J_P = 14.8 Hz, 9H), 1.37 (d, J_P = 6.6 Hz, 3H), 1.40 (s, 9H), 1.45 (d, J = 6.5 Hz, 3H), 1.61 – 1.84 (m, 2H), 1.87 – 2.03 (m, 3H), 2.03 – 2.17 (m, 1H), 2.23 – 2.45 (m, 2H), 3.14 – 3.37 (m, 1H), 3.74 (tt, J = 15.4, 4.9 Hz, 1H), 3.88 (br s, 1H), 4.25 – 4.39 (m, 1H), 4.46 (br s, 1H), 4.64 (br s, 1H), 5.00 – 5.13 (m, 1H), 7.52 (s, 4H), 7.71 (s, 8H), 7.82 (t, J = 1.8 Hz, 1H), 8.19 (d, J = 1.8 Hz, 2H) ppm. **^{13}C-NMR (101**

MHz, $CDCl_3$) δ: 13.5 (d, J_P = 27.3 Hz, CH_3), 15.1 (s, CH_3), 25.3 (d, J_P = 3.8 Hz, 3xCH_3), 28.0 (s, CH_2), 29.6 (s, CH_2), 31.4 (s, 6xCH_3), 32.4 (s, CH_2), 33.9 (s, CH_2), 35.4 (s, 2xCq), 35.8 (d, J_P = 32.5 Hz, Cq), 44.0 (s, CH_2), 62.7 (s, CH), 63.2 (s, CH), 69.5 (s, CH), 79.9 (s, CH), 89.1 (d, J_P = 8.1 Hz, CH), 93.6 (d, J_P = 10.9 Hz, CH), 117.6 (s, 4xCH), 123.0 (s, Cq), 124.7 (s, 2xCH), 124.7 (q, J_F = 272.6 Hz, 8xCF_3), 128.4 – 129.7 (m, 8xCF_3), 129.9 (s, CH), 134.9 (s, 8xCH), 152.4 (s, 2xCq), 161.9 (q, J_B = 50.1 Hz, 4xCq) ppm. **^{31}P-NMR (162 MHz, $CDCl_3$) δ:** 60.5 (s) ppm. **HRMS (ESI, infusion)**: calc for $[C_{32}H_{53}IrN_2OP]^+$: 705.3519, found 705.3545. Calc for $[C_{32}H_{12}BF_{24}]^-$: 863.0654, found 863.0680.

Synthesis of (*R*$_P$)-13

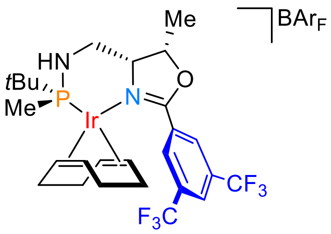

(*R*$_P$)-**13**

Following GP5, using (*S*$_P$)-**9d** (119.8 mg, 0.27 mmol), pyrrolidine (4.5 mL), $[Ir(cod)Cl]_2$ (91.0 mg, 0.14 mmol), DCM (4.5 mL) and NaBAr$_F$ (248.0 mg, 0.27 mmol) afforded (*R*$_P$)-**13** as an orange solid (300.0 mg, 70% yield). $[\alpha]_D$: + 34.3 (c 1.02, $CHCl_3$). **IR (ATR-FTIR) υ_{max}:** 2962, 2925, 2891, 2847, 1736, 1628, 1610, 1353, 1272, 1117 cm^{-1}. **^{1}H-NMR (400 MHz, $CDCl_3$) δ:** 1.03 (d, J_P = 15.3 Hz, 9H), 1.36 (d, J_P = 8.1 Hz, 3H), 1.41 (d, J = 6.7 Hz, 3H), 1.55 – 1.69 (m, 1H), 1.80 – 1.93 (m, 1H), 2.11 – 2.28 (m, 3H), 2.28 – 2.43 (m, 1H), 2.51 – 2.64 (m, 2H), 3.13 – 3.28 (m, 1H), 3.40 – 3.57 (m, 1H), 3.67 (qu, J = 7.1 Hz, 1H), 3.75 – 3.87 (m, 1H), 4.35 – 4.44 (m, 1H), 4.57 (td, J = 8.3, 3.7 Hz, 1H), 4.72 (br s, 1H), 5.07 – 5.18 (m, 1H), 7.52 (s, 4H), 7.70 (s, 8H), 8.24 (s, 1H), 9.13 (s, 2H) ppm. **^{13}C-NMR (101 MHz, $CDCl_3$) δ:** 9.6 (d, J_P = 35.1 Hz, CH_3), 13.6 (s, CH_3), 25.6 (s, CH_2), 26.2 (d, J_P = 5.2 Hz, 3xCH_3), 29.3 (s, CH_2), 32.0 (s, CH_2), 36.6 (s, CH_2), 37.6 (d, J_P = 30.0 Hz, Cq), 44.1 (s, CH_2), 66.3 (s, CH), 68.4 (s, CH), 70.7 (s, CH), 81.3 (s, CH), 90.3 (d, J_P = 12.9 Hz, CH), 97.2 (d, J_P = 11.4 Hz, CH), 117.6 (s, 4xCH), 122.6 (q, J_F = 273.3 Hz, 2xCF_3), 124.7 (q, J_F = 272.5 Hz, 8xCF_3), 124.7 (s, Cq), 127.7 – 128.0 (m, CH), 128.5 – 129.6 (m, 8xCq), 131.3 (s, 2xCH), 133.1 (q, J_F = 34.5 Hz, 2xCq), 134.9 (s, 8xCH), 161.8 (q, J_B = 50.1 Hz, 4xCq), 165.2 (s, Cq) ppm. **^{31}P-NMR (162 MHz, $CDCl_3$) δ:** 61.2 (s) ppm. **HRMS (ESI, infusion)**: calc for $[C_{26}H_{31}IrN_2OP]^+$: 729.2015, found 705.2015. Calc for $[C_{32}H_{12}BF_{24}]^-$: 863.0654, found 863.0671.

Synthesis of (S_P)-13

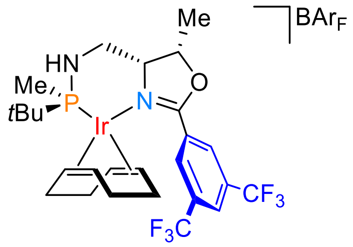

(S_P)-**13**

Following GP5, using (R_P)-**9d** (64.5 mg, 0.15 mmol), pyrrolidine (2.4 mL), [Ir(cod)Cl]$_2$ (49.0 mg, 0.07 mmol), DCM (2.4 mL) and NaBAr$_F$ (133.0 mg, 0.15 mmol) afforded (S_P)-**13** as an orange solid (139.0 mg, 60% yield). **[α]$_D$:** + 62.4 (c 1.17, CHCl$_3$). **IR (ATR-FTIR) υ_{max}:** 2963, 2925, 2878, 2850, 1736, 1629, 1609, 1535, 1469, 1353, 1272, 1112 cm^{-1}. **^{1}H-NMR (400 MHz, CDCl$_3$) δ:** 1.09 (d, J_P = 15.0 Hz, 9H), 1.31 (d, J_P = 6.3 Hz, 3H), 1.36 (d, J = 6.7 Hz, 3H), 1.55 – 1.69 (m, 1H), 1.76 – 1.96 (m, 2H), 1.97 – 2.13 (m, 2H), 2.20 – 2.33 (m, 3H), 2.46 – 2.57 (m, 1H), 3.26 – 3.41 (m, 1H), 3.65 – 3.76 (m, 1H), 3.78 – 3.91 (m, 1H), 4.43 – 4.46 (m, 2H), 4.76 – 4.87 (m, 2H), 5.10 – 5.21 (m, 1H), 7.52 (s, 4H), 7.69 (s, 8H), 8.27 (s, 1H), 9.30 (s, 2H) ppm. **^{13}C-NMR (101 MHz, CDCl$_3$) δ:** 13.7 (d, J_P = 30.8 Hz, CH$_3$), 15.7 (s, CH$_3$), 25.1 (d, J_P = 3.8 Hz, 3xCH$_3$), 26.6 (s, CH$_2$), 31.0 (s, 2xCH$_2$), 35.1 (s, CH$_2$), 36.4 (d, J_P = 30.3 Hz, Cq), 44.3 (s, CH$_2$), 64.2 (s, CH), 66.9 (s, CH), 69.7 (s, CH), 81.3 (s, CH), 88.9 (d, J_P = 13.8 Hz, CH), 94.1 (d, J_P = 10.8 Hz, CH), 117.6 (s, 4xCH), 122.6 (q, J_F = 273.5 Hz, 2xCF$_3$), 124.7 (q, J_F = 272.6 Hz, 8xCF$_3$), 125.4 (s, Cq), 127.8 – 128.0 (m, CH), 128.5 – 129.6 (m, 8xCq), 131.1 (s, 2xCH), 133.2 (q, J_F = 34.6 Hz, 2xCq), 134.9 (s, 8xCH), 161.8 (q, J_B = 50.0 Hz, 4xCq), 165.0 (s, Cq) ppm. **^{31}P-NMR (162 MHz, CDCl$_3$) δ:** 62.5 (s) ppm. **HRMS (ESI, infusion)**: calc for [$C_{26}H_{31}IrN_2OP$]$^+$: 729.2015, found 705.2010. Calc for [$C_{32}H_{12}BF_{24}$]$^-$: 863.0654, found 863.0646.

Synthesis of (R_P)-14

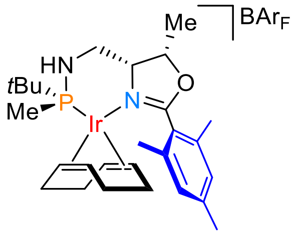

(R_P)-**14**

Following GP5, using (S_P)-**9e** (30.7 mg, 0.09 mmol), pyrrolidine (1.5 mL), [Ir(cod)Cl]$_2$ (29.6 mg, 0.05 mmol), DCM (1.5 mL) and NaBAr$_F$ (80.5 mg, 0.09 mmol) afforded (R_P)-**14** as a red solid (96.2

mg, 73% yield). [α]D: - 11.2 (c 0.80, $CHCl_3$). **IR (ATR-FTIR) υ_{max}:** 2958, 2925, 2847, 2025, 1609, 1480, 1354, 1277, 1125 cm^{-1}. **^{1}H-NMR (400 MHz, $CDCl_3$) δ:** 1.14 (d, J_P = 14.7 Hz, 9H), 1.33 – 1.53 (m, 3H), 1.39 (d, J = 7.3 Hz, 6H), 1.76 – 1.85 (m, 1H), 1.93 – 2.19 (m, 5H), 2.23 (s, 3H), 2.32 (s, 3H), 2.53 (s, 3H), 3.17 – 3.32 (m, 1H), 3.32 – 3.54 (m, 2H), 3.58 – 3.70 (m, 1H), 3.86 – 3.94 (m, 1H), 4.45 (td, J = 9.9, 2.4 Hz, 1H), 4.60 – 4.68 (m, 1H), 4.96 – 5.15 (m, 1H), 6.93 (d, J = 16.2 Hz, 2H), 7.54 (s, 4H), 7.71 (s, 8H) ppm. **^{13}C-NMR (101 MHz, $CDCl_3$) δ:** 13.0 (d, J_P = 37.1 Hz, CH_3), 13.4 (s, CH_3), 20.5 (s, CH_3), 21.4 (s, CH_3), 22.4 (s, CH_3), 27.0 (d, J_P = 4.9 Hz, 3xCH_3), 27.5 (s, CH_2), 30.8 (s, CH_2), 31.2 (s, CH_2), 34.9 (d, J_P = 4.9 Hz, CH_2), 38.9 (d, J_P = 29.1 Hz, Cq), 45.8 (s, CH_2), 61.0 (s, CH), 64.2 (s, CH), 70.1 (d, J_P = 3.8 Hz, CH), 79.0 (s, CH), 90.2 (d, J_P = 13.4 Hz, CH), 93.0 (d, J_P = 11.8 Hz, CH), 117.4 – 117.8 (m, 4xCH), 122.5 (s, Cq), 124.7 (q, J_F = 272.6 Hz, 8xCF_3), 128.5 – 129.6 (m, 8xCq), 129.0 (s, 2xCH), 135.0 (s, 8xCH), 138.40 (s, Cq), 143.0 (s, 2xCq), 161.9 (q, J_B = 50.5 Hz, 4xCq), 173.3 (s, Cq) ppm. **^{31}P-NMR (162 MHz, $CDCl_3$) δ:** 53.5 (s) ppm. **HRMS (ESI, infusion)**: calc for $[C_{27}H_{43}IrN_2OP]^+$: 635.2736, found 635.2731. Calc for $[C_{32}H_{12}BF_{24}]^-$: 863.0654, found 863.0667.

Synthesis of (S_P)-14

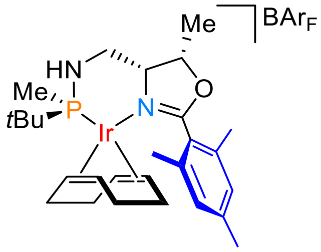

(S_P)-**14**

Following GP5, using (R_P)-**9e** (86.8 mg, 0.25 mmol), pyrrolidine (4.2 mL), [Ir(cod)Cl]$_2$ (83.7 mg, 0.13 mmol), DCM (4.2mL) and NaBAr$_F$ (227.7 mg, 0.25 mmol) afforded (S_P)-**14** as a red solid (160.5 mg, 43% yield). [α]D: + 8.2 (c 1.03, $CHCl_3$). **IR (ATR-FTIR) υ_{max}:** 2960, 2926, 2027, 1610, 1480, 1354, 1277, 1126 cm^{-1}. **^{1}H-NMR (400 MHz, $CDCl_3$) δ:** 1.11 (d, J_P = 14.8 Hz, 9H), 1.38 (d, J = 6.7 Hz, 3H), 1.44 (d, J_P = 7.4 Hz, 3H), 1.47 – 1.54 (m, 1H), 1.59 – 1.68 (m, 1H), 1.85 – 2.15 (m, 7H), 2.25 (s, 3H), 2.32 (s, 3H), 2.53 (s, 3H), 3.31 – 3.46 (m, 2H), 3.70 – 3.77 (m, 1H), 3.78 – 3.87 (m, 2H), 4.42 – 4.52 (m, 2H), 4.94 – 5.06 (m, 1H), 6.94 (d, J = 17.5 Hz, 2H), 7.53 (s, 4H), 7.71 (s, 8H) ppm. **^{13}C-NMR (101 MHz, $CDCl_3$) δ:** 12.3 (d, J_P = 33.7 Hz, CH_3), 12.7 (s, CH_3), 20.7 (s, CH_3), 21.4 (s, CH_3), 21.5 (s, CH_3), 26.3 (d, J_P = 4.5 Hz, 3xCH_3), 27.4 (s, CH_2), 30.4 (s, CH_2), 31.6 (s, CH_2), 34.1 (s, CH_2), 36.9 (d, J_P = 32.6 Hz, Cq), 43.6 (s, CH_2), 59.4 (s, CH), 63.0 (d, J_P = 5.0 Hz, CH), 79.4

(s, CH), 91.1 (d, J_P = 13.4 Hz, CH), 94.8 (d, J_P = 11.1 Hz), 117.6 (s, 4xCH), 122.3 (s, Cq), 124.7 (q, J_F = 272.7 Hz, 8xCF_3), 128.5 – 129.7 (m, 8xCq), 129.5 (s, CH), 129.6 (s, CH), 135.0 (s, 8xCH), 138.3 (s, Cq), 143.4 (s, 2xCq), 161.9 (q, J_B = 49.8 Hz, 4xCq), 174.5 (s, Cq) ppm. **^{31}P-NMR (162 MHz, $CDCl_3$) δ:** 54.4 (s) ppm. **HRMS (ESI, infusion)**: calc for $[C_{27}H_{43}IrN_2OP]^+$: 635.2736, found 635.2731. Calc for $[C_{32}H_{12}BF_{24}]^-$: 863.0654, found 863.0666.

Synthesis of (*R*$_P$)-15

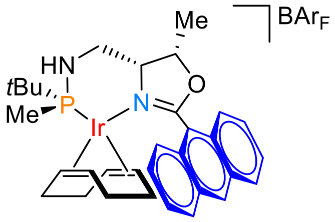

(R_P)-**15**

Following GP5, using (S_P)-**9f** (48.9 mg, 0.12 mmol), pyrrolidine (2 mL), $[Ir(cod)Cl]_2$ (40.4 mg, 0.06 mmol), DCM (2 mL) and NaBAr$_F$ (110.0 mg, 0.12 mmol) afforded (R_P)-**15** as a red solid (133.3 mg, 71% yield). **$[\alpha]_D$:** - 67.8 (c 1.01, $CHCl_3$). **IR (ATR-FTIR) υ_{max}:** 2949, 2924, 2874, 2855, 1593, 1468, 1353, 1272, 1113 cm^{-1}. **^{1}H-NMR (400 MHz, $CDCl_3$) δ:** 0.60 – 0.72 (m, 1H), 0.86 – 1.00 (m, 1H), 1.03 – 1.52 (m, 4H), 1.22 (d, J_P = 14.7 Hz, 9H), 1.40 (d, J_P = 7.8 Hz, 3H), 1.56 (d, J = 7.3 Hz, 3H), 1.86 – 2.02 (m, 2H), 2.43 – 2.56 (m, 2H), 3.51 – 3.67 (m, 4H), 4.54 (br s, 1H), 4.61 – 4.75 (m, 1H), 5.43 – 5.52 (m, 1H), 7.51 – 7.66 (m, 4H), 7.54 (s, 4H), 7.73 (s, 8H), 7.75 – 7.81 (m, 1H), 7.97 (d, J = 8.7 Hz, 1H), 8.12 (dd, J = 23.2, 8.3 Hz, 2H), 8.73 (s, 1H) ppm. **^{13}C-NMR (101 MHz, $CDCl_3$) δ:** 12.3 (d, J_P = 38.8 Hz, CH_3), 13.8 (s, CH_3), 27.1 (d, J_P = 5.2 Hz, 3xCH_3), 27.6 (s, CH_2), 29.9 (s, CH_2), 31.1 (d, J_P = 18.0 Hz, CH_2), 33.8 (d, J_P = 3.6 Hz, CH_2), 39.1 (d, J_P = 28.1 Hz, Cq), 46.3 (CH_2), 62.0 (s, CH), 64.7 (s, CH), 70.8 (d, J_P = 3.3 Hz, CH), 80.2 (s, CH), 88.1 (d, J_P = 14.4 Hz, CH), 91.2 (d, J_P = 11.2 Hz, CH), 117.6 (s, 4xCH), 118.4 (s, Cq), 123.0 (s, CH), 123.8 (s, CH), 124.7 (q, J_F = 272.6 Hz, 8xCF_3), 126.5 (s, CH), 126.6 (s, CH), 128.3 (s, CH), 128.9 (s, CH), 128.5 – 129.7 (m, 8xCq), 129.5 (s, CH), 129.6 (s, CH), 130.6 (s, 3xCq), 131.0 (s, Cq), 132.7 (s, CH), 135.0 (s, 8xCH), 161.9 (q, J_B = 49.8 Hz, 4xCq), 172.2 (s, Cq) ppm. **^{31}P-NMR (162 MHz, $CDCl_3$) δ:** 55.3 (s) ppm. **HRMS (ESI, infusion)**: calc for $[C_{32}H_{41}IrN_2OP]^+$: 693.2580, found 693.2575. Calc for $[C_{32}H_{12}BF_{24}]^-$: 863.0654, found 863.0674.

Synthesis of (S_P)-15

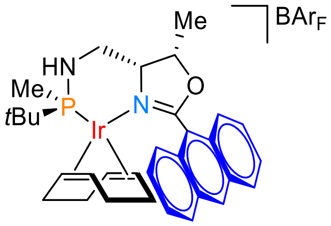

(S_P)-**15**

Following GP5, using (R_P)-**9f** (68.7 mg, 0.17 mmol), pyrrolidine (2.8 mL), [Ir(cod)Cl]$_2$ (57.0 mg, 0.08 mmol), DCM (2.8 mL) and NaBAr$_F$ (155.0 mg, 0.17 mmol) afforded (S_P)-**15** as a red solid (99.5 mg, 38% yield). **[α]$_D$:** - 9.0 (c 0.92, CHCl$_3$). **IR (ATR-FTIR) υ_{max}:** 2961, 2925, 2889, 2852, 1608, 1586, 1459, 1352, 1272, 1113 cm^{-1}. **^{1}H-NMR (400 MHz, CDCl$_3$) δ:** 0.49 – 0.58 (m, 1H), 0.93 – 1.10 (m, 2H), 1.17 (d, J_P = 14.7 Hz, 9H), 1.22 – 1.34 (m, 1H), 1.56 – 1.60 (m, 1H), 1.58 (d, J = 6.7 Hz, 3H), 1.62 (d, J_P = 7.3 Hz, 3H), 1.64 – 1.78 (m, 1H), 1.83 – 2.09 (m, 2H), 2.38 (br s, 1H), 2.57 – 2.66 (m, 1H), 3.51 – 3.84 (m, 4H), 4.49 (br s, 1H), 4.69 – 4.75 (m, 1H), 5.35 – 5.48 (m, 1H), 7.54 (s, 4H), 7.56 – 7.68 (m, 4H), 7.72 (s, 8H), 7.80 (d, J = 8.7 Hz, 1H), 8.11 (d, J = 8.4 Hz, 1H), 8.12 – 8.22 (m, 2H), 8.76 (s, 1H) ppm. **^{13}C-NMR (101 MHz, CDCl$_3$) δ:** 12.6 (d, J_P = 33.4 Hz, CH$_3$), 12.9 (s, CH$_3$), 26.1 (s, CH$_2$), 26.6 (d, J_P = 4.5 Hz, 3xCH$_3$), 30.2 (s, CH$_2$), 31.9 (s, CH$_2$), 34.4 (d, J_P = 3.7 Hz, CH$_2$), 37.2 (d, J_P = 30.4 Hz, Cq), 43.5 (s, CH$_2$), 59.0 (s, CH), 65.7 (s, CH), 67.1 (s, CH), 80.8 (s, CH), 88.2 (d, J_P = 15.4 Hz, CH), 94.1 (d, J_P = 10.0 Hz, CH), 117.6 (s, 4xCH), 118.2 (s, Cq), 123.6 (s, CH), 123.7 (s, CH), 124.7 (q, J_F = 272.5 Hz, 8xCF$_3$), 126.5 (s, CH), 126.6 (s, CH), 128.1 (s, CH), 128.5 -129.7 (m, 8xCq, CH), 129.2 (s, CH), 129.5 (s, 2xCH), 130.6 (s, Cq), 130.8 (s, Cq), 131.2 (s, 2xCq), 133.3 (s, CH), 135.0 (s, 8xCH), 161.9 (q, J_B = 49.8 Hz, 4xCq), 172.8 (s, Cq) ppm. **^{31}P-NMR (162 MHz, CDCl$_3$) δ:** 55.5 (s) ppm. **HRMS (ESI, infusion)**: calc for [$C_{32}H_{41}IrN_2OP$]$^+$: 693.2580, found 693.2594. Calc for [$C_{32}H_{12}BF_{24}$]$^-$: 863.0654, found 863.0683.

2.2.4 Preparation of dicyclohexylphosphine catalyst 17

General synthetic scheme for the preparation of **17**:

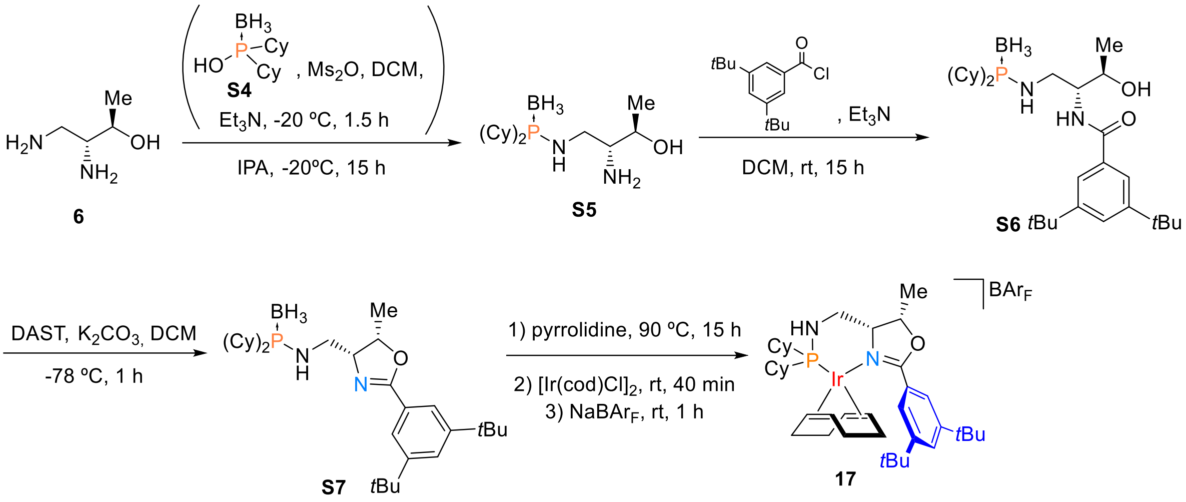

Preparation of dicyclohexyl phosphinous acid **S4** was performed as previously described by our group.[12]

Synthesis of aminophosphine S5

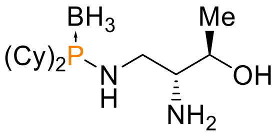

S5

A solution of methanesulfonic anhydride (518 mg, 2.88 mmol) in DCM (6 mL) was prepared in an oven-dried 100 mL round-bottom flask and cooled down to -20 °C. Then, dicyclohexyl phosphinous acid **S4** (547.6 mg, 2.40 mmol) was dissolved in DCM (2 mL) and slowly added to the first solution (for more information regarding the preparation of **S4**, see ref. 12). Triethylamine (0.84 mL, 6.00 mmol) was subsequently added to the mixture dropwise and the reaction was stirred at -20 °C for 1.5 h. After, a solution of **6** (500.0 mg, 4.80 mmol) in IPA (3.5 mL) was slowly added and the reaction was left to stir at the same temperature for 15 h. After that time, NaOH 1M was added to the solution and it was warmed to room temperature. The solution was diluted with DCM, the organic layer was separated and the aqueous phase was extracted thrice with DCM. The combined extracts were dried over $MgSO_4$, filtered and concentrated under vacuum, obtaining **S5** as a white solid without need of further purification (493.3 mg, 65% yield). **M.p.**: 79 – 81 °C. **[α]$_D$:** + 5.6 (c 1.06, $CHCl_3$). **IR (ATR-FTIR) υ_{max}:** 3340, 3320, 2925, 2850, 2363, 1541 cm^{-1}. **^{1}H-NMR (400 MHz, $CDCl_3$) δ:** 0.00 – 0.84 (m, BH_3), 1.20 (d, *J* = 6.3 Hz, 3H), 1.21 – 1.44 (m, 6H), 1.58 – 1.91 (m, 18H), 2.55 (dt, *J* = 7.1, 4.8 Hz, 1H), 2.90 (dq, *J* = 13.5, 6.8 Hz, 1H), 3.04 – 3.17 (m, 1H), 3.63 (qd, *J* = 6.3, 4.8 Hz, 1H) ppm.

^{13}**C-NMR (101 MHz, CDCl$_3$) δ:** 20.5 (s, CH$_3$), 25.6 – 26.9 (m, 10xCH$_2$), 34.6 (dd, J_P = 38.3, 19.8 Hz, 2xCH), 47.1 (s, CH$_2$), 58.3 (s, CH), 68.1 (s, CH) ppm. ^{31}**P-NMR (162 MHz, CDCl$_3$) δ:** 69.4 – 72.4 (m) ppm. **HRMS (ESI)**: calc for [$C_{16}H_{36}BN_2OP$+H]$^+$: 315.2731, found 315.2737.

Synthesis of phosphinooxazoline S7

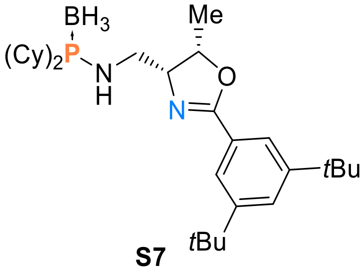

S7

Initially, the corresponding acid chloride was prepared by adding oxalyl chloride (0.77 mL, 2.17 mmol) and one drop of DMF to a solution of 3,5-di-*tert*-butyl benzoic acid (424.2 mg, 1.77 mmol) in DCM (6 mL) at 0 °C. The solution was stirred at room temperature for 3 h and then evaporated and dried under vacuum for 2 h. Afterwards, following GP4, using **S5** (557.5 mg, 1.77 mmol), DCM (11.7 mL), Et$_3$N (0.49 mL, 3.55 mmol) and 3,5-di-*tert*-butyl benzoyl chloride (447.3 mg, 1.77 mmol) afforded **S6** as a white solid (681.3 mg, 72% yield). ^{1}**H-NMR (400 MHz, CDCl$_3$) δ:** 0.03 – 0.82 (m, BH$_3$), 0.69 – 1.24 (m, 8H), 1.26 (d, J = 6.5 Hz, 4H), 1.35 (s, 18H), 1.60 – 1.88 (m, 14H), 2.76 (br s, 1H), 3.26 (q, J = 7.1 Hz, 2H), 3.91 (q, J = 7.4 Hz, 1H), 4.22 (br s, 1H), 6.65 (d, J = 8.3 Hz, 1H), 7.58 (t, J = 1.8 Hz, 1H), 7.62 (d, J = 1.8 Hz, 2H) ppm.

Consecutively, following GP4, **S6** (668.8 mg, 1.26 mmol), K$_2$CO$_3$ (696.9 mg, 5.04 mmol), DCM (15.5 mL) and DAST (428.0 mg, 2.52 mmol) were used. Purification by silica column chromatography (hexanes:EtOAc 97:3) afforded **S7** as a white solid (498.6 mg, 77% yield). **M.p.**: 142 – 144 °C. **[α]$_D$:** + 88.0 (c 0.87, CHCl$_3$). **IR (ATR-FTIR) $υ_{max}$**: 3353, 2924, 2849, 2367, 1647, 1636 cm^{-1}. ^{1}**H-NMR (400 MHz, CDCl$_3$) δ:** 0.05 – 0.80 (m, BH$_3$), 1.13 – 1.49 (m, 8H), 1.35 (s, 18H), 1.39 (d, J = 6.7 Hz, 3H), 1.63 – 2.02 (m, 14H), 2.93 – 3.06 (m, 1H), 3.17 – 3.32 (m, 1H), 4.20 (td, J = 9.1, 4.2 Hz, 1H), 4.79 – 4.92 (m, 1H), 7.56 (t, J = 1.8 Hz, 1H), 7.78 (d, J = 1.8 Hz, 2H) ppm. ^{13}**C-NMR (101 MHz, CDCl$_3$) δ:** 15.0 (s, CH$_3$), 25.4 – 27.1 (m, 10xCH$_2$), 31.5 (s, 6xCH$_3$), 34.4 (d, J_P = 40.0 Hz, CH), 34.9 (d, J_P = 37.6 Hz, CH), 35.1 (s, 2xCq), 44.3 (s, CH$_2$), 69.8 (d, J_P = 6.2 Hz, CH), 78.1 (s, CH), 122.6 (s, 2xCH), 125.9 (s, CH), 127.3 (s, Cq), 151.1 (s, 2xCq), 164.9 (s, Cq) ppm. ^{31}**P-NMR (162 MHz, CDCl$_3$) δ:** 68.8 – 71.8 (m) ppm. **HRMS (ESI)**: calc for [$C_{31}H_{54}BN_2OP$+H]$^+$: 513.4139, found: 513.4151.

Synthesis of catalyst 17

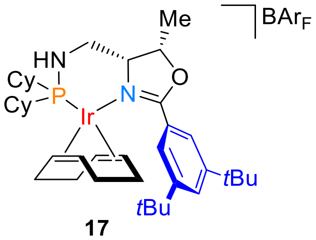

17

Following GP5, using **S7** (100.0 mg, 0.20 mmol), pyrrolidine (3.3 mL), [Ir(cod)Cl]$_2$ (65.5 mg, 0.098 mmol), DCM (3.3 mL) and NaBAr$_F$ (178.2 mg, 0.20 mmol) afforded **17** as an orange solid (260.0 mg, 80% yield). **[α]$_D$:** + 20.7 (c 1.01, CHCl$_3$). **IR (ATR-FTIR) υ$_{max}$:** 2957, 2925, 2822, 1540, 1353, 1275, 1120 cm^{-1}. **^{1}H-NMR (400 MHz, CDCl$_3$) δ:** 1.16 – 1.50 (m, 9H), 1.38 – 1.41 (m, 21H), 1.56 – 1.58 (m, 2H), 1.62 – 1.72 (m, 2H), 1.70 – 1.83 (m, 5H), 1.83 – 1.97 (m, 6H), 2.00 – 2.19 (m, 2H), 2.19 – 2.39 (m, 3H), 2.41 – 2.53 (m, 1H), 3.24 – 3.35 (m, 1H), 3.47 – 3.61 (m, 3H), 4.41 (br s, 1H), 4.44 – 4.52 (m, 1H), 4.72 (br s, 1H), 4.94 – 5.03 (m, 1H), 7.52 (s, 4H), 7.68 – 7.73 (m, 8H), 7.81 (t, *J* = 1.8 Hz, 1H), 7.96 (d, *J* = 1.8 Hz, 2H) ppm. **^{13}C-NMR (101 MHz, CDCl$_3$) δ:** 13.1 (s, CH$_3$), 25.6 – 26.9 (m, 9xCH$_2$), 28.6 (s, CH$_2$), 29.4 (d, J_P = 8.3 Hz, CH$_2$), 30.1 (s, CH$_2$), 31.5 (s, 6xCH$_3$), 31.7 (s, CH$_2$), 35.6 (s, 2xCq), 36.0 (d, J_P = 34.5 Hz, CH), 36.4 (d, J_P = 3.0 Hz, CH$_2$), 42.5 (d, J_P = 29.1 Hz, CH), 45.2 (s, CH$_2$), 63.7 (s, CH), 64.7 (s, CH), 68.4 (d, J_P = 3.0 Hz, CH), 79.6 (s, CH), 87.4 (d, J_P = 14.4 Hz, CH), 95.8 (d, J_P = 9.5 Hz, CH), 117.6 (s, 4xCH), 123.4 (s, Cq), 124.5 (s, 2xCH), 124.7 (q, J_F = 272.6 Hz, 8xCF$_3$), 128.4 – 129.7 (m, 8xCF$_3$), 129.8 (s, CH), 134.9 (s, 8xCH), 152.3 (s, 2xCq), 161.9 (q, J_B = 50.1 Hz, 4xCq), 171.2 (s, Cq) ppm. **^{31}P-NMR (162 MHz, CDCl$_3$) δ:** 57.6 (s) ppm. **HRMS (ESI, infusion)**: calc for [$C_{39}H_{63}IrN_2OP$]$^+$: 799.4301, found 799.4293. Calc for [$C_{32}H_{12}BF_{24}$]$^-$: 863.0654, found 863.0664.

2.3 Asymmetric hydrogenation of 2,3-diarylallyl amines 3a-p

- All solid allylic substrates were recrystallized in hexanes:Et$_2$O previous to the asymmetric hydrogenation.
- To find suitable HPLC separation conditions, racemates of compounds **19a-c,e-j,n-p** were prepared by treating the corresponding allyl amines **3** with 20 mol% of Pd/C in DCM at 15 bar of H$_2$, for 15 h. Afterwards, reaction crudes were filtered through a small plug of silica to afford analytically pure compounds. Alternatively, racemate of **19d** was prepared by treating **3d** with 3 mol% of Crabtree's catalyst in DCM at 50 bar of H$_2$. Racemates of **19k-m** were

prepared by treating **3k-m** with 5 mol% of Pd/C in EtOH, using a H_2 baloon and short reaction times to avoid dechlorination (reaction control by ^{1}H-NMR).

General procedure for the asymmetric hydrogenation of 2,3-diarylallyl amines 3 at 1 mol% (GP6):

The corresponding substrate (1 eq.), catalyst (S_P)-**12** and a PTFE-coated stir-bar were placed in a glass tube inside a stainless steel high pressure reactor. The reactor was entered into a N_2-filled glove box and anhydrous DCM (0.3 M) was added. The reactor was closed, removed from the glove box and connected to a hydrogen manifold. The connection was purged with vacuum-nitrogen cycles, the valve of the reactor was opened, it was evacuated and then charged at 50 bar of hydrogen pressure. The valve was closed, the hydrogen manifold was unplugged and the mixture was left to stir at room temperature for 24 or 48 hours. The reactor was then depressurized, the solvent was evaporated and the conversion of the reaction was determined by ^{1}H-NMR. The crude was purified by silica column chromatography (eluting with hexanes:EtOAc), affording the desired product. The enantiomeric excess was determined by chiral HPLC chromatography.

***tert*-Butyl (*R*)-(2-(4-methoxyphenyl)-3-phenylpropyl)carbamate, 19a**

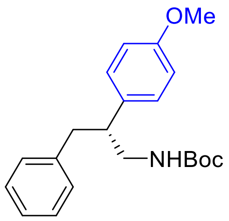

19a

Following GP6, using substrate **3a** (65.0 mg, 0.19 mmol) and catalyst (S_P)-**12** (3.0 mg, $1.9 \cdot 10^{-3}$ mmol). The reaction was stopped after 24 h and gave 100% conversion. Purification by silica column chromatography (hexanes:EtOAc 9:1) afforded **19a** as a white solid (59.7 mg, 91% yield, 99% ee). **M.p.:** 93 – 95 °C. **[α]$_D$:** - 21.7 (c 1.00, $CHCl_3$). **IR (ATR-FTIR) υ_{max}:** 3346, 2976, 2365, 1667, 1514 cm^{-1}. **^{1}H-NMR (400 MHz, $CDCl_3$) δ**: 1.38 (s, 9H), 2.75 – 2.95 (m, 2H), 2.95 – 3.05 (m, 2H), 3.12 – 3.26 (m, 1H), 3.46 – 3.60 (m, 1H), 3.78 (s, 3H), 4.32 (br s, 1H), 6.78 – 6.85 (m, 2H), 6.97 – 7.07 (m, 4H), 7.13 (t, J = 7.3 Hz, 1H), 7.19 (t, J = 7.3 Hz, 2H) ppm. **^{13}C-NMR (101 MHz, $CDCl_3$) δ:** 28.5, 41.0, 45.6, 47.1, 55.4, 79.4, 114.1, 126.1, 128.3, 129.0, 129.2, 134.1, 139.8, 156.0, 158.5 ppm. **HRMS (ESI):** calc for $[C_{21}H_{27}NO_3+Na]^+$: 364.1883, found 364.1884. **HPLC**: Chiralpak IA, heptane:EtOH 98:2, 0.5 mL/min, λ = 210 nm, t_R (R) = 18.1 min, t_R (S) = 20.4 min.

***tert*-Butyl (*R*)-(2-(4-chlorophenyl)-3-phenylpropyl)carbamate, 19b**

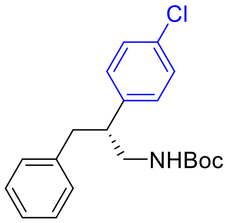

19b

Following GP6, using substrate **3b** (66.6 mg, 0.19 mmol) and catalyst (S_P)-**12** (3.2 mg, $1.9 \cdot 10^{-3}$ mmol). The reaction was stopped after 24 h and gave 100% conversion. Purification by silica column chromatography (hexanes:EtOAc 85:15) afforded **19b** as a white solid (62.3 mg, 93% yield, 99% ee). **M.p.:** 94 – 98 °C. $[\alpha]_D$: - 28.6 (c 0.90, $CHCl_3$). **IR (ATR-FTIR)** υ_{max}**:** 3332, 2971, 2917, 2851, 1697, 1678, 1518, 1492, 1367, 1290, 1275 cm^{-1}. **^{1}H-NMR (400 MHz, $CDCl_3$) δ**: 1.39 (s, 9H), 2.77 – 2.87 (m, 1H), 2.91 – 3.00 (m, 1H), 3.07 (br s, 1H), 3.24 (br s, 1H), 3.45 – 3.61 (m, 1H), 4.33 (br s, 1H), 6.90 – 7.09 (m, 4H), 7.10 – 7.28 (m, 5H) ppm. **^{13}C-NMR (101 MHz, $CDCl_3$) δ:** 28.3, 40.5, 45.4, 47.3, 79.3, 126.1, 128.3, 128.7, 129.0, 129.3, 132.4, 139.1, 140.5, 156.0 ppm. **HRMS (ESI):** calc for $[C_{20}H_{24}ClNO_2+Na]^+$: 368.1388, found 368.1397. **HPLC**: Chiralpak IA, heptane:IPA 98:2, 0.5 mL/min, λ = 210 nm, t_R (*R*) = 30.9 min, t_R (*S*) = 36.3 min.

***tert*-Butyl (*R*)-(2-(4-fluorophenyl)-3-phenylpropyl)carbamate, 19c**

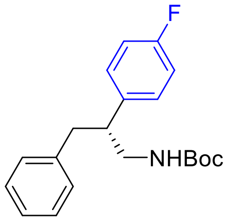

19c

Following GP6, using substrate **3c** (54.1 mg, 0.17 mmol) and catalyst (S_P)-**12** (2.6 mg, $1.7 \cdot 10^{-3}$ mmol). The reaction was stopped after 48 h and gave 100% conversion. Purification by silica column chromatography (hexanes:EtOAc 93:7) afforded **19c** as a white solid (49.0 mg, 90% yield, 98% ee). **M.p.:** 56 – 58 °C. $[\alpha]_D$: - 20.8 (c 0.61, $CHCl_3$). **IR (ATR-FTIR)** υ_{max}**:** 3446, 2980, 2930, 1701, 1508, 1224 cm^{-1}. **^{1}H-NMR (400 MHz, $CDCl_3$) δ**: 1.38 (s, 9H), 2.82 (dd, *J* = 13.5, 8.5 Hz, 1H), 2.95 (dd, *J* = 13.5, 6.3 Hz, 1H), 3.02 – 3.13 (m, 1H), 3.17 – 3.32 (m, 1H), 3.48 – 3.62 (m, 1H), 4.33 (br s, 1H), 6.92 – 7.03 (m, 4H), 7.03 – 7.11 (m, 2H), 7.11 – 7.22 (m, 3H) ppm. **^{13}C-NMR (101 MHz, $CDCl_3$) δ:** 28.5, 40.8, 45.6, 47.3, 79.4, 115.5 (d, J_F = 21.1 Hz), 126.3, 128.4, 129.1, 129.5 (d, J_F = 7.8 Hz), 137.8, 139.4, 155.9, 161.8 (d, J_F = 244.7 Hz) ppm. **HRMS (ESI):** calc for $[C_{20}H_{24}FNO_2+Na]^+$:

352.1683, found 352.1687. **HPLC**: Chiralpak IA, heptane:IPA 98:2, 0.5 mL/min, λ = 210 nm, t_R (*R*) = 22.0 min, t_R (*S*) = 26.1 min.

***tert*-Butyl (*R*)-(2-(4-benzoylphenyl)-3-phenylpropyl)carbamate, 19d**

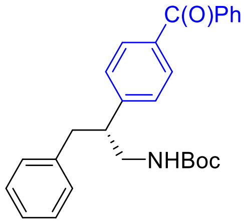

19d

Following GP6, using substrate **3d** (49.6 mg, 0.12 mmol) and catalyst (S_P)-**12** (1.9 mg, 1.2·10^{-3} mmol). The reaction was stopped after 48 h and gave 100% conversion. Purification by silica column chromatography (hexanes:EtOAc 85:15) afforded **19d** as a white solid (47.9 mg, 96% yield, 98% ee). **M.p.:** 113 – 115 °C. $[\alpha]_D$: - 35.5 (c 1.16, $CHCl_3$). **IR (ATR-FTIR)** υ_{max}**:** 3449, 3373, 2965, 2927, 1697, 1654, 1507, 1366, 1278 cm^{-1}. **^{1}H-NMR (400 MHz, $CDCl_3$) δ**: 1.39 (s, 9H), 2.91 (dd, *J* = 13.7, 8.3 Hz, 1H), 3.02 (dd, *J* = 13.8, 6.3 Hz, 1H), 3.14 – 3.26 (m, 1H), 3.27 – 3.38 (m, 1H), 3.52 – 3.64 (m, 1H), 4.39 (br s, 1H), 7.04 (d, *J* = 7.3 Hz, 2H), 7.12 – 7.26 (m, 5H), 7.48 (t, *J* = 7.5 Hz, 2H), 7.59 (t, *J* = 7.4 Hz, 1H), 7.76 (dd, *J* = 17.9, 7.5 Hz, 4H) ppm. **^{13}C-NMR (101 MHz, $CDCl_3$) δ:** 28.3, 40.3, 45.3, 48.0, 79.4, 126.2, 127.9, 128.2, 128.3, 129.0, 130.0, 130.4, 132.3, 136.1, 137.6, 139.0, 147.2, 155.7, 196.3 ppm. **HRMS (ESI):** calc for $[C_{27}H_{29}NO_3+Na]^+$: 438.2040, found 438.2031. **HPLC**: Chiralpak IA, heptane:EtOH 9:1, 0.5 mL/min, λ = 210 nm, t_R (*R*) = 18.2 min, t_R (*S*) = 21.0 min.

Methyl (*R*)-4-(1-((*tert*-butoxycarbonyl)amino)-3-phenylpropan-2-yl)benzoate, 19e

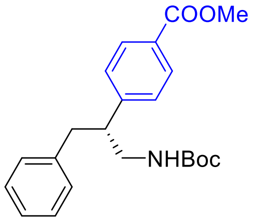

19e

Following GP6, using substrate **3e** (52.6 mg, 0.14 mmol) and catalyst (S_P)-**12** (2.3 mg, 1.4·10^{-3} mmol). The reaction was stopped after 48 h and gave 99% conversion. Purification by silica column chromatography (hexanes:EtOAc 8:2) afforded **19e** as a white solid (46.2 mg, 87% yield, 98% ee). **M.p.:** 107 – 109 °C. $[\alpha]_D$: - 30.2 (c 1.05, $CHCl_3$). **IR (ATR-FTIR)** υ_{max}**:** 3439, 3373, 2977, 2927, 1704, 1506, 1279, 1249 cm^{-1}. **^{1}H-NMR (400 MHz, $CDCl_3$) δ**: 1.38 (s, 9H), 2.86 (dd, *J* = 13.7, 8.5

Hz, 1H), 2.99 (dd, *J* = 13.7, 6.1 Hz, 1H), 3.13 – 3.22 (m, 1H), 3.24 – 3.35 (m, 1H), 3.51 – 3.67 (m, 1H), 4.33 (br s, 1H), 6.99 (d, *J* = 7.2 Hz, 2H), 7.09 – 7.22 (m, 5H), 7.94 (d, *J* = 8.2 Hz, 2H) ppm. **^{13}C-NMR (101 MHz, $CDCl_3$) δ:** 28.3, 40.3, 45.3, 48.0, 52.0, 79.4, 126.2, 128.0, 128.3, 128.7, 128.94, 129.8, 139.0, 147.5, 155.7, 166.9 ppm. **HRMS (ESI):** calc for $[C_{22}H_{27}NO_4+Na]^+$: 392.1832, found 392.1831. **HPLC**: Chiralpak IC, heptane:IPA 85:15, 0.5 mL/min, λ = 210 nm, t_R (*S*) = 24.3 min, t_R (*R*) = 27.1 min.

***tert*-Butyl (*R*)-(2-(2-methoxyphenyl)-3-phenylpropyl)carbamate, 19f**

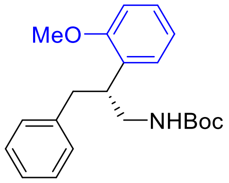

19f

Following GP6, using substrate **3f** (48.6 mg, 0.14 mmol) and catalyst (*S*$_P$)-**12** (2.2 mg, $1.4{\cdot}10^{-3}$ mmol). The reaction was stopped after 48 h and gave 100% conversion. Purification by silica column chromatography (hexanes:EtOAc 9:1) afforded **19f** as a white solid (47.2 mg, 97% yield, 96% ee). **M.p.:** 81 – 83 °C. [α]$_D$: - 4.2 (c 1.12, $CHCl_3$). **IR (ATR-FTIR) υ_{max}:** 3436, 2977, 2927, 1701, 1493, 1241, 1164 cm^{-1}. **^{1}H-NMR (400 MHz, $CDCl_3$) δ**: 1.37 (s, 9H), 2.83 – 2.99 (m, 2H), 3.28 – 3.40 (m, 1H), 3.46 – 3.59 (m, 2H), 3.76 (s, 3H), 4.39 (br s, 1H), 6.84 (d, *J* = 8.2 Hz, 1H), 6.90 (t, *J* = 7.5 Hz, 1H), 7.06 – 7.16 (m, 4H), 7.15 – 7.23 (m, 3H) ppm. **^{13}C-NMR (101 MHz, $CDCl_3$) δ:** 28.4, 39.2, 40.8, 43.8, 55.3, 78.9, 110.7, 120.7, 125.9, 127.6, 128.1, 129.0, 130.2, 134.1, 140.2, 155.9, 157.6 ppm. **HRMS (ESI):** calc for $[C_{21}H_{27}NO_3+H]^+$: 342.2064, found 342.2058. **HPLC**: Chiralpak IA, heptane:IPA 97:3, 0.5 mL/min, λ = 210 nm, t_R (*S*) = 24.3 min, t_R (*R*) = 27.1 min.

***tert*-Butyl (*R*)-(2-(2-fluorophenyl)-3-phenylpropyl)carbamate, 19g**

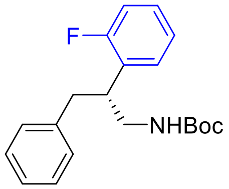

19g

Following GP6, using substrate **3g** (62.6 mg, 0.19 mmol) and catalyst (*S*$_P$)-**12** (3.0 mg, $1.9{\cdot}10^{-3}$ mmol). The reaction was stopped after 48 h and gave 100% conversion. Purification by silica column chromatography (hexanes:EtOAc 95:5) afforded **19g** as a white solid (62.0 mg, 98% yield, 96% ee).

M.p.: 78 – 80 °C. [**α**]$_D$: - 9.3 (c 1.01, $CHCl_3$). **IR (ATR-FTIR) υ$_{max}$:** 3446, 2974, 2927, 1698, 1491 cm^{-1}. **^{1}H-NMR (400 MHz, $CDCl_3$) δ**: 1.37 (s, 3H), 2.88 – 3.62 (m, 3H), 4.39 (br s, 1H), 6.95 – 7.02 (m, 1H), 7.01 – 7.09 (m, 3H), 7.11 – 7.23 (m, 5H) ppm. **^{13}C-NMR (101 MHz, $CDCl_3$) δ:** 28.5, 39.2, 41.7, 44.3, 79.3, 115.7 (d, J_F = 23.0 Hz), 124.30 (d, J = 3.6 Hz), 126.3, 128.3, 128.4, 128.9 (d, J = 14.1 Hz), 129.1, 129.4 (d, J = 5.3 Hz), 139.5, 155.9, 161.5 (d, J = 245.1 Hz) ppm. **HRMS (ESI):** calc for [$C_{20}H_{24}FNO_2$+Na]$^+$: 352.1683, found 352.1684. **HPLC**: Chiralpak IC, heptane:IPA 95:5, 0.5 mL/min, λ = 210 nm, t_R (*R*) = 16.9 min, t_R (*S*) = 19.8 min.

***tert*-Butyl (*R*)-(3-phenyl-2-(*o*-tolyl)propyl)carbamate, 19h**

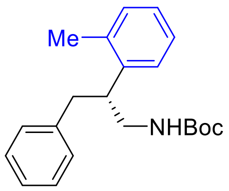

19h

Following GP6, using substrate **3h** (61.9 mg, 0.19 mmol) and catalyst (S_P)-**12** (3.0 mg, 1.9·10^{-3} mmol). The reaction was stopped after 48 h and gave 86% conversion. Purification by silica column chromatography (hexanes:EtOAc 95:5) afforded **19h** as a colourless oil (51.7 mg, 83% yield, 99% ee). [**α**]$_D$: - 7.7 (c 1.47, $CHCl_3$). **IR (ATR-FTIR) υ$_{max}$:** 3439, 2980, 2929, 1700, 1495, 1365, 1249 cm^{-1}. **^{1}H-NMR (400 MHz, $CDCl_3$) δ**: 1.38 (s, 9H), 2.10 (s, 3H), 2.82 (dd, J = 13.6, 8.0 Hz, 1H), 2.91 (dd, J = 13.5, 6.6 Hz, 1H), 3.20 – 3.29 (m, 1H), 3.36 – 3.49 (m, 1H), 3.54 – 3.67 (m, 1H), 4.34 (br s, 1H), 7.01 (d, J = 7.1 Hz, 2H), 7.05 – 7.27 (m, 7H) ppm. **^{13}C-NMR (101 MHz, $CDCl_3$) δ:** 19.7, 28.5, 40.8, 42.5, 45.2, 79.3, 125.9, 126.2, 126.4, 126.5, 128.3, 129.1, 130.6, 137.2, 139.8, 140.5, 160.0 ppm. **HRMS (ESI):** calc for [$C_{21}H_{27}NO_2$+Na]$^+$: 348.1934, found 348.1933. **HPLC**: Chiralpak IC, heptane:IPA 95:5, 0.5 mL/min, λ = 210 nm, t_R (*R*) = 14.0 min, t_R (*S*) = 18.7 min.

***tert*-Butyl (*R*)-(2-(3,5-dimethylphenyl)-3-phenylpropyl)carbamate, 19i**

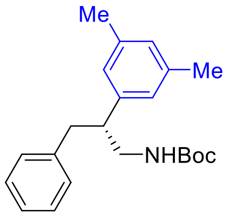

18i

Following GP6, using substrate **3i** (65.8 mg, 0.19 mmol) and catalyst (S_P)-**12** (3.0 mg, 1.9·10^{-3} mmol). The reaction was stopped after 48 h and gave 100% conversion. Purification by silica column

chromatography (hexanes:EtOAc 95:5) afforded **19i** as a white solid (55.6 mg, 84% yield, 93% ee). **M.p.:** 76 – 78 °C. $[\alpha]_D$: - 13.7 (c 0.68, $CHCl_3$). **IR (ATR-FTIR) υ_{max}:** 2980, 2914, 1701, 1496, 1351 cm^{-1}. **^{1}H-NMR (400 MHz, $CDCl_3$) δ**: 1.39 (s, 9H), 2.29 (s, 3H), 2.86 – 2.92 (m, 2H), 2.95 – 3.03 (m, 1H), 3.15 – 3.25 (m, 1H), 3.47 – 3.57 (m, 1H), 4.32 (br s, 1H), 6.77 (s, 2H), 6.86 (s, 1H), 7.07 (d, J = 7.4 Hz, 2H), 7.16 (t, J = 7.1 Hz, 1H), 7.22 (t, J = 7.3 Hz, 2H) ppm. **^{13}C-NMR (101 MHz, $CDCl_3$) δ:** 21.5, 28.5, 40.9, 45.4, 47.5, 79.2, 125.8, 126.2, 128.4, 128.6, 129.2, 138.1, 139.9, 142.3, 155.9 ppm. **HRMS (ESI):** calc for $[C_{22}H_{29}NO_2+H]^+$: 340.2271, found 340.2274. **HPLC**: Chiralpak IA, heptane:IPA 98:2, 0.5 mL/min, λ = 210 nm, t_R (R) = 13.2 min, t_R (S) = 14.5 min.

***tert*-Butyl (*R*)-(2-(3,4-dimethoxyphenyl)-3-phenylpropyl)carbamate, 19j**

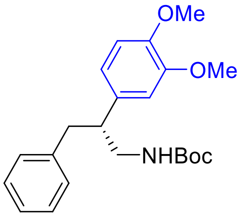

19j

Following GP6, using substrate **3j** (316.6 mg, 0.86 mmol) and catalyst (S_P)-**12** (13.4 mg, $8.6 \cdot 10^{-3}$ mmol). The reaction was stopped after 24 h and gave 100% conversion. Purification by silica column chromatography (hexanes:EtOAc 8:2) afforded **19j** as a white solid (291.6 mg, 92% yield, 98% ee). **M.p.:** 86 – 88 °C. $[\alpha]_D$: - 22.1 (c 0.84, $CHCl_3$). **IR (ATR-FTIR) υ_{max}:** 3383, 3084, 2982, 2962, 2933, 2888, 2837, 1680, 1591, 1515 cm^{-1}. **^{1}H-NMR (400 MHz, $CDCl_3$) δ**: 1.39 (s, 9H), 2.83 (dd, J = 13.4, 8.2 Hz, 1H), 2.87 – 3.04 (m, 2H), 3.18 – 3.32 (m, 1H), 3.47 – 3.62 (m, 1H), 3.80 (s, 3H), 3.85 (s, 3H), 4.37 (br s, 1H), 6.57 (d, J = 2.0 Hz, 1H), 6.67 (dd, J = 8.2, 2.0 Hz, 1H), 6.79 (d, J = 8.2 Hz, 1H), 7.01 (d, J = 7.4 Hz, 2H), 7.09 – 7.23 (m, 3H) ppm. **^{13}C-NMR (101 MHz, $CDCl_3$) δ:** 28.5, 41.0, 45.5, 47.5, 55.9, 56.0, 79.3, 111.3, 111.4, 119.9, 126.1, 128.3, 129.2, 134.6, 139.7, 147.9, 149.0, 156.0 ppm. **HRMS (ESI):** calc for $[C_{22}H_{29}NO_4\text{-}tBu]^+$: 316.1543, found 316.1543. **HPLC:** Chiralpak IC, heptane:EtOH 8:2, 0.5 mL/min, λ = 210 nm, t_R (R) = 13.9 min, t_R (S) = 17.4 min.

tert-Butyl (_R_)-(3-(2-chlorophenyl)-2-phenylpropyl)carbamate, 19k

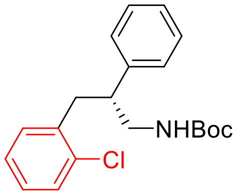

19k

Following GP6, using substrate **3k** (747.6 mg, 2.17 mmol) and catalyst (S_P)-**12** (34.1 mg, $2.2 \cdot 10^{-3}$ mmol). The reaction was stopped after 48 h and gave 100% conversion. Purification by silica column chromatography (hexanes:EtOAc 95:5) afforded **19k** as a white solid (643.3 mg, 86% yield, 99% ee). **M.p.:** 76 – 78 °C. $[\alpha]_D$: - 27.2 (c 1.02, $CHCl_3$). **IR (ATR-FTIR) υ_{max}:** 3379, 3085, 3064, 3028, 2997, 2981, 2968, 2933, 2894, 1687, 1521 cm^{-1}. **^{1}H-NMR (400 MHz, $CDCl_3$) δ**: 1.38 (s, 9H), 2.89 – 2.99 (m, 1H), 3.07 – 3.22 (m, 2H), 3.29 – 3.38 (m, 1H), 3.49 – 3.59 (m, 1H), 4.38 (br s, 1H), 6.94 (dd, J = 7.4, 1.8 Hz, 1H), 7.00 – 7.14 (m, 2H), 7.15 (d, J = 7.3 Hz, 2H), 7.19 – 7.24 (m, 1H), 7.26 – 7.33 (m, 3H) ppm. **^{13}C-NMR (101 MHz, $CDCl_3$) δ:** 28.5, 38.4, 45.2, 45.9, 79.3, 126.6, 127.0, 127.8, 128.0, 128.7, 129.6, 131.5, 134.2, 137.4, 142.0, 155.2 ppm. **HRMS (ESI):** calc for [$C_{20}H_{24}ClNO_2$-*t*Bu]$^+$: 290.0942, found 290.0941. **HPLC**: Chiralpak IA, heptane:EtOH 98:2, 0.5 mL/min, λ = 210 nm, t_R (*R*) = 14.0 min, t_R (*S*) = 16.8 min.

tert-Butyl (_R_)-(3-(2-chlorophenyl)-2-(4-fluorophenyl)propyl)carbamate, 19l

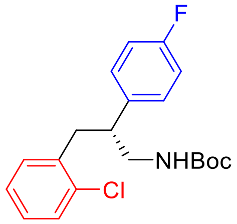

19l

Following GP6, using substrate **3l** (434.2 mg, 1.20 mmol) and catalyst (S_P)-**12** (18.8 mg, 0.012 mmol). The reaction was stopped after 48 h and gave 100% conversion. Purification by silica column chromatography (hexanes:EtOAc 9:1) afforded **19l** as a white solid (362.4 mg, 83% yield, 95% ee). **M.p.:** 62 – 63 °C. $[\alpha]_D$: - 35.7 (c 0.92, $CHCl_3$). **IR (ATR-FTIR) υ_{max}:** 3361, 2968, 2927, 1685, 1536, 1510 cm^{-1}. **^{1}H-NMR (400 MHz, $CDCl_3$) δ**: 1.39 (s, 9H), 2.87 (dd, J = 12.9, 7.5 Hz, 1H), 3.09 – 3.21 (m, 2H), 3.26 – 3.37 (m, 1H), 3.47 – 3.56 (m, 1H), 4.40 (br s, 1H), 6.91 (d, J = 7.5 Hz, 1H), 6.96 (t, J = 8.7 Hz, 2H), 7.00 – 7.14 (m, 3H), 7.31 (d, J = 7.7 Hz, 1H) ppm. **^{13}C-NMR (101 MHz, $CDCl_3$) δ:** 28.5, 38.5, 45.3, 45.4, 79.4, 115.5 (d, J_F = 21.2 Hz), 126.7, 127.8, 129.4 (d, J_F = 7.8 Hz), 129.7,

131.4, 134.2, 137.2, 137.6 (d, J_F = 3.1 Hz), 155.9, 161.9 (d, J_F = 244.8 Hz) ppm. **HRMS (ESI):** calc for $[C_{20}H_{23}ClFNO_2$-*t*Bu$]^+$: 308.0848, found 308.0856. **HPLC**: Chiralpak IA, heptane:IPA 98:2, 0.5 mL/min, λ = 210 nm, t_R (*R*) = 19.0 min, t_R (*S*) = 21.4 min.

***tert*-Butyl (*R*)-(3-(2-chlorophenyl)-2-(2-methoxyphenyl)propyl)carbamate, 19m**

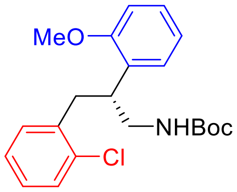

19m

Following GP6, using substrate **3m** (333.8 mg, 0.89 mmol) and catalyst (S_P)-**12** (14.0 mg, $8.9{\cdot}10^{-3}$ mmol). The reaction was stopped after 48 h and gave 100% conversion. Purification by silica column chromatography (hexanes:EtOAc 9:1) afforded **19m** as a colourless oil (264.1 mg, 79% yield, 94% ee). $[\alpha]_D$: - 18.5 (c 1.25, $CHCl_3$). **IR (ATR-FTIR) υ_{max}:** 3440, 3059, 2974, 2931, 2837, 1699, 1492, 1474 cm^{-1}. **^{1}H-NMR (400 MHz, $CDCl_3$) δ**: 1.38 (s, 9H), 2.97 (dd, *J* = 13.6, 7.8 Hz, 1H), 3.14 (dd, *J* = 13.7, 7.0 Hz, 1H), 3.34 – 3.45 (m, 1H), 3.48 – 3.57 (m, 1H), 3.63 (qu, *J* = 7.2 Hz, 1H), 3.71 (s, 3H), 4.45 (br s, 1H), 6.81 (d, *J* = 8.2 Hz, 1H), 6.90 (t, *J* = 7.4 Hz, 1H), 6.94 – 7.11 (m, 3H), 7.11 – 7.23 (m, 2H), 7.30 (d, *J* = 7.6 Hz, 1H) ppm. **^{13}C-NMR (101 MHz, $CDCl_3$) δ:** 28.5, 36.7, 39.7, 44.0, 55.4, 79.1, 110.9, 120.9, 126.5, 127.5, 127.8, 128.5, 129.4, 130.1, 131.3, 134.4, 138.0, 156.0, 157.8 ppm. **HRMS (ESI):** calc for $[C_{21}H_{26}ClNO_3$-Boc$]^+$: 276.1150, found 276.1156. **HPLC**: Chiralpak IA, heptane:IPA 95:5, 0.5 mL/min, λ = 210 nm, t_R (*R*) = 12.8 min, t_R (*S*) = 14.9 min.

(*R*)-*N*-(2-(4-Methoxyphenyl)-3-phenylpropyl)-4-methylbenzenesulfonamide, 19n

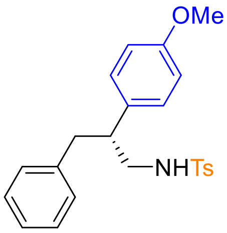

19n

Following GP6, using substrate **3n** (54.2 mg, 0.14 mmol) and catalyst (S_P)-**12** (2.2 mg, $1.4{\cdot}10^{-3}$ mmol). The reaction was stopped after 48 h and gave 100% conversion. Purification by silica column chromatography (hexanes:EtOAc 8:2) afforded **19n** as a white solid (37.3 mg, 69% yield, 97% ee). **M.p.:** 132 – 135 °C. $[\alpha]_D$: - 35.2 (c 1.01, $CHCl_3$). **IR (ATR-FTIR) υ_{max}:** 3243, 2936, 1739, 1606,

1510, 1311, 1246 cm^{-1}. **^{1}H-NMR (400 MHz, $CDCl_3$) δ**: 2.44 (s, 3H), 2.74 – 2.94 (m, 3H), 2.95 – 3.11 (m, 1H), 3.21 – 3.33 (m, 1H), 3.78 (s, 3H), 4.08 (br s, 1H), 6.78 (d, *J* = 8.6 Hz, 2H), 6.86 – 6.98 (m, 4H), 7.11 – 7.24 (m, 3H), 7.27 (d, *J* = 7.5 Hz, 2H), 7.59 (d, *J* = 8.3 Hz, 2H) ppm. **^{13}C-NMR (101 MHz, $CDCl_3$) δ:** 21.4, 40.3, 46.3, 47.4, 55.1, 114.1, 126.0, 126.9, 128.1, 128.5, 128.8, 129.5, 132.5, 136.6, 138.8, 143.2, 158.5 ppm. **HRMS (ESI):** calc for $[C_{23}H_{25}NO_3S+H]^+$: 396.1628, found 396.1625. **HPLC**: Chiralpak IA, heptane:IPA 9:1, 0.5 mL/min, λ = 210 nm, t_R (*R*) = 35.1 min, t_R (*S*) = 38.2 min.

(*R*)-*N*-(2-(4-Chlorophenyl)-3-phenylpropyl)-4-methylbenzenesulfonamide, 19o

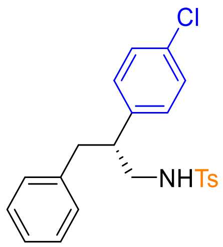

19o

Following GP6, using substrate **3o** (43.5 mg, 0.11 mmol) and catalyst (S_P)-**12** (1.7 mg, 1.1·10^{-3} mmol). The reaction was stopped after 48 h and gave 100% conversion. Purification by silica column chromatography (hexanes:EtOAc 8:2) afforded **19o** as a white solid (40.7 mg, 93% yield, 95% ee). **M.p.:** 146 – 147 °C. [**α**]$_D$: - 43.3 (c 1.08, $CHCl_3$). **^{1}H-NMR (400 MHz, $CDCl_3$) δ**: 2.37 (s, 3H), 2.69 (dd, *J* = 13.4, 7.7 Hz, 1H), 2.76 – 2.93 (m, 2H), 2.93 – 3.02 (m, 1H), 3.11 – 3.26 (m, 1H), 4.02 – 4.12 (m, 1H), 6.78 – 6.96 (m, 4H), 7.06 – 7.15 (m, 4H), 7.16 – 7.23 (m, 3H), 7.44 – 7.60 (m, 2H) ppm. **^{13}C-NMR (101 MHz, $CDCl_3$) δ:** 21.5, 40.1, 46.9, 47.5, 126.4, 127.1, 128.4, 128.9, 129.0, 129.1, 129.7, 132.9, 136.7, 138.5, 139.3, 143.5 ppm. The NMR data for this compound agreed with that quoted in the literature.[13] **HPLC**: Chiralpak IA, heptane:IPA 9:1, 0.5 mL/min, λ = 210 nm, t_R (*R*) = 29.1 min, t_R (*S*) = 31.5 min.

(*R*)-4-Methyl-*N*-(3-phenyl-2-(*o*-tolyl)propyl)benzenesulfonamide, 19p

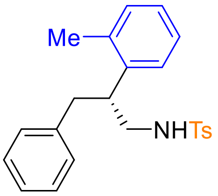

19p

Following GP6, using substrate **3p** (59.4 mg, 0.16 mmol) and catalyst (S_P)-**12** (2.5 mg, $1.6{\cdot}10^{-3}$ mmol). The reaction was stopped after 48 h and gave 100% conversion. Purification by silica column chromatography (hexanes:EtOAc 85:15) afforded **19p** as a white solid (55.5 mg, 93% yield, 98% ee). **M.p.:** 86 – 88 °C. $[\alpha]_D$: - 37.1 (c 1.03, $CHCl_3$). **^{1}H-NMR (400 MHz, $CDCl_3$) δ:** 1.93 (s, 3H), 2.35 (s, 3H), 2.67 (dd, *J* = 13.5, 7.3 Hz, 1H), 2.76 (dd, *J* = 13.5, 6.9 Hz, 1H), 2.95 – 3.07 (m, 1H), 3.14 – 3.32 (m, 2H), 4.07 – 4.19 (m, 1H), 6.85 (d, *J* = 5.6 Hz, 2H), 6.91 – 7.14 (m, 7H), 7.18 (d, *J* = 7.7 Hz, 2H), 7.52 (d, *J* = 8.0 Hz, 2H) ppm. **^{13}C-NMR (101 MHz, $CDCl_3$) δ:** 19.1, 21.2, 40.1, 41.7, 46.5, 125.1, 125.9, 126.2, 126.4, 126.7, 127.9, 128.6, 129.3, 130.4, 136.3, 136.7, 138.7, 138.7, 143.0 ppm. The NMR data for this compound agreed with that quoted in the literature.[13] **HPLC**: Chiralpak IA, heptane:IPA 7:3, 0.5 mL/min, λ = 210 nm, t_R (*R*) = 11.0 min, t_R (*S*) = 12.6 min.

2.4 Synthesis of 3-aryl-tetrahydroquinolines 21k-m

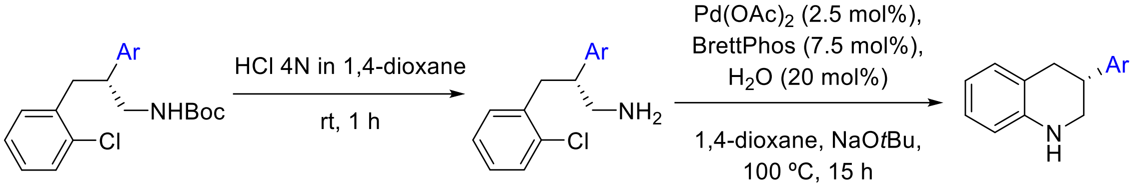

General procedure for the preparation of N-unprotected 2,3-diarylpropyl amines (GP7):

The corresponding *N*-Boc-2,3-diarylpropyl amine **19** was dissolved in a solution of HCl 4N in 1,4-dioxane (0.24M) and stirred at room temperature for 1 h. After that time, the solvent was evaporated, the crude was dissolved in DCM and NaOH 1M was added. The organic layer was separated and the aqueous phase was extracted thrice with DCM. The combined extracts were dried over $MgSO_4$, filtered and concentrated under vacuum, affording the N-unprotected intermediate **20** without need of further purification.

Synthesis of (*R*)-3-(2-chlorophenyl)-2-phenylpropan-1-amine, 20k

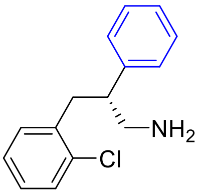

20k

Following GP7, using **19k** (528.2 mg, 1.53 mmol) and HCl 4N in dioxane (6.4 mL) afforded **20k** as a yellow oil without need of further purification (381.1 mg, quantitative yield). $[\alpha]_D$: - 57.9 (c 1.32, $CHCl_3$). **IR (ATR-FTIR) υ_{max}:** 3368, 3060, 3027, 2924, 2856, 1601, 1570, 1473, 1443 cm^{-1}. **^{1}H-NMR (400 MHz, $CDCl_3$) δ**: 2.92 (dd, *J* = 13.1, 6.3 Hz, 1H), 2.96 – 3.07 (m, 2H), 3.13 (dd, *J* = 13.1, 5.8 Hz, 1H), 6.95 (dd, *J* = 7.5, 1.8 Hz, 1H), 7.05 (td, *J* = 7.4, 1.6 Hz, 1H), 7.09 (td, *J* = 7.6, 1.9 Hz, 1H), 7.16 – 7.24 (m, 3H), 7.27 – 7.34 (m, 3H) ppm. **^{13}C-NMR (101 MHz, $CDCl_3$) δ:** 38.6, 46.8, 49.8, 126.5, 126.8, 127.6, 128.1, 128.7, 129.6, 131.4, 134.2, 137.9, 142.7 ppm**. HRMS (ESI):** calc for $[C_{15}H_{16}ClN+H]^+$: 246.1044, found 246.1042.

Synthesis of (*R*)-3-(2-chlorophenyl)-2-(4-fluorophenyl)propan-1-amine, 20l

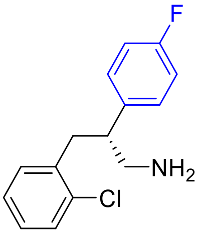

20l

Following GP7, using **19l** (335.7 mg, 0.92 mmol) and HCl 4N in dioxane (3.8 mL) afforded **20l** as a yellow oil without need of further purification (233.9 mg, 96% yield). $[\alpha]_D$: - 71.4 (c 1.25, $CHCl_3$). **IR (ATR-FTIR) υ_{max}:** 3376, 3060, 2923, 2856, 1603, 1508, 1474, 1442 cm^{-1}. **^{1}H-NMR (400 MHz, $CDCl_3$) δ**: 2.86 (dd, *J* = 13.1, 7.2 Hz, 1H), 2.93 – 3.05 (m, 3H), 3.12 (dd, *J* = 13.1, 6.2 Hz, 1H), 6.91 (dd, *J* = 7.4, 1.9 Hz, 1H), 6.93 – 7.02 (m, 2H), 7.01 – 7.16 (m, 4H), 7.31 (dd, *J* = 7.8, 1.5 Hz, 1H) ppm. **^{13}C-NMR (101 MHz, $CDCl_3$) δ:** 38.7, 47.1, 49.0, 115.5 (d, J_F = 21.0 Hz), 126.6, 127.7, 129.5 (d, J_F = 7.8 Hz), 129.6, 131.4, 134.2, 137.7, 138.4 (d, J_F = 3.5 Hz), 161.8 (d, J_F = 244.3 Hz) ppm. **HRMS (ESI):** calc for $[C_{15}H_{15}ClFN+H]^+$: 264.0950, found 264.0949.

Synthesis of (*R*)-3-(2-chlorophenyl)-2-(2-methoxyphenyl)propan-1-amine, 20m

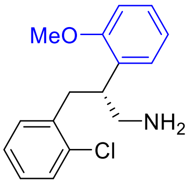

20m

Following GP7, using **19m** (286.4 mg, 0.76 mmol) and HCl 4N in dioxane (3.2 mL) afforded **20m** as a yellow oil without need of further purification (193.2 mg, 92% yield). $[\alpha]_D$: - 33.1 (c 1.11,

$CHCl_3$). **IR (ATR-FTIR) υ_{max}:** 3376, 3061, 2933, 2855, 2834, 1598, 1584, 1490, 1462, 1438 cm^{-1}. **^{1}H-NMR (400 MHz, $CDCl_3$) δ**: 1.22 (br s, 2H), 2.86 – 3.07 (m, 3H), 3.15 (dd, *J* = 13.6, 7.2 Hz, 1H), 3.47 – 3.60 (m, 1H), 3.69 (s, 3H), 6.81 (d, *J* = 8.5 Hz, 1H), 6.92 (td, *J* = 7.4, 1.2 Hz, 1H), 6.97 (dd, *J* = 7.3, 2.2 Hz, 1H), 7.05 (qud, *J* = 7.3, 1.8 Hz, 2H), 7.13 – 7.21 (m, 2H), 7.30 (dd, *J* = 7.6, 1.7 Hz, 1H) ppm. **^{13}C-NMR (101 MHz, $CDCl_3$) δ:** 37.0, 42.8, 45.7, 55.4, 110.9, 120.8, 126.4, 127.3, 127.5, 128.2, 129.3, 130.8, 131.2, 134.4, 138.4, 158.0 ppm. **HRMS (ESI):** calc for $[C_{16}H_{18}ClNO+H]^+$: 276.1150, found 276.1150.

General procedure for the preparation of 3-aryl-tetrahydroquinolines 21 (GP8):[14]

An oven-dried vial was charged with $Pd(OAc)_2$ (2.5 mol%) and BrettPhos (7.5 mol%). The vial was sealed and purged with vacuum-nitrogen cycles. Then, 1,4-dioxane (0.2M) and degassed H_2O (20 mol%) were sequentially added and the resulting solution was heated in an oil bath at 100 °C for 1 min. This solution was transferred via syringe to an oven-dried Schlenk tube containing **20k-m** (1 eq.) and NaO*t*Bu (1.2 eq.). The reaction was heated in an oil bath at 100 °C for 15 h. After that time, the reaction was cooled down to room temperature, diluted with EtOAc, and washed once with H_2O. The organic phase was concentrated and the crude was purified by silica column chromatography using hexanes:EtOAc as eluent, affording the desired product.

Synthesis of (+)-(*R*)-3-phenyl-1,2,3,4-tetrahydroquinoline, 21k

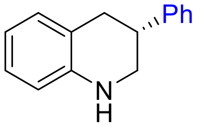

21k

Following GP8, $Pd(OAc)_2$ (7.4 mg, 0.033 mmol), BrettPhos (55.3 mg, 0.10 mmol), 1,4-dioxane (6.6 mL), degassed H_2O (5 μL, 0.26 mmol), **20k** (323.4 mg, 1.32 mmol) and NaO*t*Bu (156.9 mg, 1.58 mmol) were used. Purification by silica column chromatography (hexanes:EtOAc 97:3) afforded **21k** as a white solid (199.5 mg, 72% yield). $[\alpha]_D$: + 21.8 (c 0.74, $CHCl_3$). **^{1}H-NMR (400 MHz, $CDCl_3$) δ**: 2.92 – 3.08 (m, 2H), 3.10 – 3.21 (m, 1H), 3.34 (t, *J* = 10.7 Hz, 1H), 3.47 (ddd, *J* = 11.1, 3.9, 1.9 Hz, 1H), 4.04 (br s, 1H), 6.56 (d, *J* = 7.5 Hz, 1H), 6.65 (t, *J* = 7.4 Hz, 1H), 7.02 (t, *J* = 7.1 Hz, 2H), 7.23 – 7.28 (m, 3H), 7.30 – 7.39 (m, 2H) ppm. **^{13}C-NMR (101 MHz, $CDCl_3$) δ:** 35.27, 37.74, 48.04, 114.18, 117.24, 121.48, 126.79, 127.11, 127.35, 128.74, 129.67, 143.99, 144.17 ppm. The analytical data for this compound agreed with that quoted in the literature.[15]

Synthesis of (+)-(*R*)-3-(4-fluorophenyl)-1,2,3,4-tetrahydroquinoline, 21l

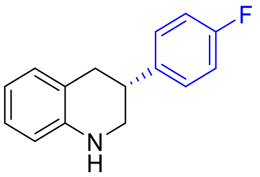

21l

Following GP8, Pd(OAc)$_2$ (4.3 mg, 0.019 mmol), BrettPhos (31.5 mg, 0.056 mmol), 1,4-dioxane (3.8 mL), degassed H_2O (3 µL, 0.17 mmol), **20l** (197.8 mg, 0.75 mmol) and NaO*t*Bu (89.1 mg, 0.90 mmol) were used. Purification by silica column chromatography (cyclohexane:EtOAc 9:1) afforded **21l** as a white solid (145.7 mg, 85% yield). [**α**]$_D$: + 18.7 (c 1.07, $CHCl_3$). **^{1}H-NMR (400 MHz, $CDCl_3$) δ**: 2.97 (d, *J* = 8.0 Hz, 2H), 3.08 – 3.20 (m, 1H), 3.30 (t, *J* = 10.6 Hz, 1H), 3.44 (dd, *J* = 10.8, 3.2 Hz, 1H), 4.02 (br s, 1H), 6.55 (d, *J* = 7.7 Hz, 1H), 6.65 (td, *J* = 7.4, 1.2 Hz, 1H), 6.94 – 7.08 (m, 4H), 7.14 – 7.23 (m, 2H) ppm. **^{13}C-NMR (101 MHz, $CDCl_3$) δ:** 34.8, 38.1, 48.5, 114.2, 115.5 (d, J_F = 21.1 Hz), 117.3, 121.2, 127.2, 128.7 (d, J_F = 7.9 Hz), 129.7, 139.7 (d, J_F = 3.3 Hz), 144.1, 161.8 (d, J_F = 244.5 Hz) ppm. The analytical data for this compound agreed with that quoted in the literature.[16]

Synthesis of (+)-(*R*)-3-(2-methoxyphenyl)-1,2,3,4-tetrahydroquinoline, 21m

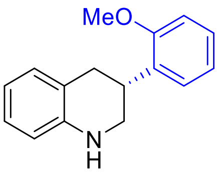

21m

Following GP8, Pd(OAc)$_2$ (3.2 mg, 0.014 mmol), BrettPhos (24.3 mg, 0.043 mmol), 1,4-dioxane (2.9 mL), degassed H_2O (2 µL, 0.11 mmol), **20m** (159.6 mg, 0.58 mmol) and NaO*t*Bu (68.9 mg, 0.70 mmol) were used. Purification by silica column chromatography (cyclohexane:EtOAc 95:5) afforded **21m** as a white solid (114.2 mg, 82% yield). **M.p.:** 60 – 62 °C. [**α**]$_D$: + 3.6 (c 1.02, $CHCl_3$). **IR (ATR-FTIR) υ_{max}:** 3416, 3013, 2923, 2837, 1603, 1581, 1491, 1461, 1436 cm^{-1}. **^{1}H-NMR (400 MHz, $CDCl_3$) δ**: 2.90 – 3.09 (m, 2H), 3.32 (t, *J* = 10.6 Hz, 1H), 3.41 – 3.49 (m, 1H), 3.54 – 3.67 (m, 1H), 3.83 (s, 3H), 3.99 (br s, 1H), 6.55 (dd, *J* = 8.3, 1.2 Hz, 1H), 6.64 (td, *J* = 7.3, 1.2 Hz, 1H), 6.90 (d, *J* = 8.2 Hz, 1H), 6.94 (td, *J* = 7.5, 1.1 Hz, 1H), 7.01 (d, *J* = 7.2 Hz, 2H), 7.15 (dd, *J* = 7.2, 1.8 Hz, 1H), 7.23 (td, *J* = 7.7, 1.7 Hz, 1H) ppm. **^{13}C-NMR (101 MHz, $CDCl_3$) δ:** 31.5, 33.3, 47.2, 55.5, 110.5, 114.2, 117.1, 120.8, 121.9, 126.9, 127.0, 127.5, 129.7, 132.1, 144.3, 157.2 ppm. **HRMS (ESI):** calc for $[C_{16}H_{17}NO+H]^+$: 240.1383, found 240.1383.

2.5 Synthesis of 4-benzyl-tetrahydroisoquinoline 23

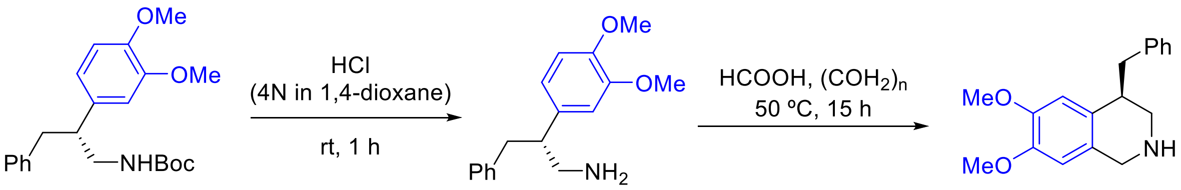

19j 22 23

Synthesis of (*R*)-2-(3,4-dimethoxyphenyl)-3-phenylpropan-1-amine, 22

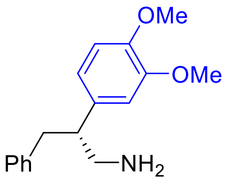

22

Following GP7, using **19j** (328.8 mg, 0.89 mmol) and HCl 4N in dioxane (3.7 mL) afforded **22** as a yellow oil without need of further purification (216.2 mg, 90% yield). $[\alpha]_D$: - 66.4 (c 0.92, $CHCl_3$). **IR (ATR-FTIR)** υ_{max}**:** 3375, 3058, 3023, 2999, 2926, 2833, 1602, 1590, 1514, 1462, 1452 cm^{-1}. **^{1}H-NMR (400 MHz, $CDCl_3$) δ:** 1.22 (br s, 2H), 2.78 – 3.00 (m, 5H), 3.82 (s, 3H), 3.85 (s, 3H), 6.62 (s, 1H), 6.71 (d, *J* = 8.2 Hz, 1H), 6.80 (d, *J* = 8.1 Hz, 1H), 7.04 (d, *J* = 7.4 Hz, 2H), 7.11 – 7.23 (m, 3H) ppm. **^{13}C-NMR (101 MHz, $CDCl_3$) δ:** 41.1, 47.1, 51.2, 55.9, 56.0, 111.3, 120.0, 126.0, 128.3, 129.2, 135.5, 140.3, 147.7, 149.0 ppm. **HRMS (ESI):** calc for $[C_{17}H_{21}NO_2+H]^+$: 272.1645, found 272.1648.

Synthesis of (*R*)-4-benzyl-6,7-dimethoxy-1,2,3,4-tetrahydroisoquinoline, 23

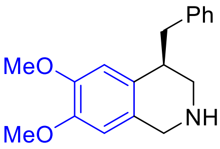

23

A vial was charged with **22** (108.1 mg, 0.40 mmol), cooled down to 0 °C and formic acid (0.22 mL) was added. After stirring at 0 °C for 10 min, paraformaldehyde (12.0 mg, 0.40 mmol) was added. The reaction mixture was then stirred at 50 °C for 15 h. Afterwards, excess formic acid was evaporated under reduced pressure, and ice-water was poured into the residue. The mixture was then basified to pH 11 with NaOH 1M and extracted with DCM (x3). The combined organic layers were dried over $MgSO_4$ and concentrated under vacuum. The crude was purified by silica column chromatography (DCM:MeOH 9:1), obtaining **23** as a colourless oil (76.9 mg, 68% yield). $[\alpha]_D$: - 58.8 (c 1.16,

$CHCl_3$). **IR (ATR-FTIR) υ_{max}:** 3337, 3056, 3023, 2997, 2930, 2832, 1608, 1511, 1463, 1452 cm^{-1}. **^{1}H-NMR (400 MHz, $CDCl_3$) δ:** 2.14 (br s, 1H), 2.83 – 3.08 (m, 5H), 3.72 (s, 5H), 3.84 (s, 5H), 3.94 (s, 2H), 6.45 (s, 1H), 6.51 (s, 1H), 7.20 (dd, *J* = 17.2, 7.1 Hz, 3H), 7.30 (t, *J* = 7.4 Hz, 2H) ppm. **^{13}C-NMR (101 MHz, $CDCl_3$) δ:** 38.8, 42.4, 47.7, 48.3, 55.9, 56.0, 108.8, 112.1, 126.2, 127.8, 128.5, 129.5, 130.1, 140.5, 147.2, 147.5 ppm. **HRMS (ESI):** calc for $[C_{18}H_{21}NO_2+H]^+$: 284.1645, found 284.1647.

2.6 References

1 A. Cabré, H. Khaizourane, M. Garçon, X. Verdaguer, A. Riera, *Org. Lett.* **2018**, *20*, 3953 – 3957.

2 S. Kim-Lee, I. Alonso, P. Mauleón, R. Arrayás, J. Carretero, *ACS Catal.* **2018**, *8*, 8993 – 9005.

3 J. Xie, Z. Guo, Y. Huang, Y. Qu, H. Song, H. Song, Y. Liu, Q. Wang, *Adv. Synth. Catal.* **2019**, *361*, 490 – 495.

4 K. M. Miller, C. Molinaro, T. F. Jamison, *Tetrahedron: Asymmetry* **2003**, *14*, 3619 – 3625.

5 L. Zhang, J. Panteleev, M. Lautens, *J. Org. Chem.* **2014**, *79*, 12159 – 12176.

6 C. Nieto-Oberhuber, P. Pérez-Galán, E. Herrero-Gómez, T. Lauterbach, D. J. Cárdenas, A. M. Echavarren, *J. Am. Chem. Soc.* **2008**, *130*, 269 – 279.

7 A. L. Moure, R. G. Arrayás, D. J. Cárdenas, I. Alonso, J. C. Carretero, *J. Am. Chem. Soc.* **2012**, *134*, 7219 – 7222.

8 S. Mannathan, M. Jeganmohan, C.-H. Cheng, *Angew. Chem. Int. Ed.* **2009**, *48*, 2192 – 2195.

9 S. Orgué, A. Flores-Gaspar, M. Biosca, O. Pàmies, M. Diéguez, A. Riera, X. Verdaguer, *Chem. Commun.* **2015**, *51*, 17548 – 17551.

10 T. León, A. Riera, X. Verdaguer, *J. Am. Chem. Soc.* **2011**, *133*, 5740 – 5743.

11 E. Salomó, A. Prades, A. Riera, X. Verdaguer, *J. Org. Chem.* **2017**, *82*, 7065 – 7069.

12 E. Salomó, P. Rojo, P. Hernández-Lladó, A. Riera, X. Verdaguer, *J. Org. Chem.* **2018**, *83*, 4618 – 4627.

13 X. Yu, Q.-Q. Zhou, P.-Z. Wang, C.-M. Liao, J.-R. Chen, W.-J. Xiao, *Org. Lett.* **2018**, *20*, 421 – 424.

14 The procedure was adapted from: B. P. Fors, D. A. Watson, M. R. Biscoe, S. L. Buchwald, *J. Am. Chem. Soc.* **2008**, *130*, 13552 – 13554.

15 M. Rueping, T. Theissmann, S. Raja, J. W. Bats, *Adv. Synth. Catal.* **2008**, *350*, 1001 – 1006.

16 X. Zhao, J. Xiao, W. Tang, *Synthesis* **2017**, *49*, 3157 – 3164.

3. Crystallographic information

X-ray of (S_P)-12:

The single crystal suited for X-ray diffraction was grown by layering recrystallization of (S_P)-**12** in an hexanes/DCM solvent system at 4 °C. The BArF counterion has been omitted for clarity. CCDC No. 2157242.

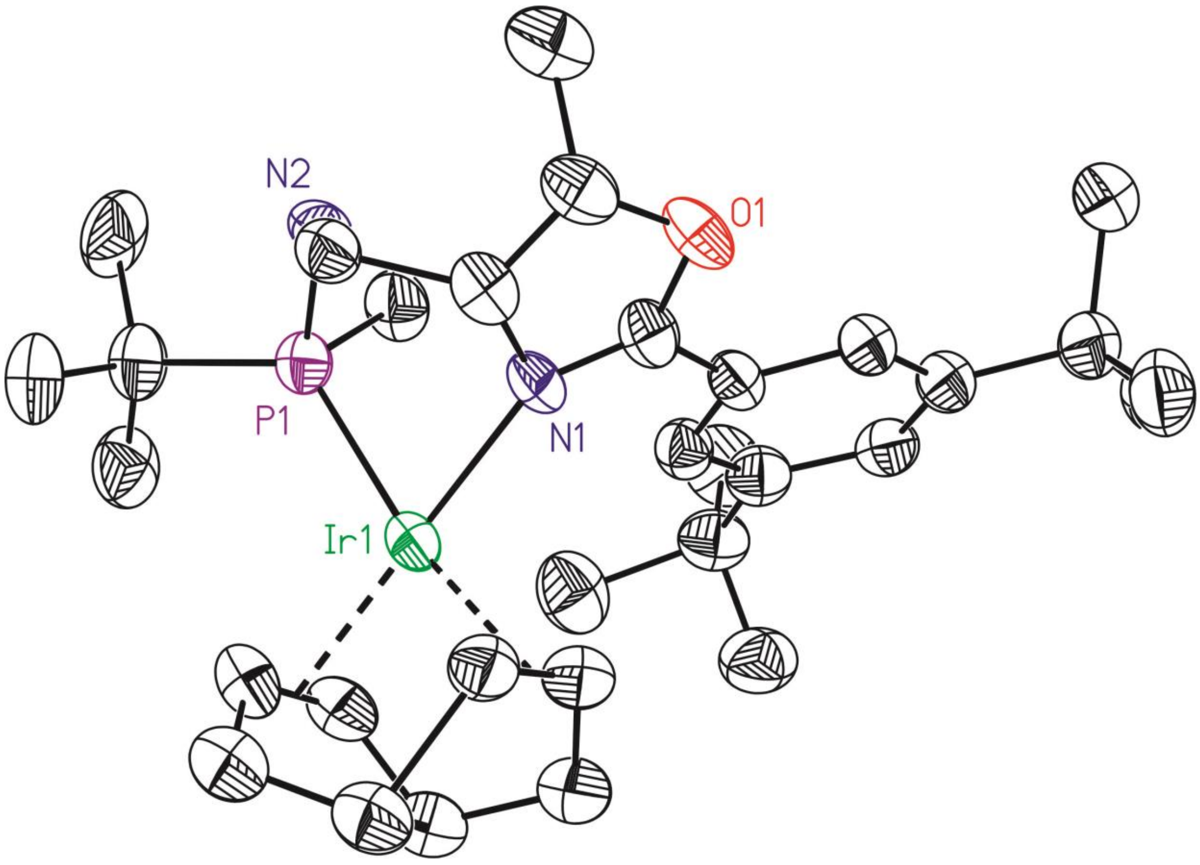

Figure S1: X-ray of (S_P)-**12**. ORTEP diagram shows thermal ellipsoids at 50% probability.

Table S1: Crystal data of (S_P)-**12**.

Empirical formula	C64 H65 B F24 Ir N2 O P
Formula weight	1568.16
Temperature	100(2)K
Wavelength	0.71073 Å
Crystal system	triclinic
Space group	P 1
Unit cell dimensions	a = 9.36972(11)Å α = 96.9834(11)°.
	b = 13.39240(18)Å β = 102.7926(11)°.
	c = 13.8805(2)Å γ = 100.6079(11)°.
Volume	1645.38(4) $Å^3$
Z	1
Density (calculated)	1.583 Mg/m^3
Absorption coefficient	2.164 mm^{-1}
F(000)	784
Crystal size	0.090 x 0.090 x 0.010 mm^3
Theta range for data collection	2.284 to 34.457°.
Index ranges	-14<=h<=14,-21<=k<=21,-22<=l<=21
Reflections collected	90329
Independent reflections	26243[R(int) = 0.0406]
Completeness to theta =34.457°	96.4%
Absorption correction	Multi-scan
Max. and min. transmission	1.00 and 0.81
Refinement method	Full-matrix least-squares on F^2
Data / restraints / parameters	26243/ 4136/ 1556
Goodness-of-fit on F^2	0.995
Final R indices [I>2sigma(I)]	R1 = 0.0541, wR2 = 0.1306
R indices (all data)	R1 = 0.0681, wR2 = 0.1382
Largest diff. peak and hole	3.257 and -1.259 e.$Å^{-3}$

X-ray of (R_P)-12:

The single crystal suited for X-ray diffraction was grown by layering recrystallization of (R_P)-**12** in an hexanes/DCM solvent system at 4 °C. CCDC No. 2157243.

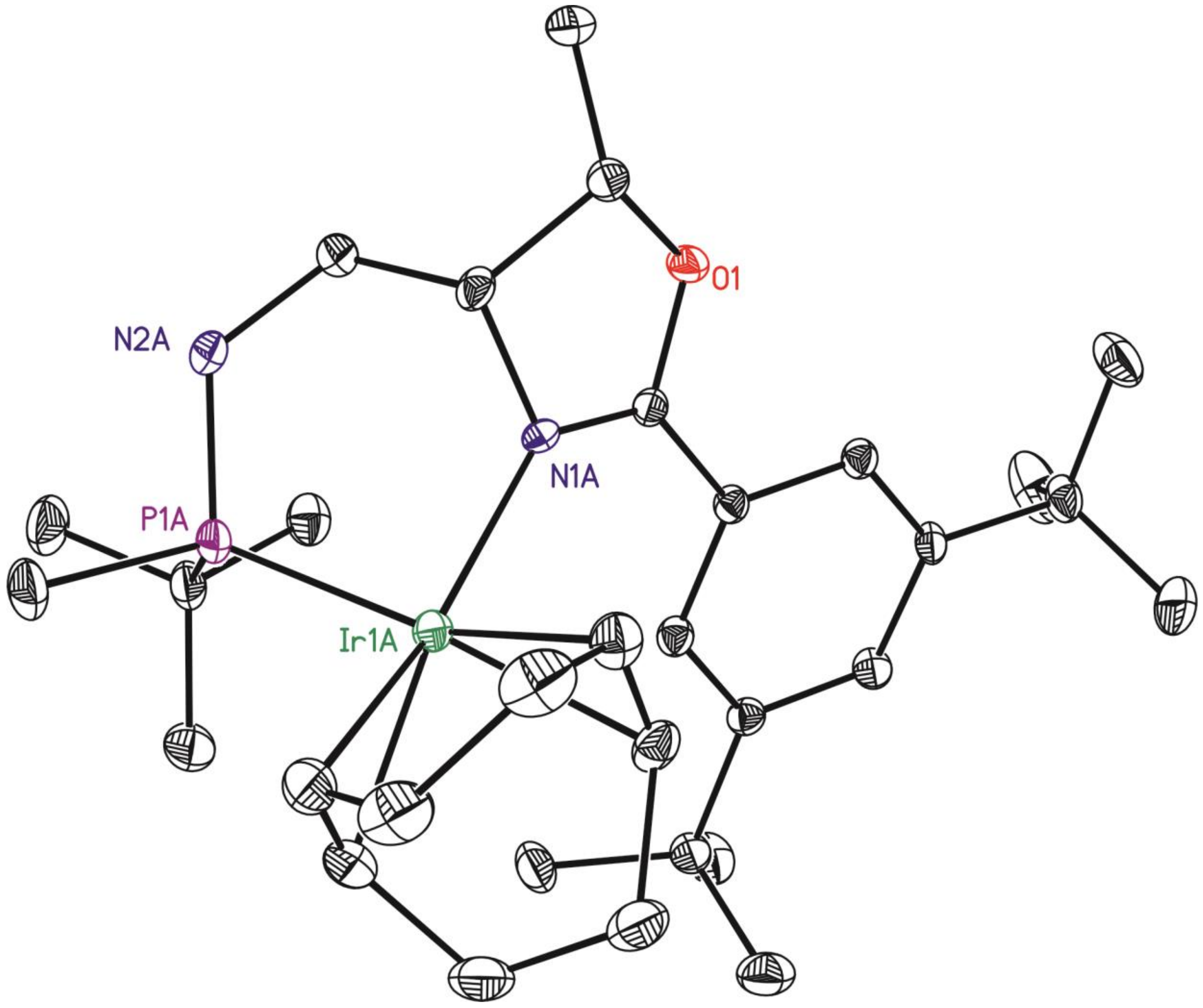

Figure S2: X-ray of (R_P)-**12**, representing one of the two twins obtained by diffraction. ORTEP diagram shows thermal ellipsoids at 50% probability.

Table S2: Crystal data of (R_P)-**12**.

Empirical formula	C128 H130 B2 F48 Ir2 N4 O2 P2	
Formula weight	3136.31	
Temperature	100(2)K	
Wavelength	0.71073 Å	
Crystal system	triclinic	
Space group	P 1	
Unit cell dimensions	a = 13.0950(8)Å	α = 103.0150(10)°.
	b = 13.8097(8)Å	β = 99.0890(10)°.
	c = 18.7211(11)Å	γ = 94.5280(10)°.
Volume	3233.9(3) $Å^3$	
Z	1	
Density (calculated)	1.610 Mg/m^3	
Absorption coefficient	2.202 mm^{-1}	
F(000)	1568	
Crystal size	0.100 x 0.100 x 0.040 mm^3	
Theta range for data collection	1.524 to 32.959°.	
Index ranges	-19<=h<=19,-20<=k<=21,-28<=l<=28	
Reflections collected	118106	
Independent reflections	118106[R(int) = ?]	
Completeness to theta =32.959°	94.0%	
Absorption correction	Multi-scan	
Max. and min. transmission	0.74 and 0.45	
Refinement method	Full-matrix least-squares on F^2	
Data / restraints / parameters	118106/ 69/ 1791	
Goodness-of-fit on F^2	1.072	
Final R indices [I>2sigma(I)]	R1 = 0.0273, wR2 = 0.0746	
R indices (all data)	R1 = 0.0283, wR2 = 0.0753	
Flack parameter	x =-0.007(2)	
Largest diff. peak and hole	2.138 and -0.722 e.$Å^{-3}$	

4. <u>Stereochemical course of the asymmetric hydrogenation.</u>

Asymmetric hydrogenation of allylic amine takes place in accordance with the Andersson quadrant model. Coordination of the olefin towards an $[IrH_2(N,P)(Solvent)_2]^+$ complex takes place in the same equatorial plane as the chiral bidentate N,P-ligand *cis* to nitrogen and *trans* to phosphorus. The resultant quadrant model rationalizes whether the steric bulk of the ligand is above or below the N-Ir-P plane. To minimize steric interactions, the substrate then preferentially coordinates with the smallest olefin substituent, in this case the vinylic proton, to the hindered environment and thus coordinates preferentially by one of the enantiotopic faces., in this case the *pro-R* face.

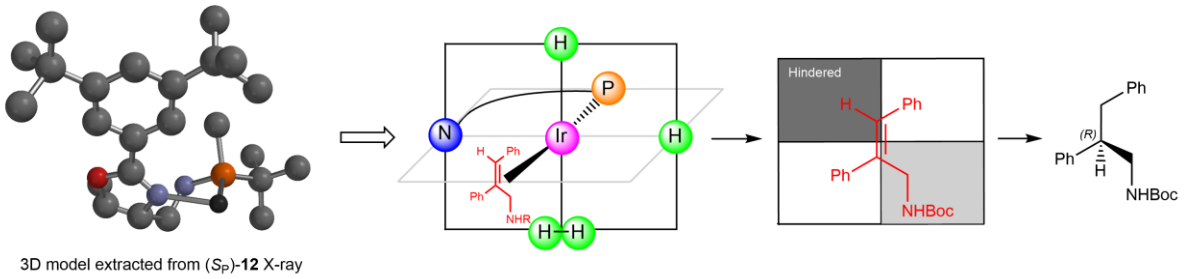

See references:

1. C. Hedberg, K. Källström, P. Brandt, L. K. Hansen, P. G. Andersson, *J. Am. Chem. Soc.* **2006**, *128*, 2995–3001.
2. T. L. Church, T. Rasmussen, P. G. Andersson, *Organometallics* **2010**, *29*, 6769–6781.

5. ^{1}H-NMR, ^{13}C-NMR and ^{31}P-NMR spectra

^{1}H-NMR (400 MHz, $CDCl_3$):

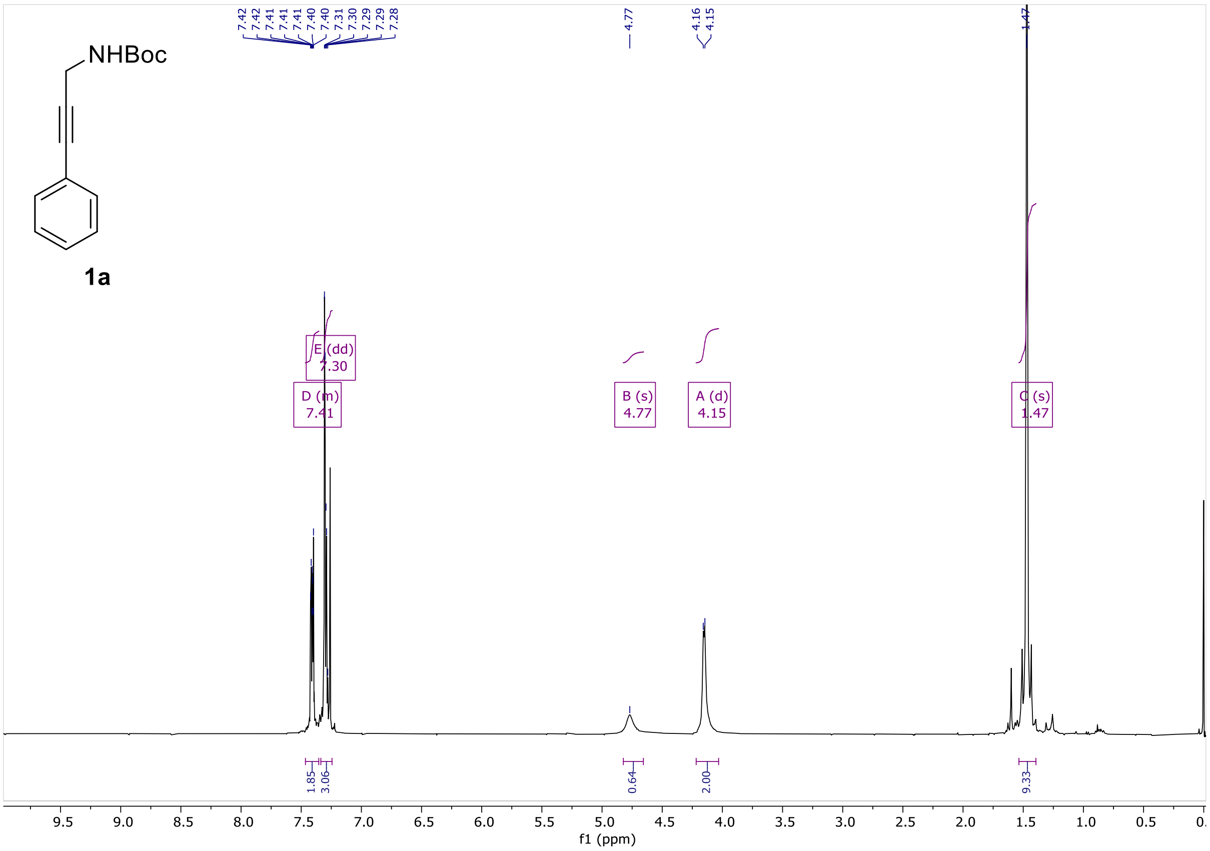

^{1}H-NMR (400 MHz, $CDCl_3$):

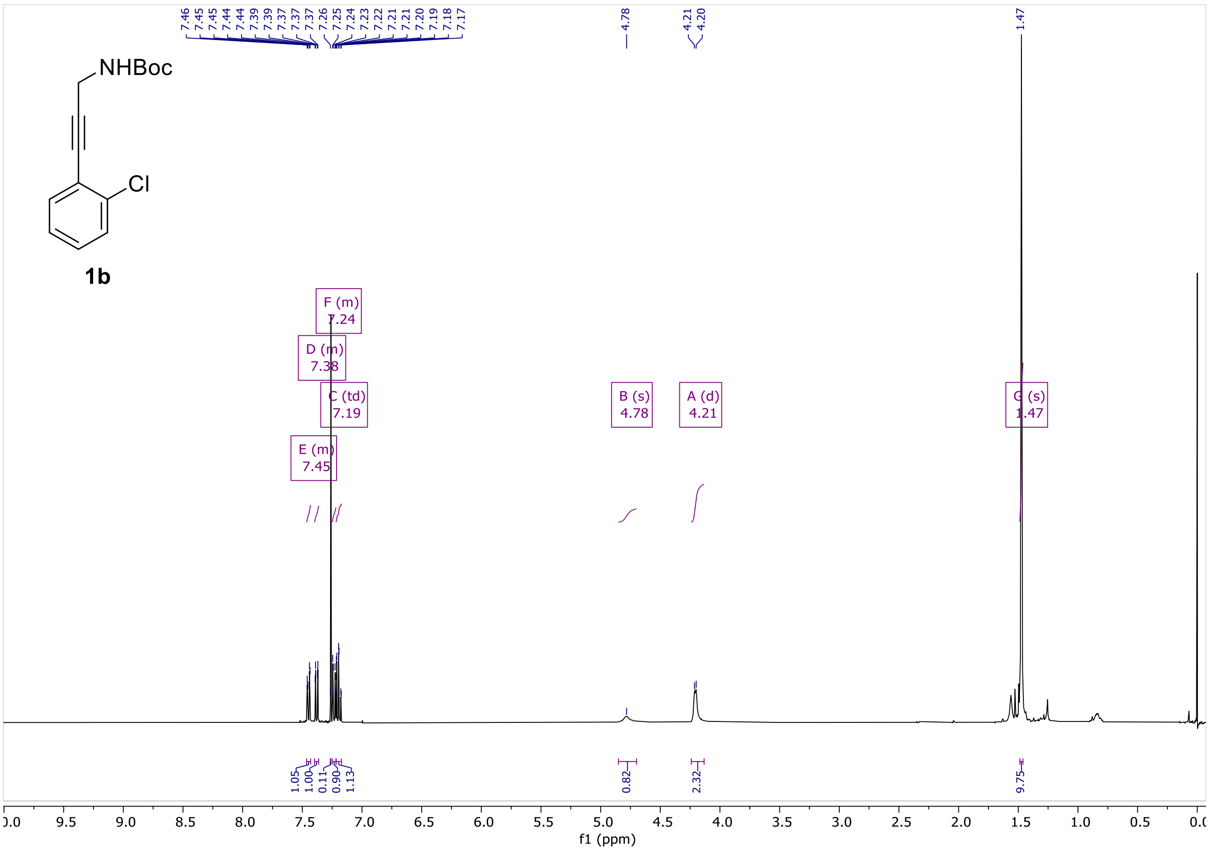

^{1}H-NMR (400 MHz, $CDCl_3$):

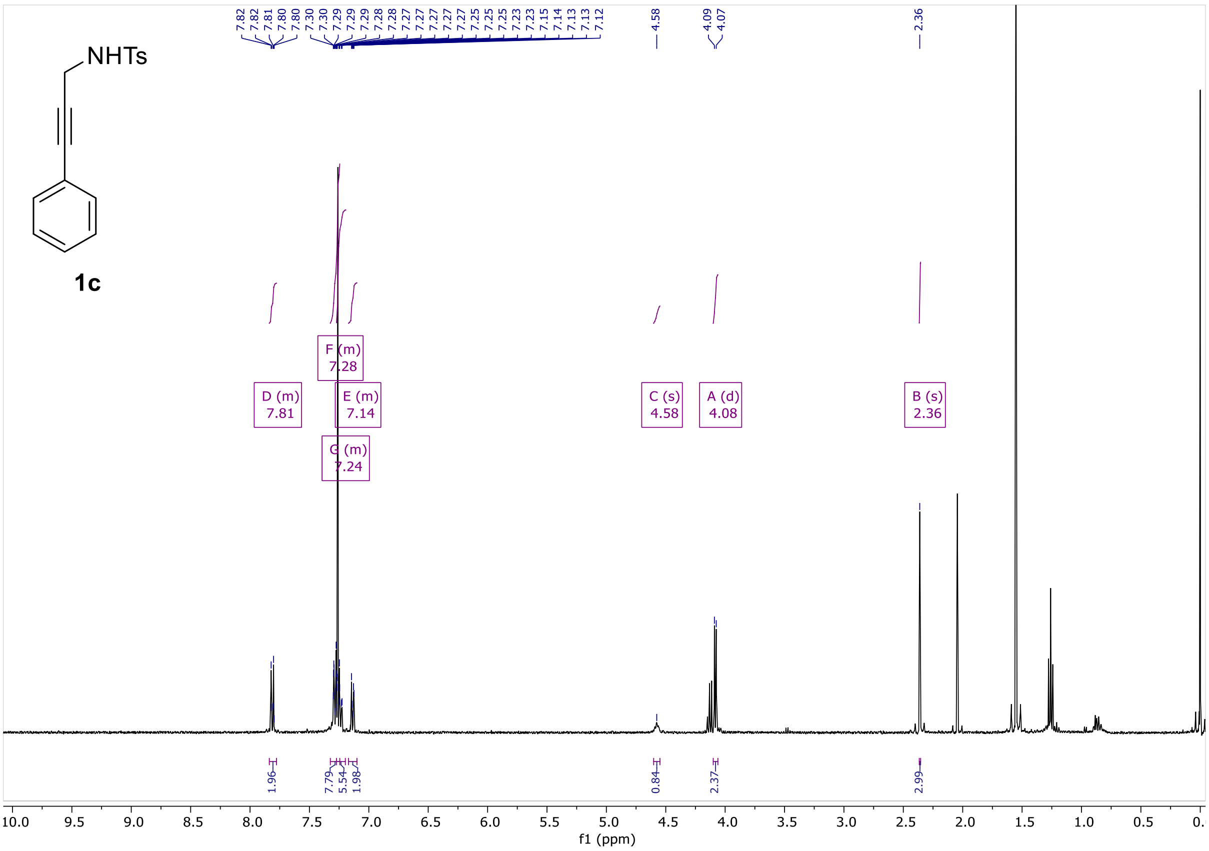

^{1}H-NMR (400 MHz, $CDCl_3$):

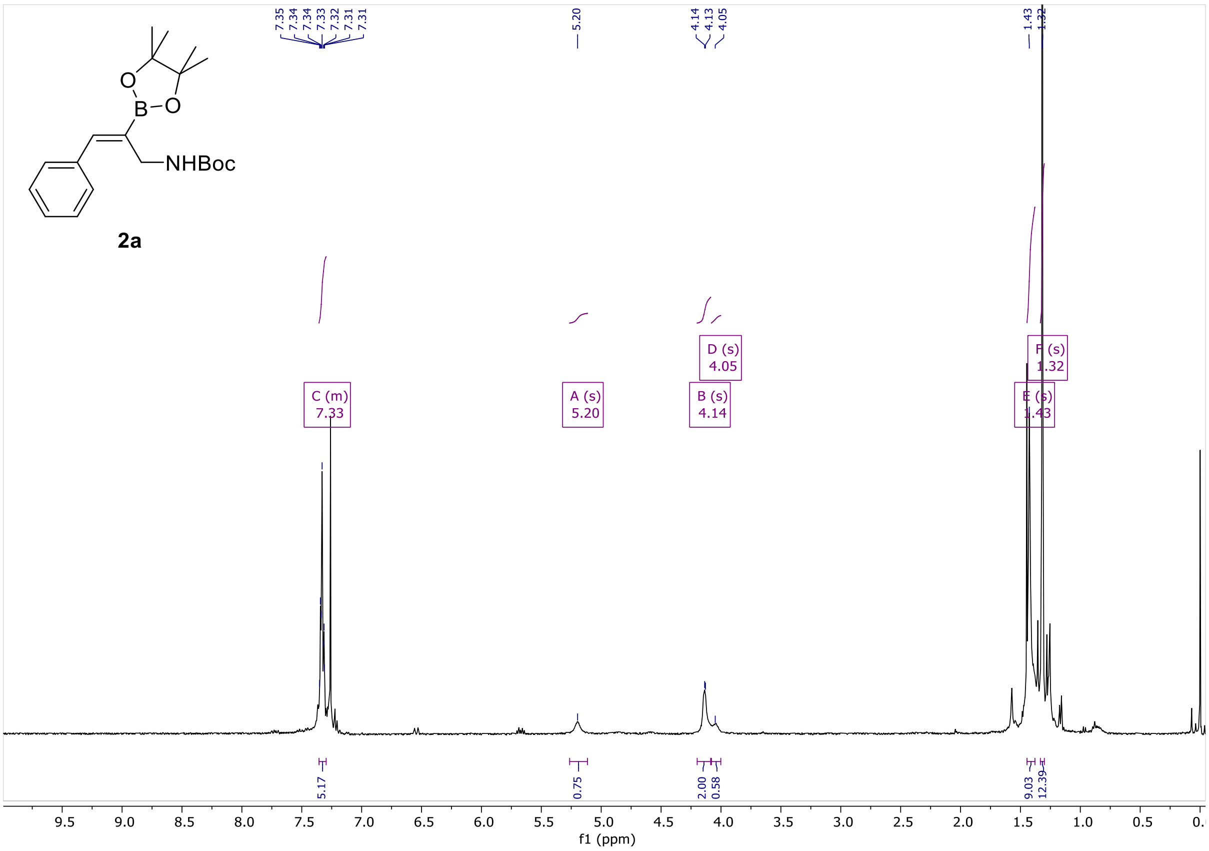

^{13}C-NMR (101 MHz, $CDCl_3$):

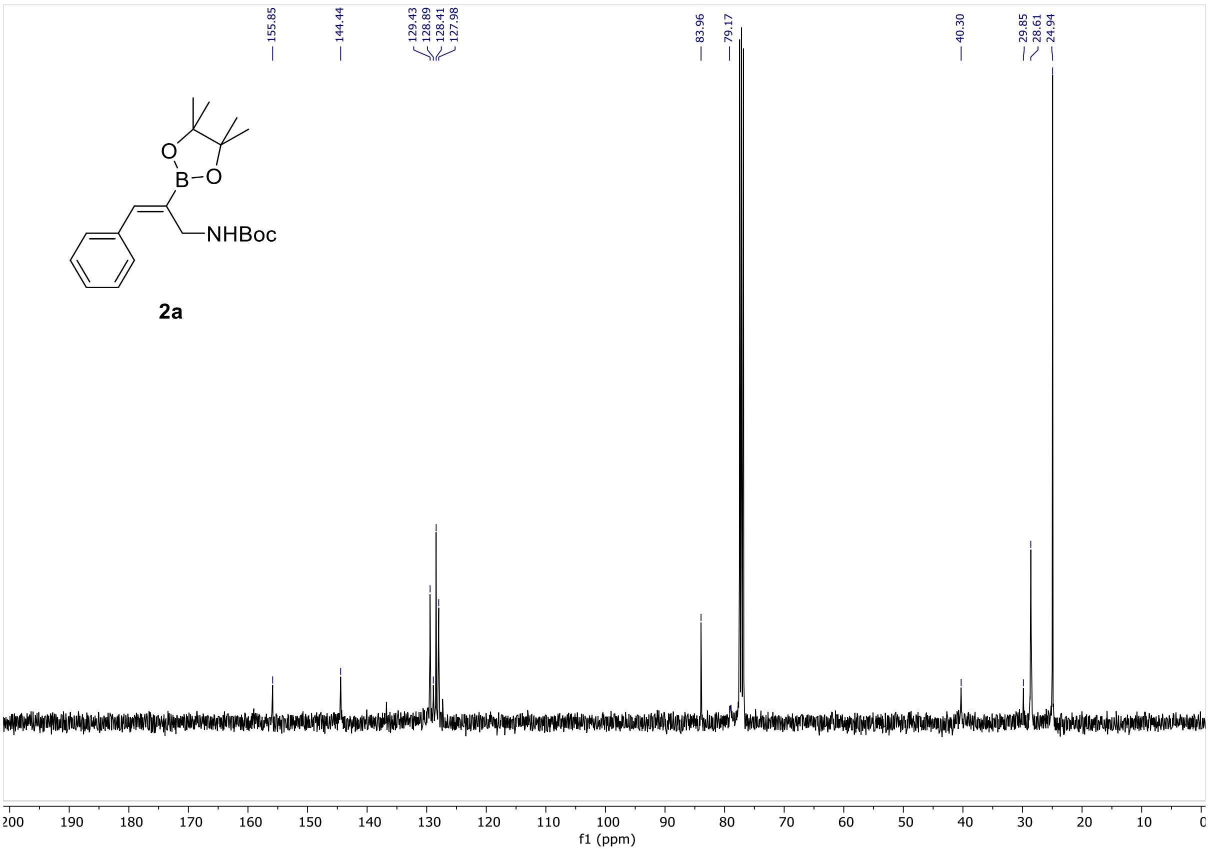

^{1}H-NMR (400 MHz, $CDCl_3$):

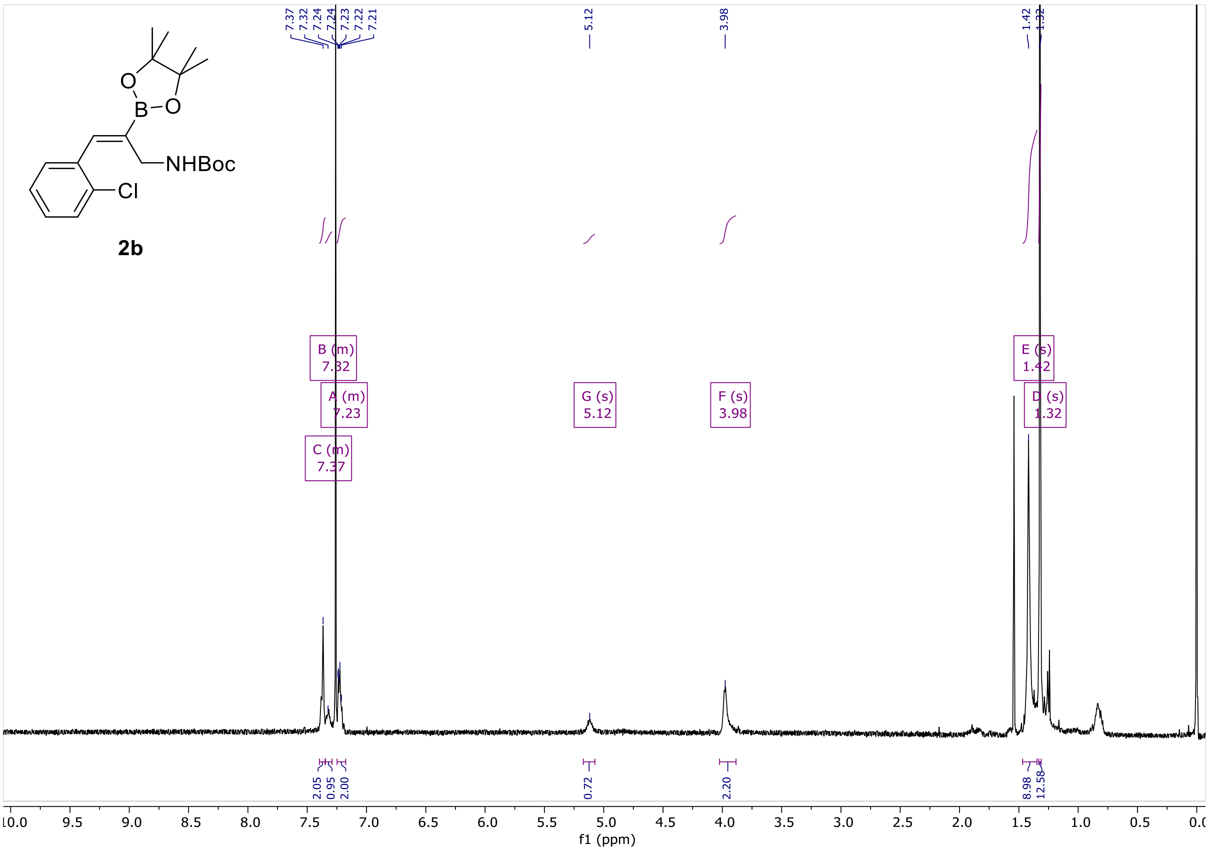

^{13}C-NMR (101 MHz, $CDCl_3$):

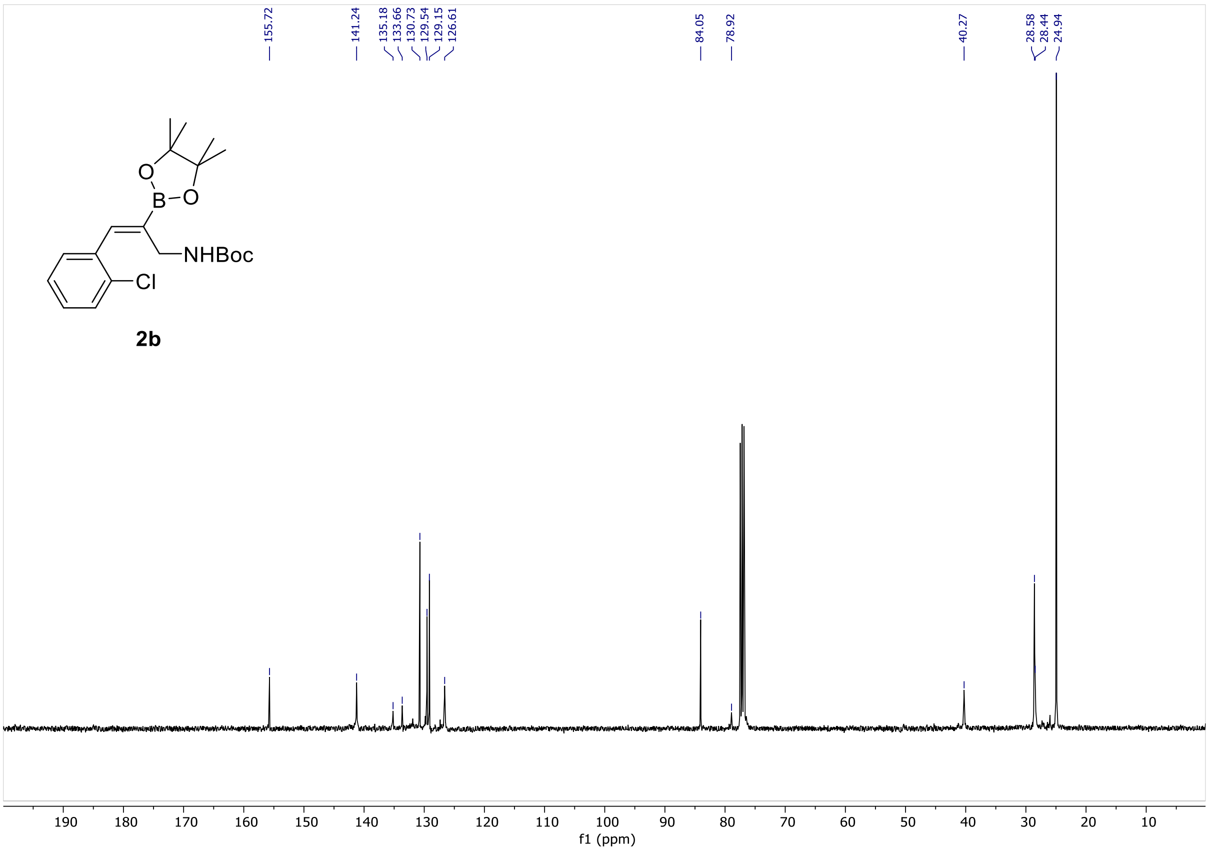

^{1}H-NMR (400 MHz, $CDCl_3$):

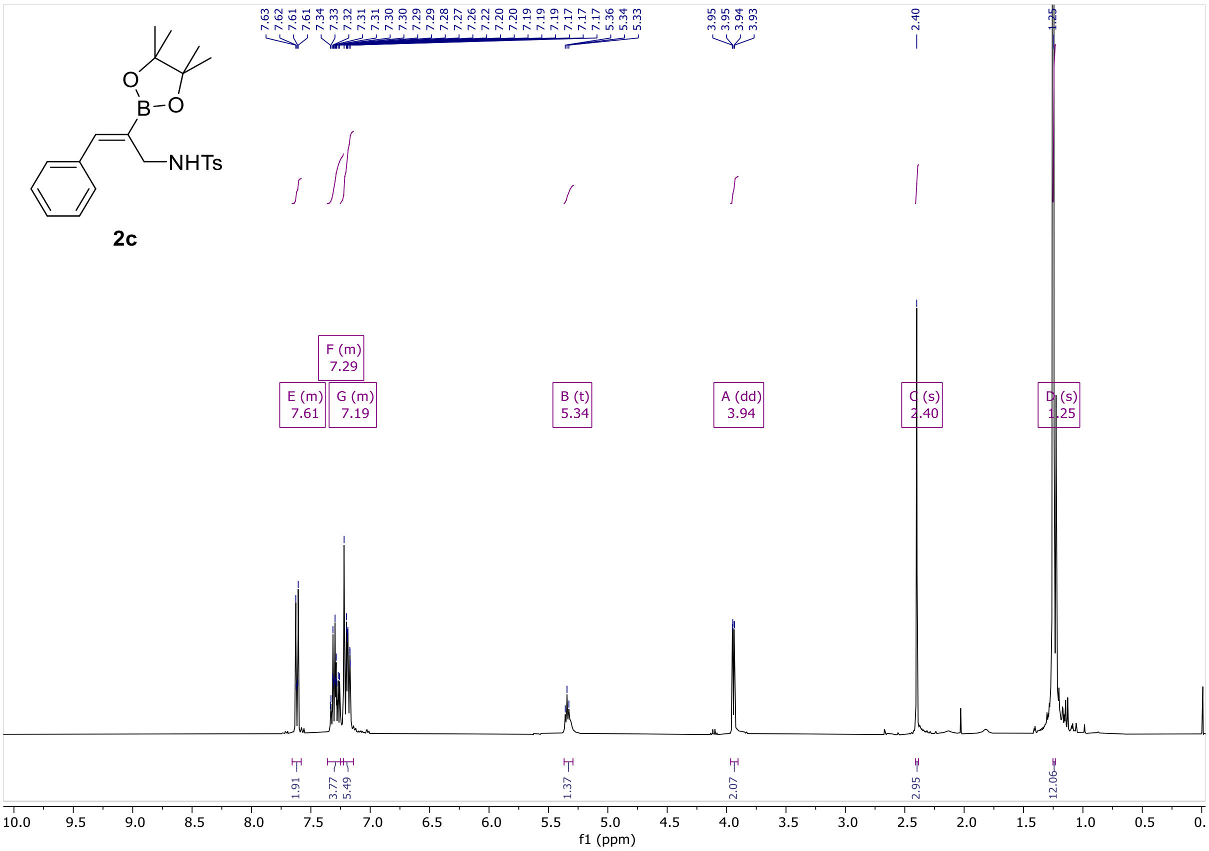

^{13}C-NMR (101 MHz, $CDCl_3$):

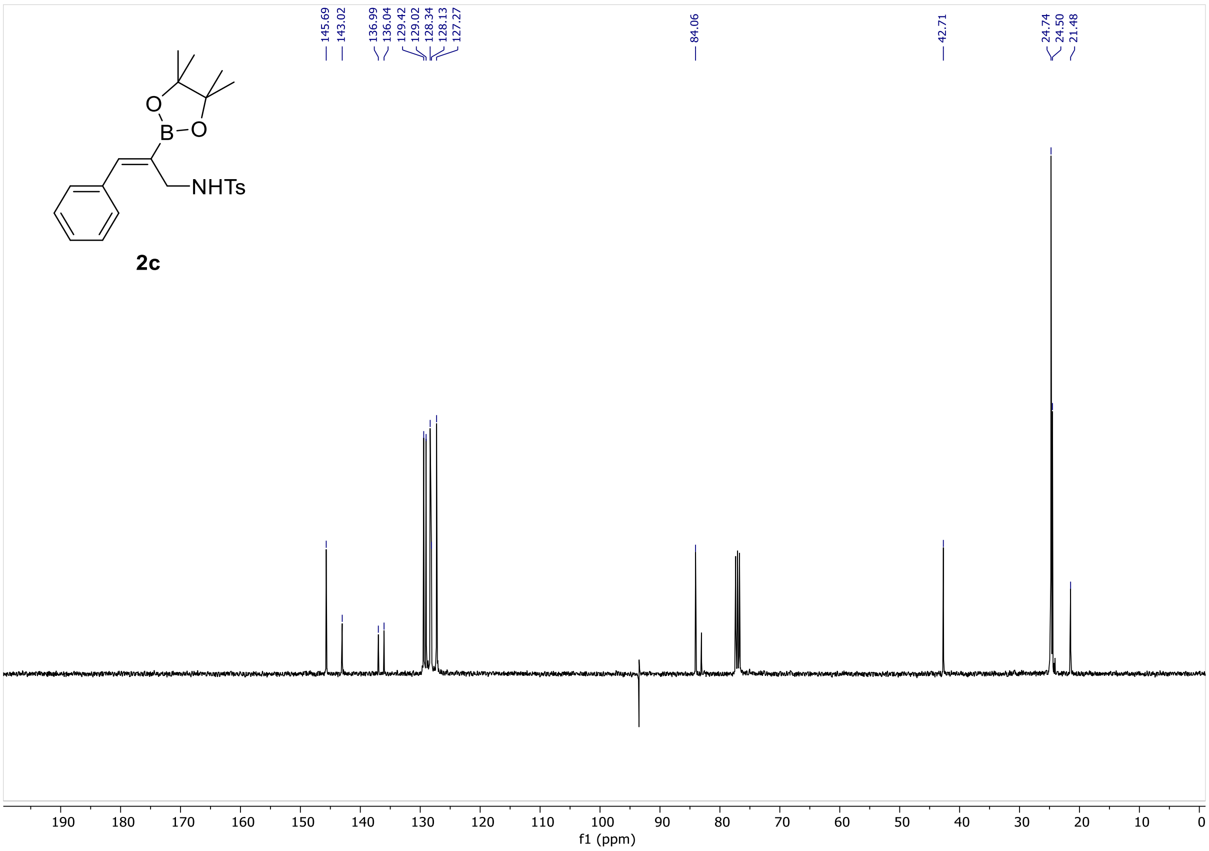

^{1}H-NMR (400 MHz, $CDCl_3$):

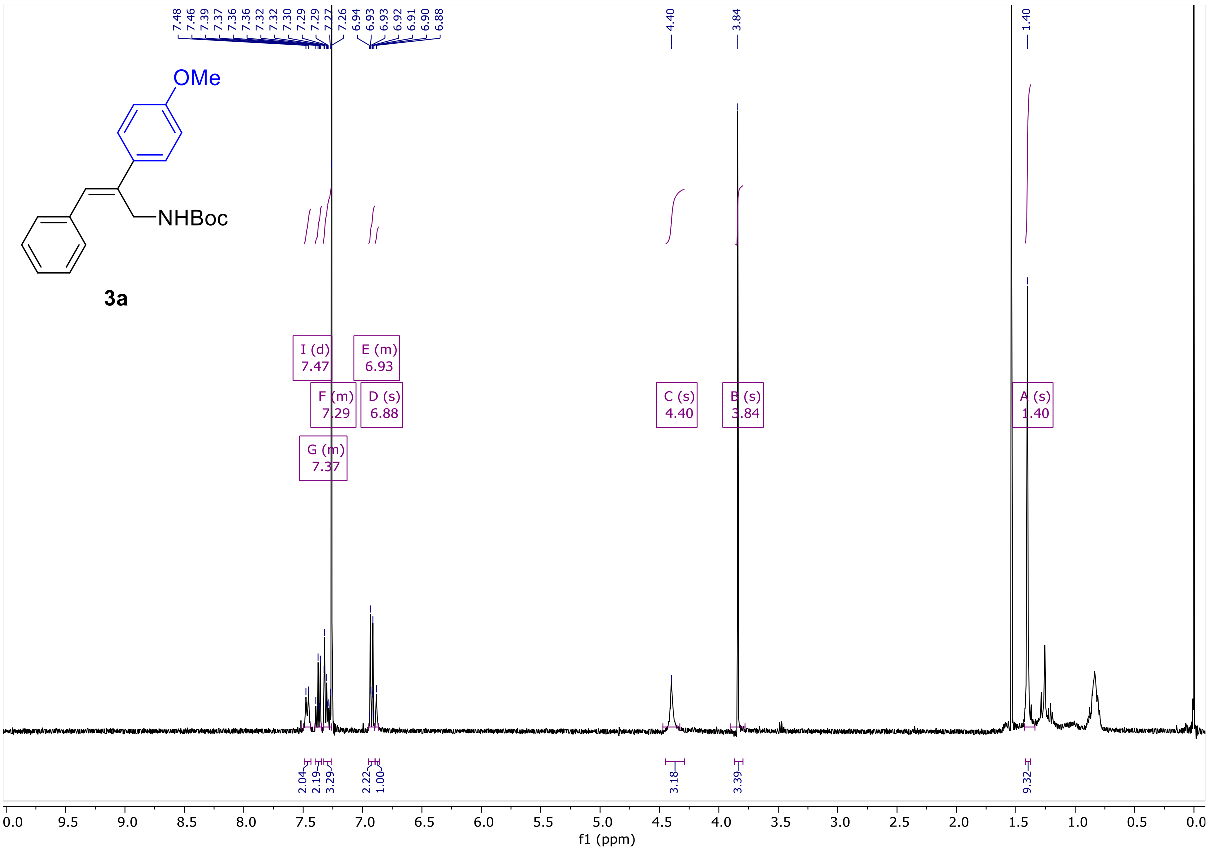

^{13}C-NMR (101 MHz, $CDCl_3$):

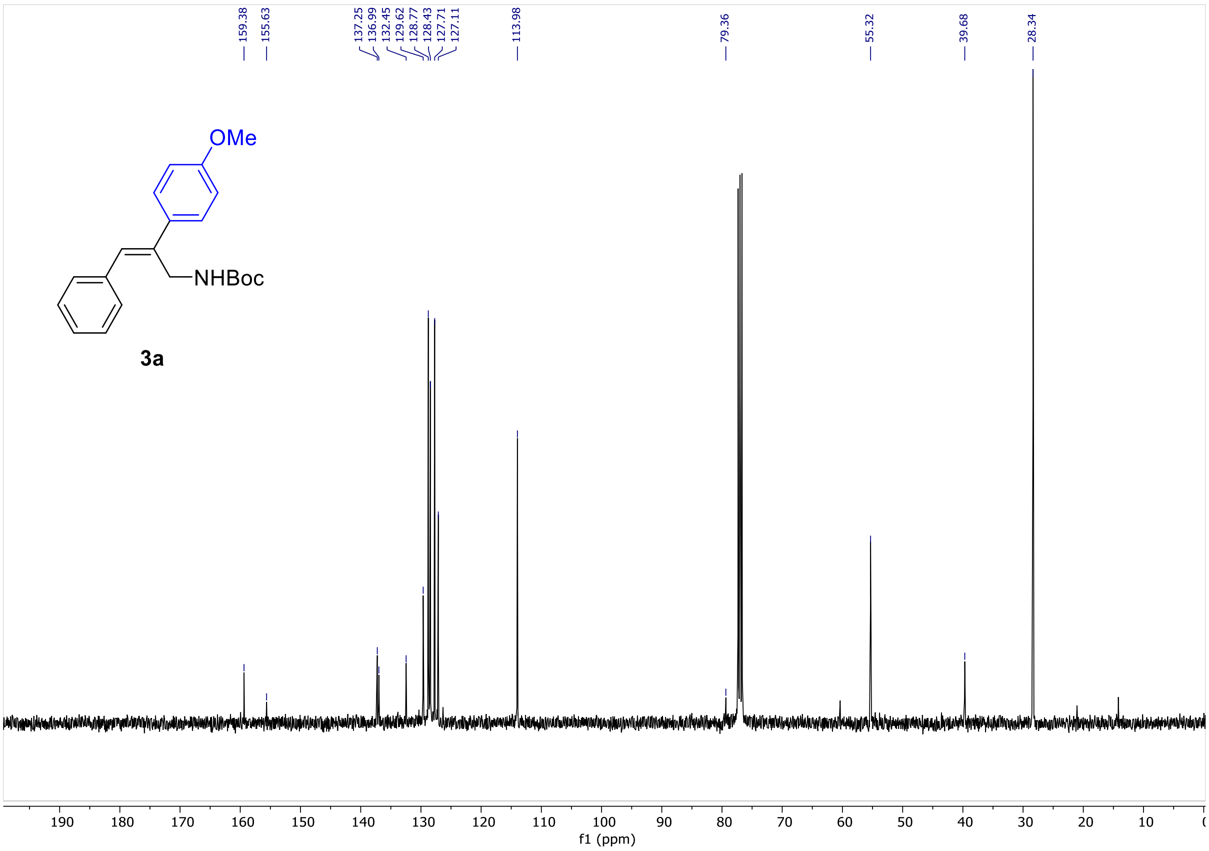

^{1}H-NMR (400 MHz, $CDCl_3$):

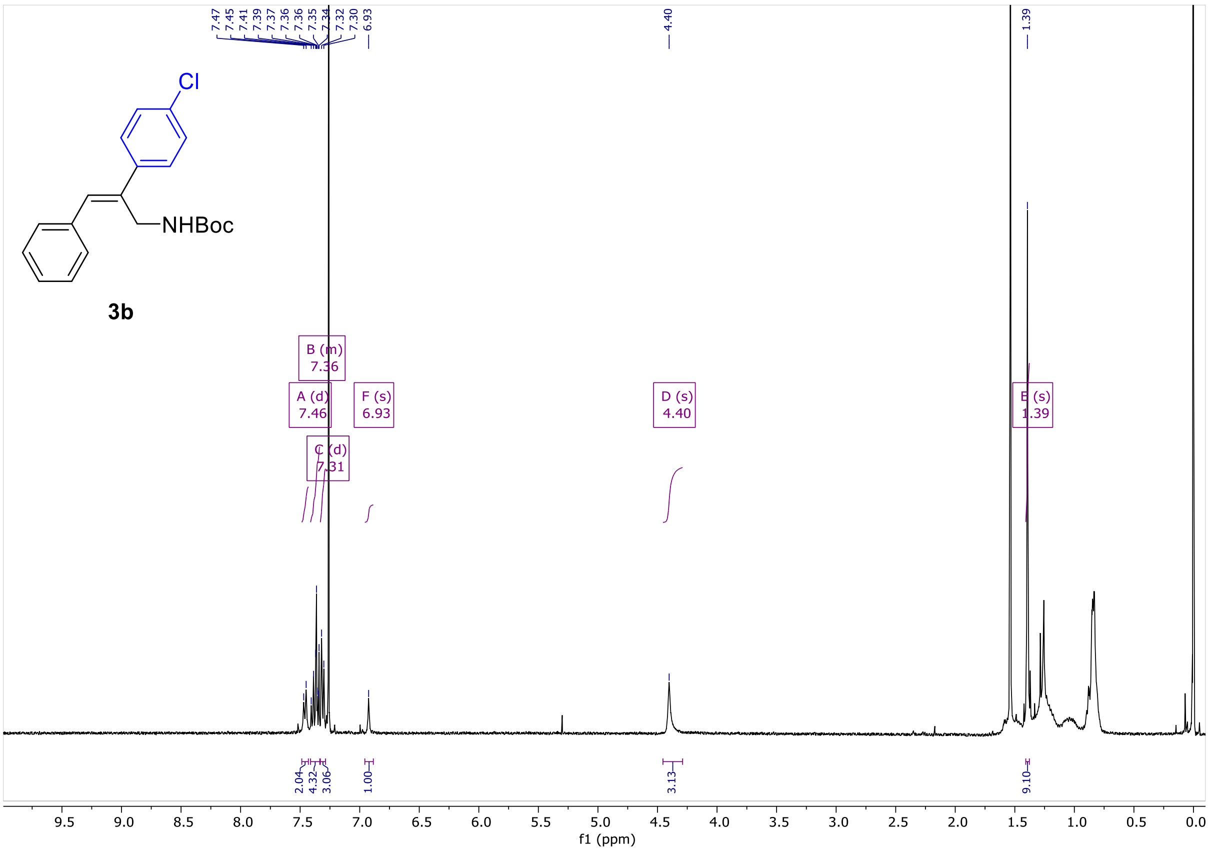

^{13}C-NMR (101 MHz, $CDCl_3$):

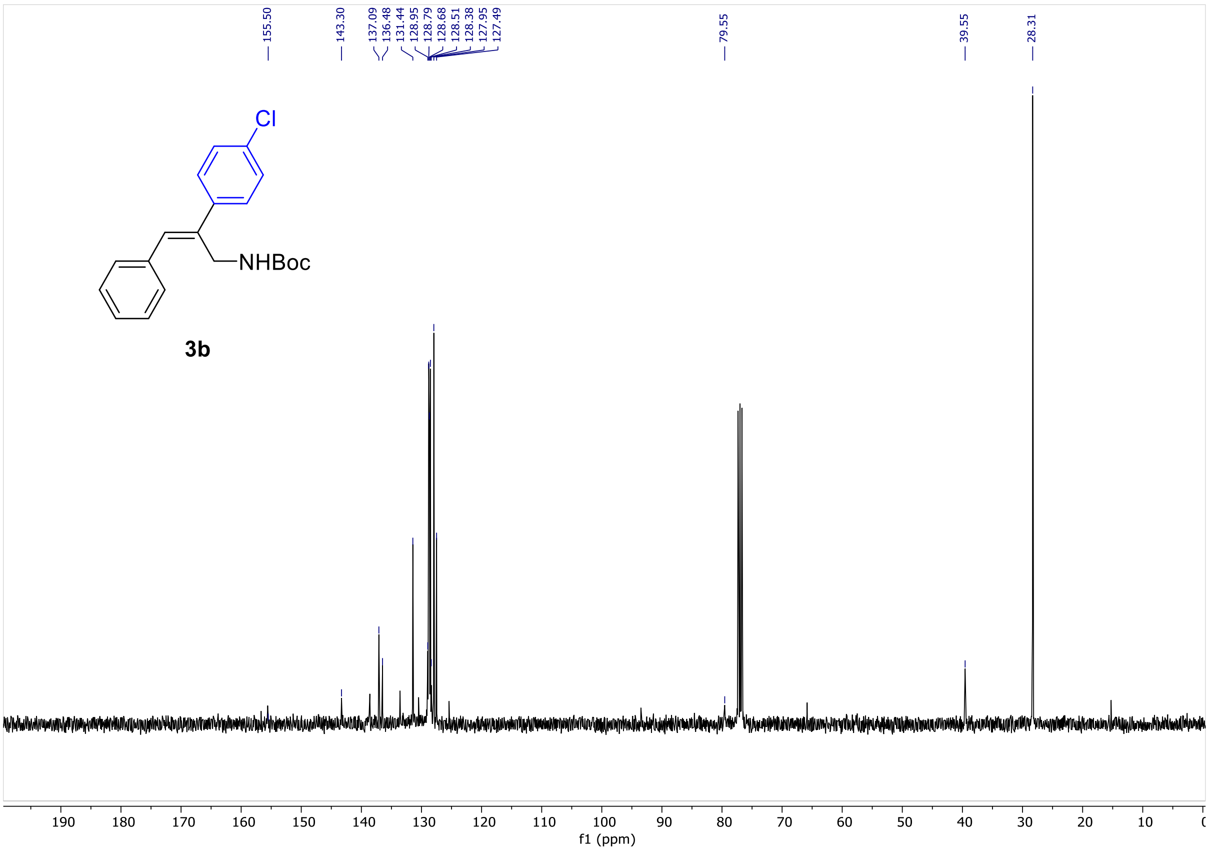

^{1}H-NMR (400 MHz, $CDCl_3$):

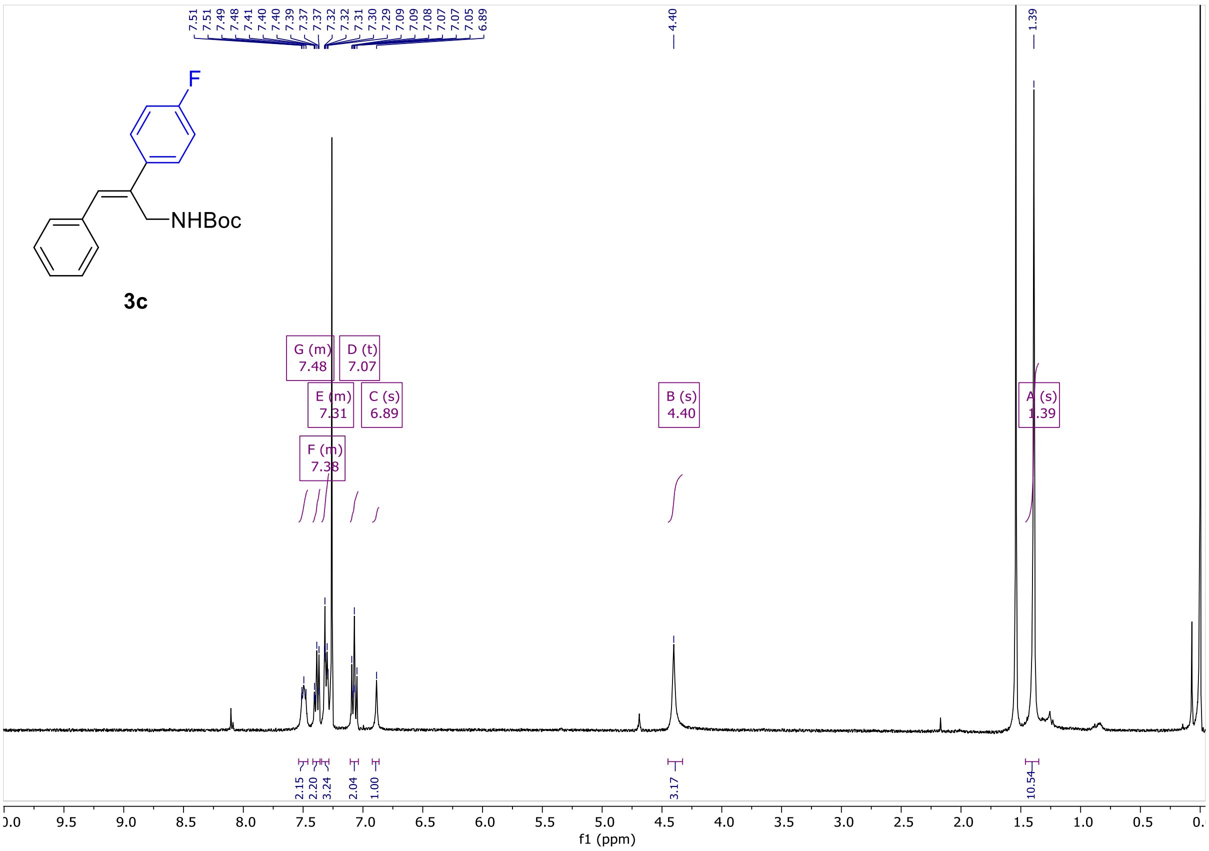

^{13}C-NMR (101 MHz, $CDCl_3$):

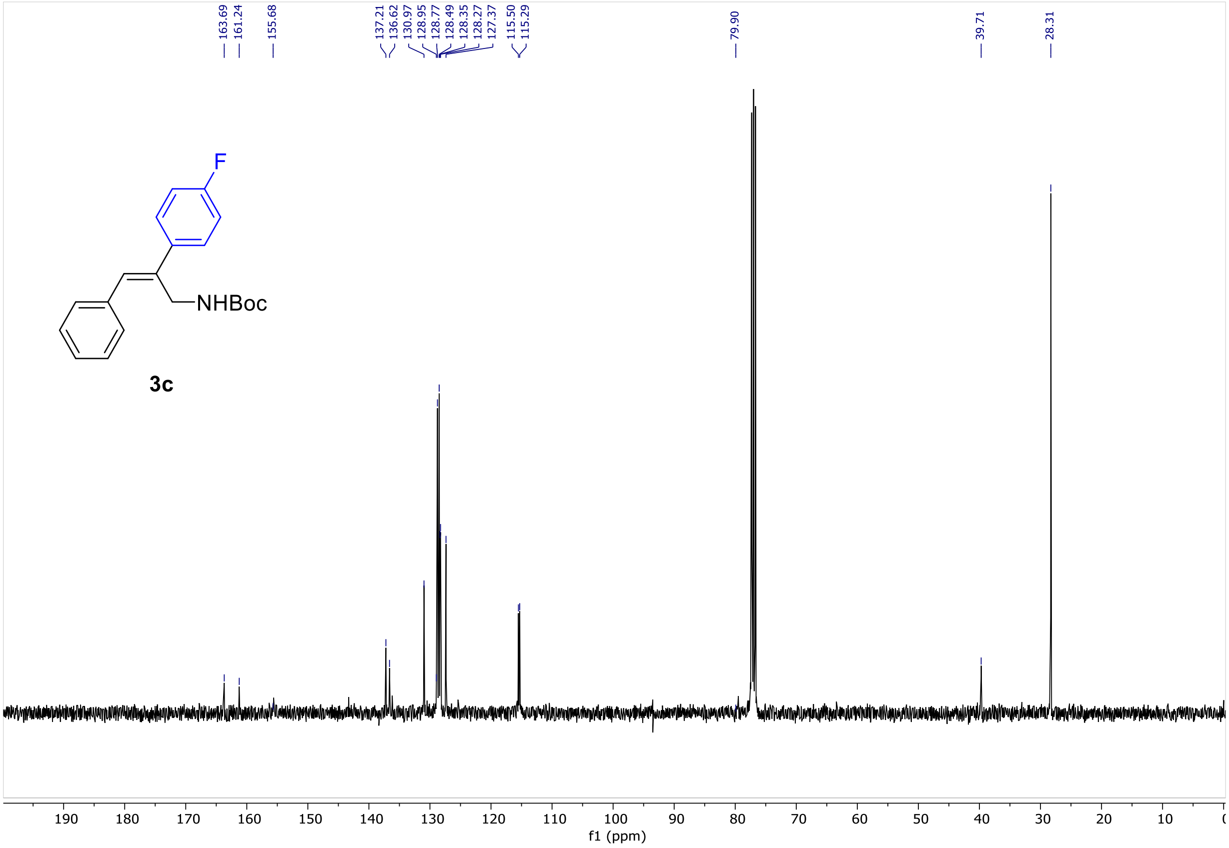

^{1}H-NMR (400 MHz, $CDCl_3$):

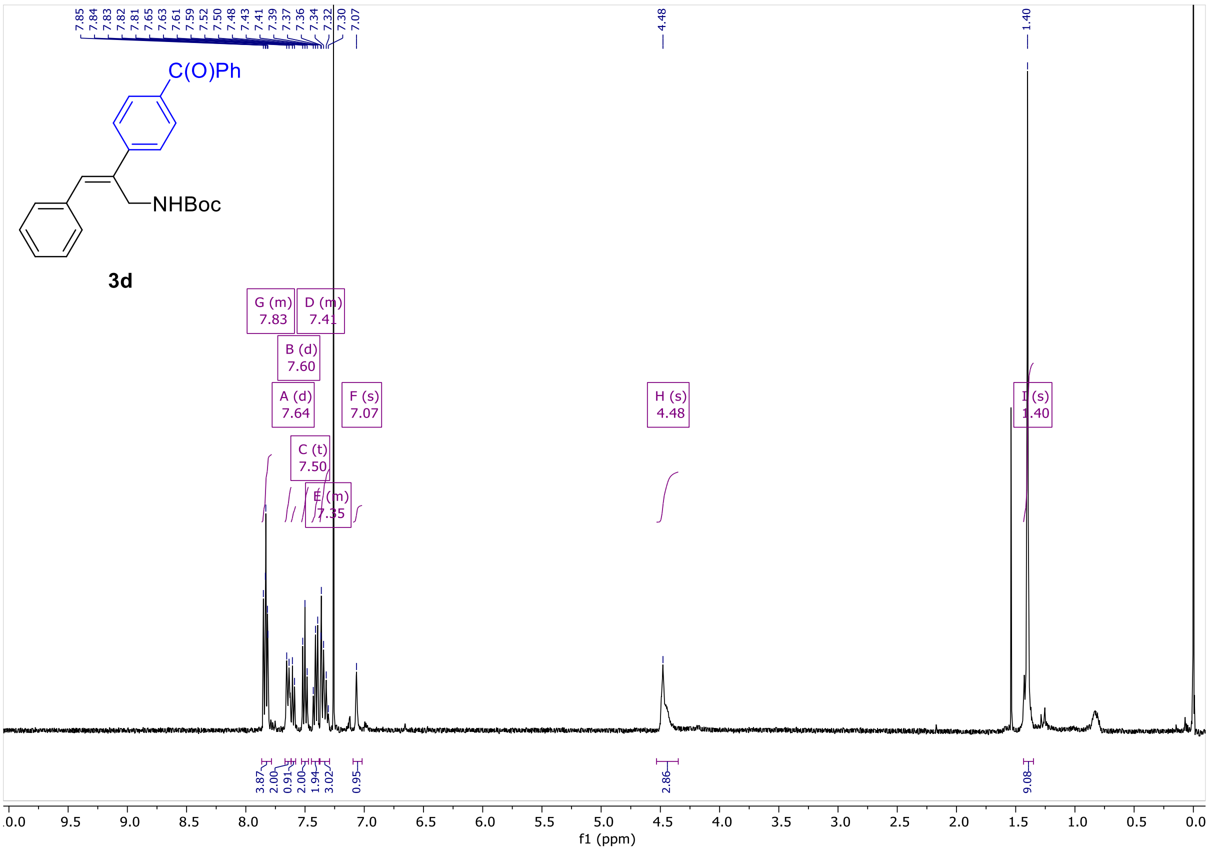

^{13}C-NMR (101 MHz, $CDCl_3$):

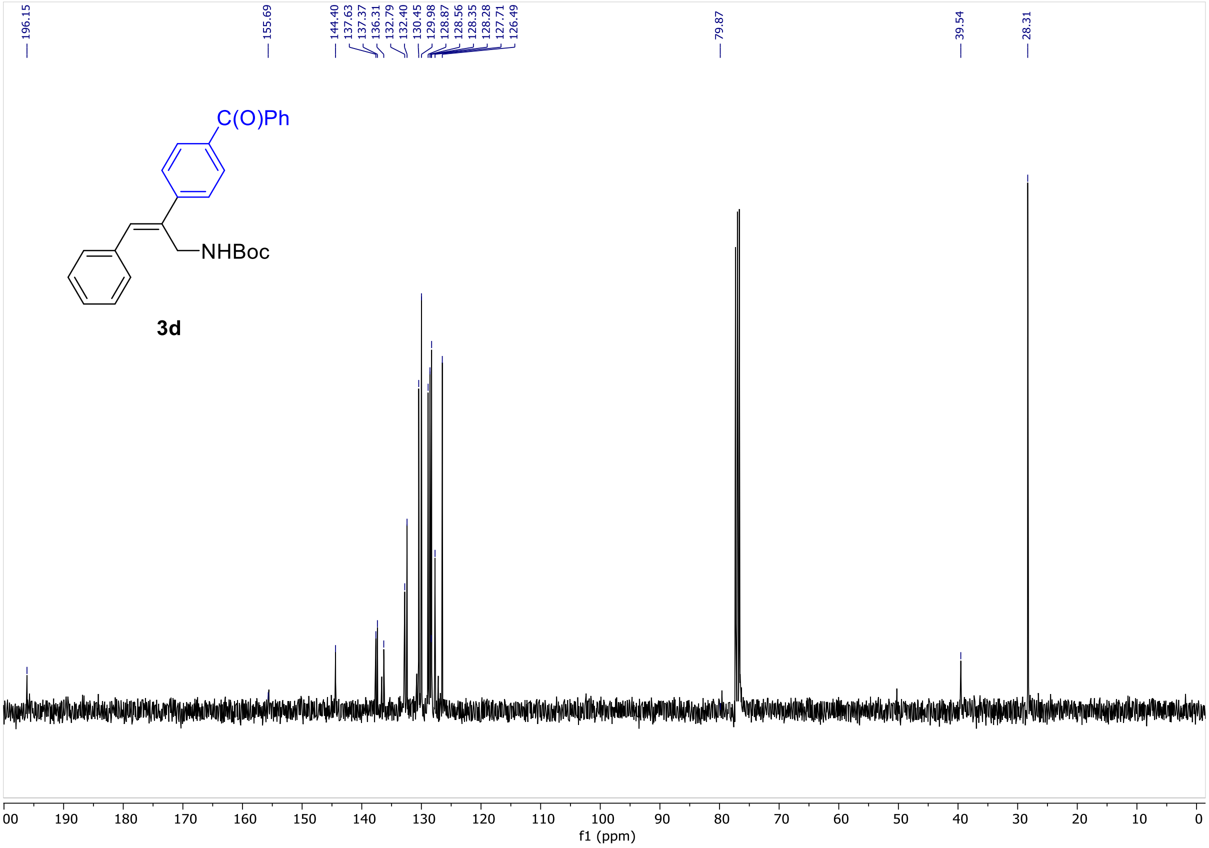

^{1}H-NMR (400 MHz, $CDCl_3$):

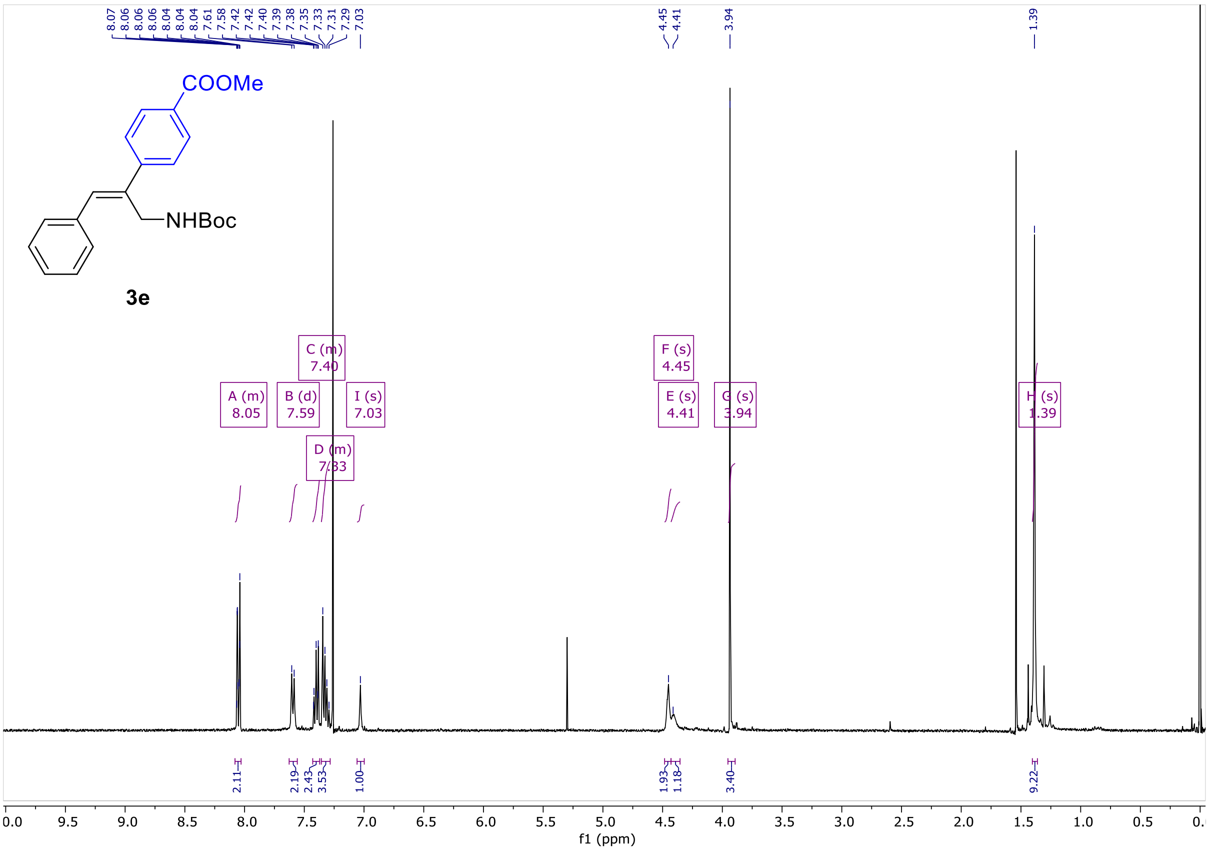

^{13}C-NMR (101 MHz, $CDCl_3$):

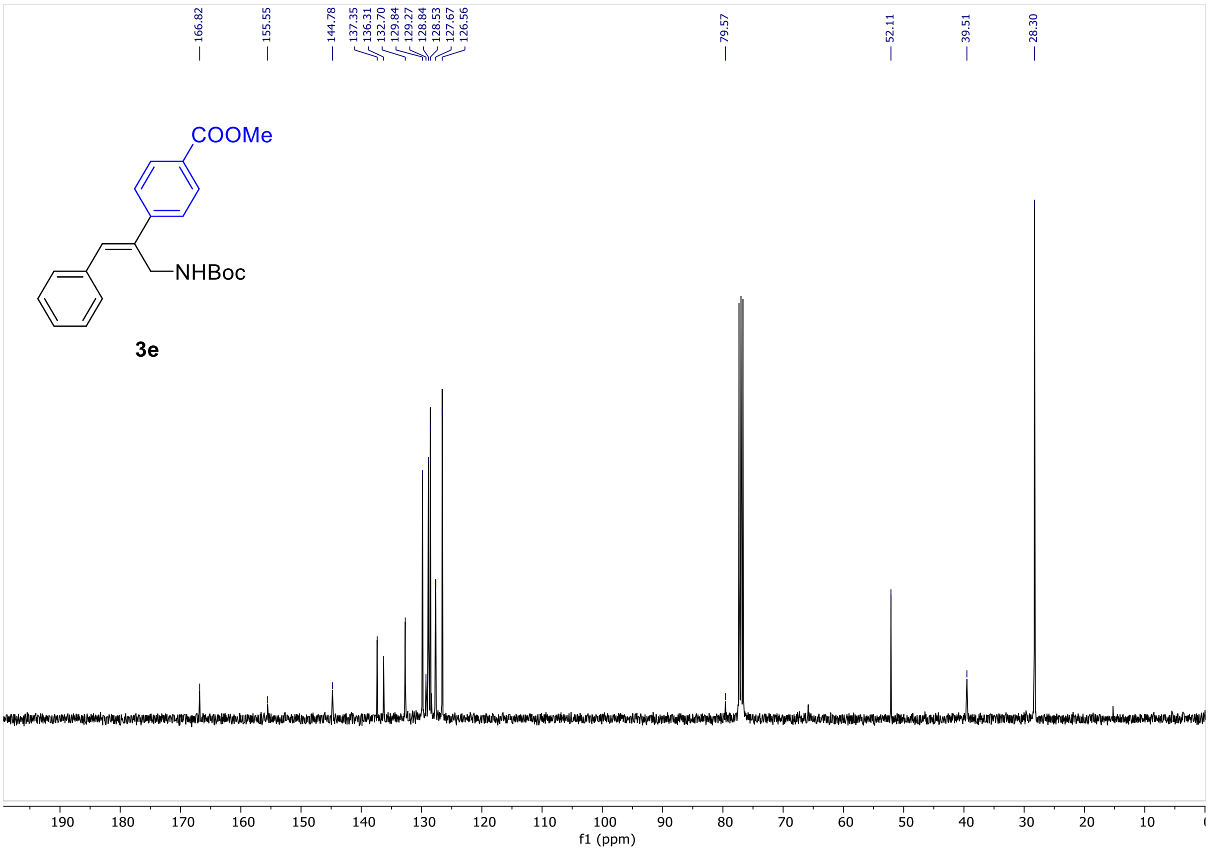

^{1}H-NMR (400 MHz, $CDCl_3$):

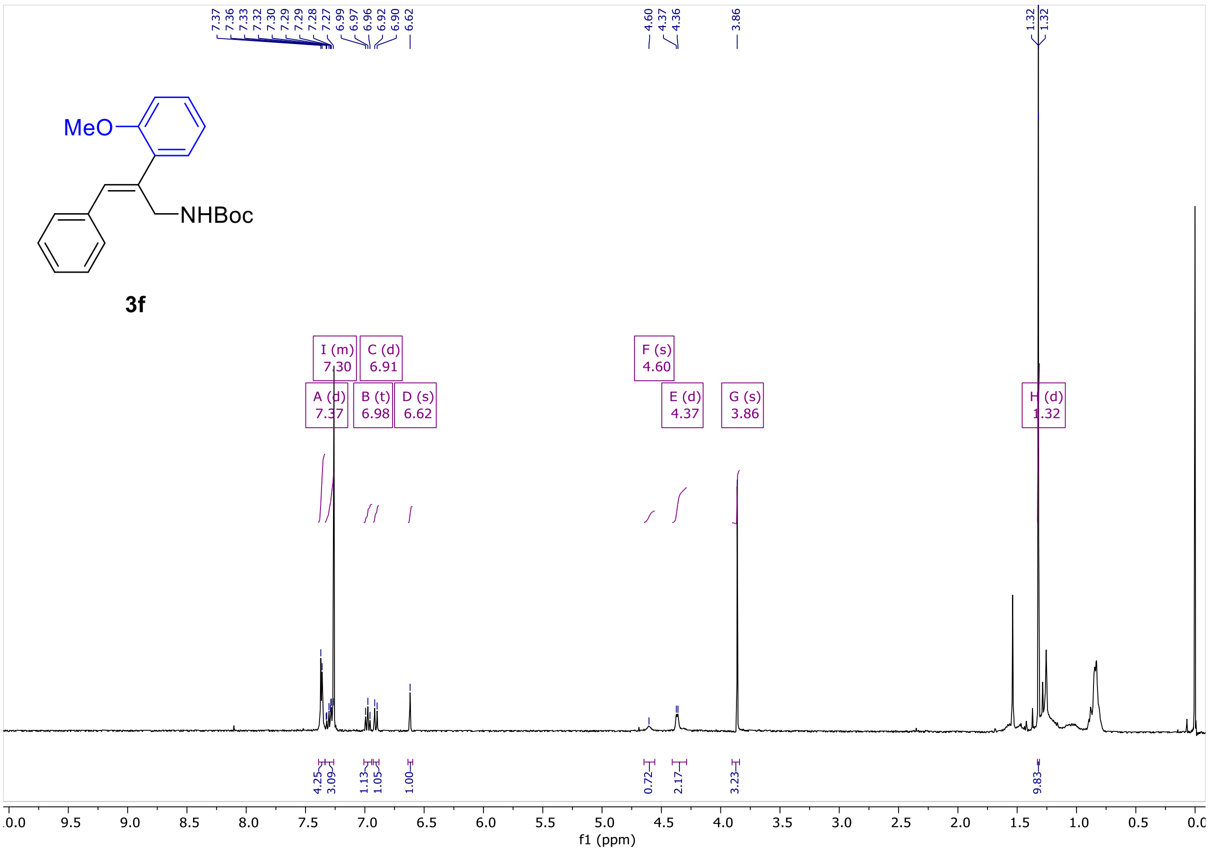

^{13}C-NMR (101 MHz, $CDCl_3$):

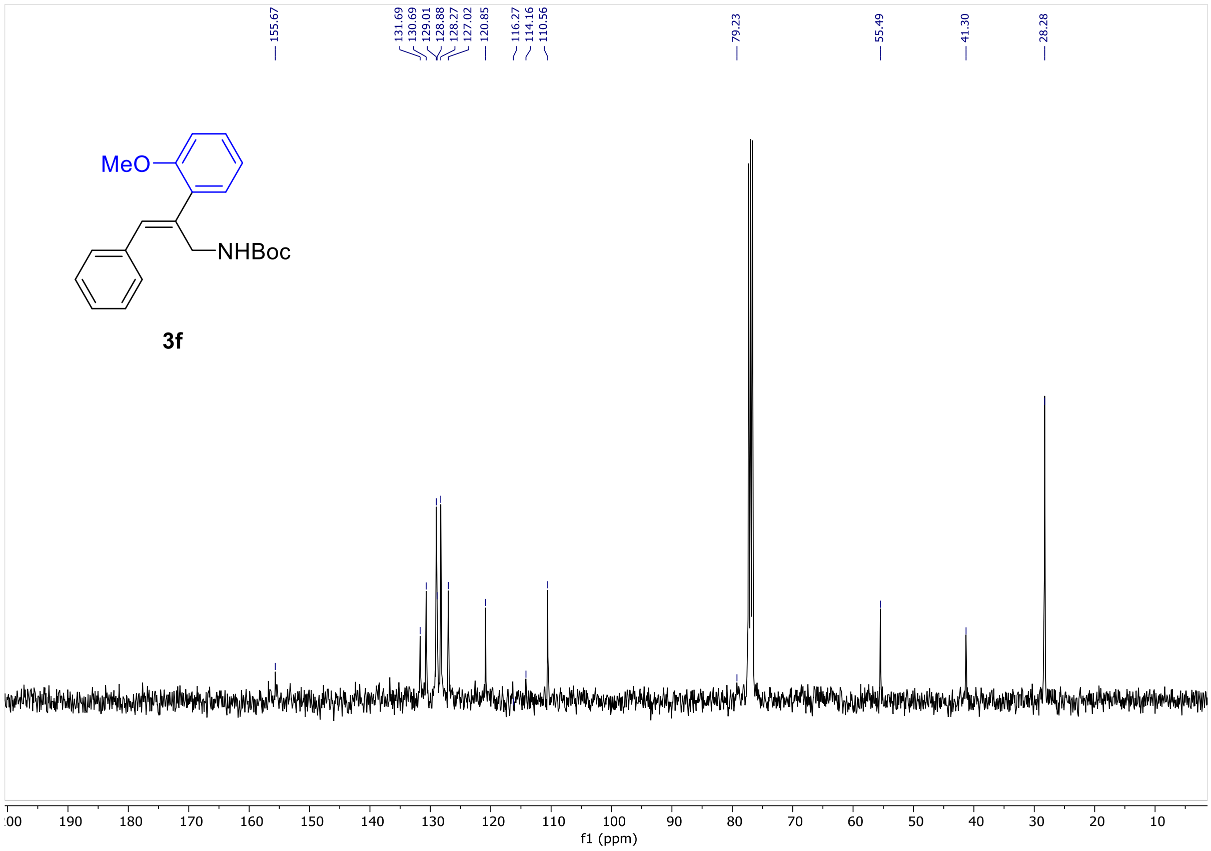

^{1}H-NMR (400 MHz, $CDCl_3$):

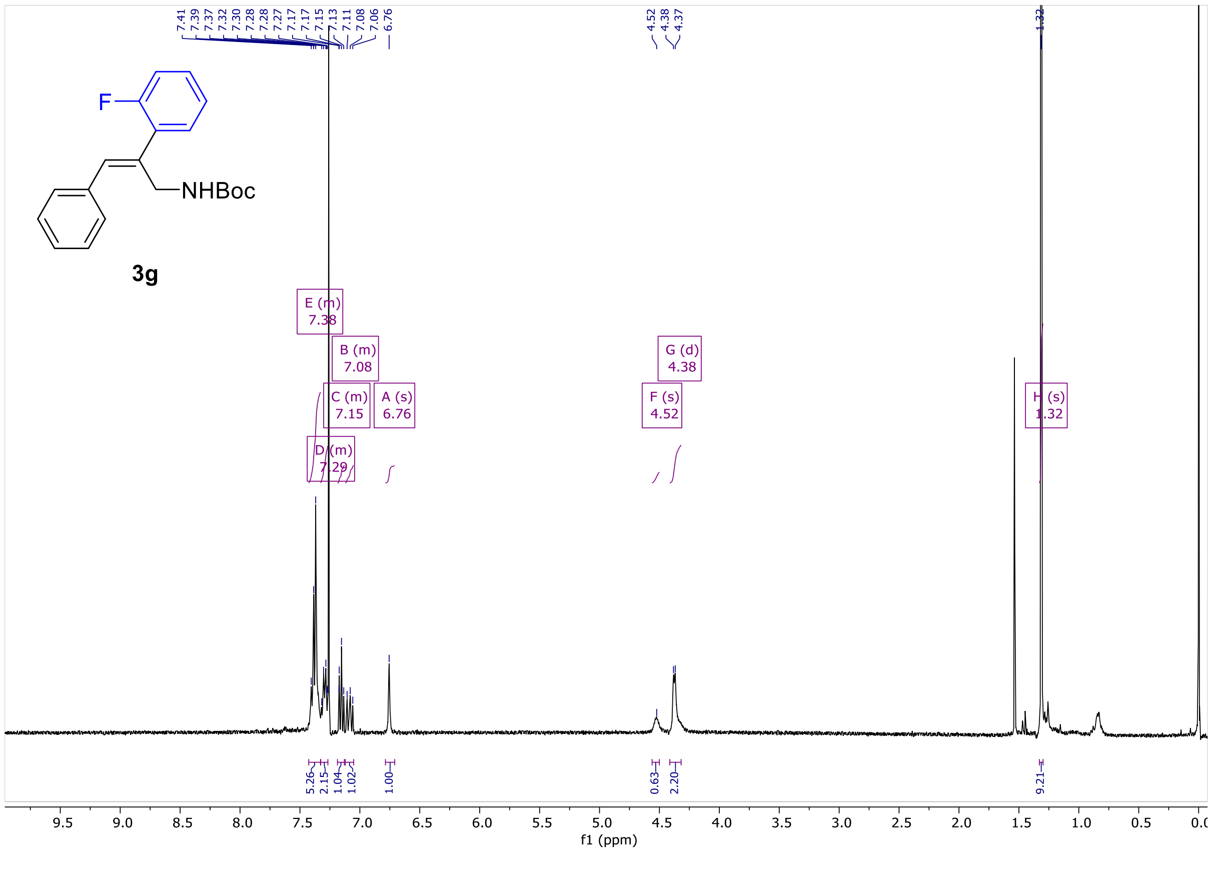

^{13}C-NMR (101 MHz, $CDCl_3$):

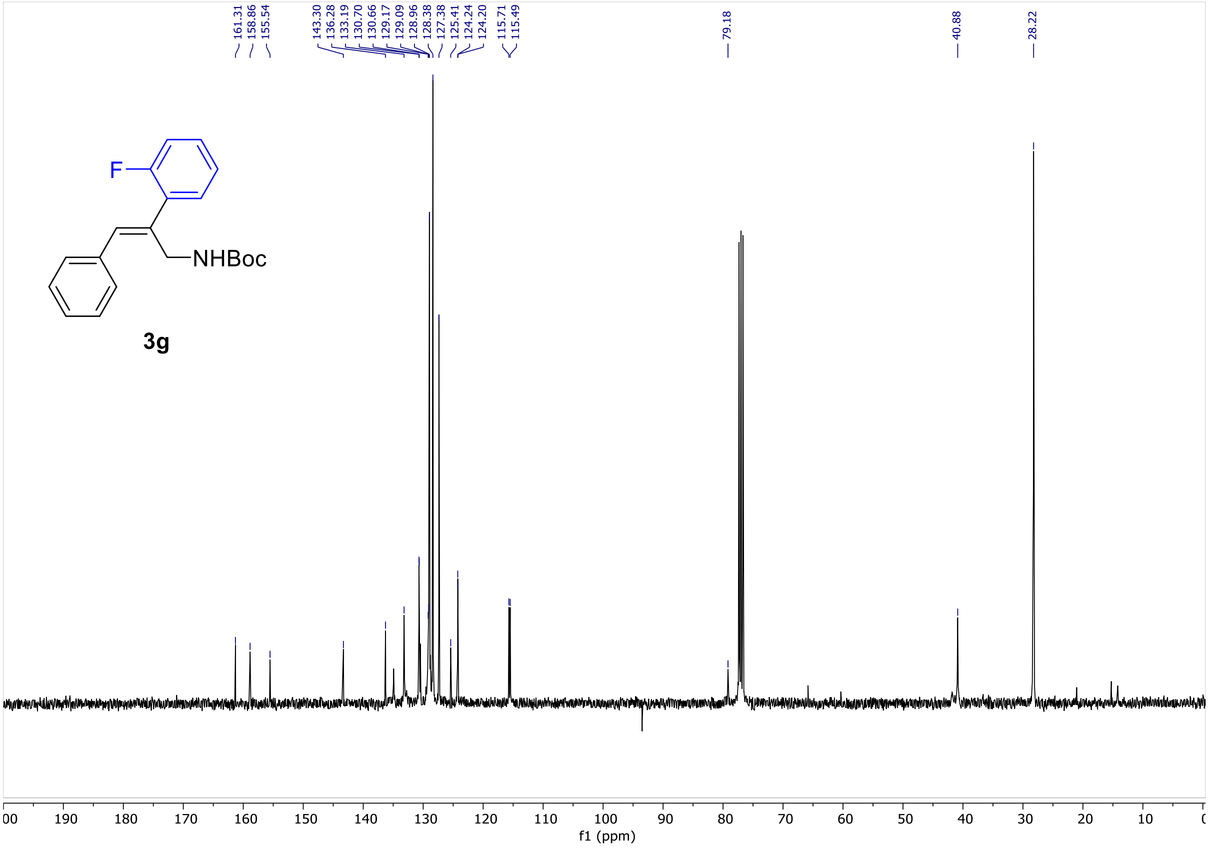

^{1}H-NMR (400 MHz, $CDCl_3$):

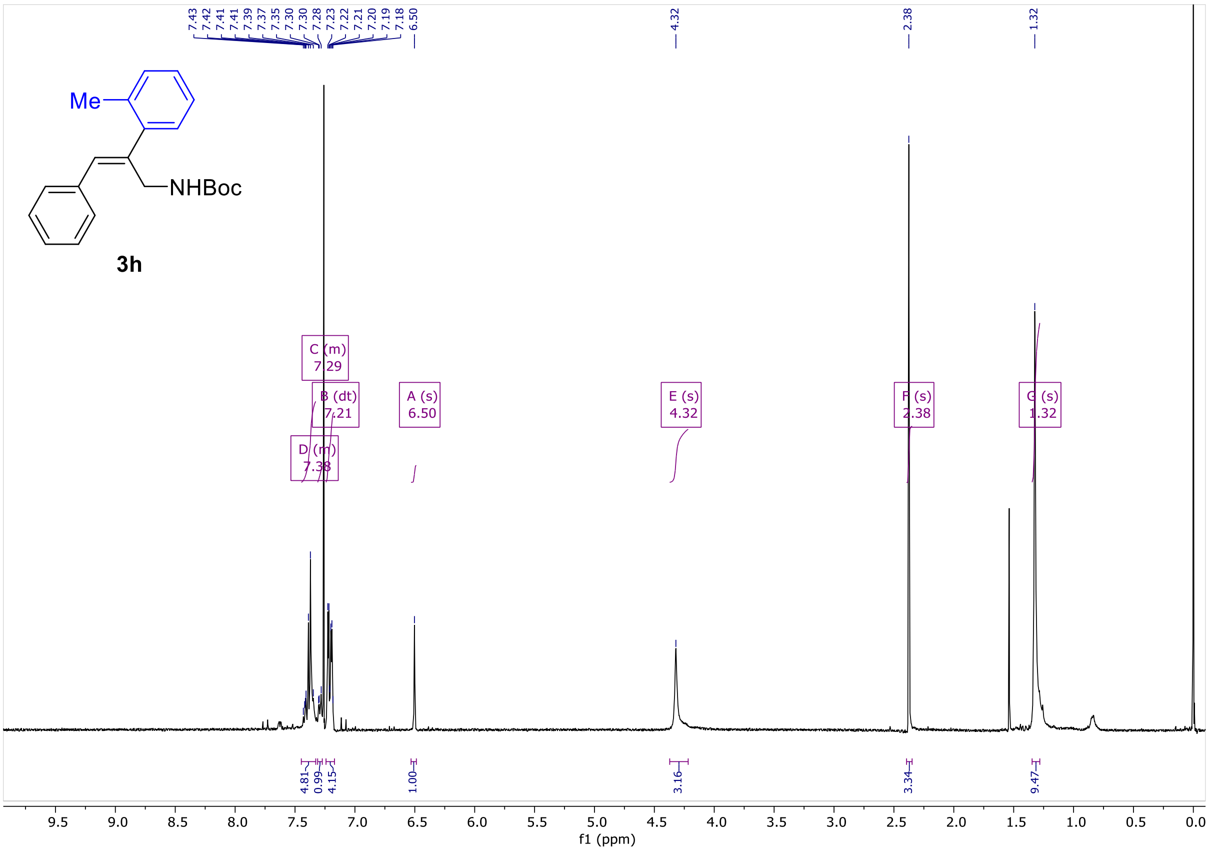

^{13}C-NMR (101 MHz, $CDCl_3$):

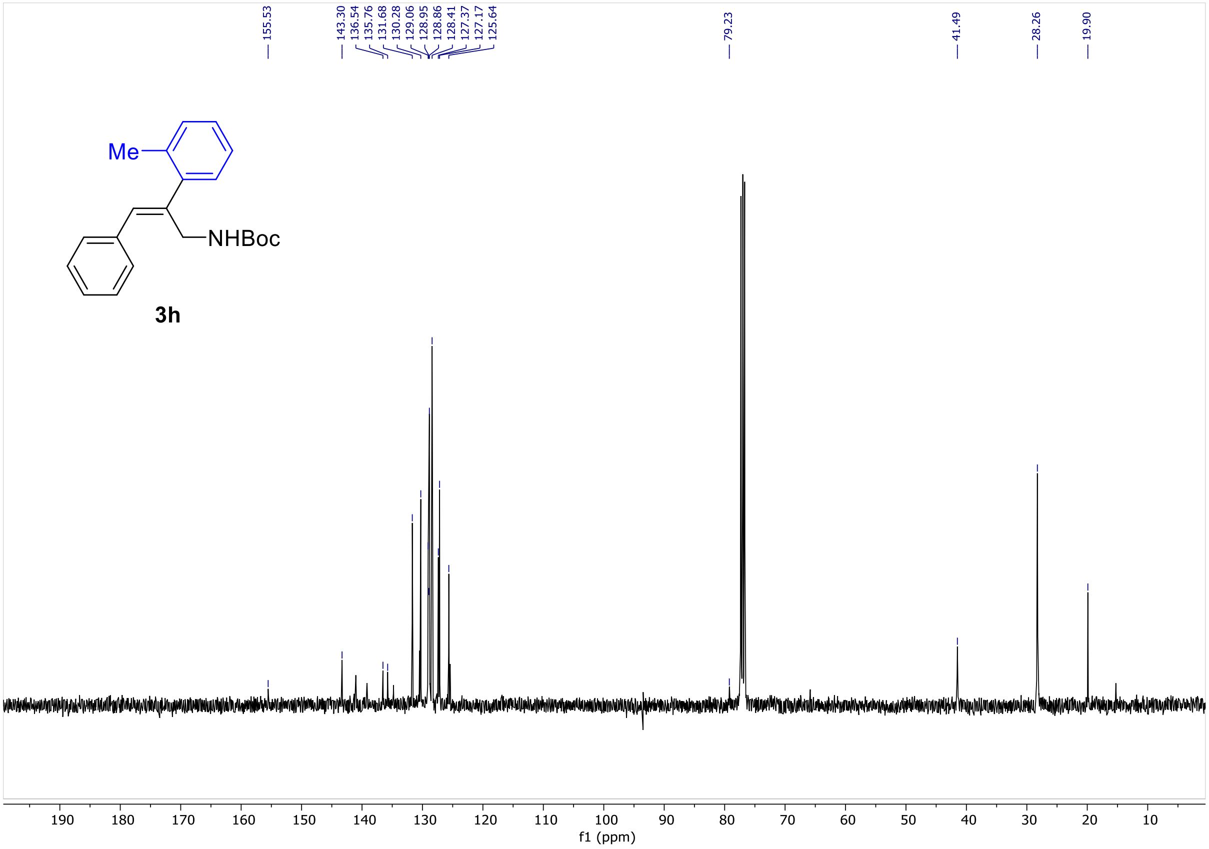

^{1}H-NMR (400 MHz, $CDCl_3$):

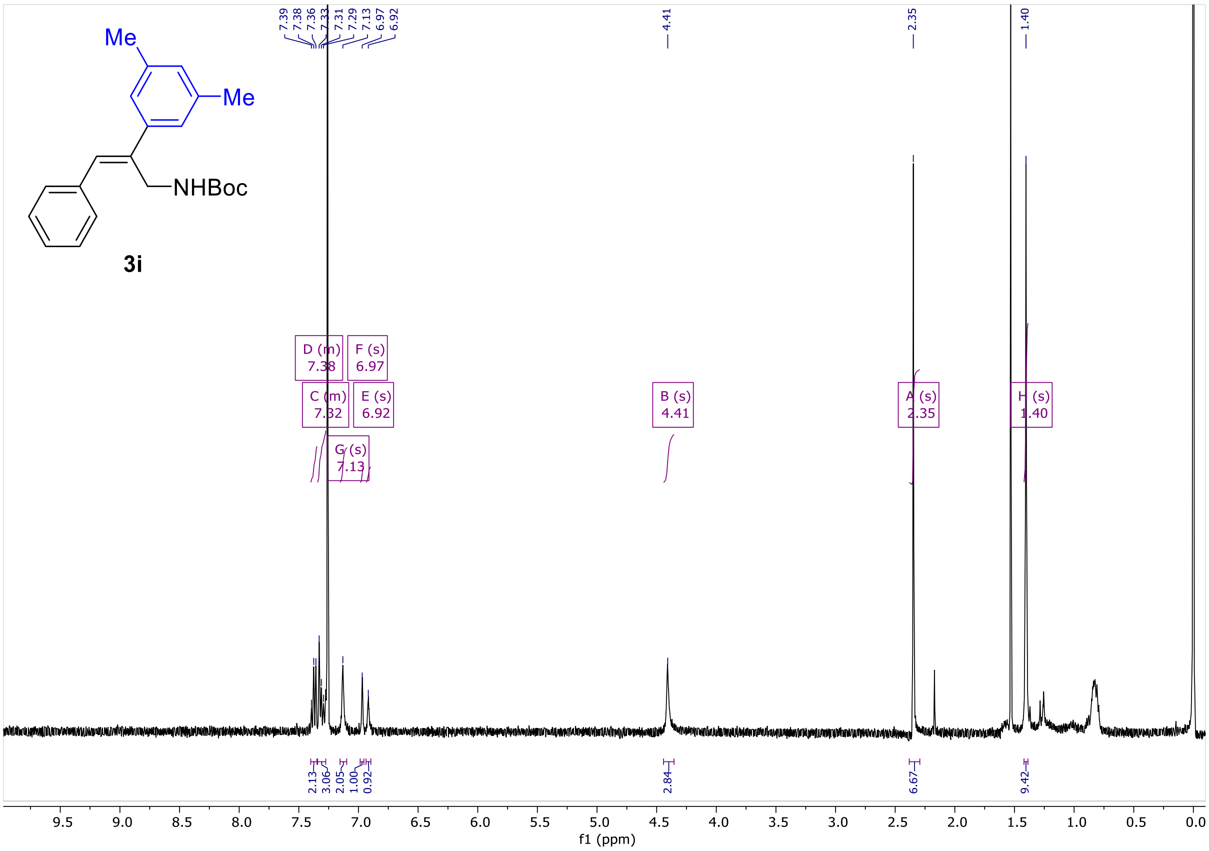

^{13}C-NMR (101 MHz, $CDCl_3$):

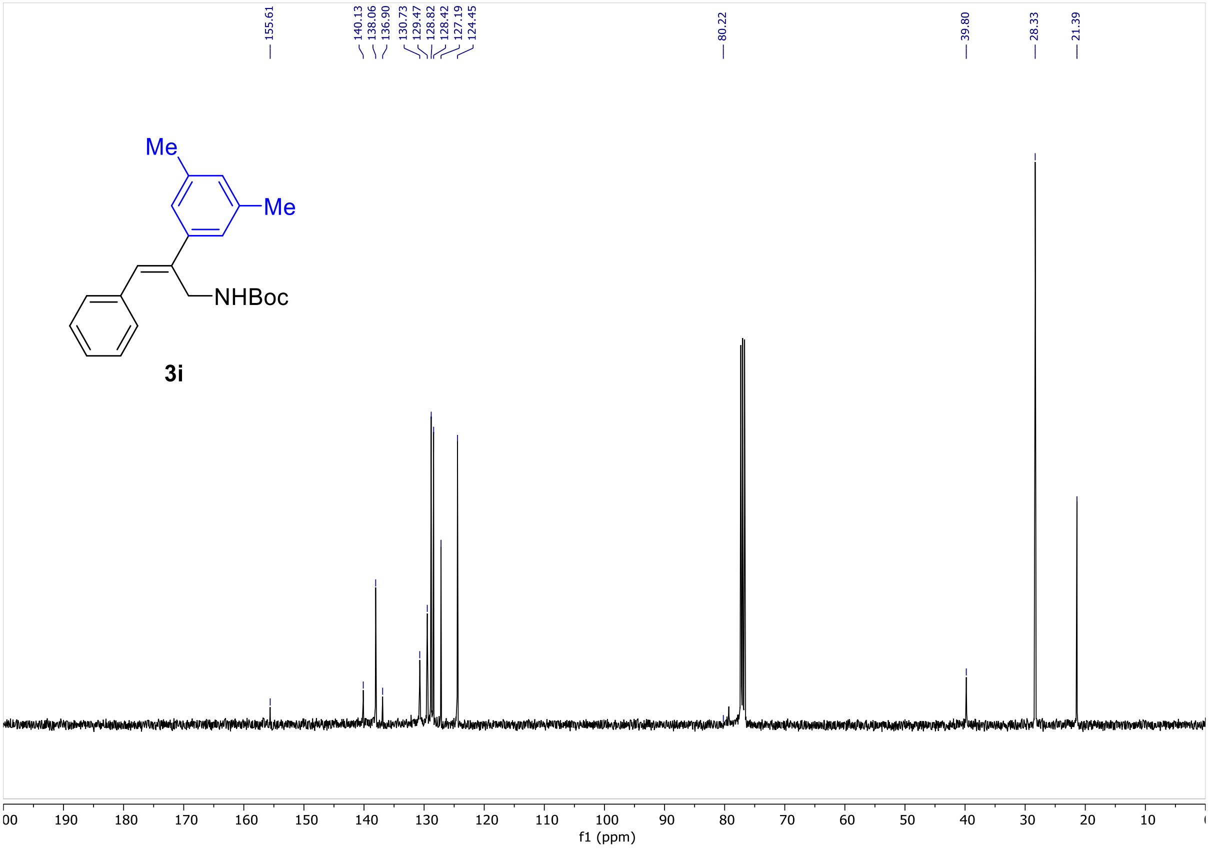

^{1}H-NMR (400 MHz, $CDCl_3$):

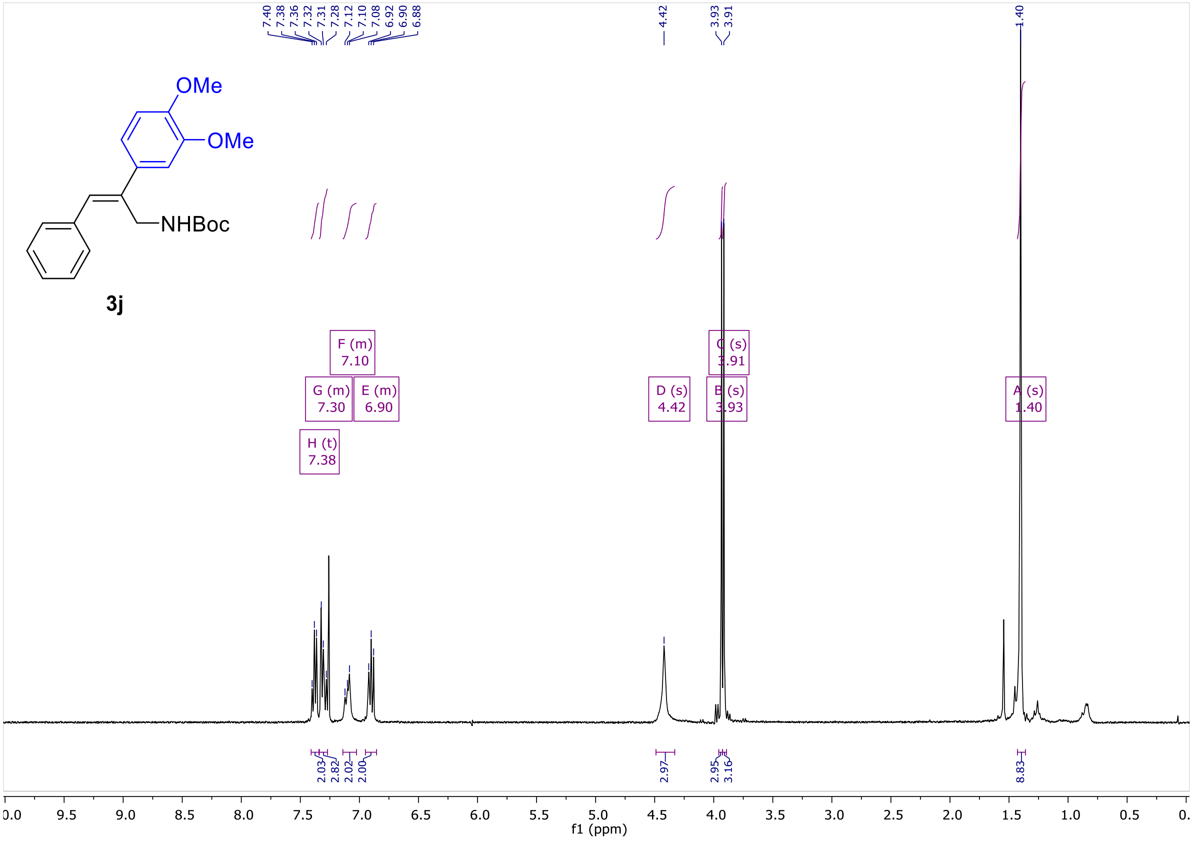

^{13}C-NMR (101 MHz, $CDCl_3$):

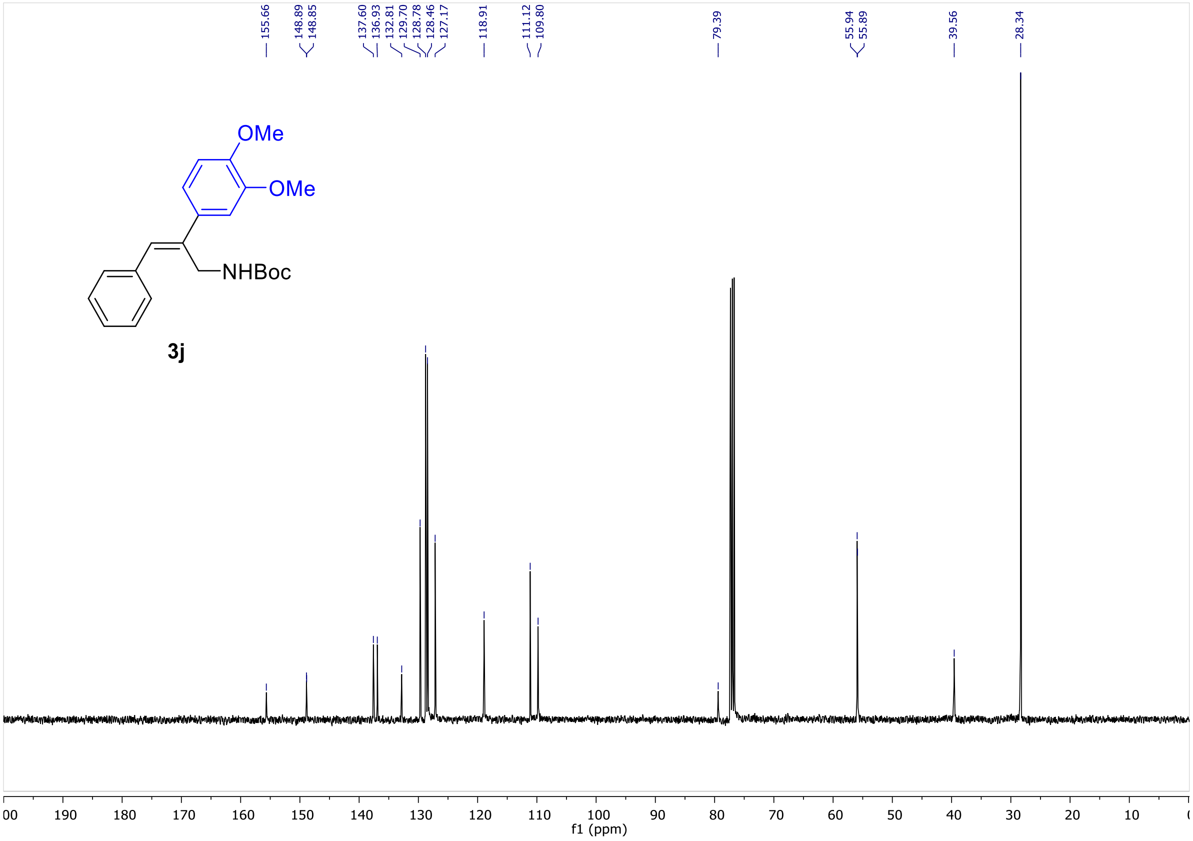

^{1}H-NMR (400 MHz, $CDCl_3$):

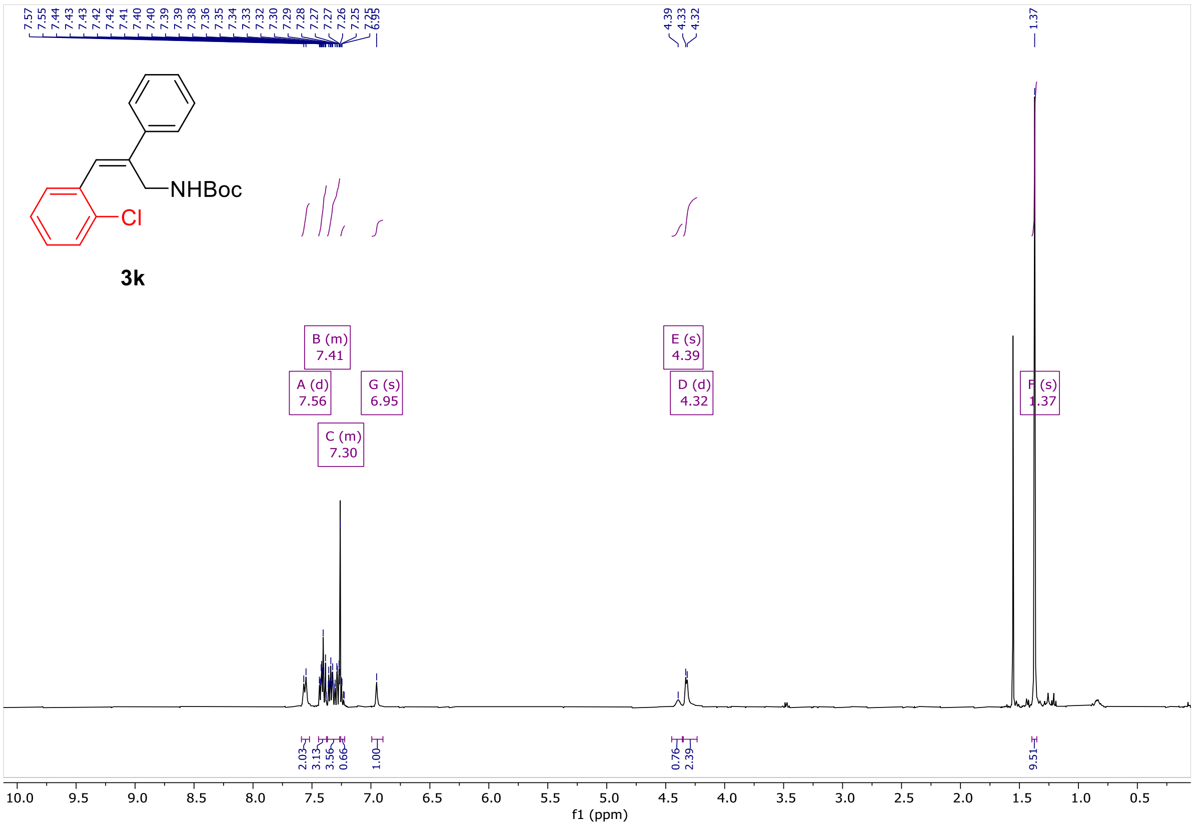

^{1}H-NMR (400 MHz, $CDCl_3$):

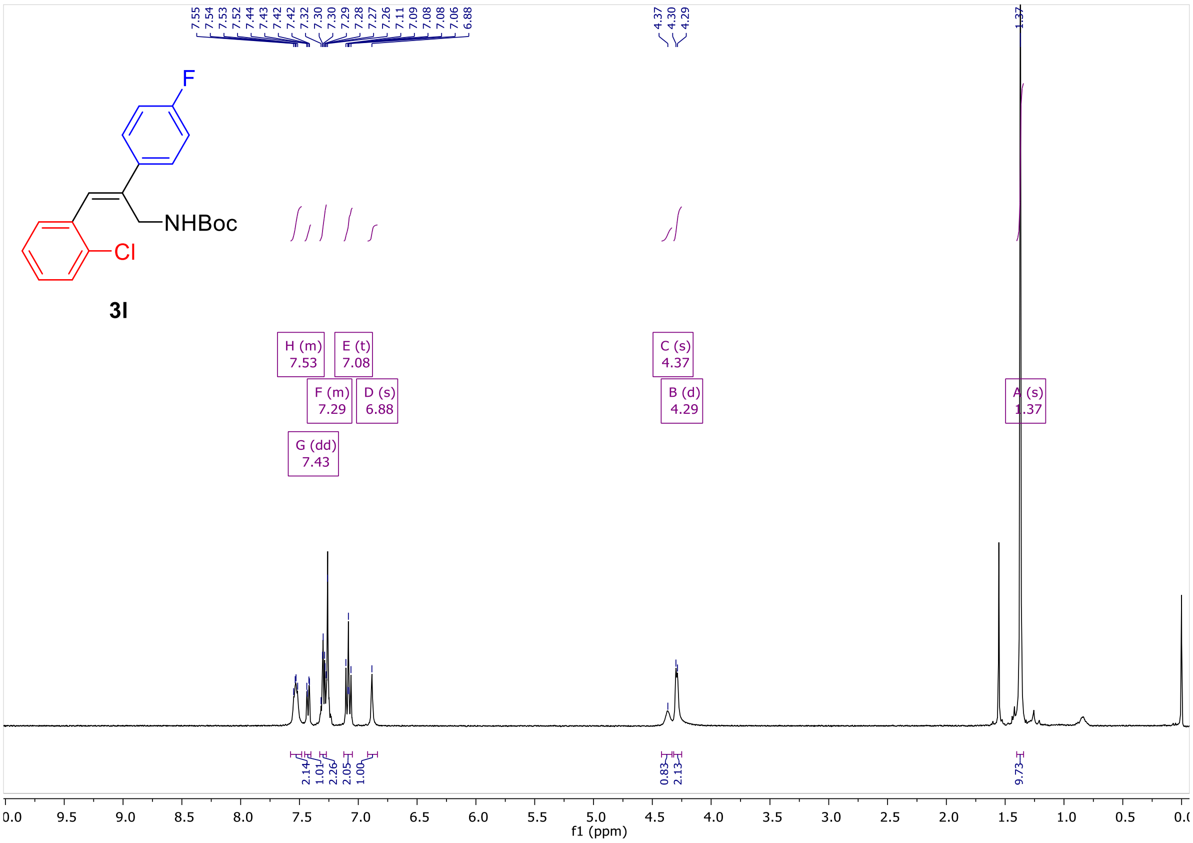

^{13}C-NMR (101 MHz, $CDCl_3$):

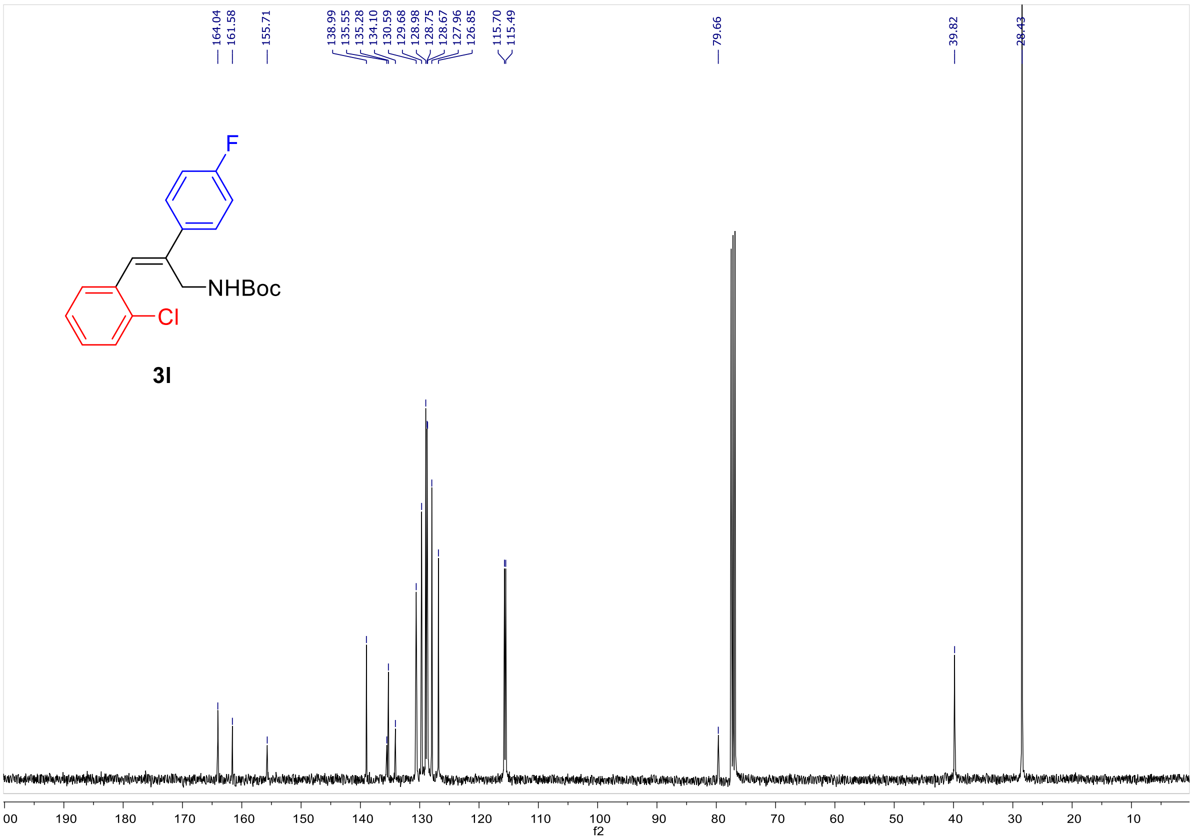

^{1}H-NMR (400 MHz, $CDCl_3$):

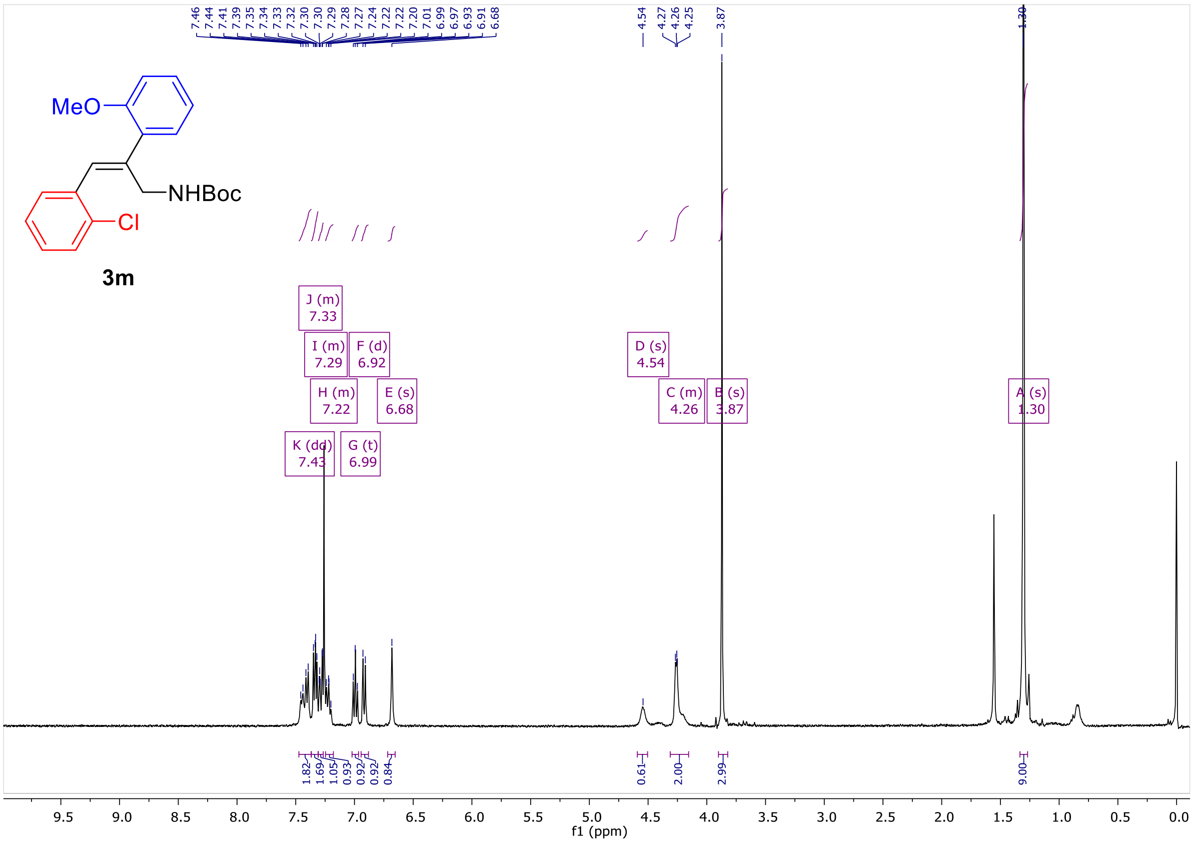

^{13}C-NMR (101 MHz, $CDCl_3$):

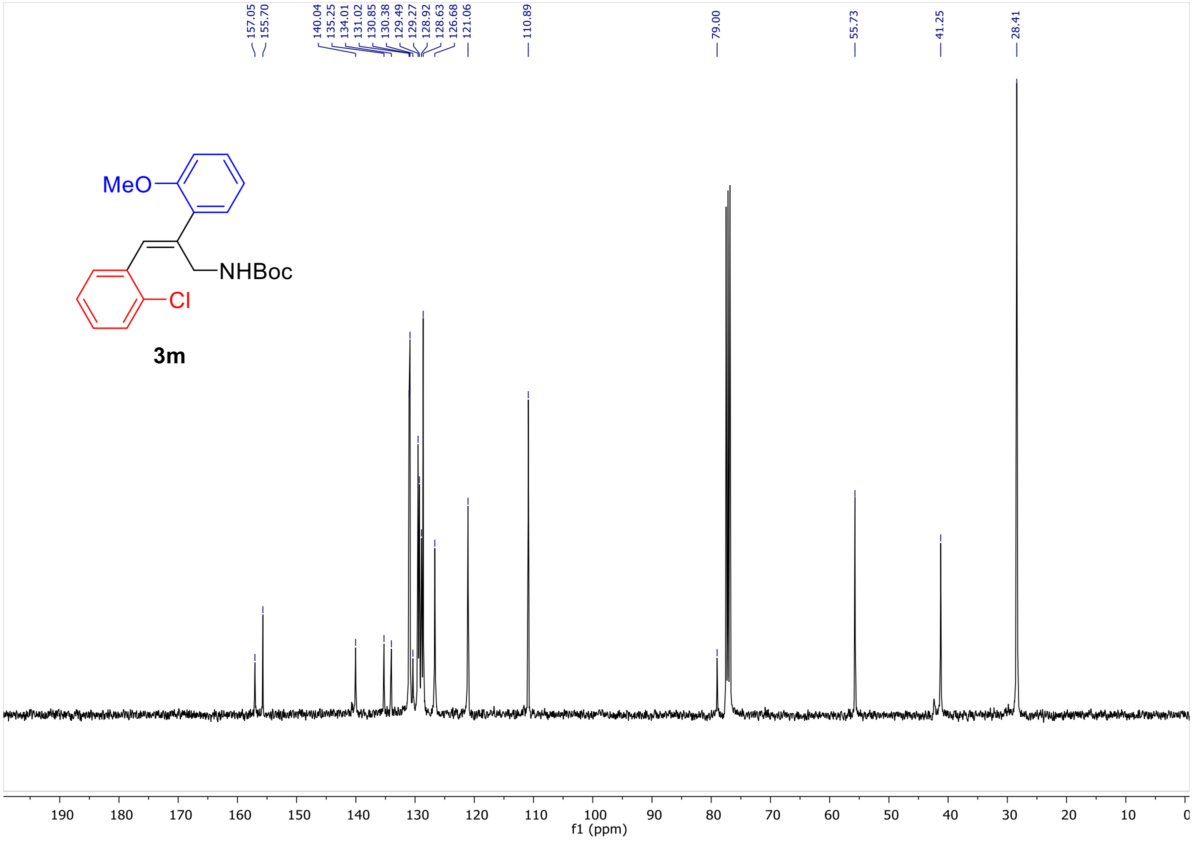

^{1}H-NMR (400 MHz, $CDCl_3$):

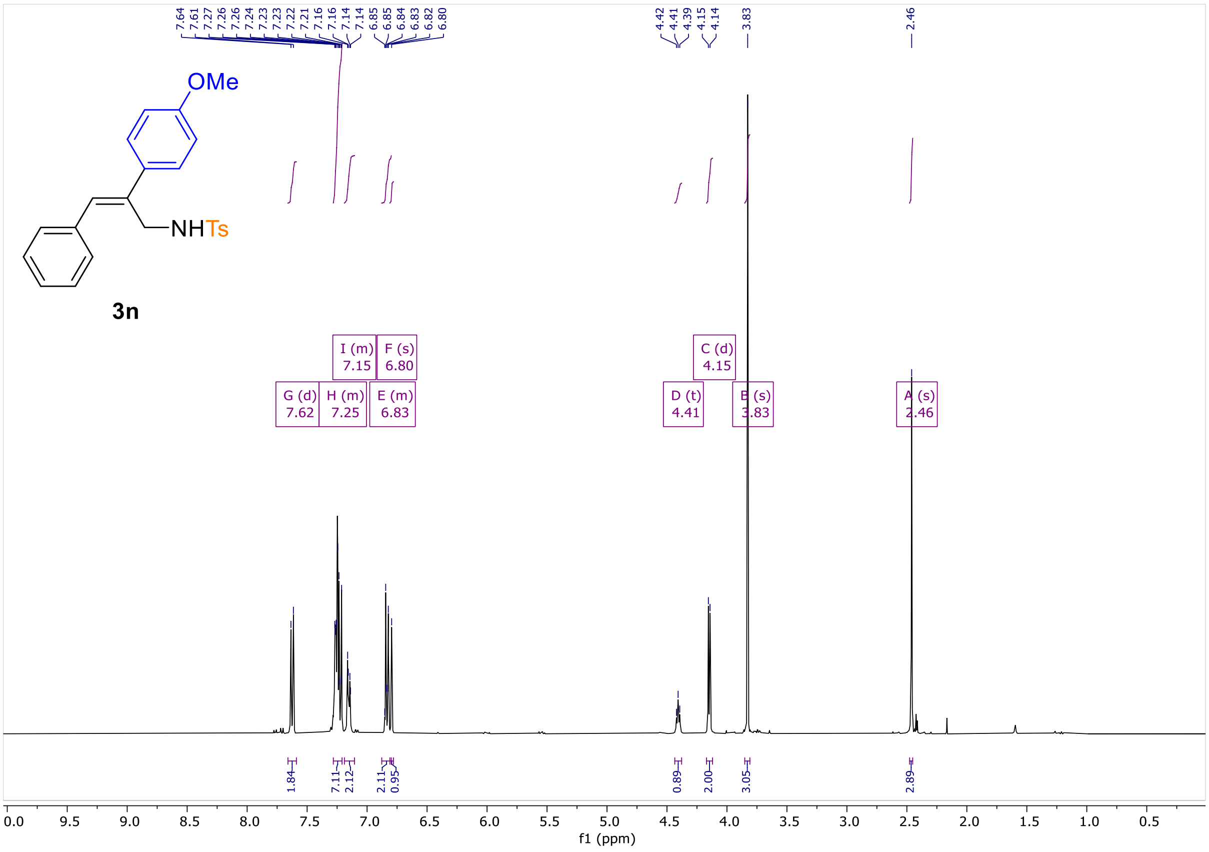

^{13}C-NMR (101 MHz, $CDCl_3$):

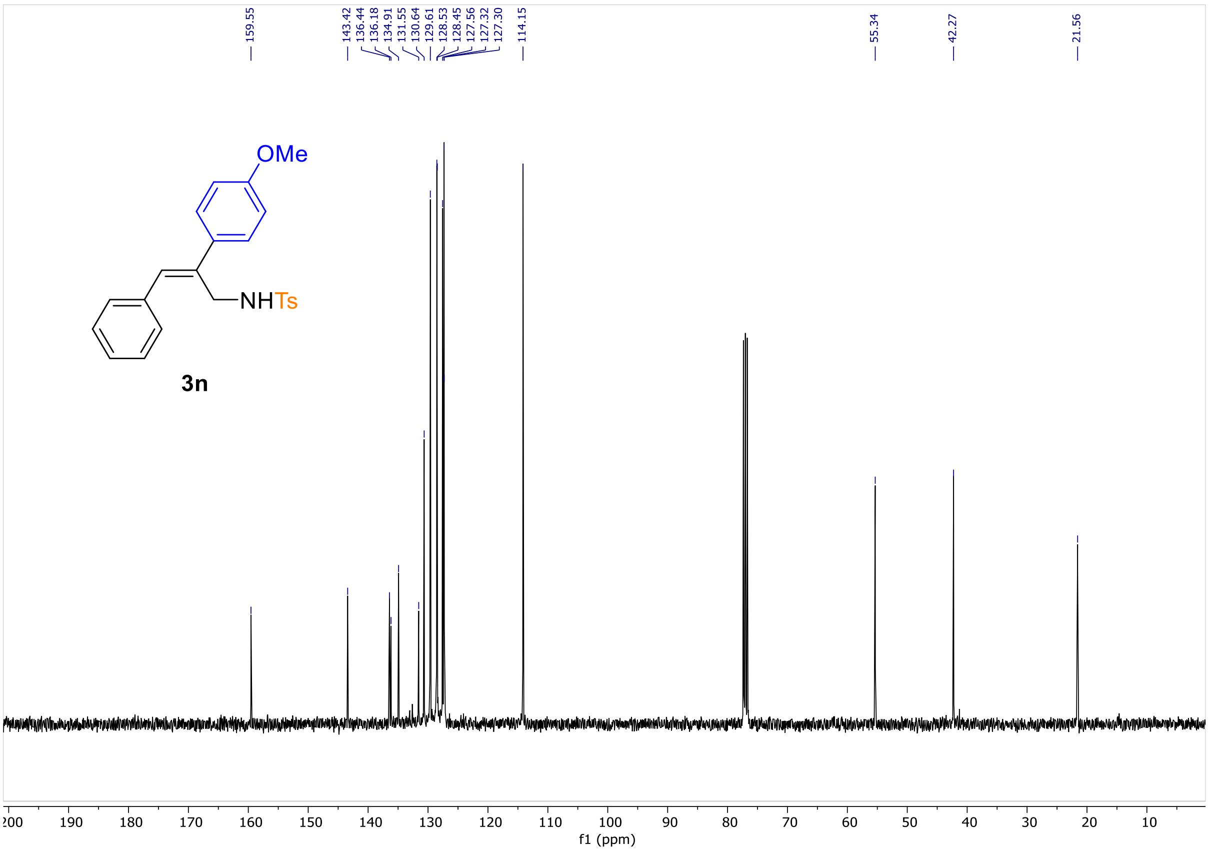

^{1}H-NMR (400 MHz, $CDCl_3$):

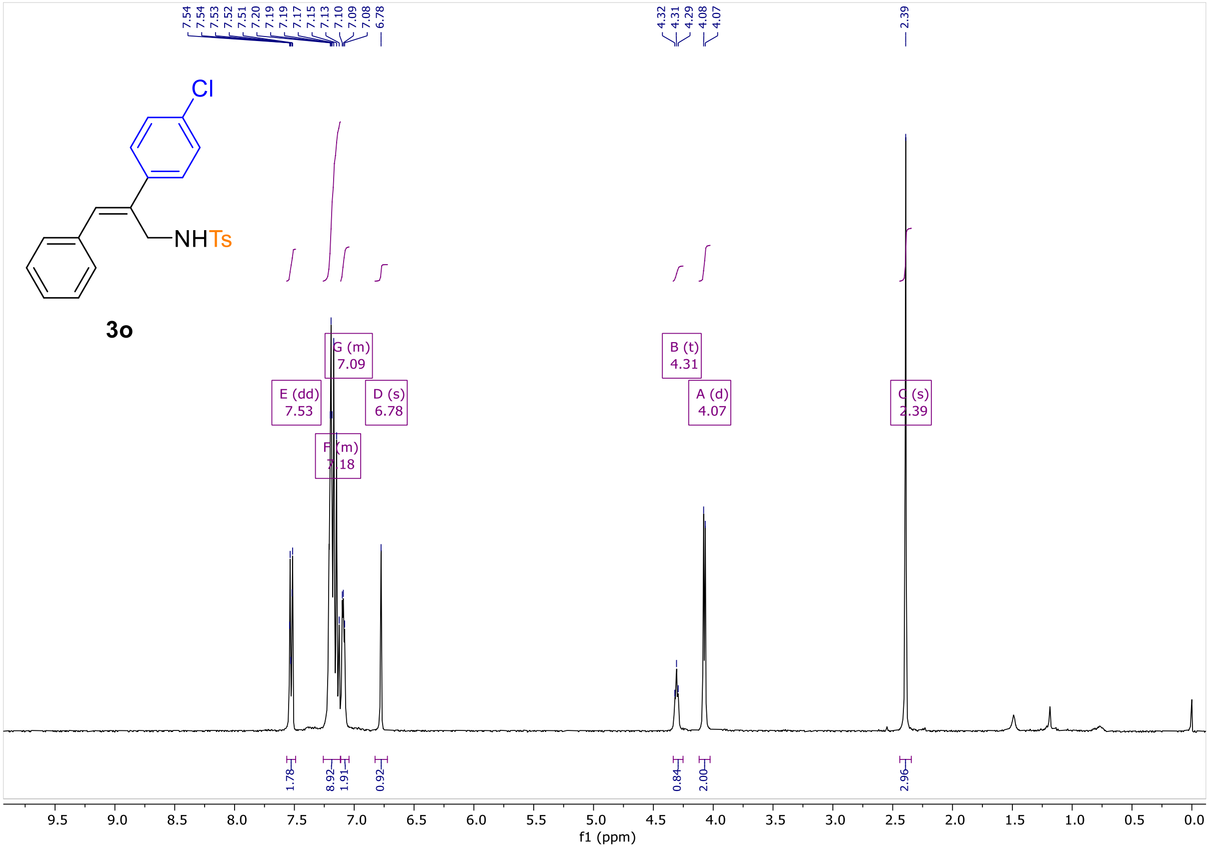

^{13}C-NMR (101 MHz, $CDCl_3$):

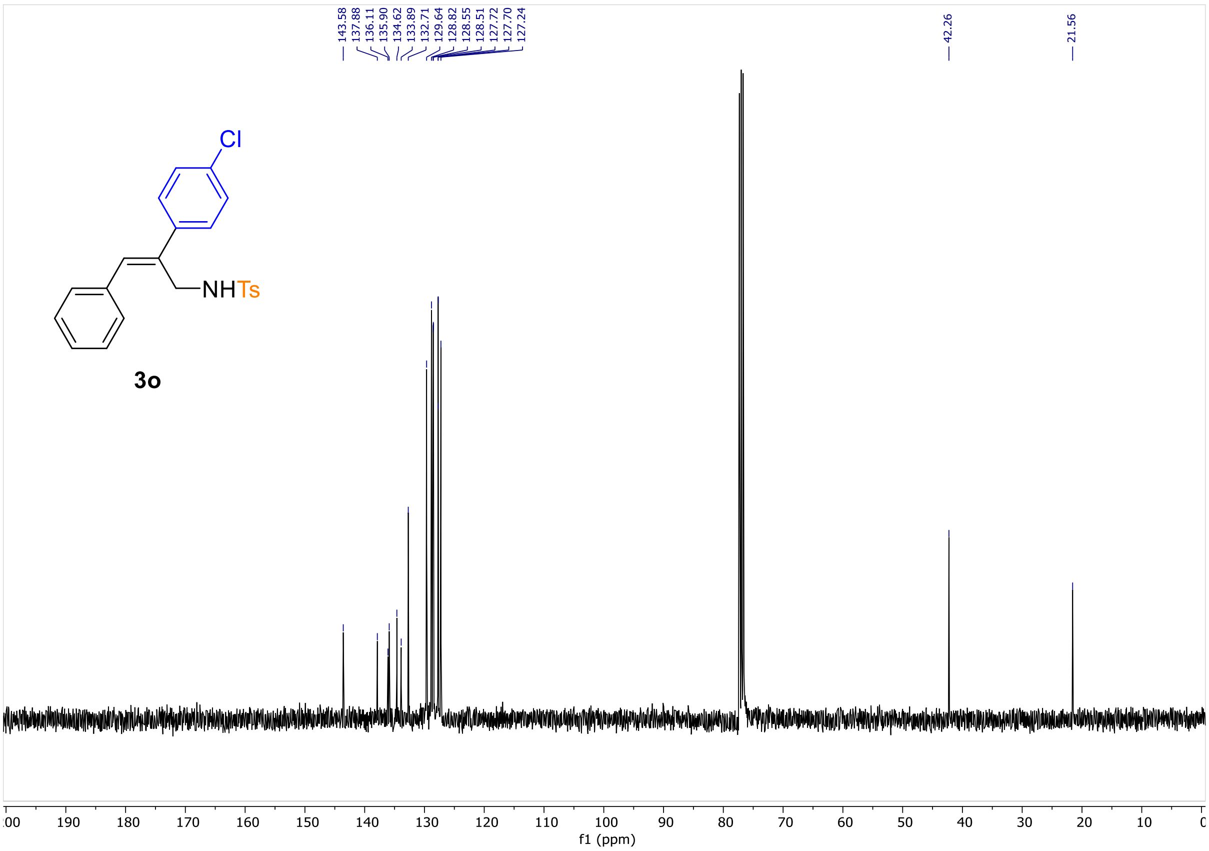

^{1}H-NMR (400 MHz, $CDCl_3$):

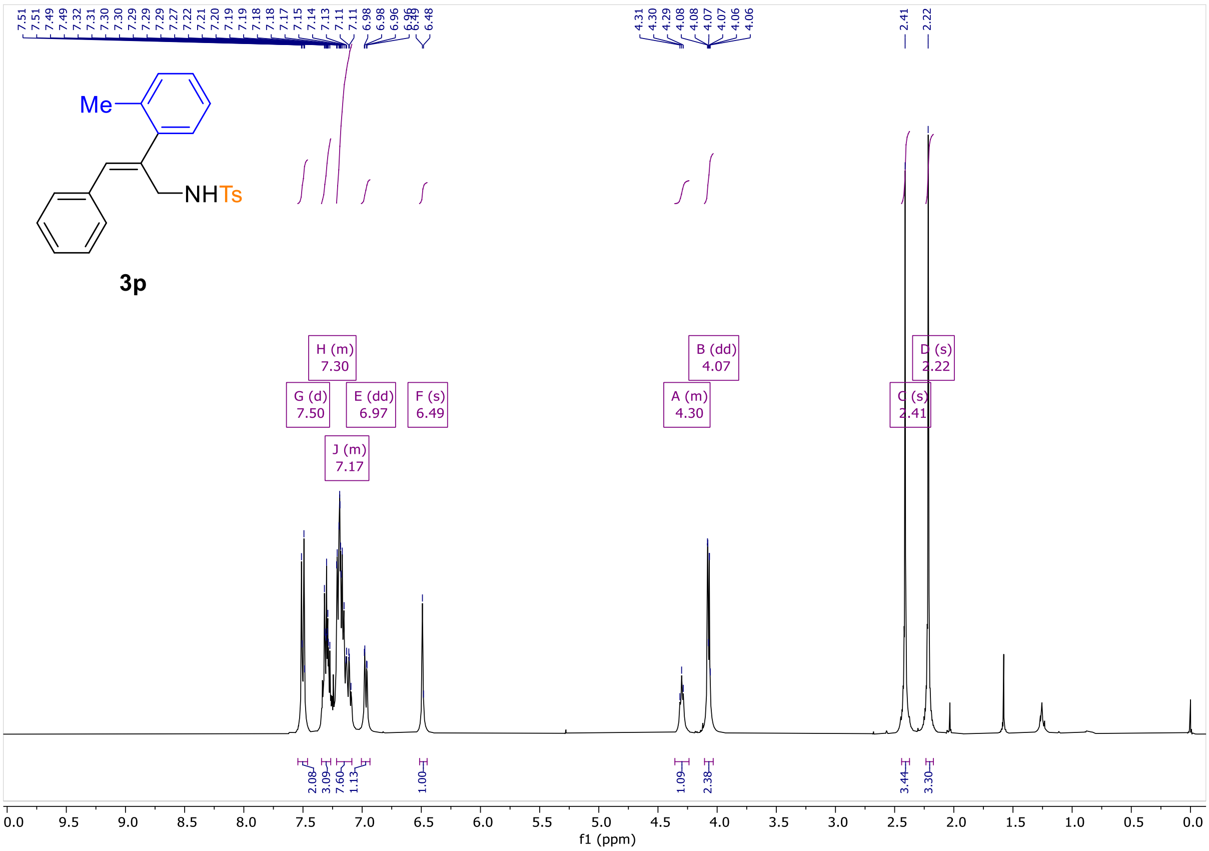

^{13}C-NMR (101 MHz, $CDCl_3$):

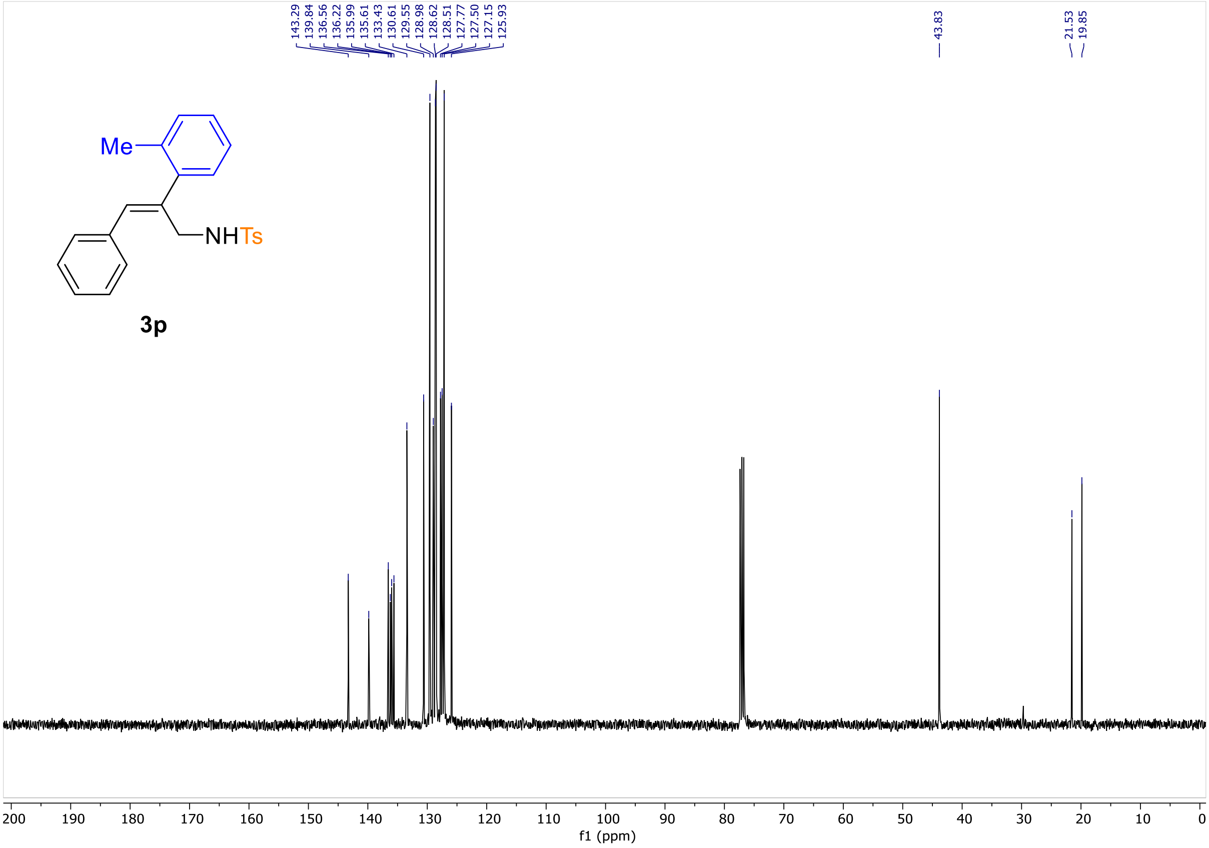

^{1}H-NMR (400 MHz, D_2O):

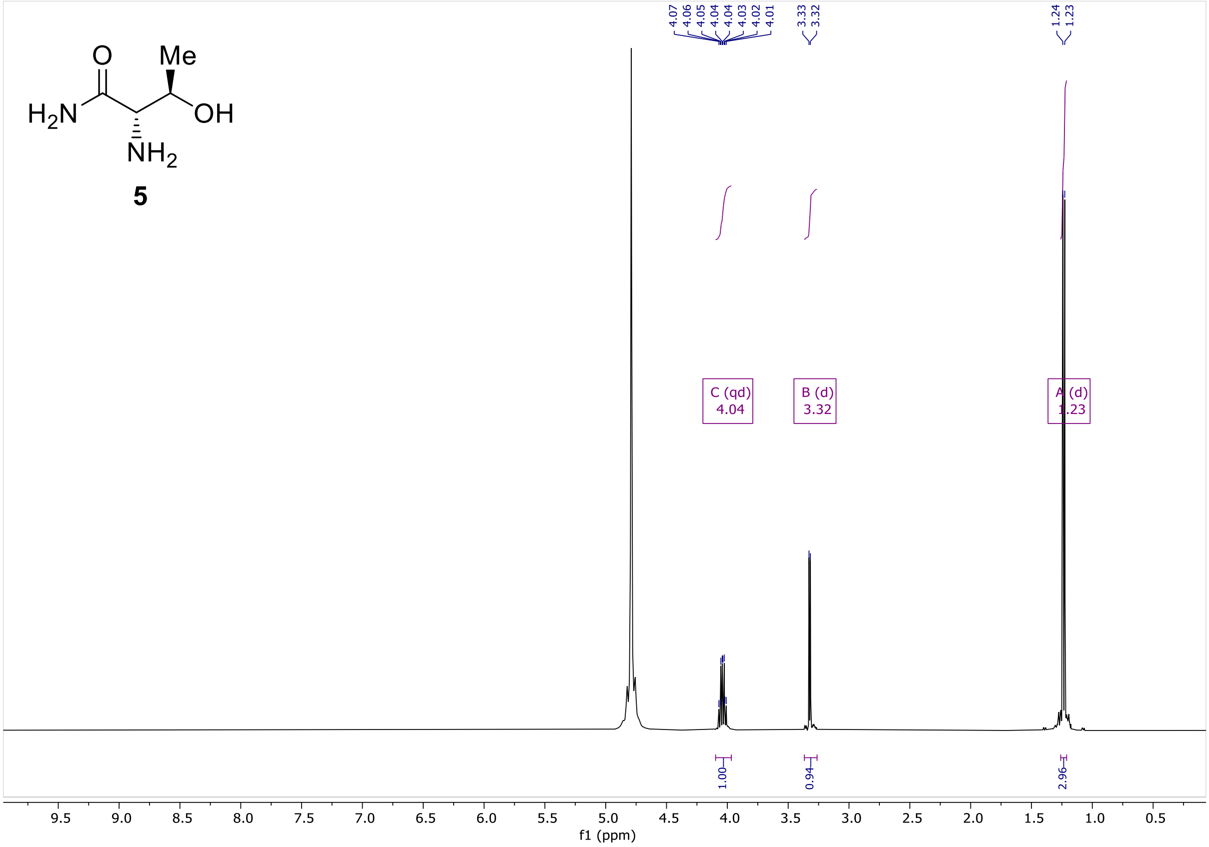

^{13}C-NMR (101 MHz, D_2O):

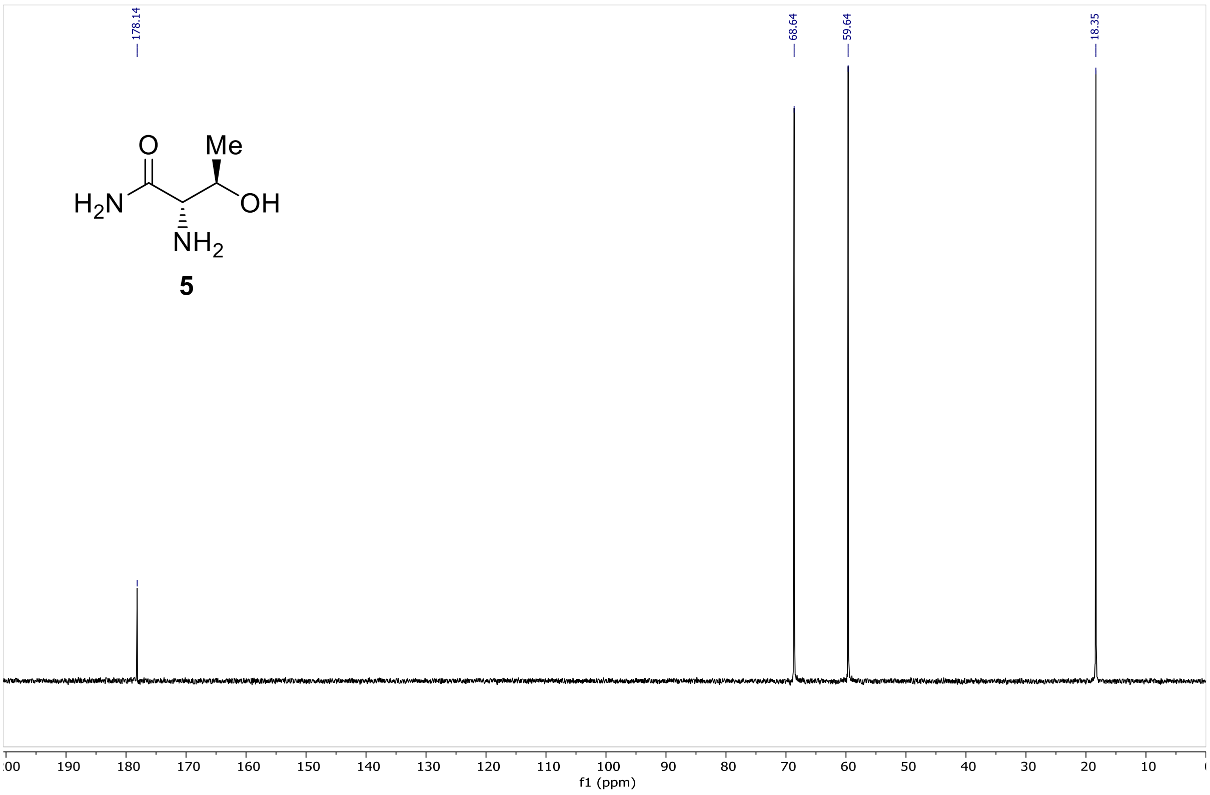

^{1}H-NMR (400 MHz, D_2O):

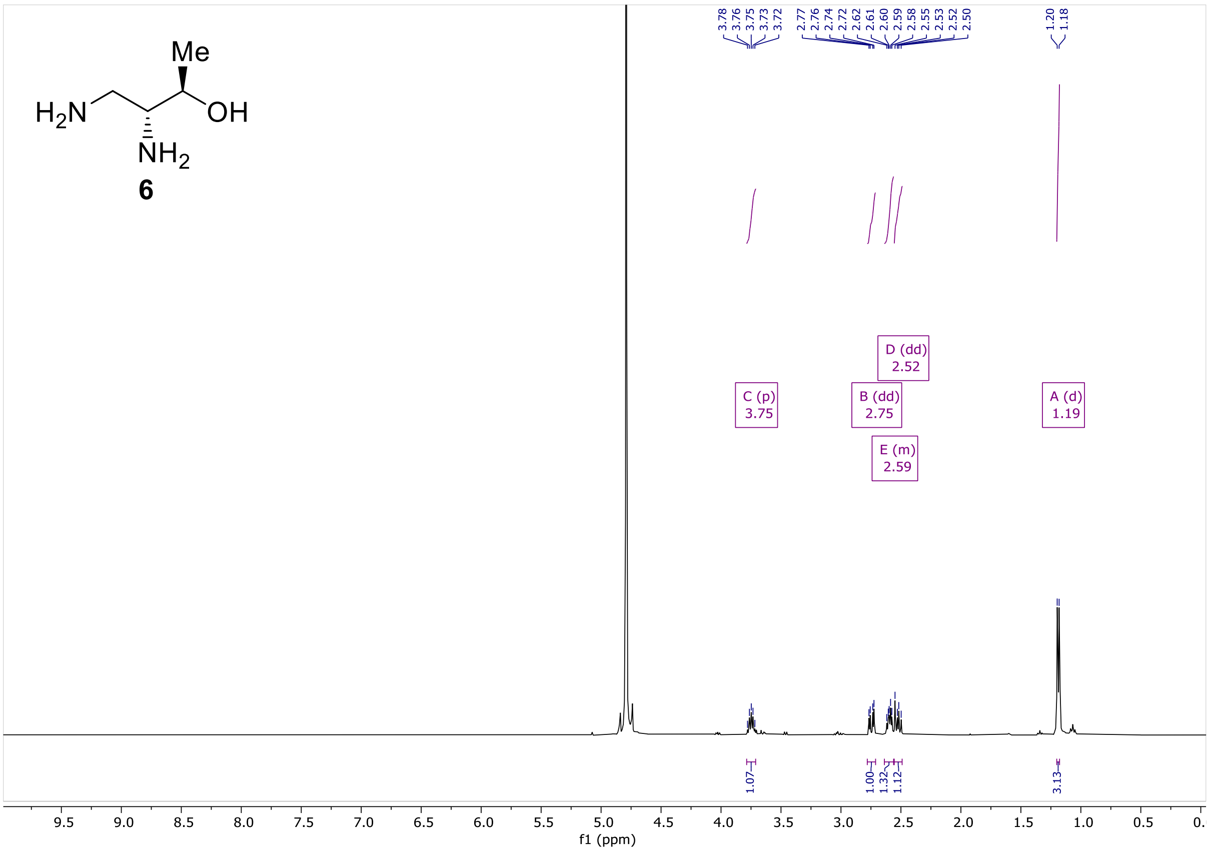

^{13}C-NMR (101 MHz, D_2O):

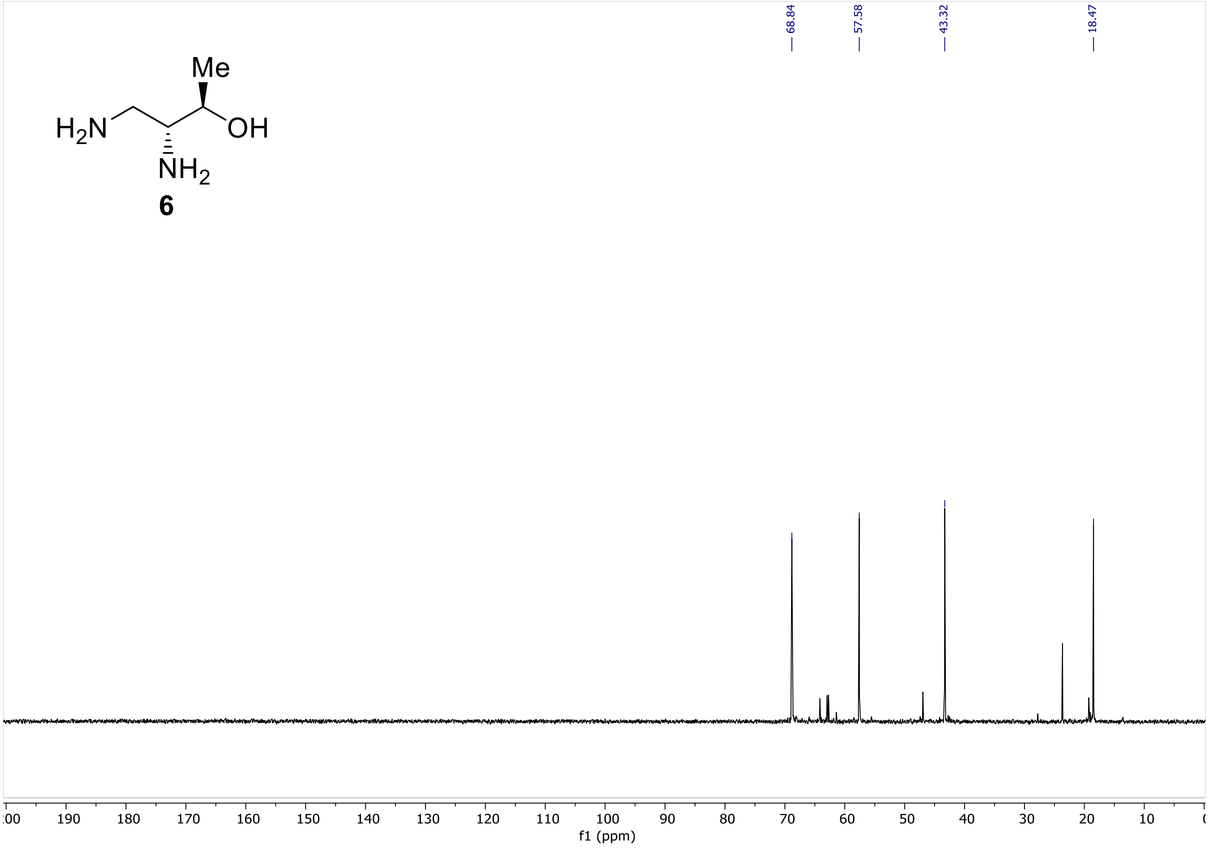

^{1}H-NMR (400 MHz, $CDCl_3$):

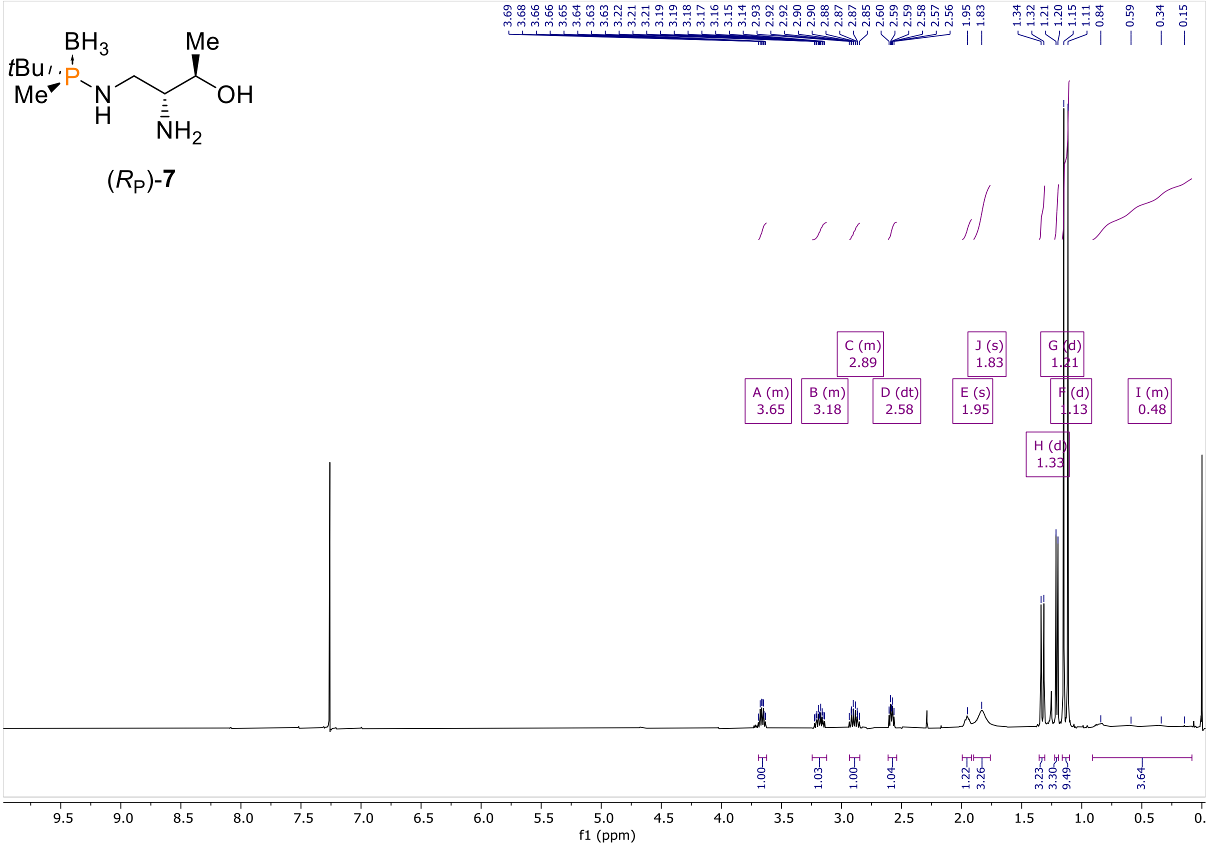

^{13}C-NMR (101 MHz, $CDCl_3$):

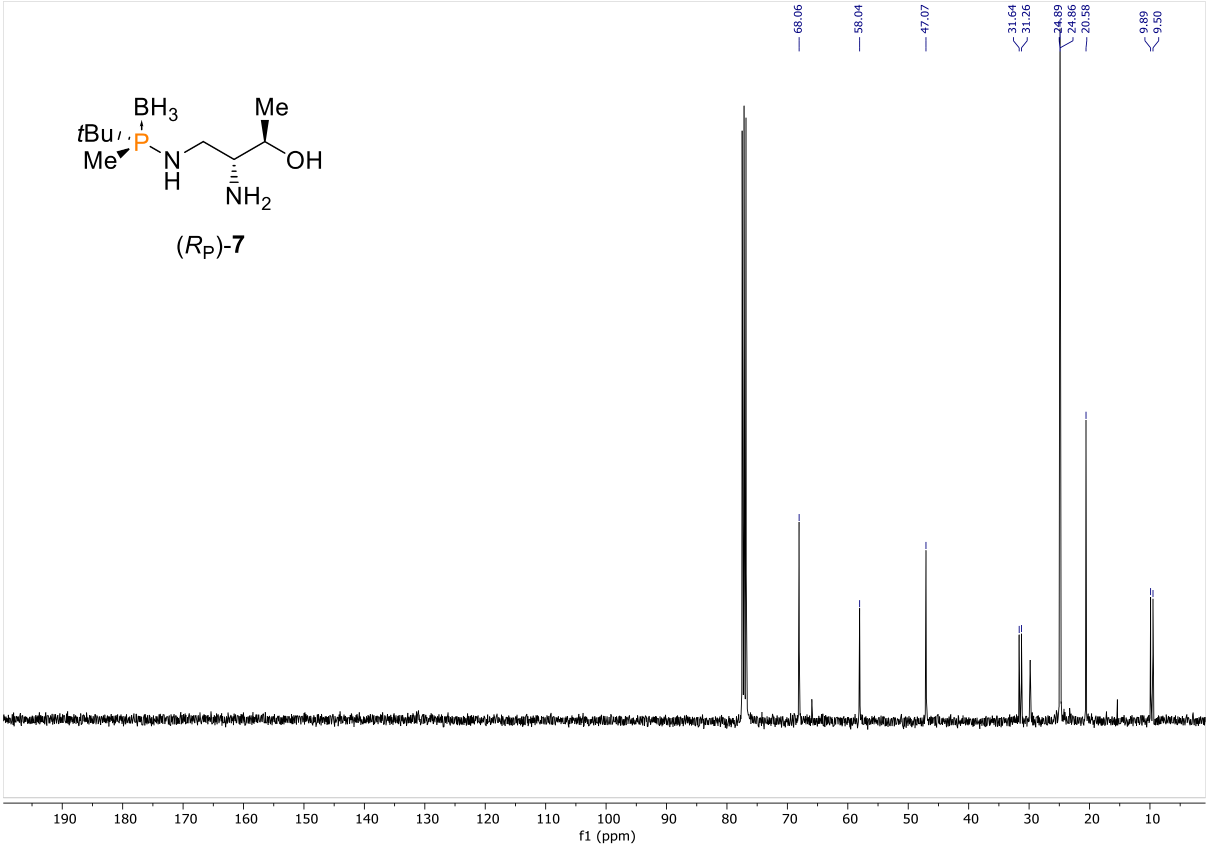

^{31}P-NMR (162 MHz, $CDCl_3$):

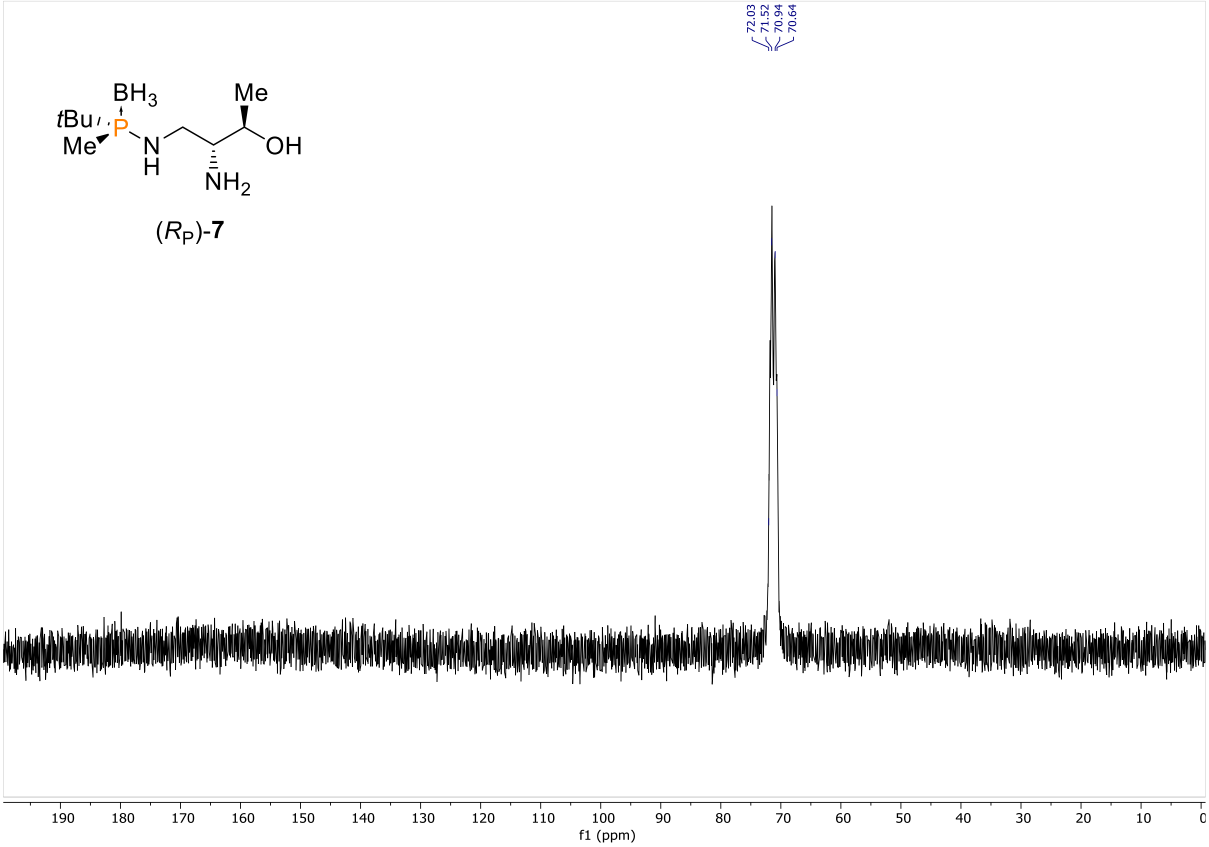

^{1}H-NMR (400 MHz, $CDCl_3$):

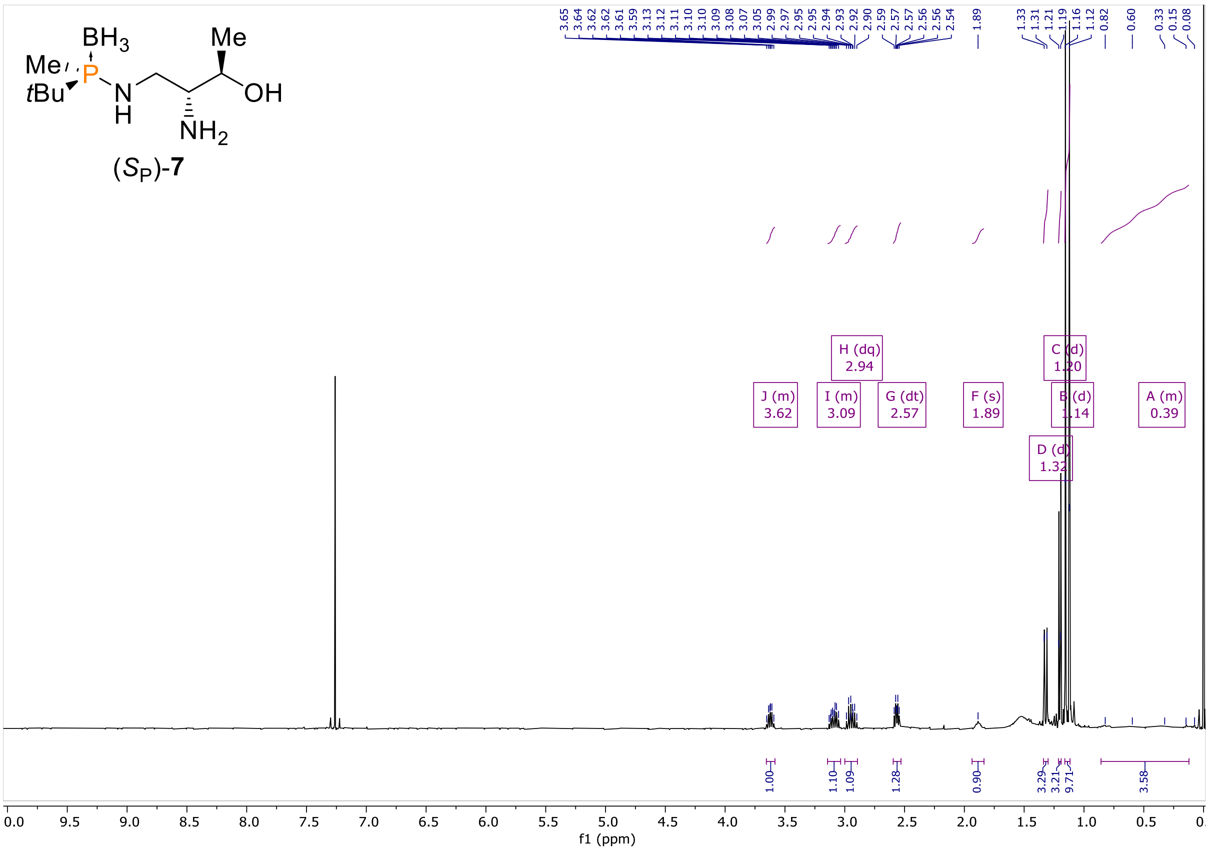

^{13}C-NMR (101 MHz, $CDCl_3$):

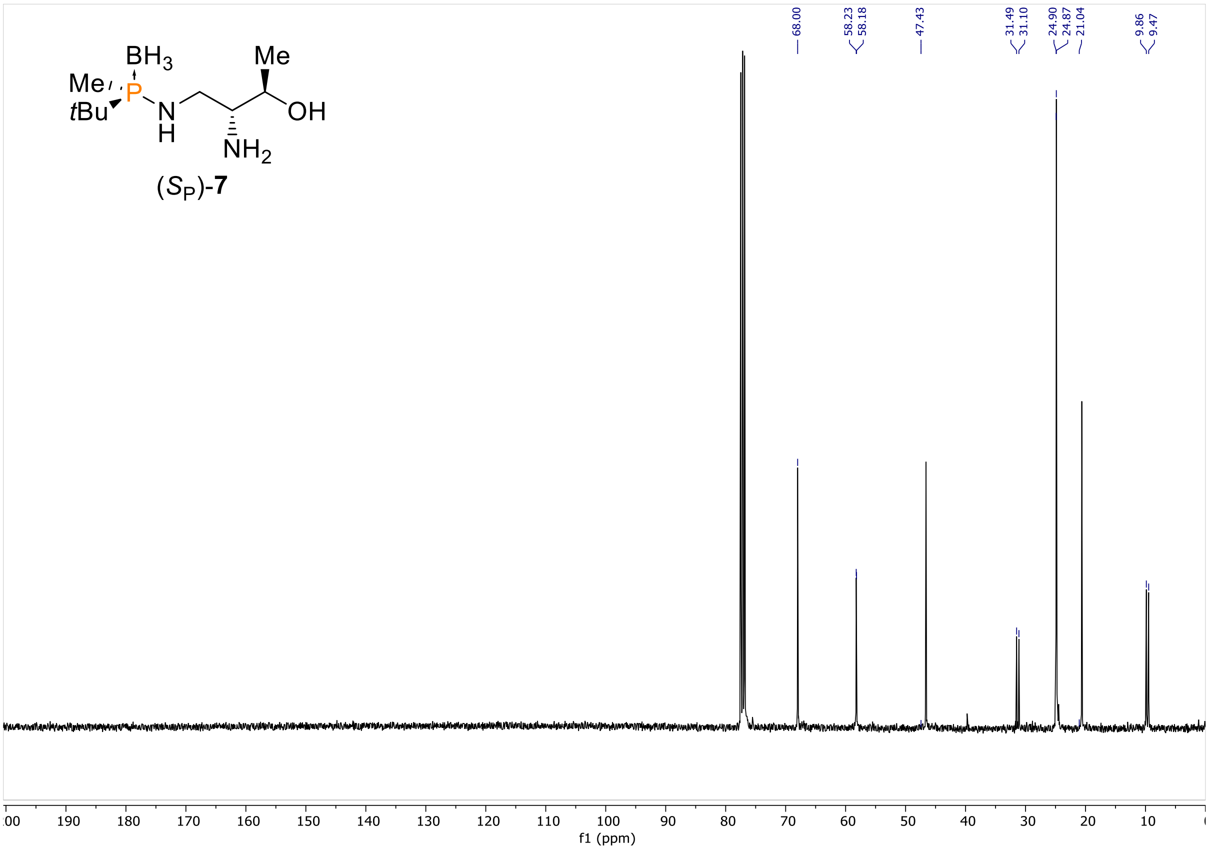

^{31}P-NMR (162 MHz, $CDCl_3$):

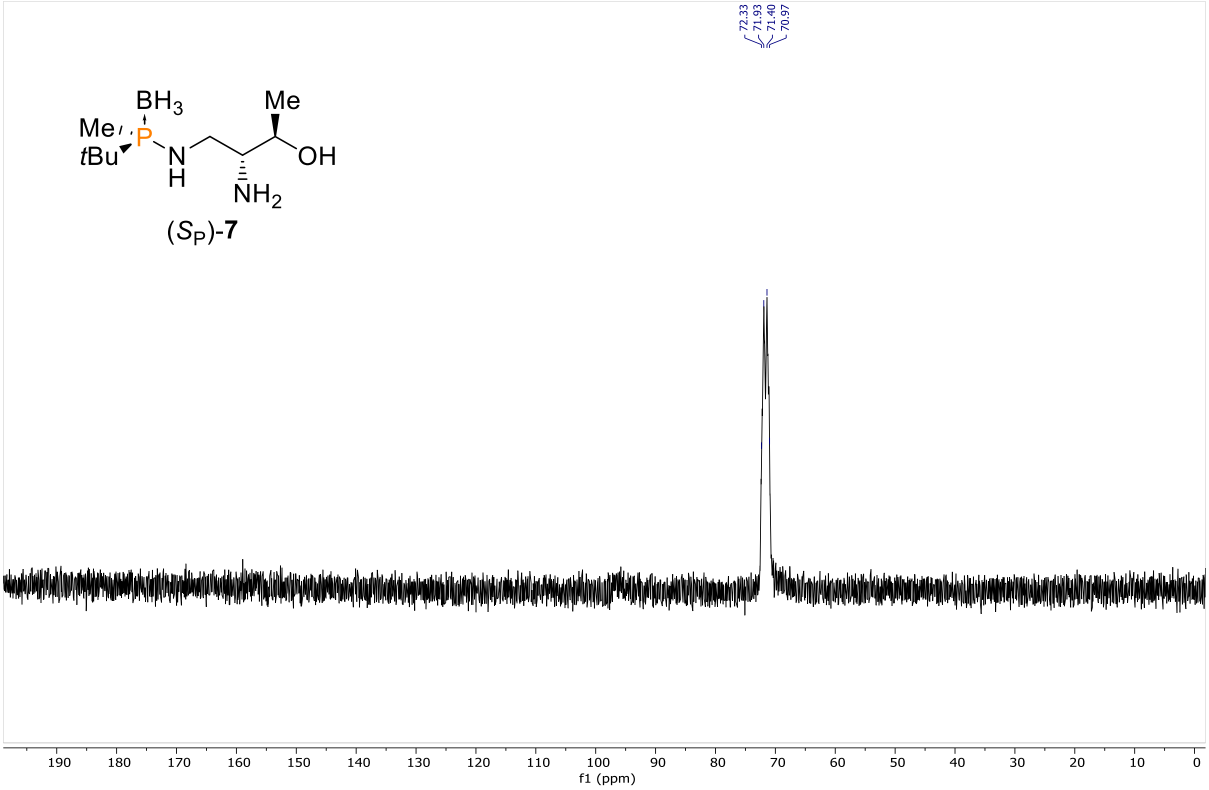

^{1}H-NMR (400 MHz, CDCl$_3$):

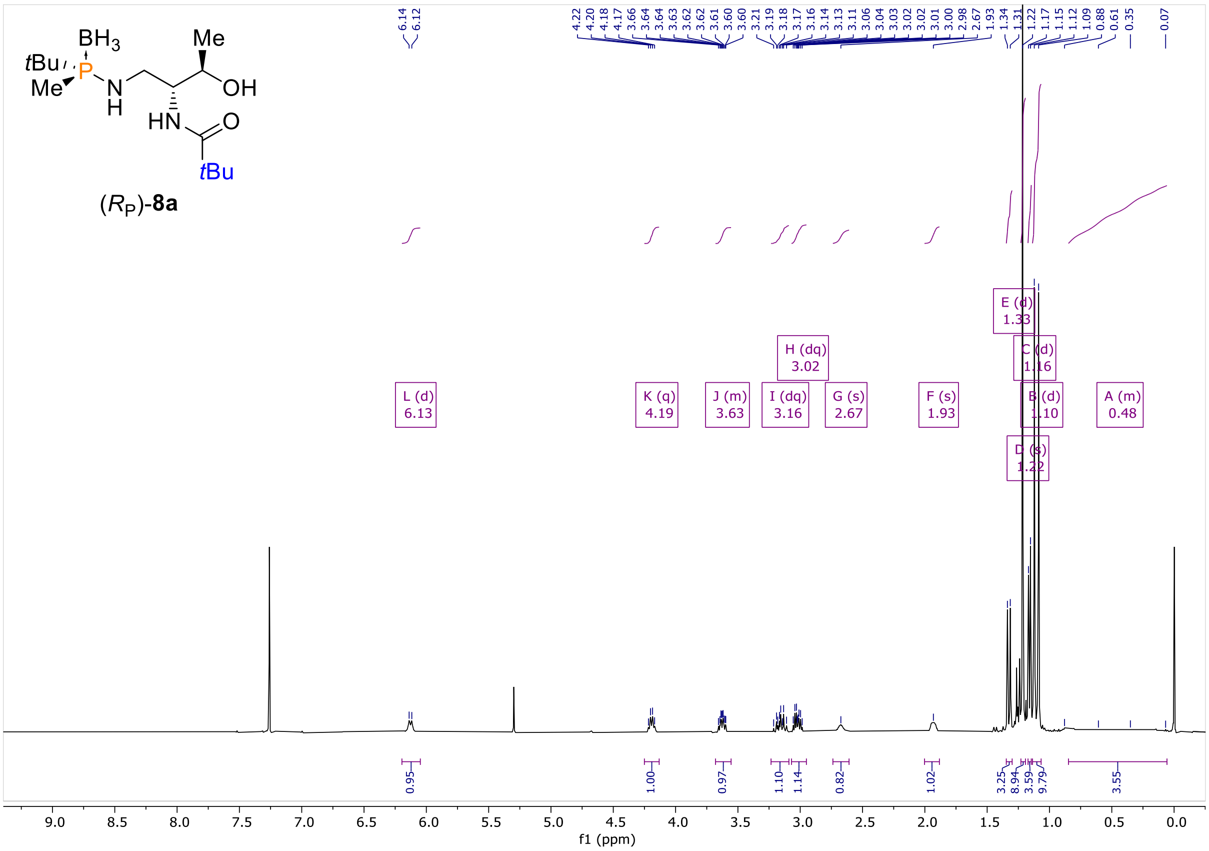

^{1}H-NMR (400 MHz, CDCl$_3$):

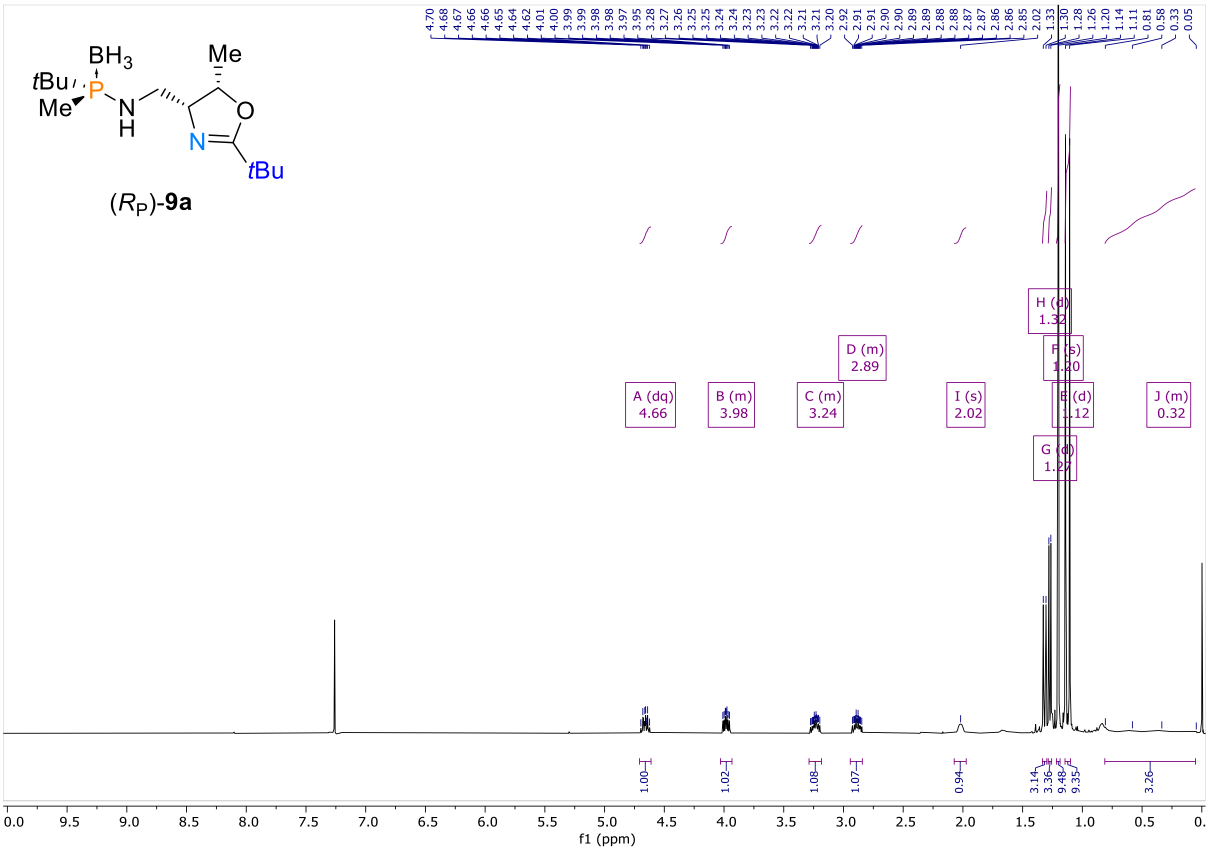

^{13}C-NMR (101 MHz, $CDCl_3$):

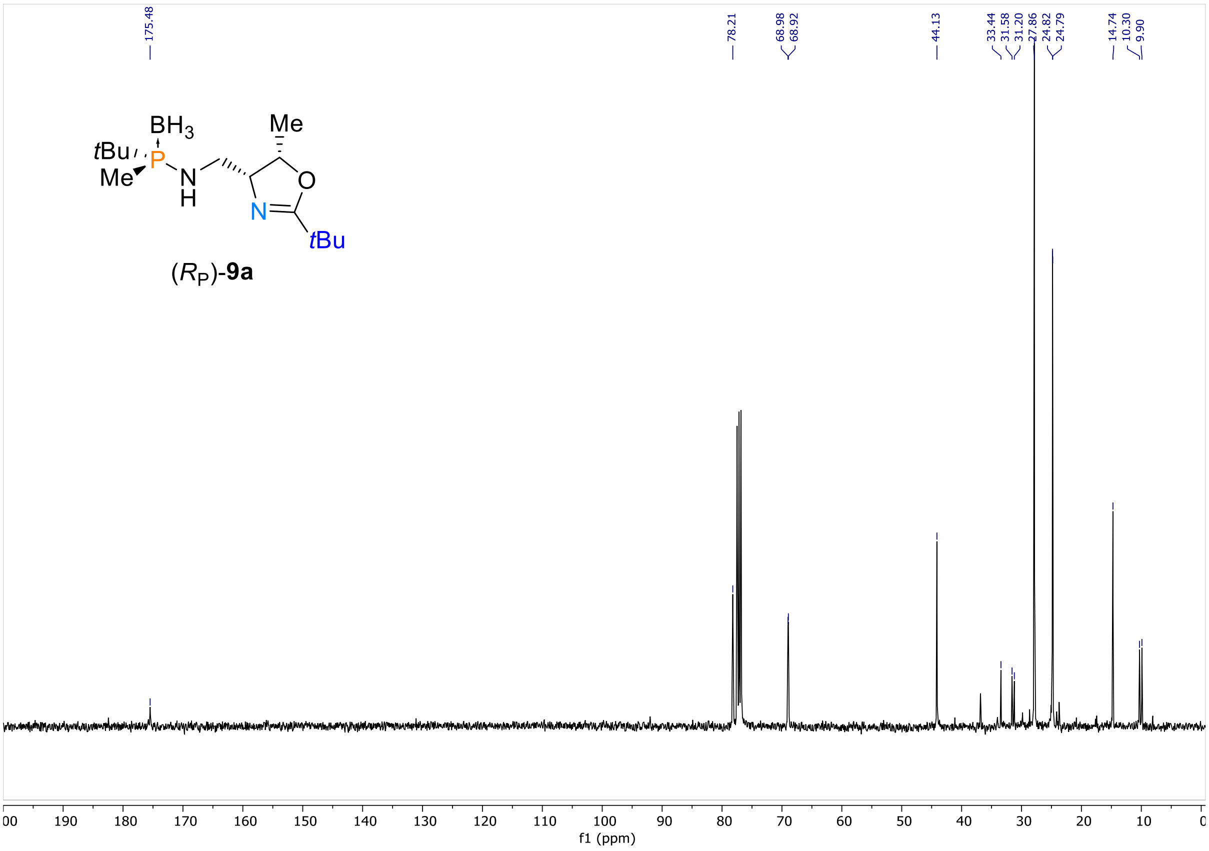

^{31}P-NMR (162 MHz, $CDCl_3$):

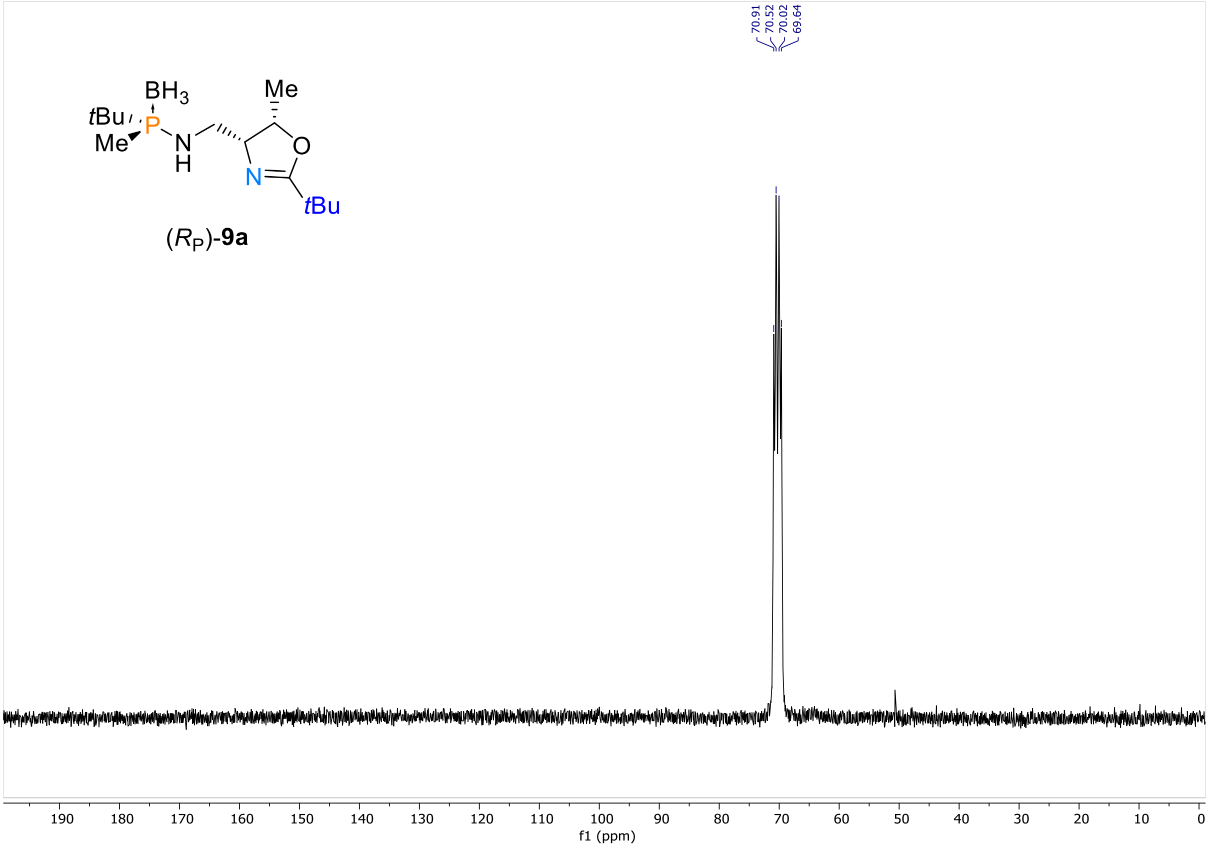

^{1}H-NMR (400 MHz, $CDCl_3$):

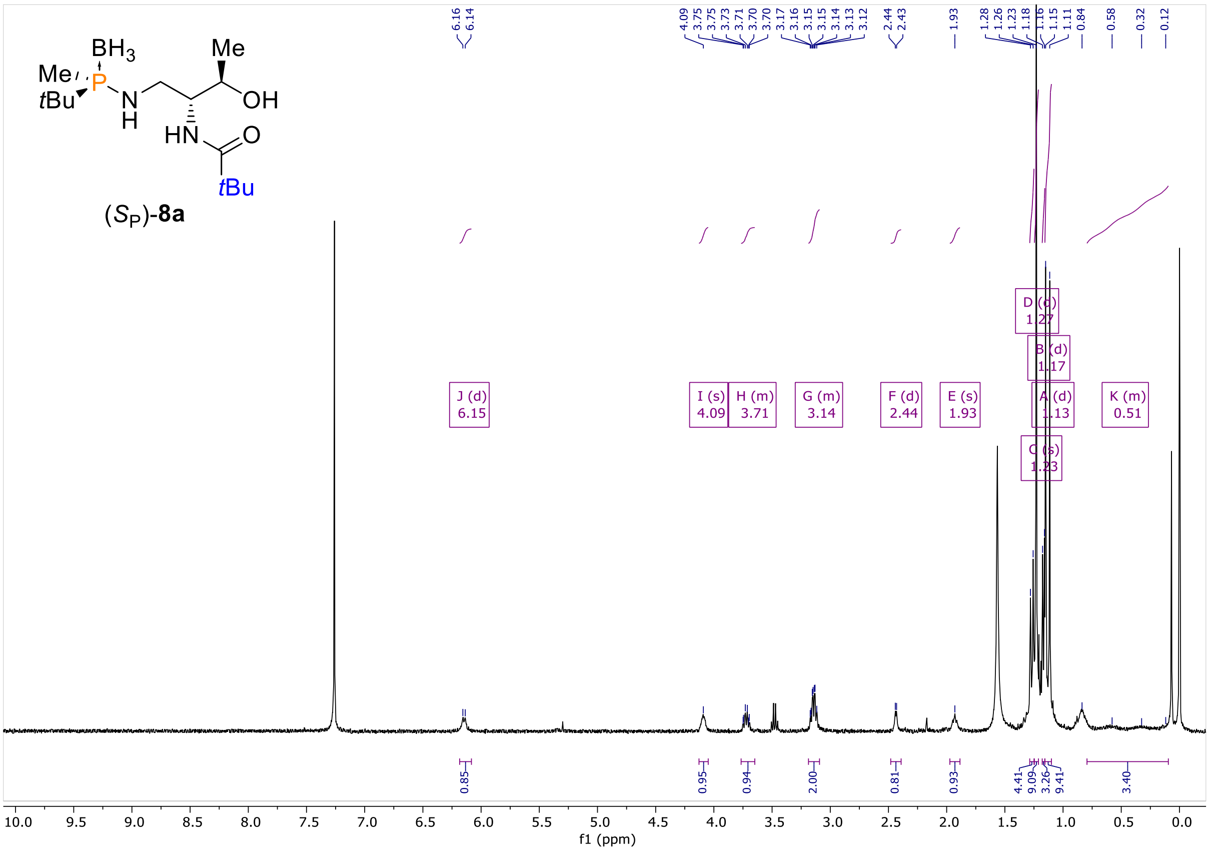

^{1}H-NMR (400 MHz, $CDCl_3$):

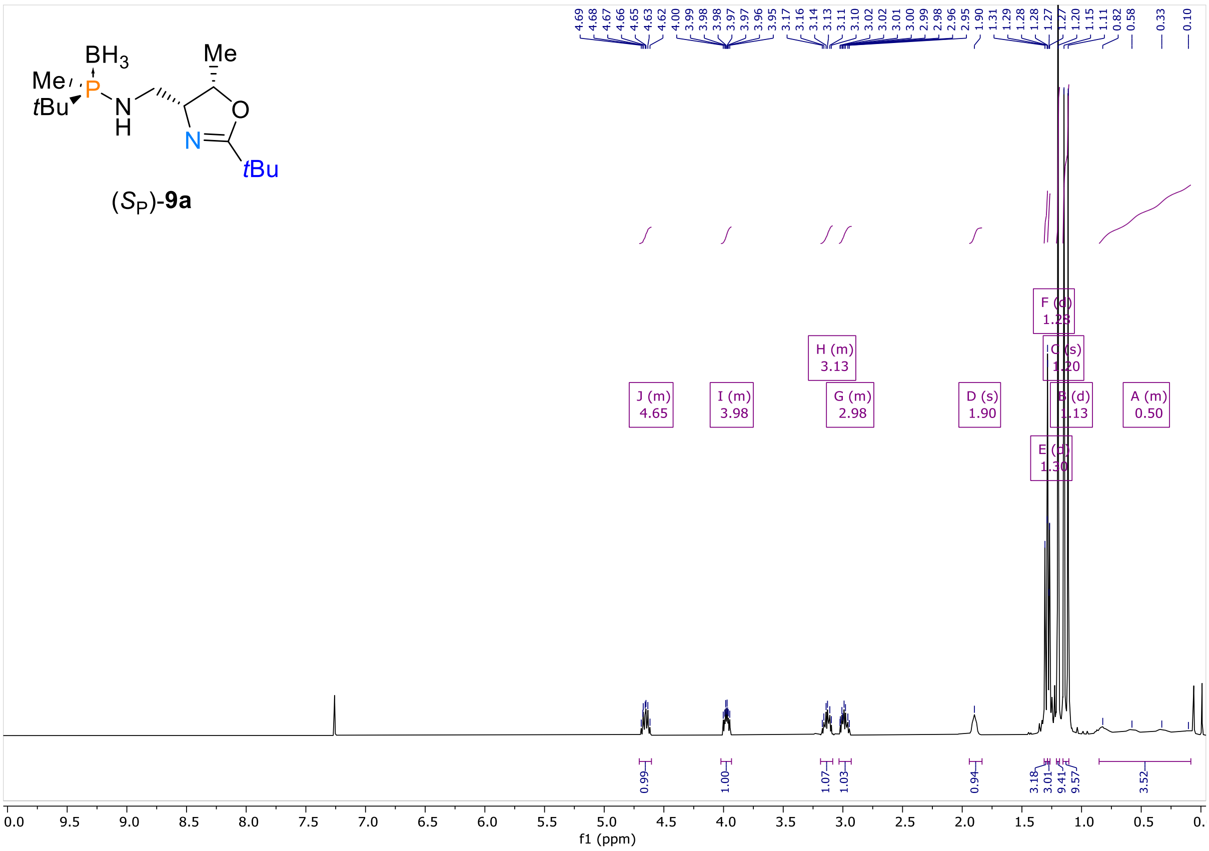

^{13}C-NMR (101 MHz, $CDCl_3$):

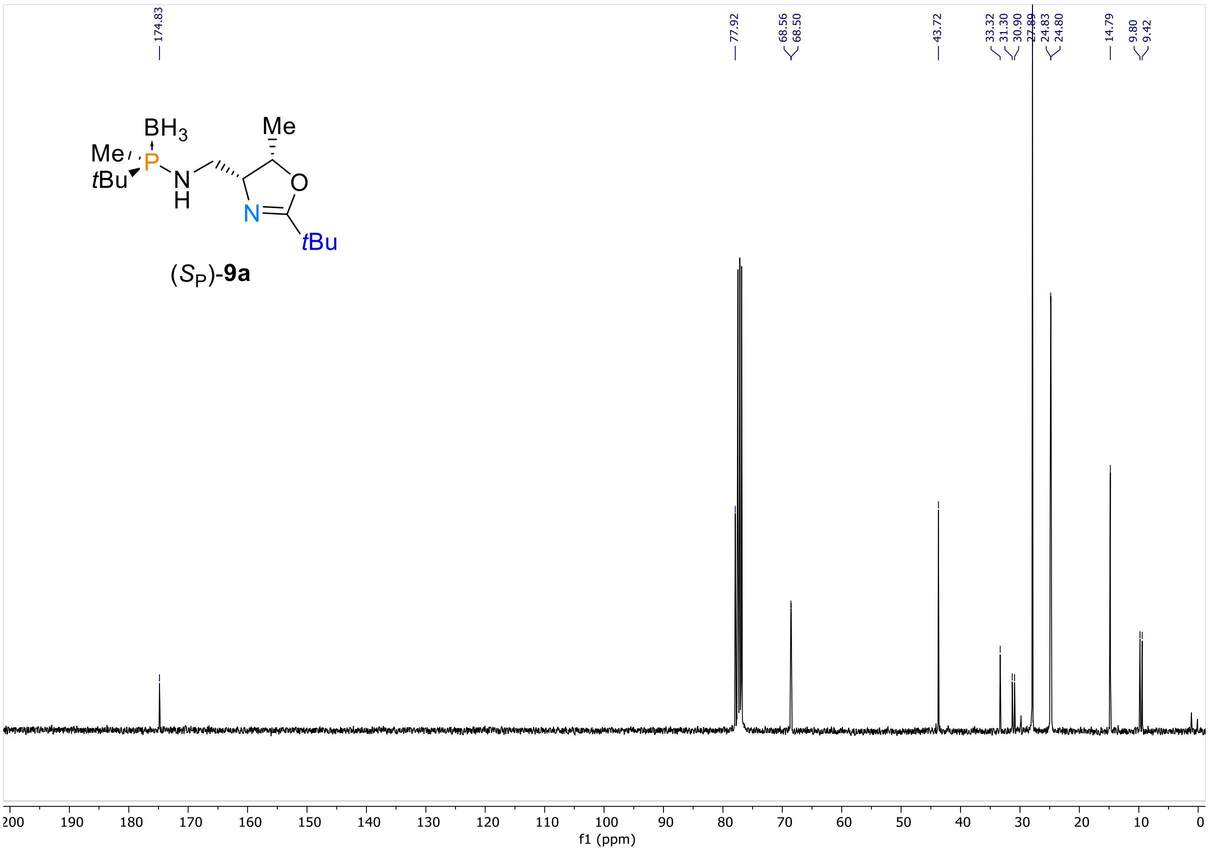

^{31}P-NMR (162 MHz, $CDCl_3$):

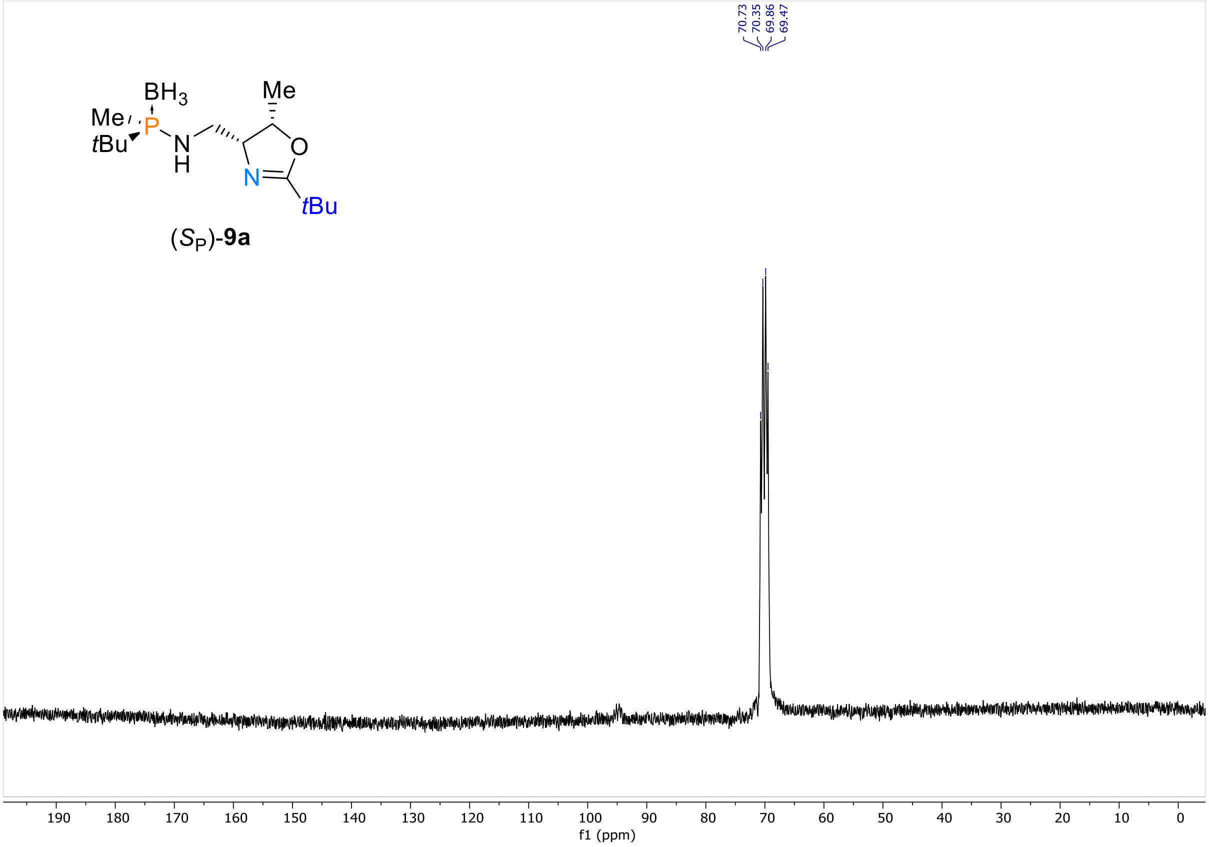

^{1}H-NMR (400 MHz, $CDCl_3$):

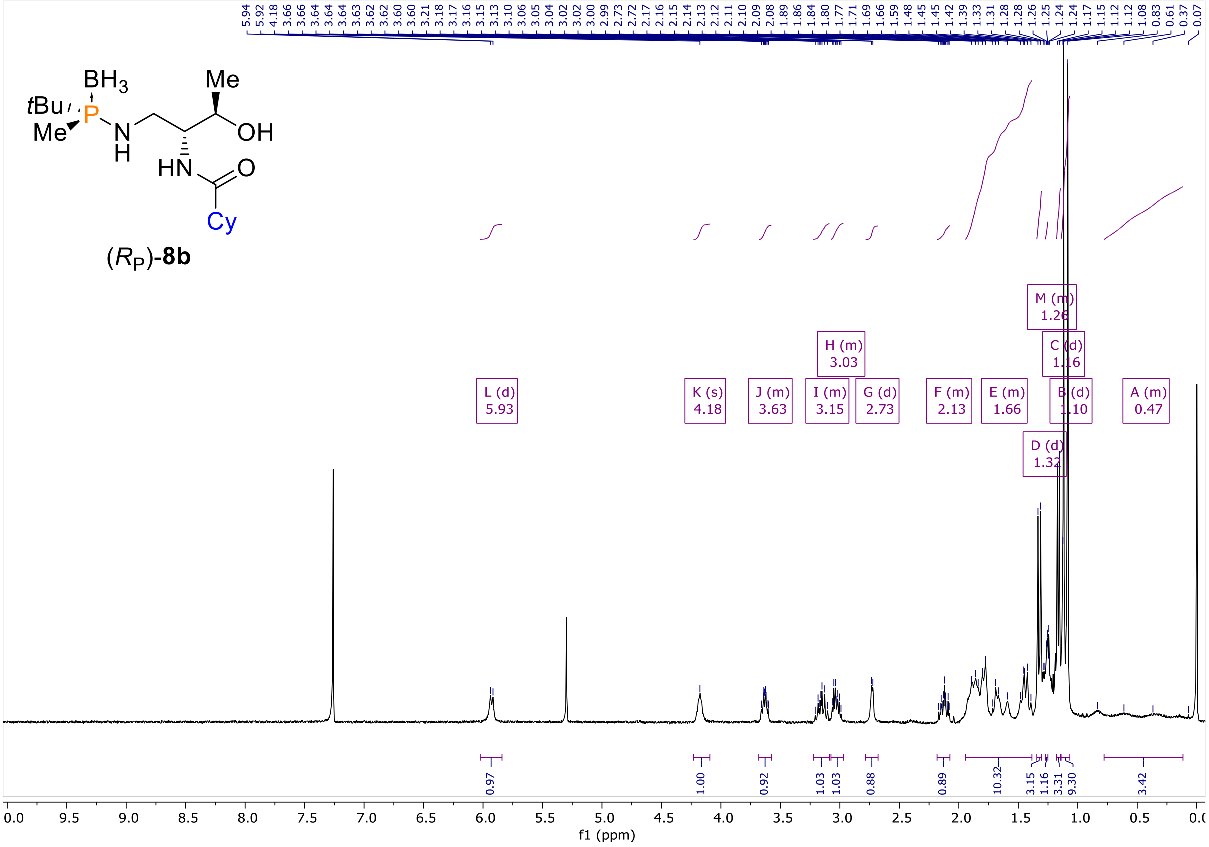

^{1}H-NMR (400 MHz, $CDCl_3$):

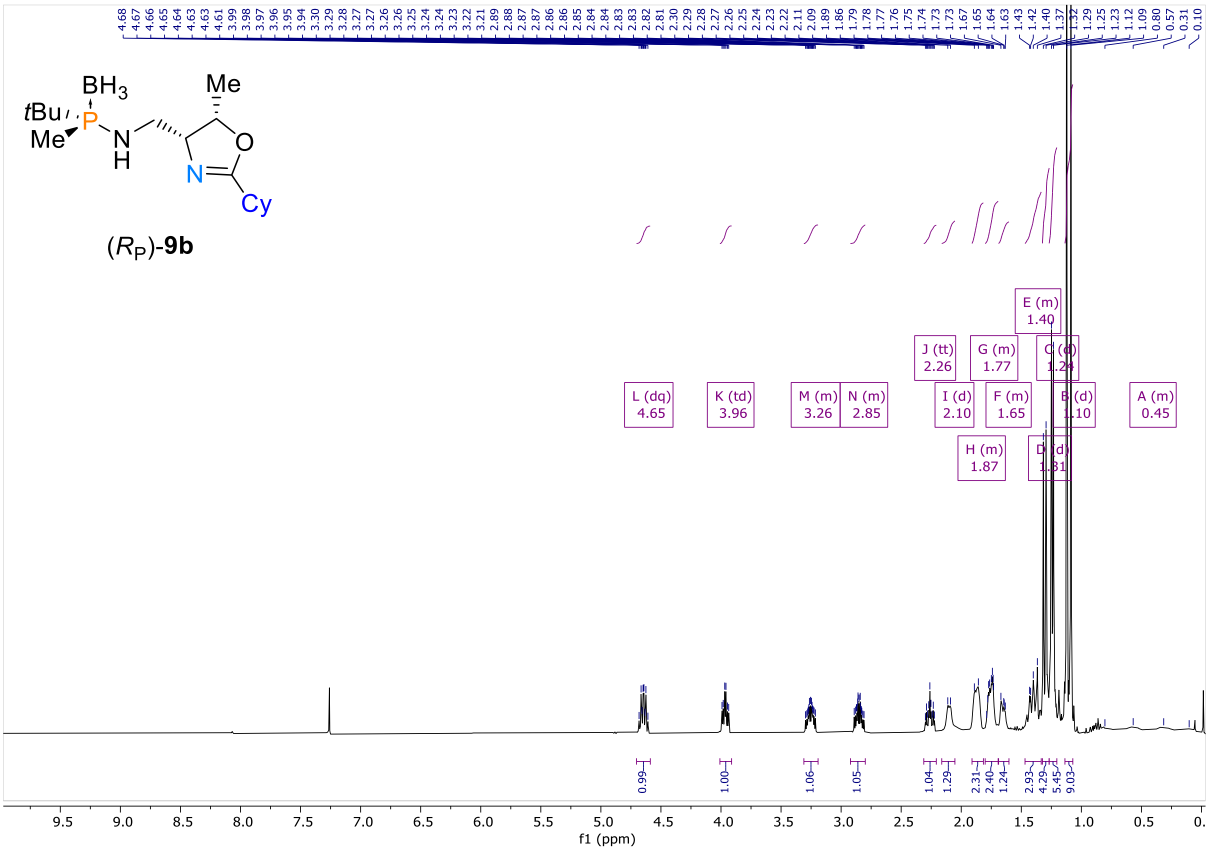

^{13}C-NMR (101 MHz, $CDCl_3$):

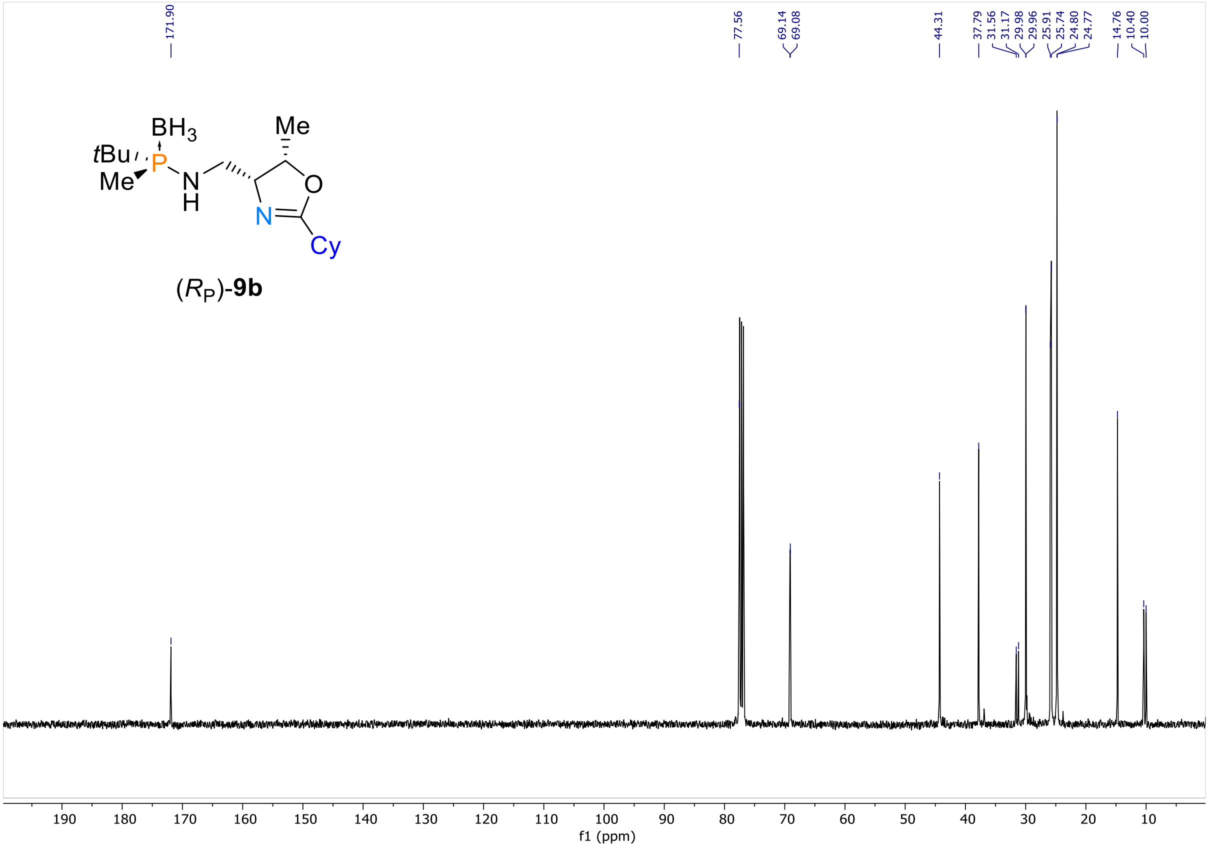

^{31}P-NMR (162 MHz, $CDCl_3$):

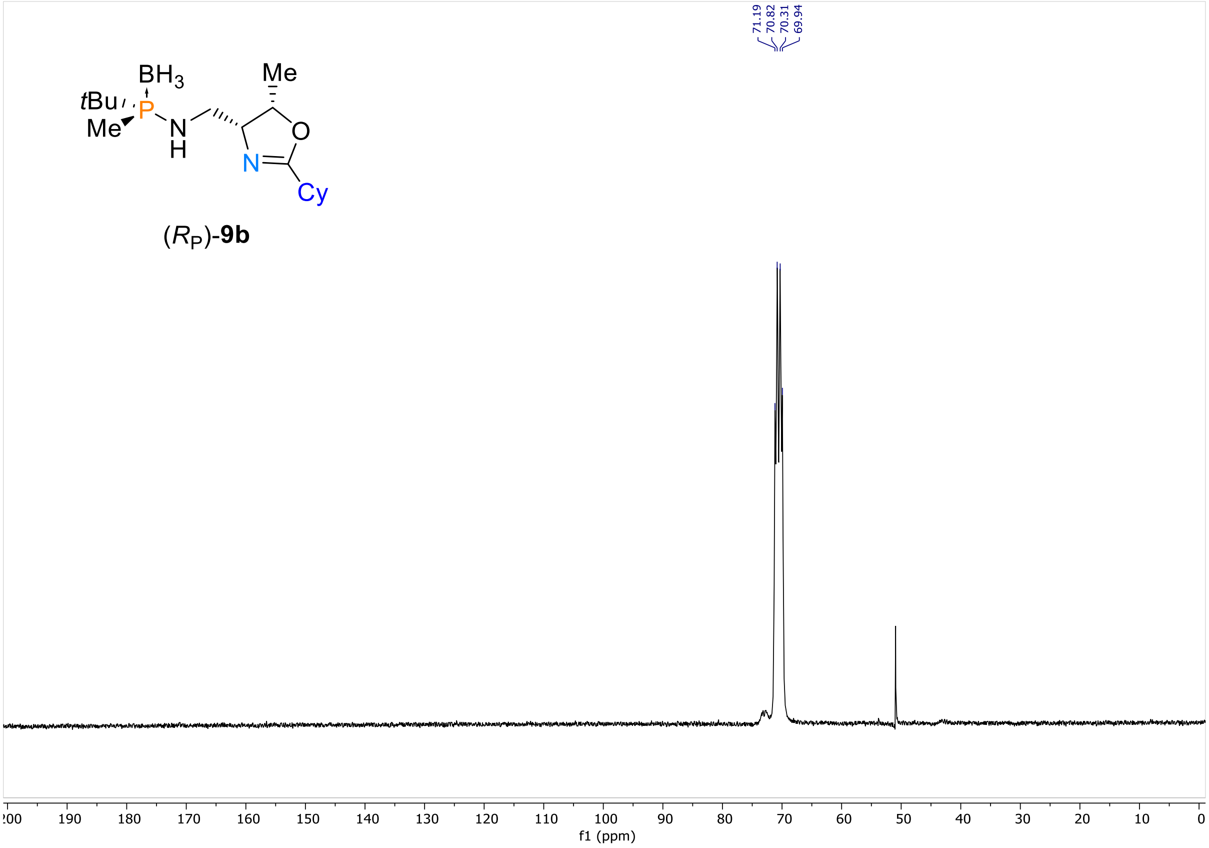

^{1}H-NMR (400 MHz, $CDCl_3$):

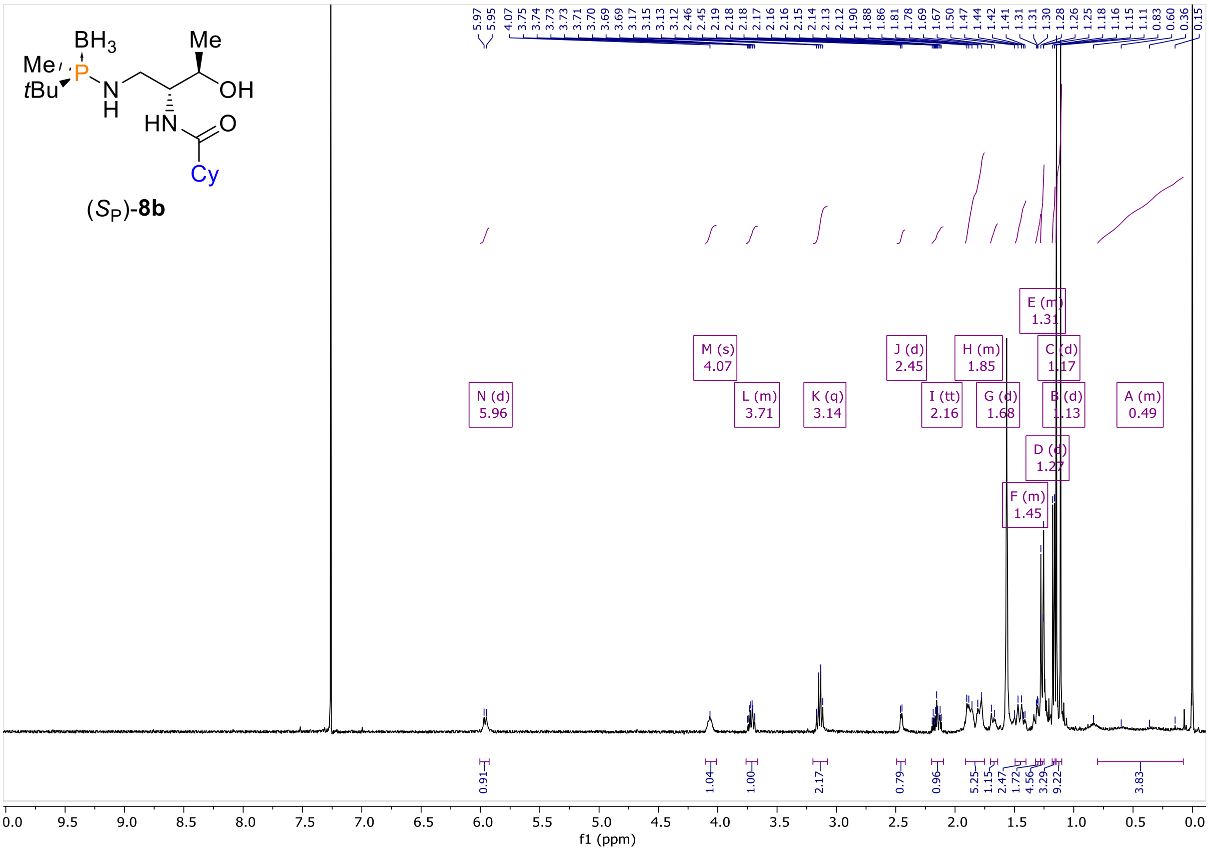

^{1}H-NMR (400 MHz, $CDCl_3$):

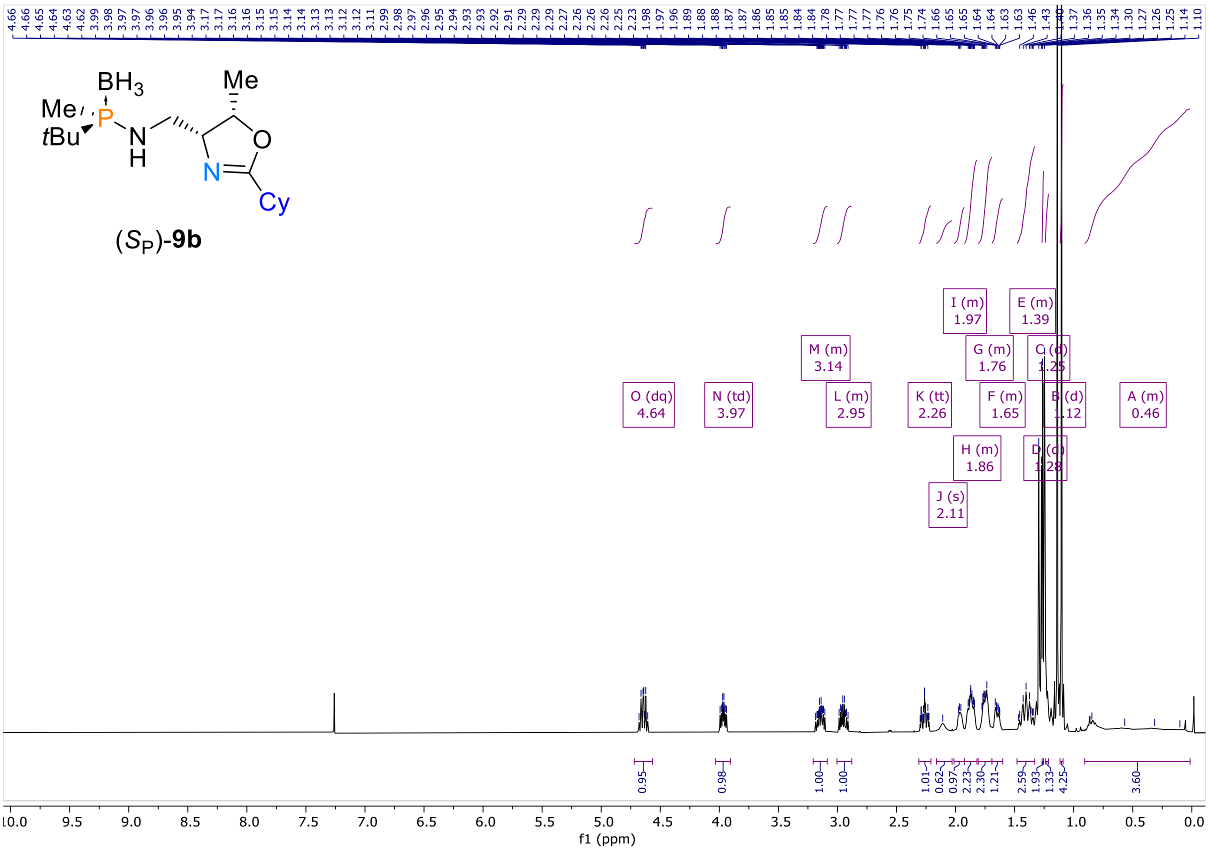

^{13}C-NMR (101 MHz, $CDCl_3$):

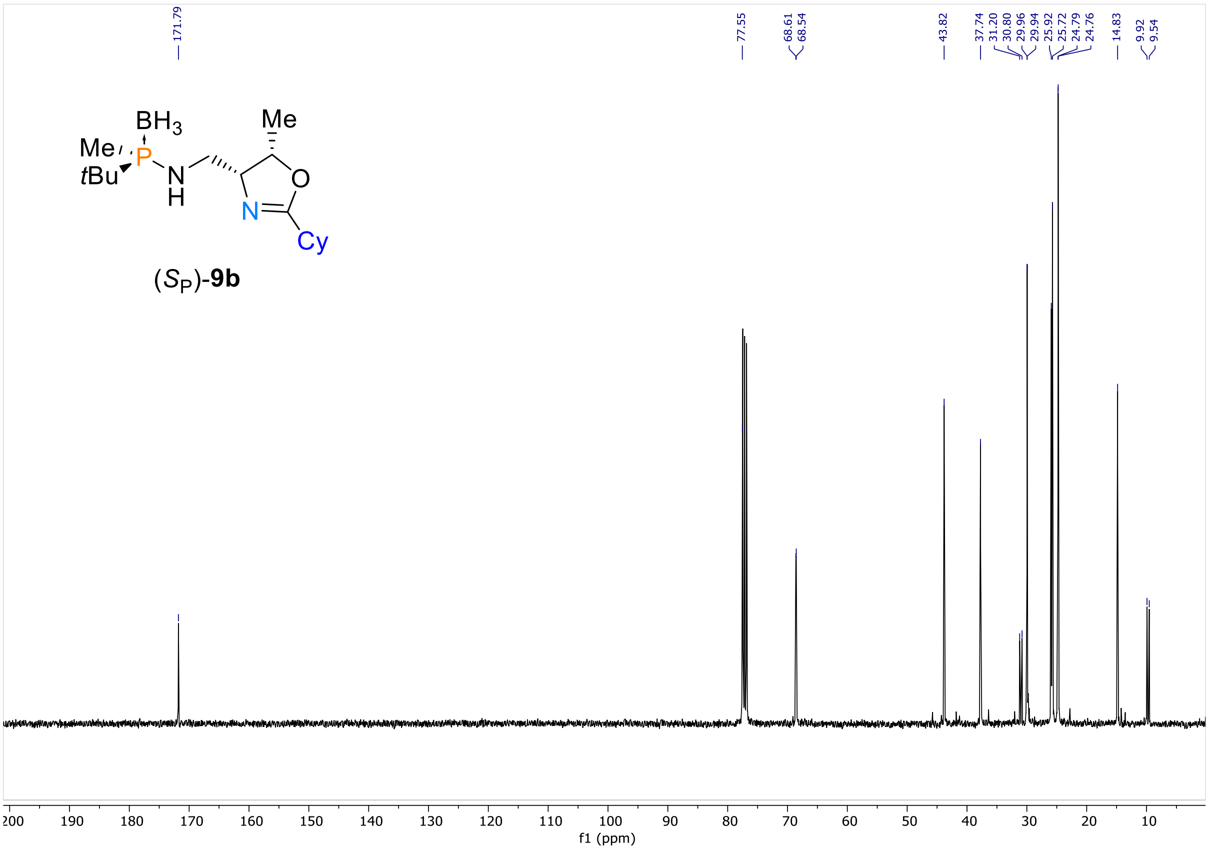

^{31}P-NMR (162 MHz, $CDCl_3$):

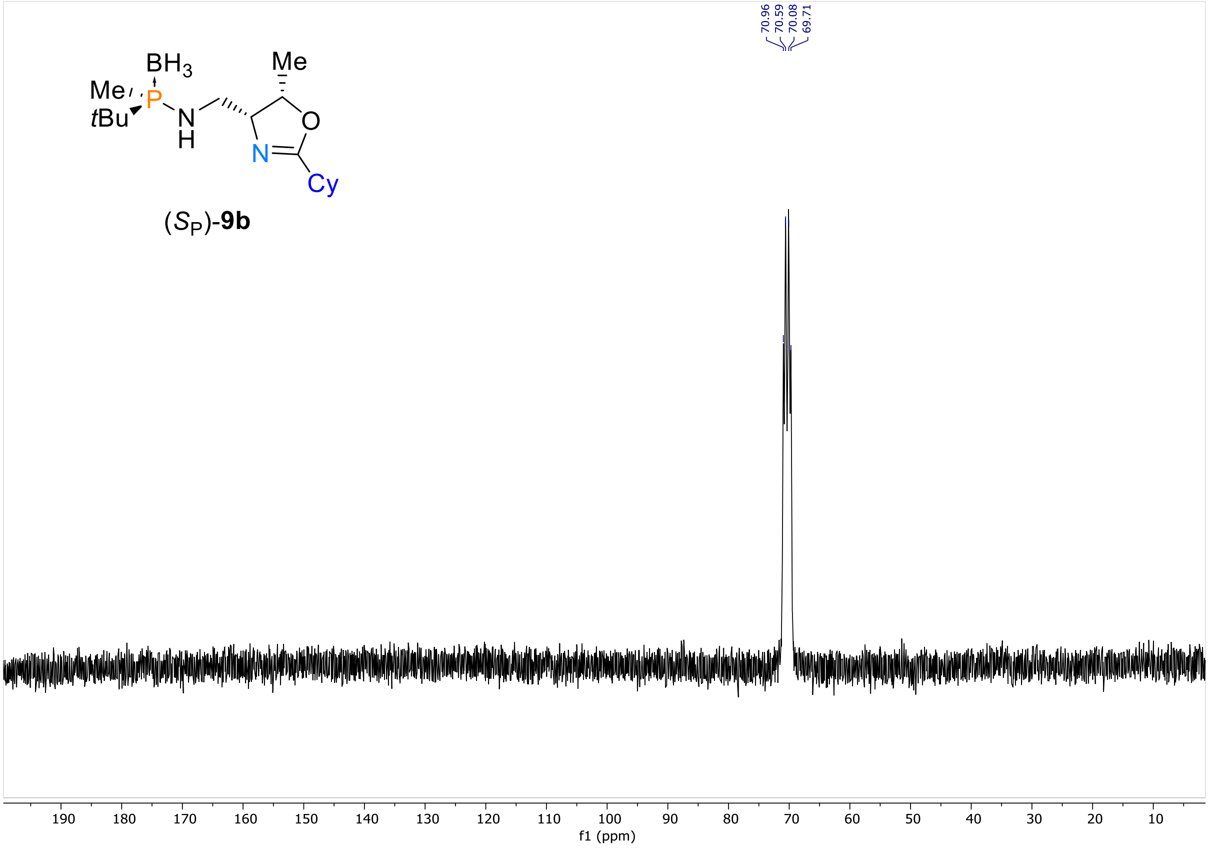

^{1}H-NMR (400 MHz, $CDCl_3$):

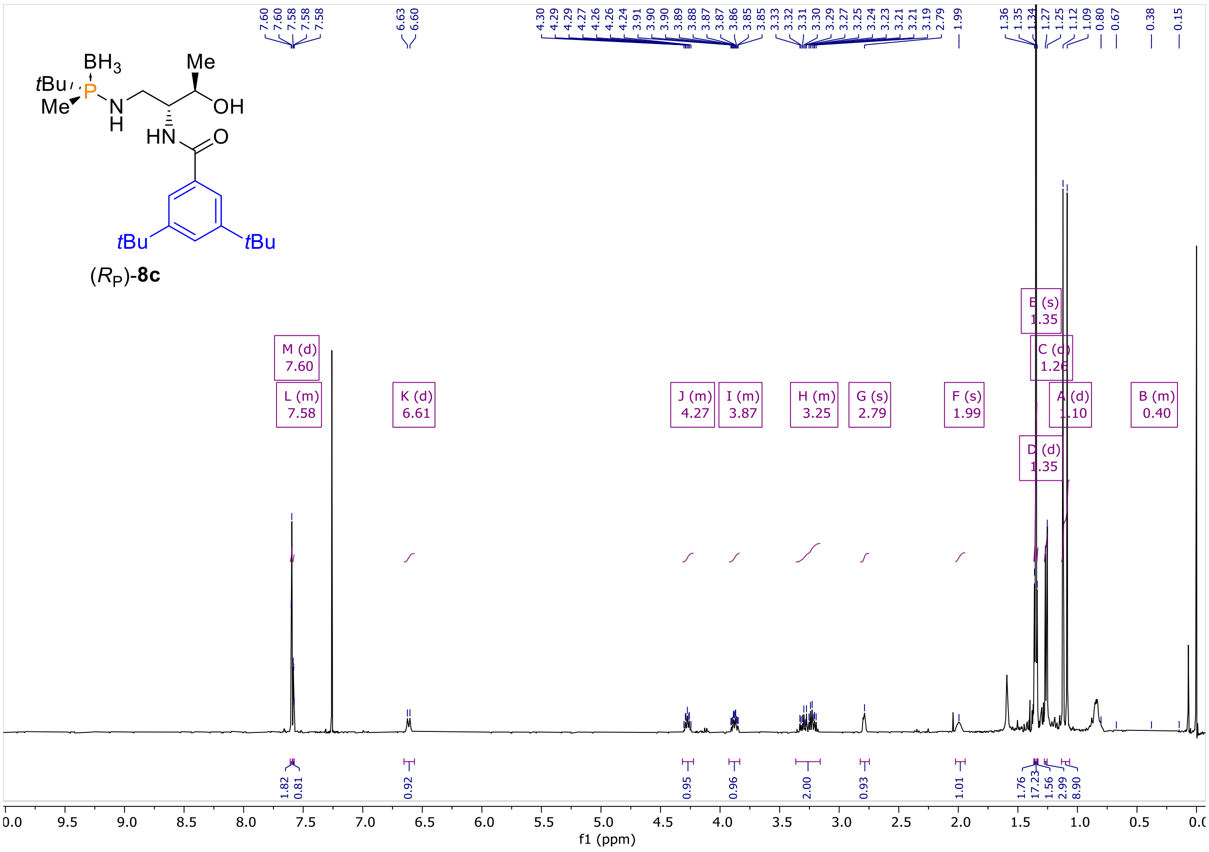

^{1}H-NMR (400 MHz, $CDCl_3$):

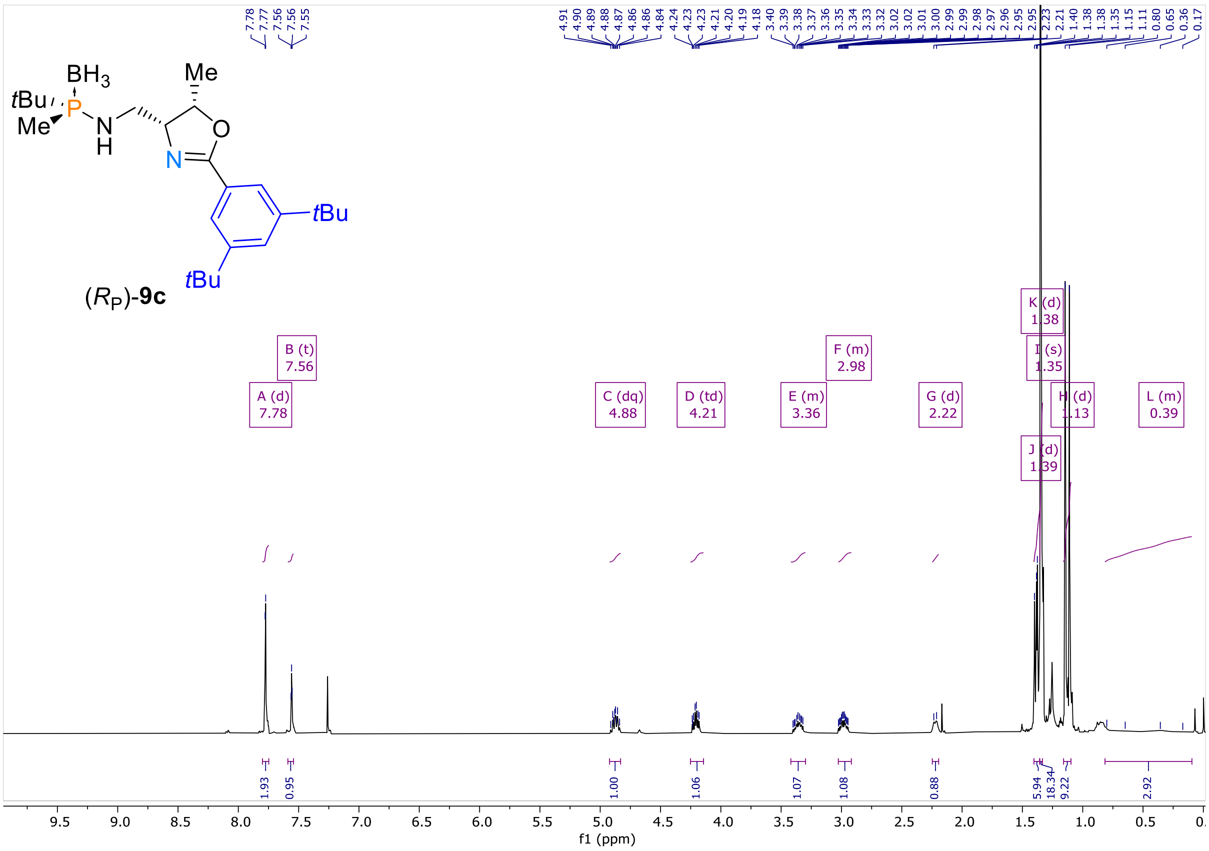

^{13}C-NMR (101 MHz, $CDCl_3$):

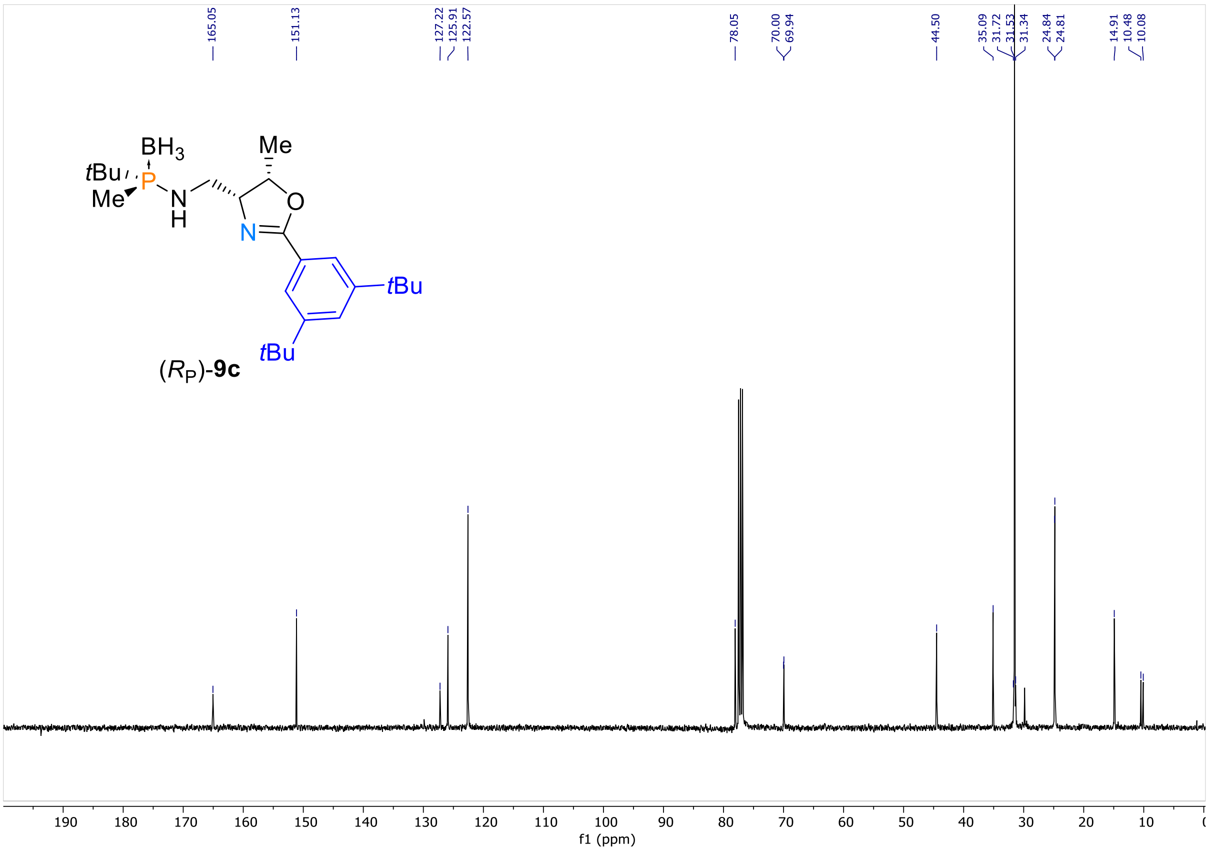

^{31}P-NMR (162 MHz, $CDCl_3$):

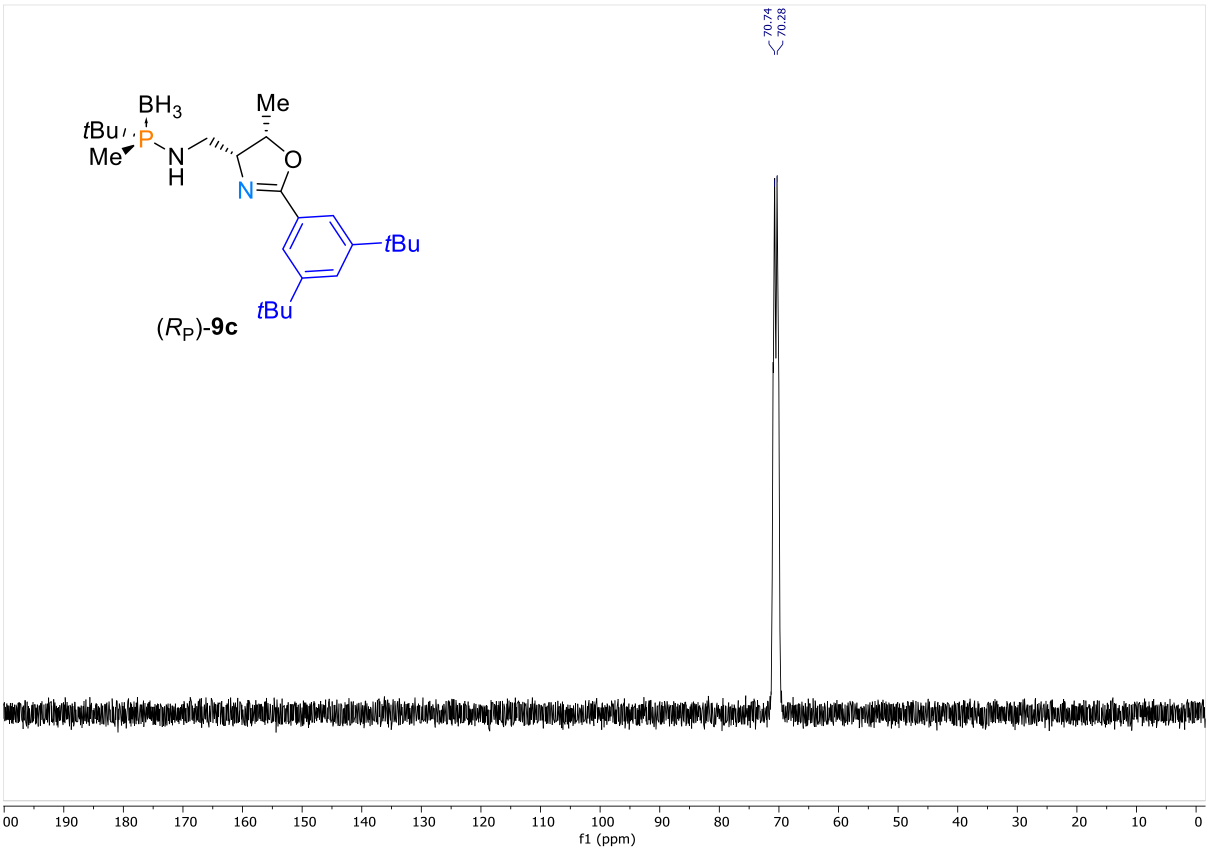

^{1}H-NMR (400 MHz, $CDCl_3$):

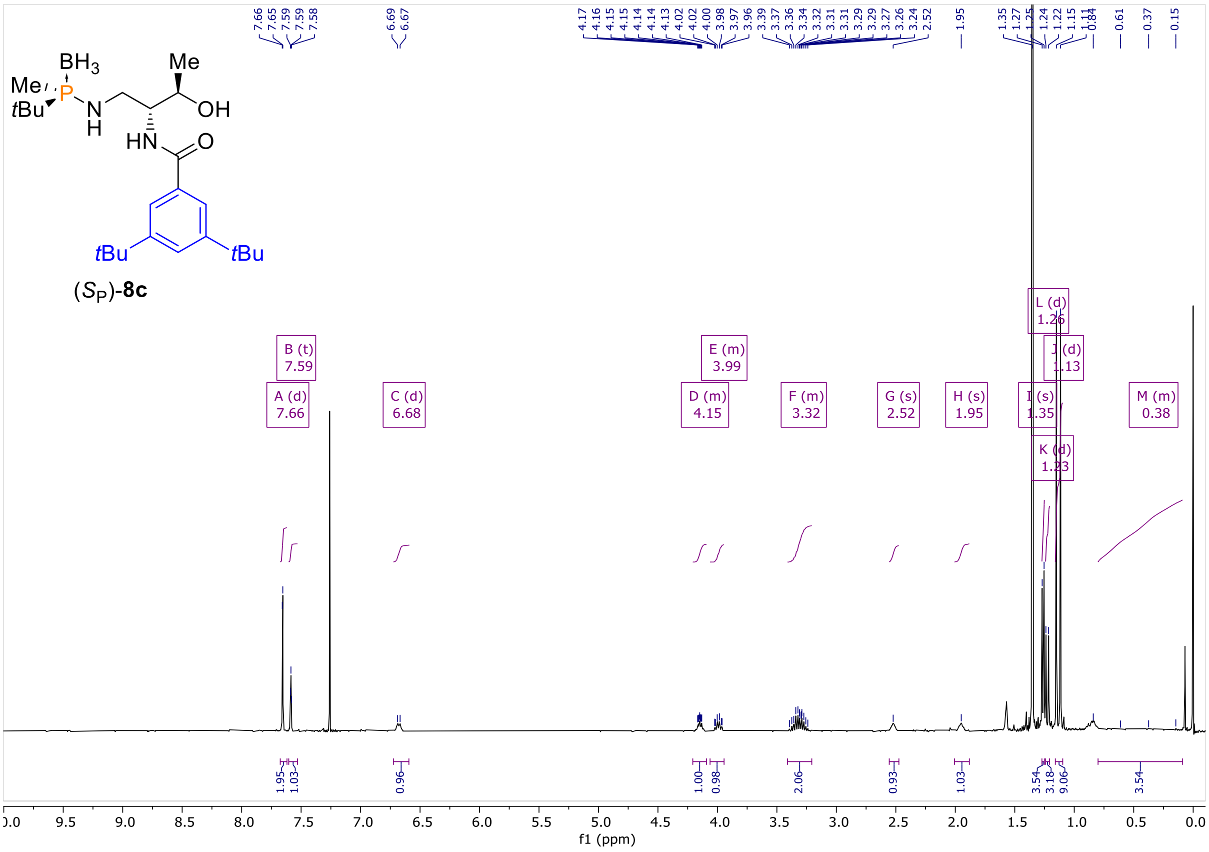

^{1}H-NMR (400 MHz, $CDCl_3$):

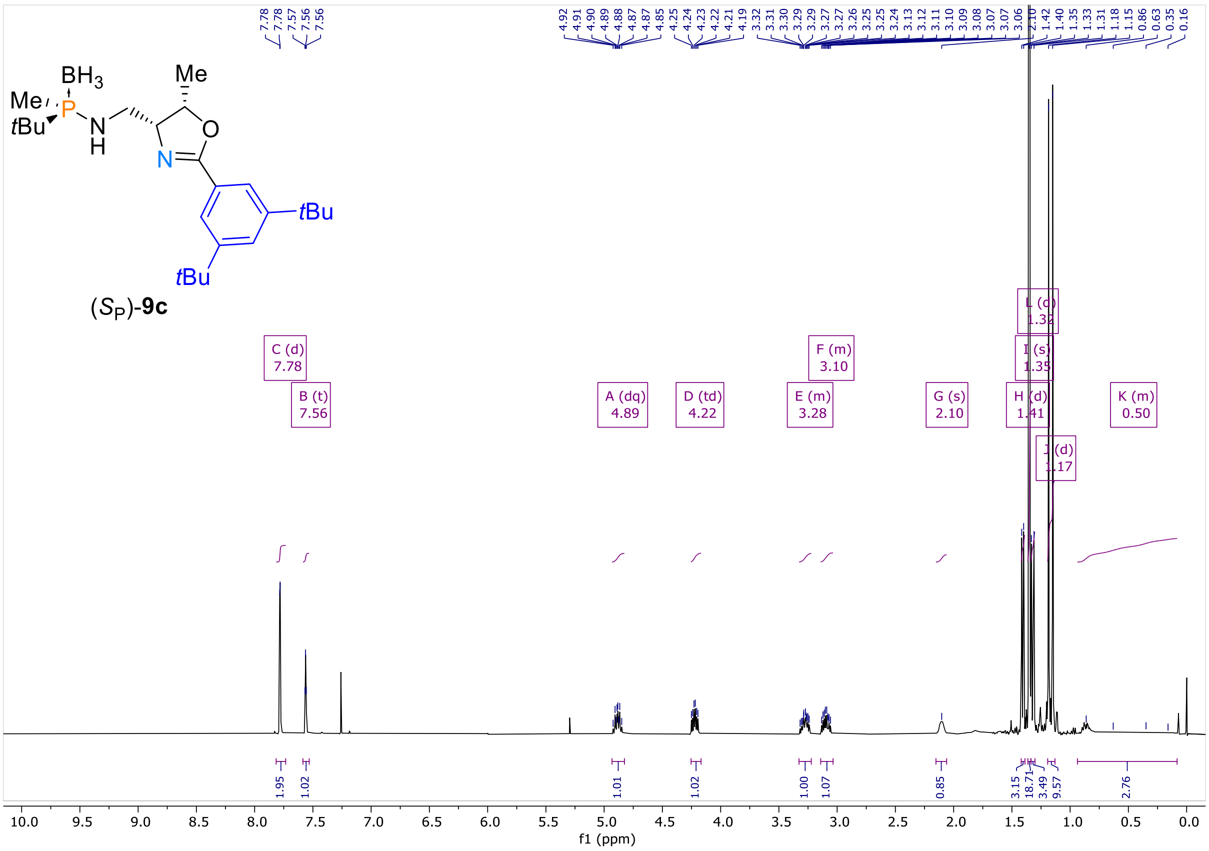

^{13}C-NMR (101 MHz, $CDCl_3$):

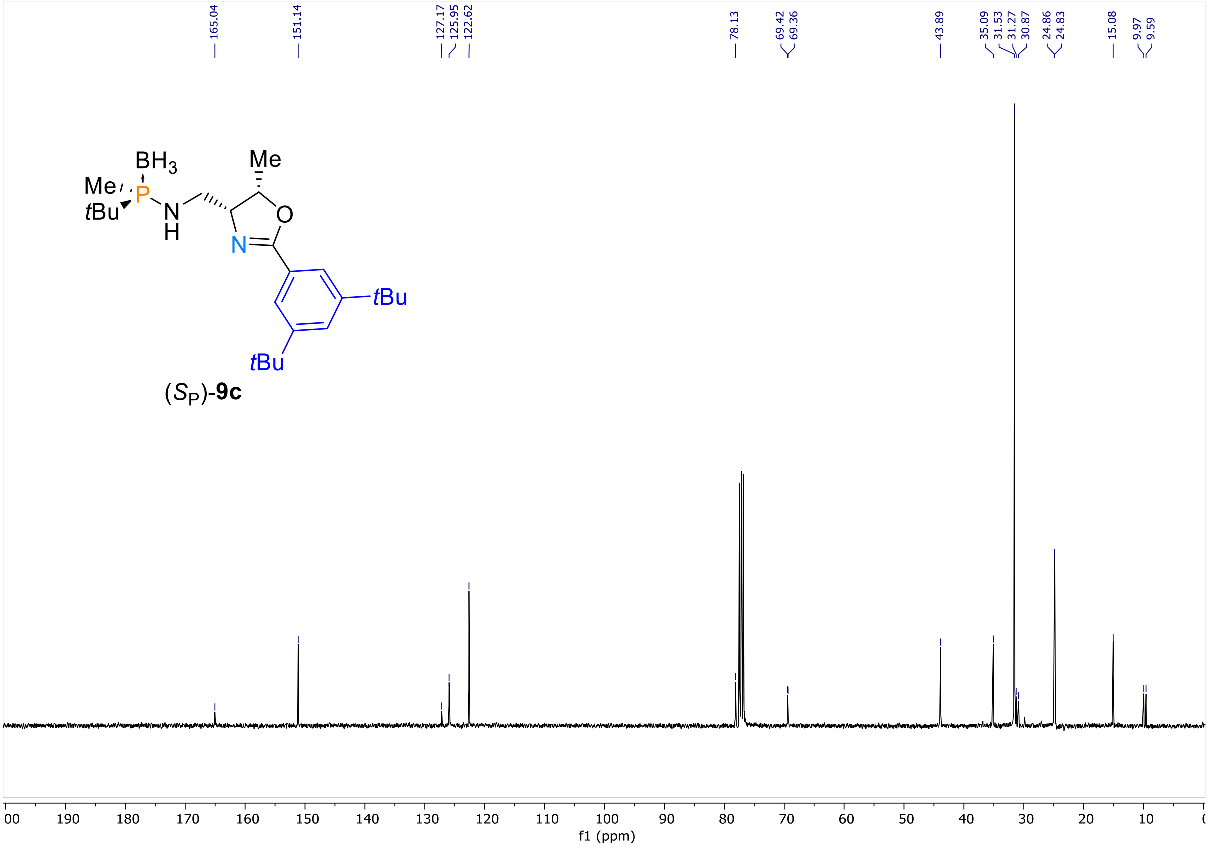

^{31}P-NMR (162 MHz, $CDCl_3$):

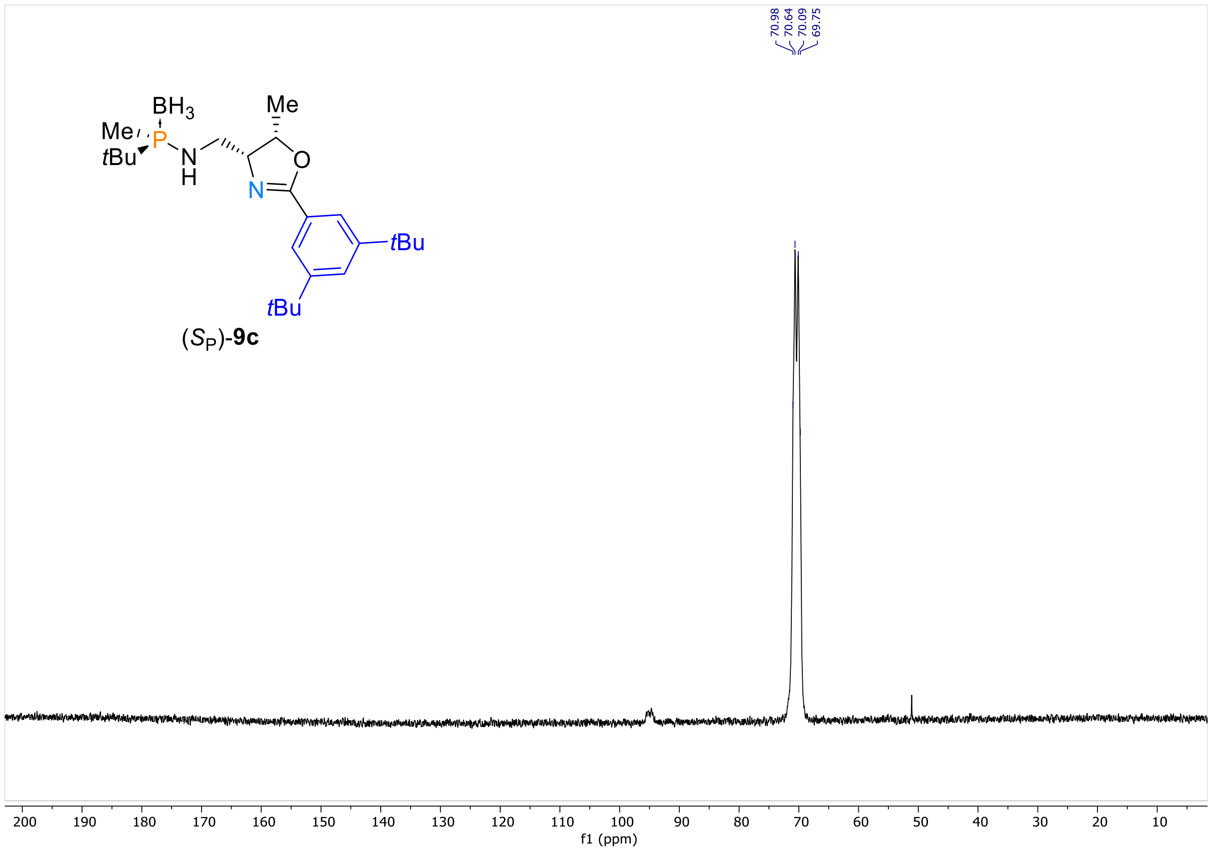

^{1}H-NMR (400 MHz, $CDCl_3$):

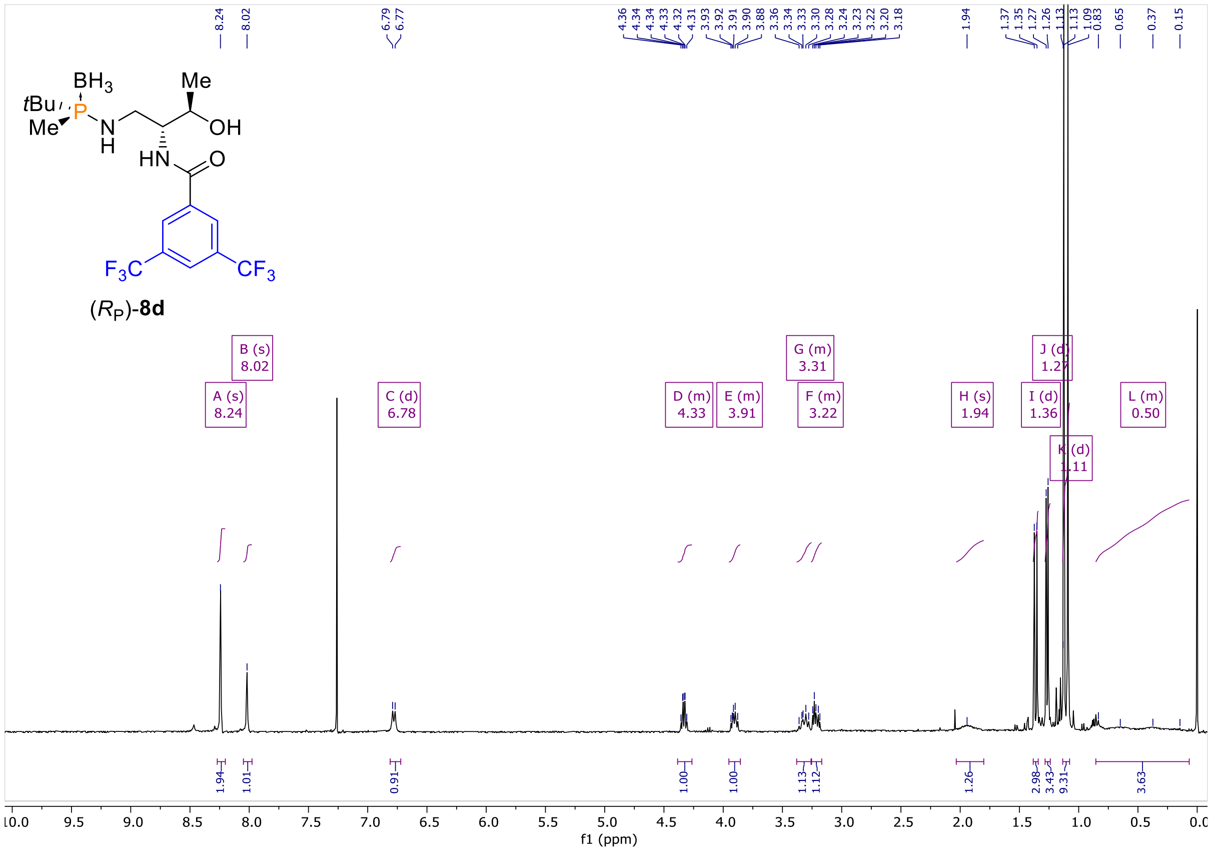

^{1}H-NMR (400 MHz, $CDCl_3$):

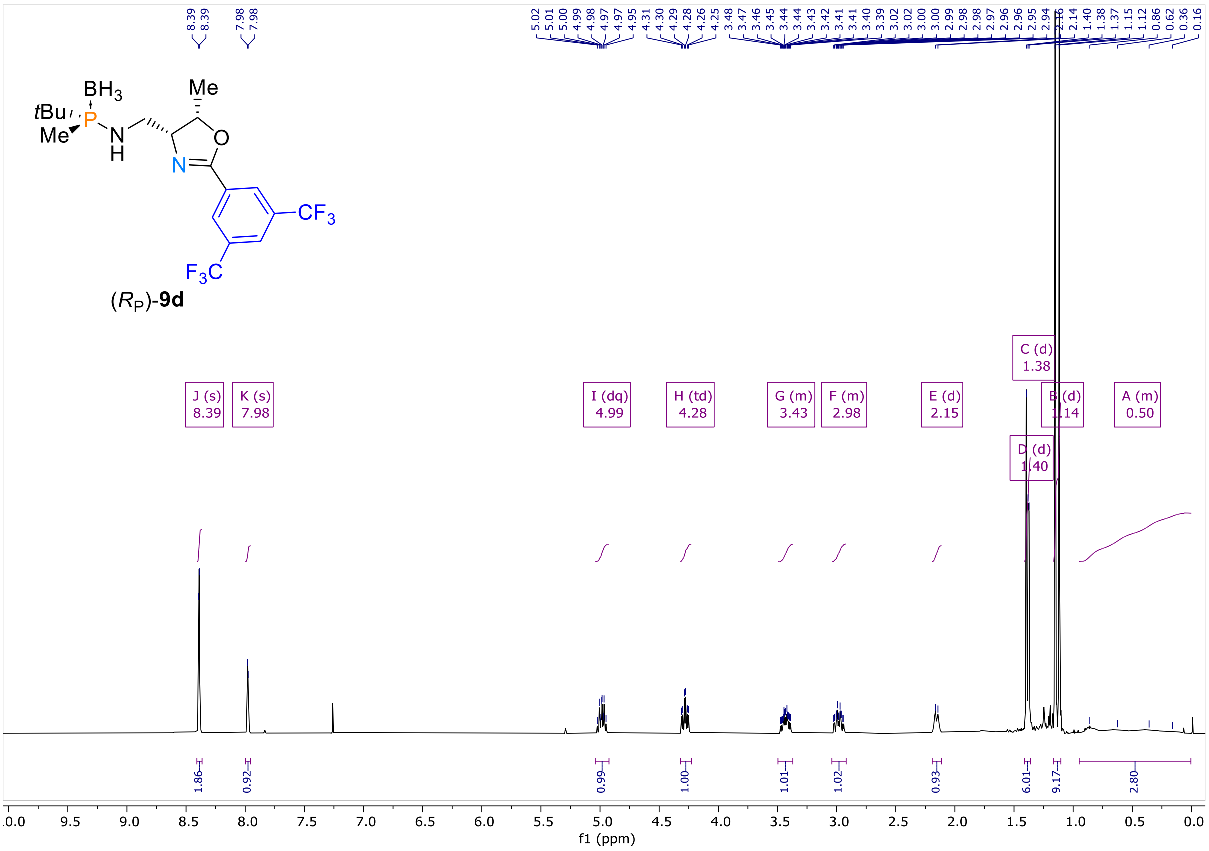

^{13}C-NMR (101 MHz, $CDCl_3$):

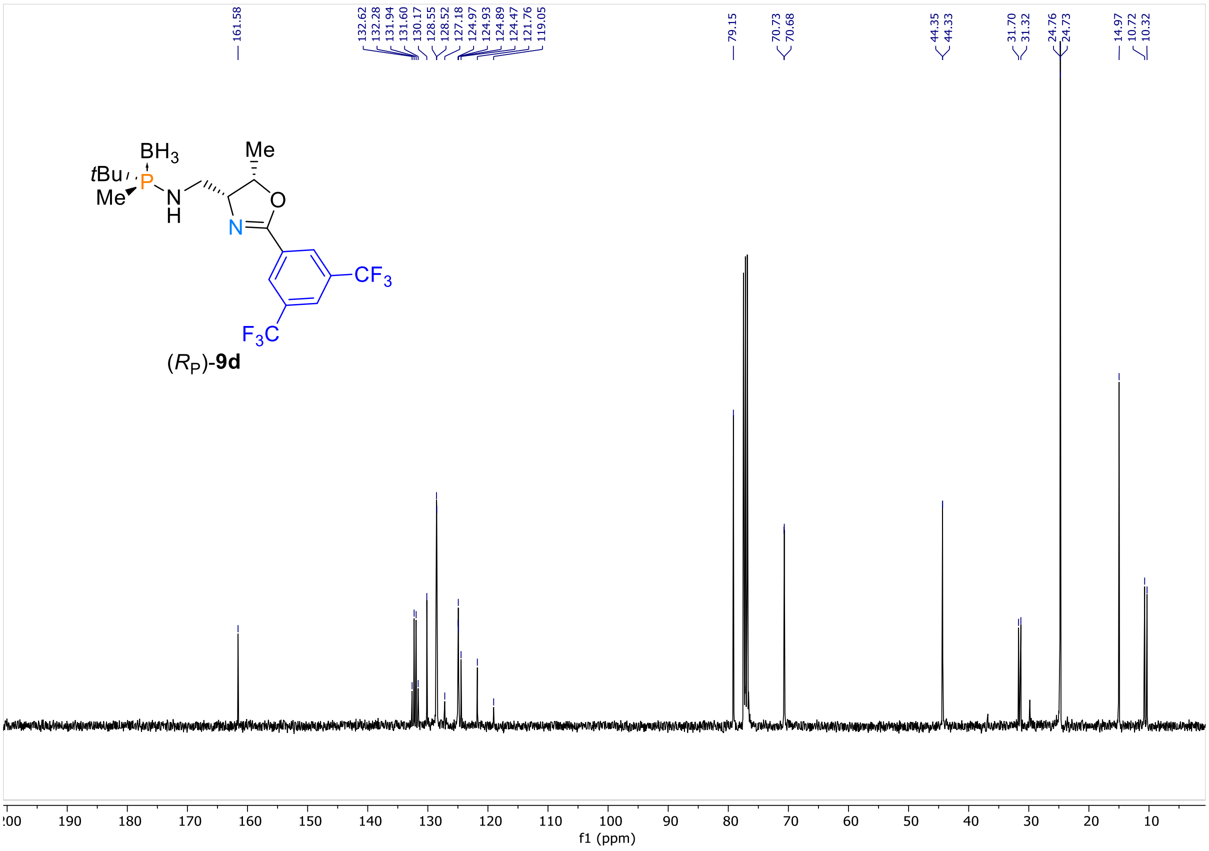

^{31}P-NMR (162 MHz, $CDCl_3$):

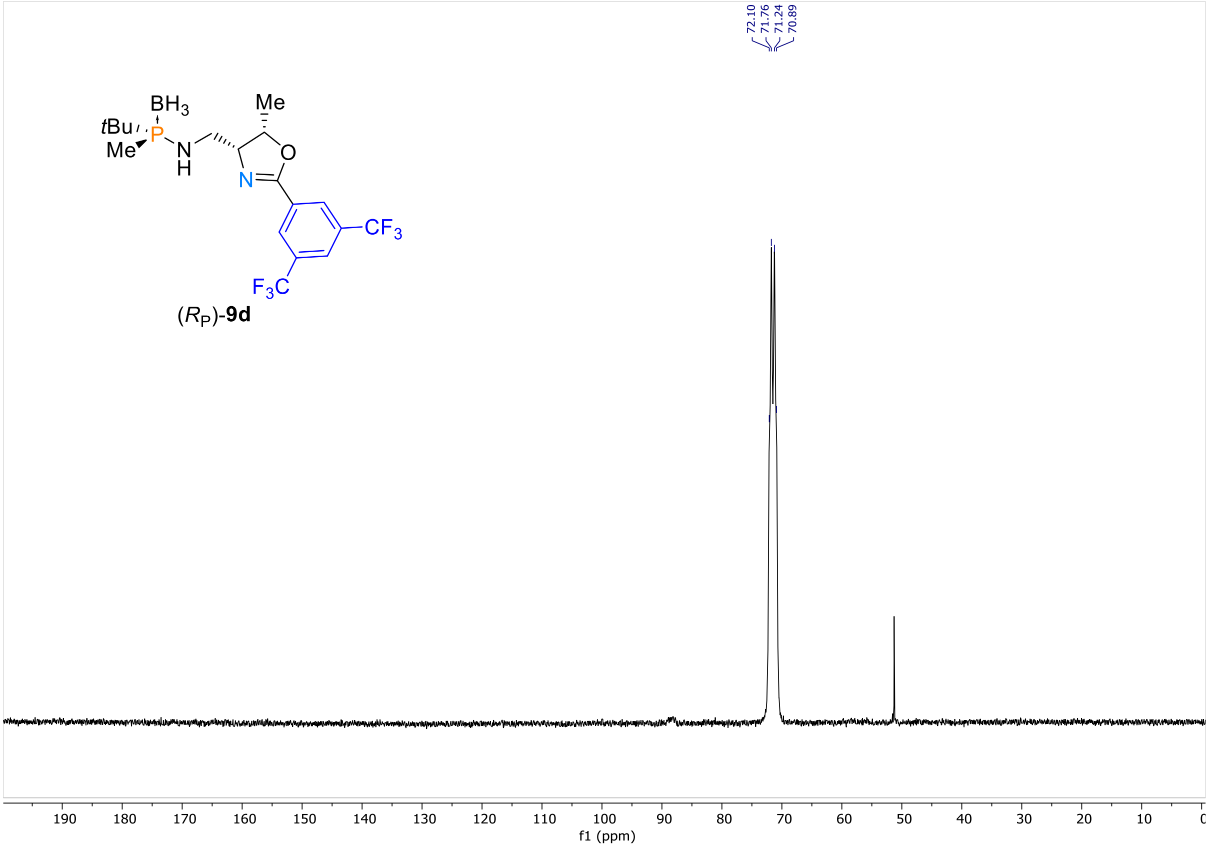

^{1}H-NMR (400 MHz, $CDCl_3$):

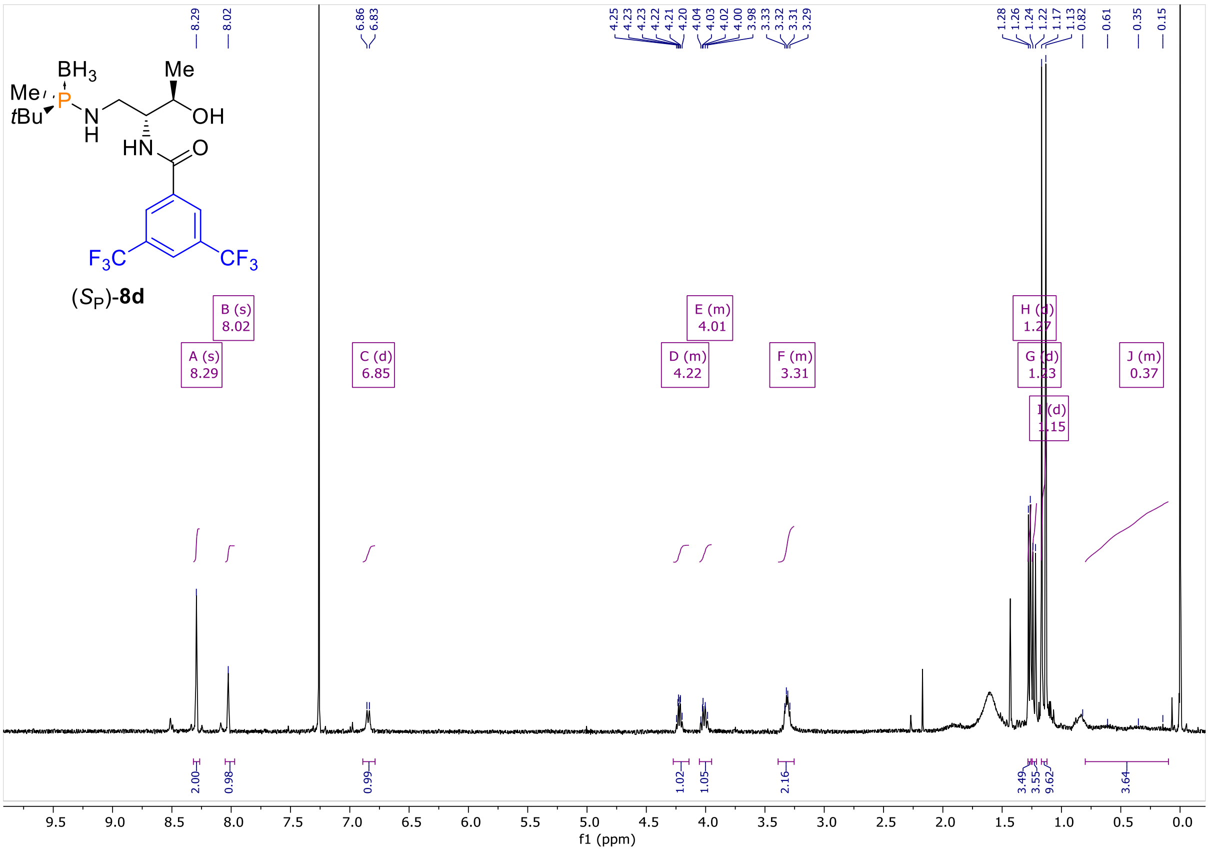

^{1}H-NMR (400 MHz, $CDCl_3$):

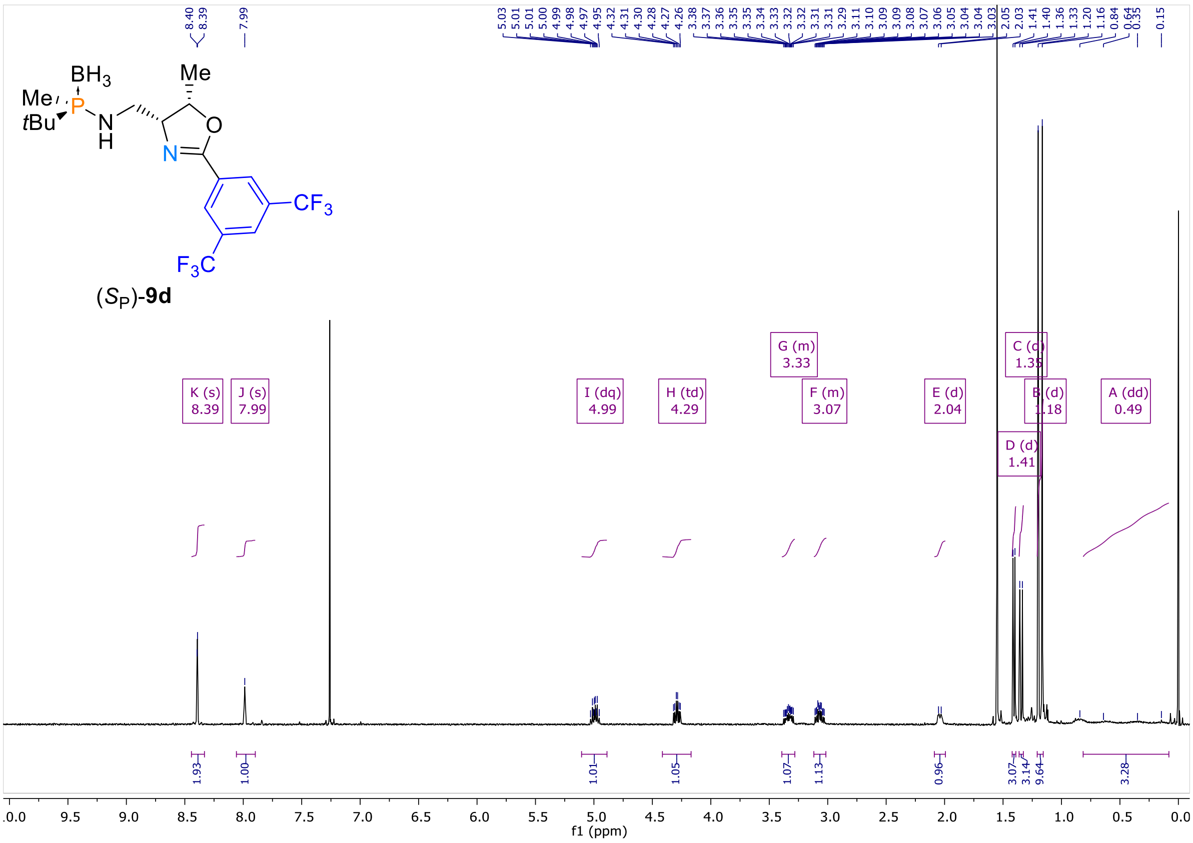

^{13}C-NMR (101 MHz, CDCl$_3$):

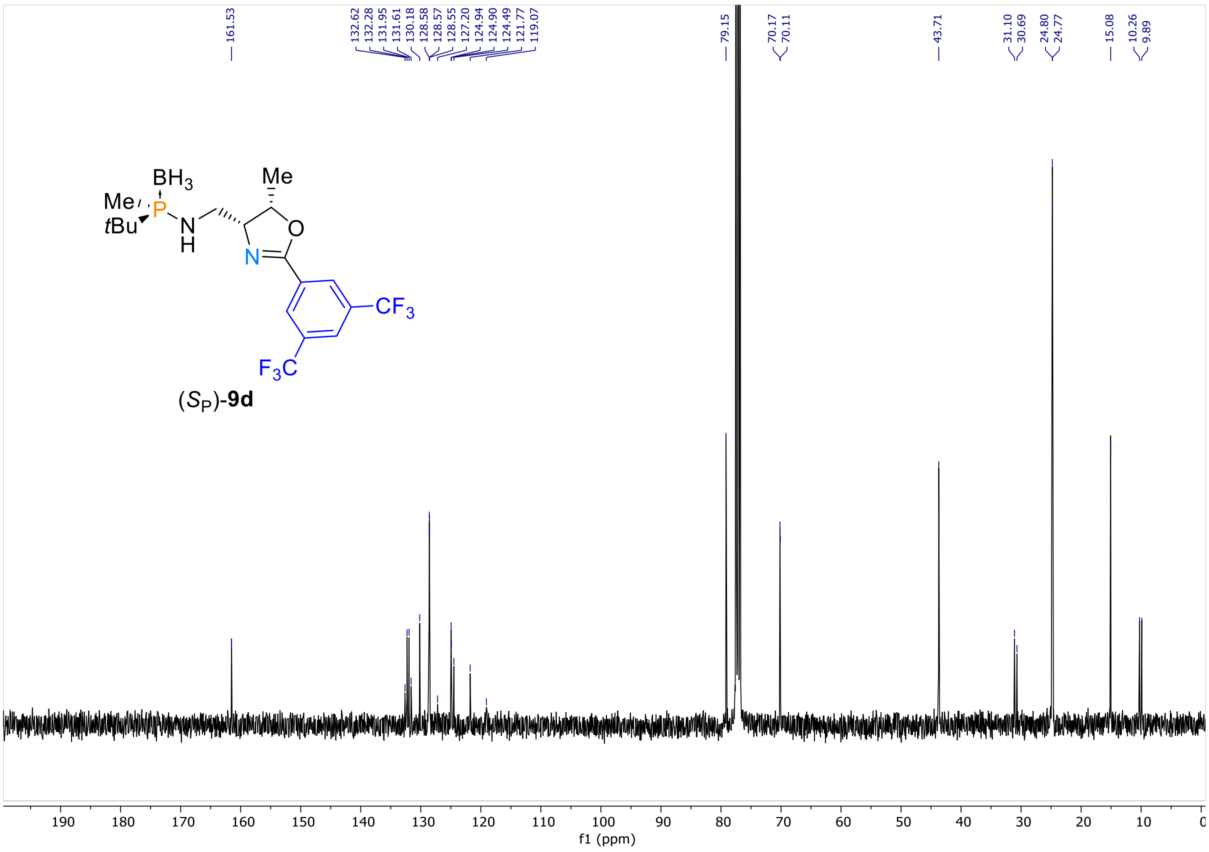

^{31}P-NMR (162 MHz, CDCl$_3$):

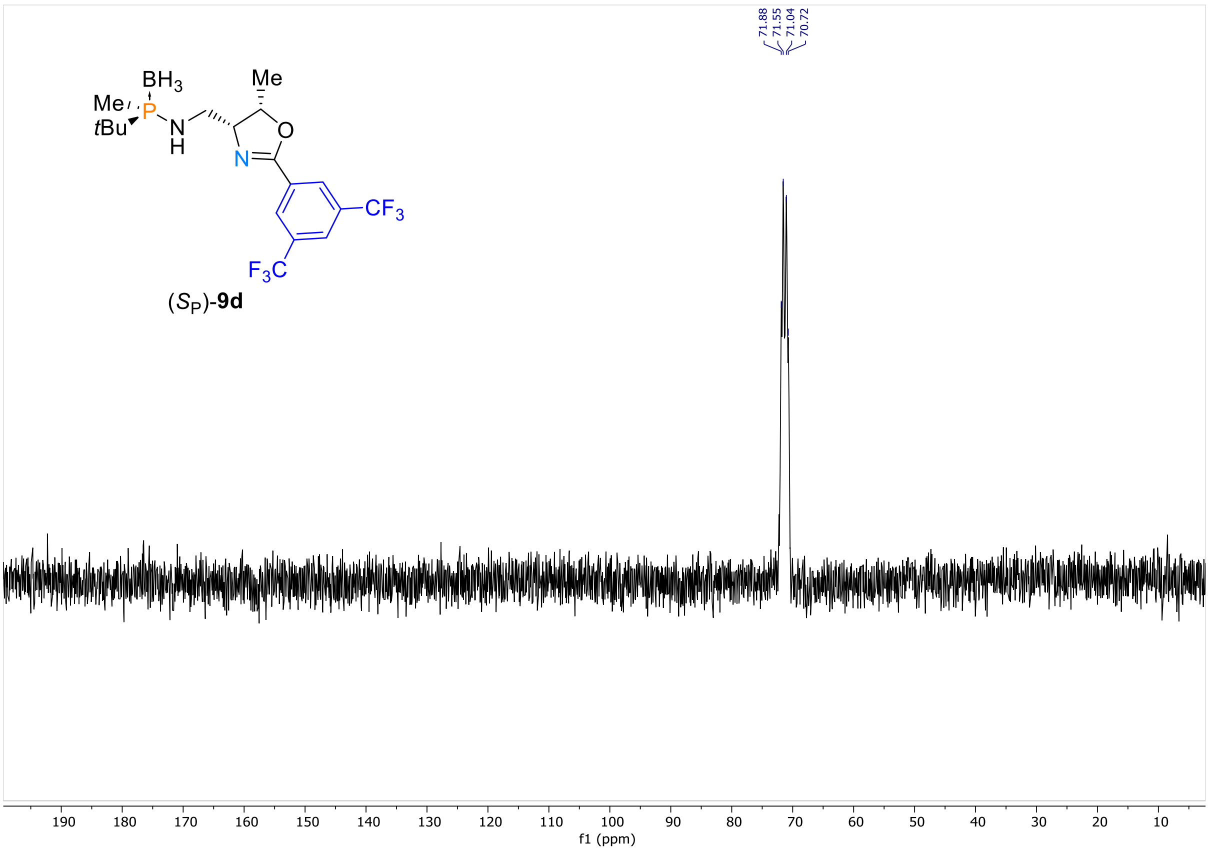

^{1}H-NMR (400 MHz, $CDCl_3$):

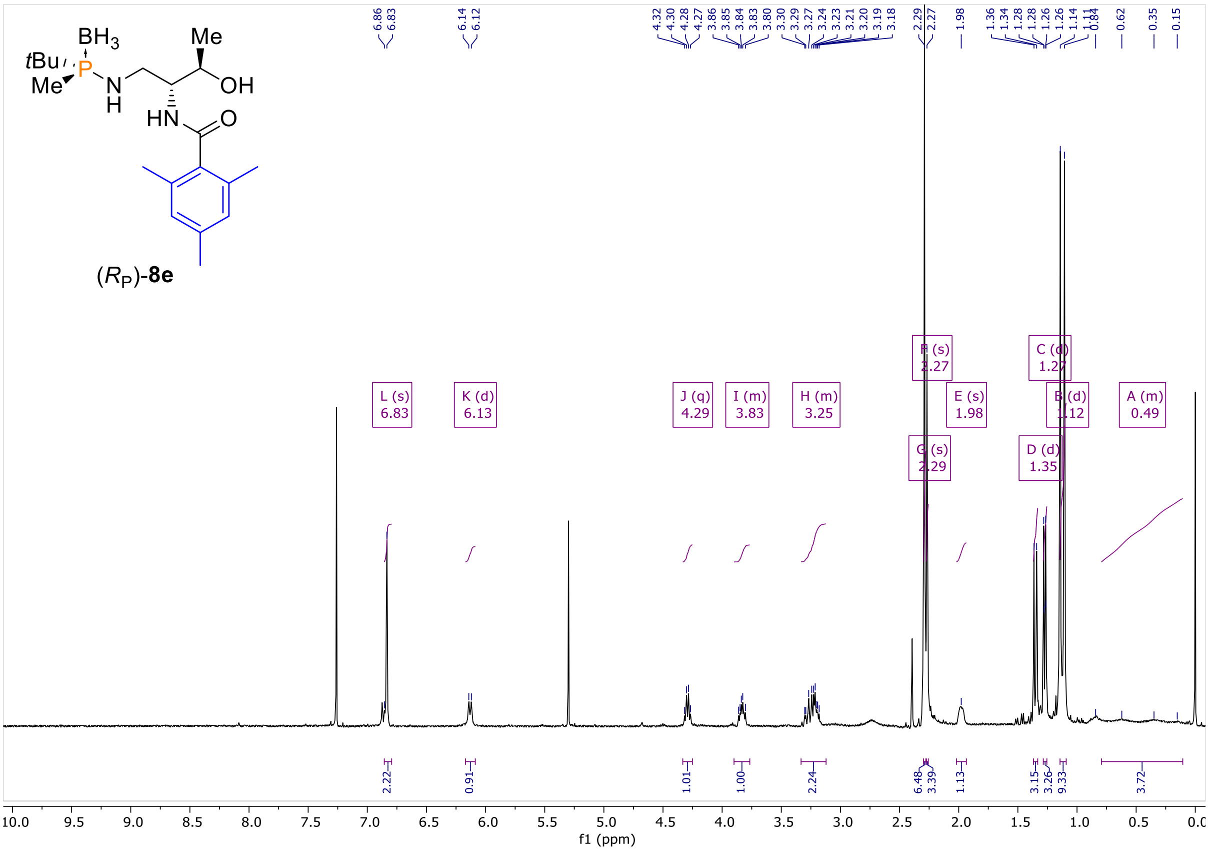

^{1}H-NMR (400 MHz, $CDCl_3$):

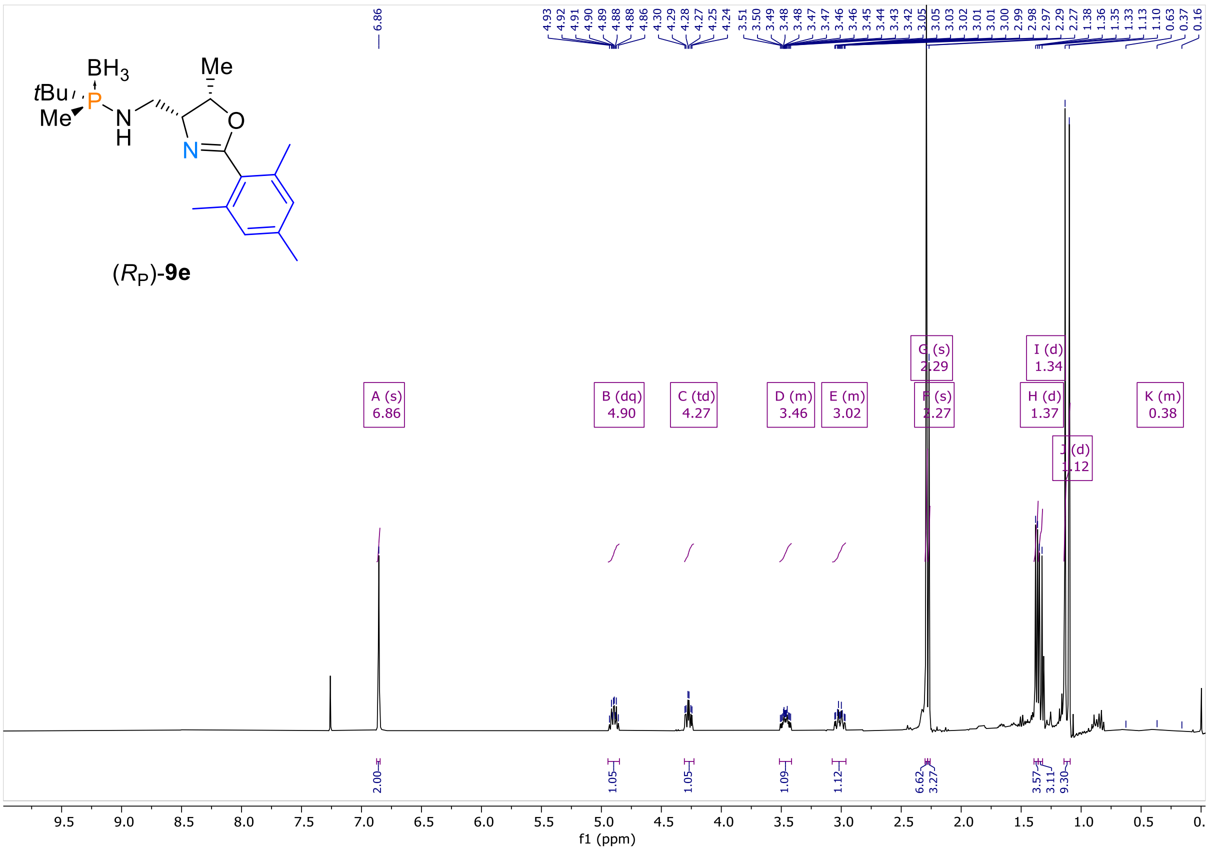

^{13}C-NMR (101 MHz, $CDCl_3$):

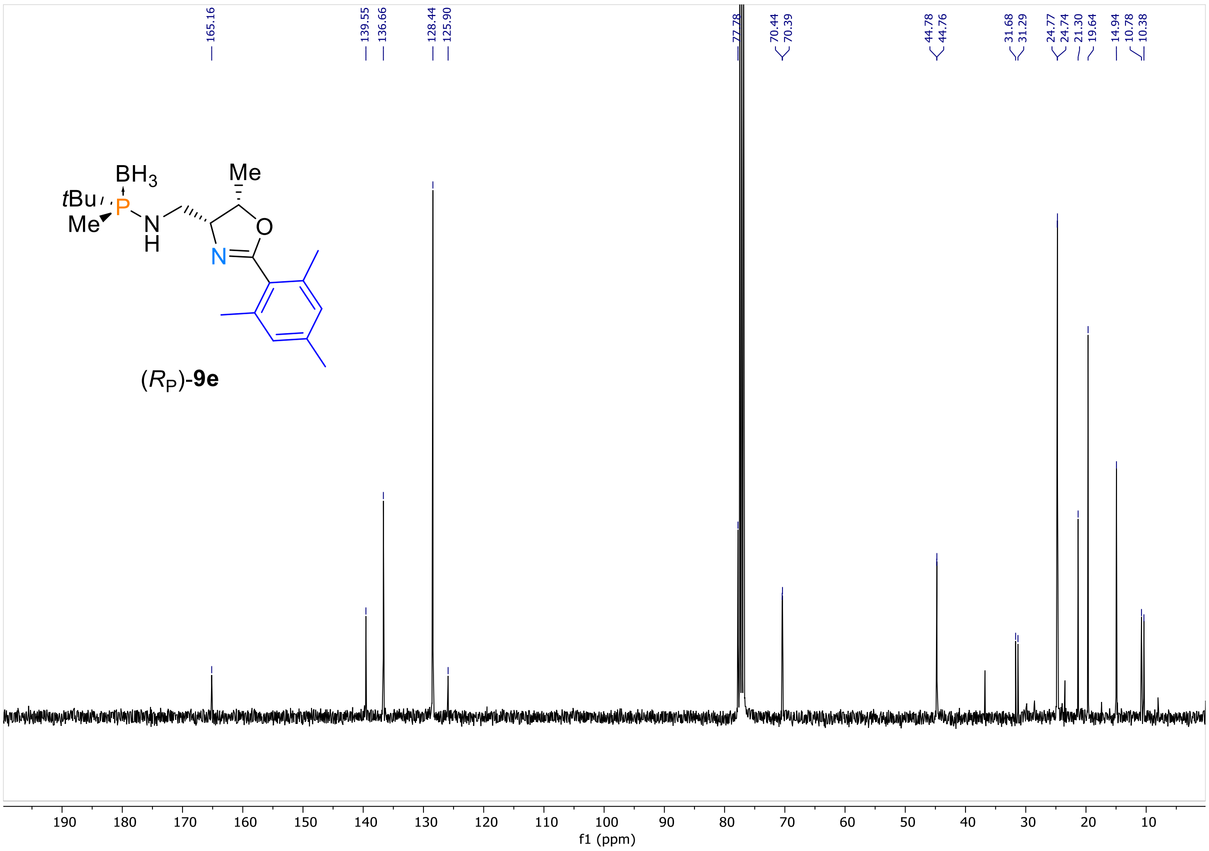

^{31}P-NMR (162 MHz, $CDCl_3$):

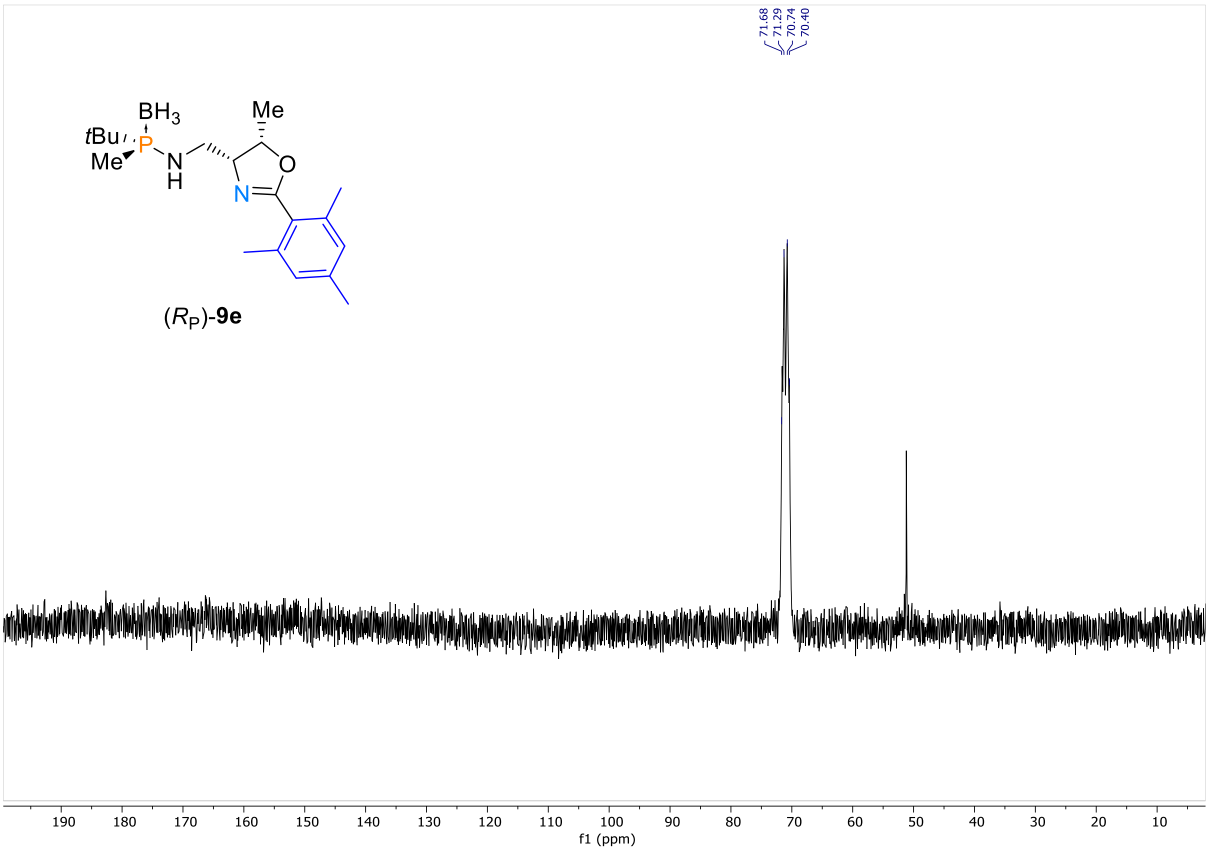

^{1}H-NMR (400 MHz, CDCl$_3$):

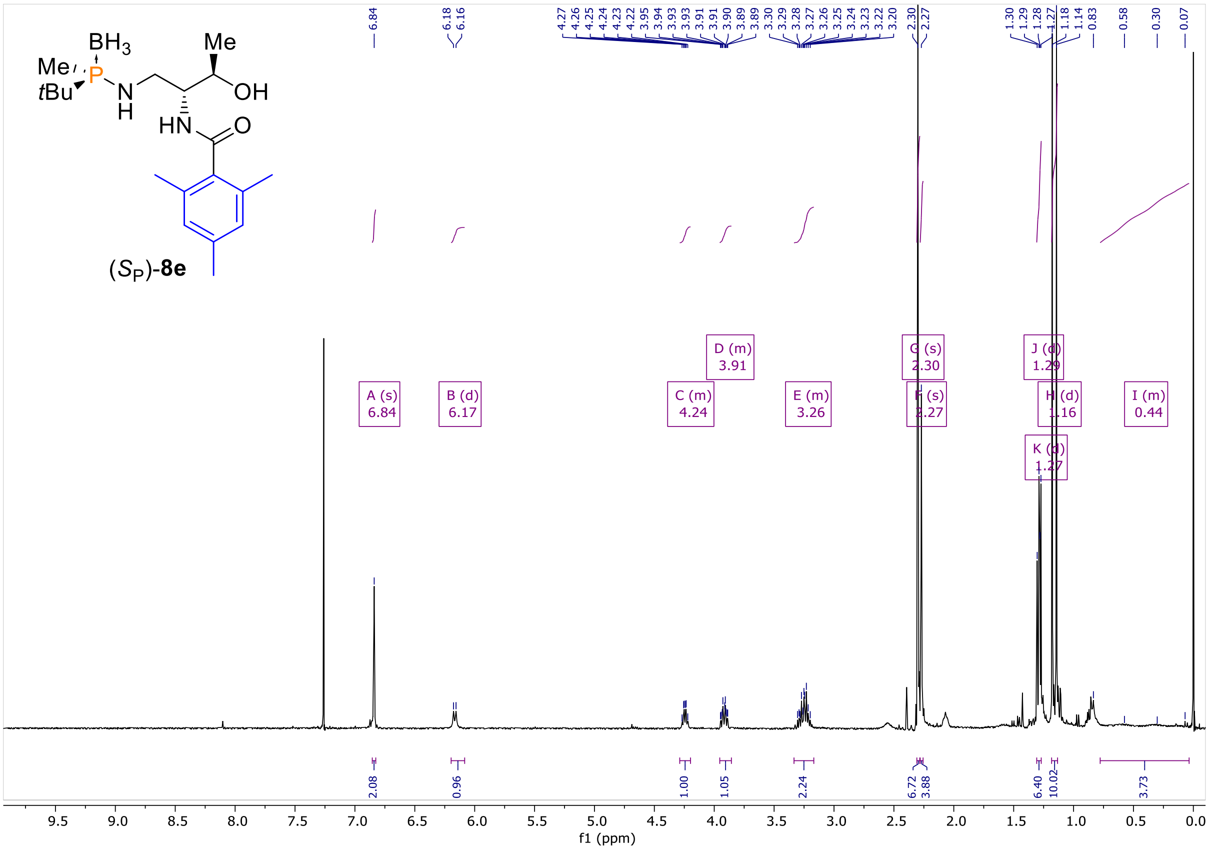

^{1}H-NMR (400 MHz, CDCl$_3$):

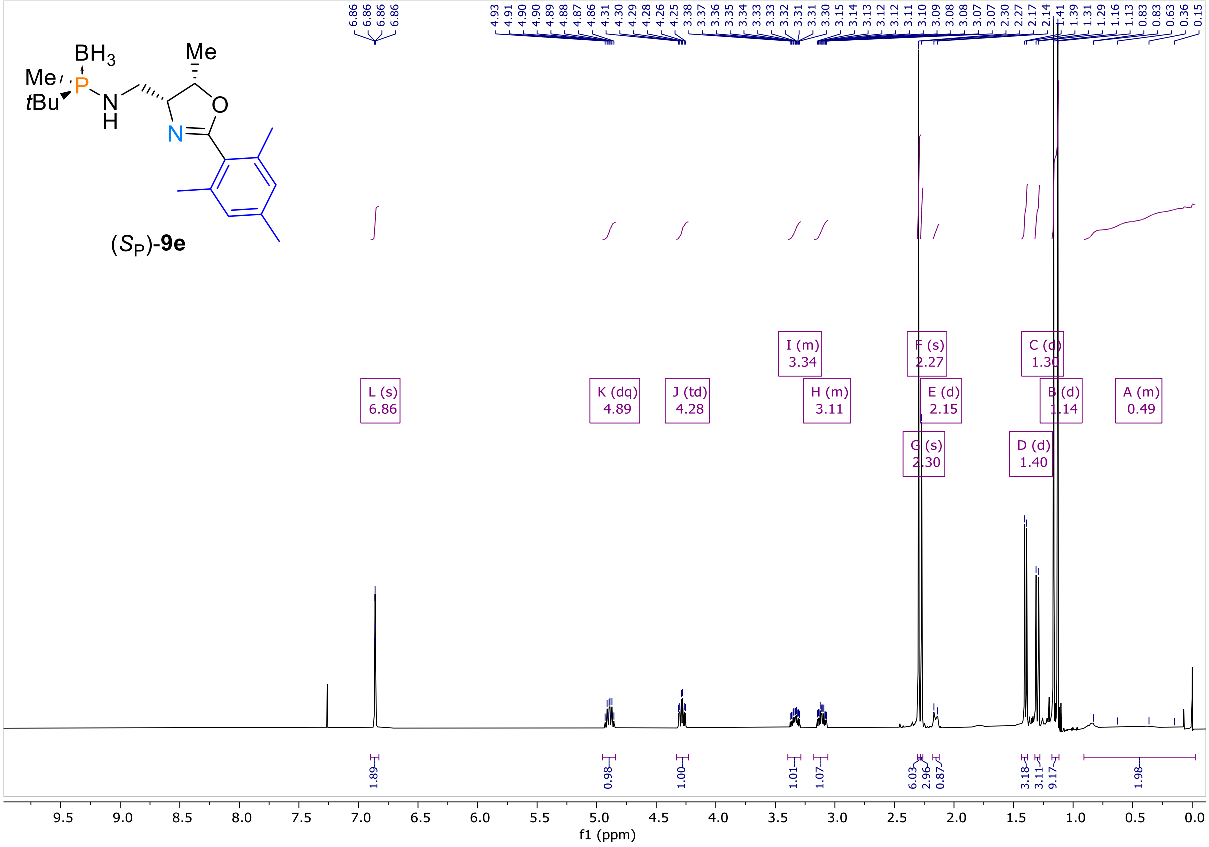

^{13}C-NMR (101 MHz, $CDCl_3$):

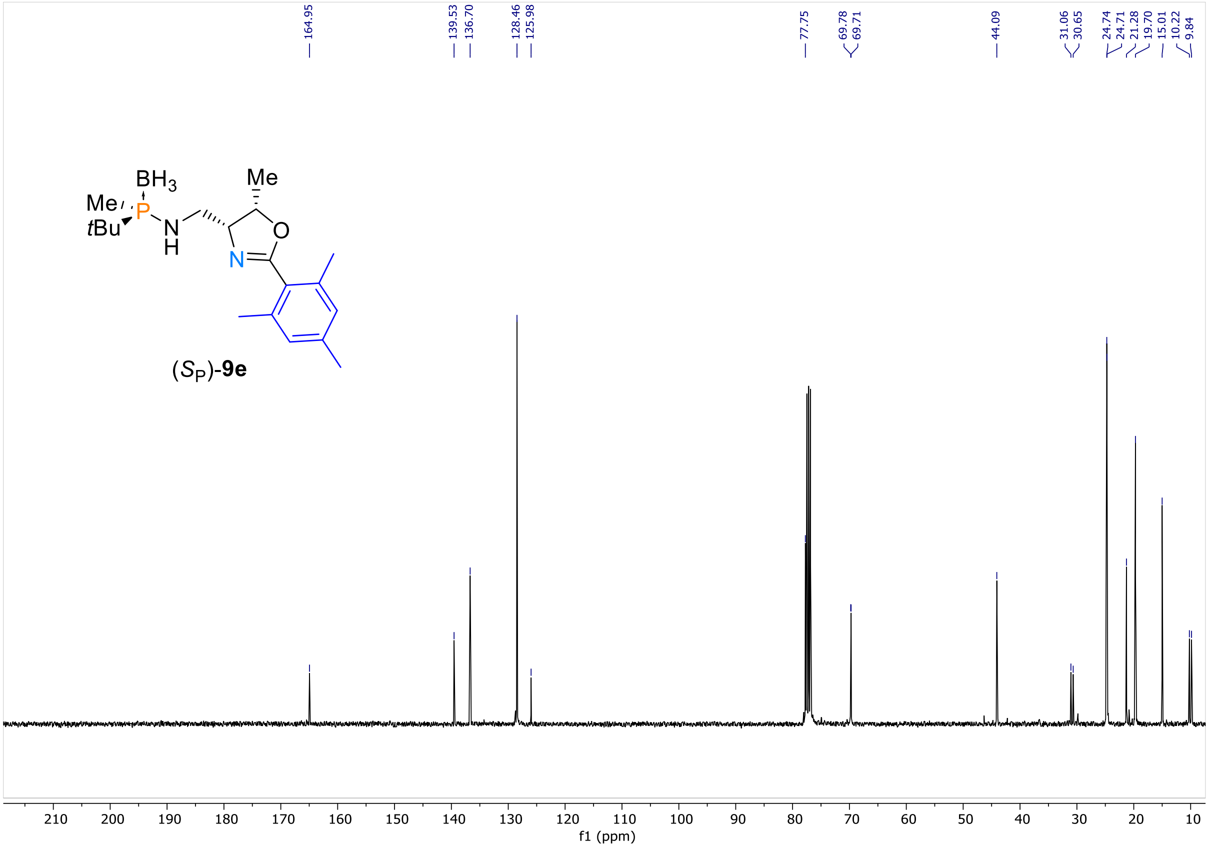

^{31}P-NMR (162 MHz, $CDCl_3$):

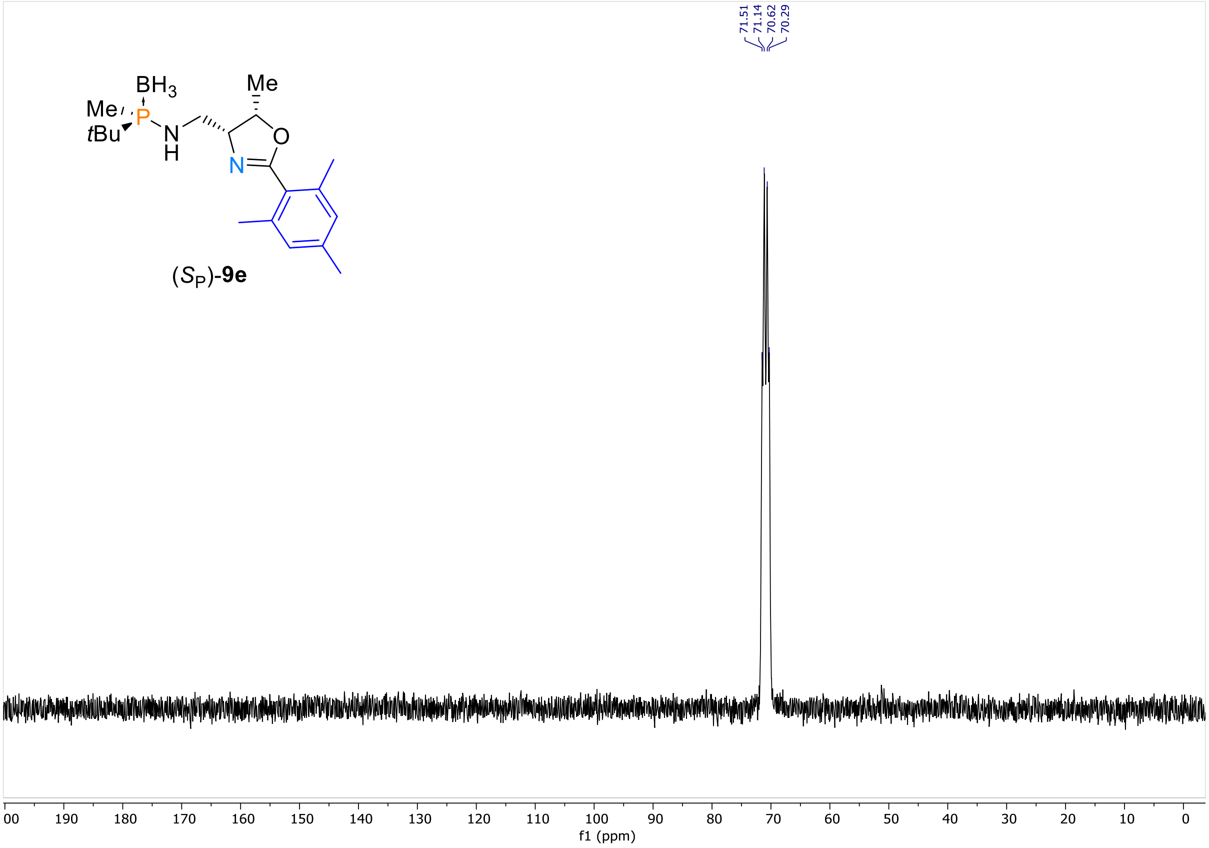

^{1}H-NMR (400 MHz, $CDCl_3$):

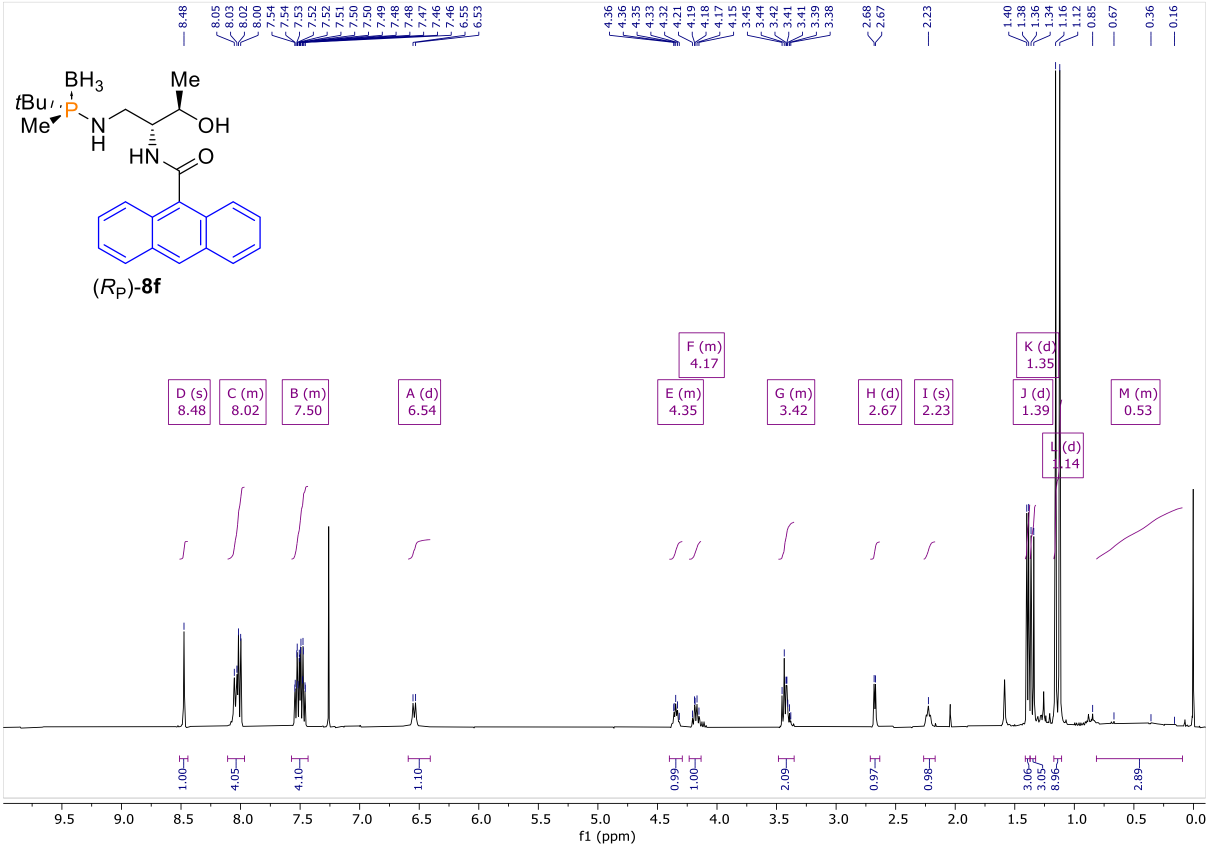

^{1}H-NMR (400 MHz, $CDCl_3$):

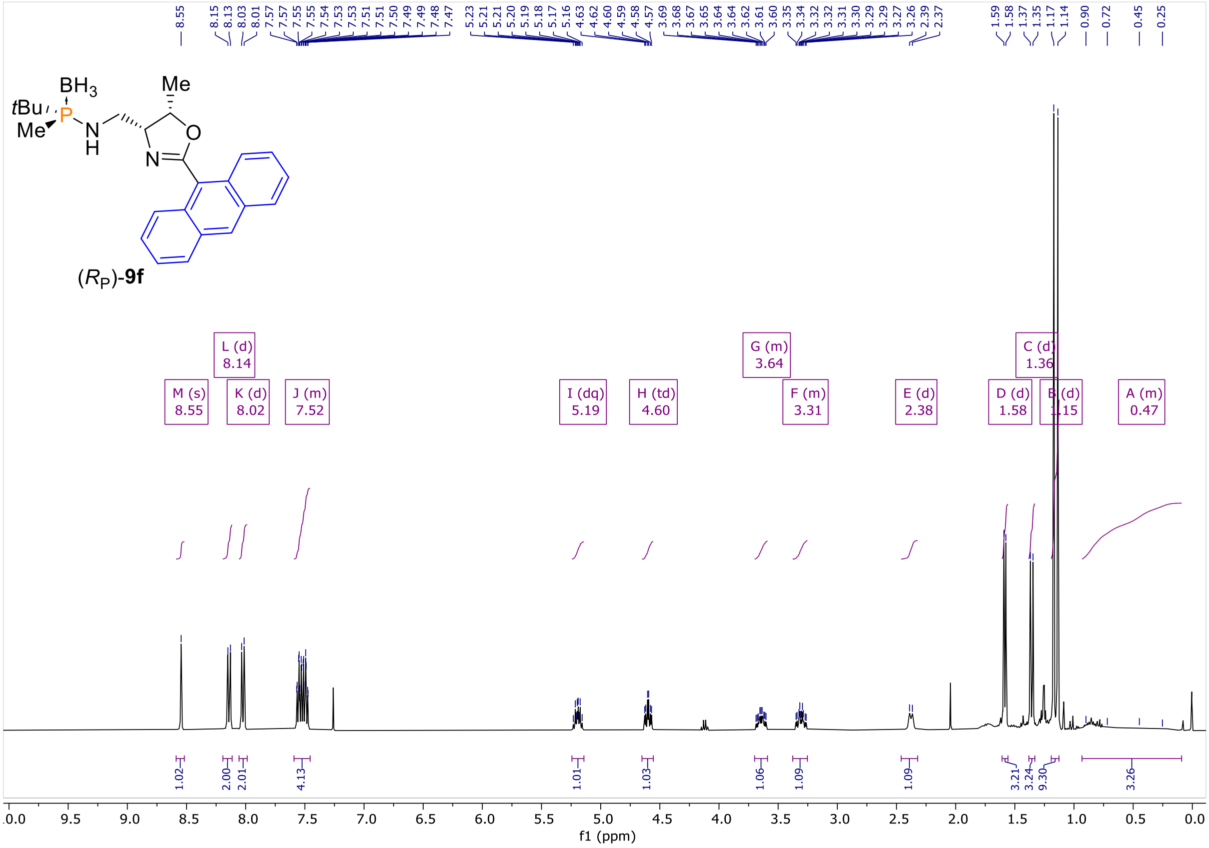

^{13}C-NMR (101 MHz, $CDCl_3$):

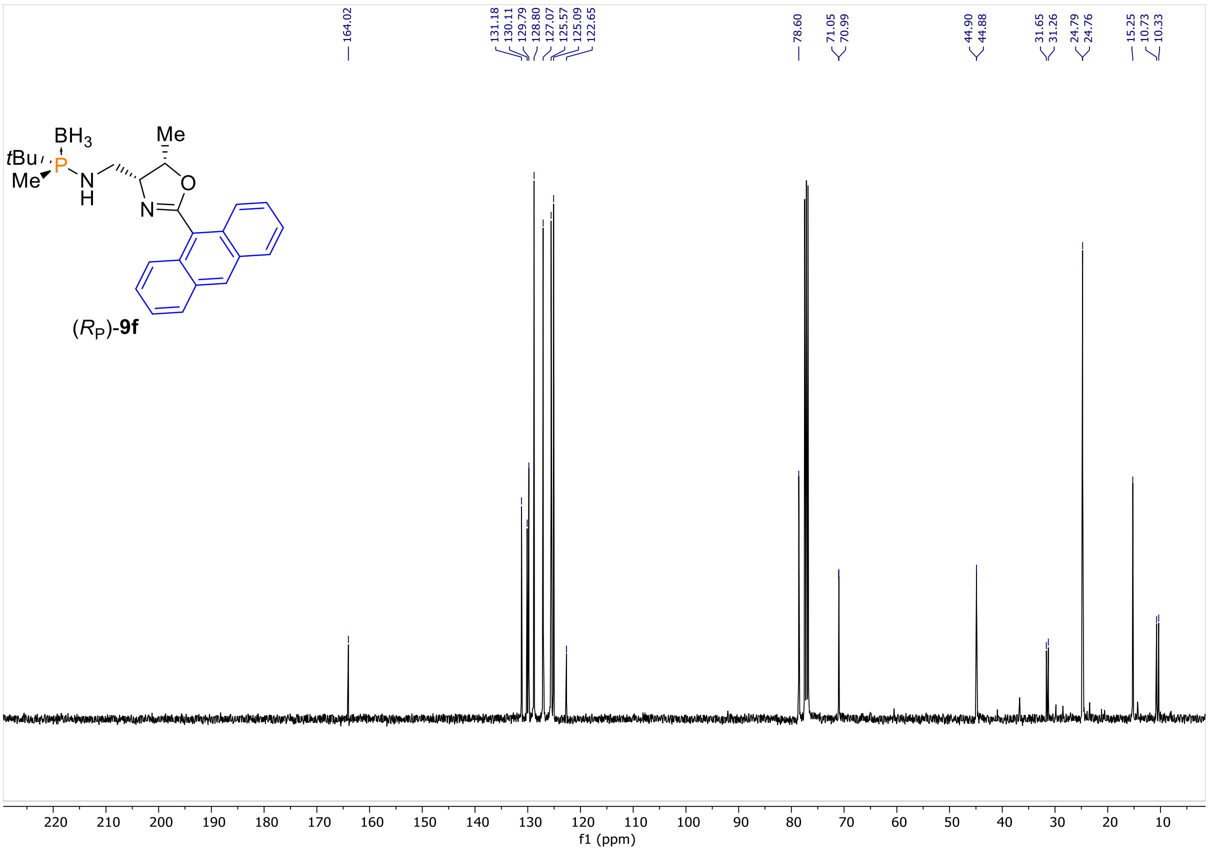

^{31}P-NMR (162 MHz, $CDCl_3$):

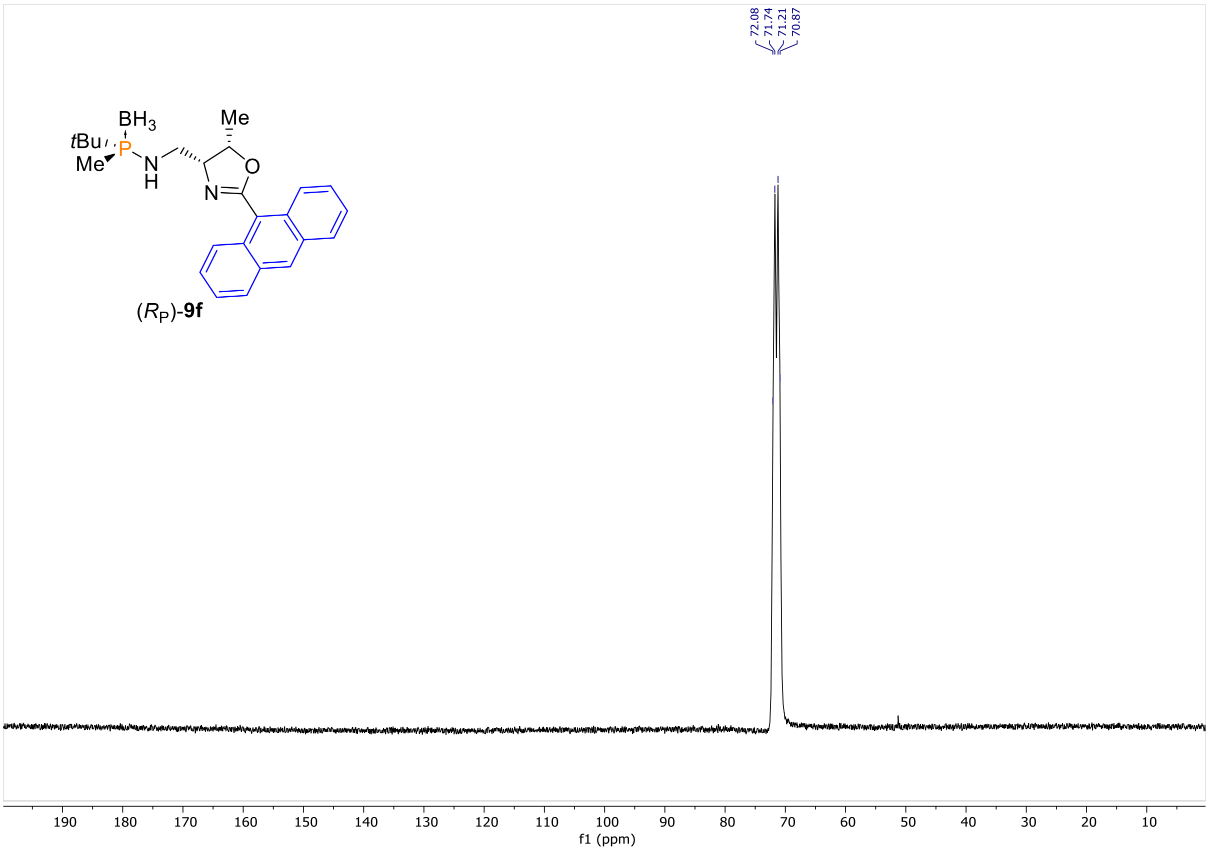

^{1}H-NMR (400 MHz, $CDCl_3$):

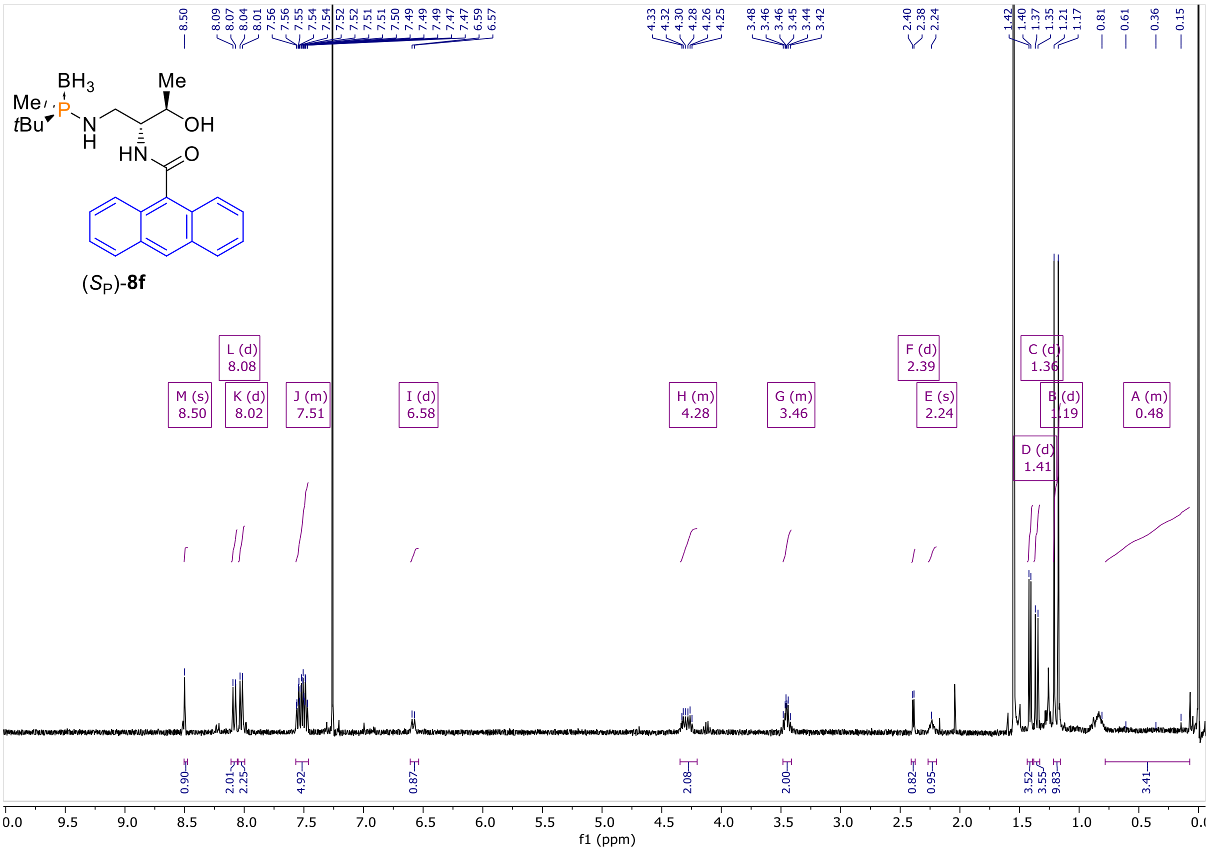

^{1}H-NMR (400 MHz, $CDCl_3$):

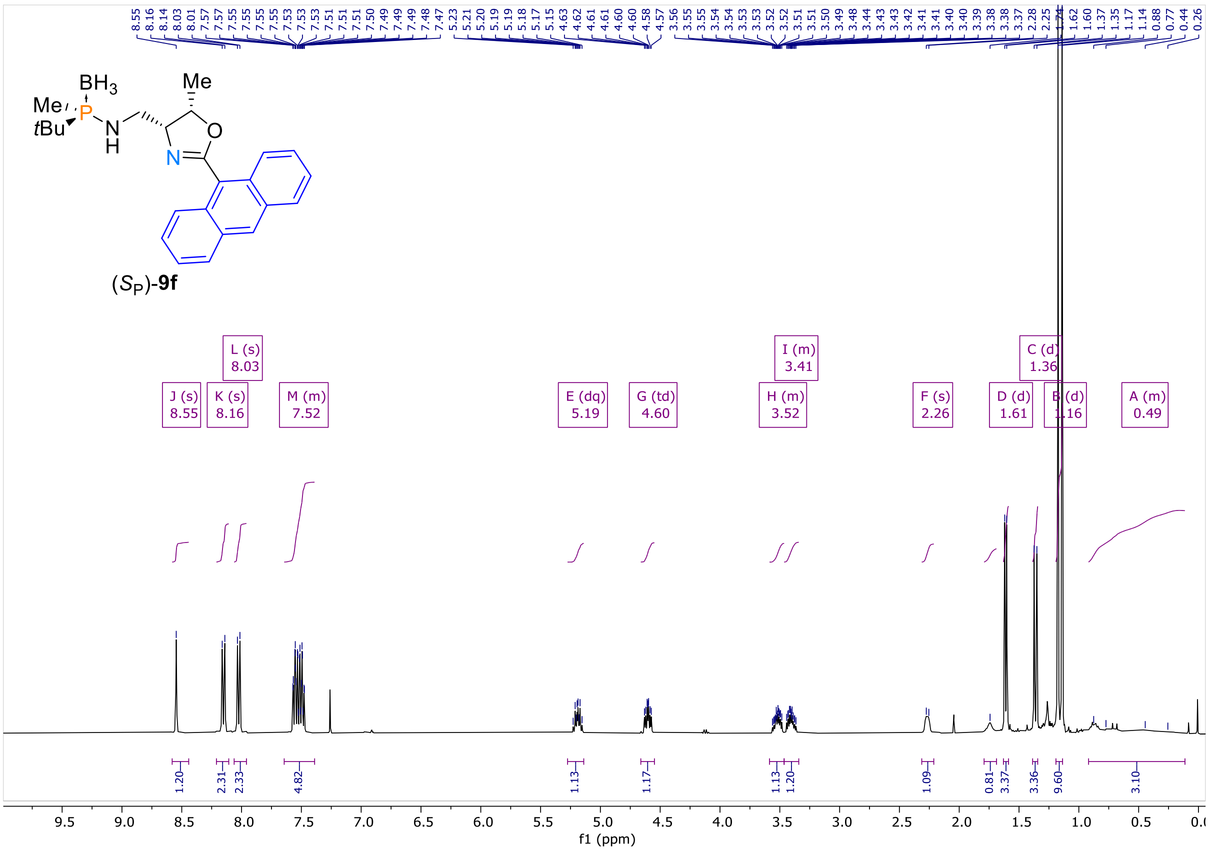

^{13}C-NMR (101 MHz, $CDCl_3$):

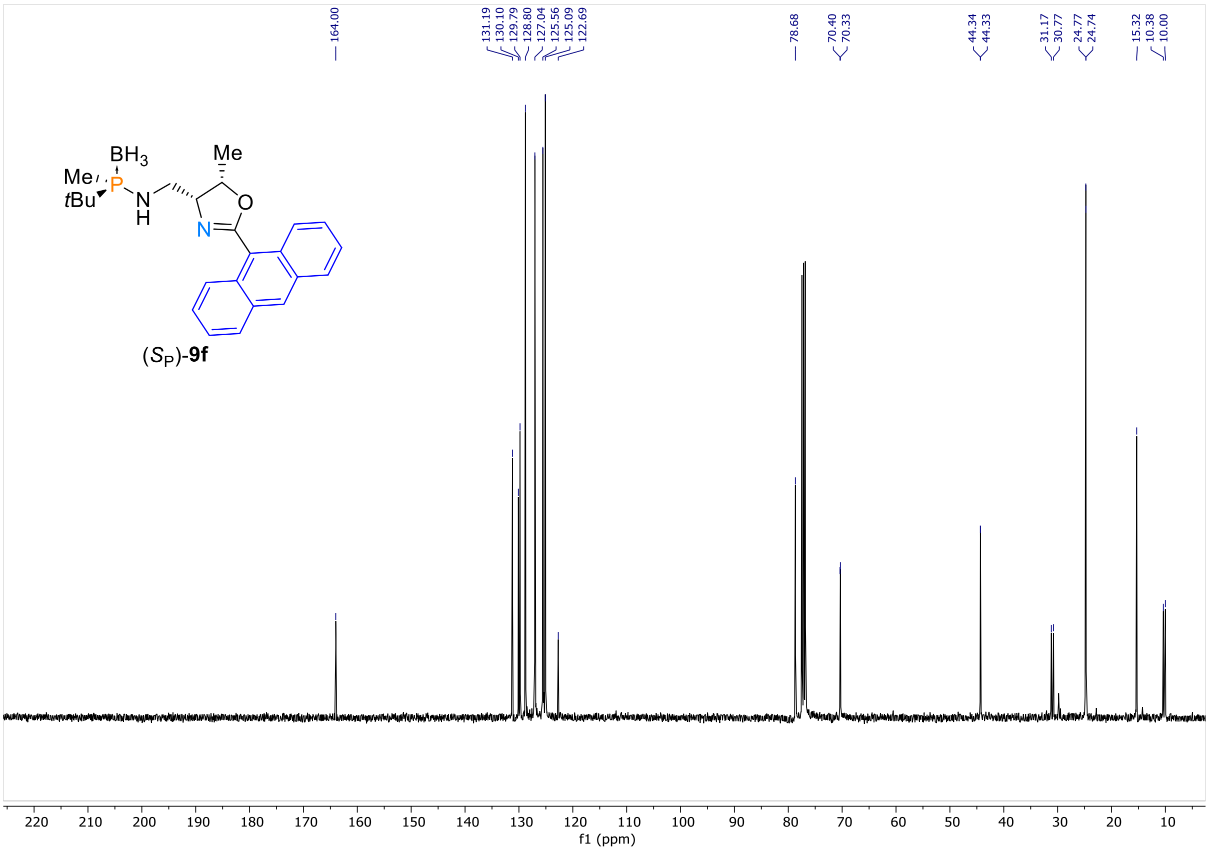

^{31}P-NMR (162 MHz, $CDCl_3$):

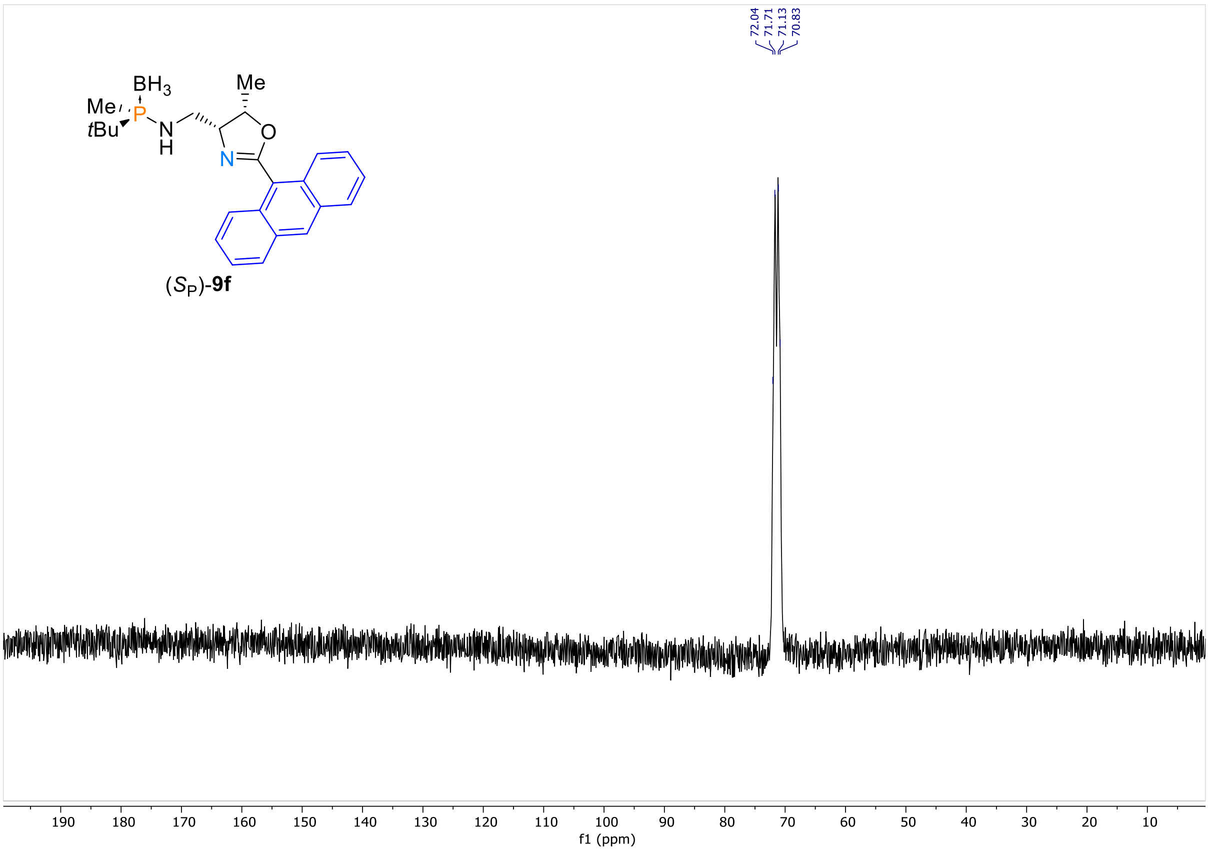

^{1}H-NMR (400 MHz, $CDCl_3$):

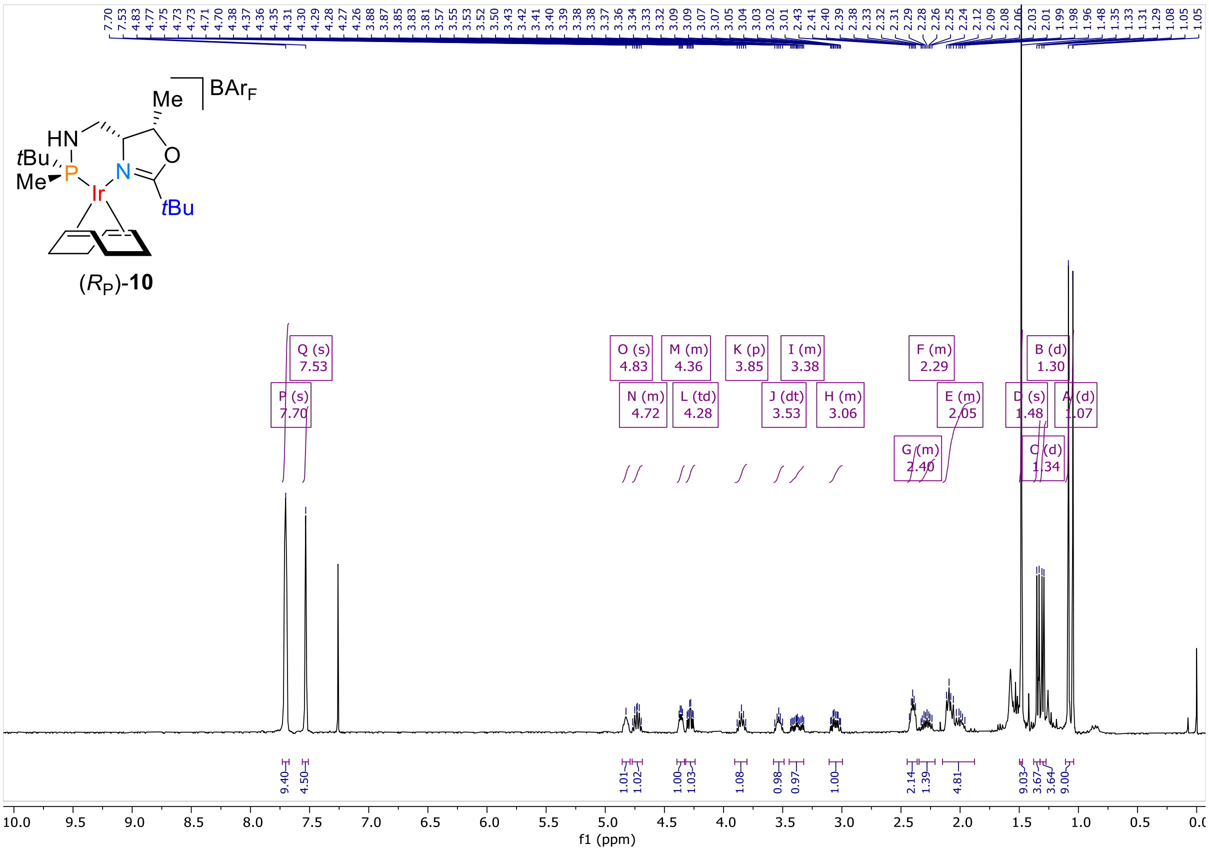

^{13}C-NMR (101 MHz, $CDCl_3$):

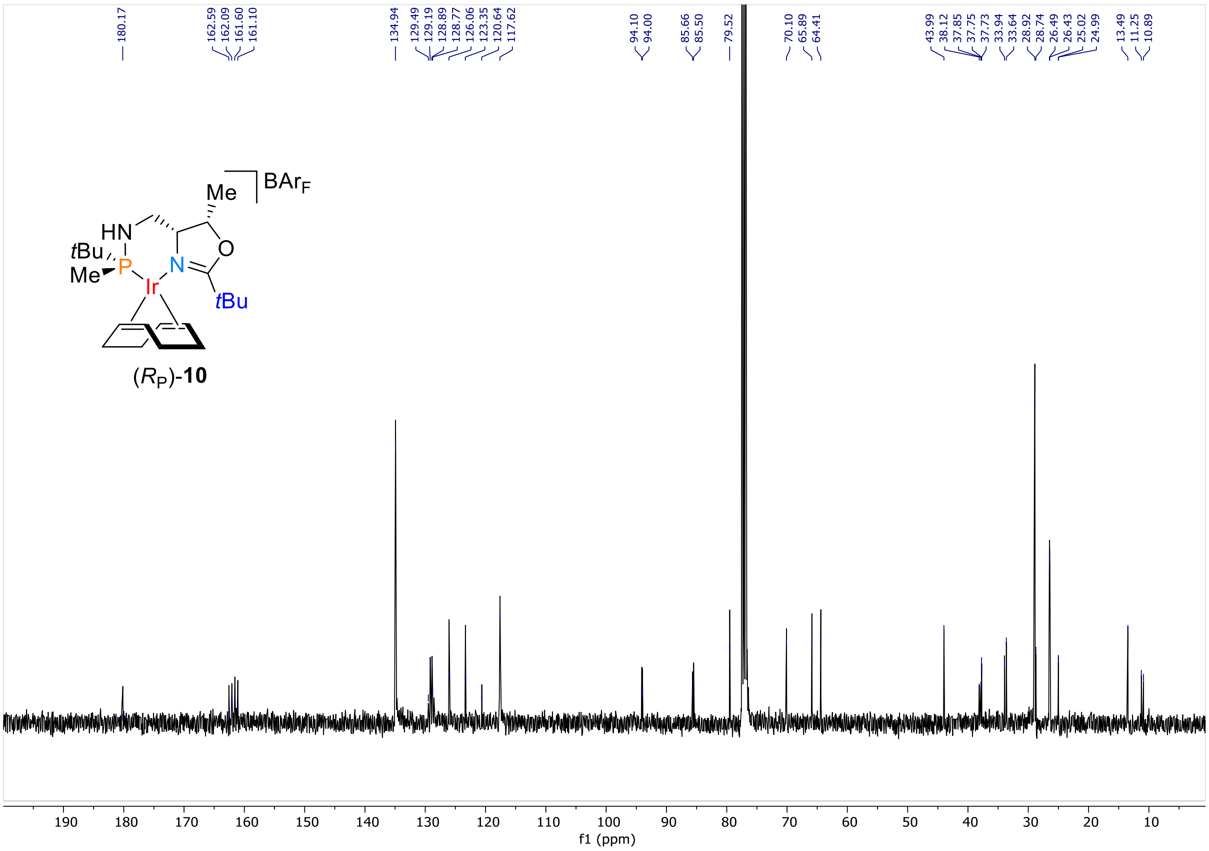

^{31}P-NMR (162 MHz, $CDCl_3$):

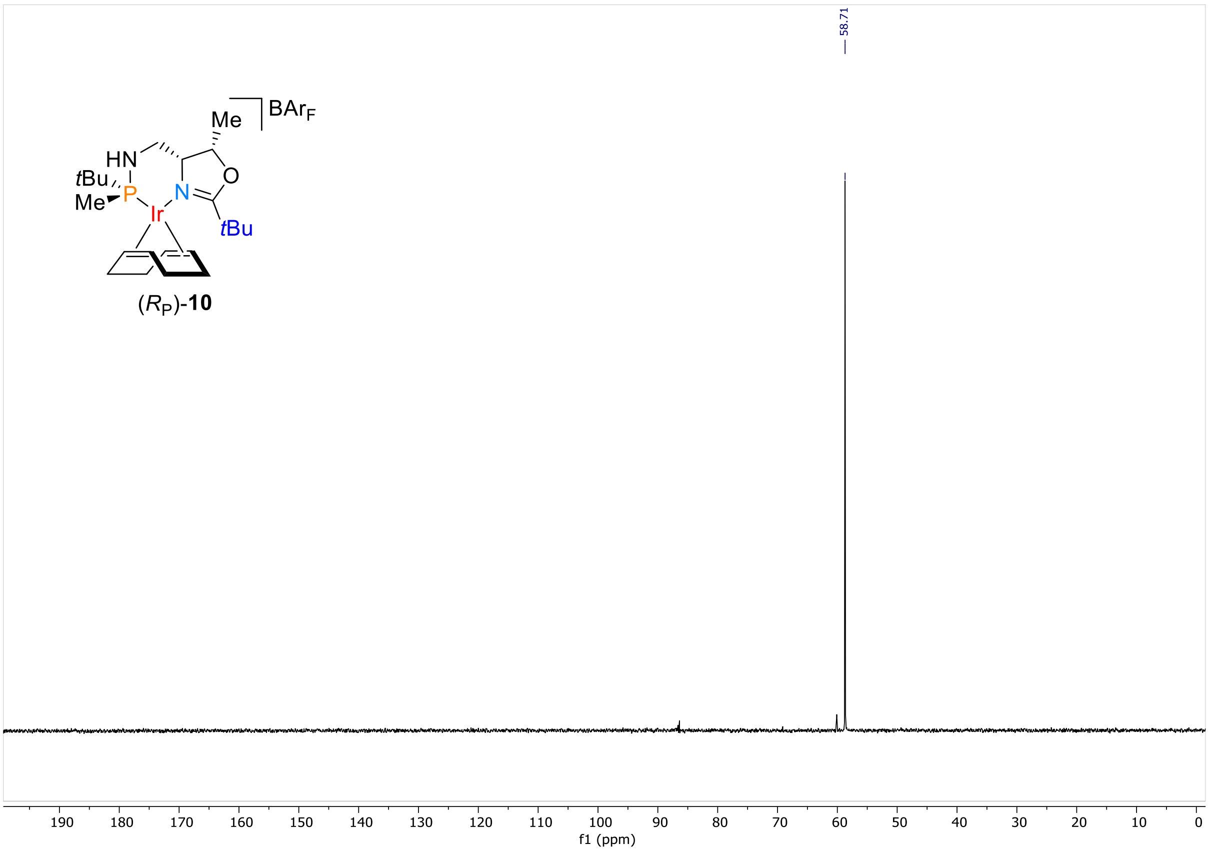

^{1}H-NMR (400 MHz, $CDCl_3$):

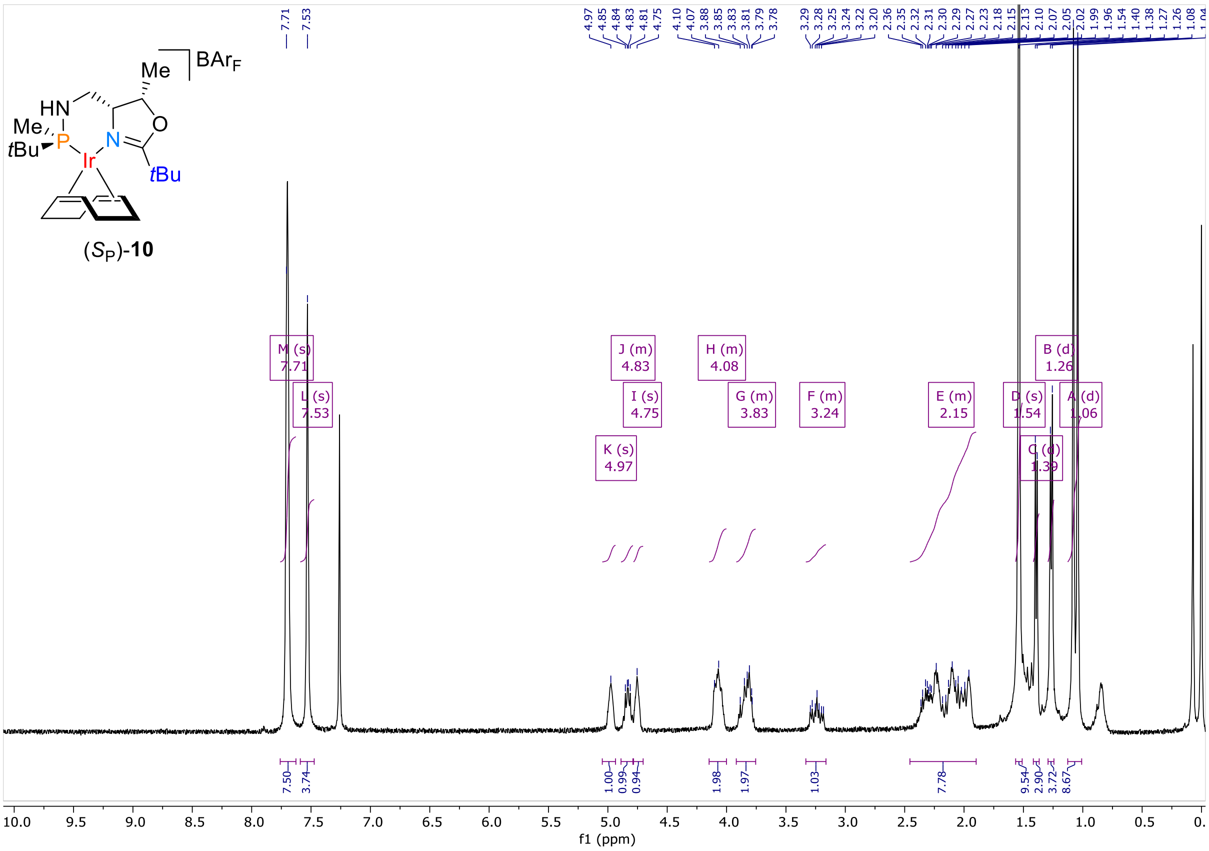

^{13}C-NMR (101 MHz, $CDCl_3$):

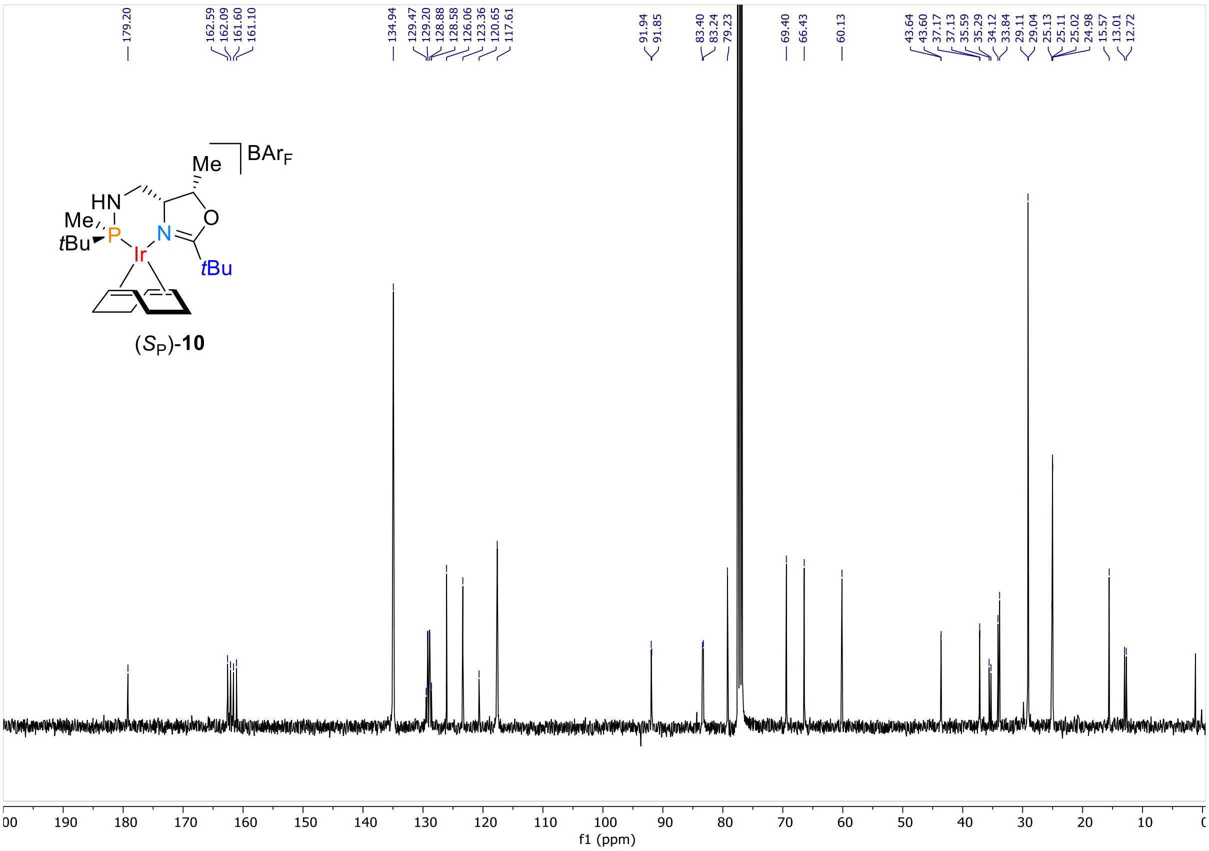

^{31}P-NMR (162 MHz, $CDCl_3$):

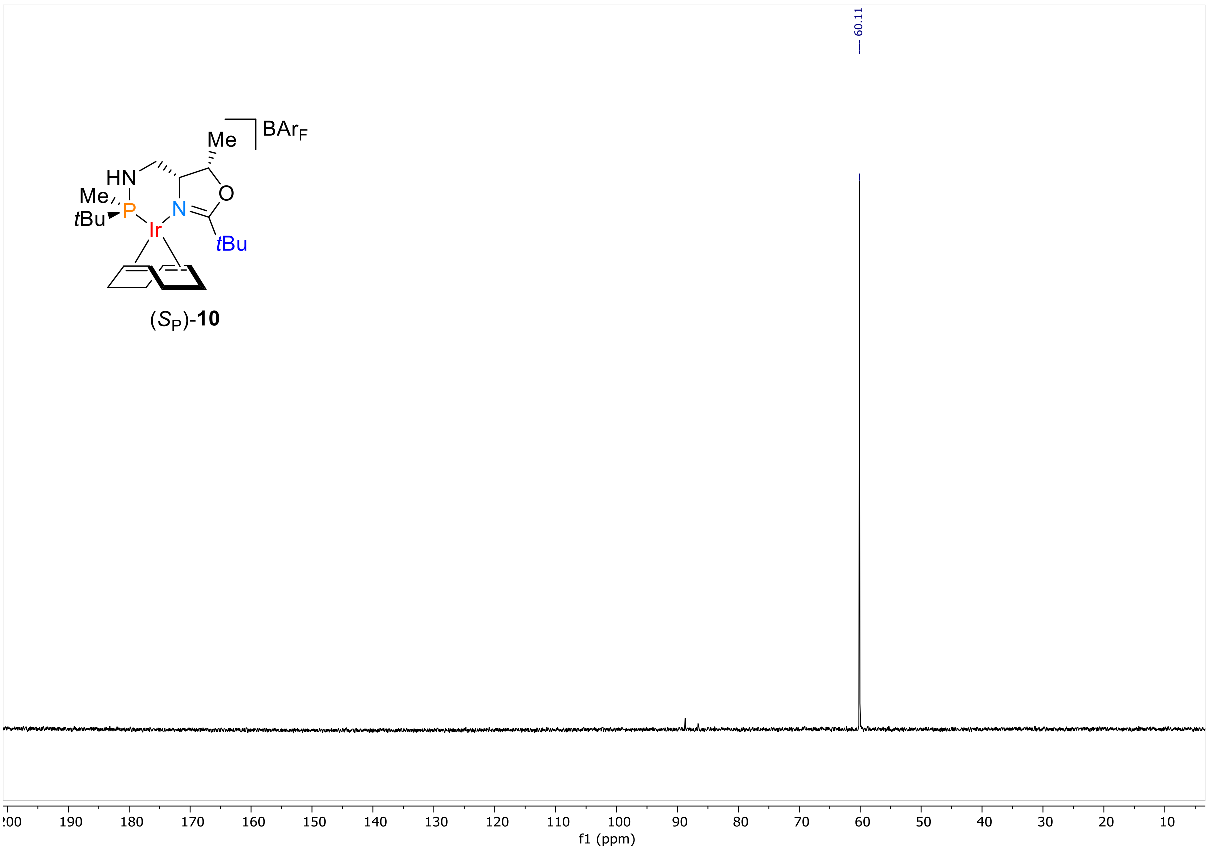

^{1}H-NMR (400 MHz, $CDCl_3$):

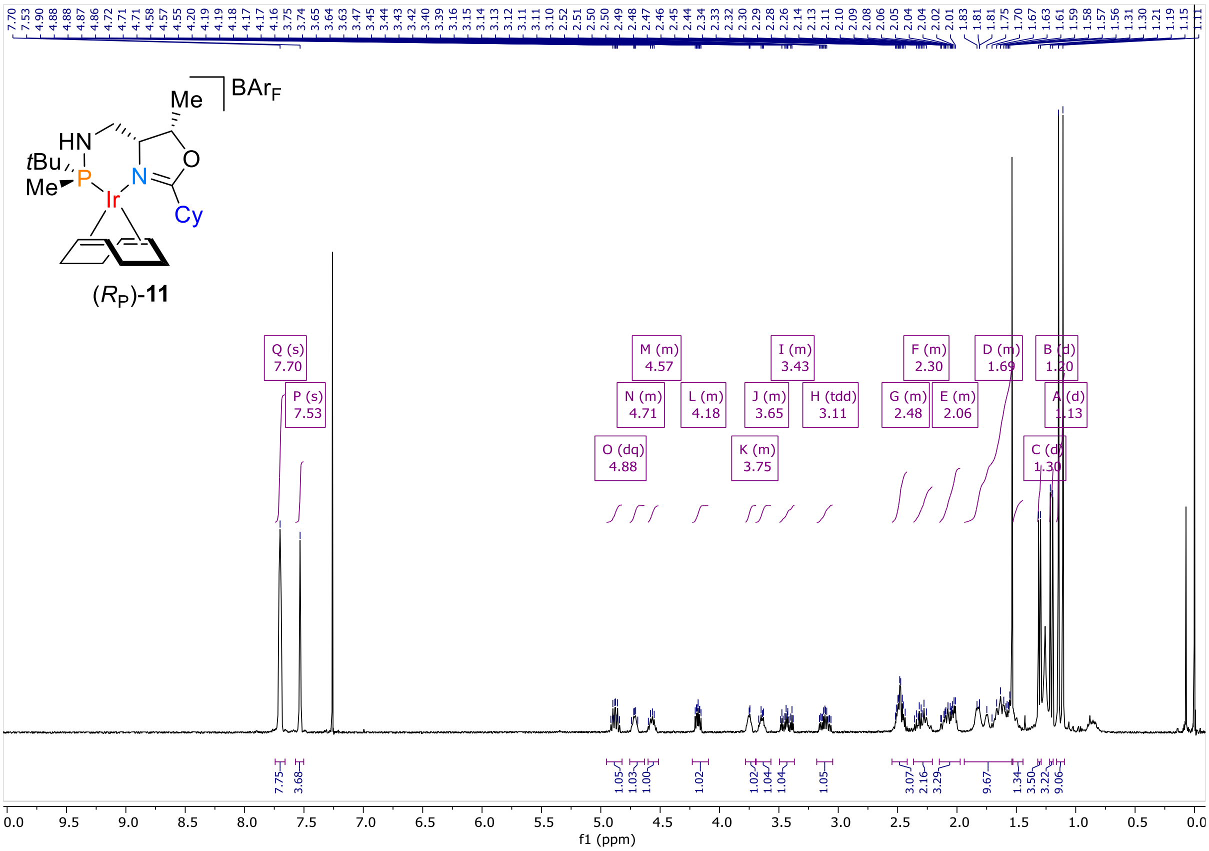

^{13}C-NMR (101 MHz, $CDCl_3$):

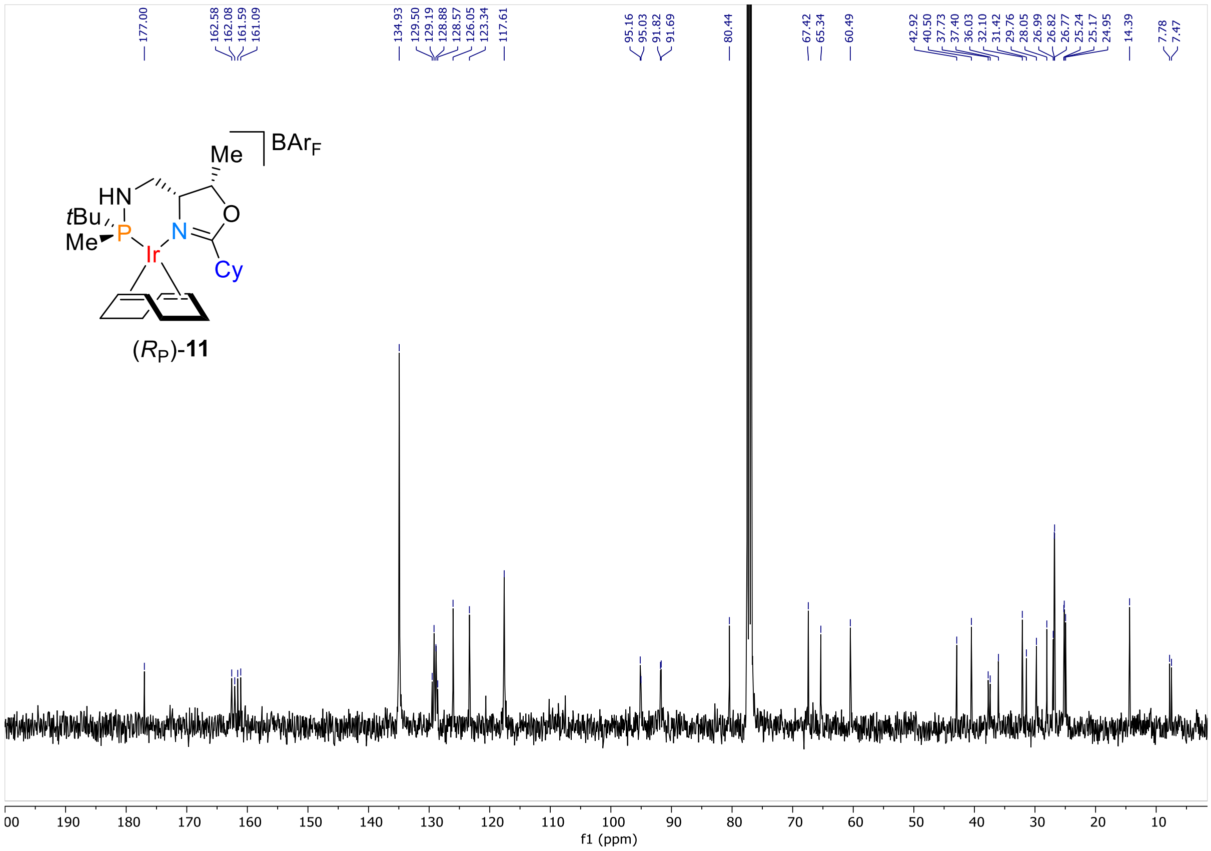

^{31}P-NMR (162 MHz, $CDCl_3$):

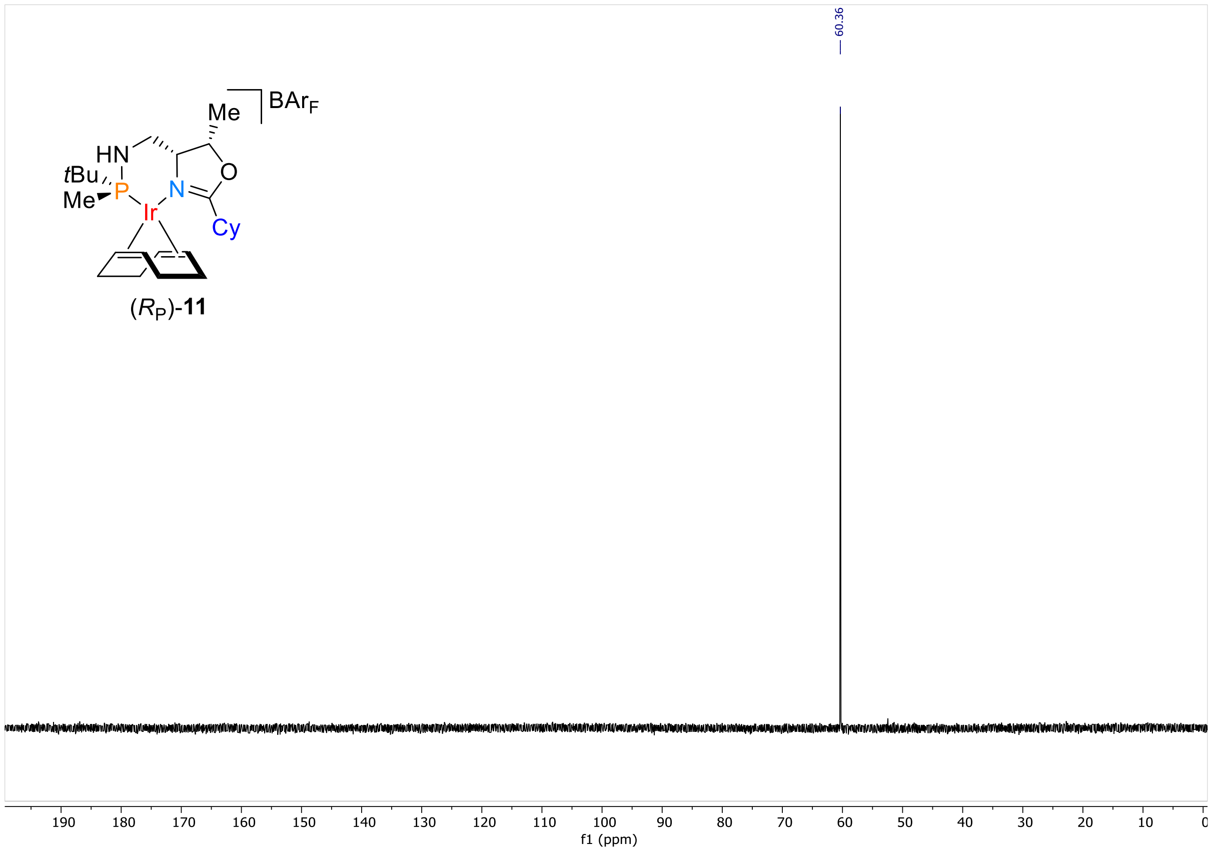

^{1}H-NMR (400 MHz, $CDCl_3$):

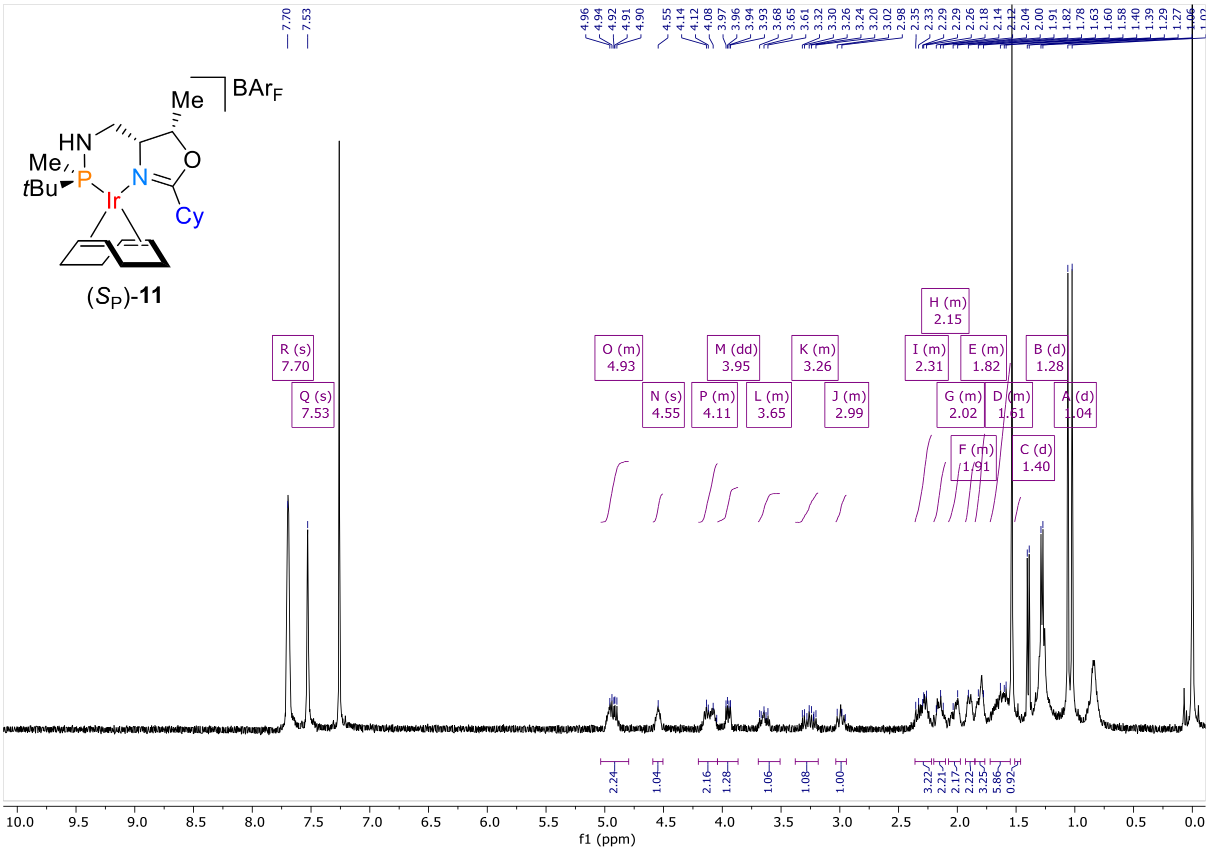

^{13}C-NMR (101 MHz, $CDCl_3$):

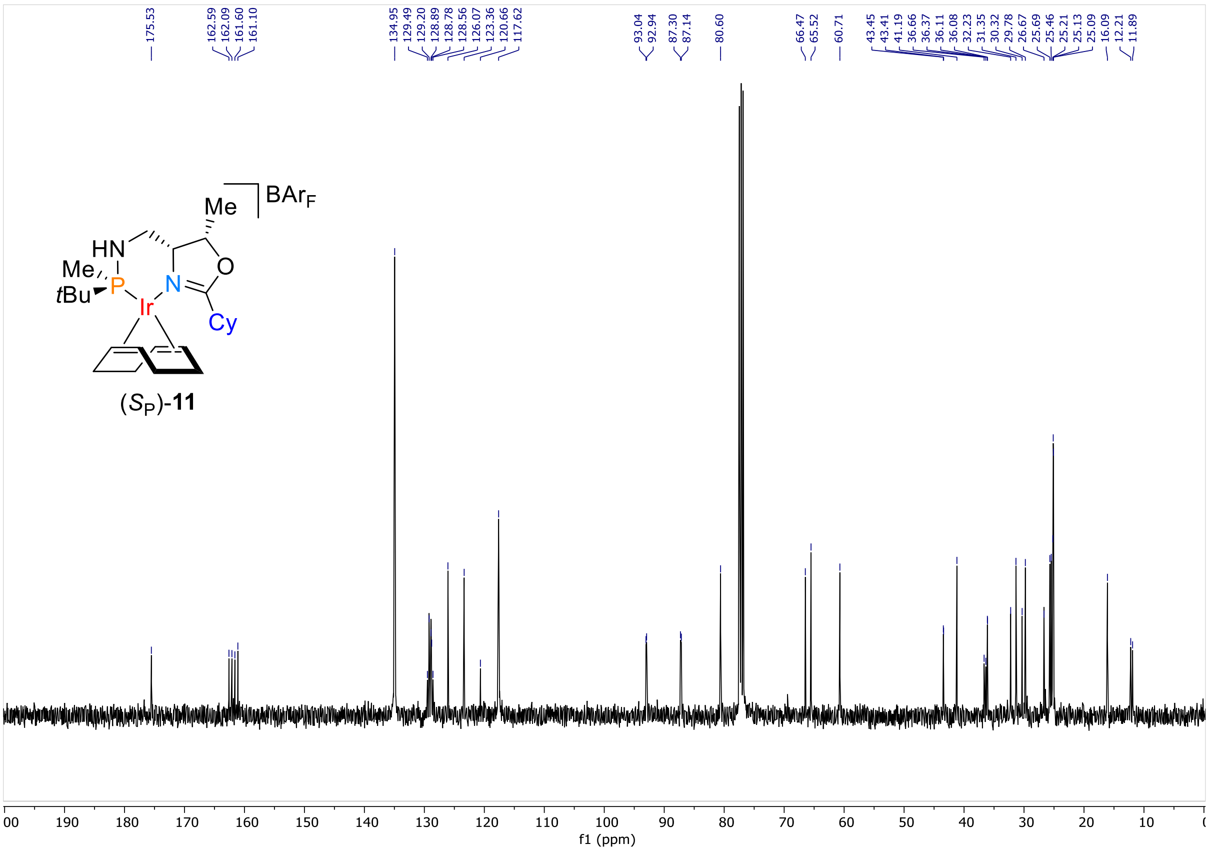

^{31}P-NMR (162 MHz, CDCl$_3$):

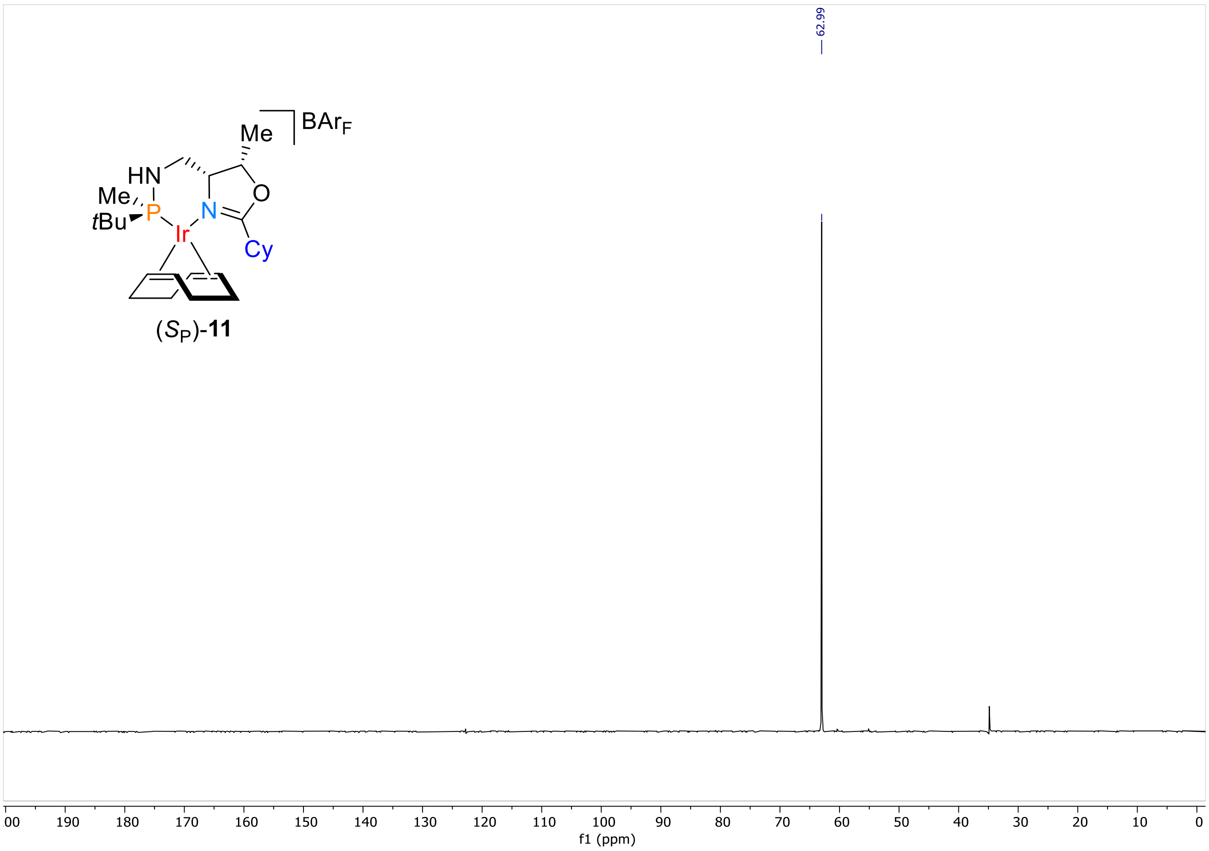

^{1}H-NMR (400 MHz, CDCl$_3$):

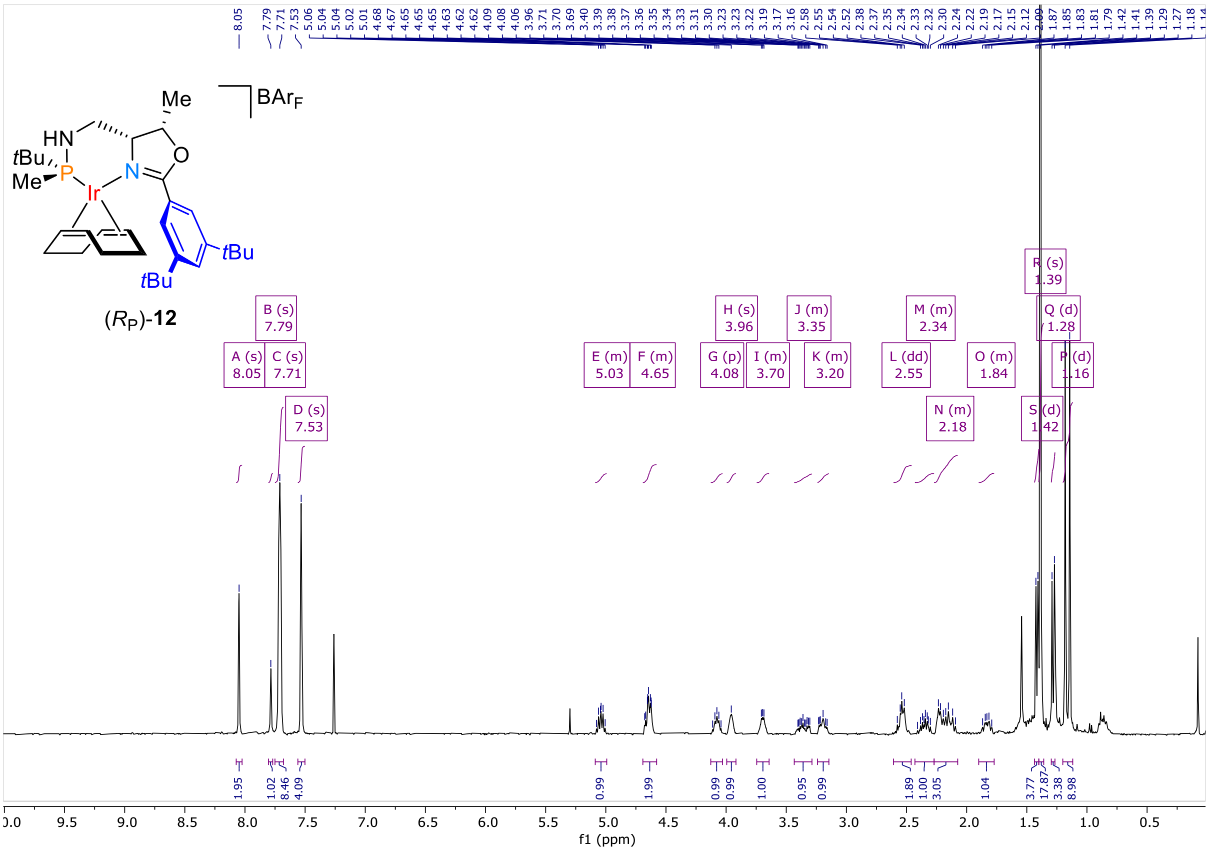

^{13}C-NMR (101 MHz, $CDCl_3$):

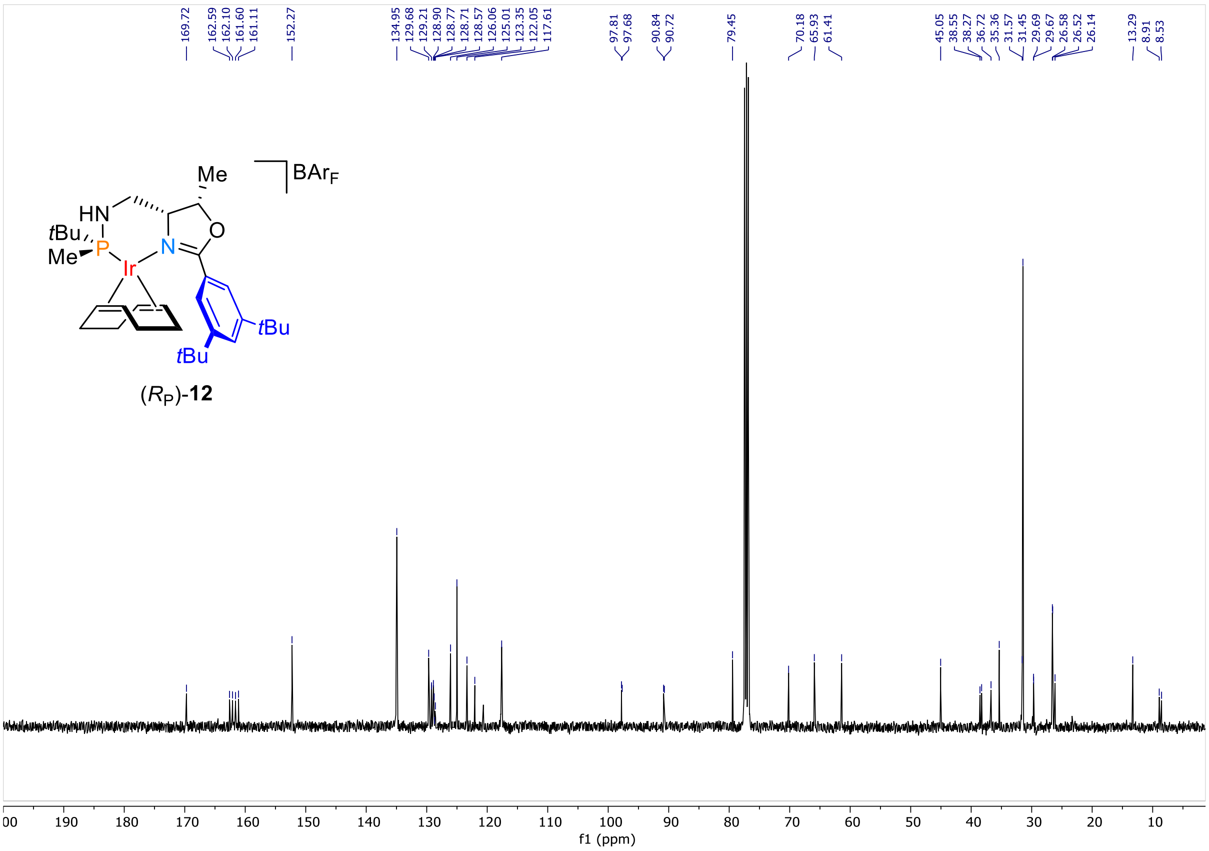

^{31}P-NMR (162 MHz, $CDCl_3$):

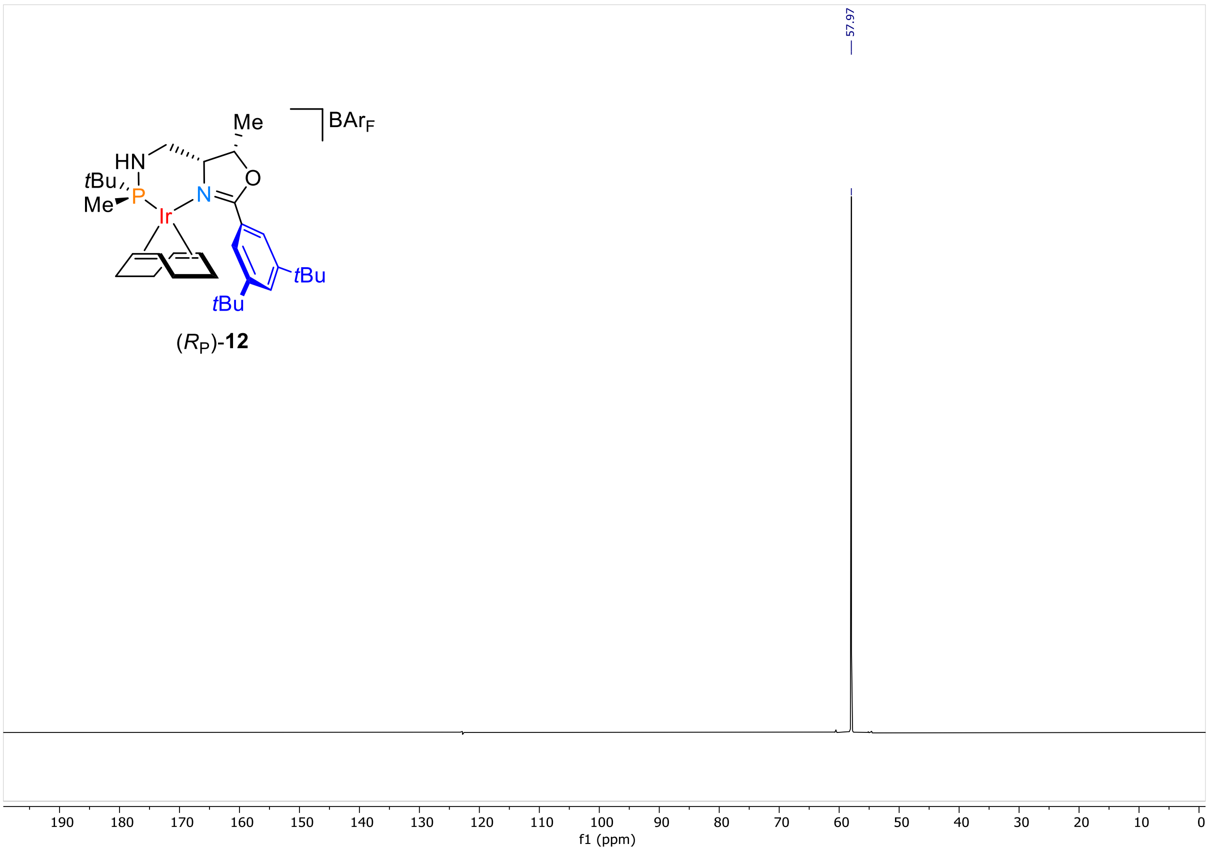

^{1}H-NMR (400 MHz, $CDCl_3$):

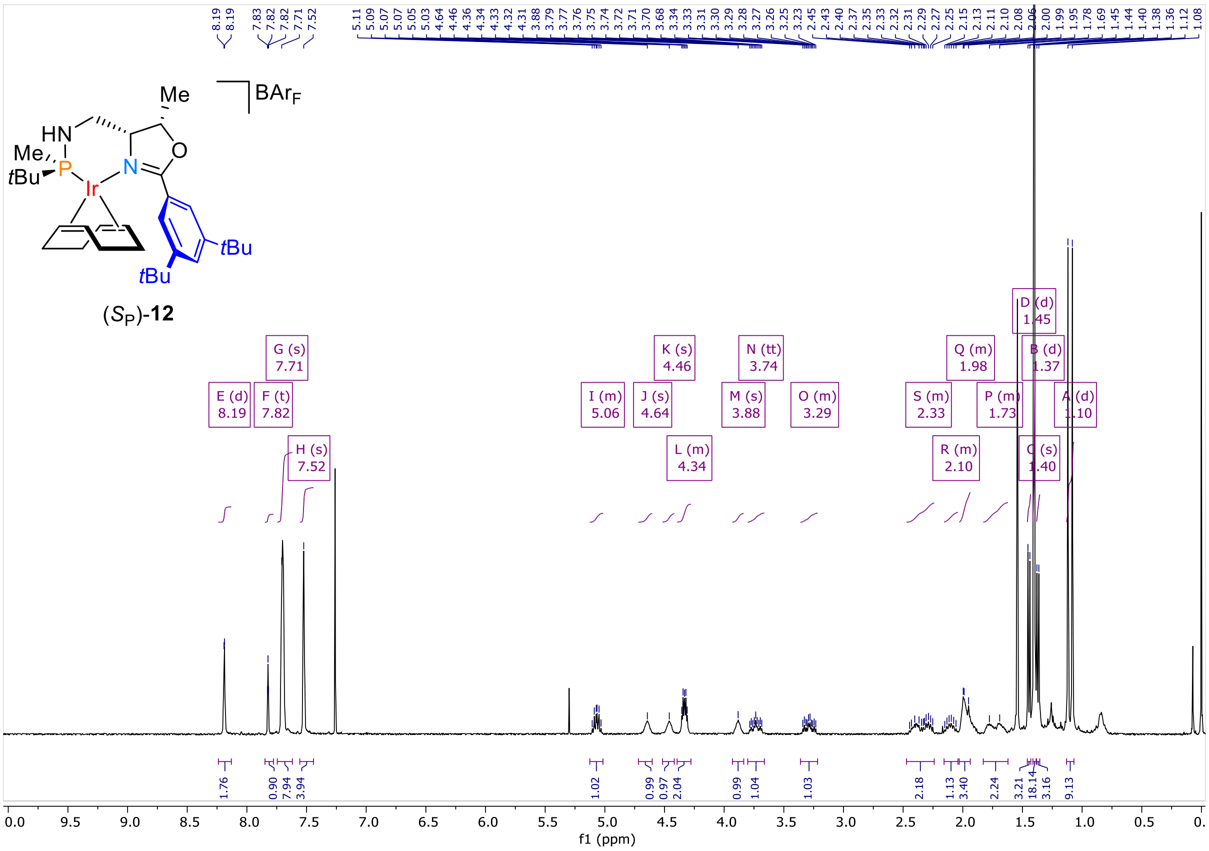

^{13}C-NMR (101 MHz, $CDCl_3$):

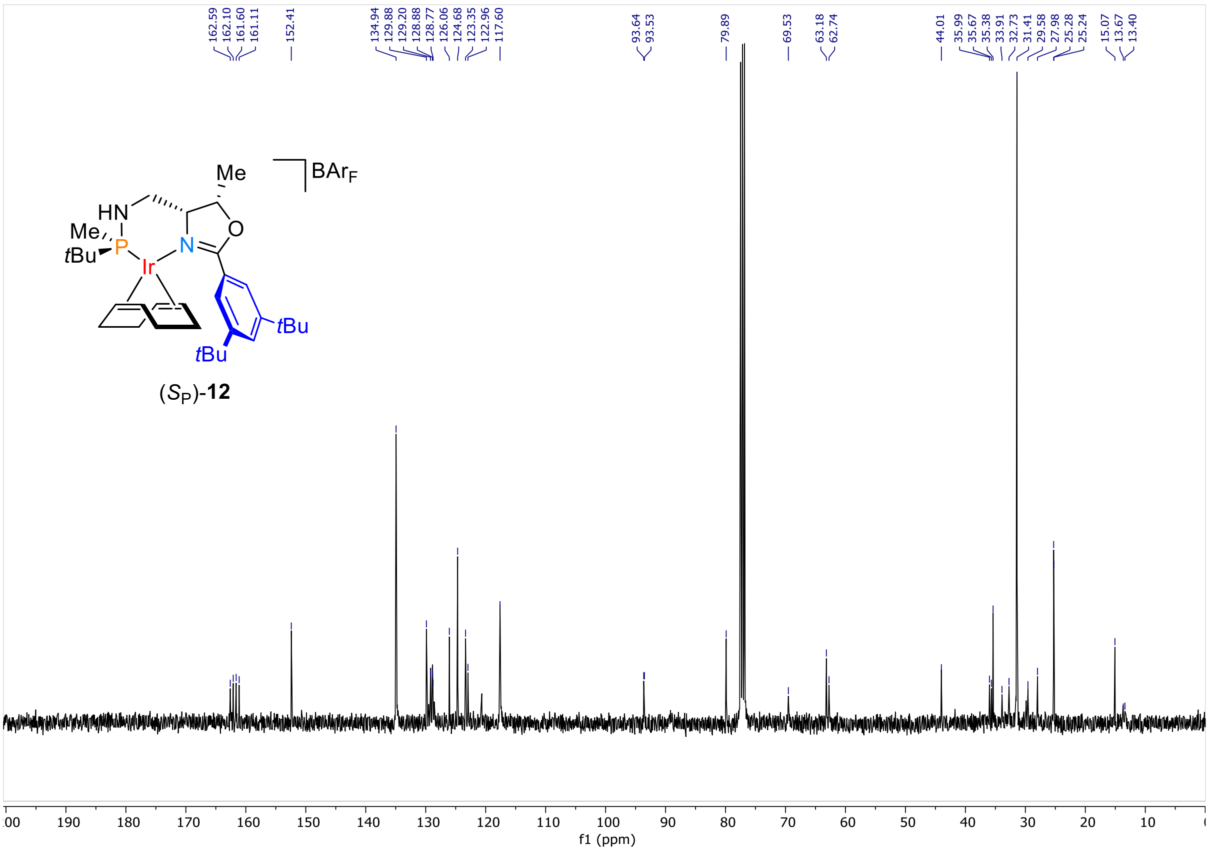

^{31}P-NMR (162 MHz, $CDCl_3$):

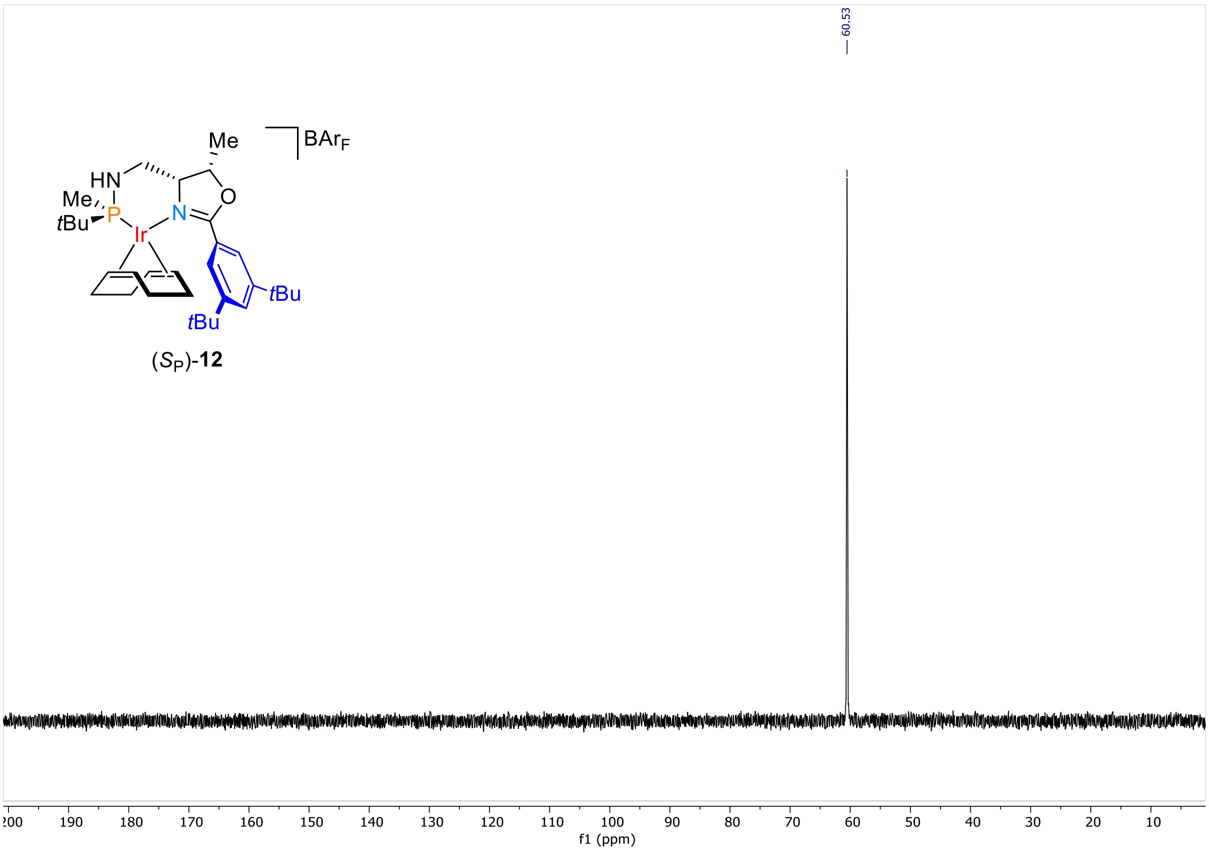

^{1}H-NMR (400 MHz, $CDCl_3$):

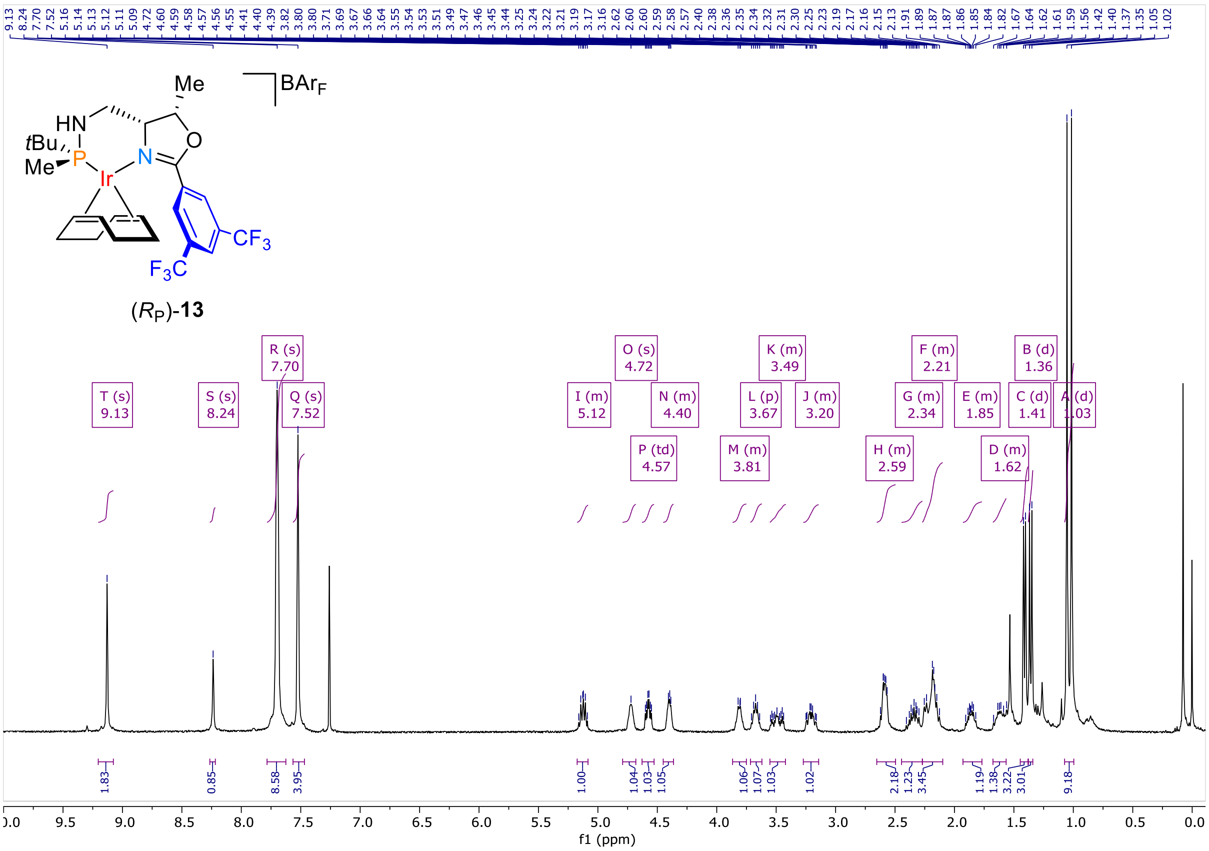

^{13}C-NMR (101 MHz, $CDCl_3$):

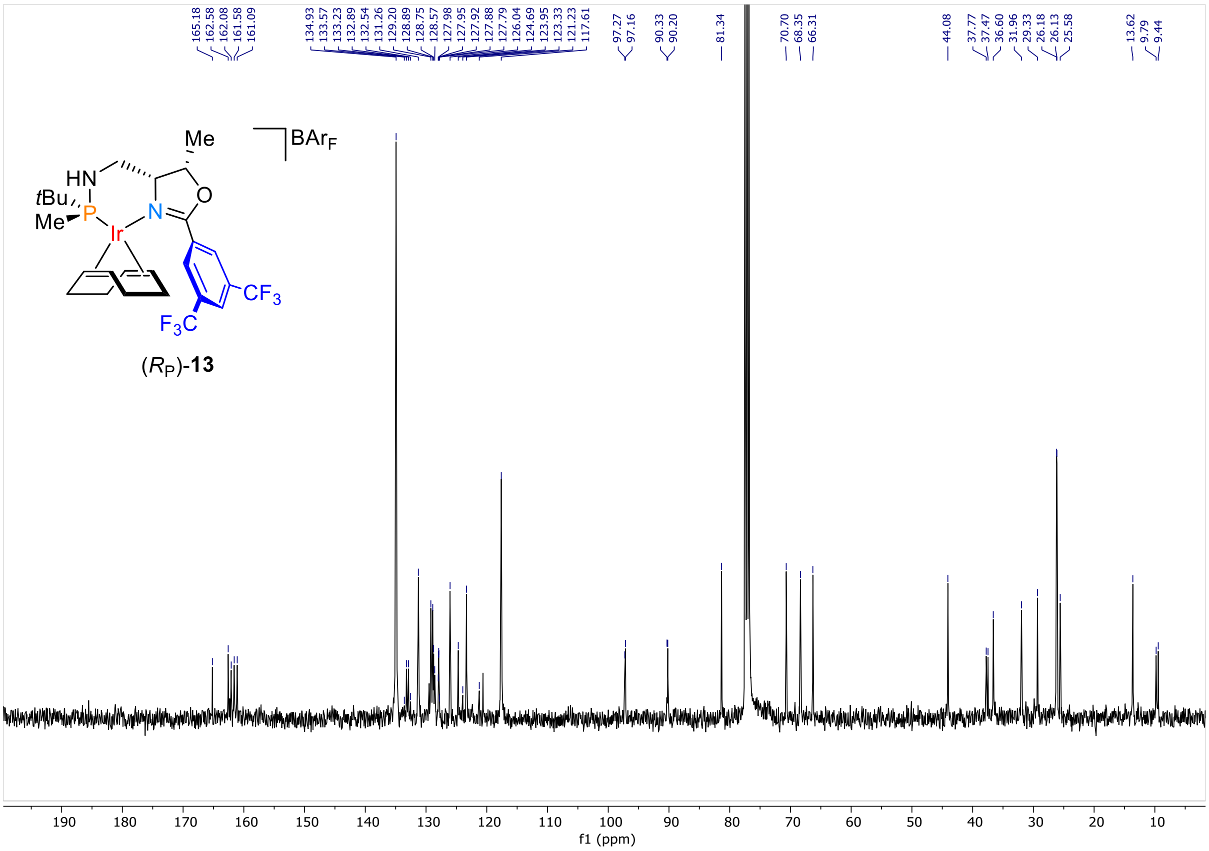

^{31}P-NMR (162 MHz, $CDCl_3$):

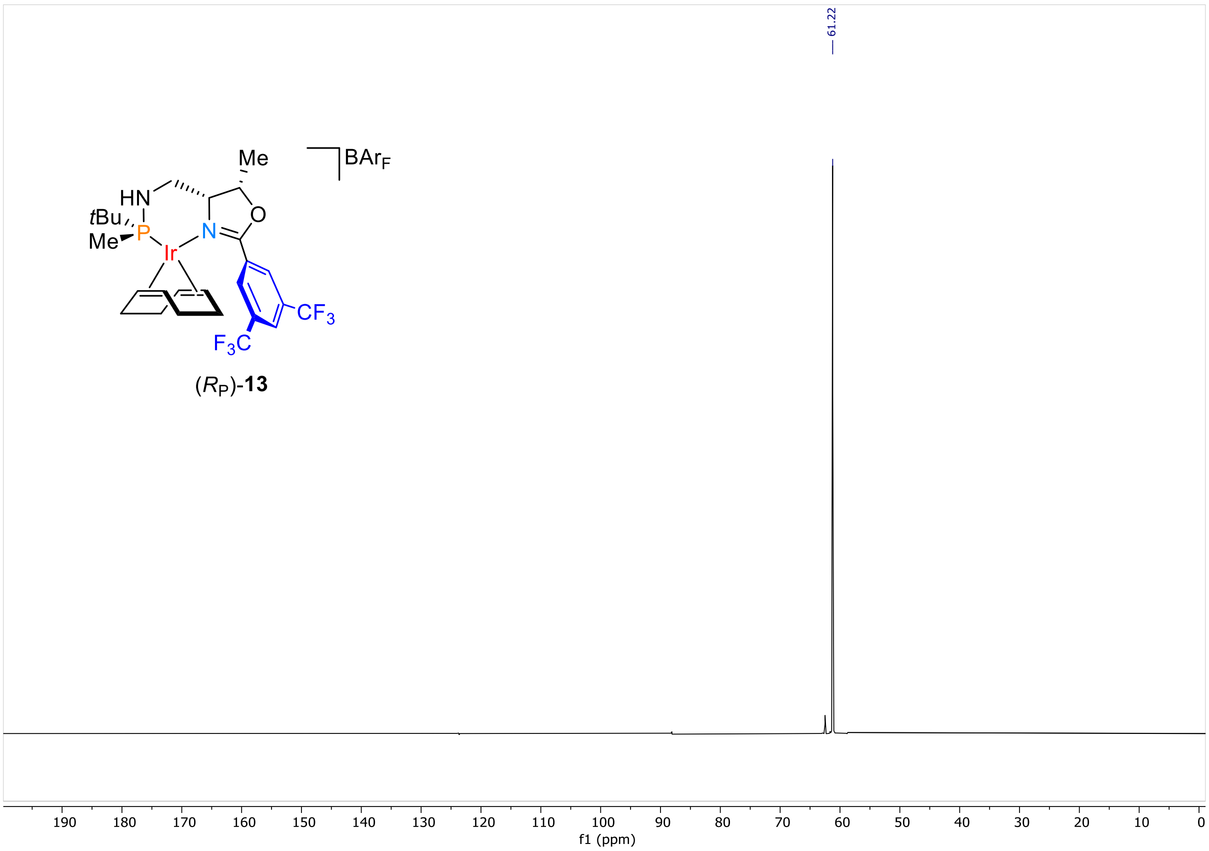

^{1}H-NMR (400 MHz, $CDCl_3$):

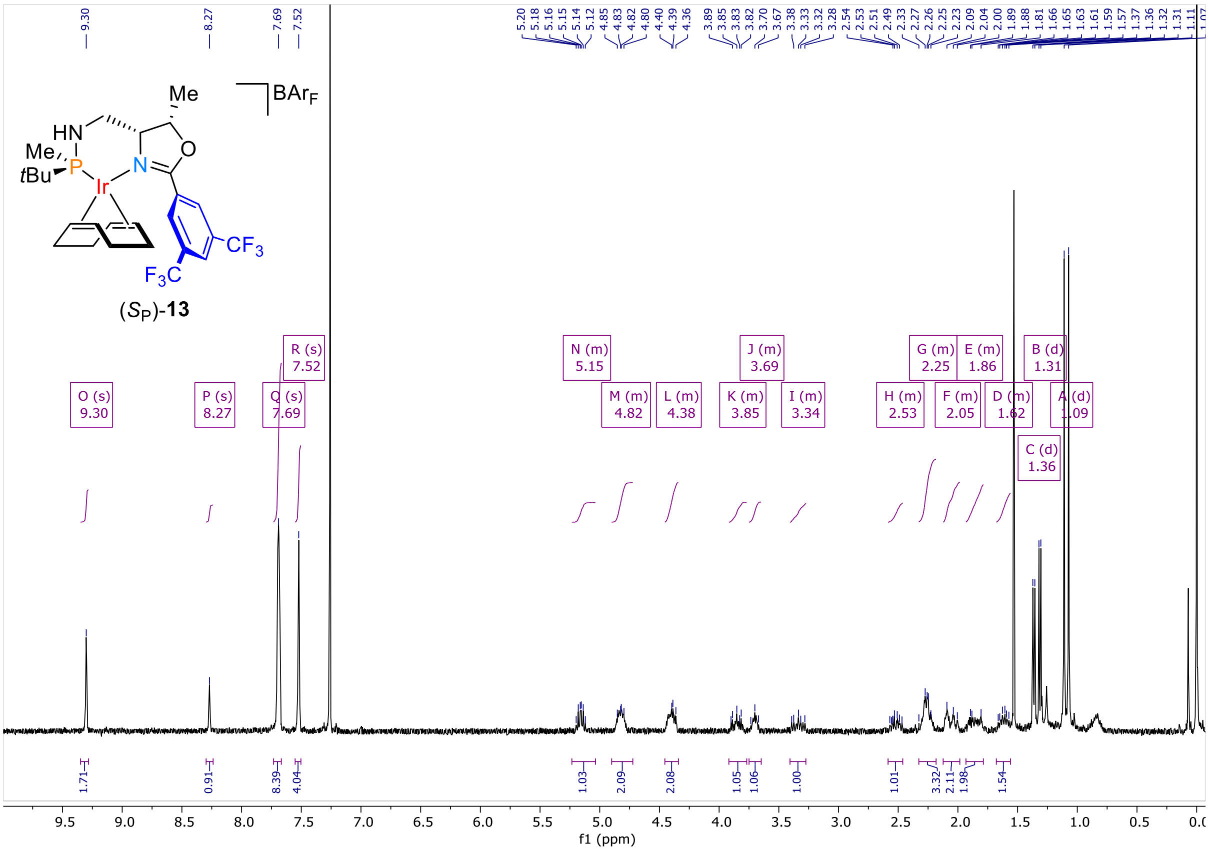

^{13}C-NMR (101 MHz, $CDCl_3$):

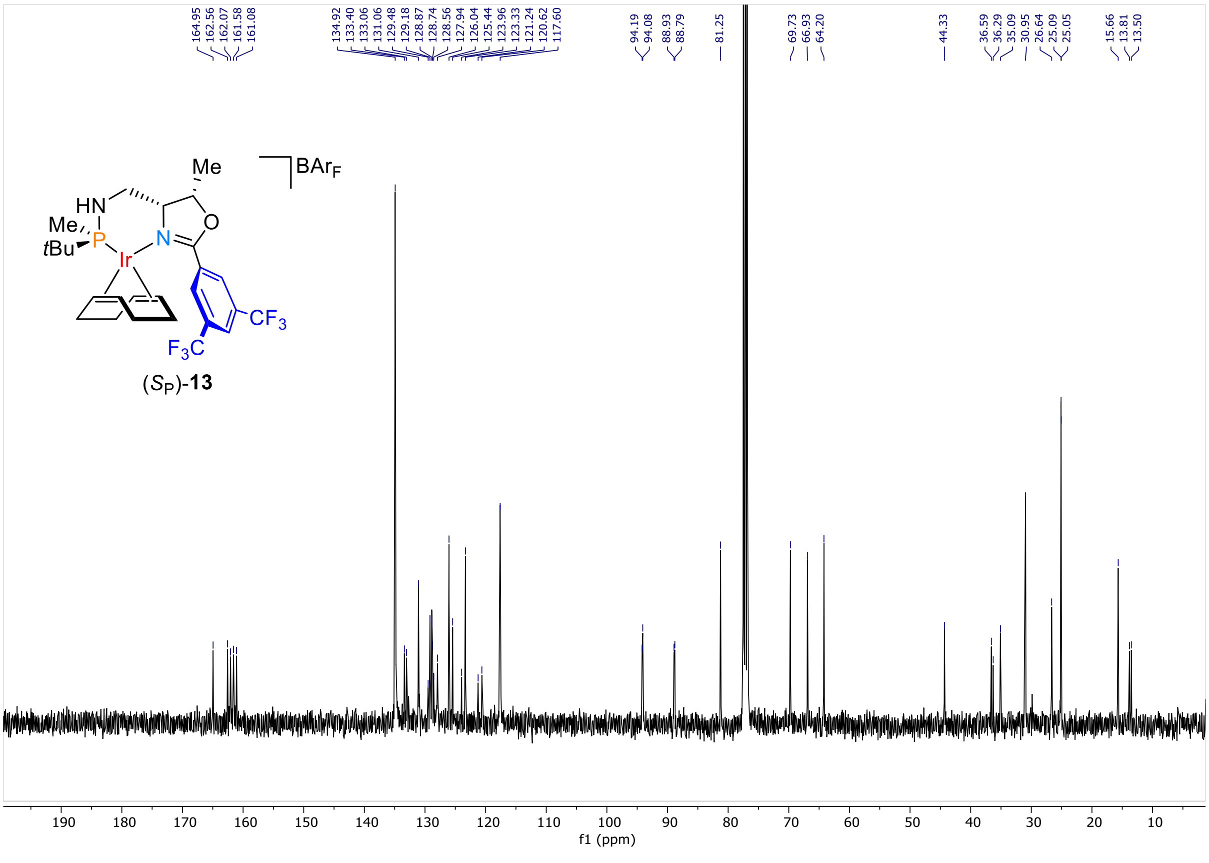

^{31}P-NMR (162 MHz, $CDCl_3$):

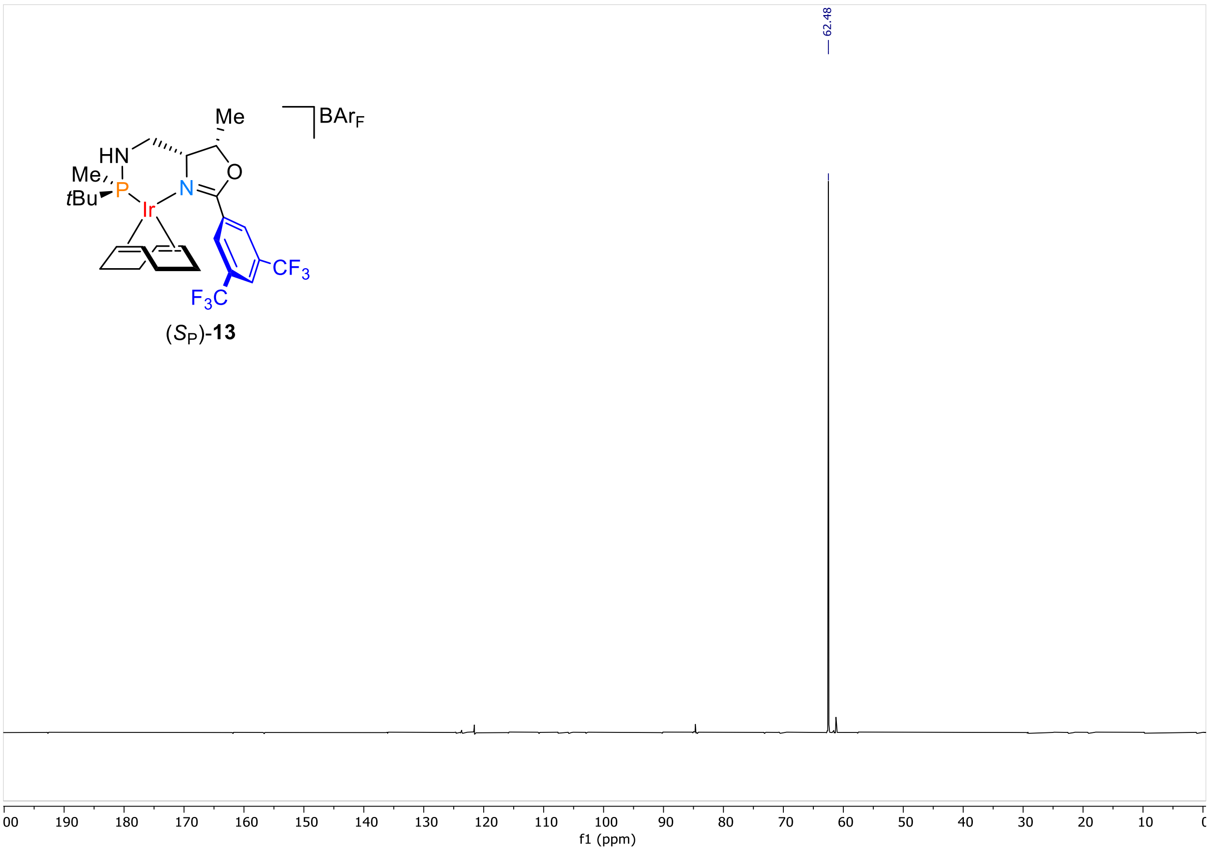

^{1}H-NMR (400 MHz, $CDCl_3$):

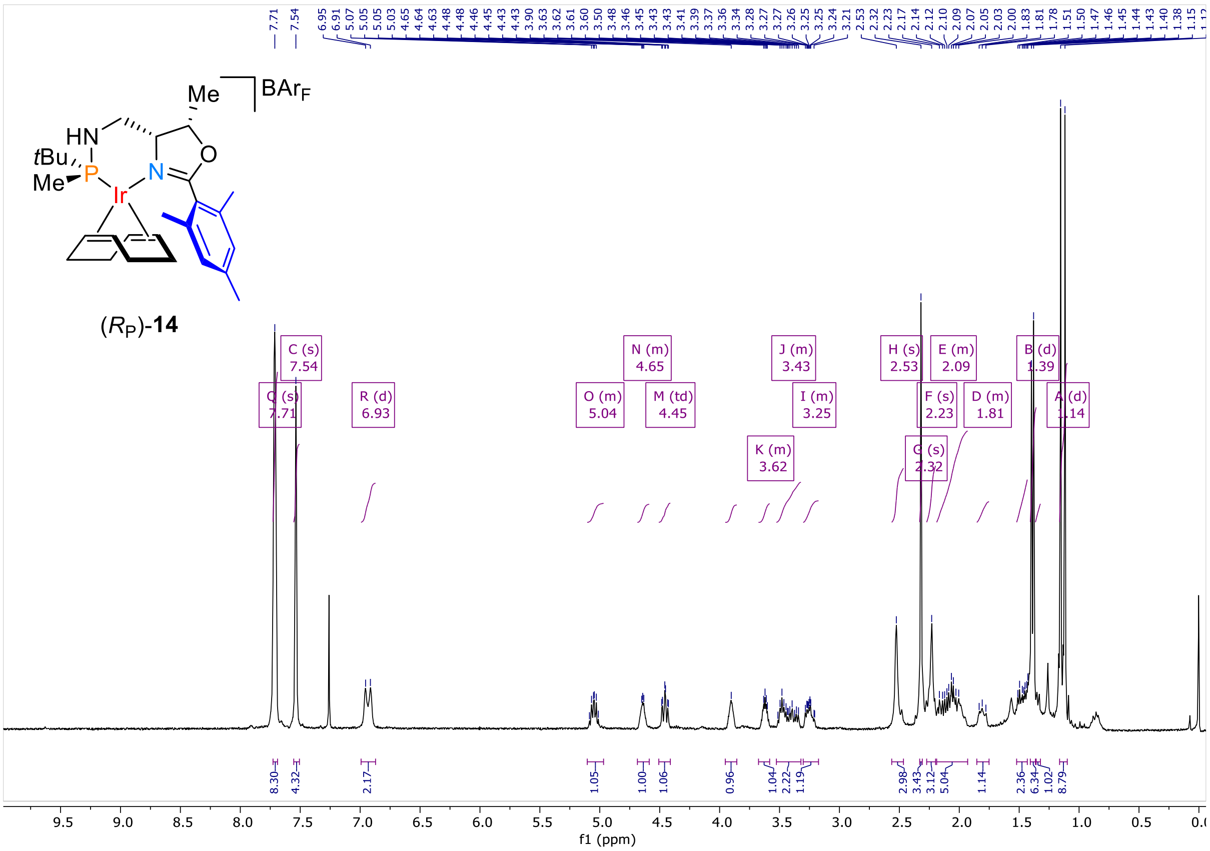

^{13}C-NMR (101 MHz, $CDCl_3$):

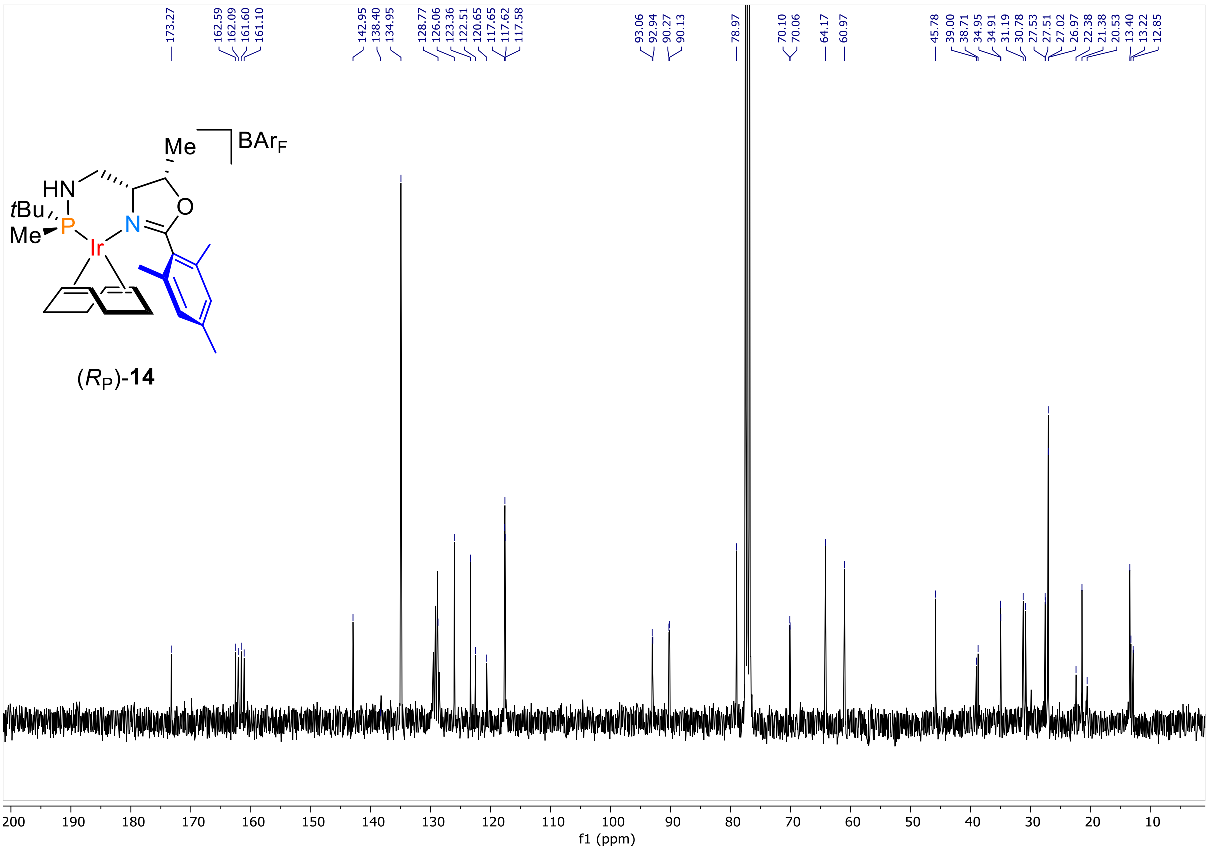

^{31}P-NMR (162 MHz, $CDCl_3$):

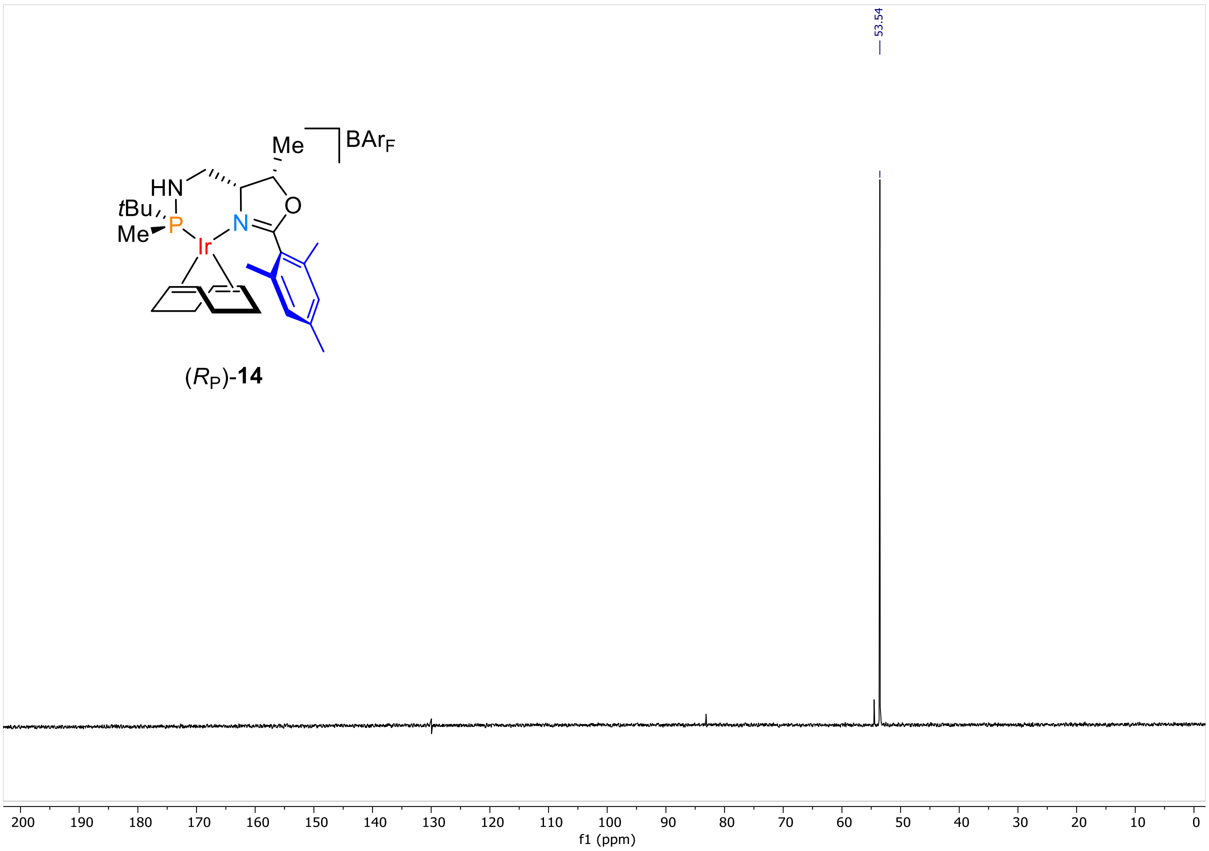

^{1}H-NMR (400 MHz, $CDCl_3$):

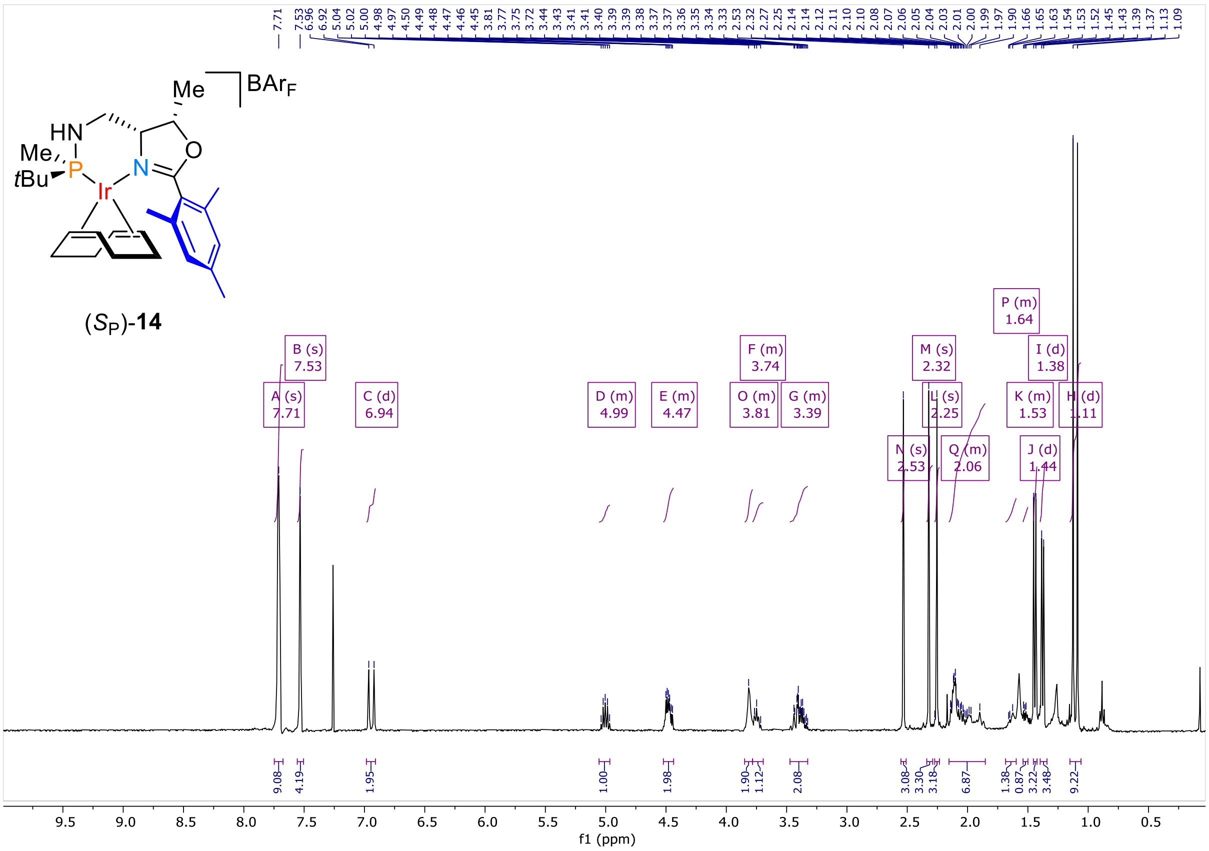

^{13}C-NMR (101 MHz, $CDCl_3$):

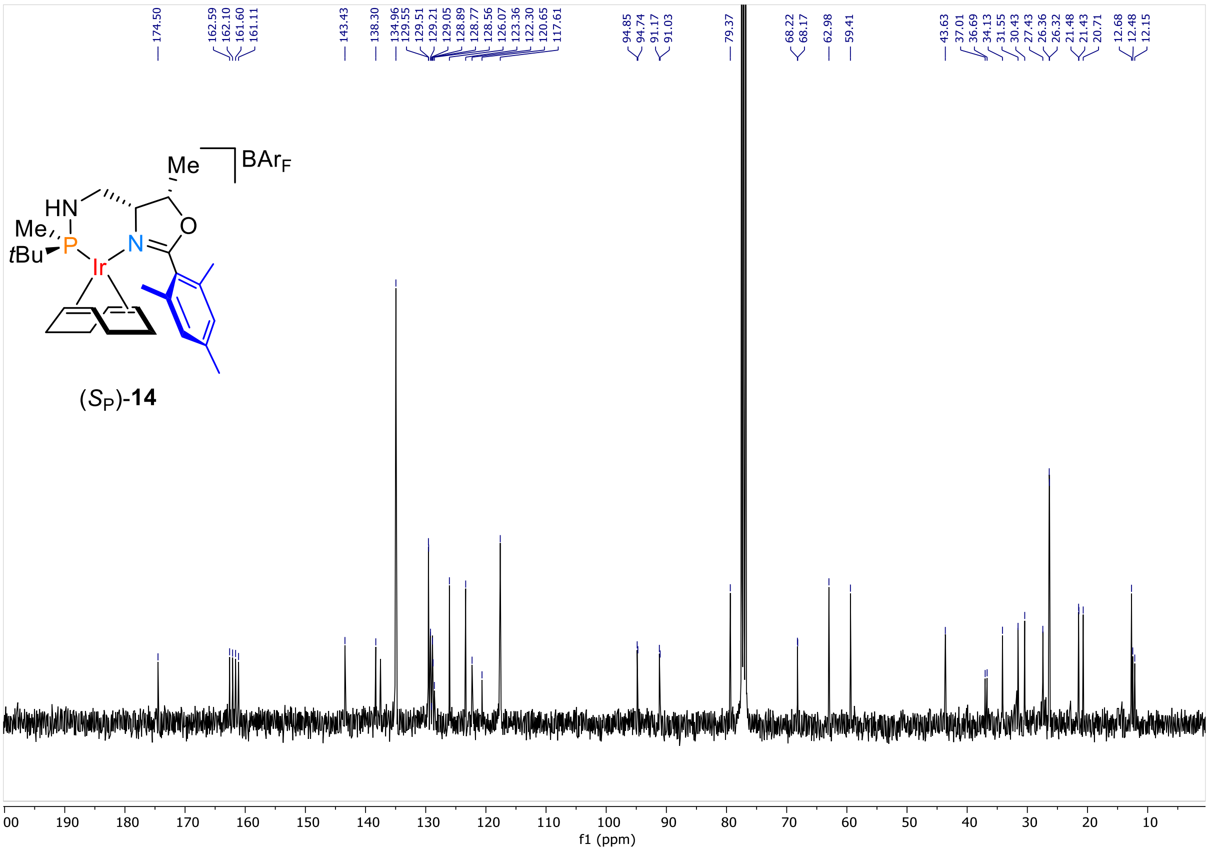

^{31}P-NMR (162 MHz, $CDCl_3$):

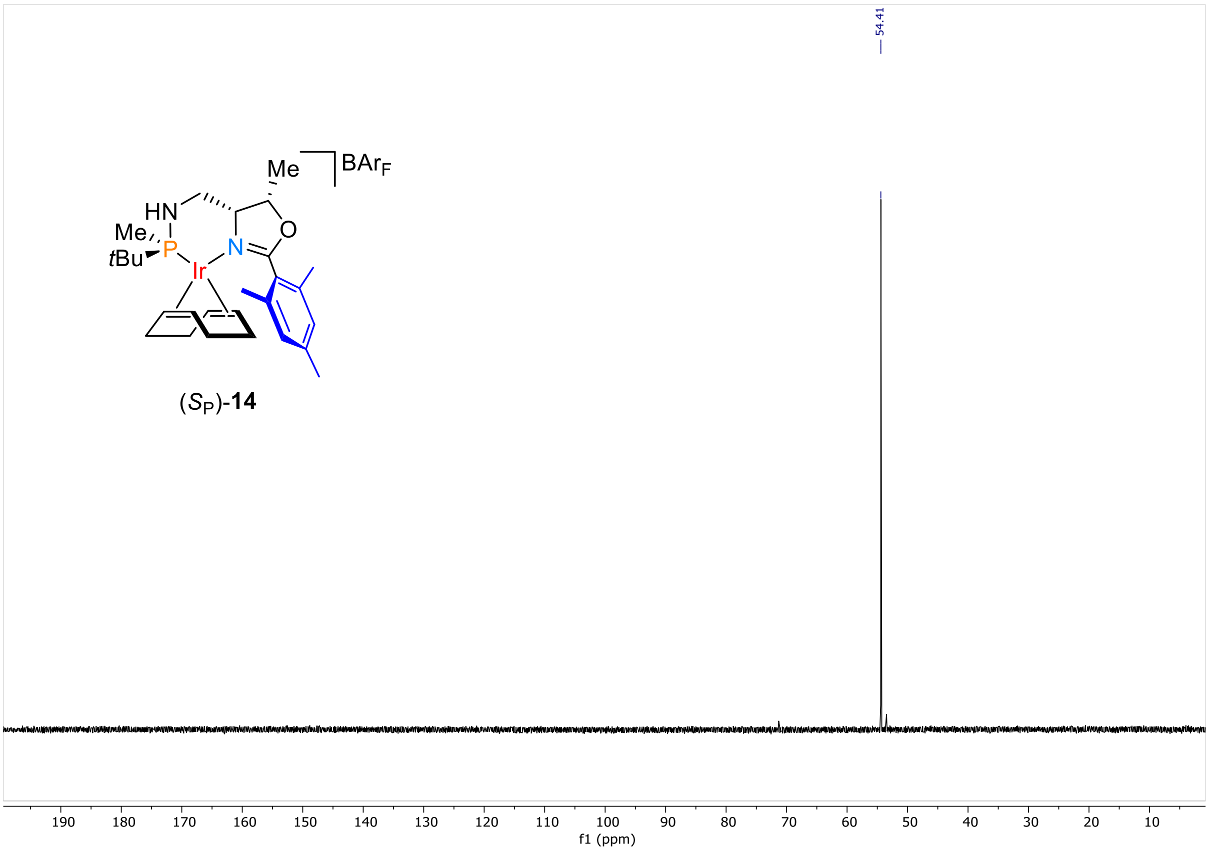

^{1}H-NMR (400 MHz, $CDCl_3$):

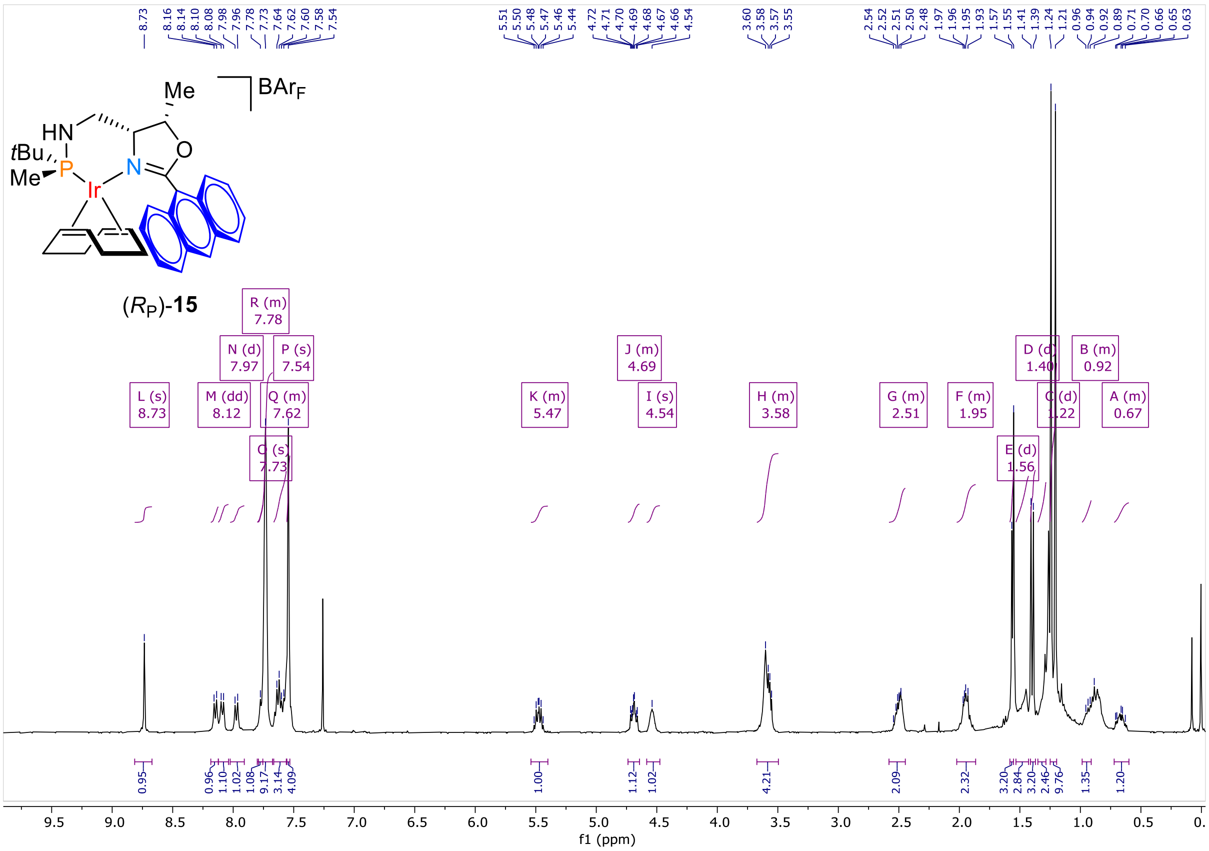

^{13}C-NMR (101 MHz, $CDCl_3$):

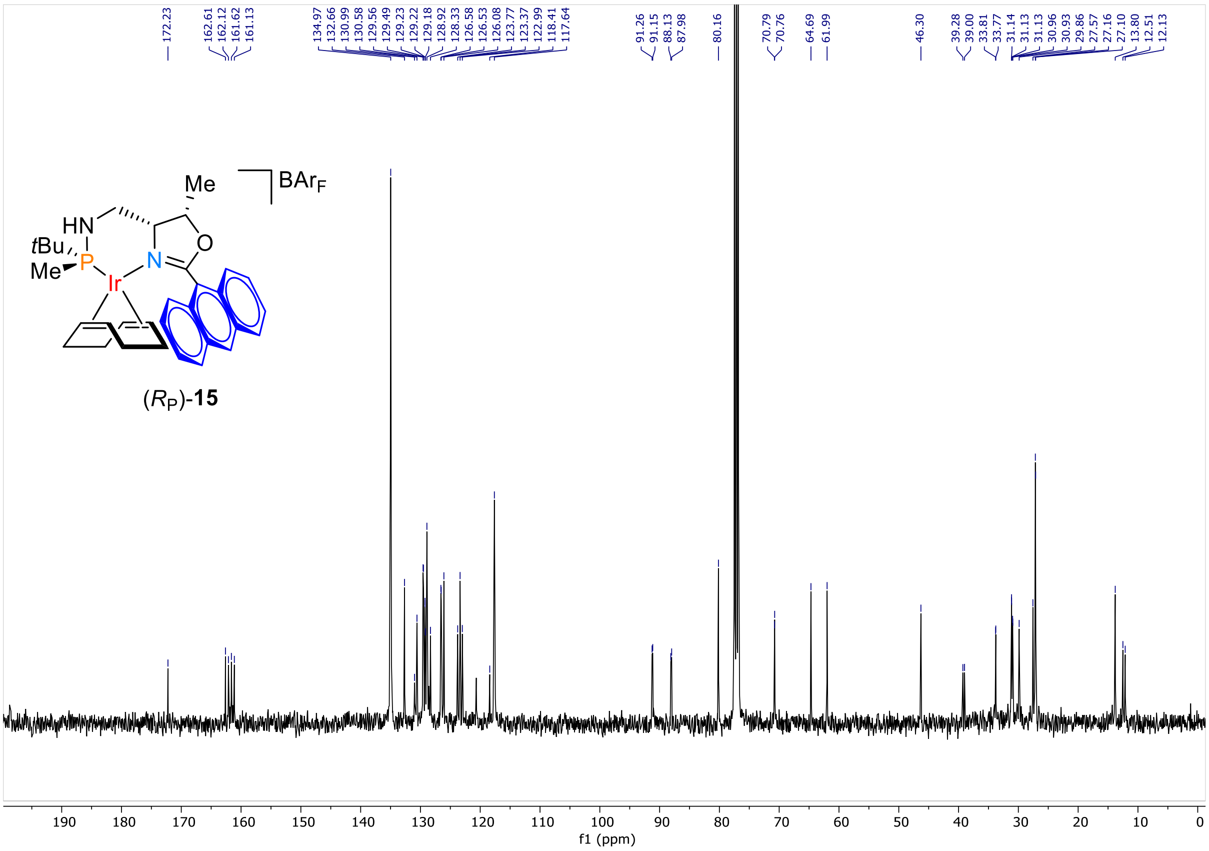

^{31}P-NMR (162 MHz, $CDCl_3$):

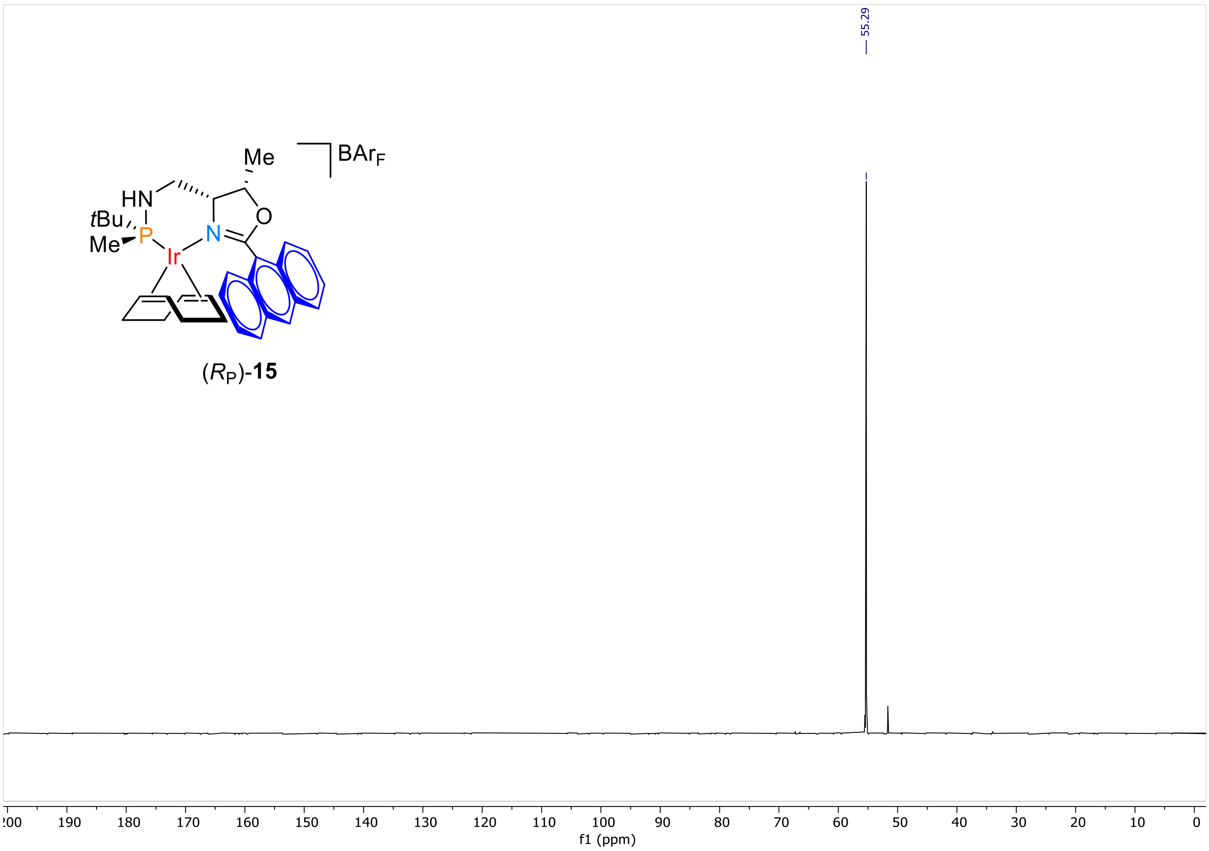

^{1}H-NMR (400 MHz, $CDCl_3$):

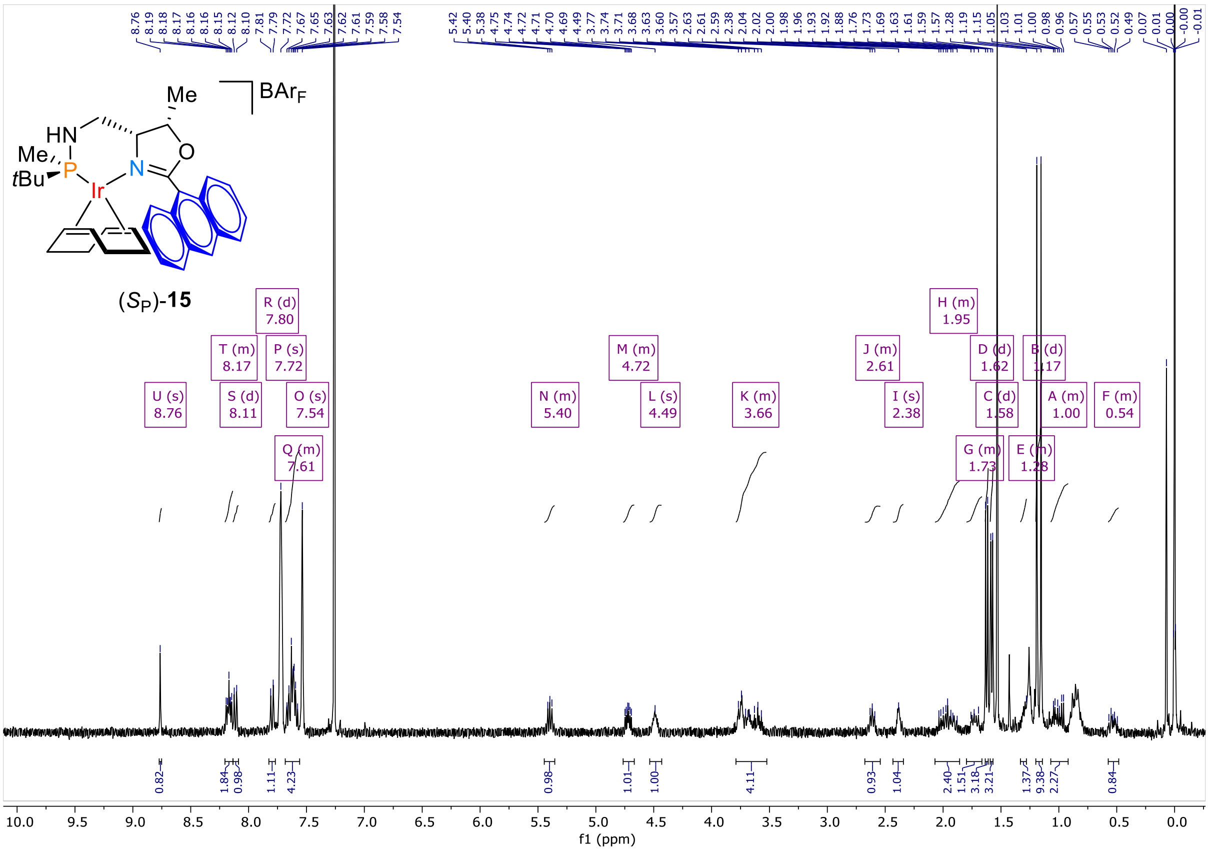

^{13}C-NMR (101 MHz, $CDCl_3$):

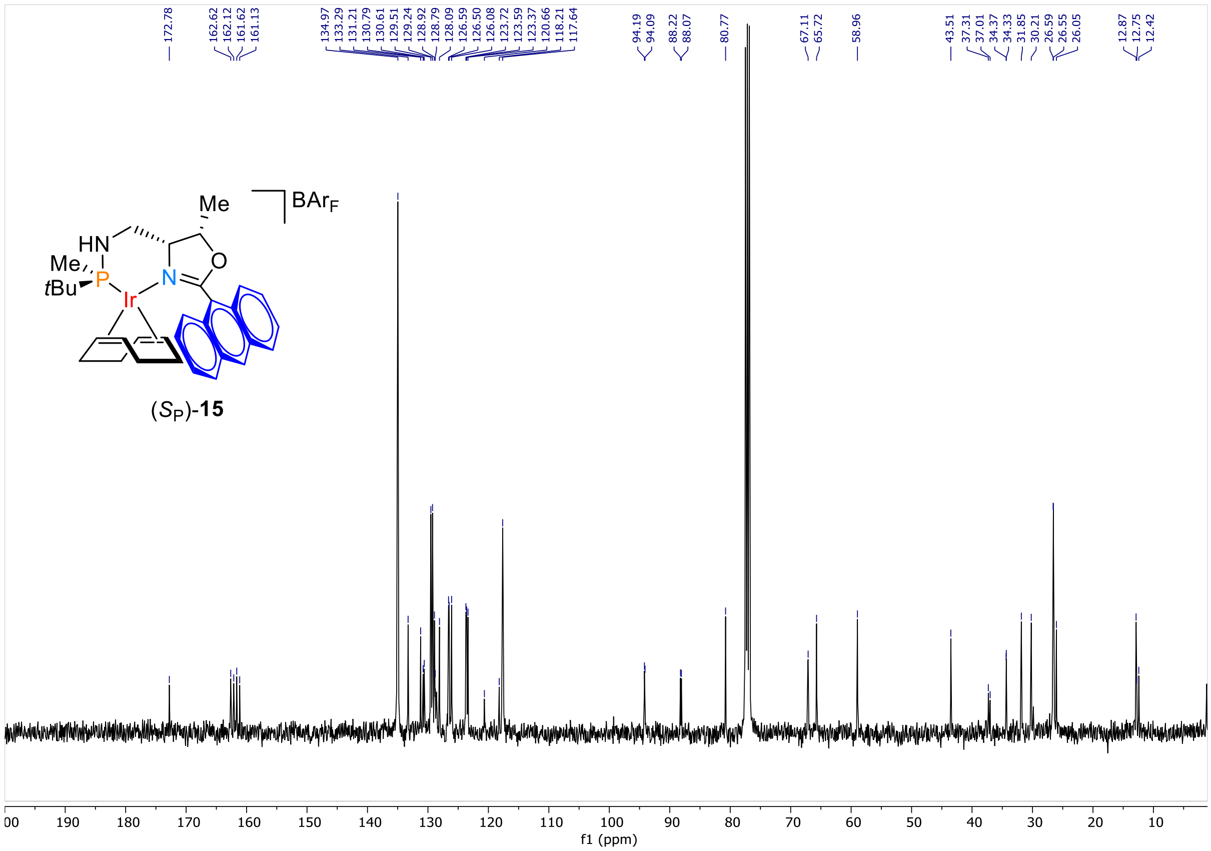

^{31}P-NMR (162 MHz, $CDCl_3$):

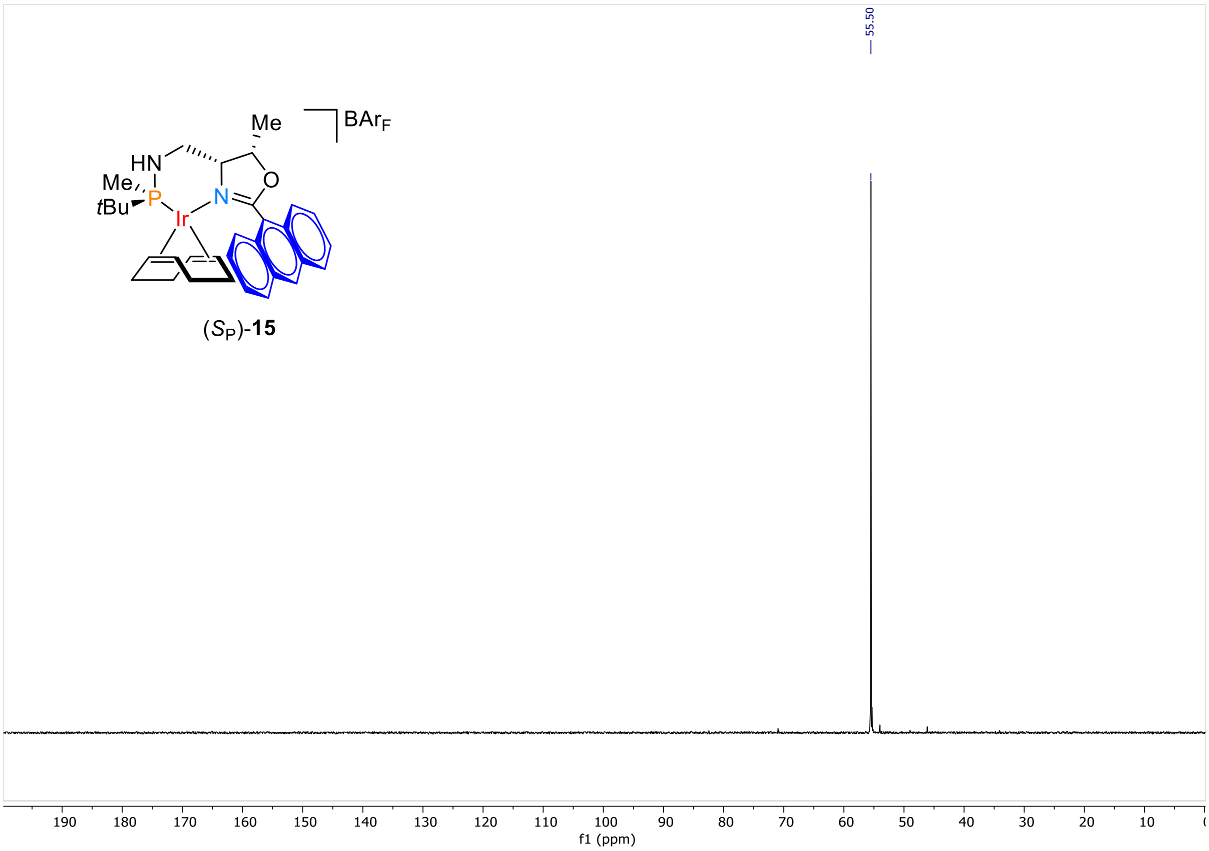

^{1}H-NMR (400 MHz, $CDCl_3$):

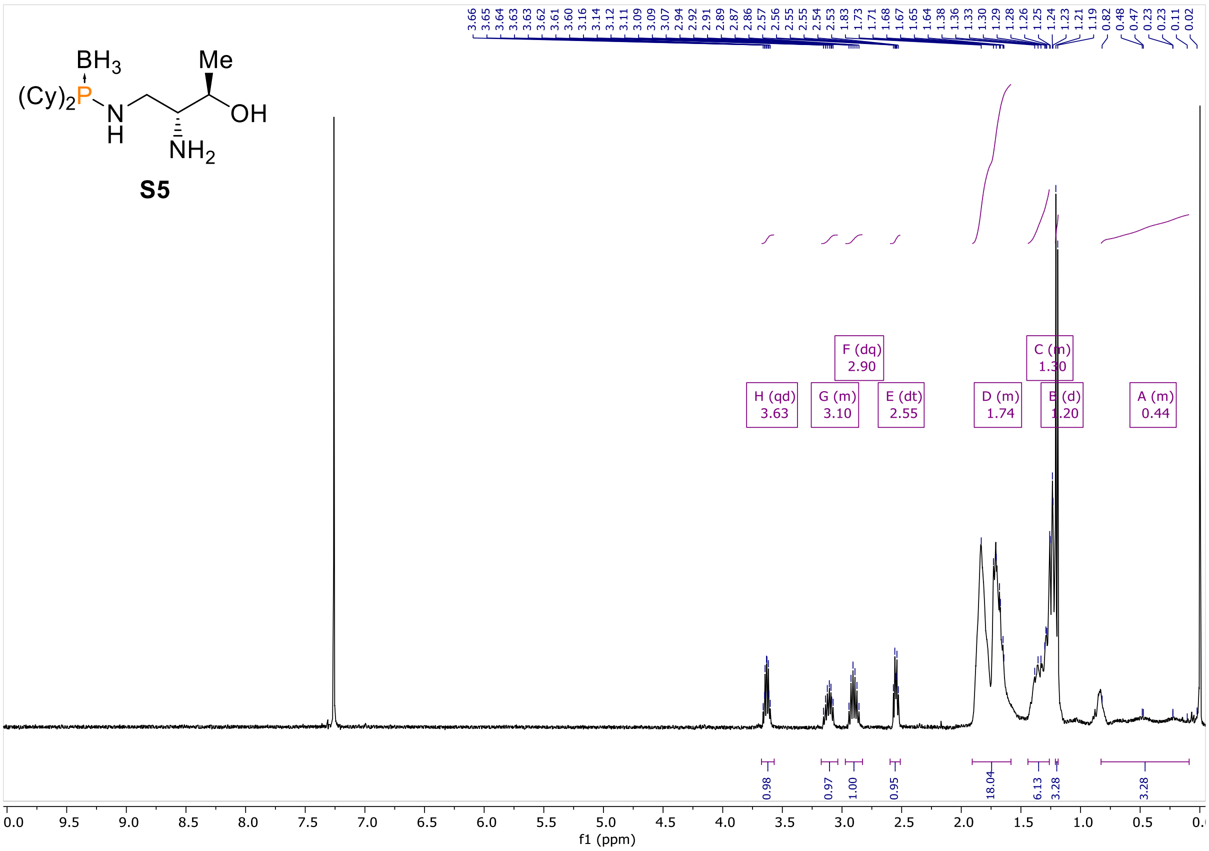

^{13}C-NMR (101 MHz, $CDCl_3$):

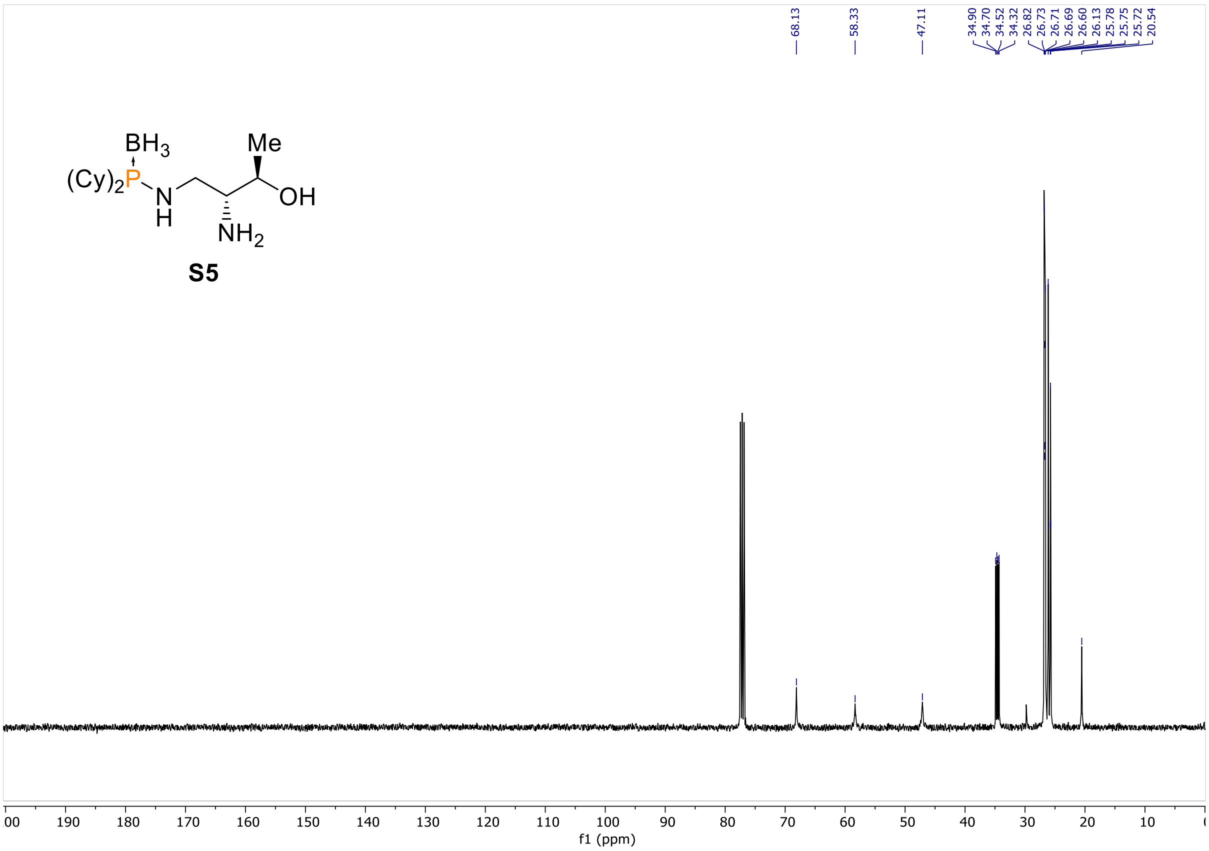

^{31}P-NMR (162 MHz, $CDCl_3$):

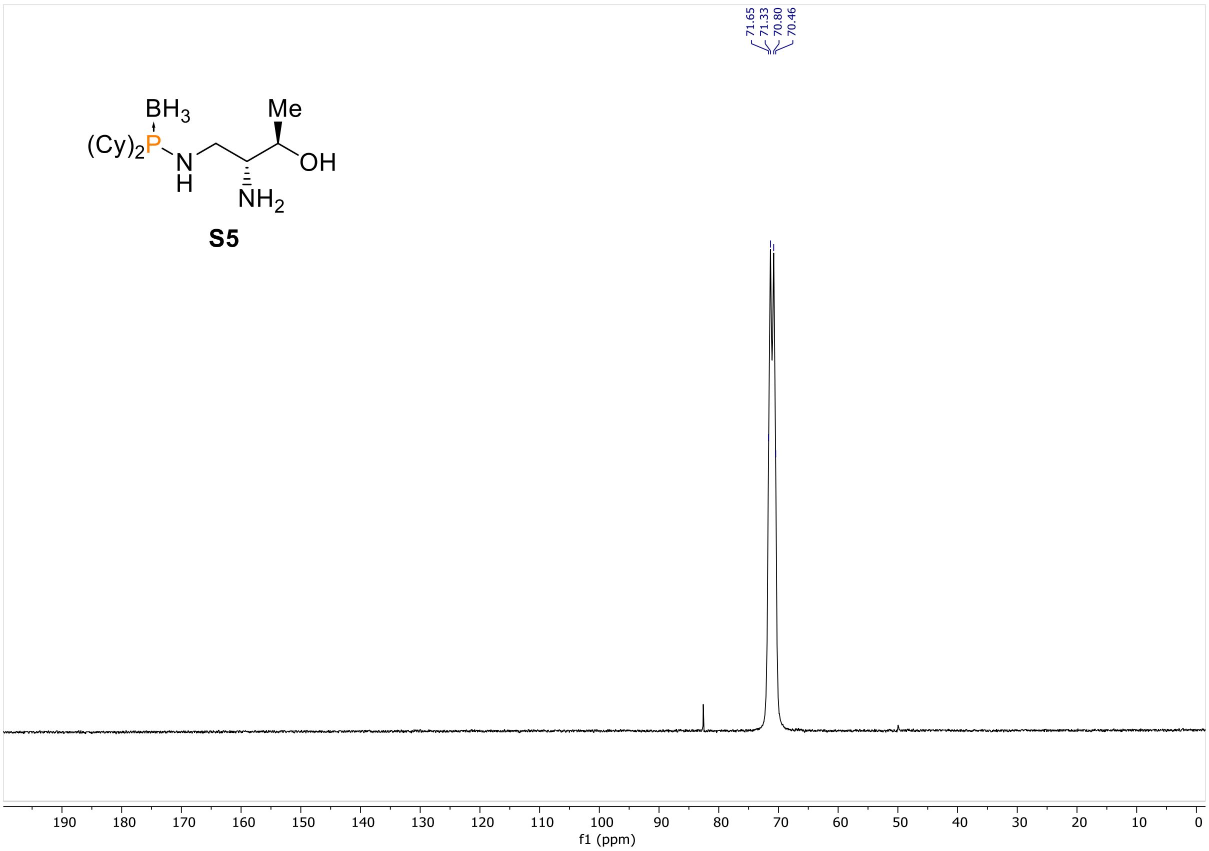

^{1}H-NMR (400 MHz, $CDCl_3$):

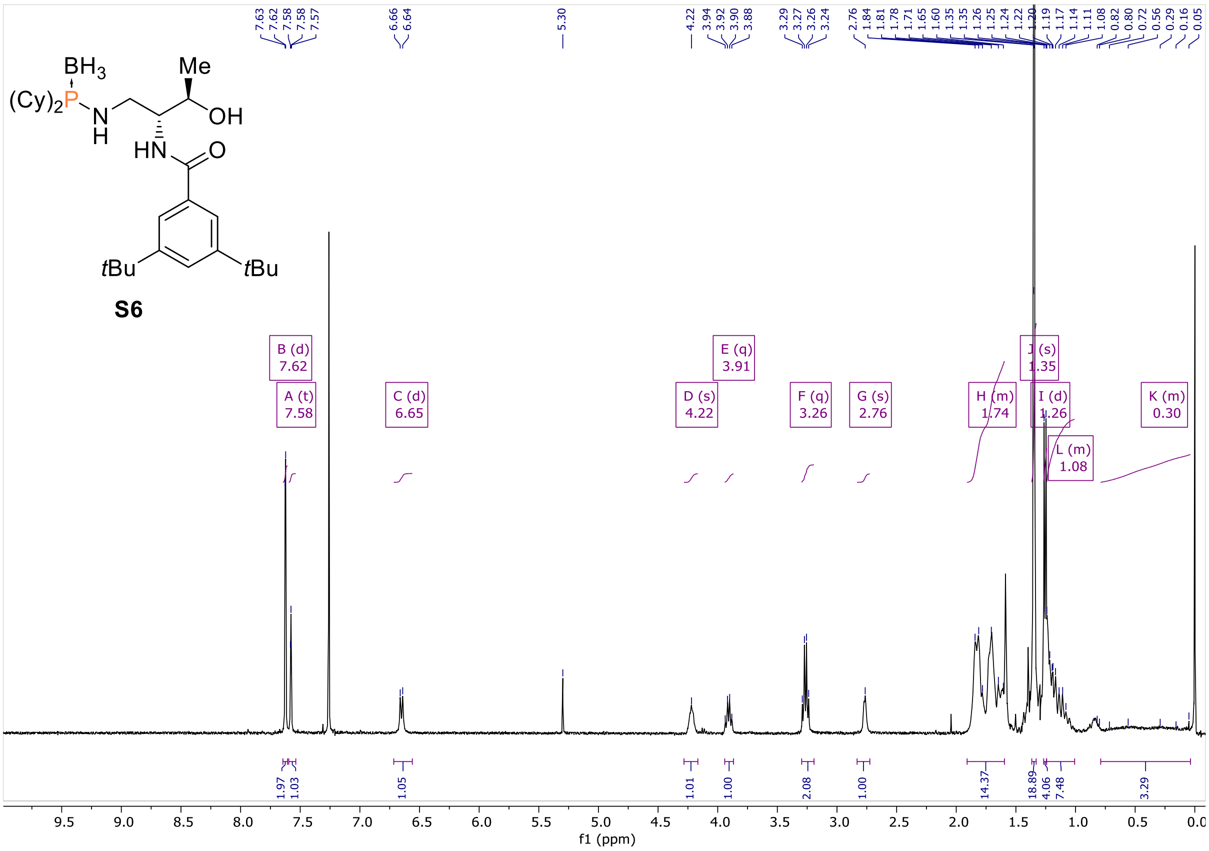

^{1}H-NMR (400 MHz, $CDCl_3$):

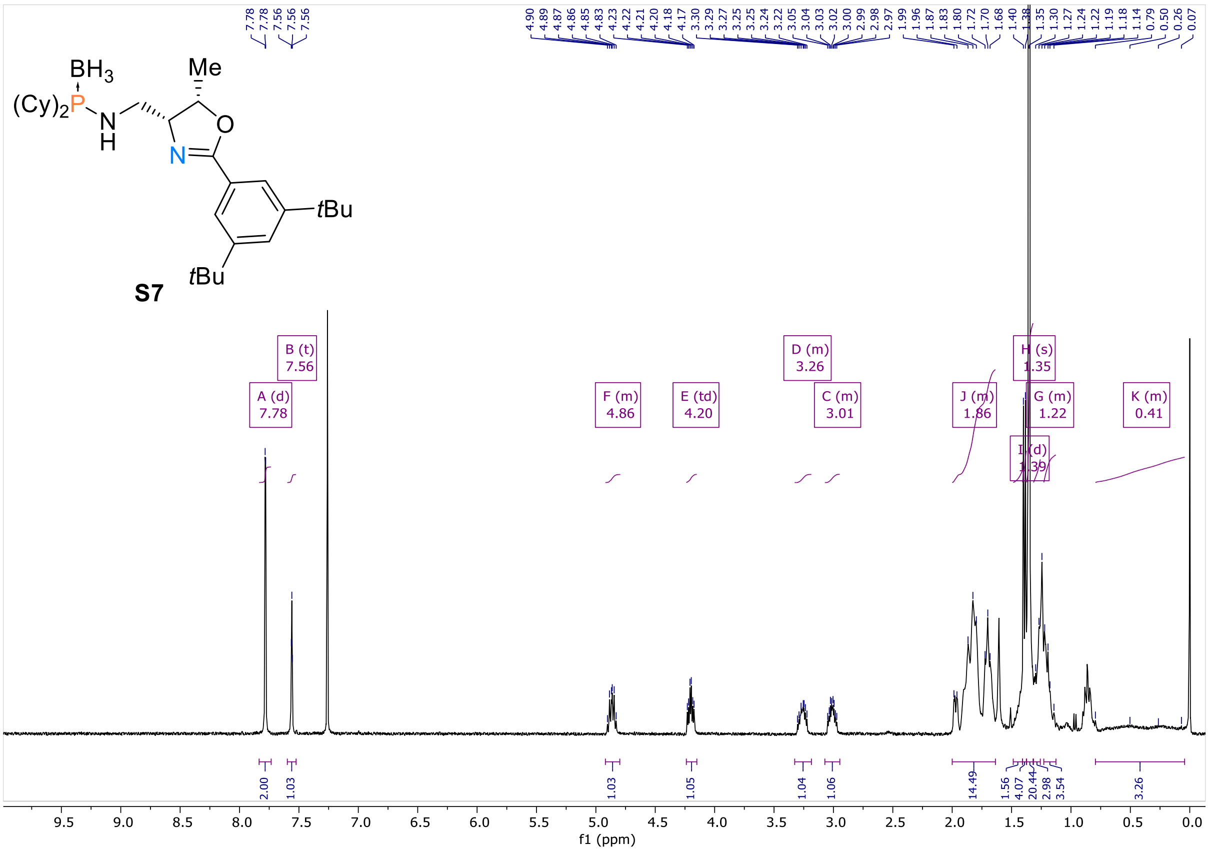

^{13}C-NMR (101 MHz, $CDCl_3$):

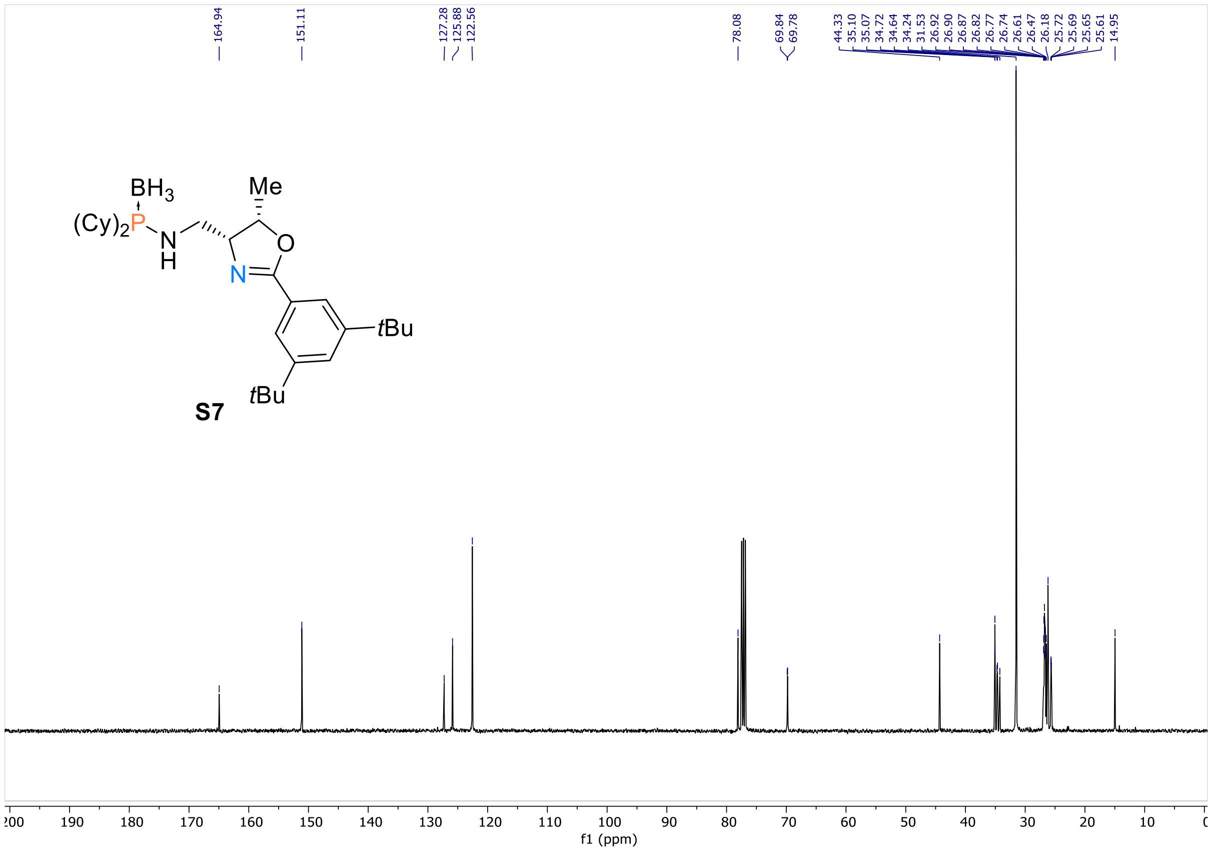

^{31}P-NMR (162 MHz, $CDCl_3$):

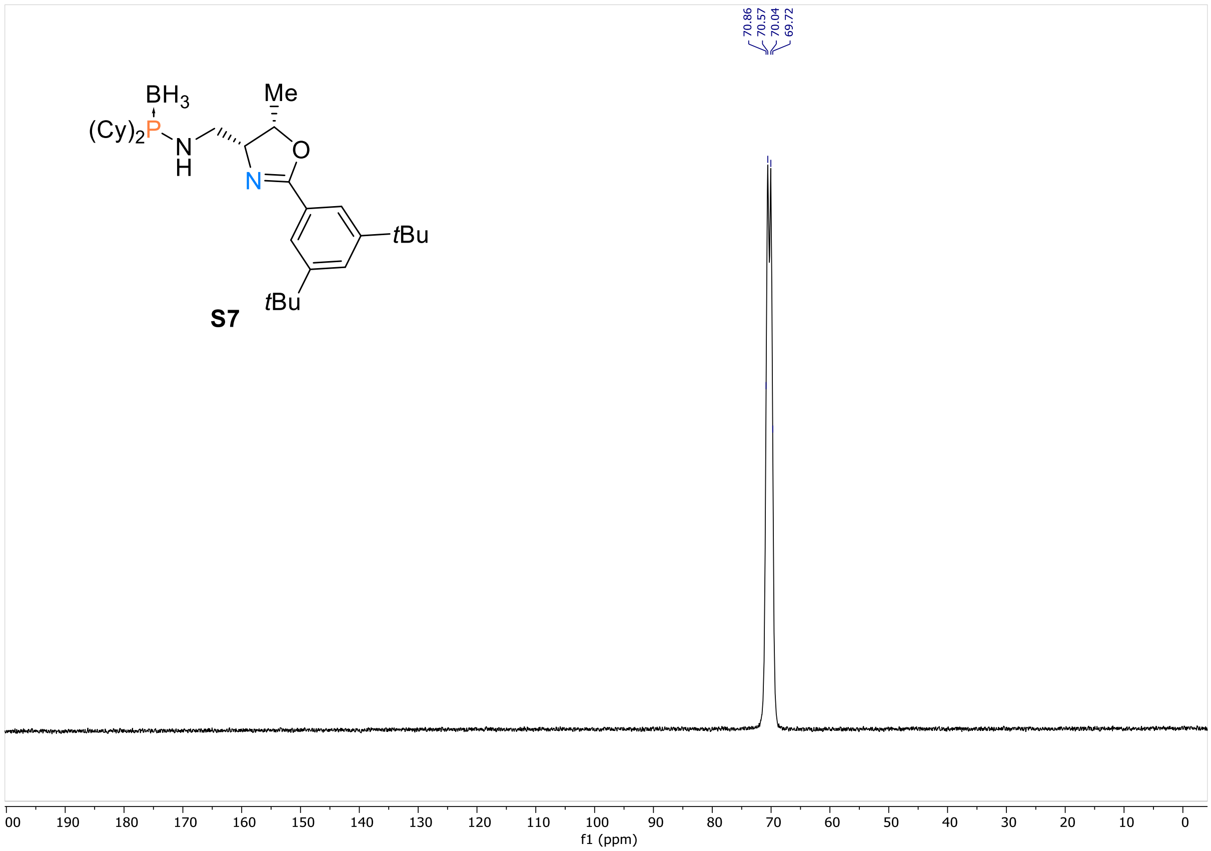

^{1}H-NMR (400 MHz, $CDCl_3$):

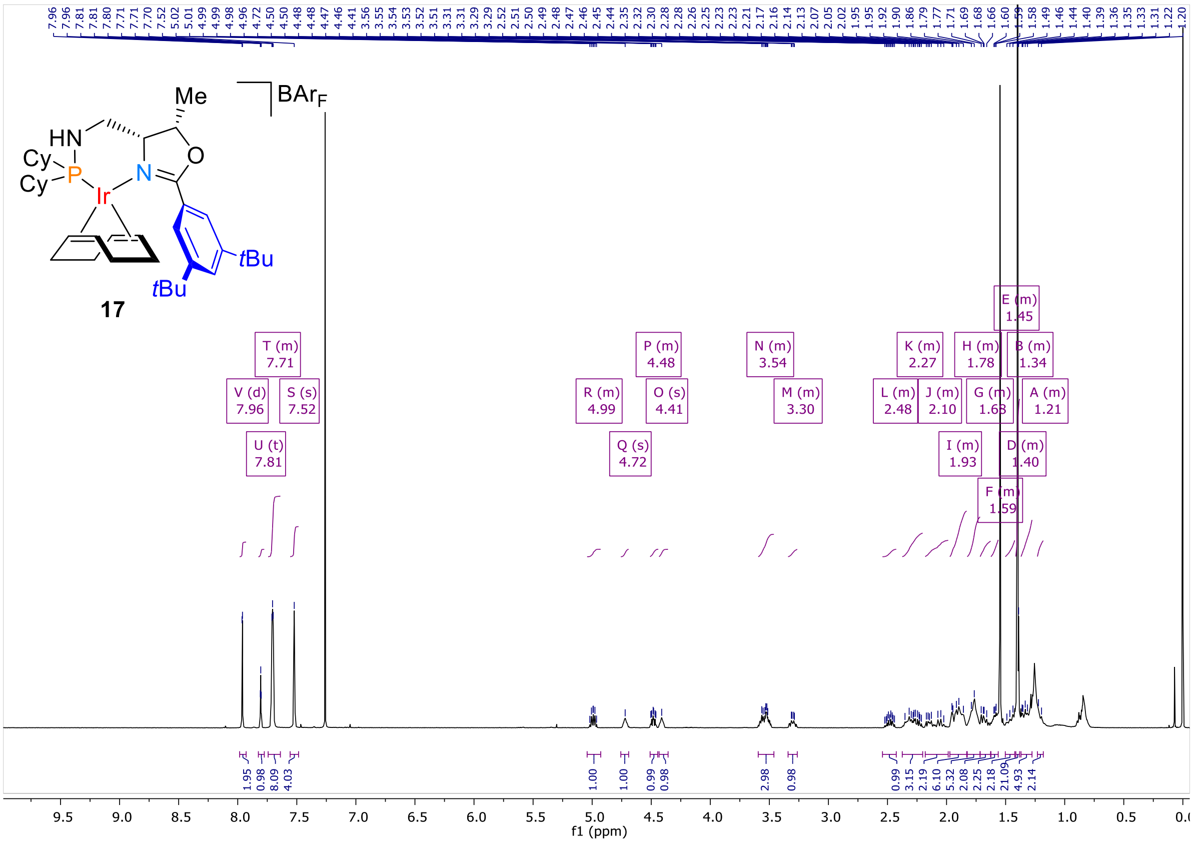

^{13}C-NMR (101 MHz, $CDCl_3$):

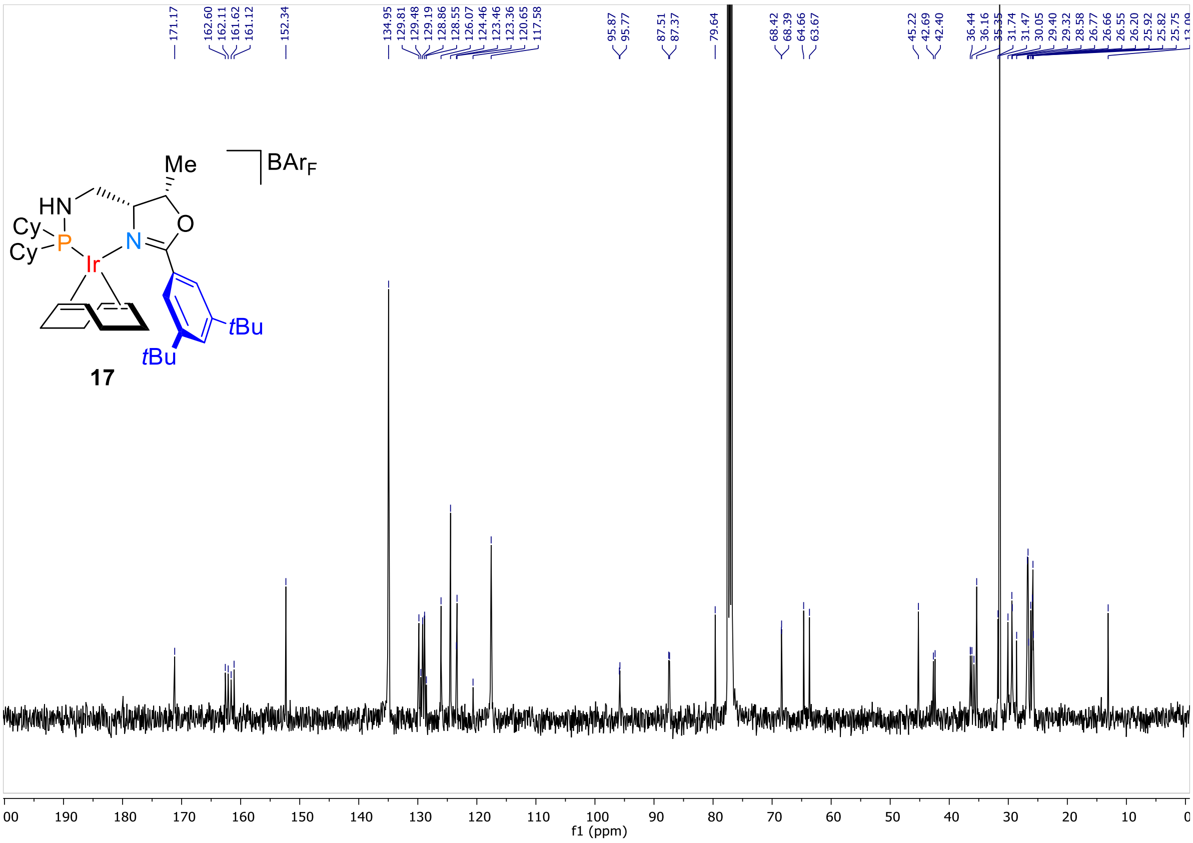

^{31}P-NMR (162 MHz, $CDCl_3$):

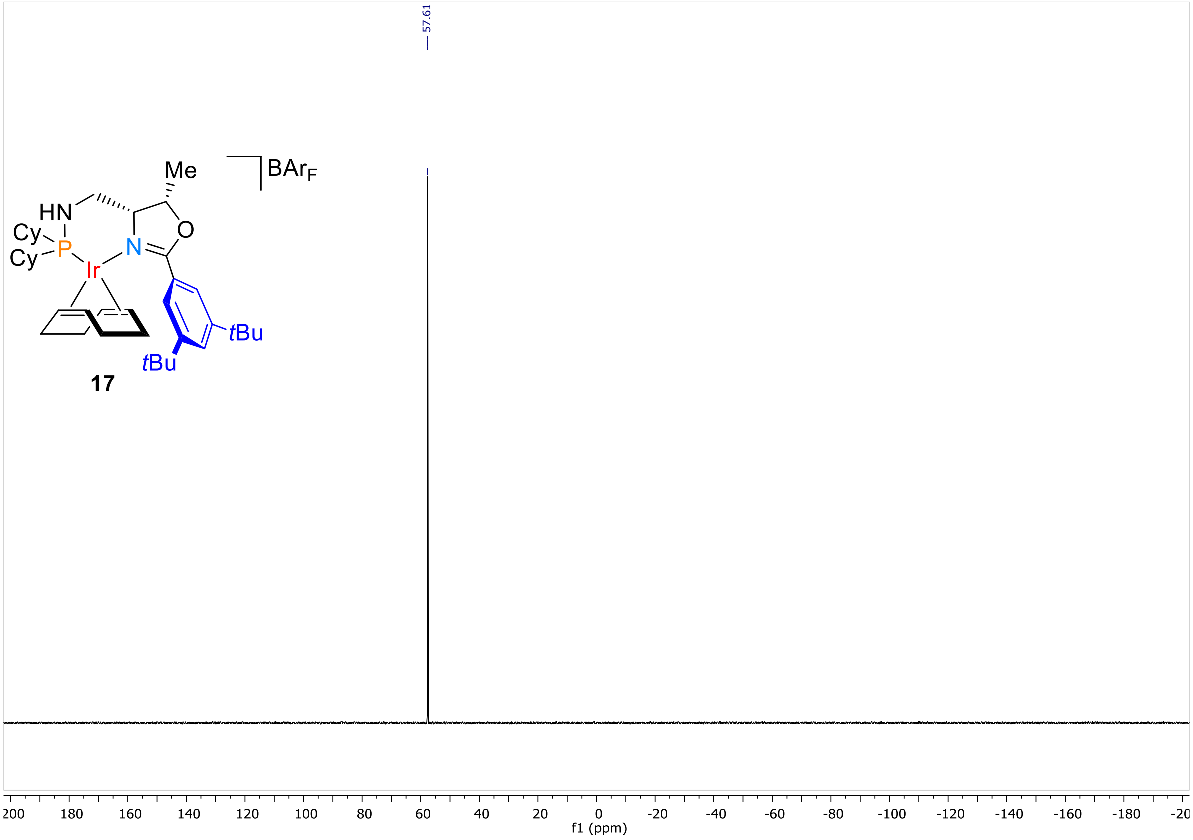

^{1}H-NMR (400 MHz, $CDCl_3$):

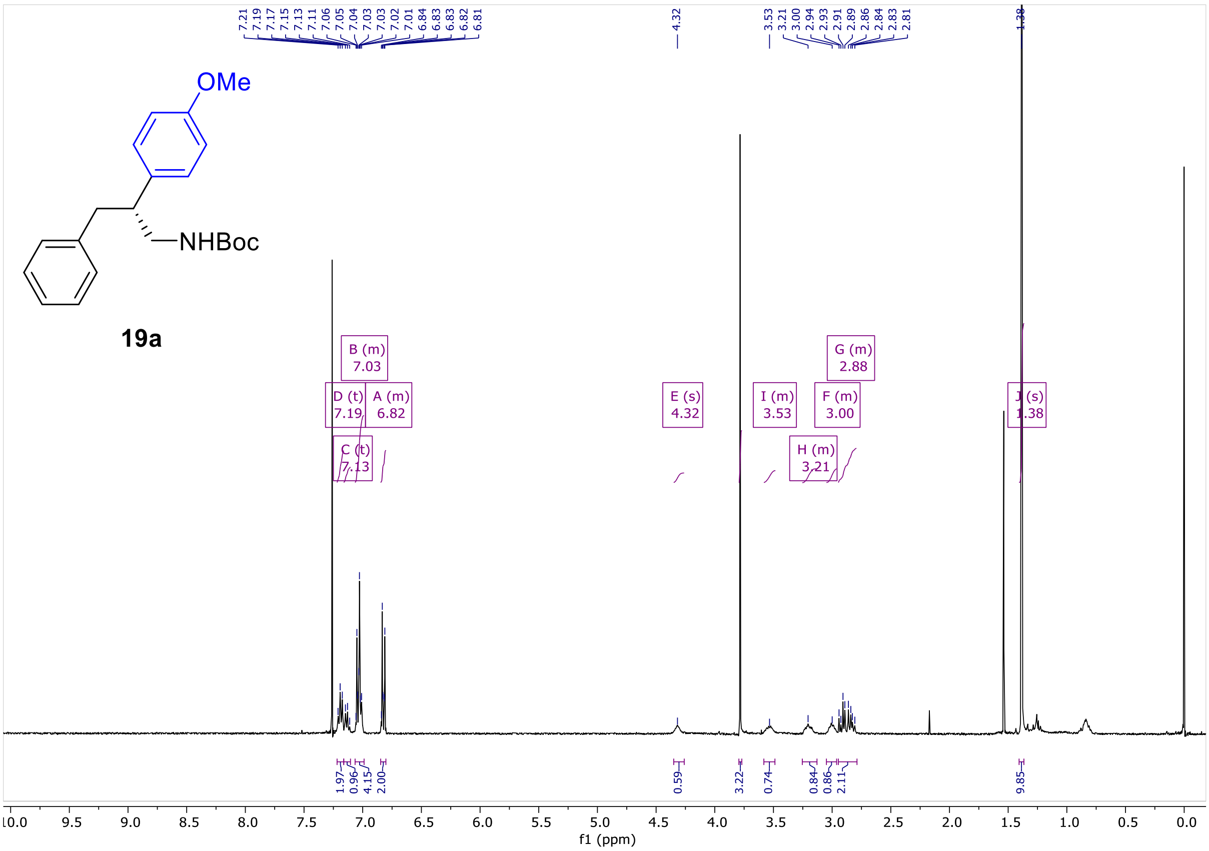

^{13}C-NMR (101 MHz, $CDCl_3$):

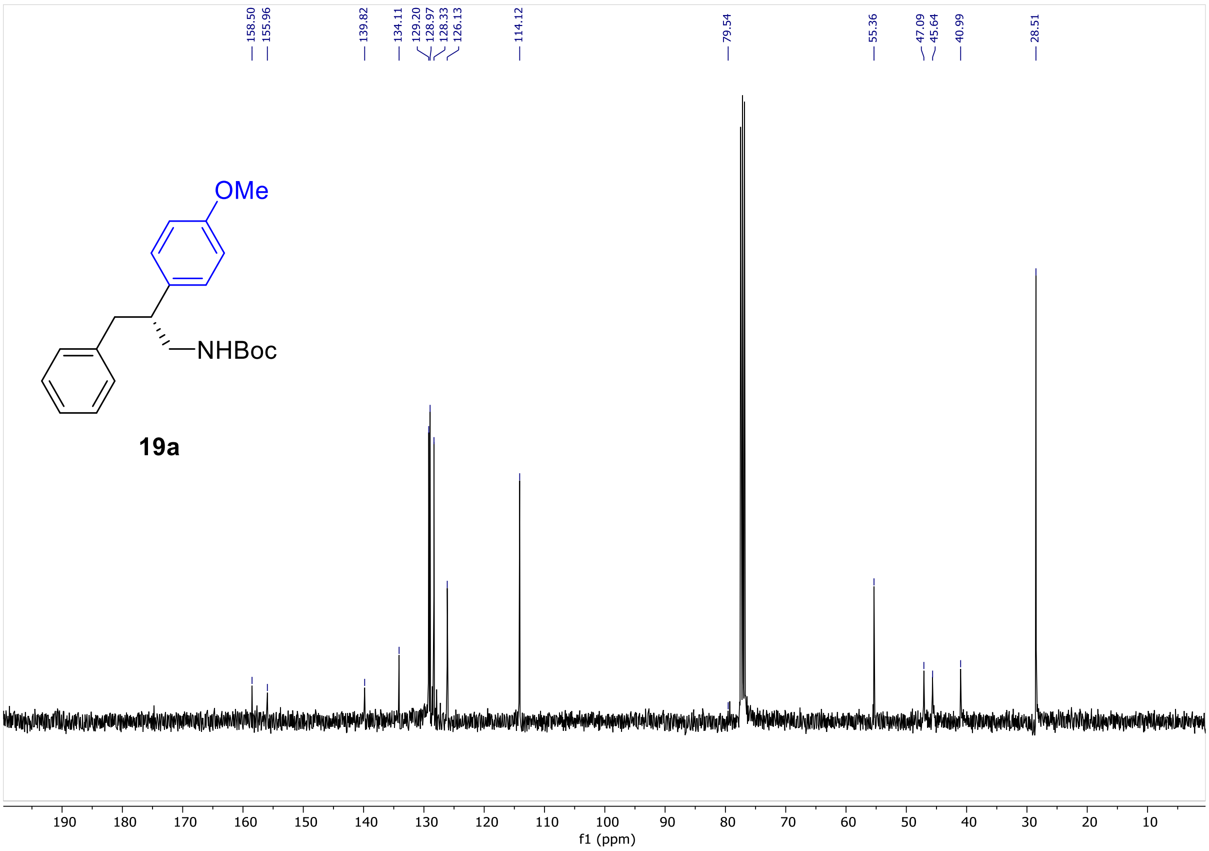

^{1}H-NMR (400 MHz, $CDCl_3$):

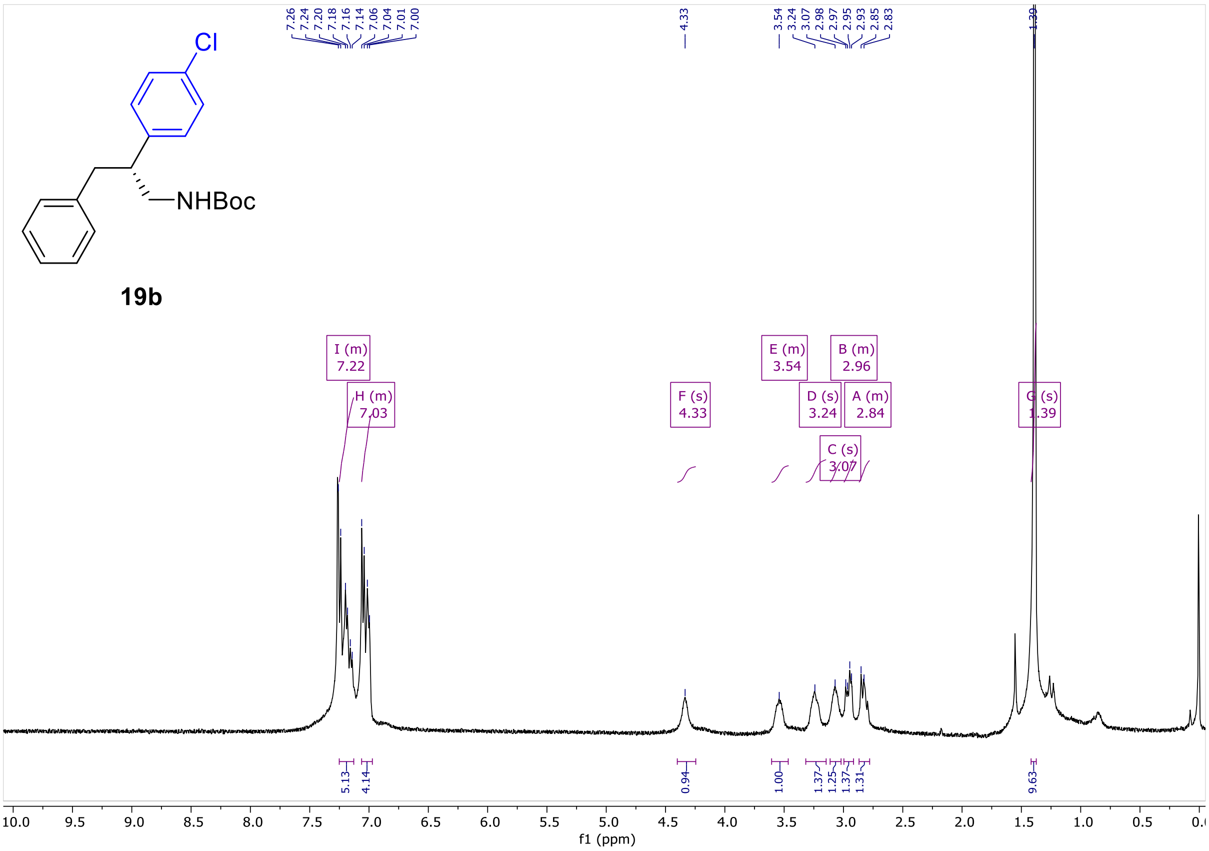

^{13}C-NMR (101 MHz, $CDCl_3$):

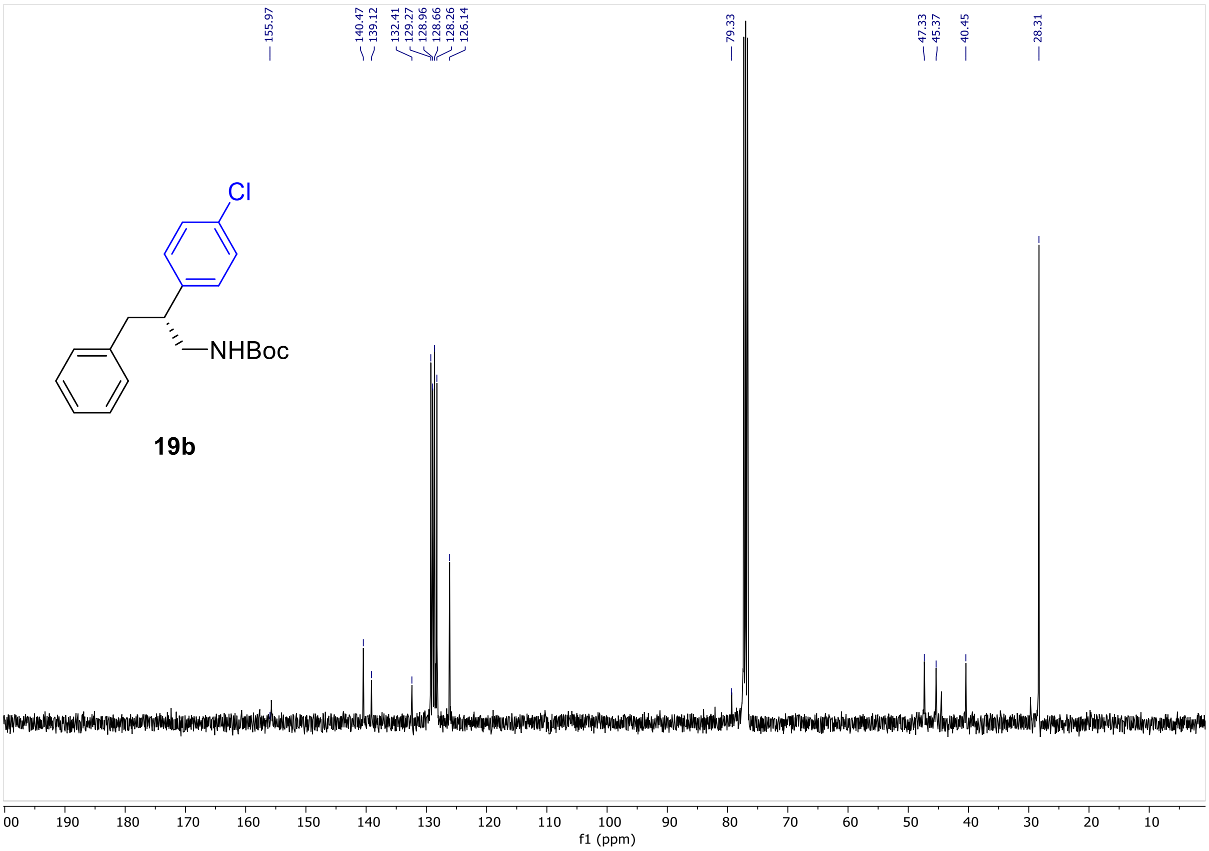

^{1}H-NMR (400 MHz, $CDCl_3$):

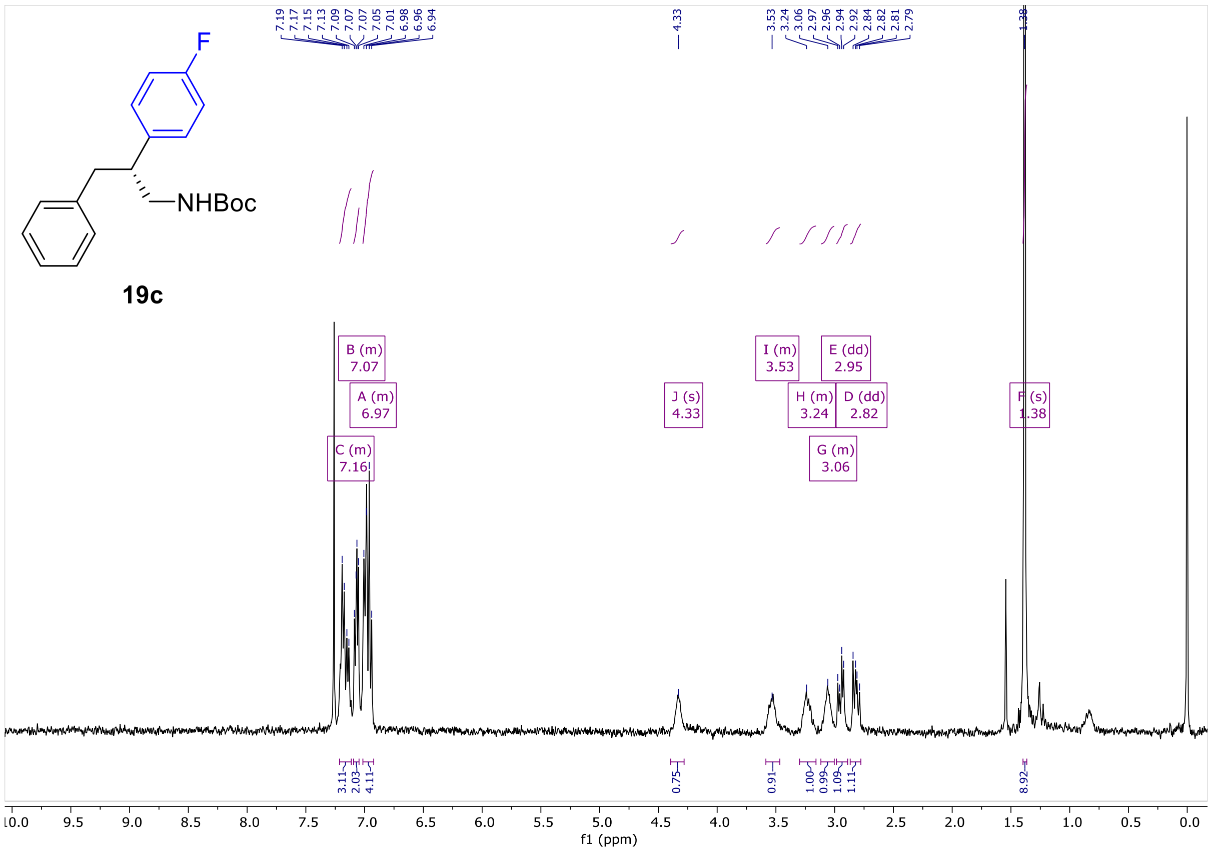

19c

^{13}C-NMR (101 MHz, $CDCl_3$):

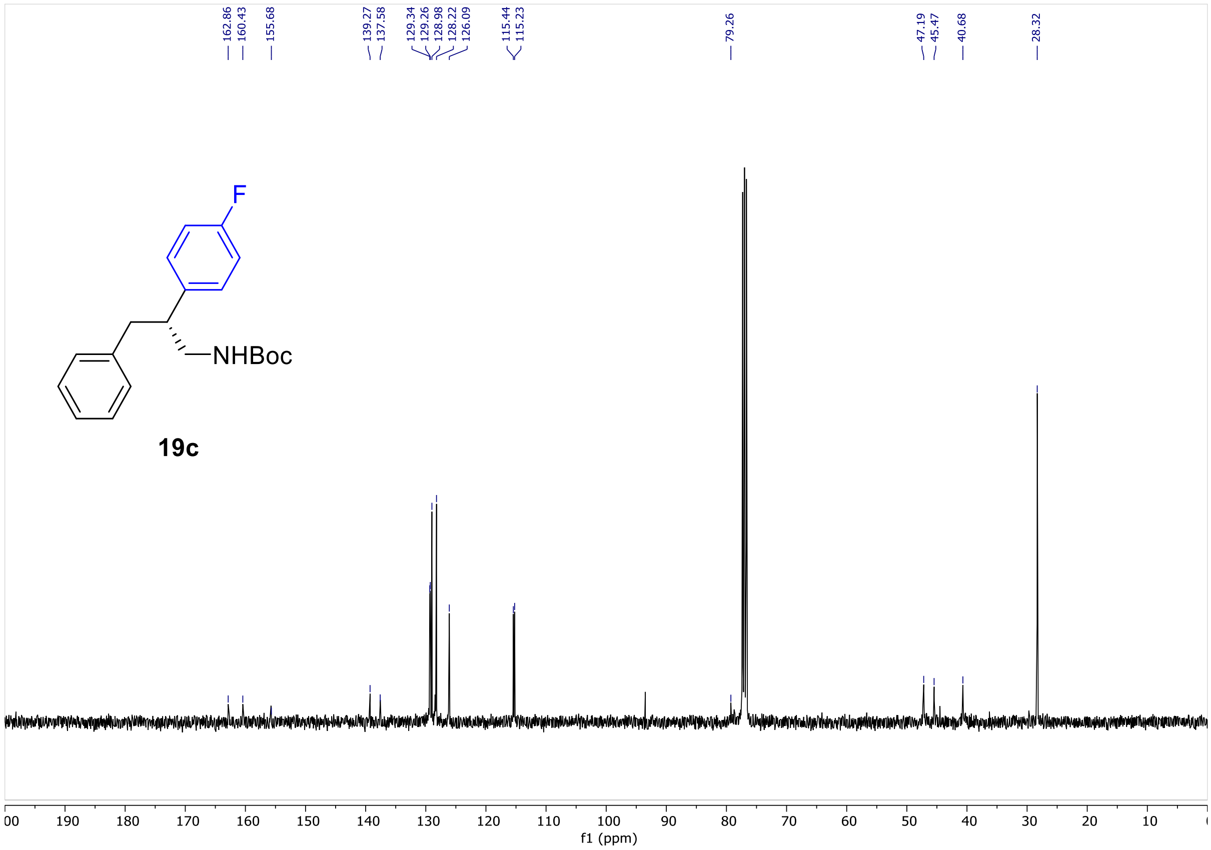

19c

^{1}H-NMR (400 MHz, $CDCl_3$):

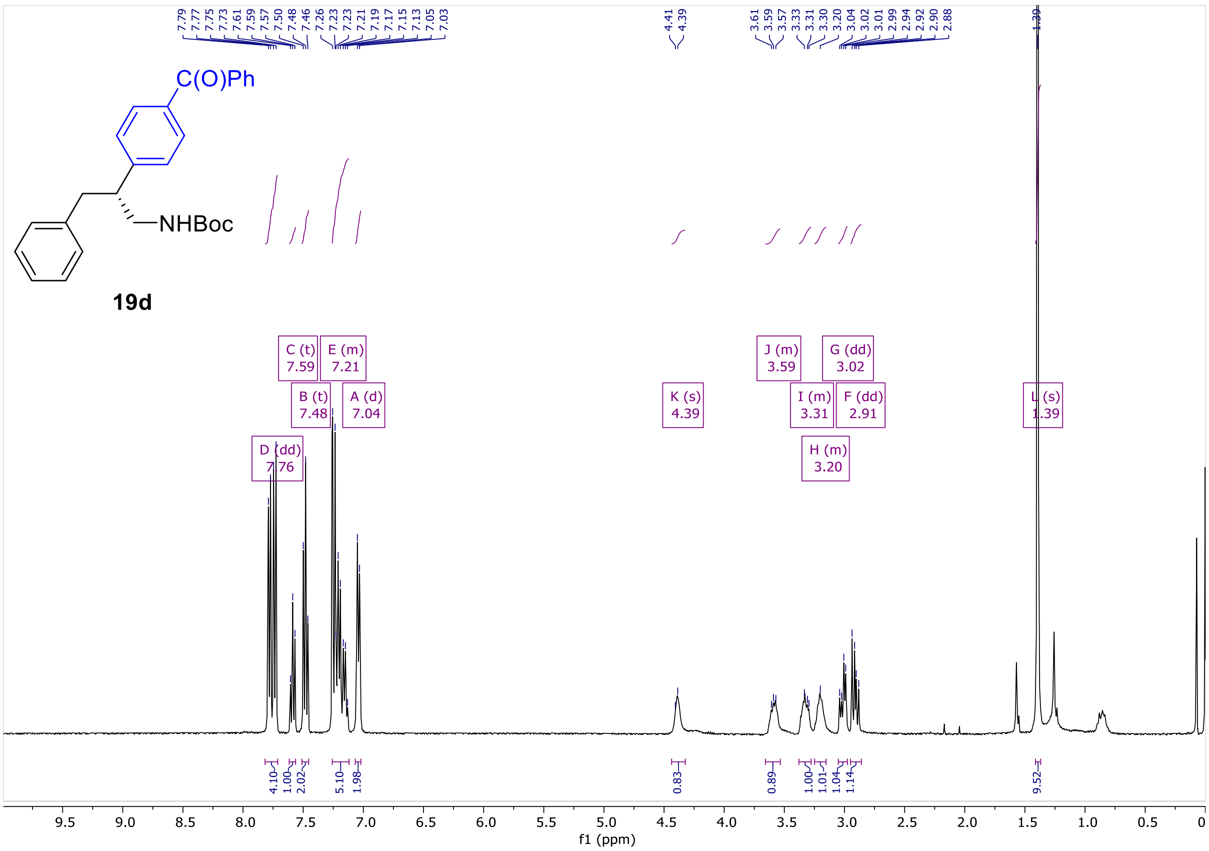

^{13}C-NMR (101 MHz, $CDCl_3$):

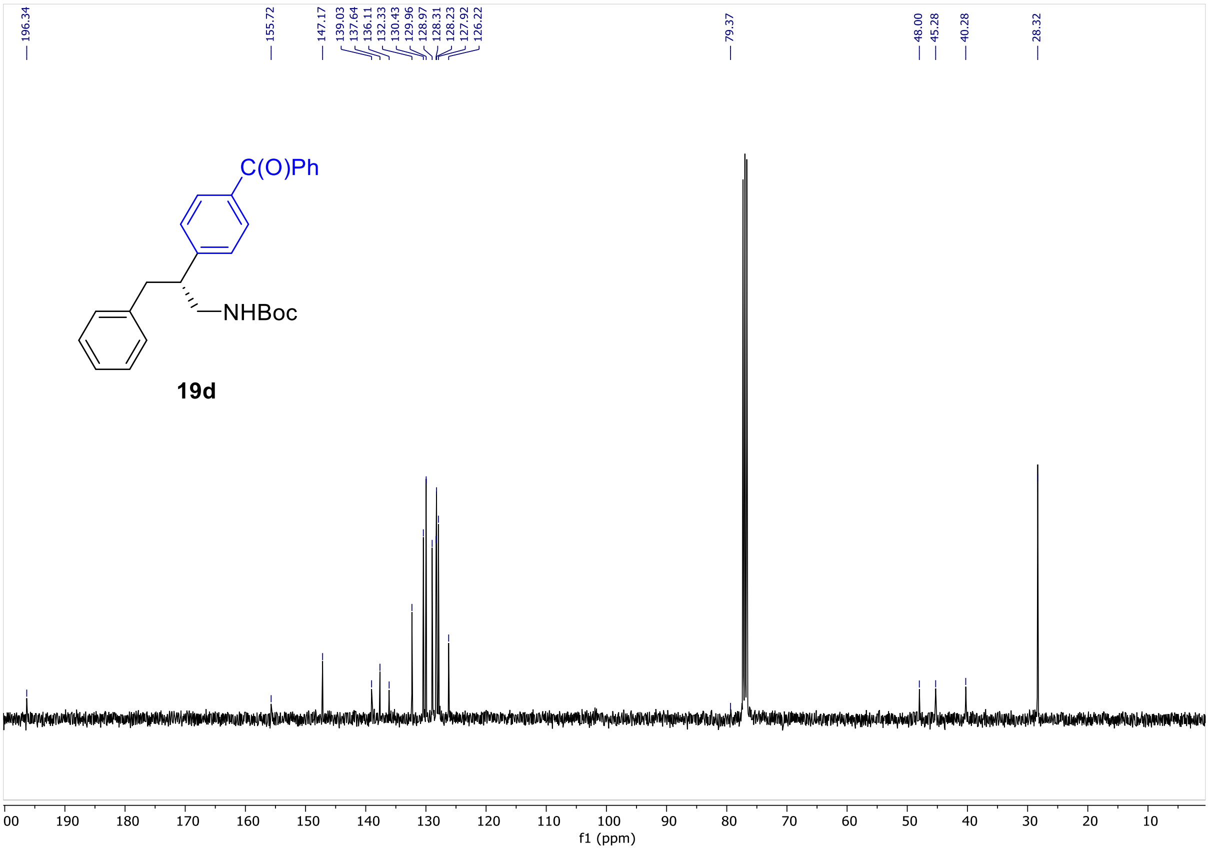

^{1}H-NMR (400 MHz, $CDCl_3$):

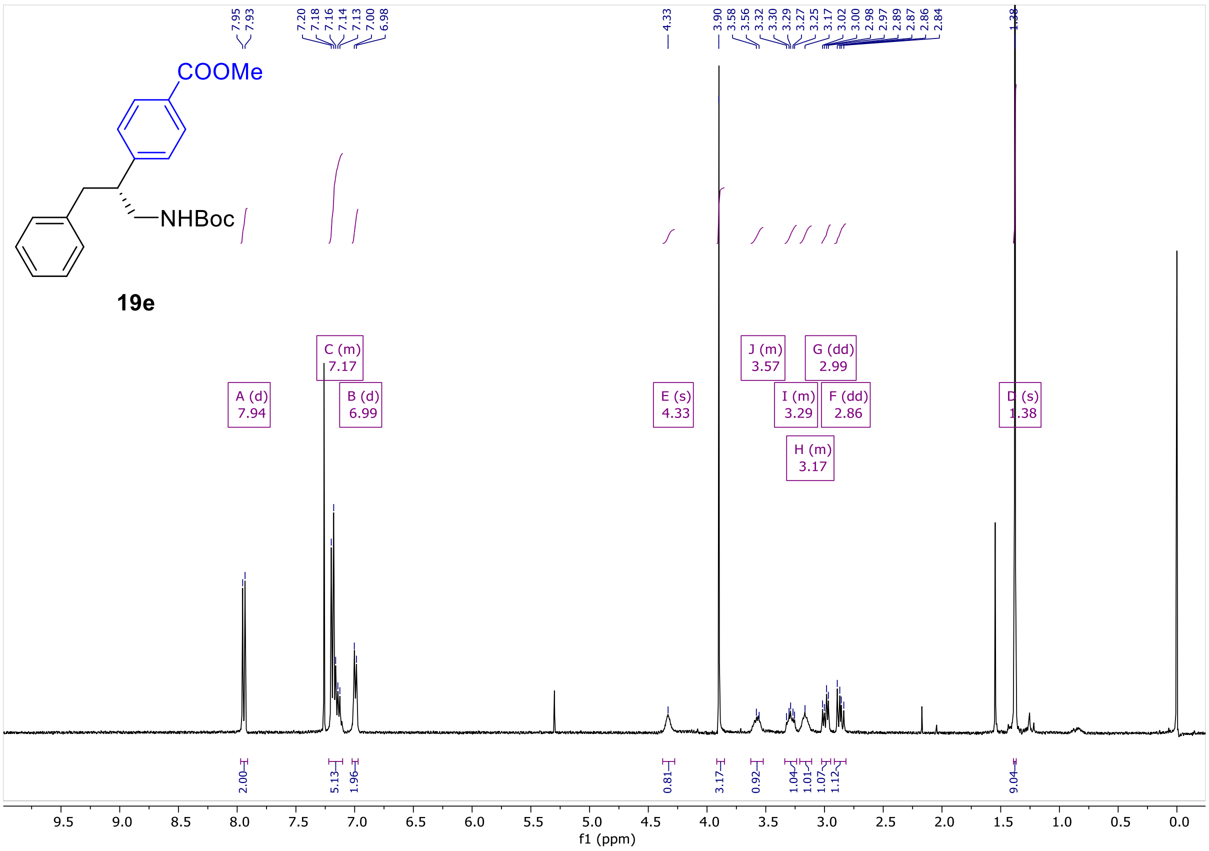

^{13}C-NMR (101 MHz, $CDCl_3$):

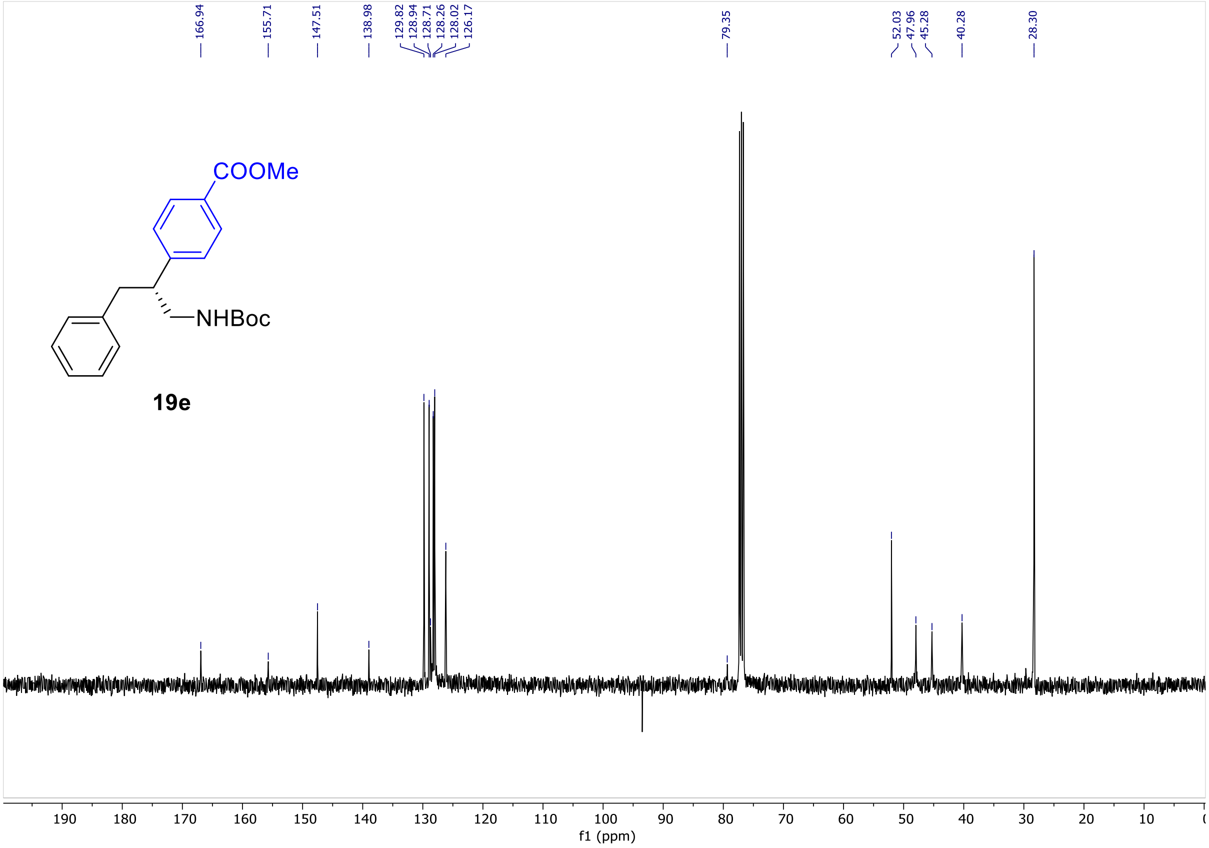

^{1}H-NMR (400 MHz, $CDCl_3$):

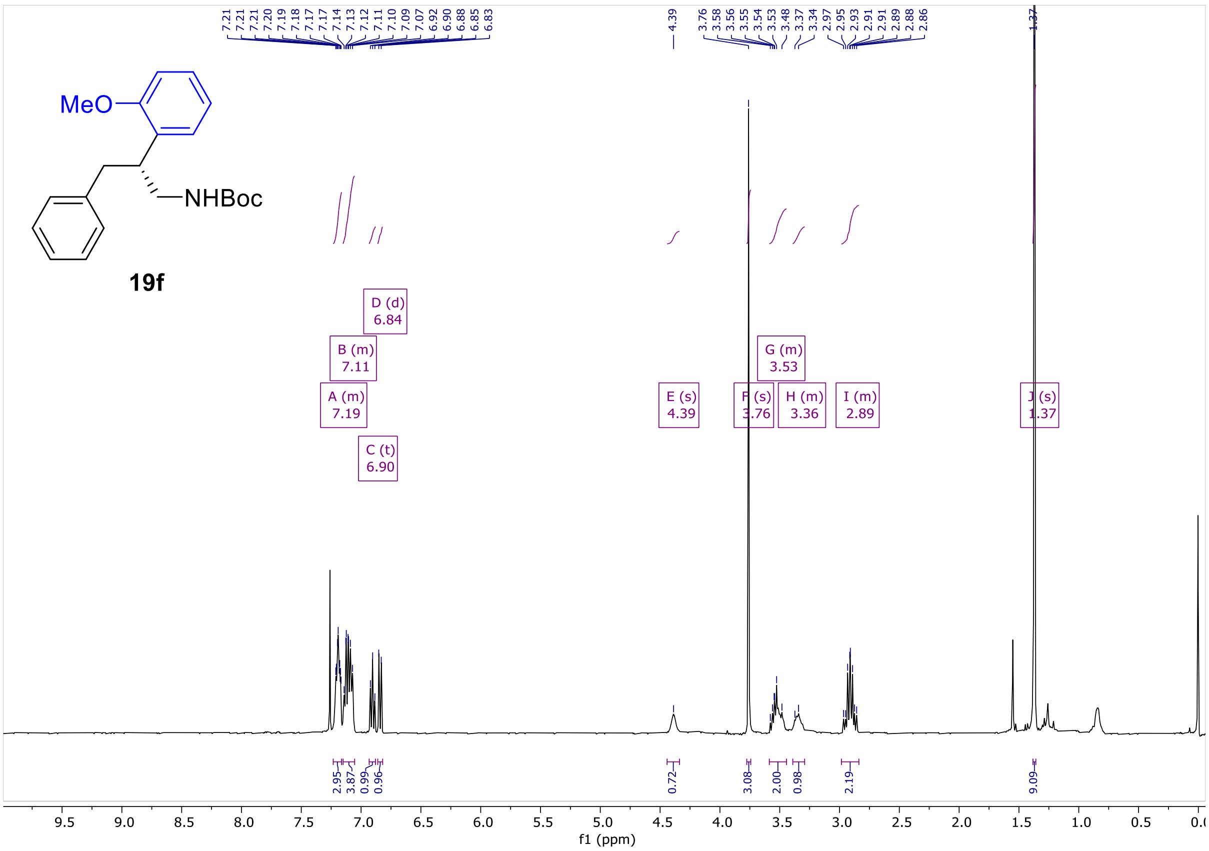

^{13}C-NMR (101 MHz, $CDCl_3$):

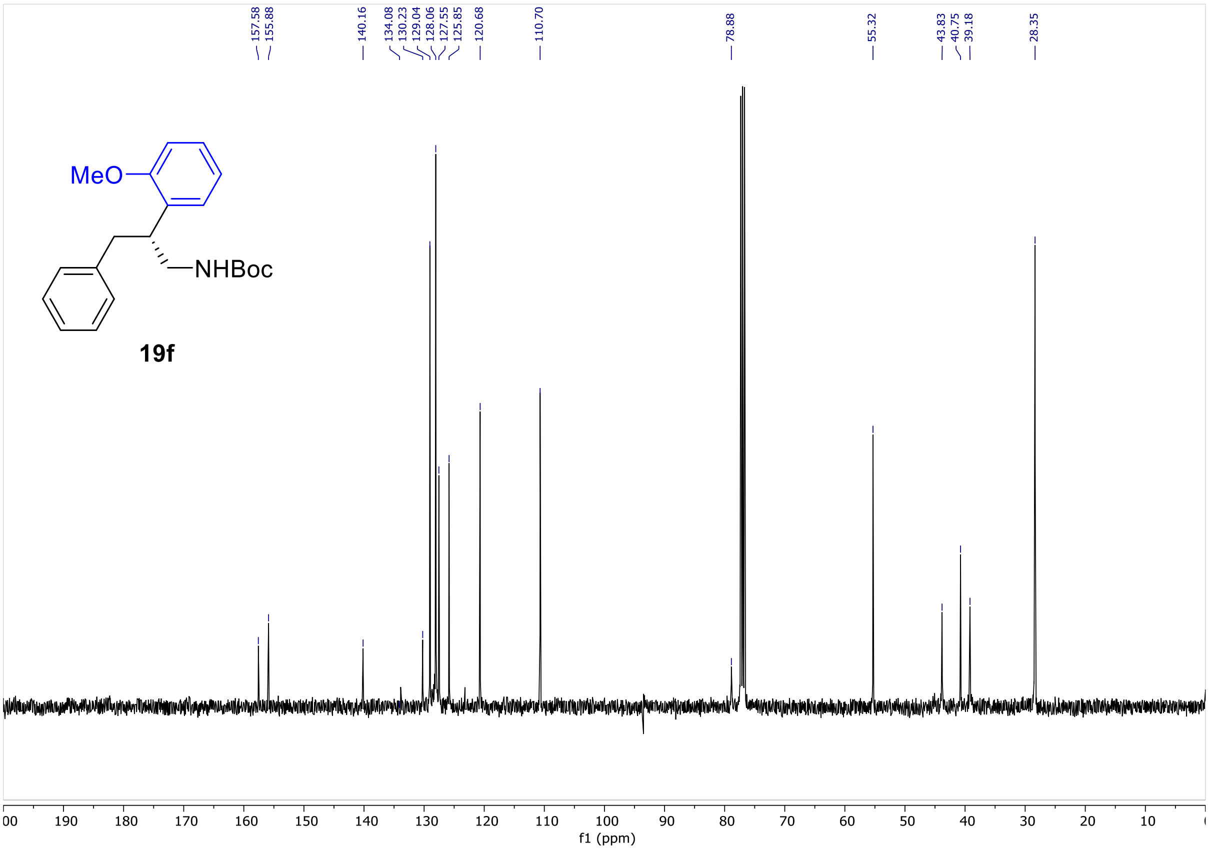

^{1}H-NMR (400 MHz, $CDCl_3$):

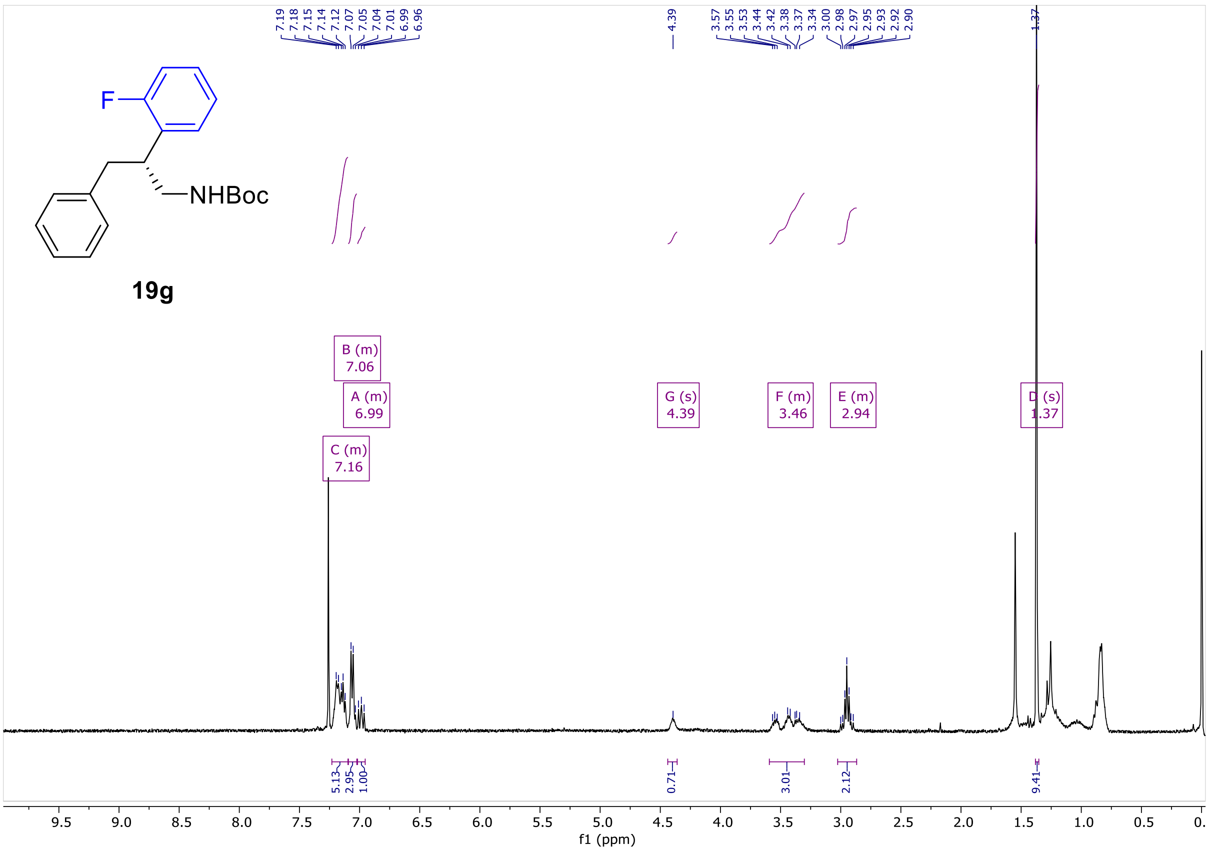

^{13}C-NMR (101 MHz, $CDCl_3$):

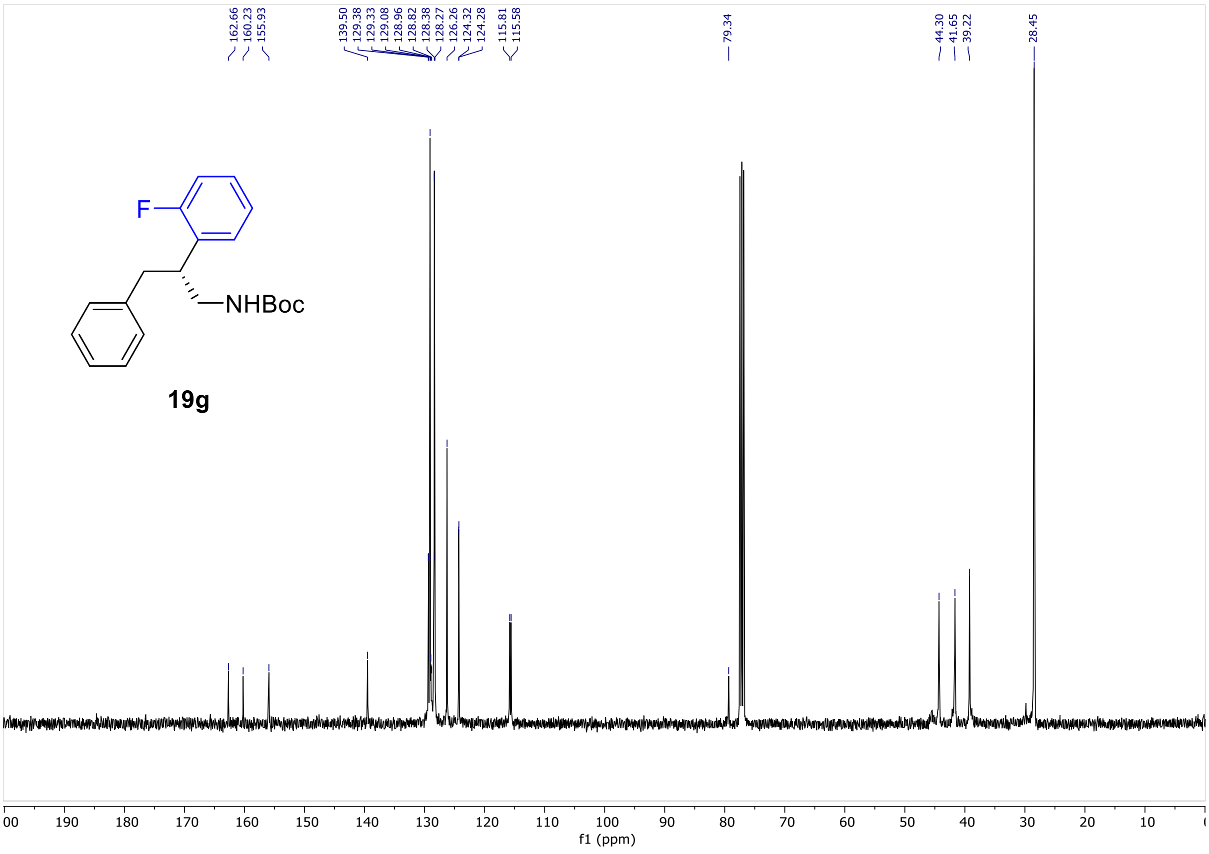

^{1}H-NMR (400 MHz, $CDCl_3$):

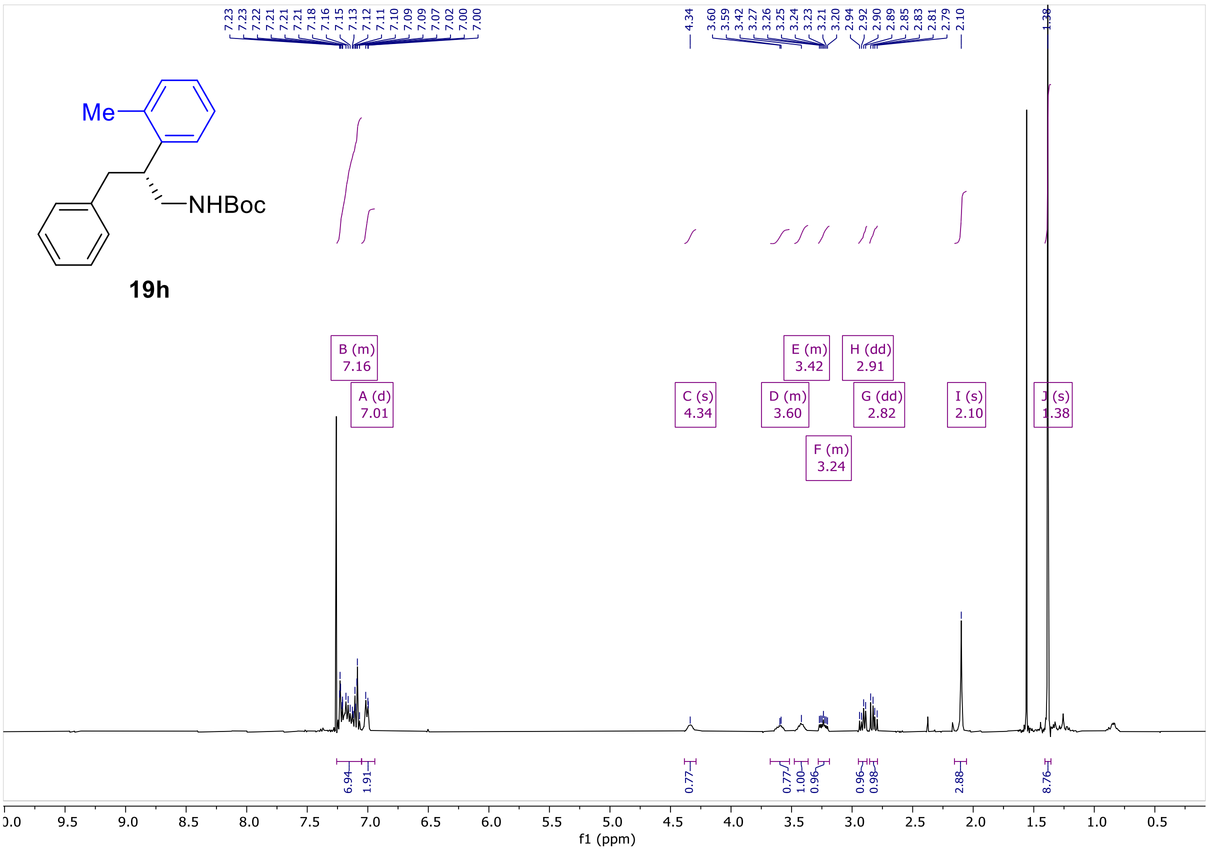

^{13}C-NMR (101 MHz, $CDCl_3$):

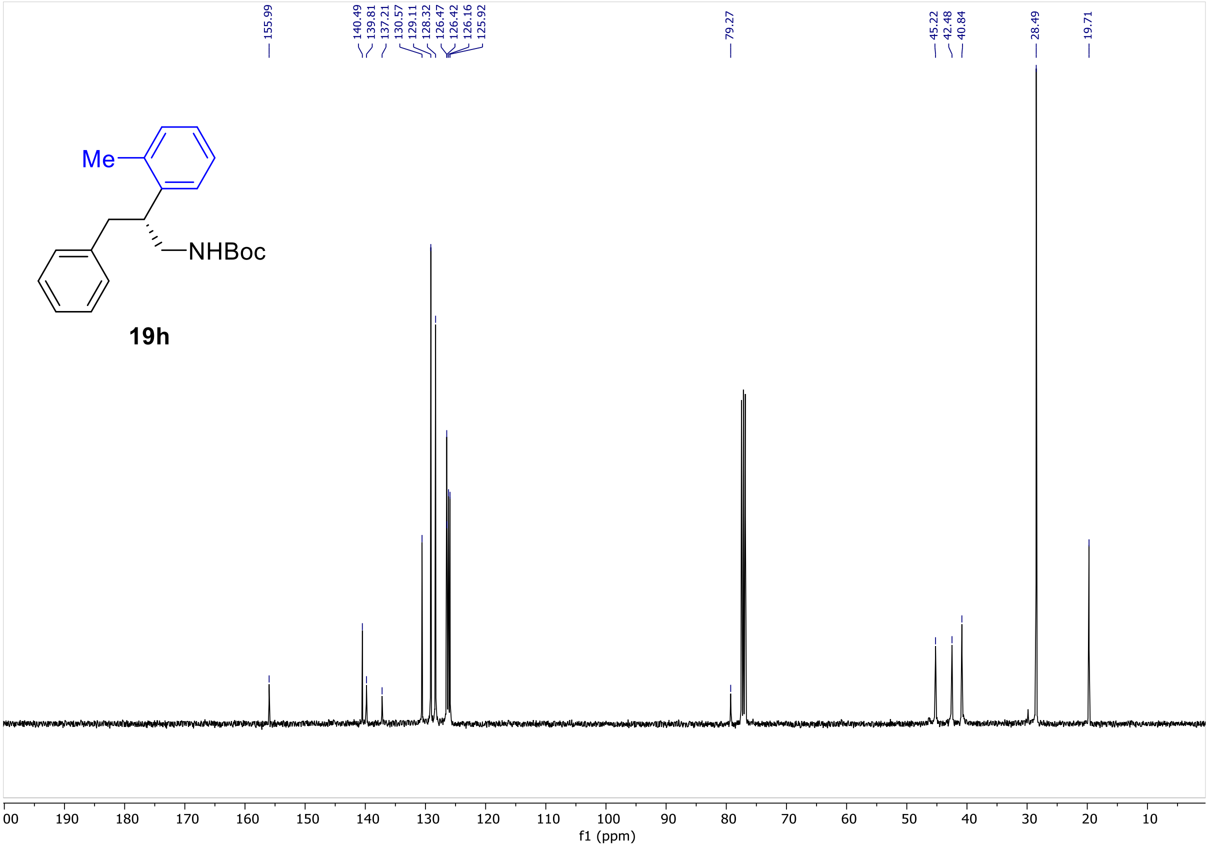

^{1}H-NMR (400 MHz, $CDCl_3$):

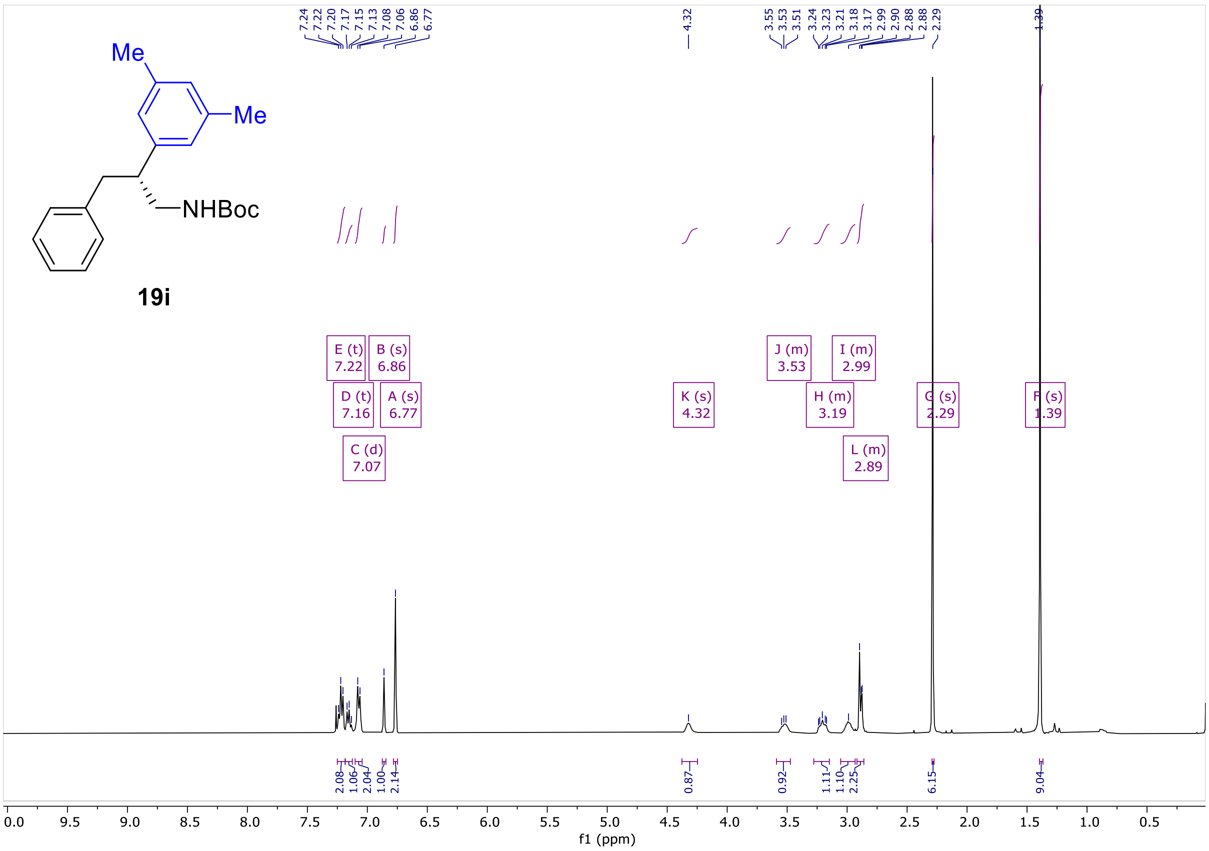

^{13}C-NMR (101 MHz, $CDCl_3$):

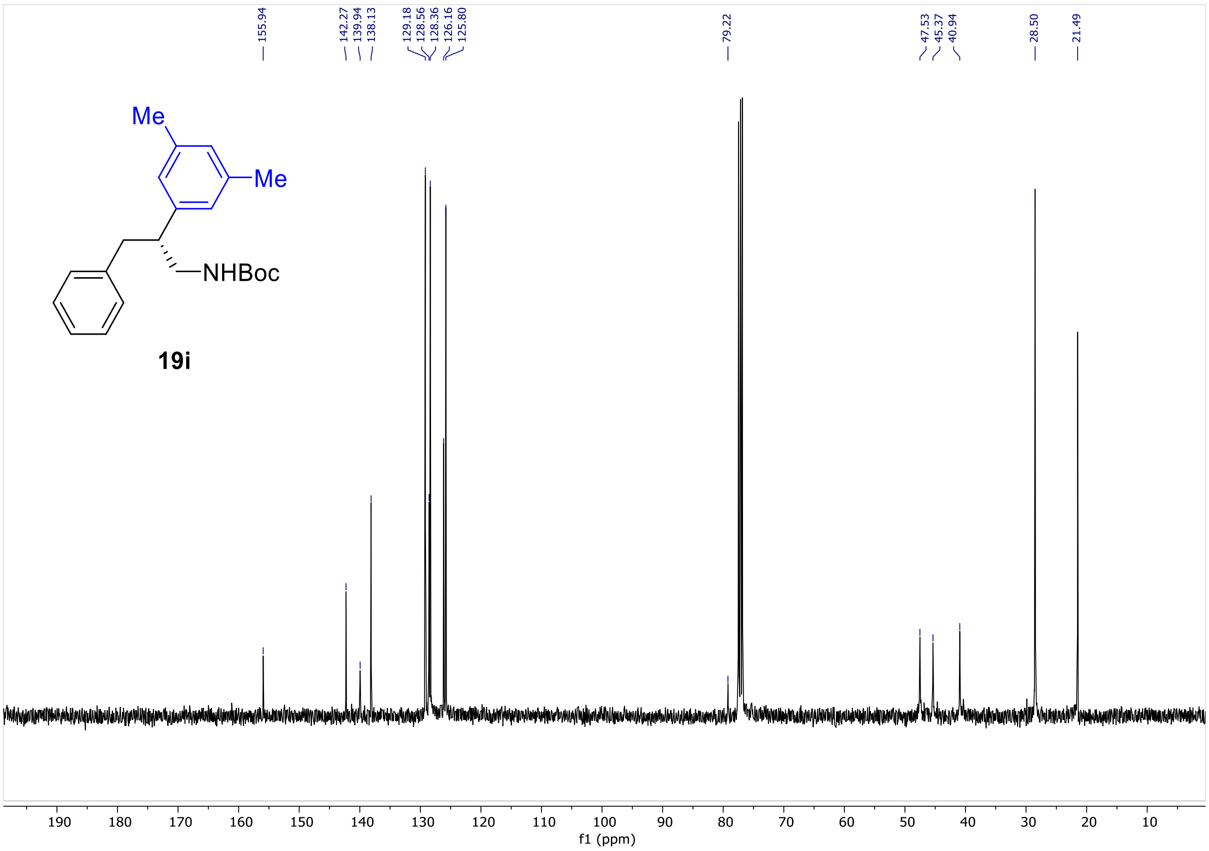

^{1}H-NMR (400 MHz, CDCl$_3$):

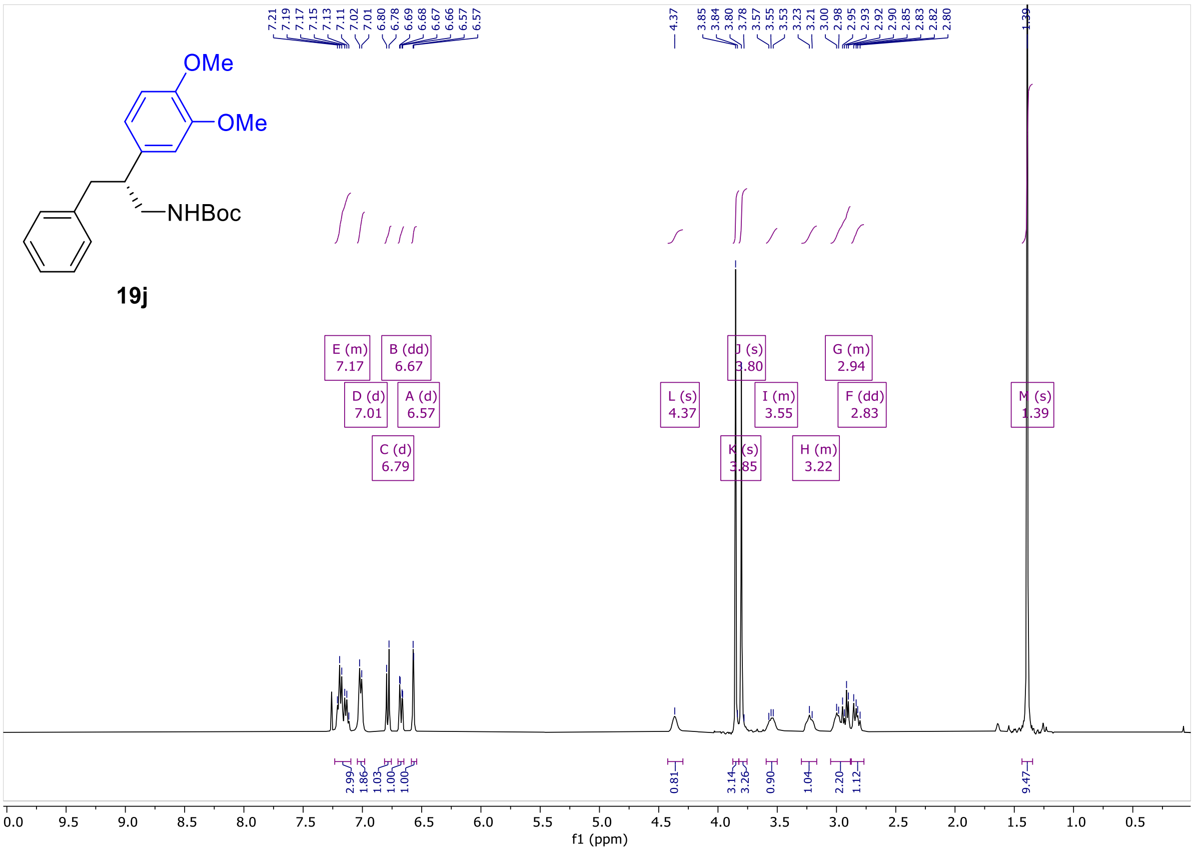

^{13}C-NMR (101 MHz, CDCl$_3$):

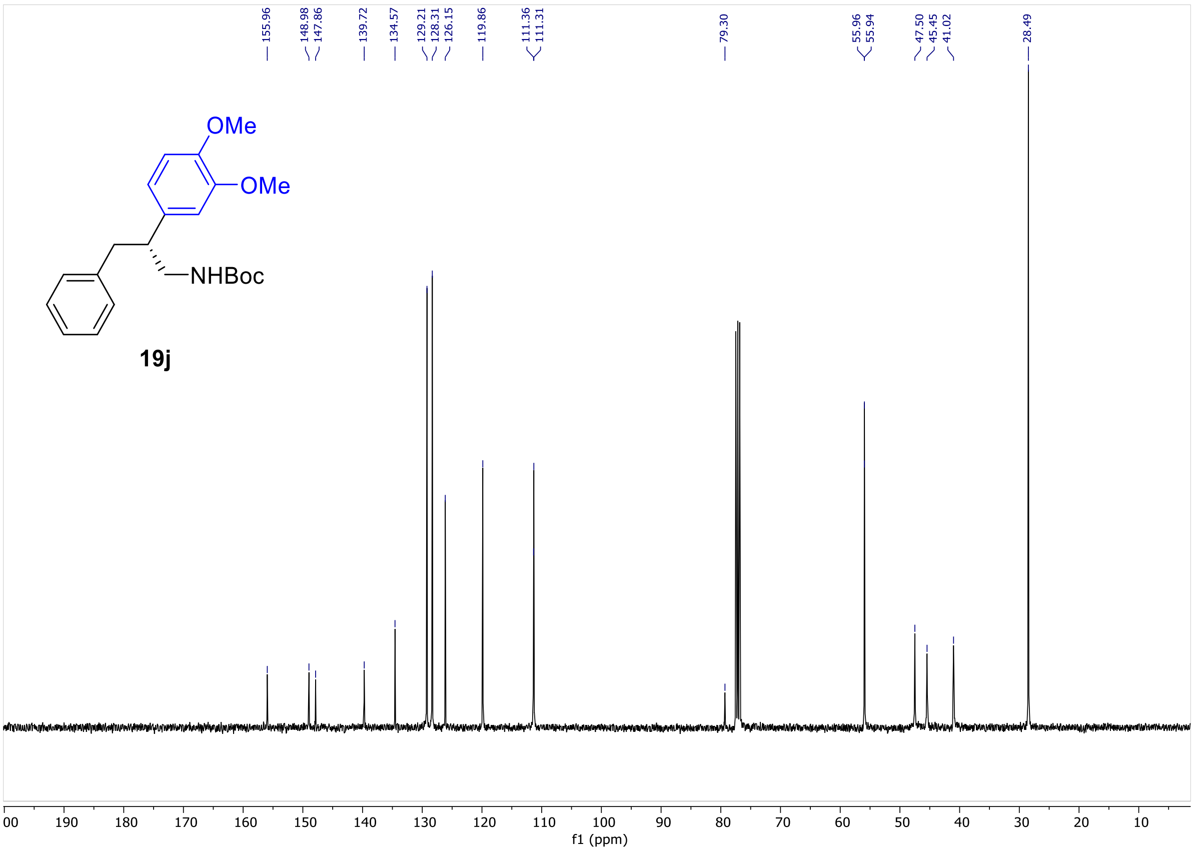

^{1}H-NMR (400 MHz, $CDCl_3$):

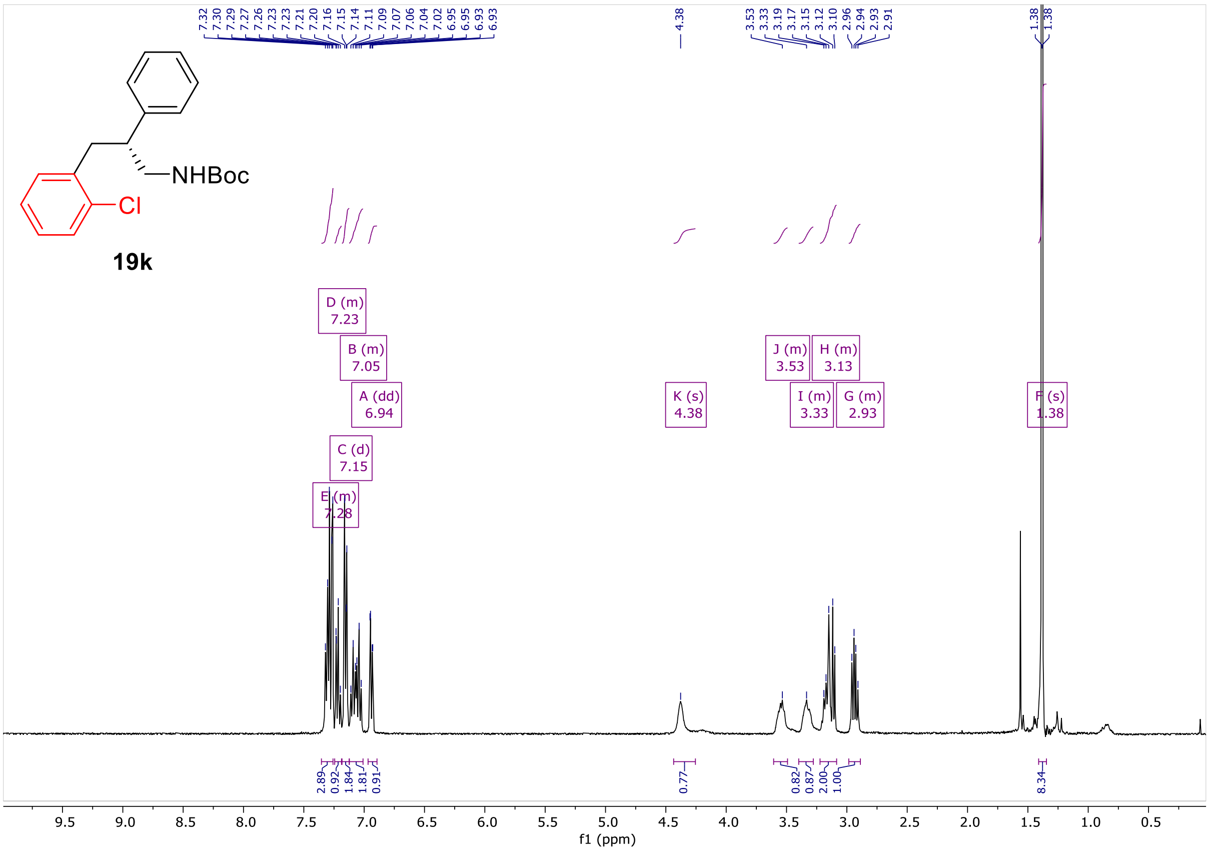

^{13}C-NMR (101 MHz, $CDCl_3$):

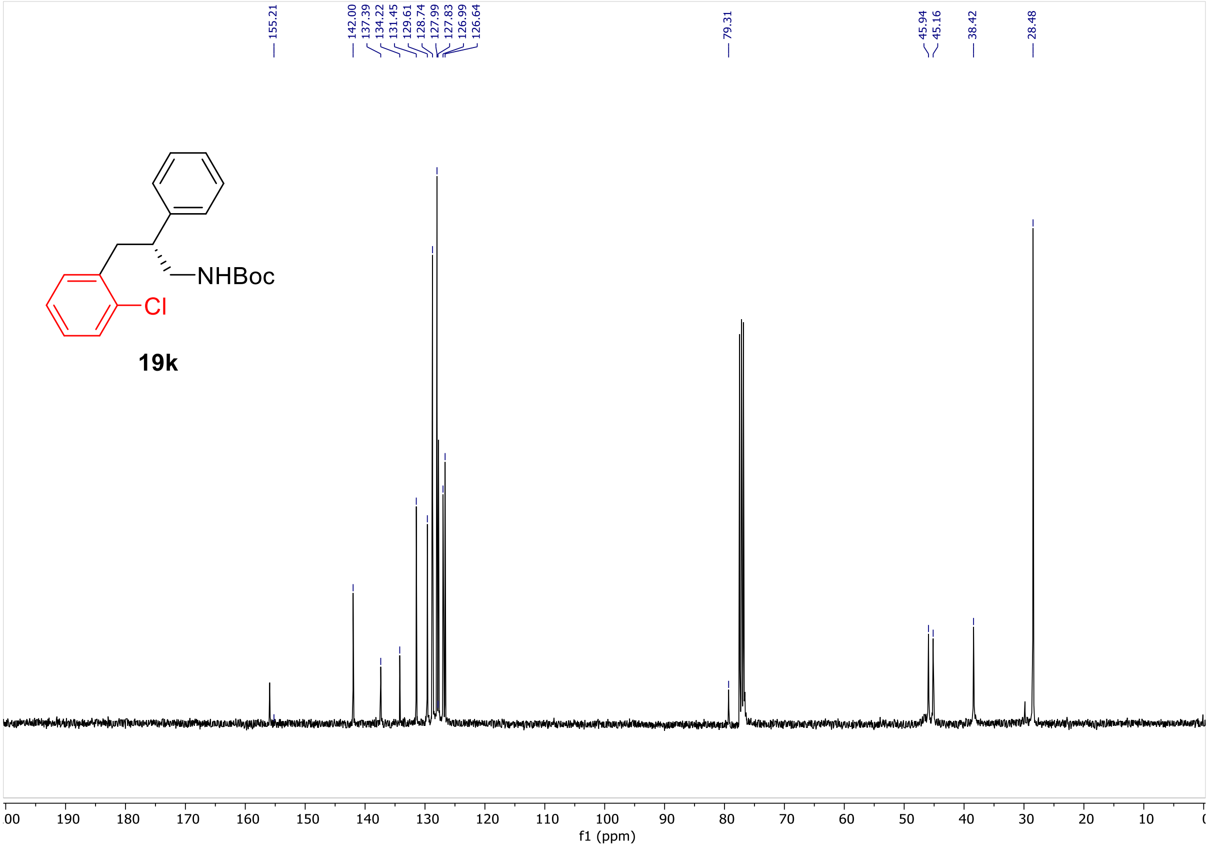

^{1}H-NMR (400 MHz, $CDCl_3$):

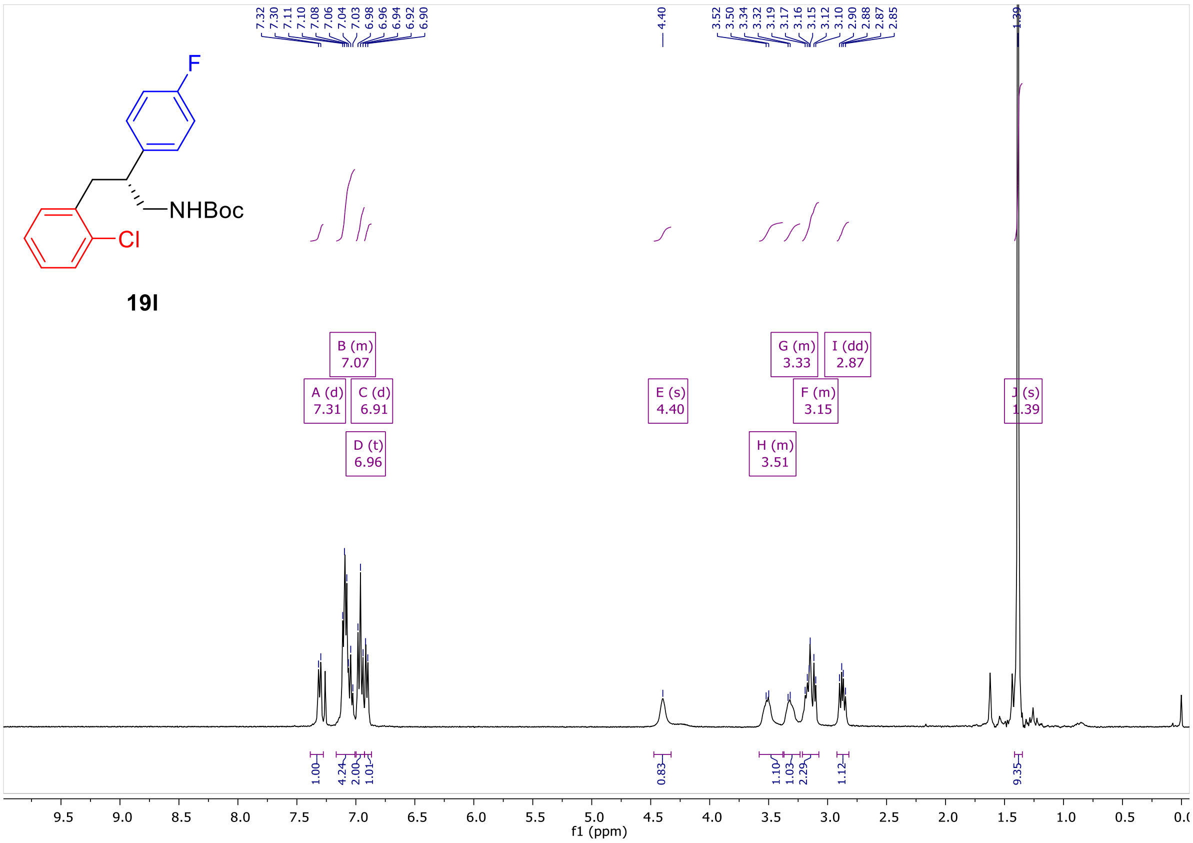

^{13}C-NMR (101 MHz, $CDCl_3$):

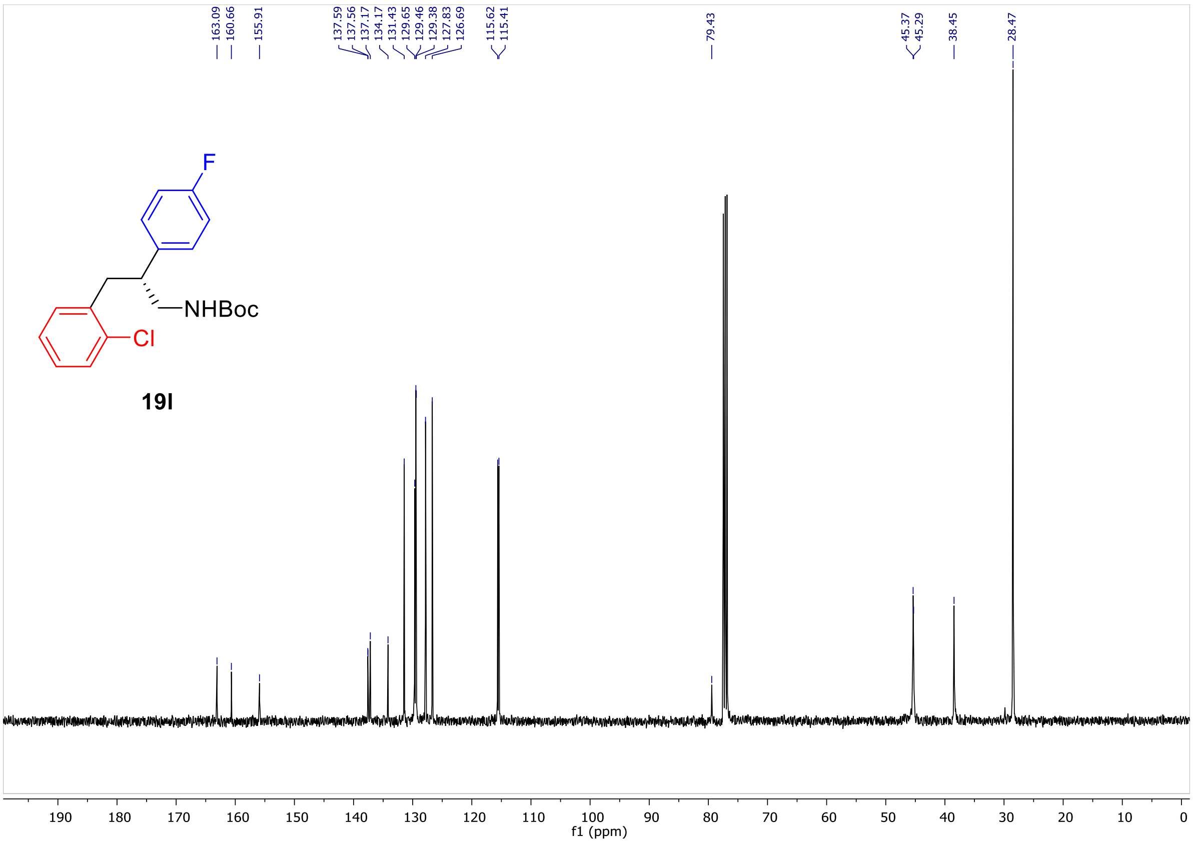

^{1}H-NMR (400 MHz, $CDCl_3$):

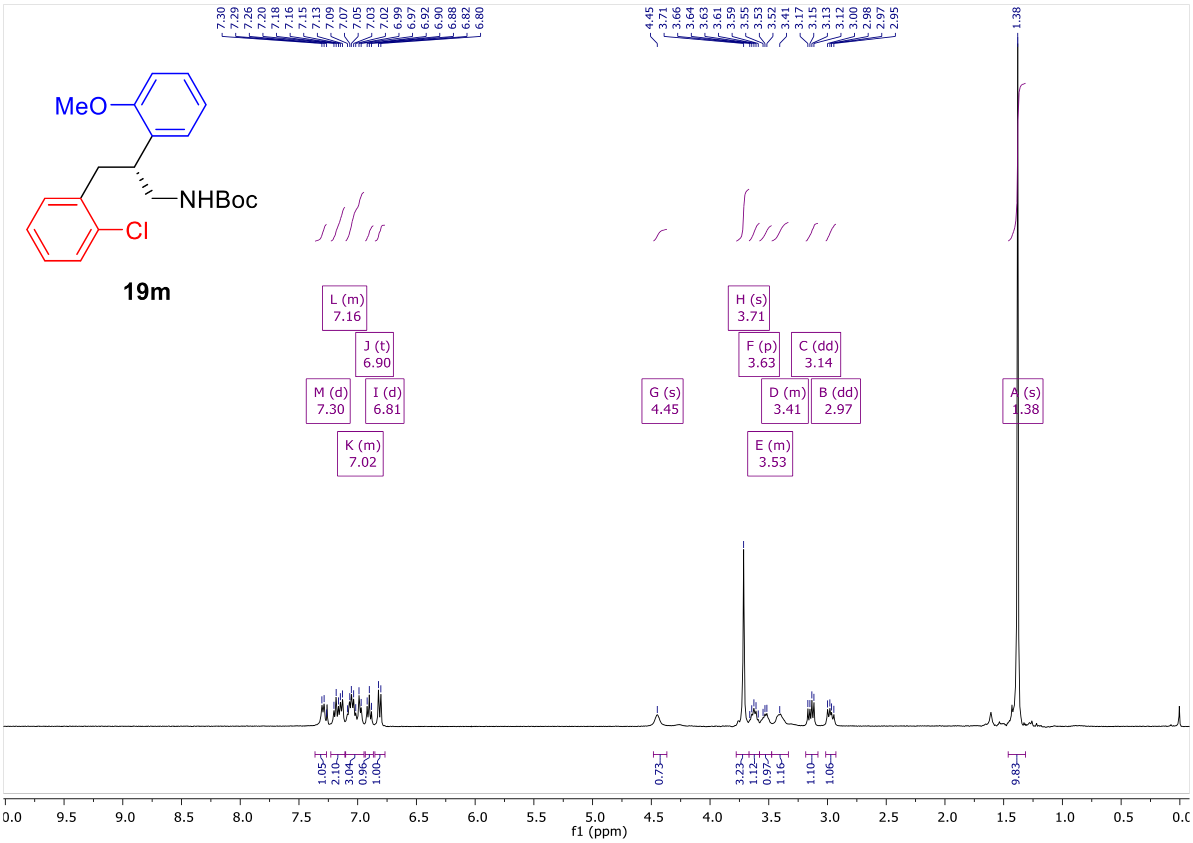

^{13}C-NMR (101 MHz, $CDCl_3$):

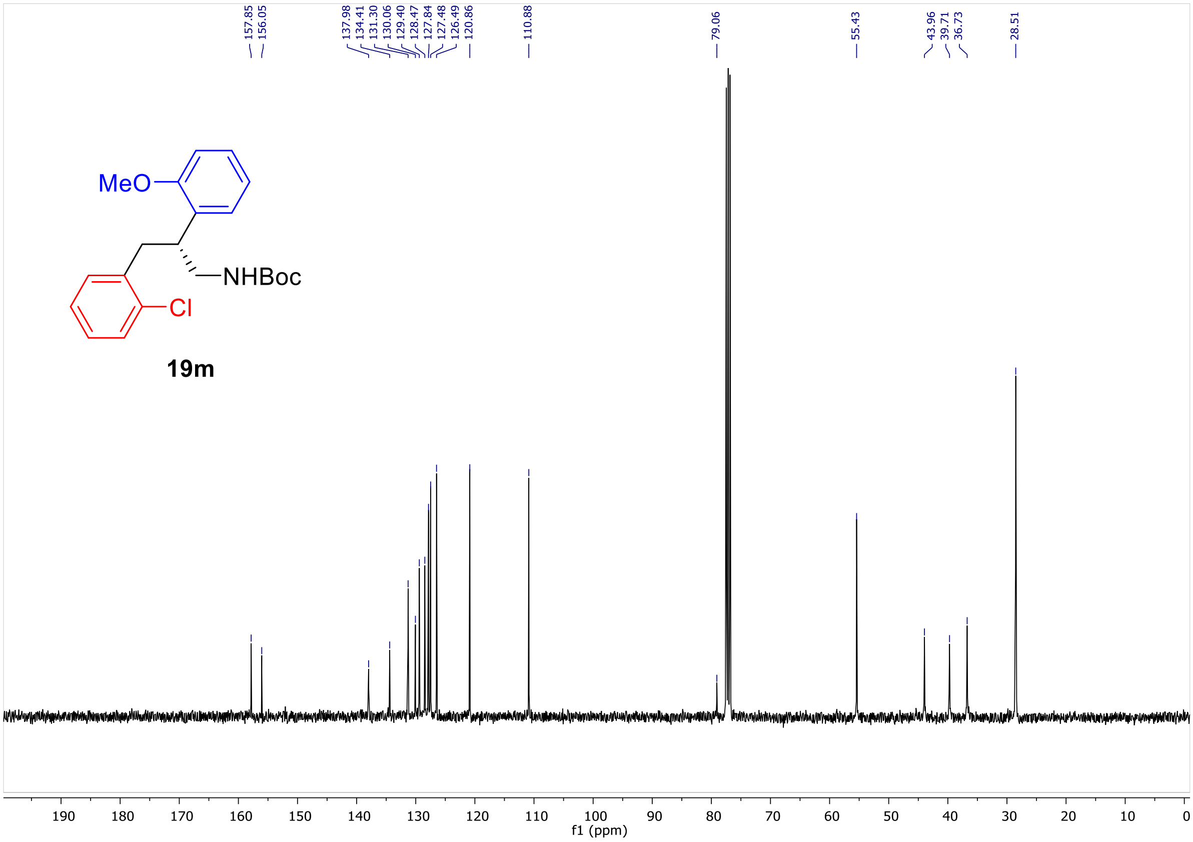

^{1}H-NMR (400 MHz, $CDCl_3$):

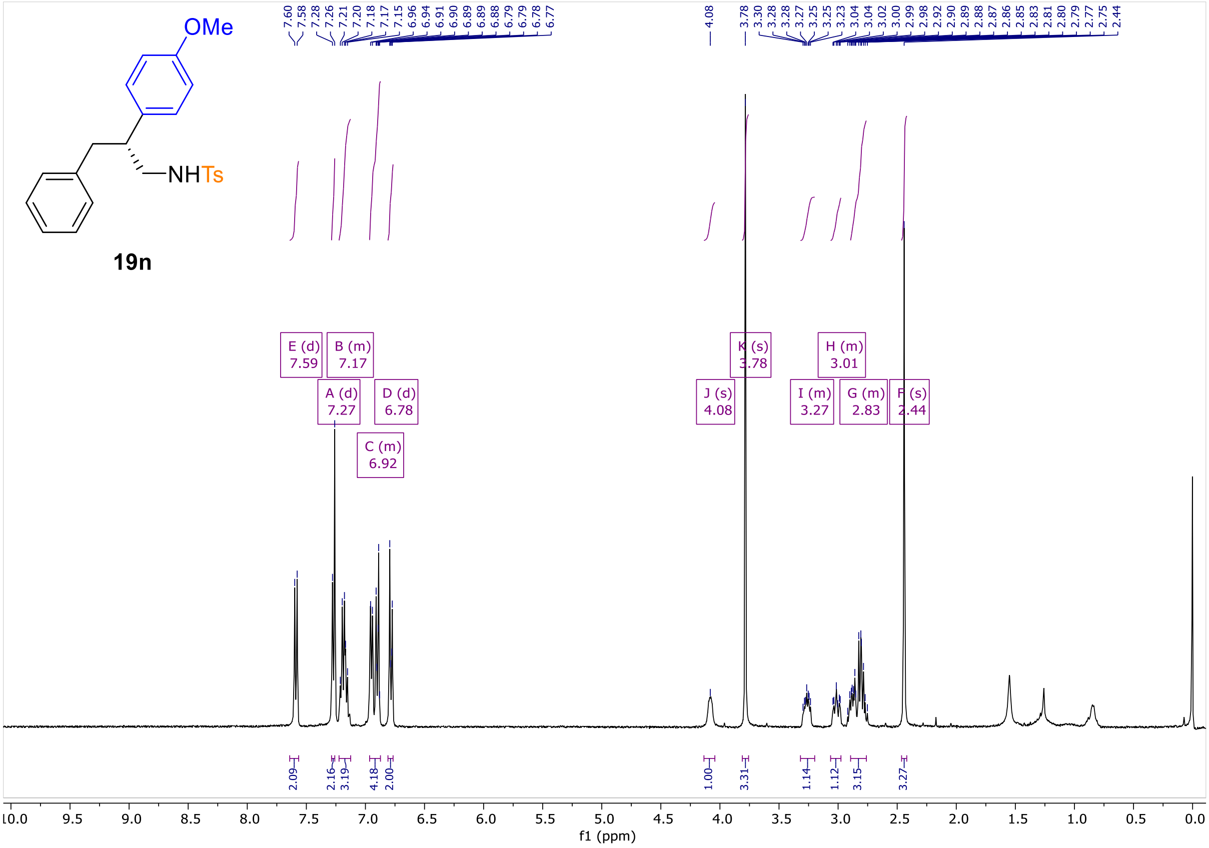

^{13}C-NMR (101 MHz, $CDCl_3$):

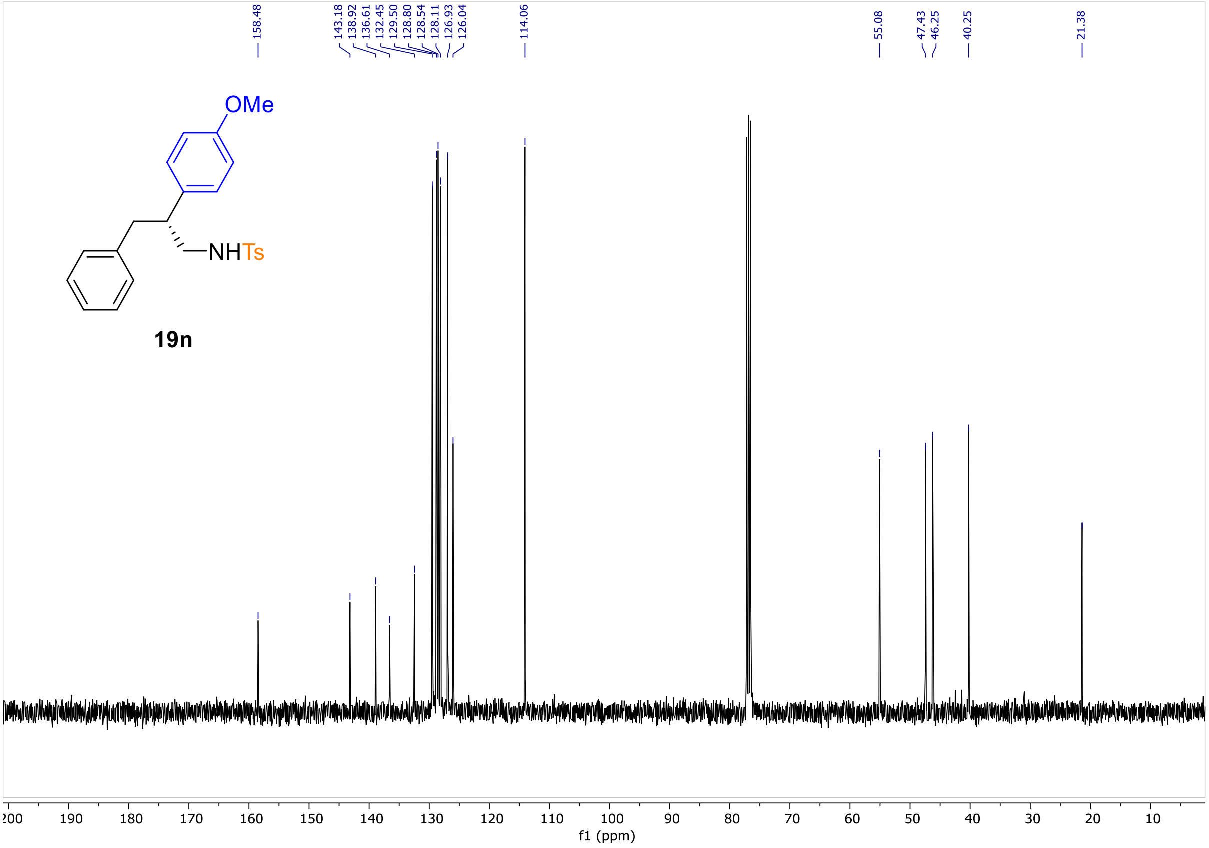

^{1}H-NMR (400 MHz, $CDCl_3$):

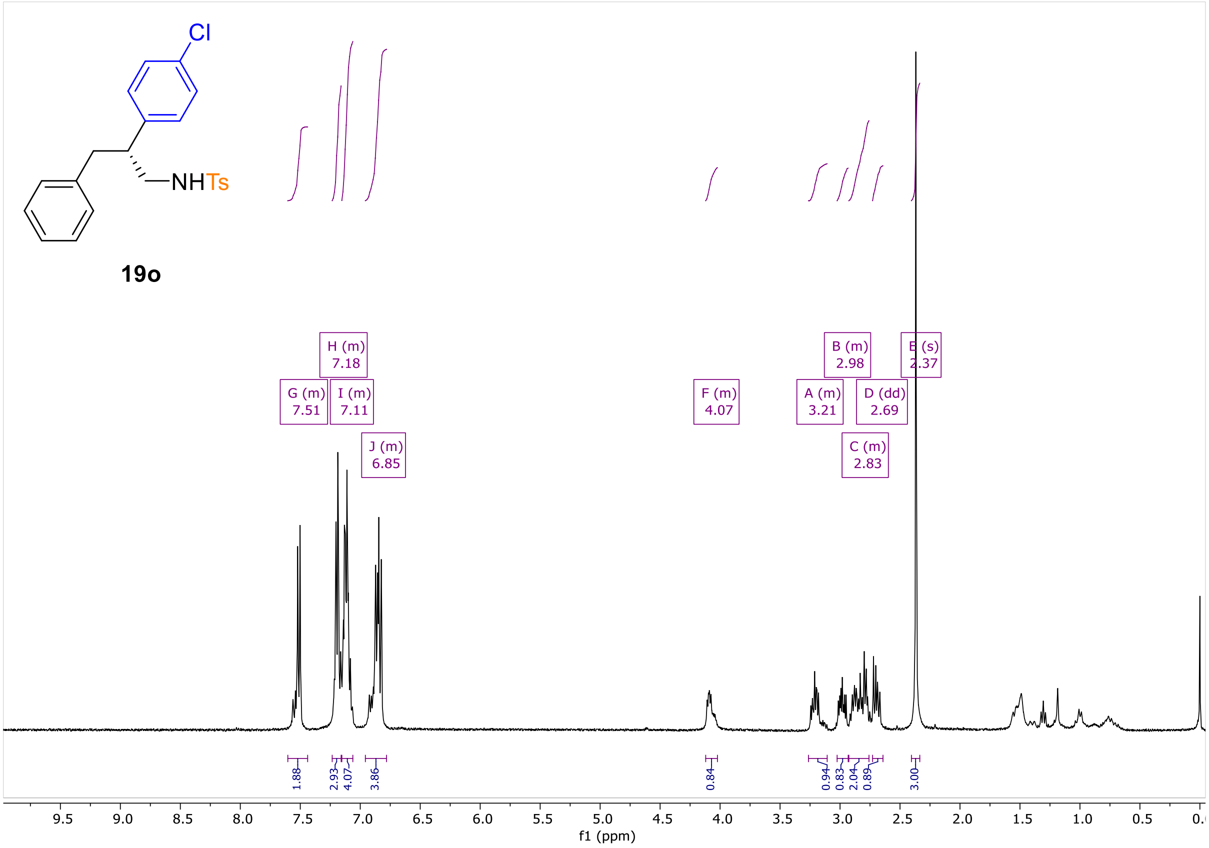

^{13}C-NMR (101 MHz, $CDCl_3$):

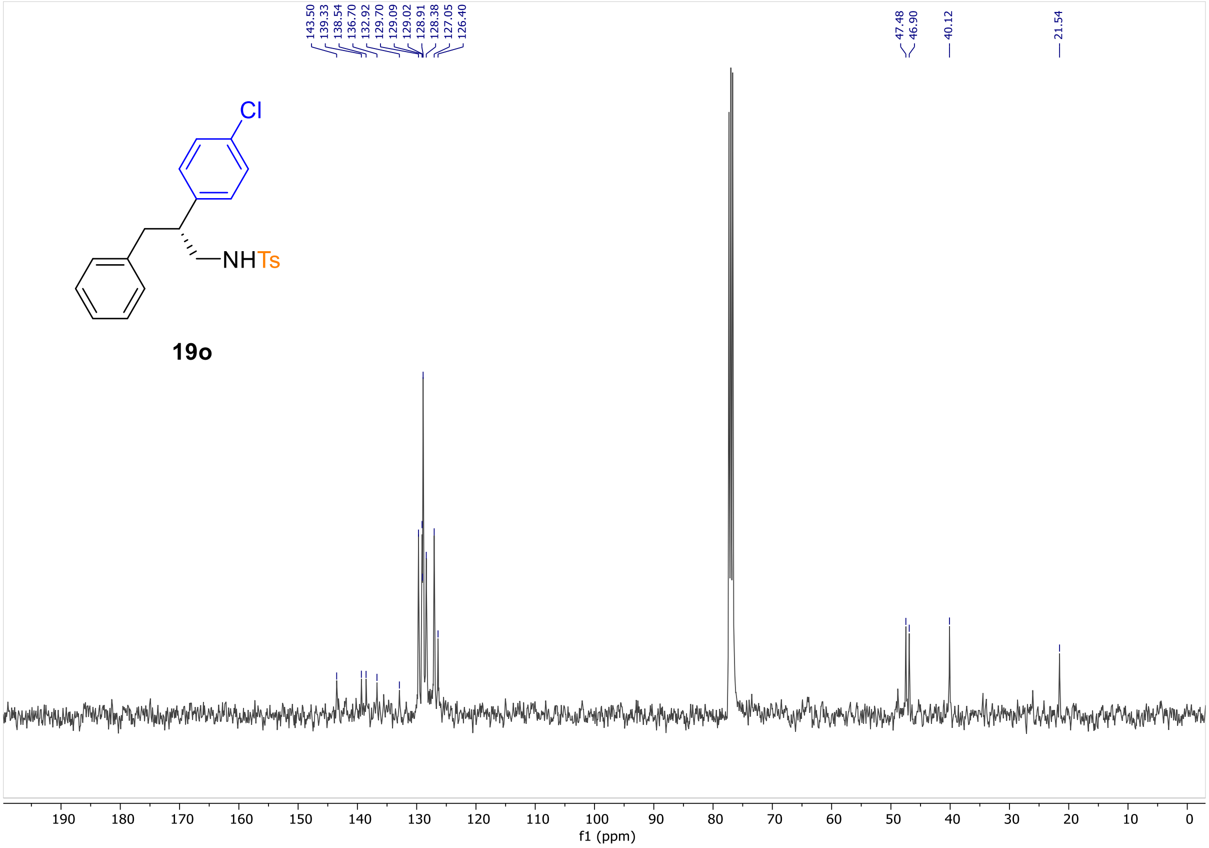

^{1}H-NMR (400 MHz, $CDCl_3$):

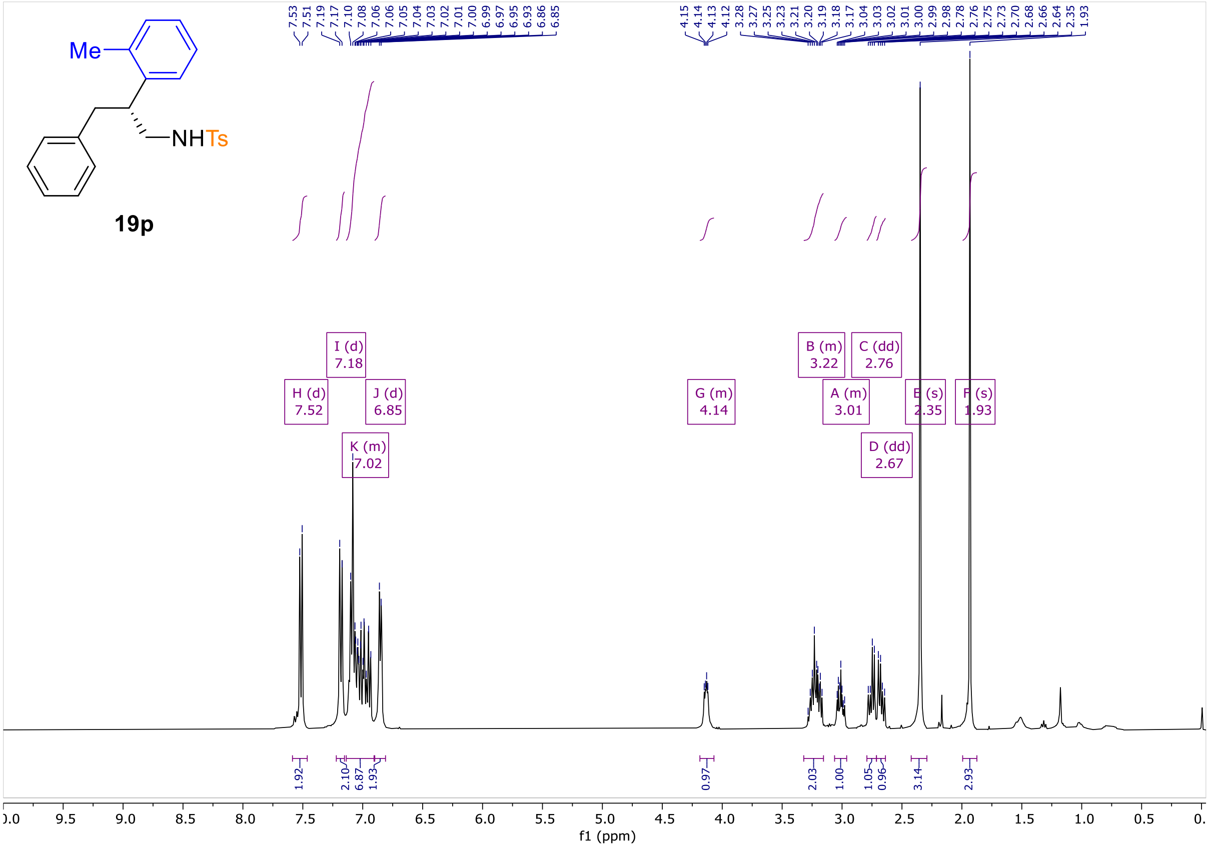

^{13}C-NMR (101 MHz, $CDCl_3$):

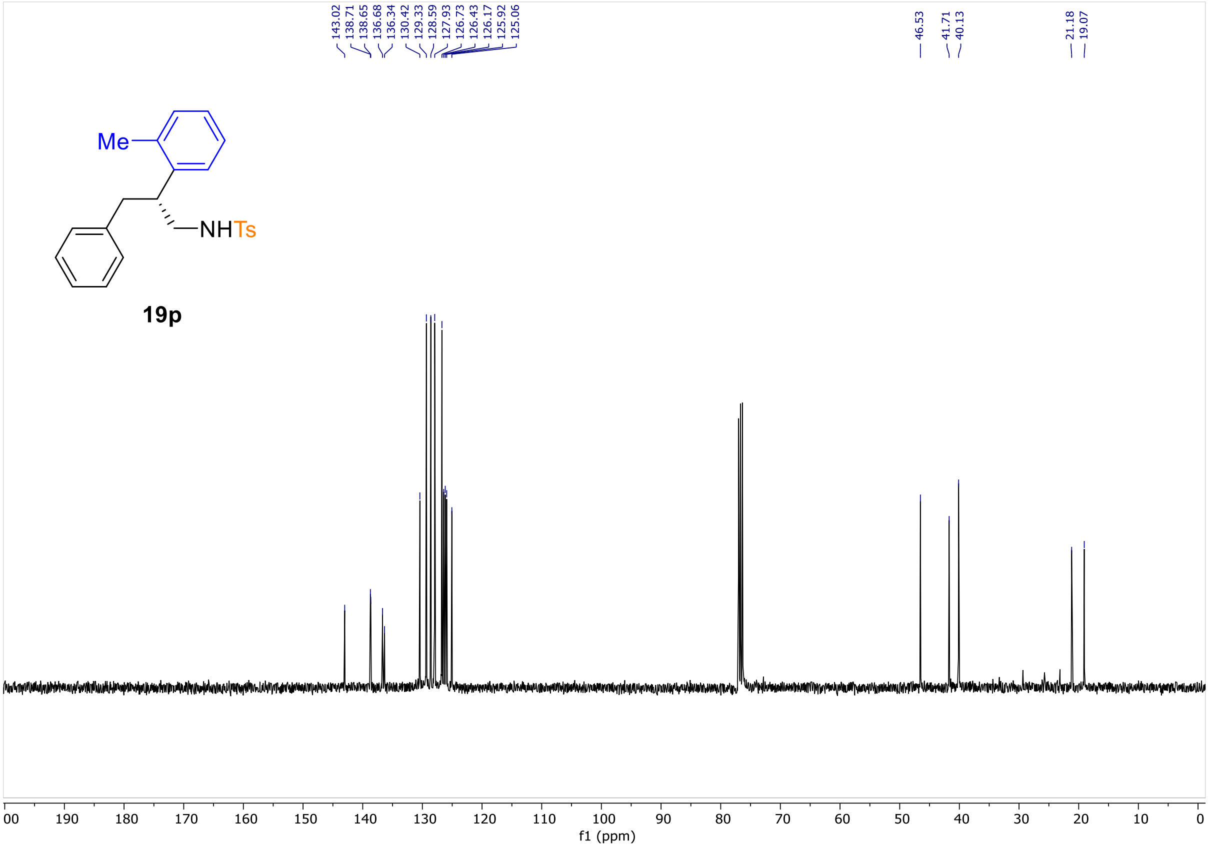

^{1}H-NMR (400 MHz, $CDCl_3$):

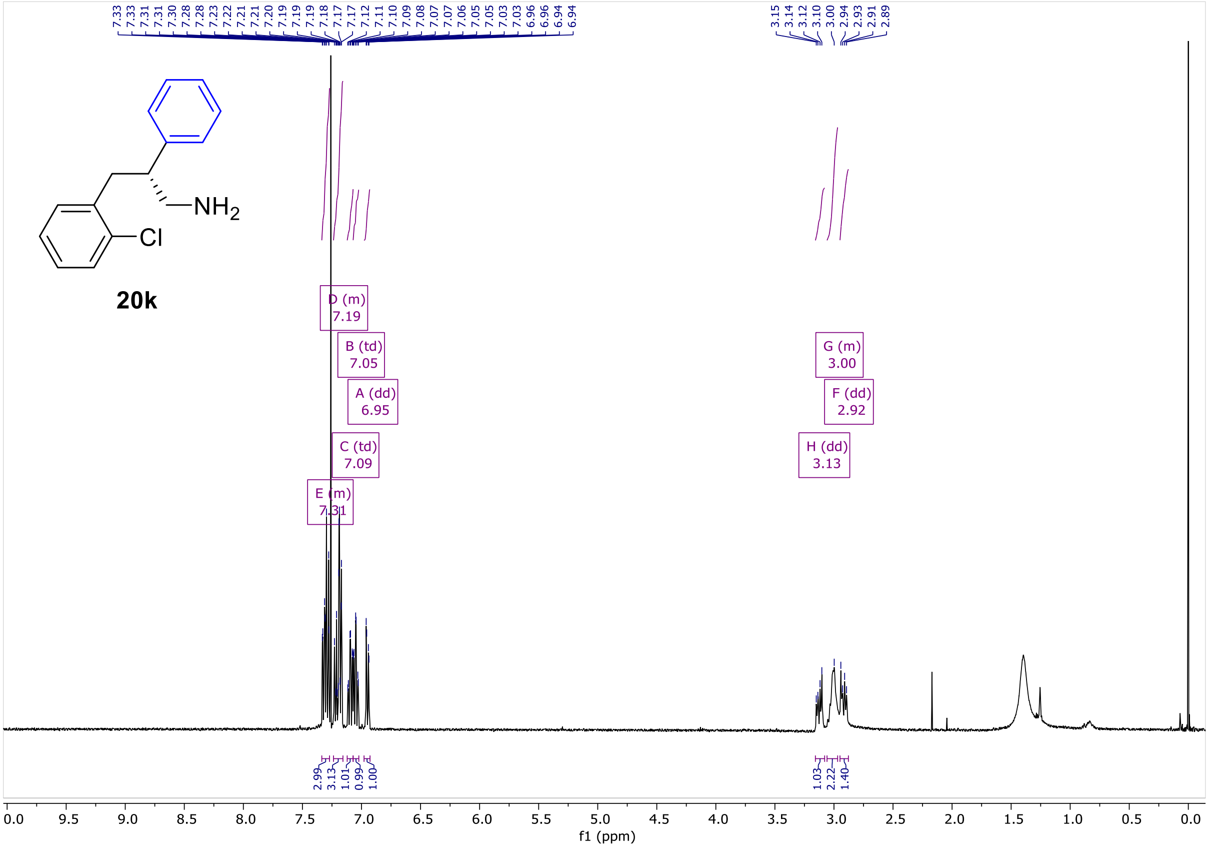

^{13}C-NMR (101 MHz, $CDCl_3$):

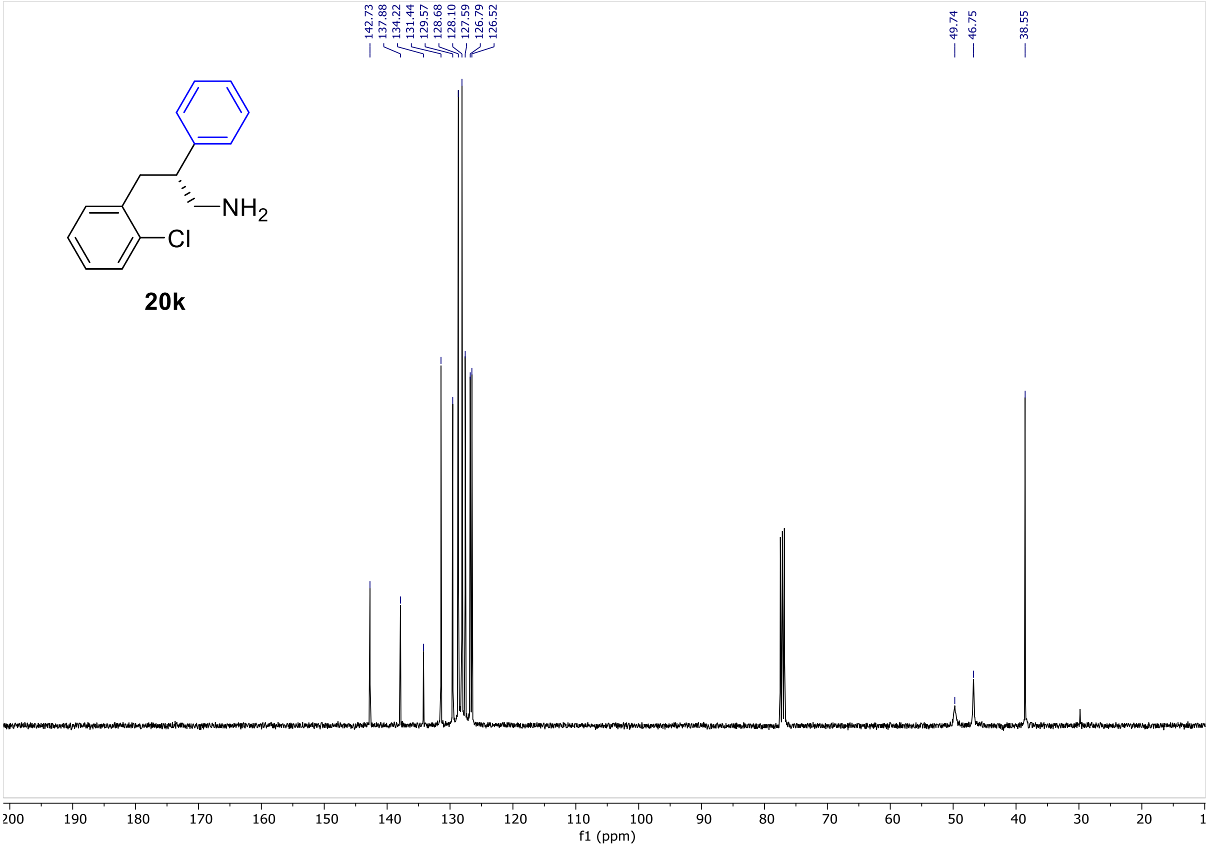

^{1}H-NMR (400 MHz, CDCl$_3$):

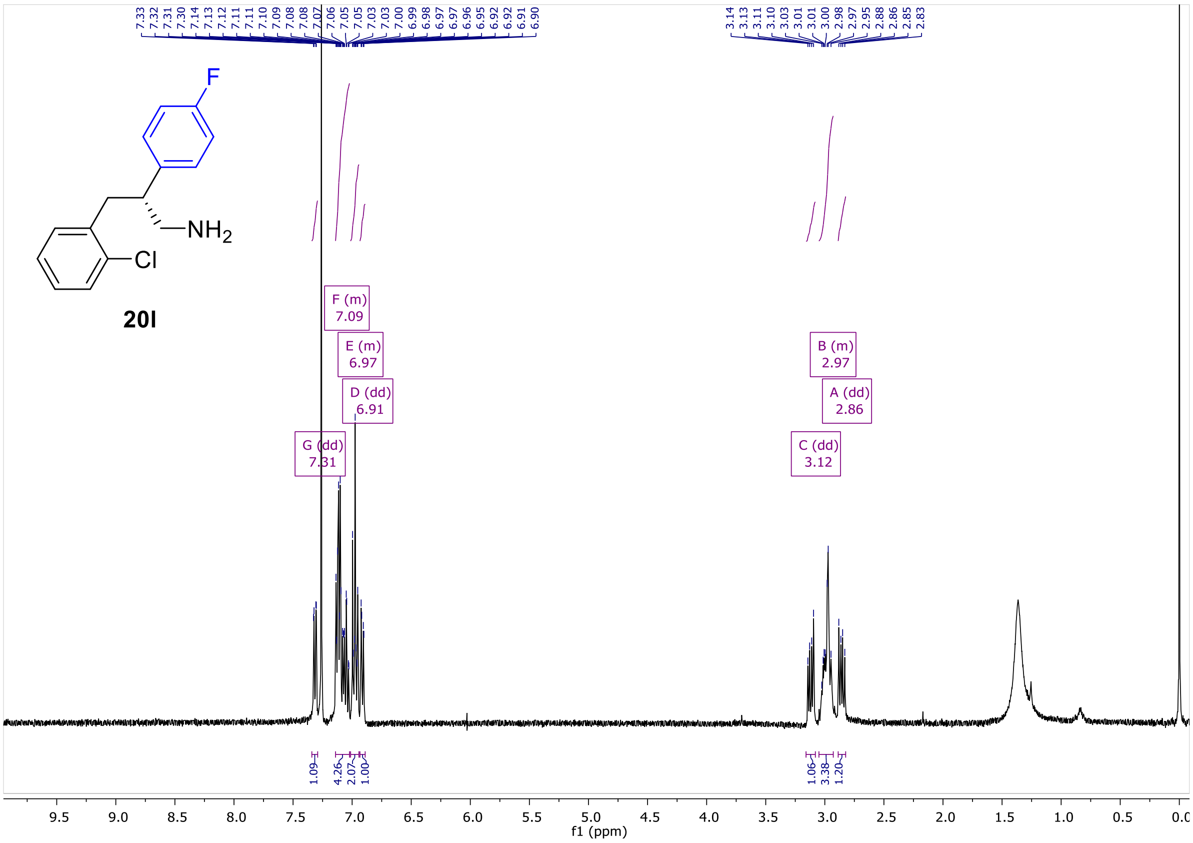

^{13}C-NMR (101 MHz, CDCl$_3$):

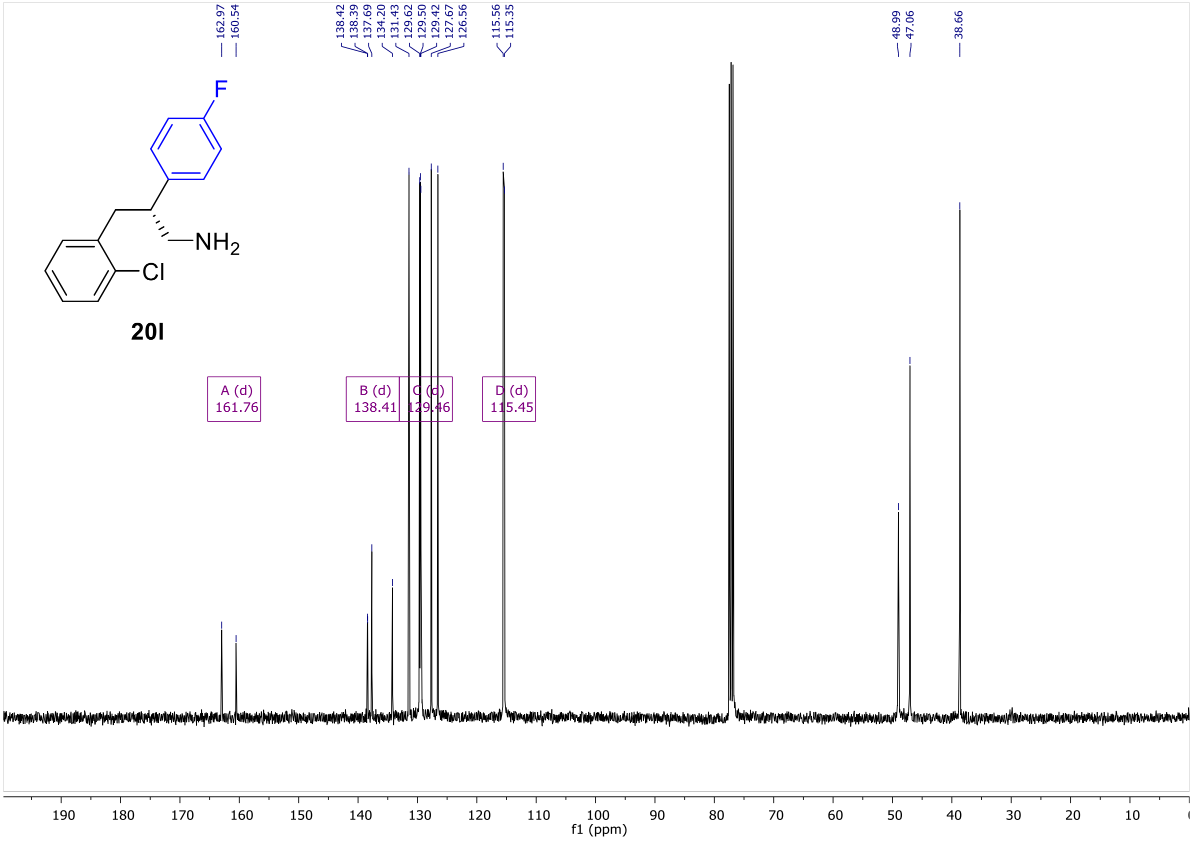

^{1}H-NMR (400 MHz, $CDCl_3$):

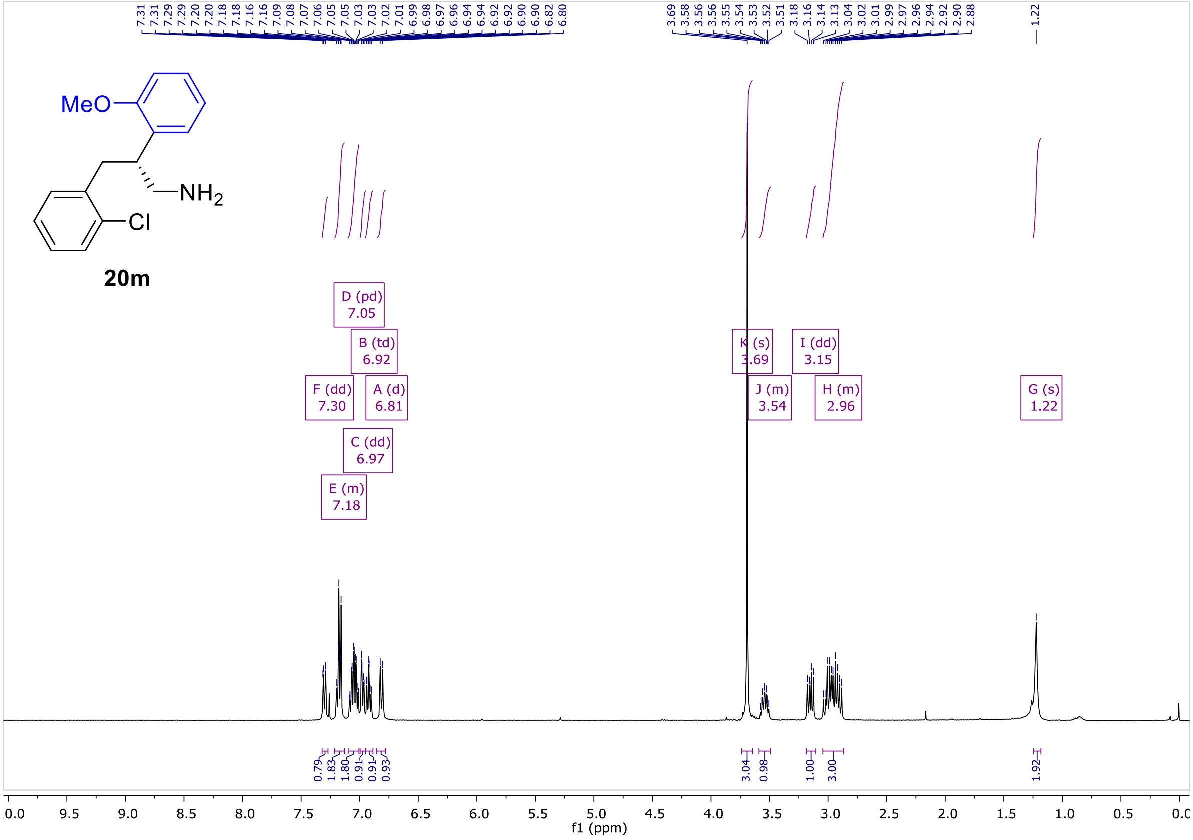

^{13}C-NMR (101 MHz, $CDCl_3$):

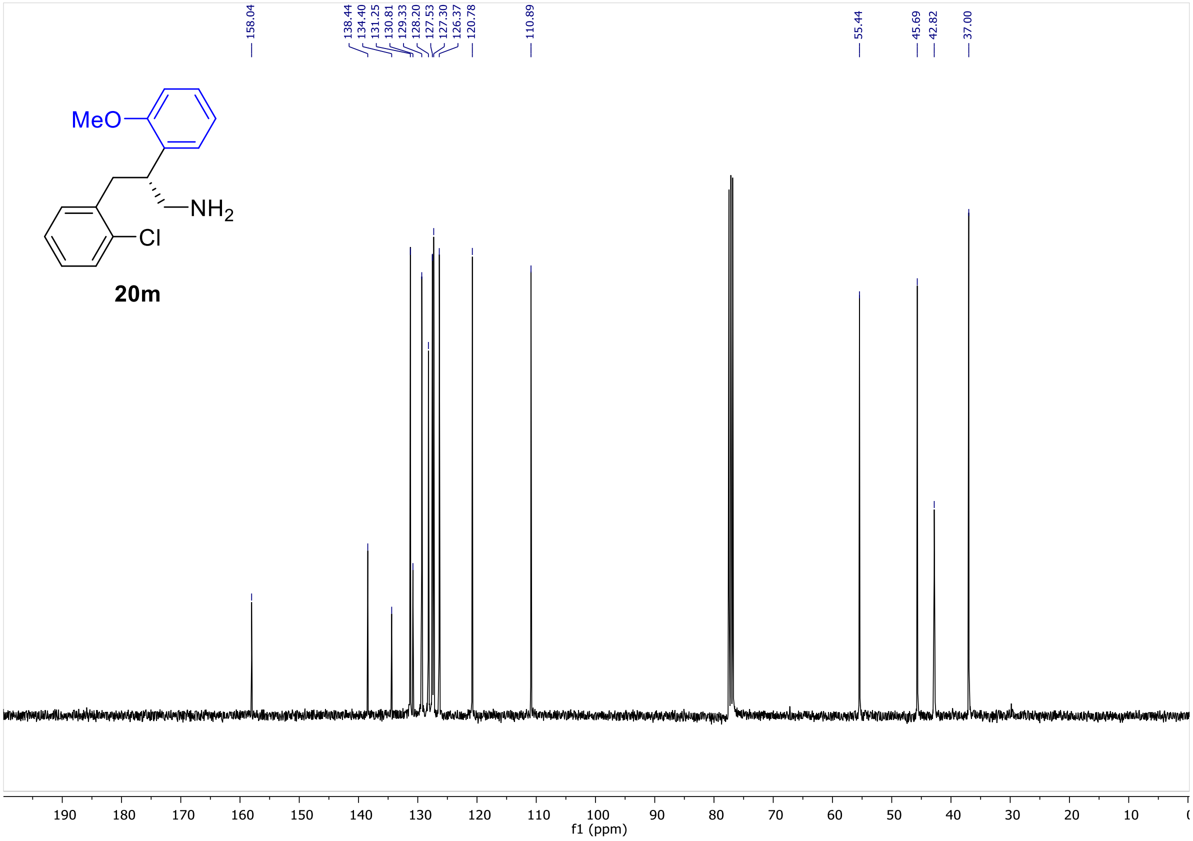

^{1}H-NMR (400 MHz, $CDCl_3$):

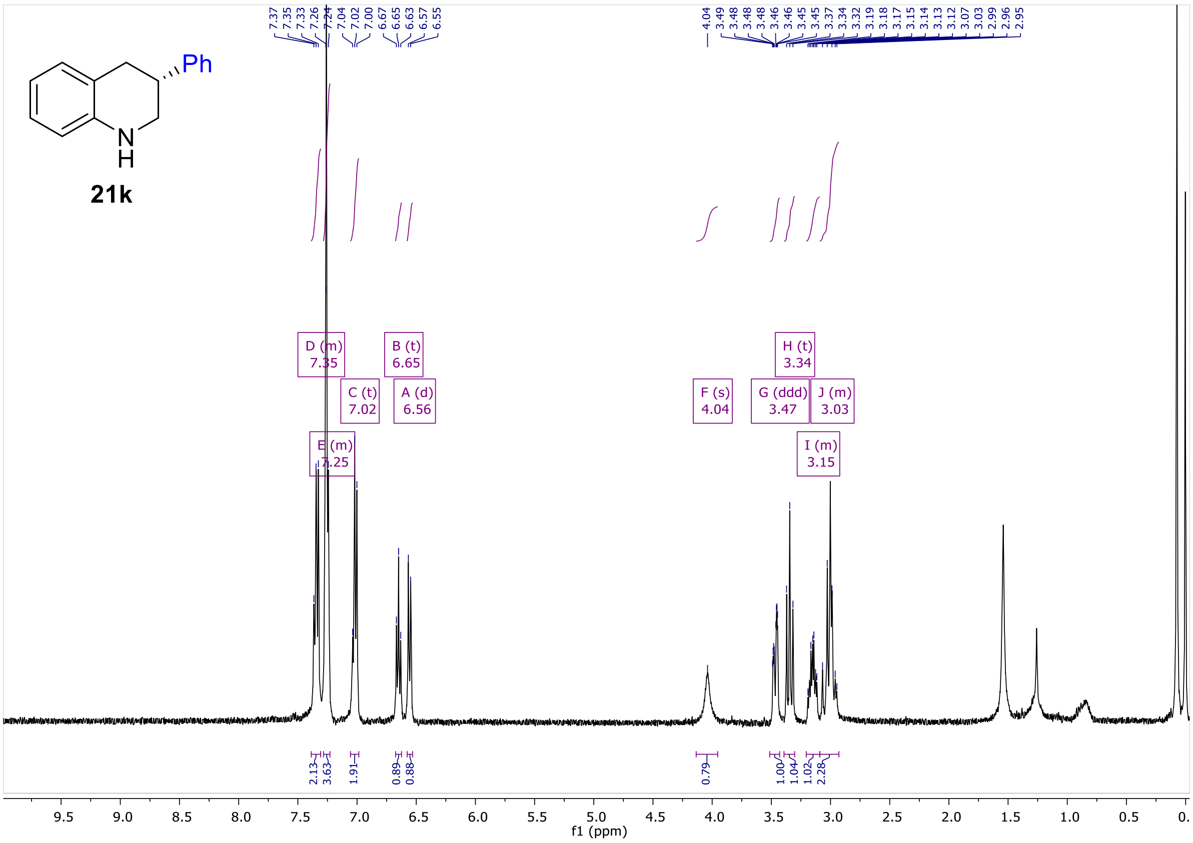

^{13}C-NMR (101 MHz, $CDCl_3$):

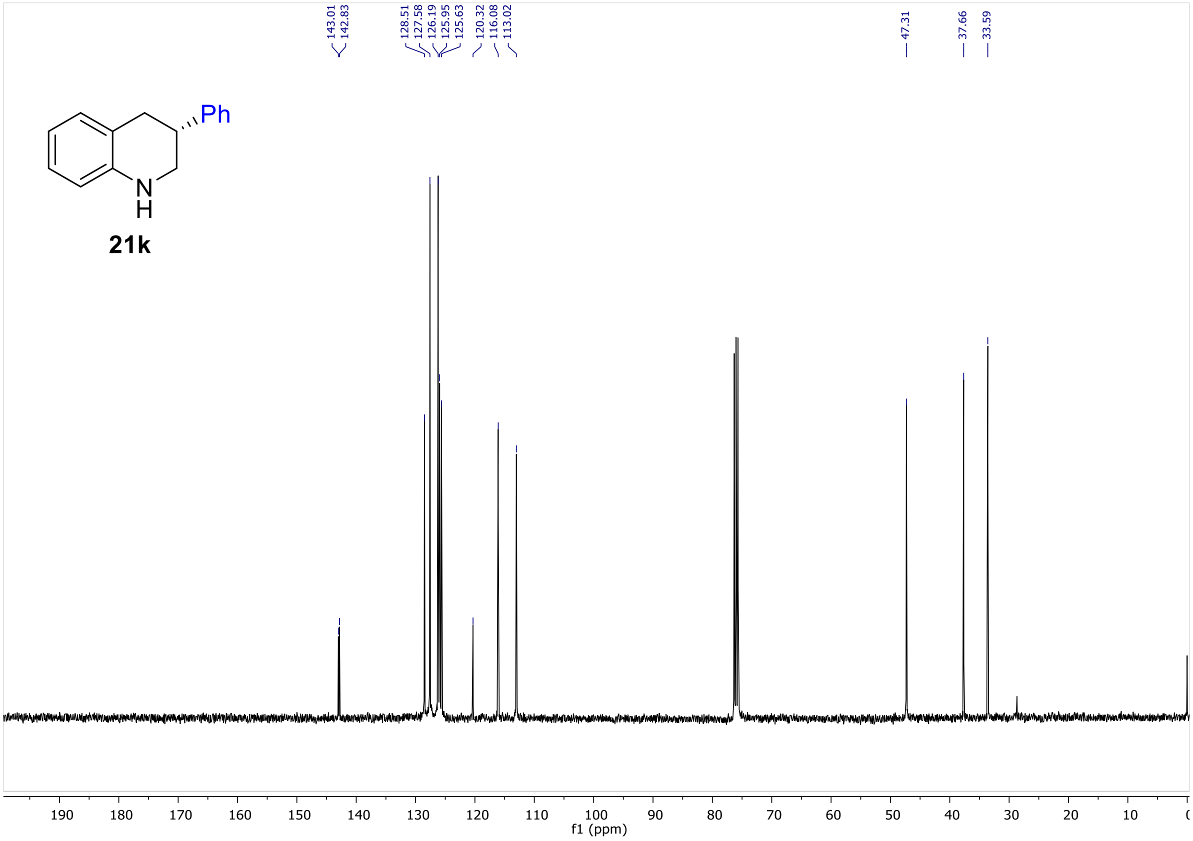

^{1}H-NMR (400 MHz, $CDCl_3$):

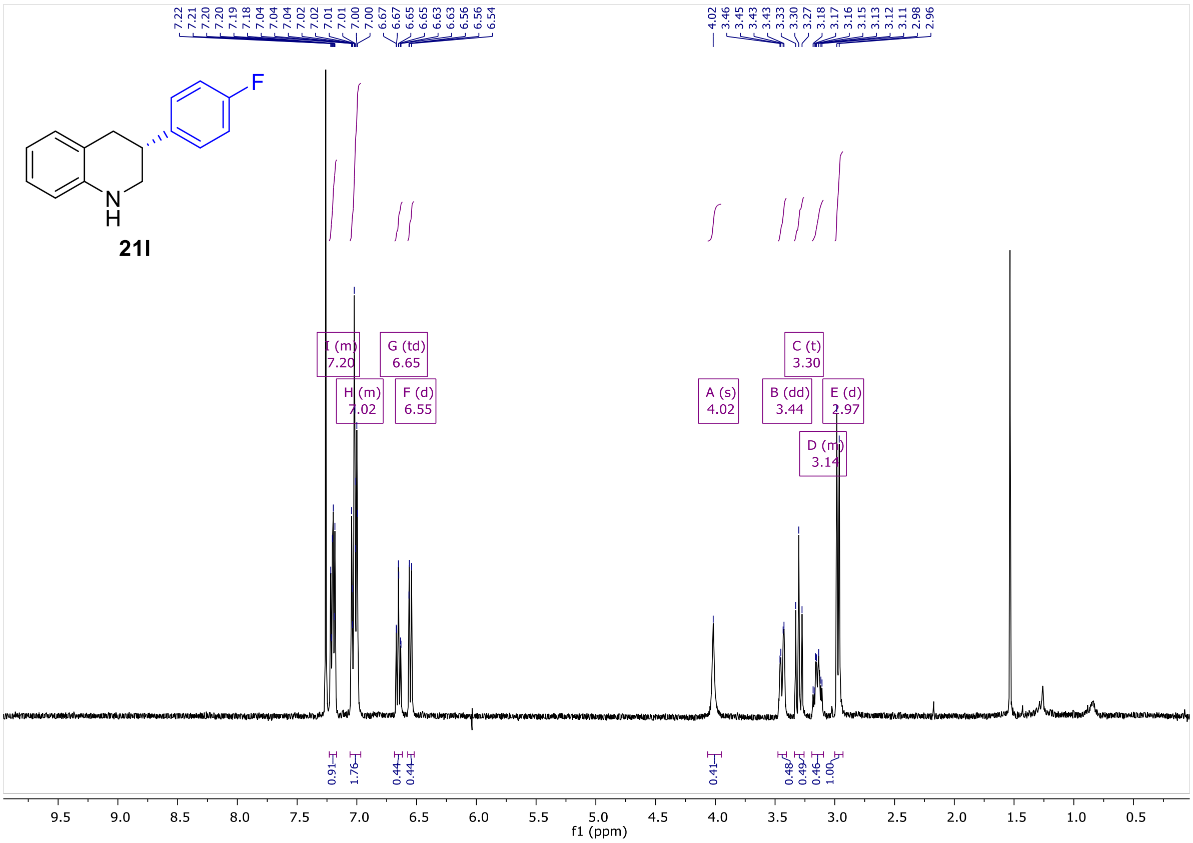

^{13}C-NMR (101 MHz, $CDCl_3$):

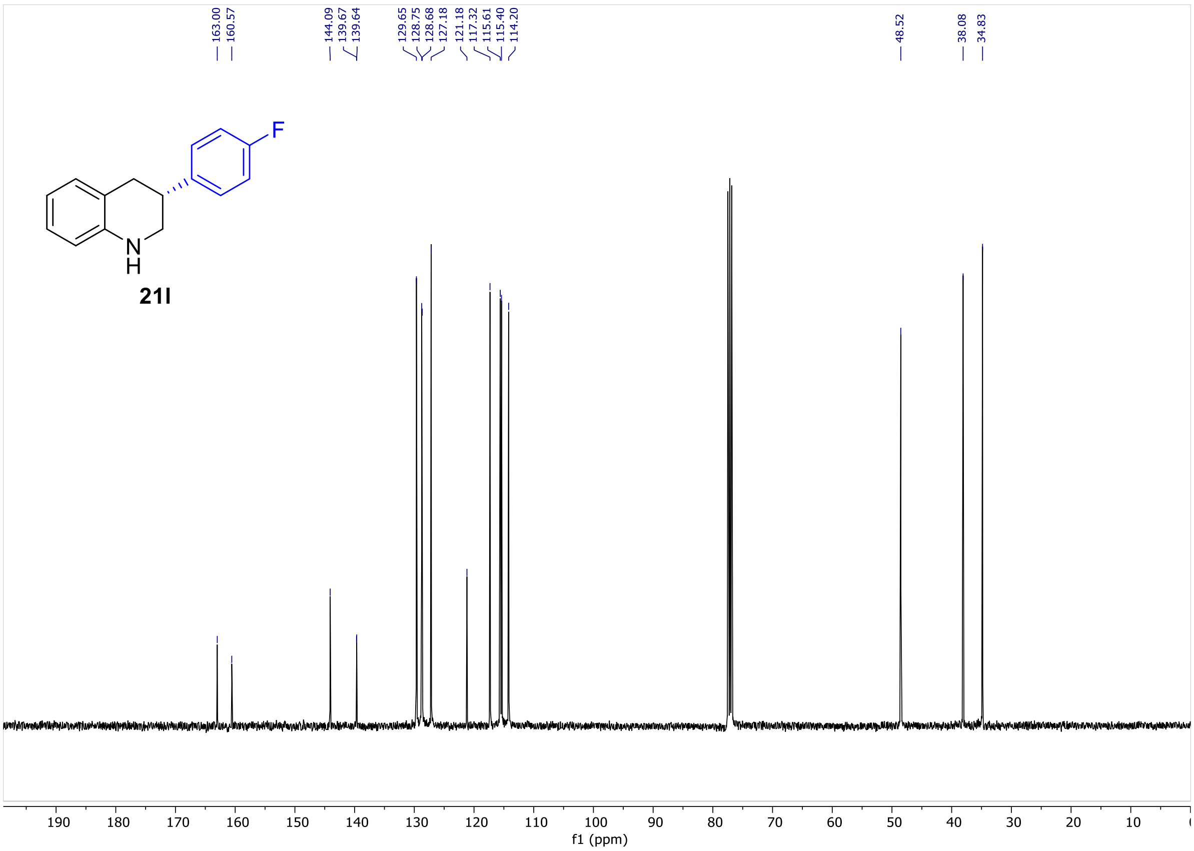

^{1}H-NMR (400 MHz, $CDCl_3$):

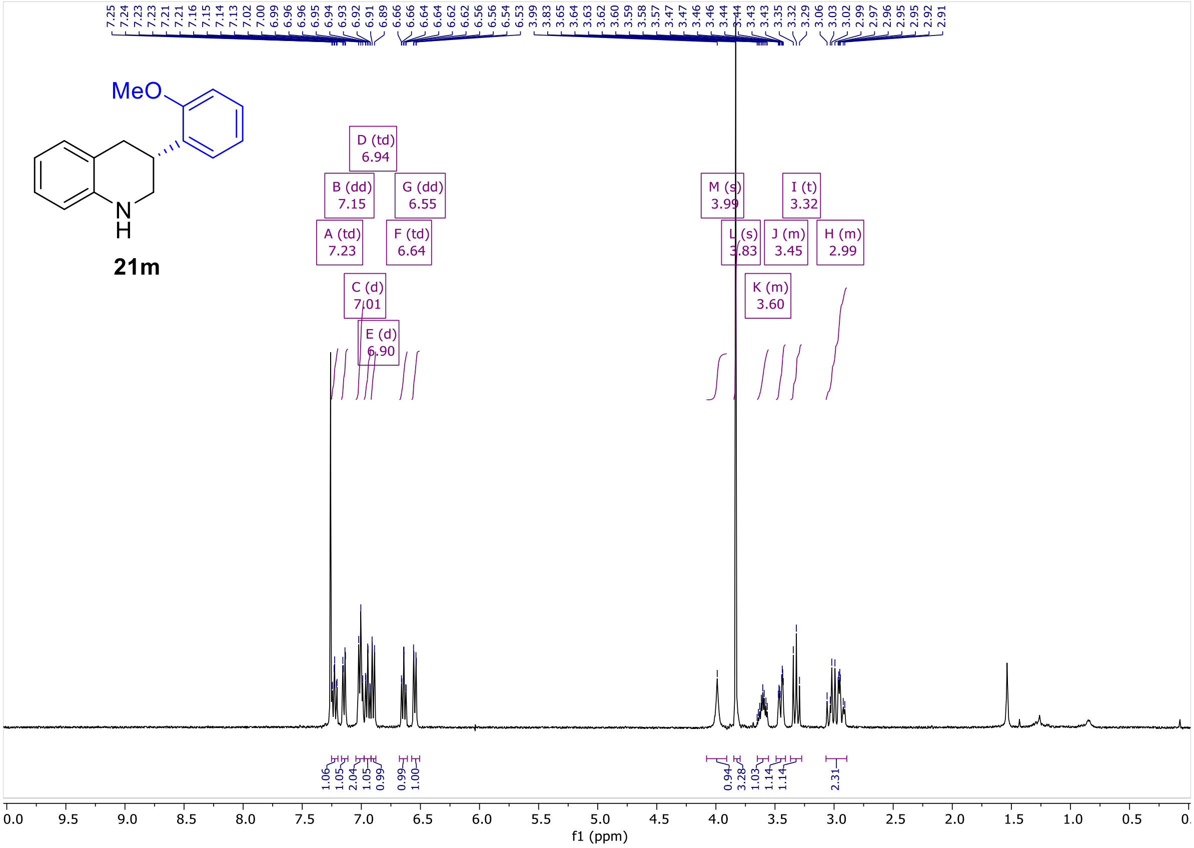

^{13}C-NMR (101 MHz, $CDCl_3$):

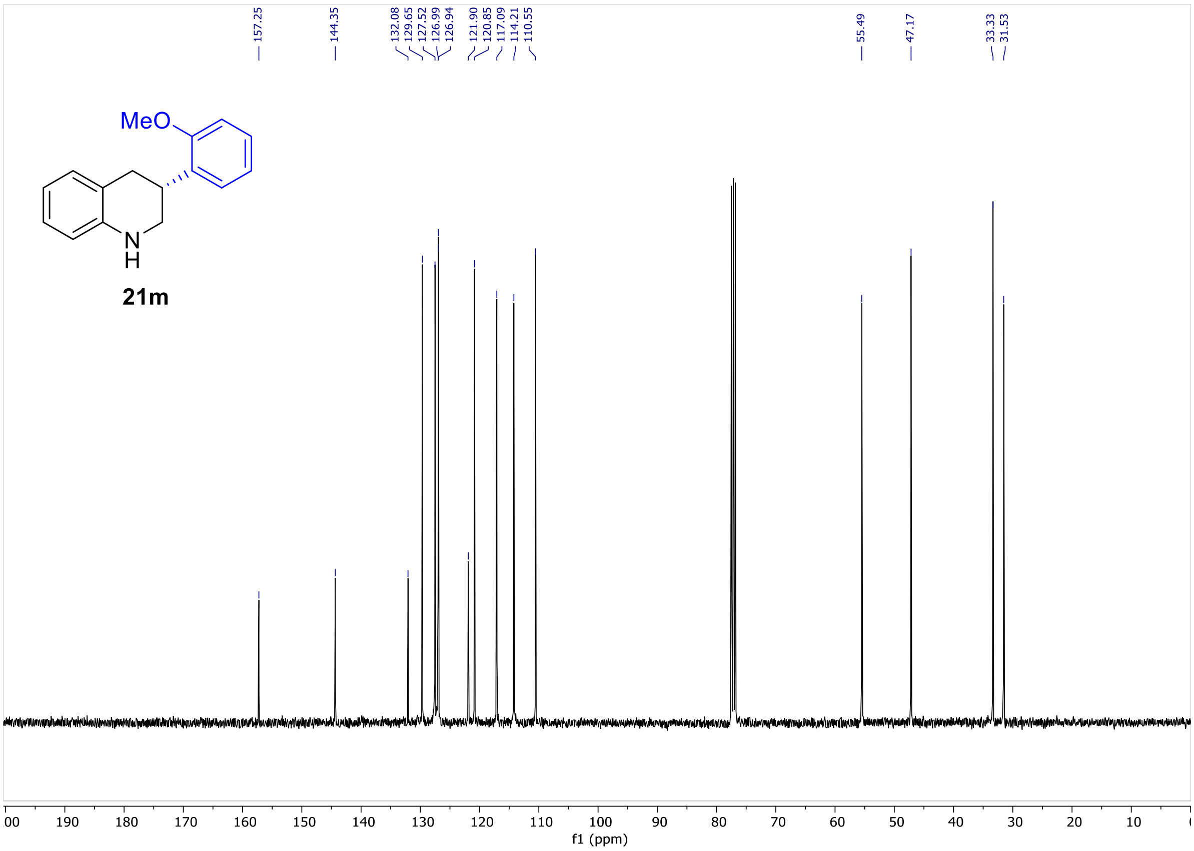

^{1}H-NMR (400 MHz, $CDCl_3$):

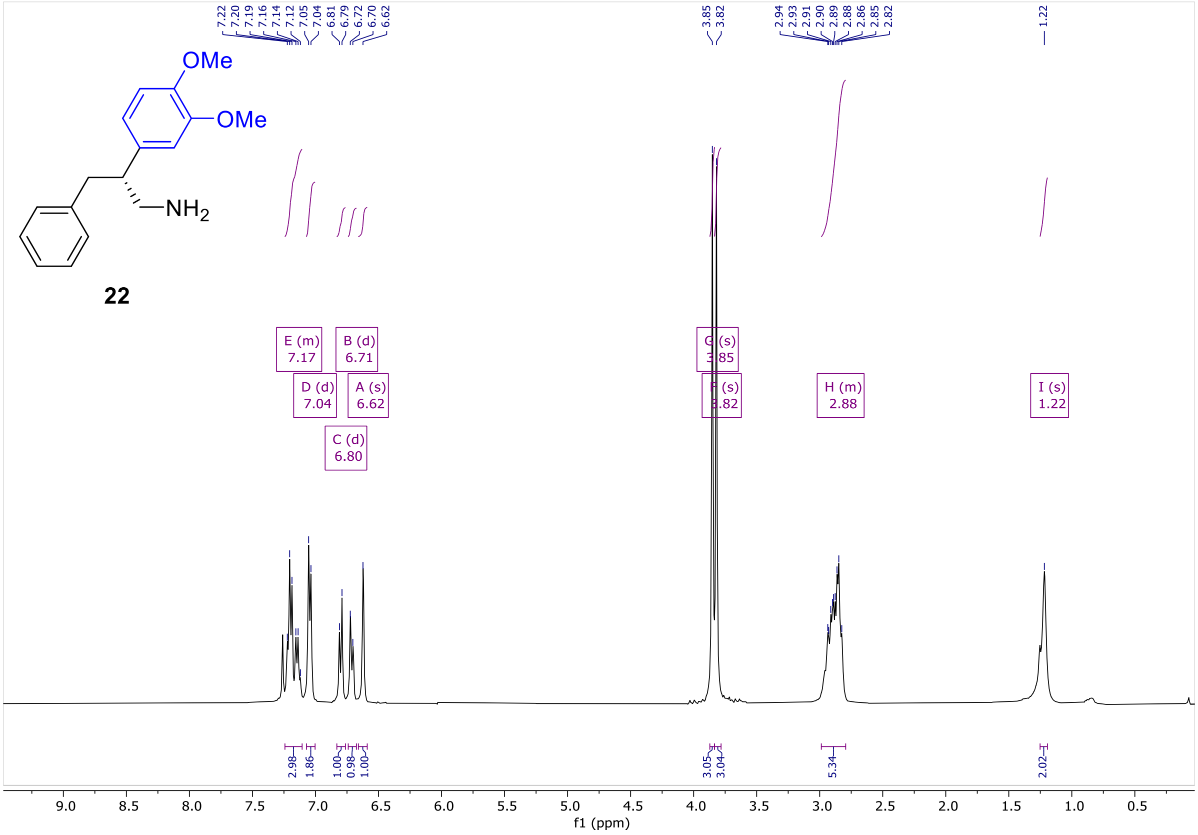

^{13}C-NMR (101 MHz, $CDCl_3$):

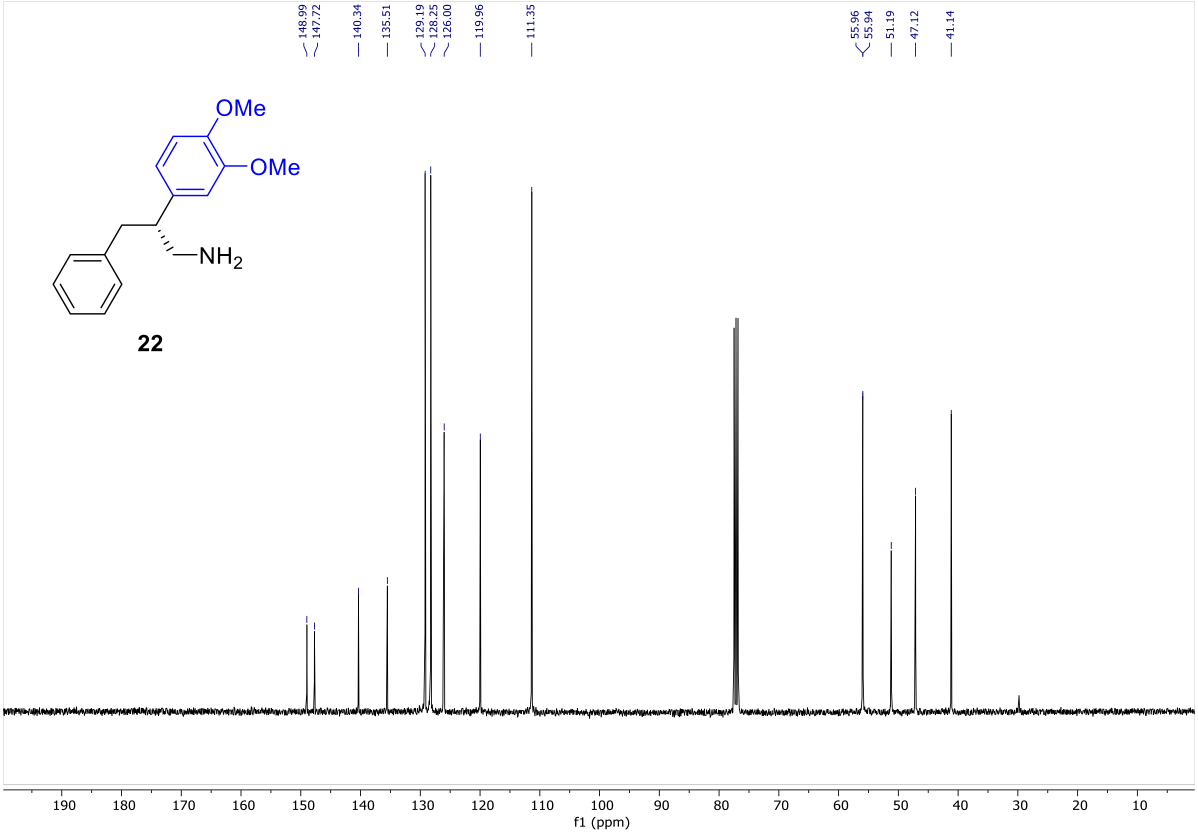

^{1}H-NMR (400 MHz, $CDCl_3$):

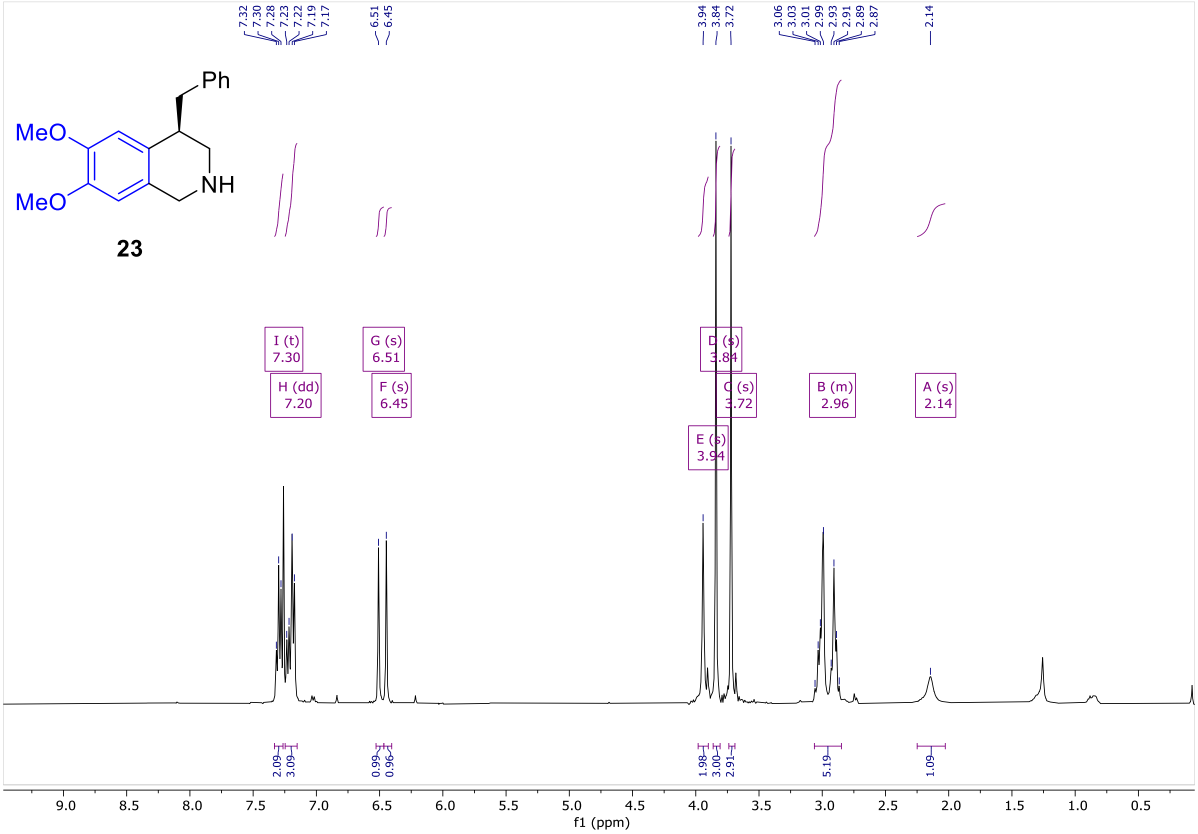

^{13}C-NMR (101 MHz, $CDCl_3$):

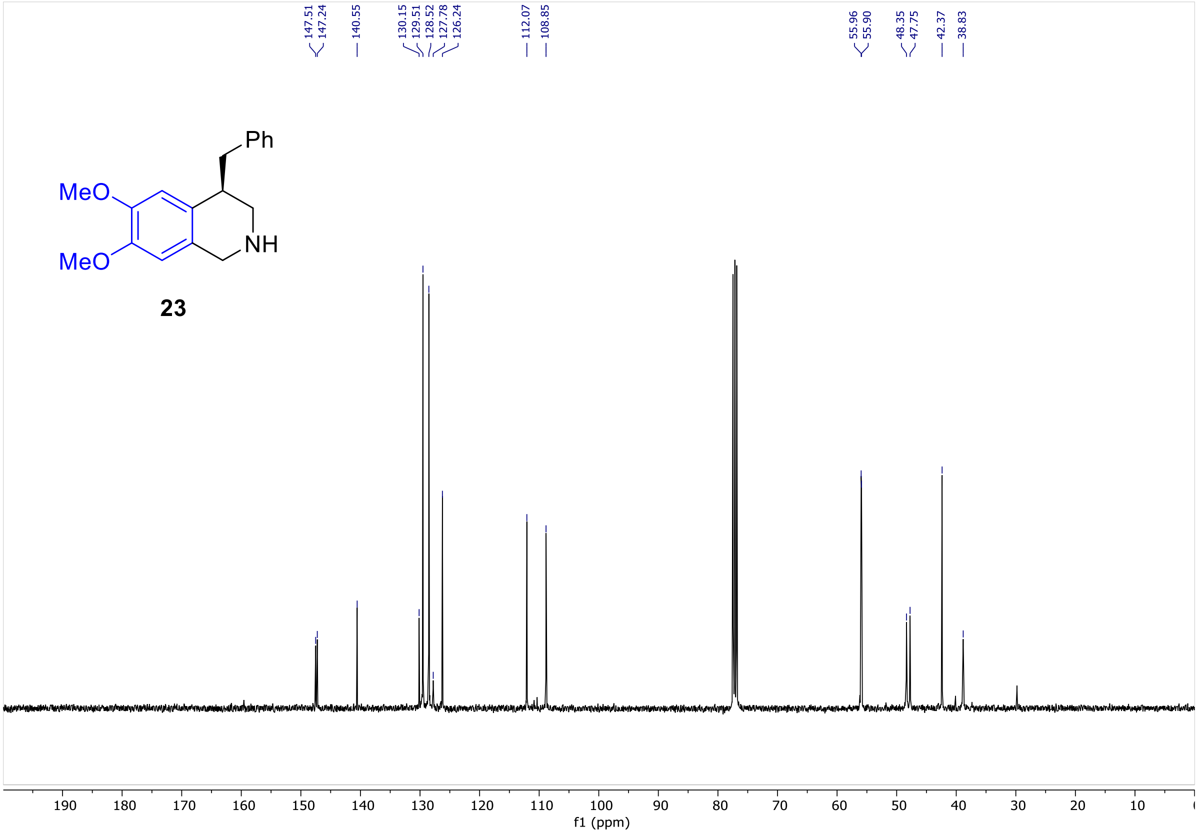

6. Chiral HPLC chromatographs

tert-Butyl (*R*)-(2-(4-methoxyphenyl)-3-phenylpropyl)carbamate, 19a

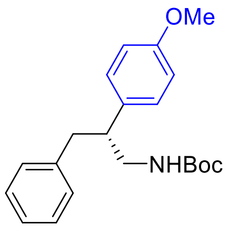

HPLC: Chiralpak IA, heptane:EtOH 98:2, 0.5 mL/min, λ = 210 nm, t_R(*R*) = 18.1 min, t_R(*S*) = 20.4 min.

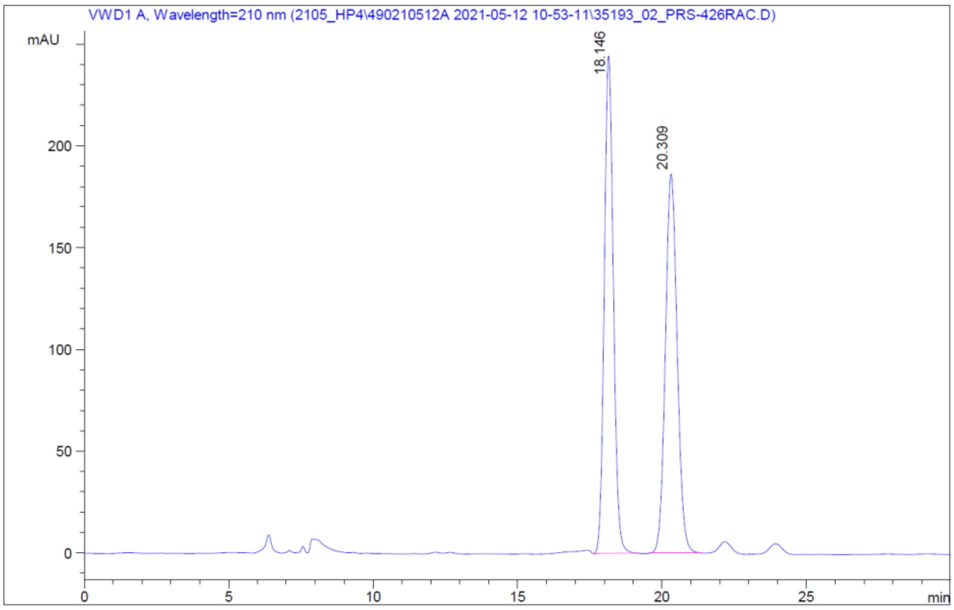

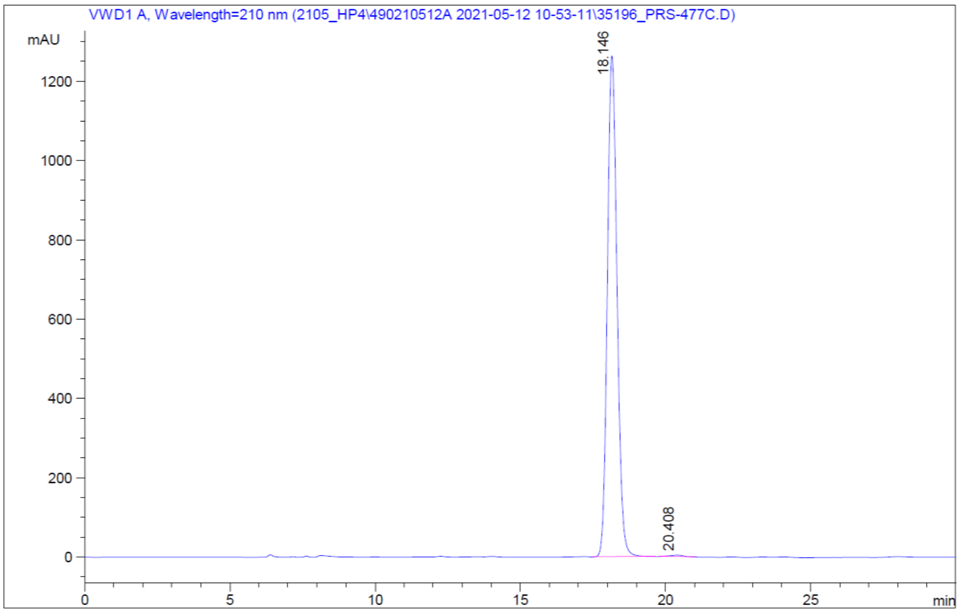

#	Meas. R	Pea	Width	Area	Height	Area %
1	18.146	MM	0.387	29343.912	1.264e3	99.514
2	20.408	MM	0.560	143.214	4.262	0.486

***tert*-Butyl (*R*)-(2-(4-chlorophenyl)-3-phenylpropyl)carbamate, 19b**

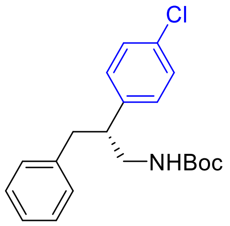

HPLC: Chiralpak IA, heptane:IPA 98:2, 0.5 mL/min, λ = 210 nm, t_R (*R*) = 30.9 min, t_R (*S*) = 36.3 min.

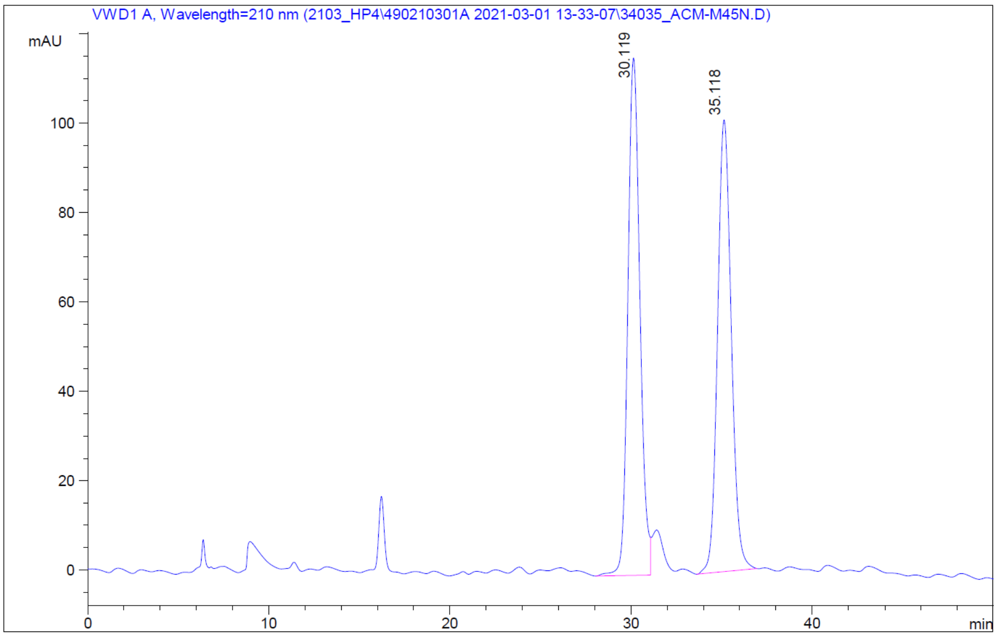

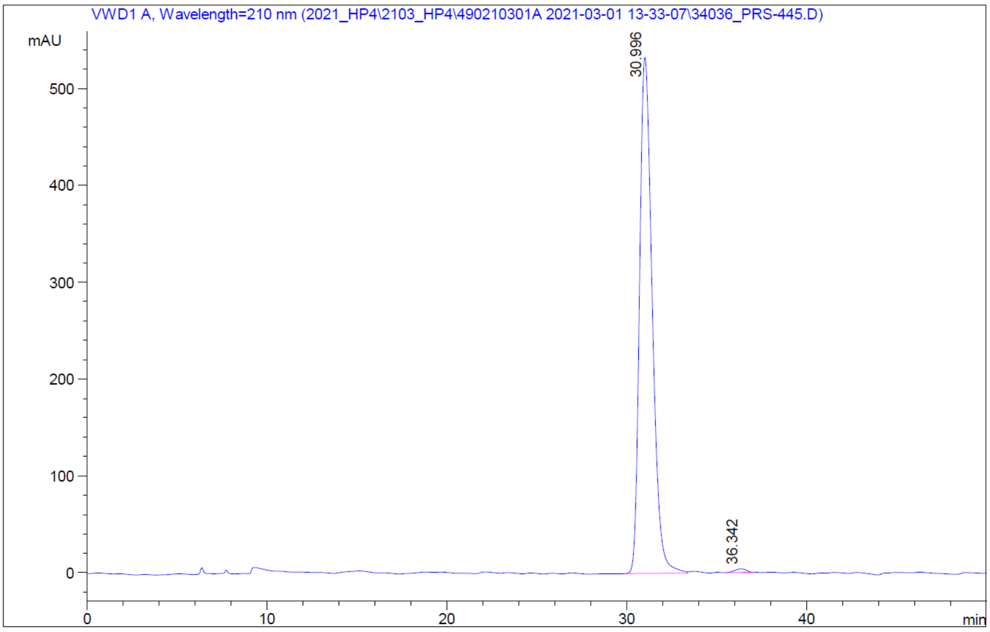

#	Meas. R	Pea	Width	Area	Height	Area %
1	30.996	BB	0.763	26085.484	533.156	99.258
2	36.342	FM	0.809	195.133	4.022	0.742

tert-Butyl (_R_)-(2-(4-fluorophenyl)-3-phenylpropyl)carbamate, 19c

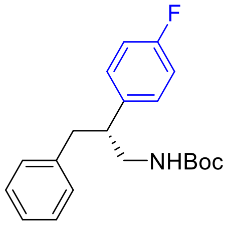

HPLC: Chiralpak IA, heptane:IPA 98:2, 0.5 mL/min, λ = 210 nm, t_R (*R*) = 22.0 min, t_R (*S*) = 26.1 min.

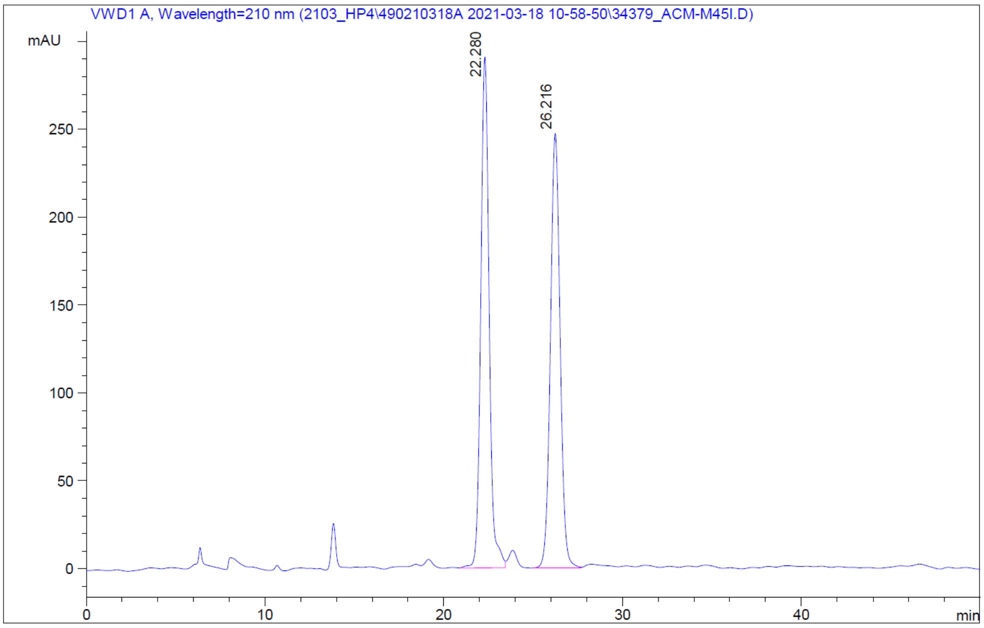

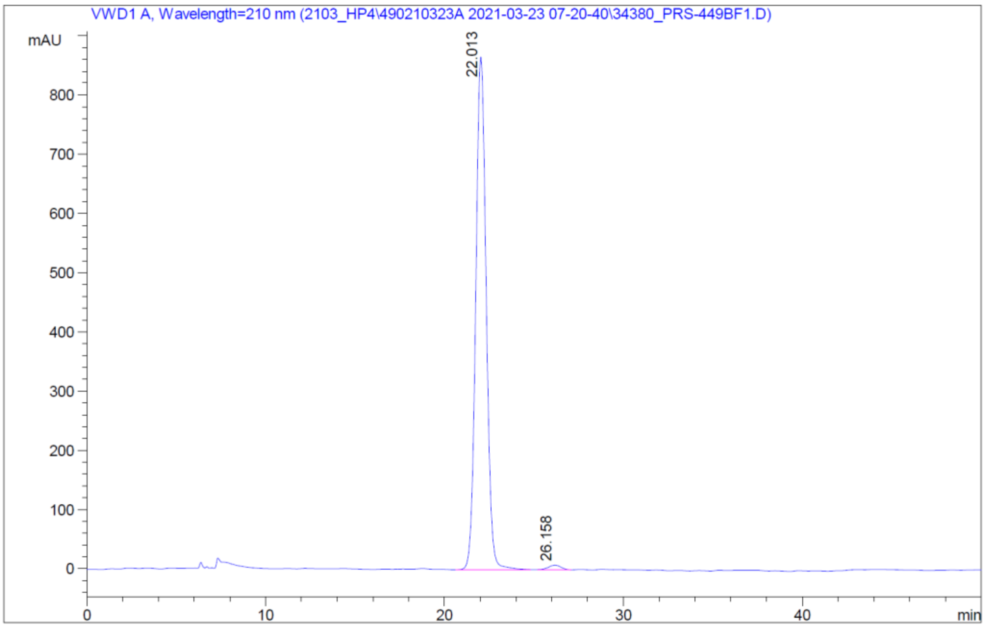

#	Meas. R	Pea	Width	Area	Height	Area %
1	22.013	BB	0.630	35073.121	865.437	98.962
2	26.158	BV	0.732	367.762	7.692	1.038

***tert*-Butyl (*R*)-(2-(4-benzoylphenyl)-3-phenylpropyl)carbamate, 19d**

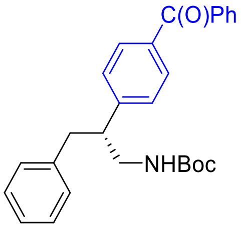

HPLC: Chiralpak IA, heptane:EtOH 9:1, 0.5 mL/min, λ = 210 nm, t_R (*R*) = 18.2 min, t_R (*S*) = 21.0 min.

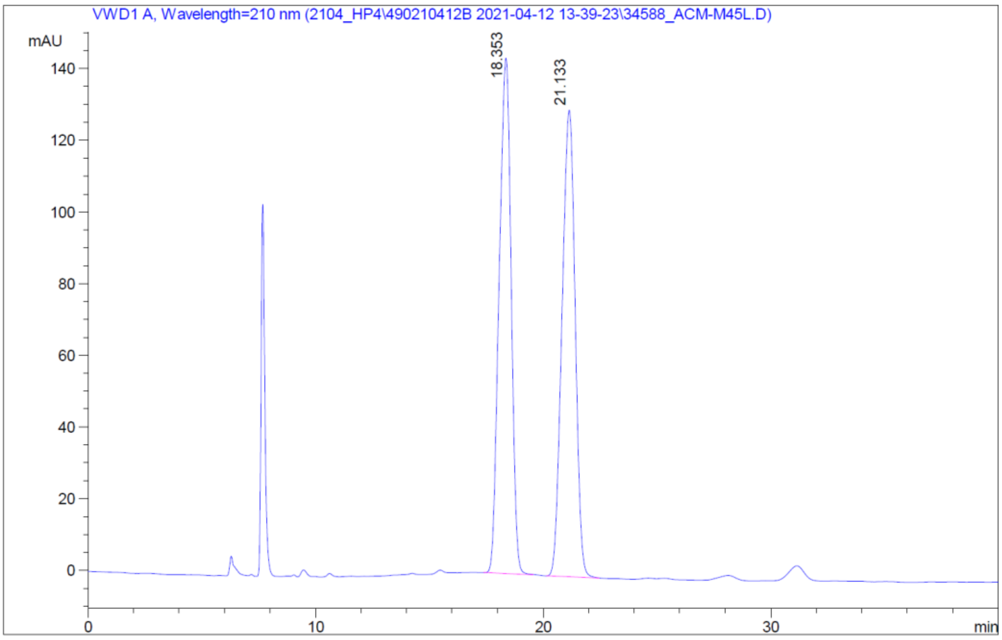

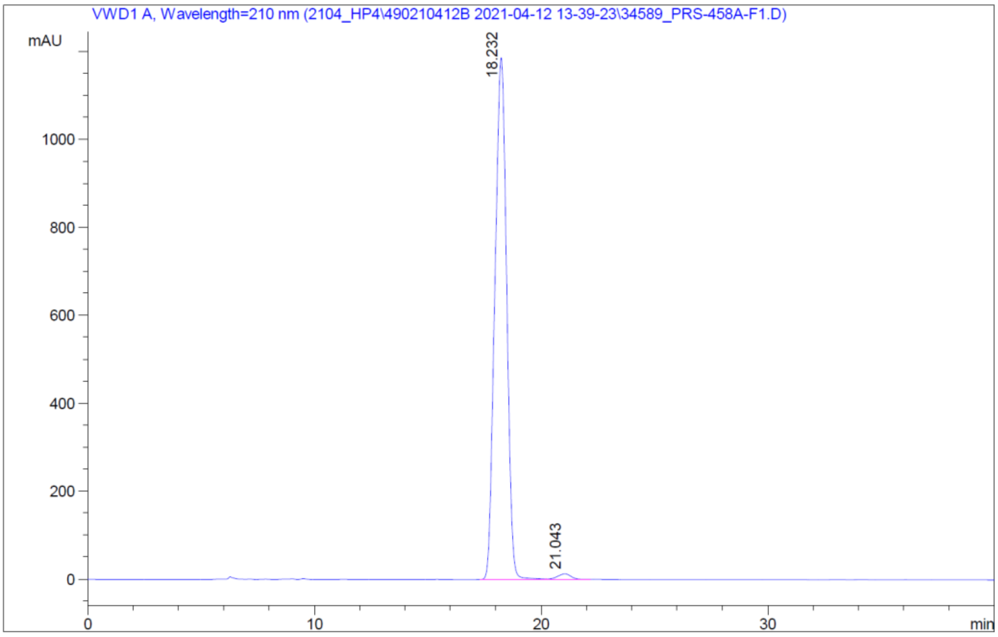

#	Meas. R	Pea	Width	Area	Height	Area %
1	18.232	BB	0.542	42573.789	1.186e3	98.837
2	21.043	BB	0.645	500.867	12.338	1.163

Methyl (*R*)-4-(1-((*tert*-butoxycarbonyl)amino)-3-phenylpropan-2-yl)benzoate, 19e

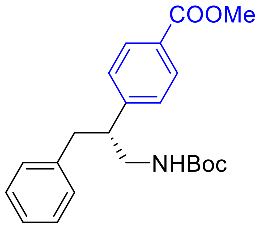

HPLC: Chiralpak IC, heptane:IPA 85:15, 0.5 mL/min, λ = 210 nm, t_R (*S*) = 24.3 min, t_R (*R*) = 27.1 min.

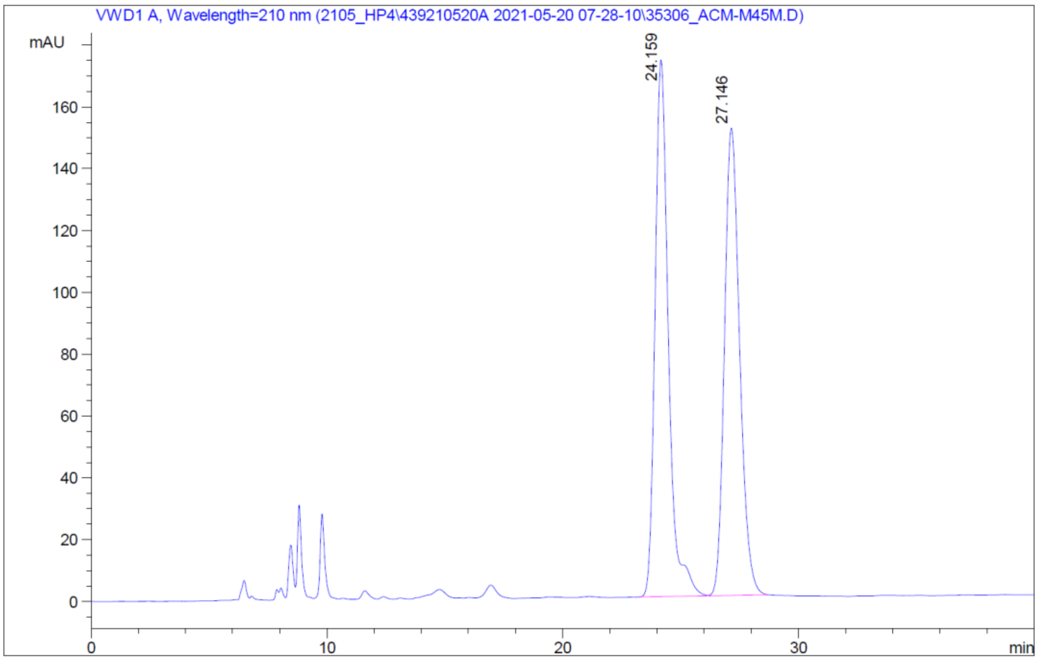

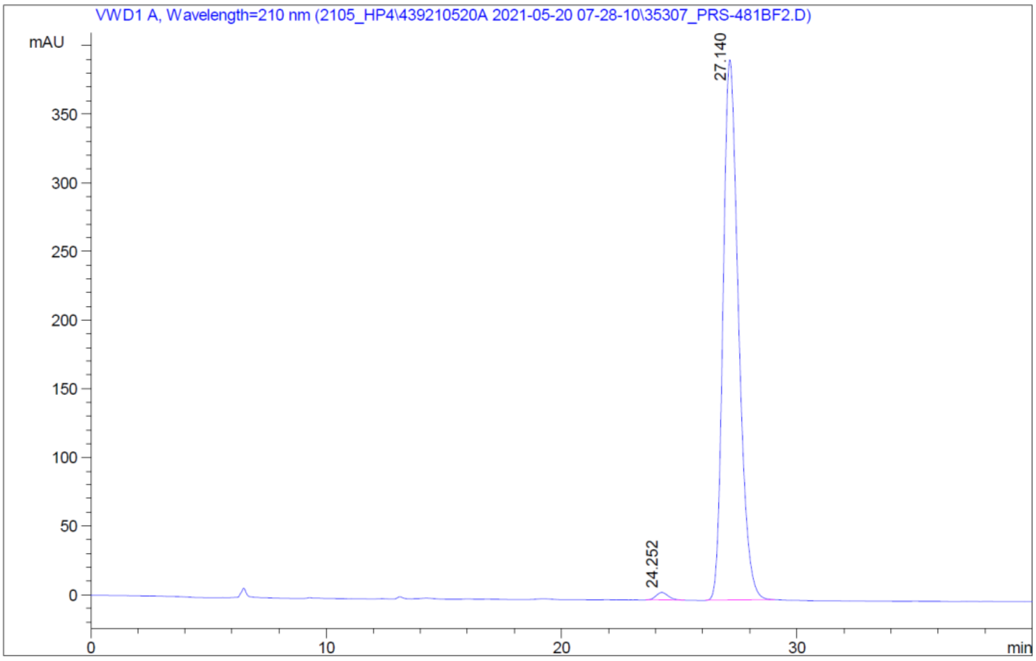

#	Meas. R	Pea	Width	Area	Height	Area %
1	24.252	BB	0.548	205.328	5.552	1.128
2	27.140	BB	0.704	18002.125	393.482	98.872

tert-Butyl (R)-(2-(2-methoxyphenyl)-3-phenylpropyl)carbamate, 19f

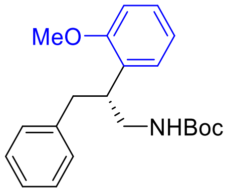

HPLC: Chiralpak IA, heptane:IPA 97:3, 0.5 mL/min, λ = 210 nm, t_R (*S*) = 24.3 min, t_R (*R*) = 27.1 min.

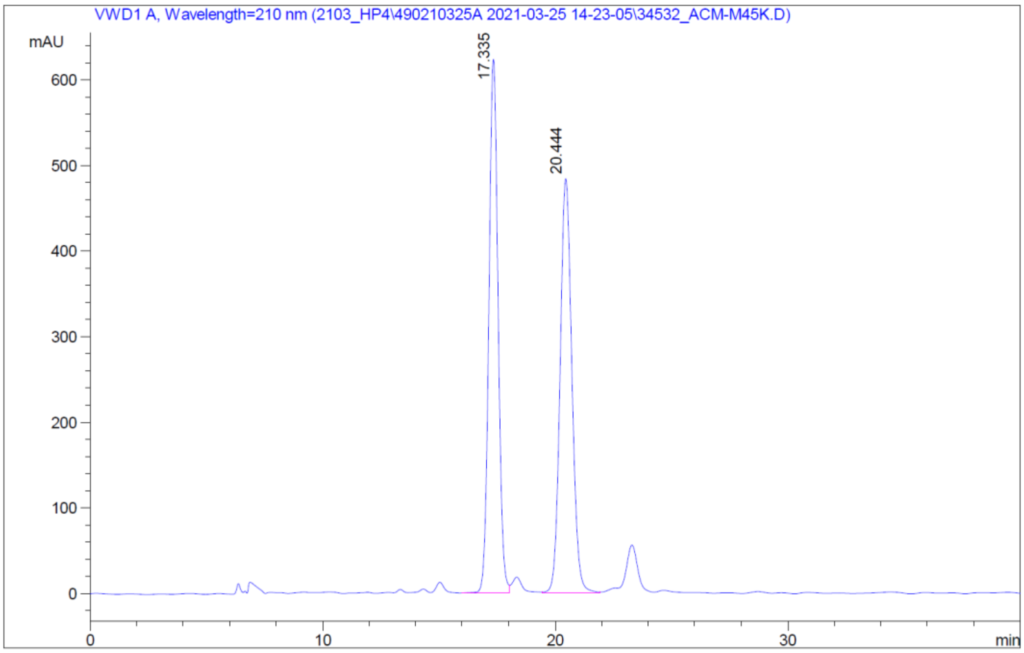

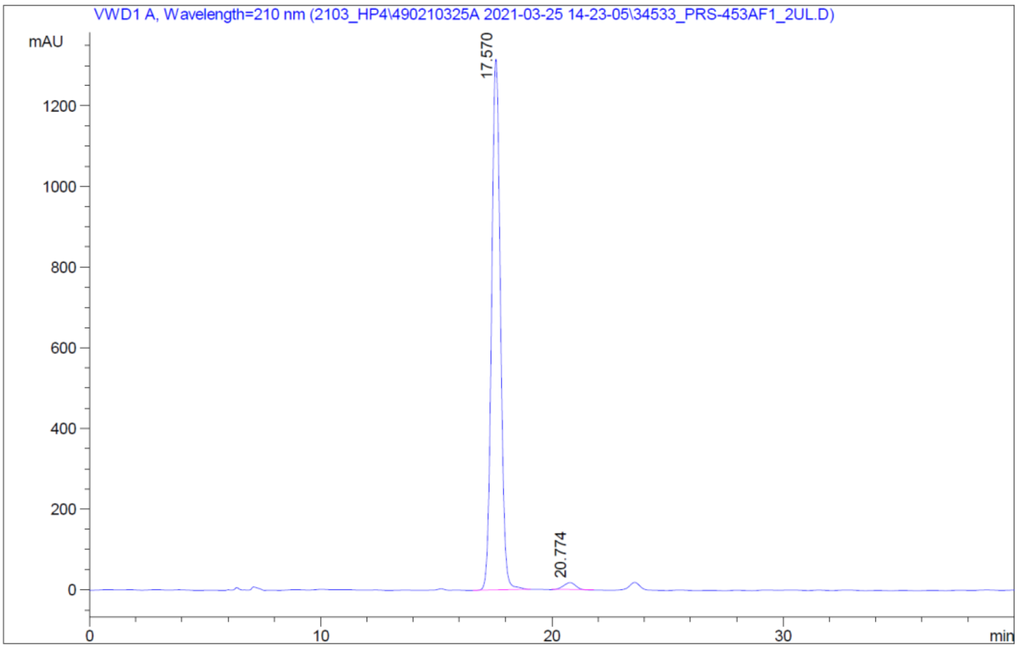

#	Meas. R	Pea	Width	Area	Height	Area %
1	17.570	VB	0.415	34974.305	1.317e3	98.147
2	20.774	BB	0.581	660.134	17.754	1.853

tert-Butyl (_R_)-(2-(2-fluorophenyl)-3-phenylpropyl)carbamate, 19g

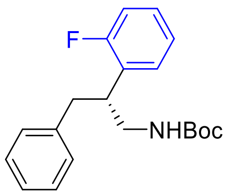

HPLC: Chiralpak IC, heptane:IPA 95:5, 0.5 mL/min, λ = 210 nm, t_R (*R*) = 16.9 min, t_R (*S*) = 19.8 min.

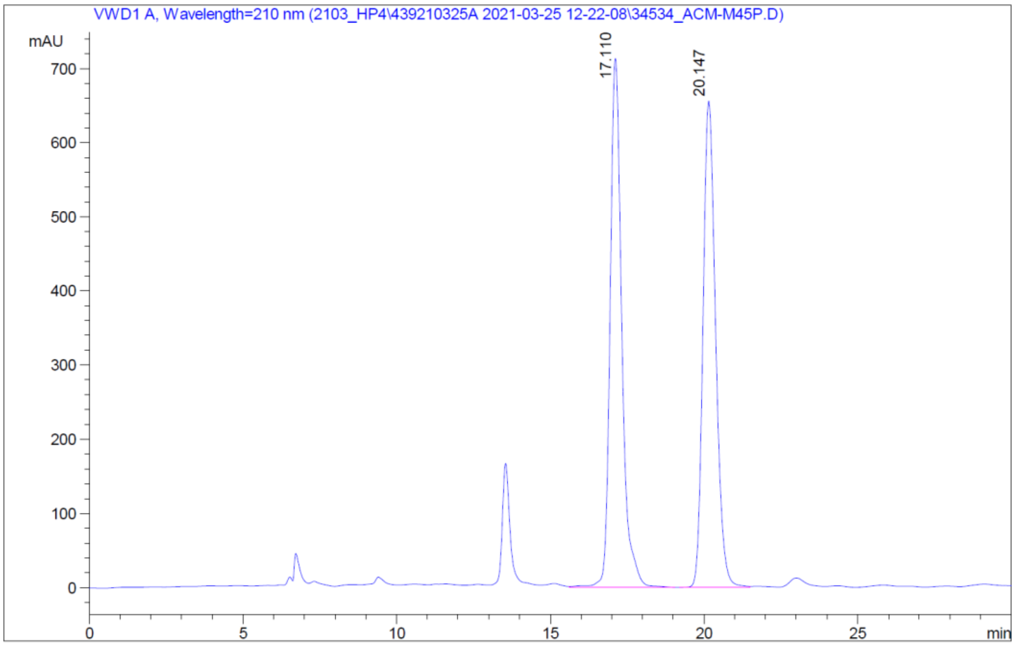

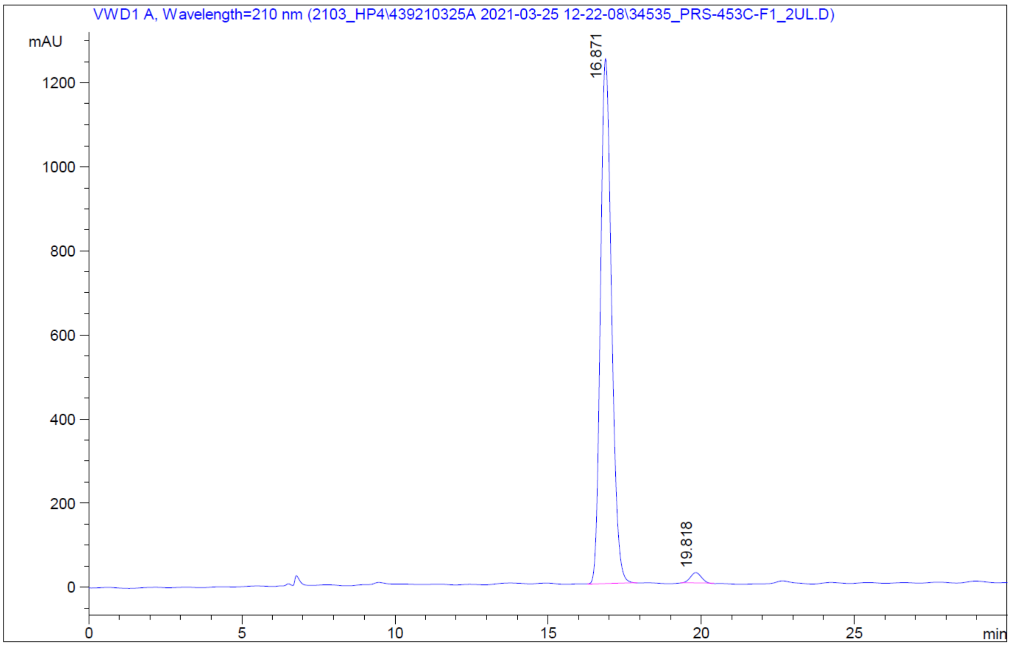

#	Meas. R	Pea	Width	Area	Height	Area %
1	16.871	MM	0.419	31432.643	1.251e3	97.966
2	19.818	MM	0.429	652.561	25.381	2.034

***tert*-Butyl (*R*)-(3-phenyl-2-(*o*-tolyl)propyl)carbamate, 19h**

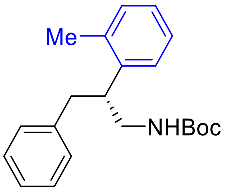

HPLC: Chiralpak IC, heptane:IPA 95:5, 0.5 mL/min, λ = 210 nm, t_R (*R*) = 14.0 min, t_R (*S*) = 18.7 min.

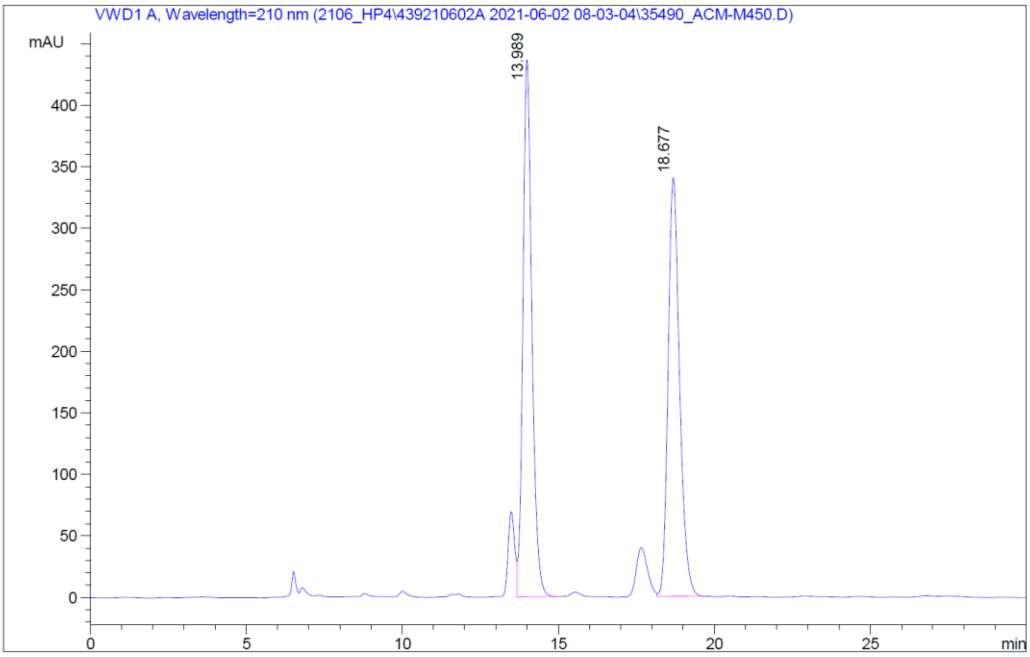

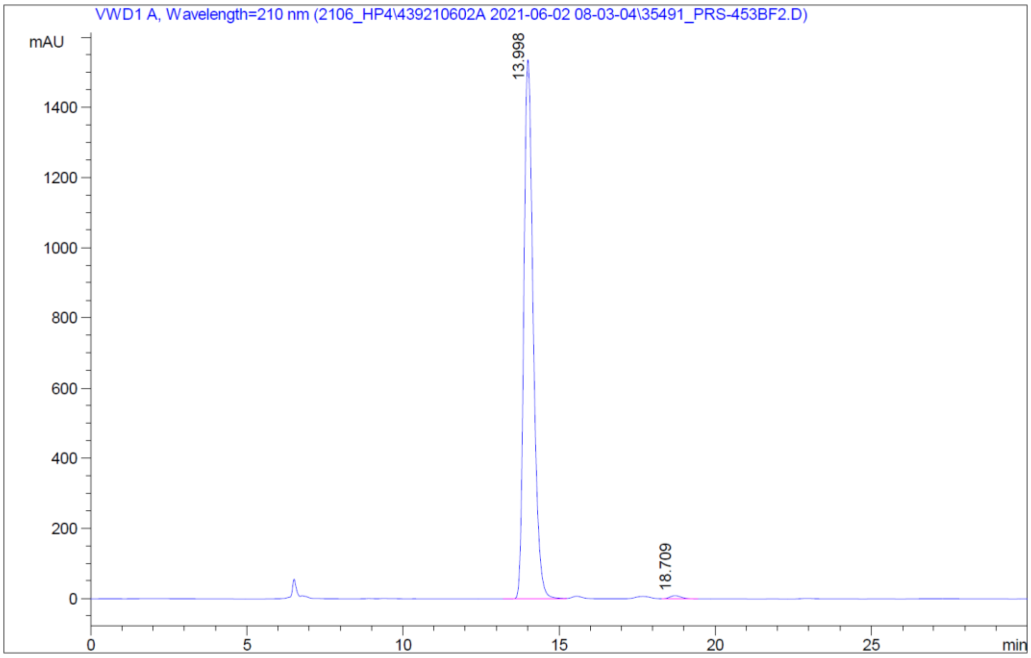

#	Meas. R	Pea	Width	Area	Height	Area %
1	13.998	VB	0.321	32182.211	1.538e3	99.274
2	18.709	VB	0.385	235.209	9.298	0.726

tert-Butyl (R)-(2-(3,5-dimethylphenyl)-3-phenylpropyl)carbamate, 19i

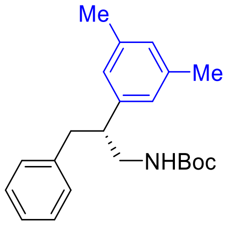

HPLC: Chiralpak IA, heptane:IPA 98:2, 0.5 mL/min, λ = 210 nm, t_R (*R*) = 13.2 min, t_R (*S*) = 14.5 min.

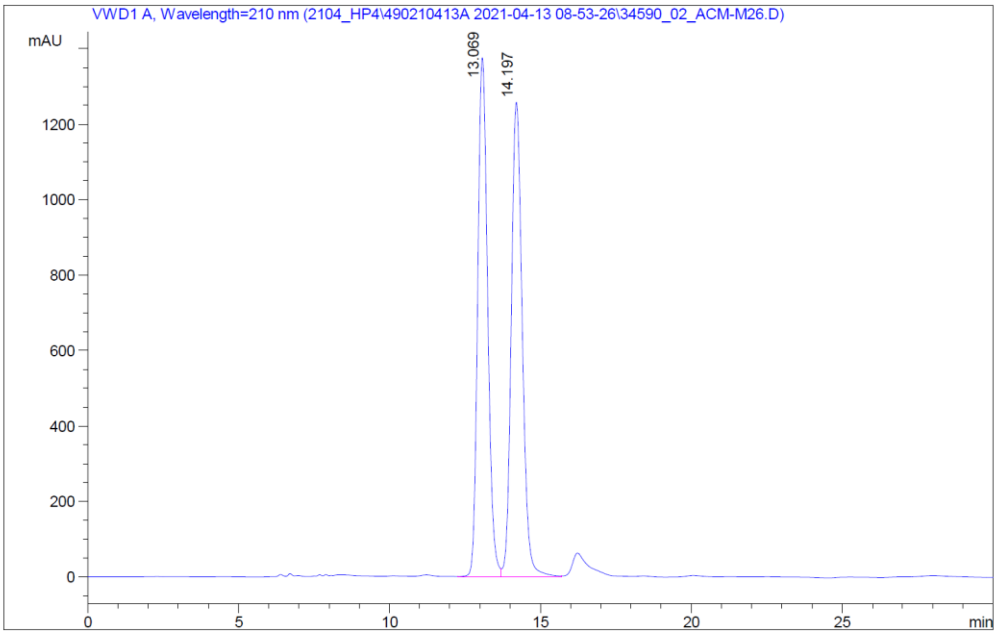

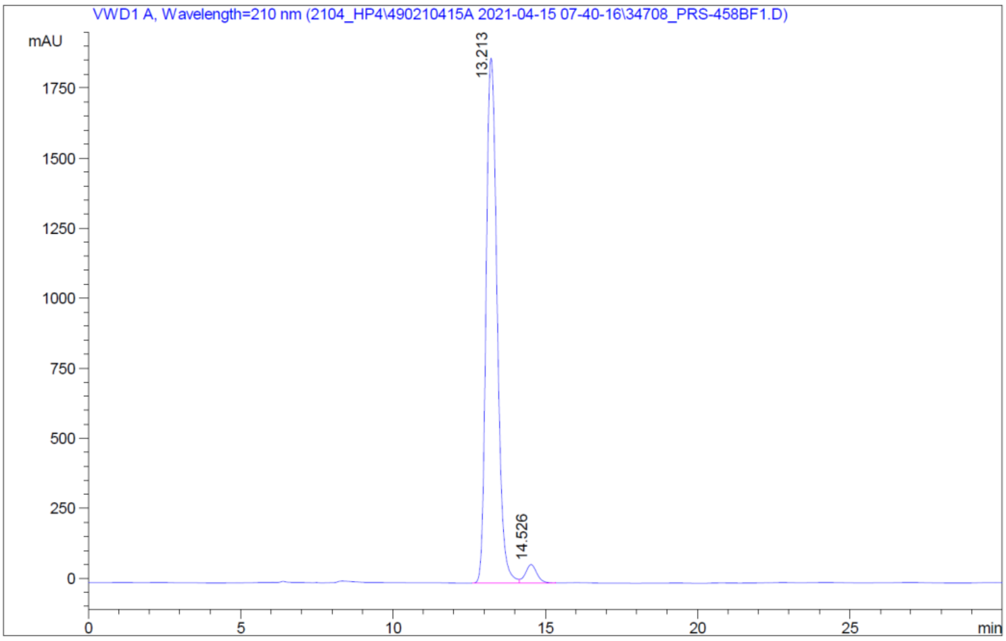

#	Meas. R	Pea	Width	Area	Height	Area %
1	13.213	MF	0.419	47086.941	1.874e3	96.457
2	14.526	FM	0.438	1729.336	65.743	3.543

tert-Butyl (R)-(2-(3,5-dimethoxyphenyl)-3-phenylpropyl)carbamate, 19j

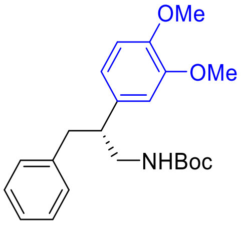

HPLC: Chiralpak IC, heptane:EtOH 8:2, 0.5 mL/min, λ = 210 nm, tR (R) = 13.9 min, tR (S) = 17.4 min.

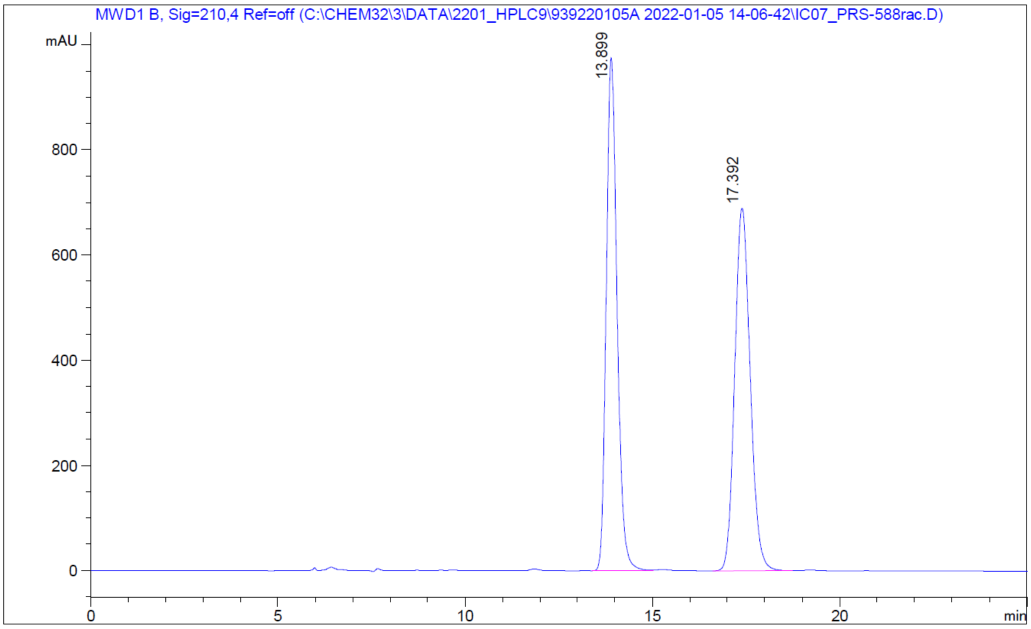

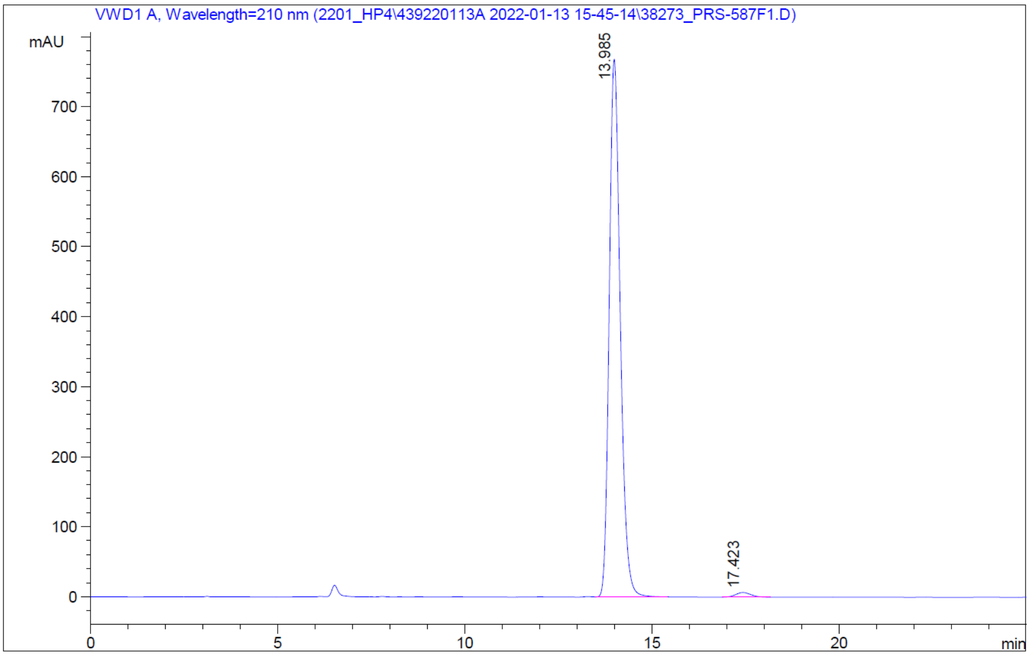

#	Meas. R	Pea	Width	Area	Height	Area %
1	13.985	VB	0.315	15737.816	768.176	98.796
2	17.423	BB	0.441	191.741	6.690	1.204

tert-Butyl (_R_)-(3-(2-chlorophenyl)-2-phenylpropyl)carbamate, 19k

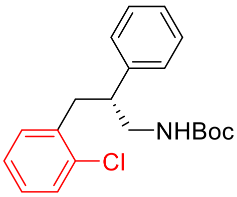

HPLC: Chiralpak IA, heptane:EtOH 98:2, 0.5 mL/min, λ = 210 nm, t_R (_R_) = 14.0 min, t_R (_S_) = 16.8 min.

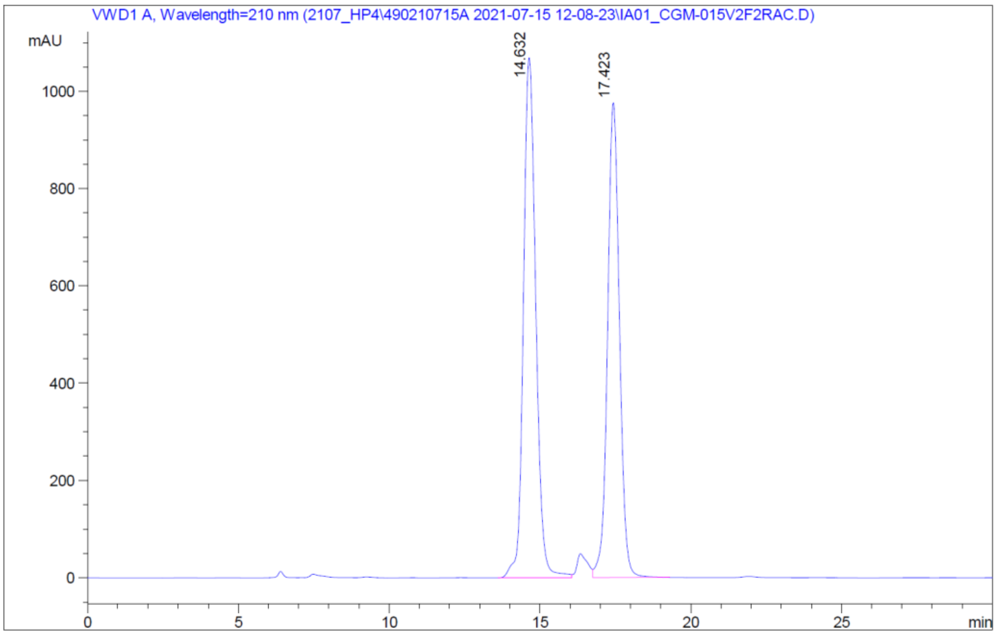

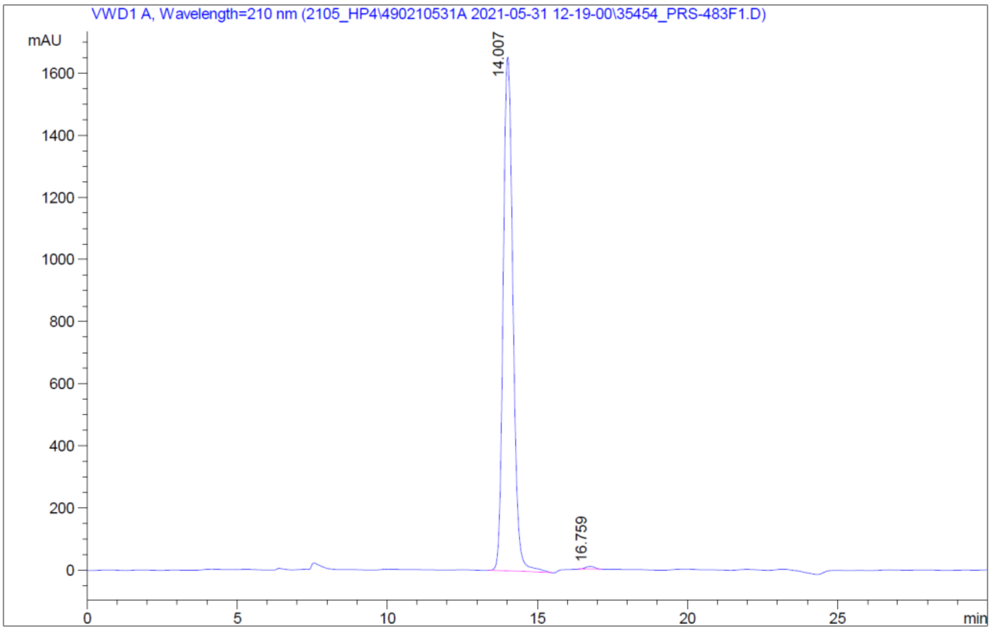

#	Meas. R	Pea	Width	Area	Height	Area %
1	14.007	MM	0.378	37533.992	1.655e3	99.431
2	16.759	MM	0.371	214.712	9.640	0.569

tert-Butyl (R)-(3-(2-chlorophenyl)-2-phenylpropyl)carbamate, 19l

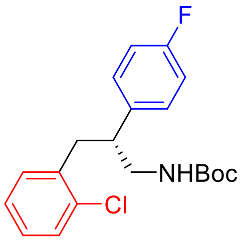

HPLC: Chiralpak IA, heptane:IPA 98:2, 0.5 mL/min, λ = 210 nm, t_R (*R*) = 19.0 min, t_R (*S*) = 21.4 min.

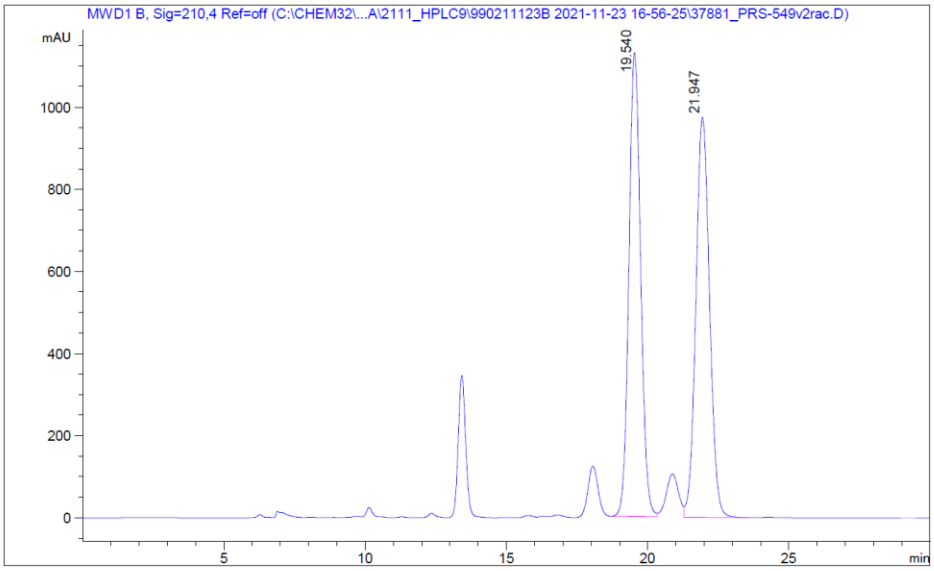

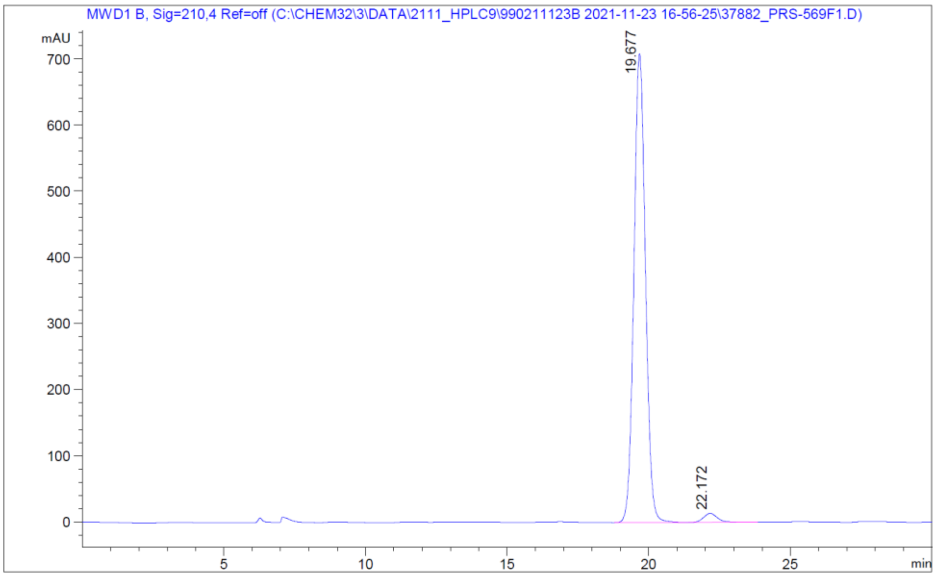

#	Meas. R	Pea	Width	Area	Height	Area %
1	19.677	BB	0.438	20069.332	708.767	97.716
2	22.172	BB	0.516	469.184	13.475	2.284

***tert*-Butyl (*R*)-(3-(2-chlorophenyl)-2-(2-methoxyphenyl)propyl)carbamate, 19m**

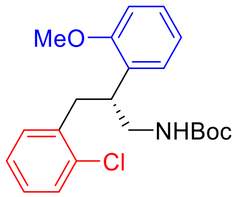

HPLC: Chiralpak IA, heptane:IPA 95:5, 0.5 mL/min, λ = 210 nm, t_R (*R*) = 12.8 min, t_R (*S*) = 14.9 min.

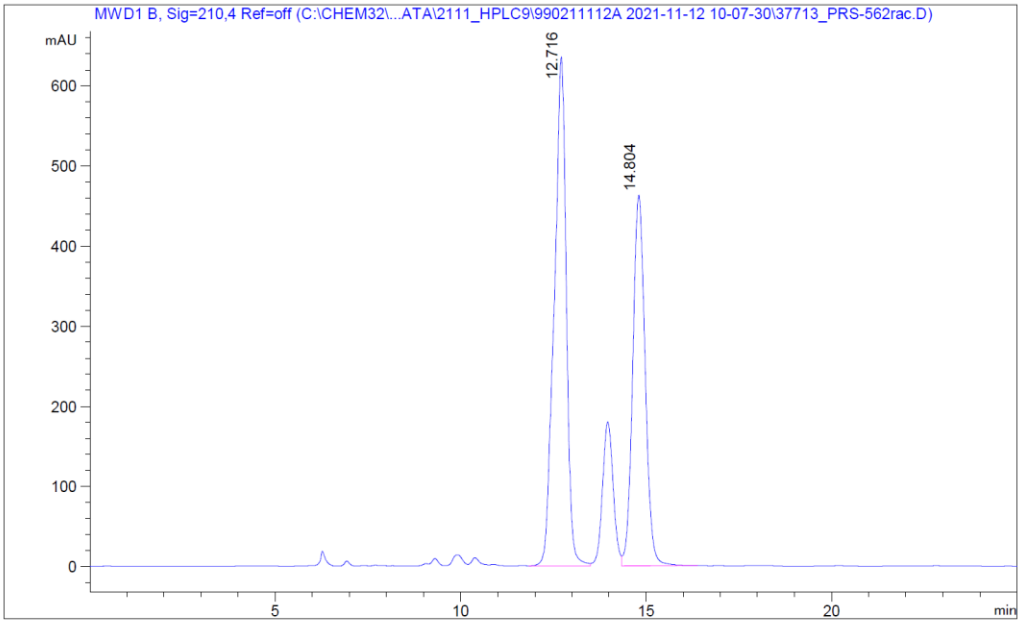

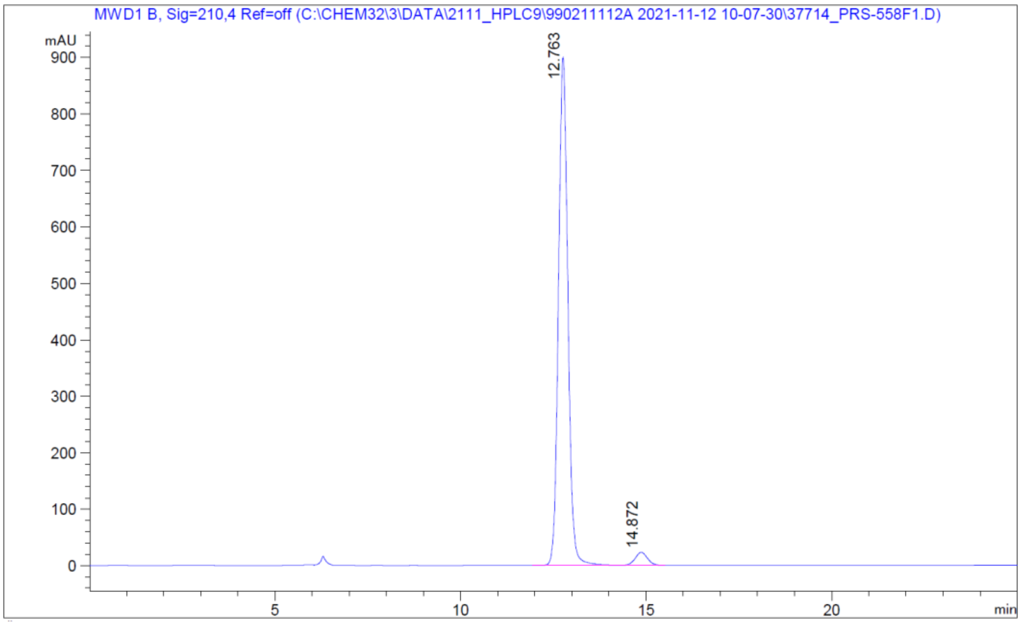

#	Meas. R	Pea	Width	Area	Height	Area %
1	12.763	BV	0.272	15905.613	899.543	96.818
2	14.872	VB	0.358	522.677	22.779	3.182

(*R*)-*N*-(2-(4-Methoxyphenyl)-3-phenylpropyl)-4-methylbenzenesulfonamide, 19n

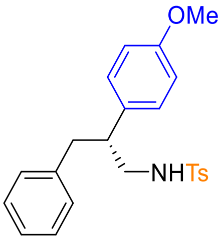

HPLC: Chiralpak IA, heptane:IPA 9:1, 0.5 mL/min, λ = 210 nm, t_R (*R*) = 35.1 min, t_R (*S*) = 38.2 min.

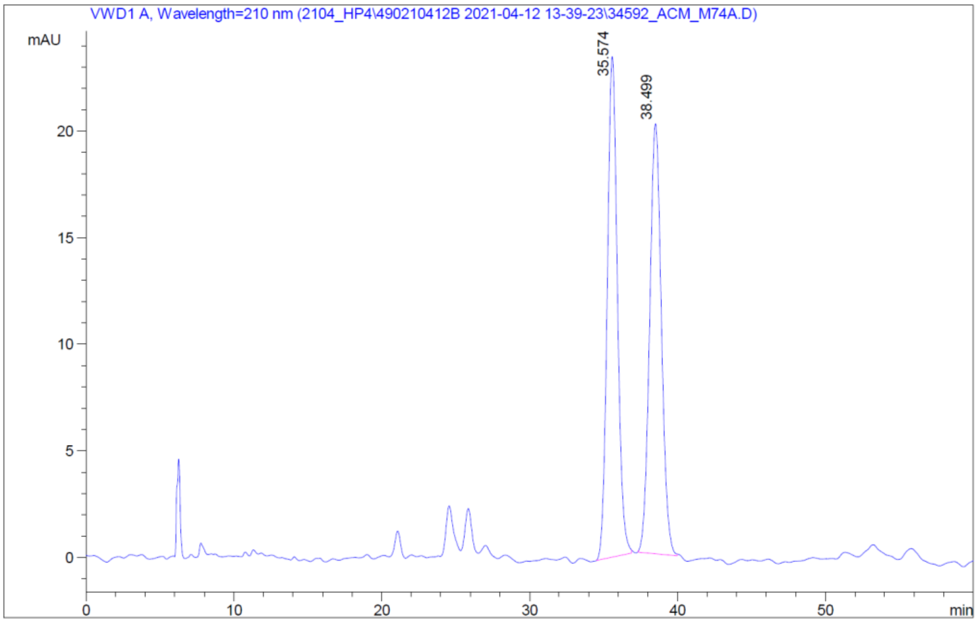

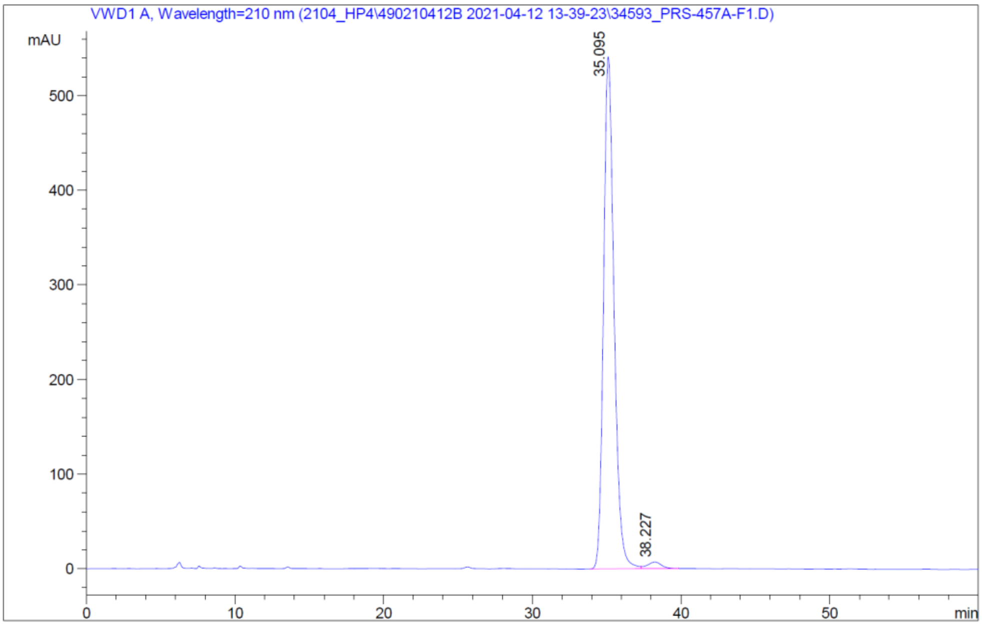

#	Meas. R	Pea	Width	Area	Height	Area %
1	35.095	BB	0.747	26572.695	541.596	98.248
2	38.227	BB	0.961	473.932	7.163	1.752

(*R*)-*N*-(2-(4-Chlorophenyl)-3-phenylpropyl)-4-methylbenzenesulfonamide, 19o

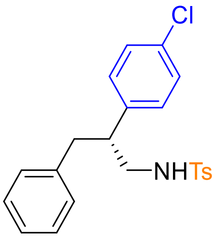

HPLC: Chiralpak IA, heptane:IPA 9:1, 0.5 mL/min, λ = 210 nm, t_R (*R*) = 29.1 min, t_R (*S*) = 31.5 min.

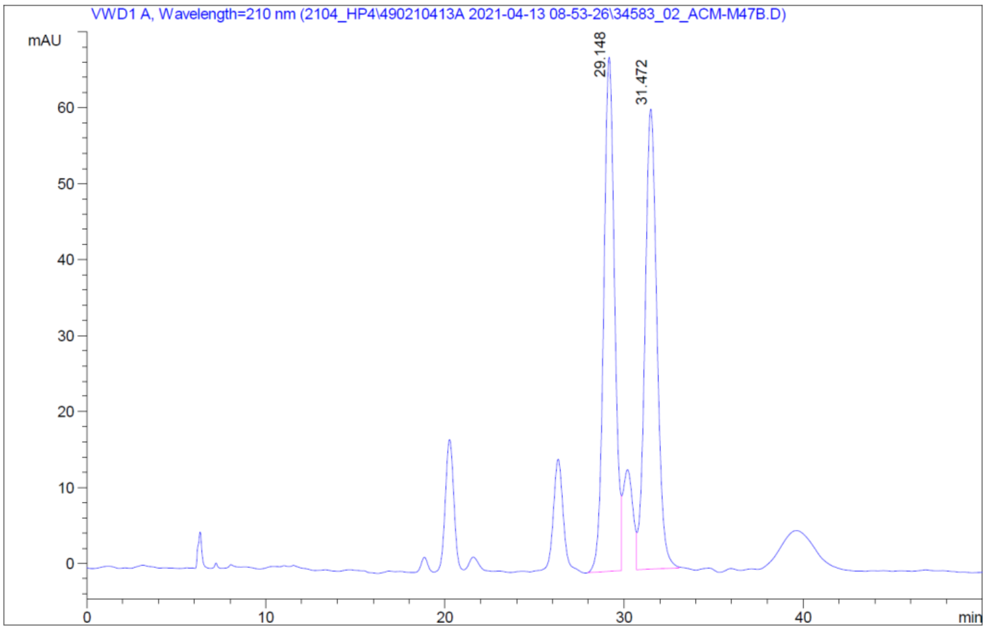

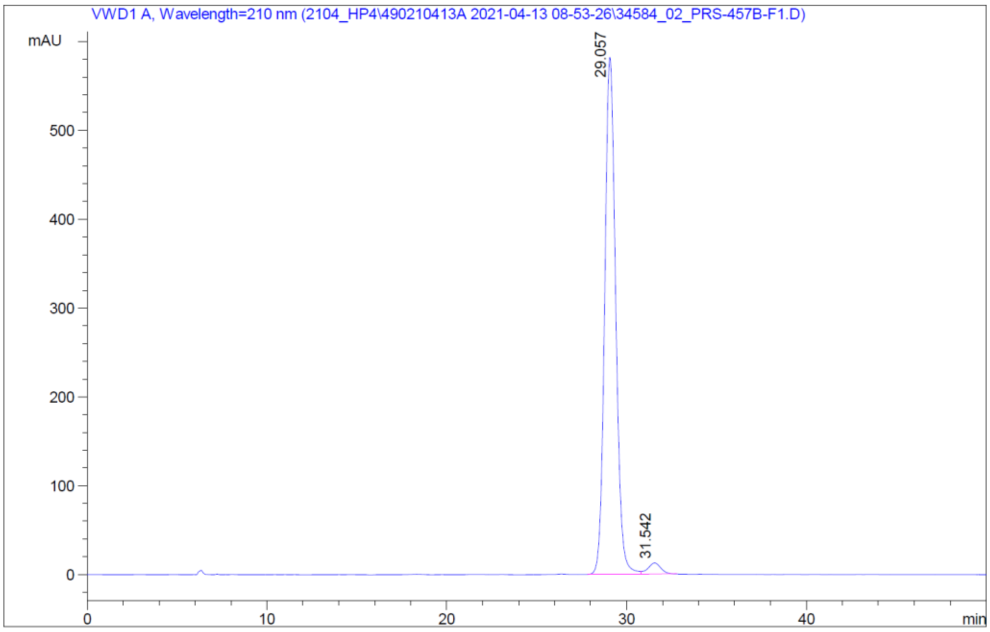

#	Meas. R	Pea	Width	Area	Height	Area %
1	29.057	BB	0.654	24659.762	582.248	97.508
2	31.542	BB	0.763	630.312	12.553	2.492

(R)-4-Methyl-N-(3-phenyl-2-(o-tolyl)propyl)benzenesulfonamide, 19p

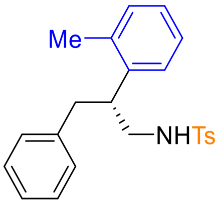

HPLC: Chiralpak IA, heptane:IPA 7:3, 0.5 mL/min, λ = 210 nm, t_R (R) = 11.0 min, t_R (S) = 12.6 min.

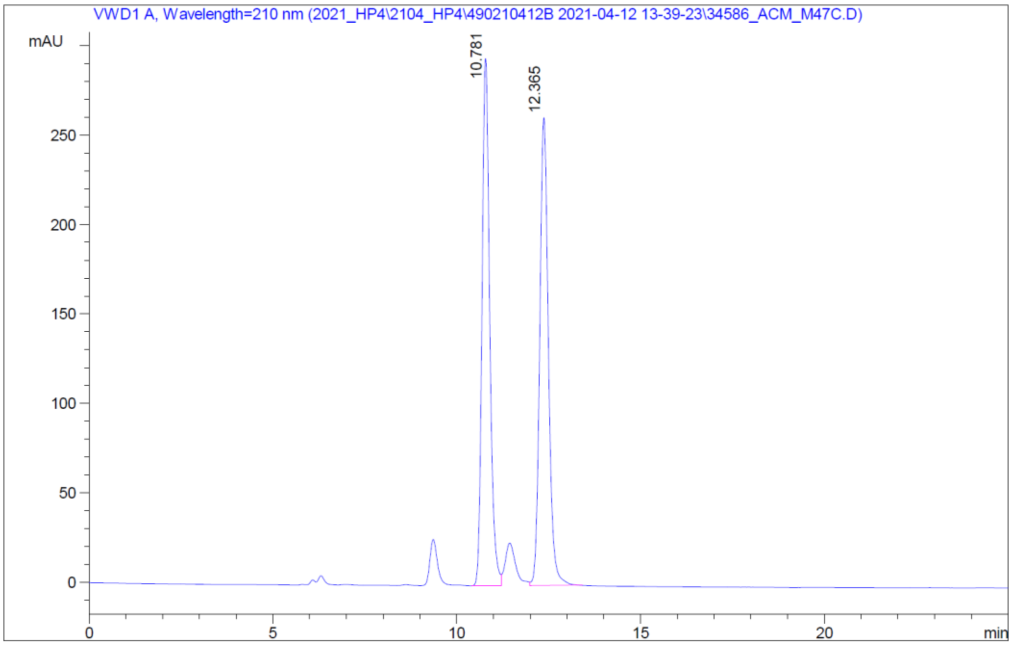

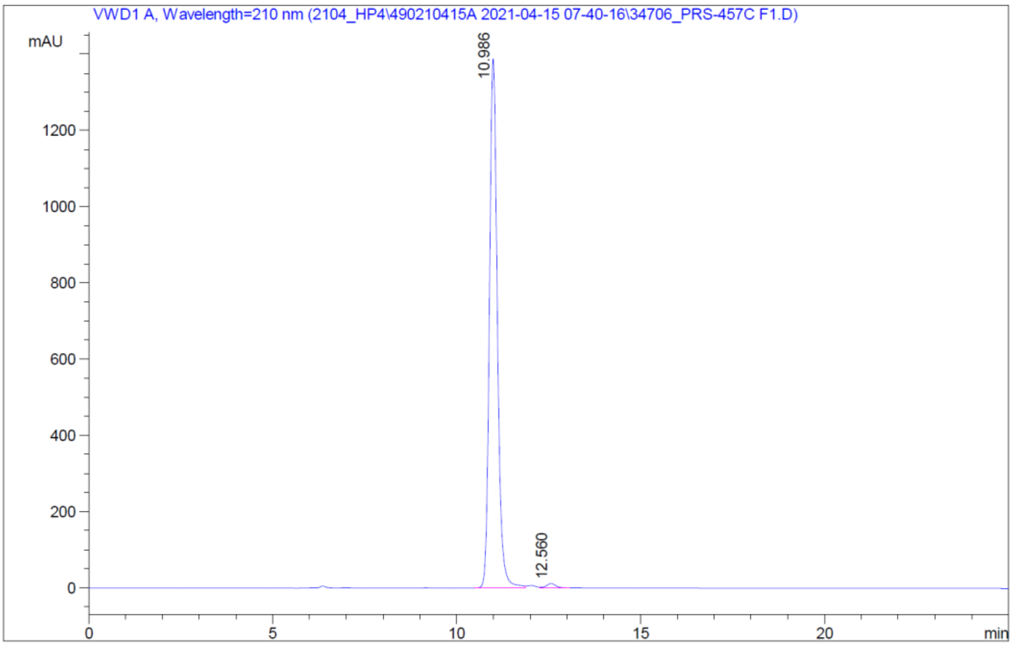

#	Meas. R	Pea	Width	Area	Height	Area %
1	10.986	MF	0.249	20756.172	1.389e3	99.003
2	12.560	FM	0.301	208.991	11.559	0.997
